# Supplementary material for: A general highly efficient synthesis of biocompatible rhodamine dyes and probes for live-cell multicolor nanoscopy
Source: Nat Commun. 2023 Mar 9;14:1306. doi: 10.1038/s41467-023-36913-2 (PMC9998615; doi:10.1038/s41467-023-36913-2)
Supplement: Supplementary file 1 — Supplementary Information [file 41467_2023_36913_MOESM1_ESM.pdf]

Supplementary information

A general highly efficient synthesis of biocompatible rhodamine dyes and probes for live-cell multicolor nanoscopy

Jonas Bucevičius[1], Rūta Gerasimaitė[1], Kamila A. Kiszka[2], Shalini Pradhan[1], Georgij Kostiuk[1], Tanja Koenen[2] and Gražvydas Lukinavičius[1]*.

[1] Chromatin Labeling and Imaging group, Department of NanoBiophotonics, Max Planck Institute for Multidisciplinary Sciences, Am Fassberg 11, 37077 Göttingen, Germany.

[2] Department of NanoBiophotonics, Max Planck Institute for Multidisciplinary Sciences, Am Fassberg 11, 37077 Göttingen, Germany.

*Corresponding author's e-mail: grazvydas.lukinavicius@mpinat.mpg.de

Contents

Supplementary Figures ... 4
Supplementary Figure 1. ... 4
Supplementary Figure 2. ... 5
Supplementary Figure 3 ... 6
Supplementary Figure 4 ... 7
Supplementary Figure 5 ... 8
Supplementary Figure 6 ... 9
Supplementary Figure 7 ... 10
Supplementary Figure 8 ... 11
Supplementary Figure 9 ... 12
Supplementary Figure 10 ... 13
Supplementary Figure 11 ... 14
Supplementary Figure 12 ... 15
Supplementary Figure 13 ... 16
Supplementary Figure 14 ... 17
Supplementary Figure 15 ... 18
Supplementary Figure 16 ... 19
Supplementary Figure 17 ... 20
Supplementary Figure 18 ... 21
Supplementary Figure 19 ... 22
Supplementary Figure 20 ... 23
Supplementary Figure 21 ... 24
Supplementary Figure 22 ... 25
Supplementary Figure 23 ... 26
Supplementary Figure 24 ... 27
Supplementary Figure 25 ... 28
Supplementary Figure 26 ... 29
Supplementary Figure 27 ... 30
Supplementary Figure 28 ... 31
Supplementary Tables ... 32
Supplementary Table 1 ... 32
Supplementary Table 2 ... 33
Supplementary Table 3 ... 34

Supplementary Table 4 35
Supplementary Table 5 36
Supplementary Table 6 37
Supplementary Methods 38
Determination of absolute Quantum yields 38
Determination of fluorescence lifetimes 38
Reaction yield determination by absorption spectroscopy 38
Plasmid construction 39
Maintenance and preparation of cells 39
Preparation and staining of mouse living primary cells and tissues 40
Primary neuronal isolation and staining 40
Tissue isolation and staining 41
Cell cycle analysis by imaging flow cytometry and EC_{50} determination 41
Wide field microscope and imaging parameters 42
Supplementary Table 7 42
Spinning disk confocal microscope and imaging parameters 42
Supplementary Table 8 42
Confocal/STED microscopes and imaging parameters 43
Supplementary Table 9 43
4-color time-lapse movies 45
Processing and visualization of the acquired images 45
General chemical experimental information and synthesis methods 47
Copies of NMR spectra 176
Supplementary References 285

Supplementary Figures

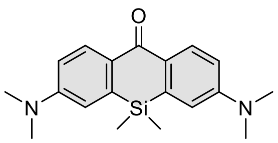
K2

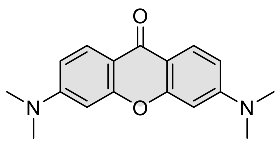
K3

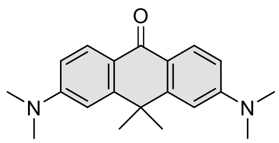
K4

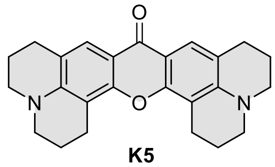
K5

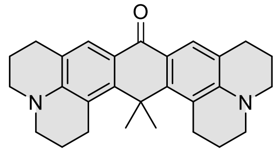
K6

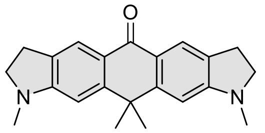
K7

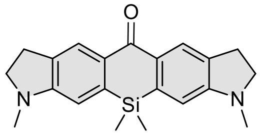
K8

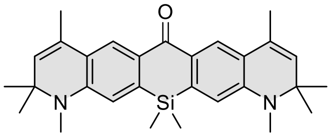
K9

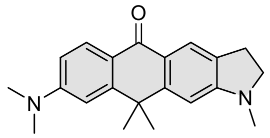
K10

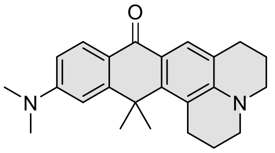
K11

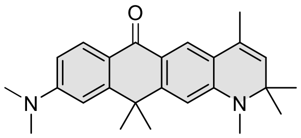
K12

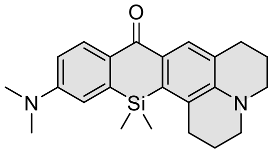
K13

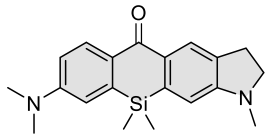
K14

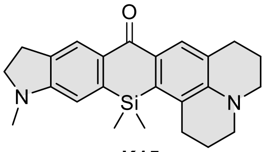
K15

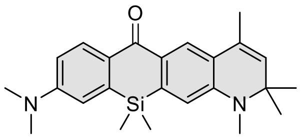
K16

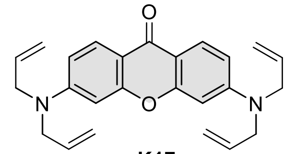
K17

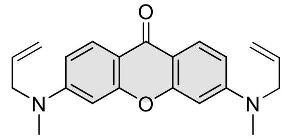
K18

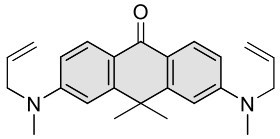
K19

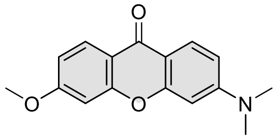
K20

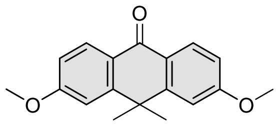
K21

Supplementary Figure 1. Structures of ketones used in the reaction scope studies.

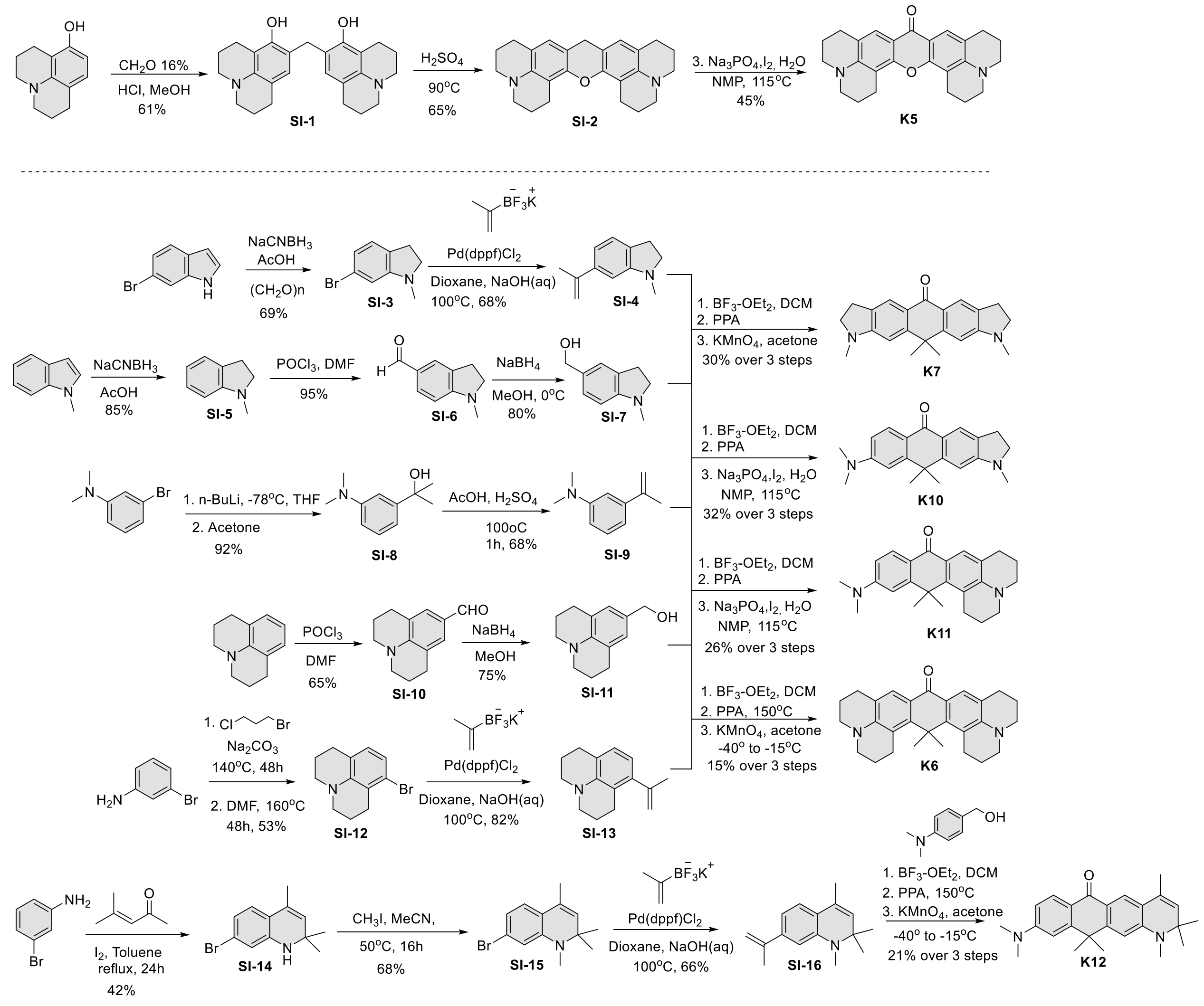

Supplementary Figure 2. Synthetic routes to symmetrical (**K5**-**K7**) and unsymmetrical (**K10**-**K12**) xanthones with carbon bridging atom.

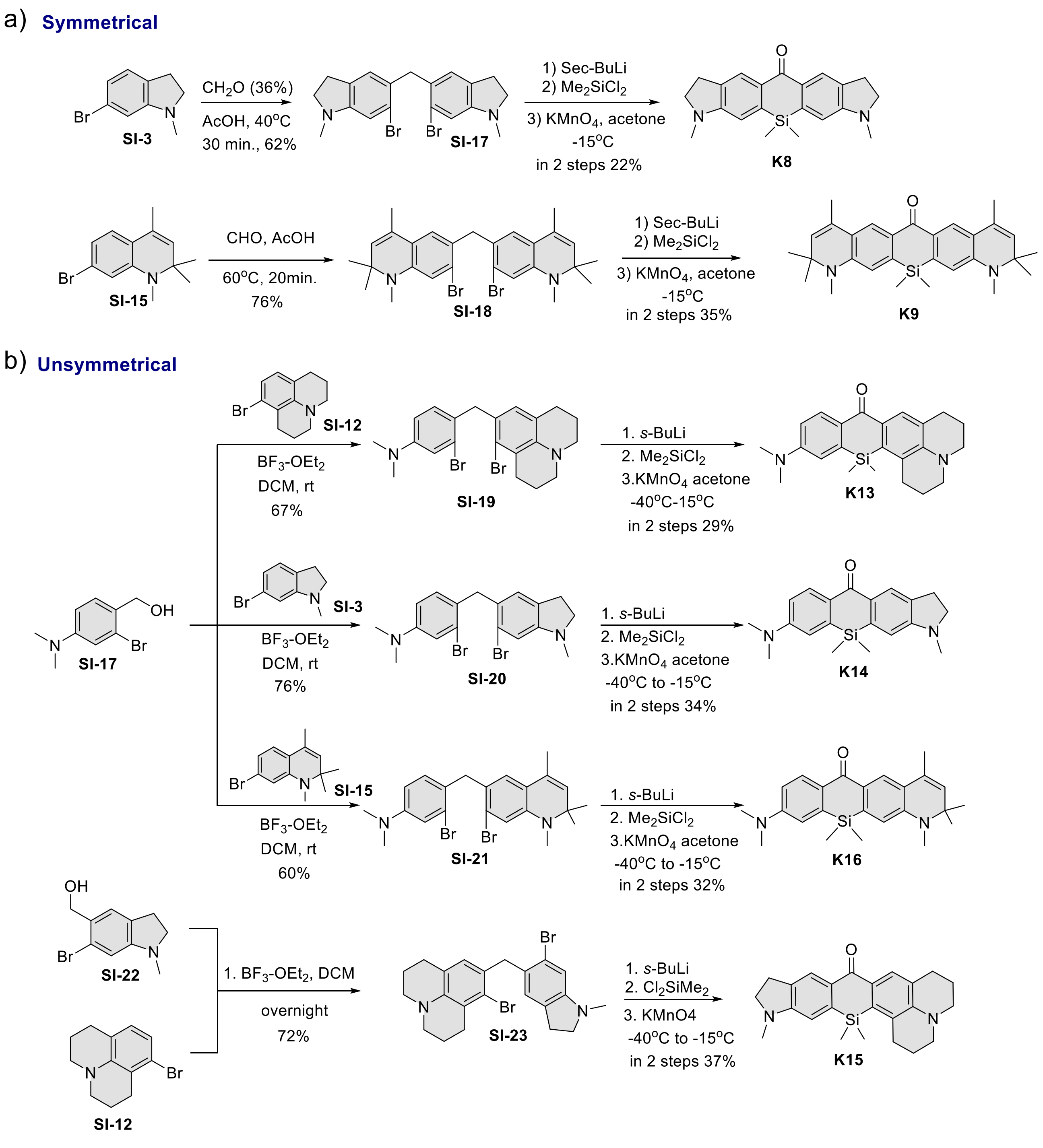

Supplementary Figure 3. **a** Synthetic routes to symmetrical (**K8-K9**). **b** Synthetic routes to unsymmetrical (**K13**-**K16**) xanthones with silicon bridging atom.

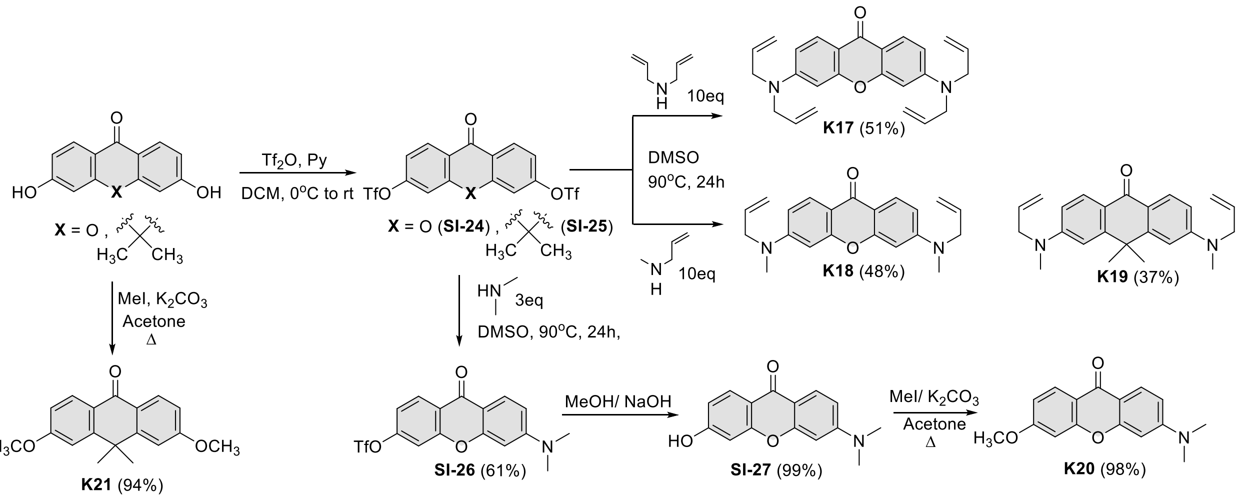

Supplementary Figure 4. Synthetic routes to ketones **K17**-**K21**.

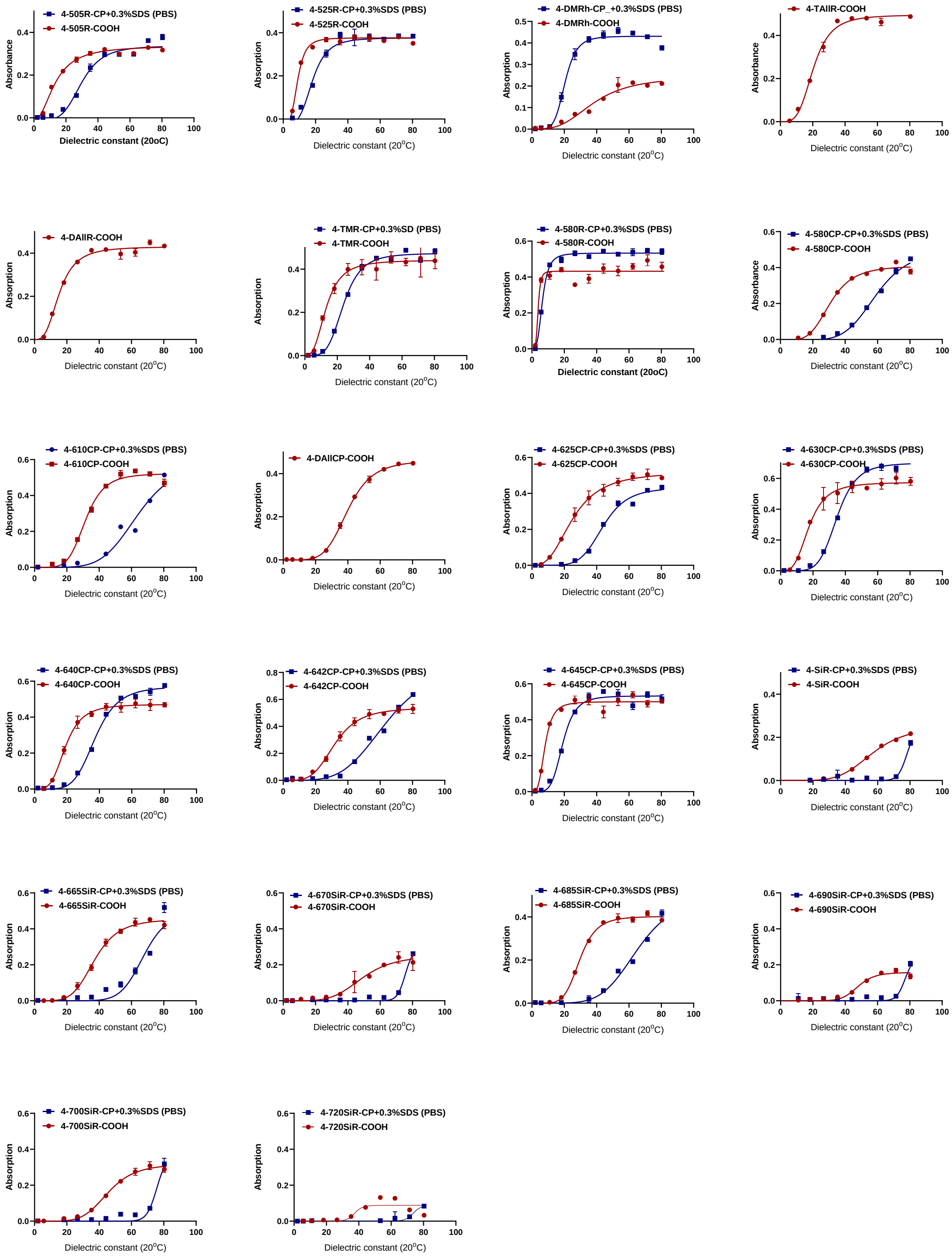

Supplementary Figure 5. Absorption of the free dyes and respective 4-(benzyloxy)-2-chloropyrimidine (CP) derivatives in 1, 4-dioxane-water mixtures. Plots show absorbance of free dye (red) or CP derivative (blue) at λ_{max} versus dielectric constant of 1,4-dioxane-water mixtures. D_{50} values, obtained by fitting the data to EC_{50} dose-response equation, are listed in supplementary table S2. Data points are presented as mean ± s.d. of three independently repeated experiments (N=3). When titrating the probes, SDS was included to suppress their aggregation.

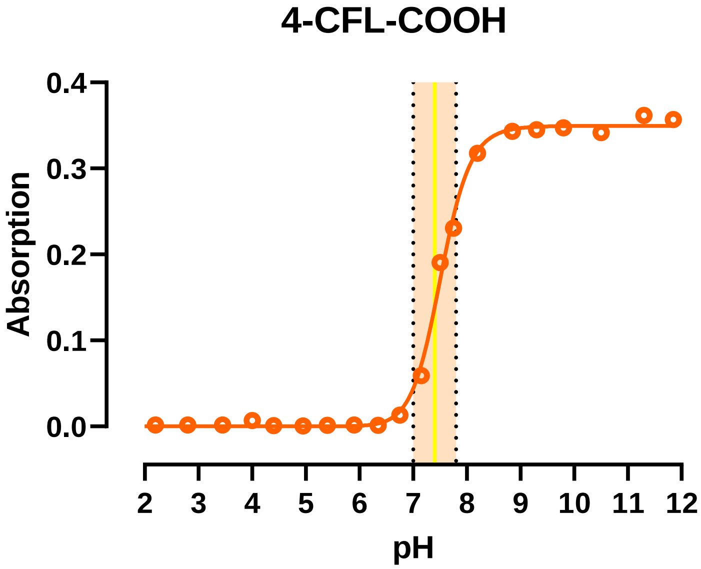

Supplementary Figure 6. Absorbance at λ_{max} versus pH for **4-CFL-COOH**. Dashed lines mark interval pH 7.0-7.8 and yellow line indicates pH = 7.4. Measurements were performed in universal buffer for UV spectrophotometry which was prepared by taking 50mL solution consisting of 0.1 M citric acid (21.01 g/l), 0.1M KH_2PO_4 (13.61 g/l), 0.1 M sodium tetraborate (19.07 g/l), 0.1 M Tris (12.11 g/l), 0.1 M KCl (7.46 g/l) and adjusting the pH to the required values by adding x mL of 0.4 M HCl or 0.4 M NaOH, followed by the dilution to 200 ml. Buffers in the range of pH 2-12 were prepared in ~0.5 increments.

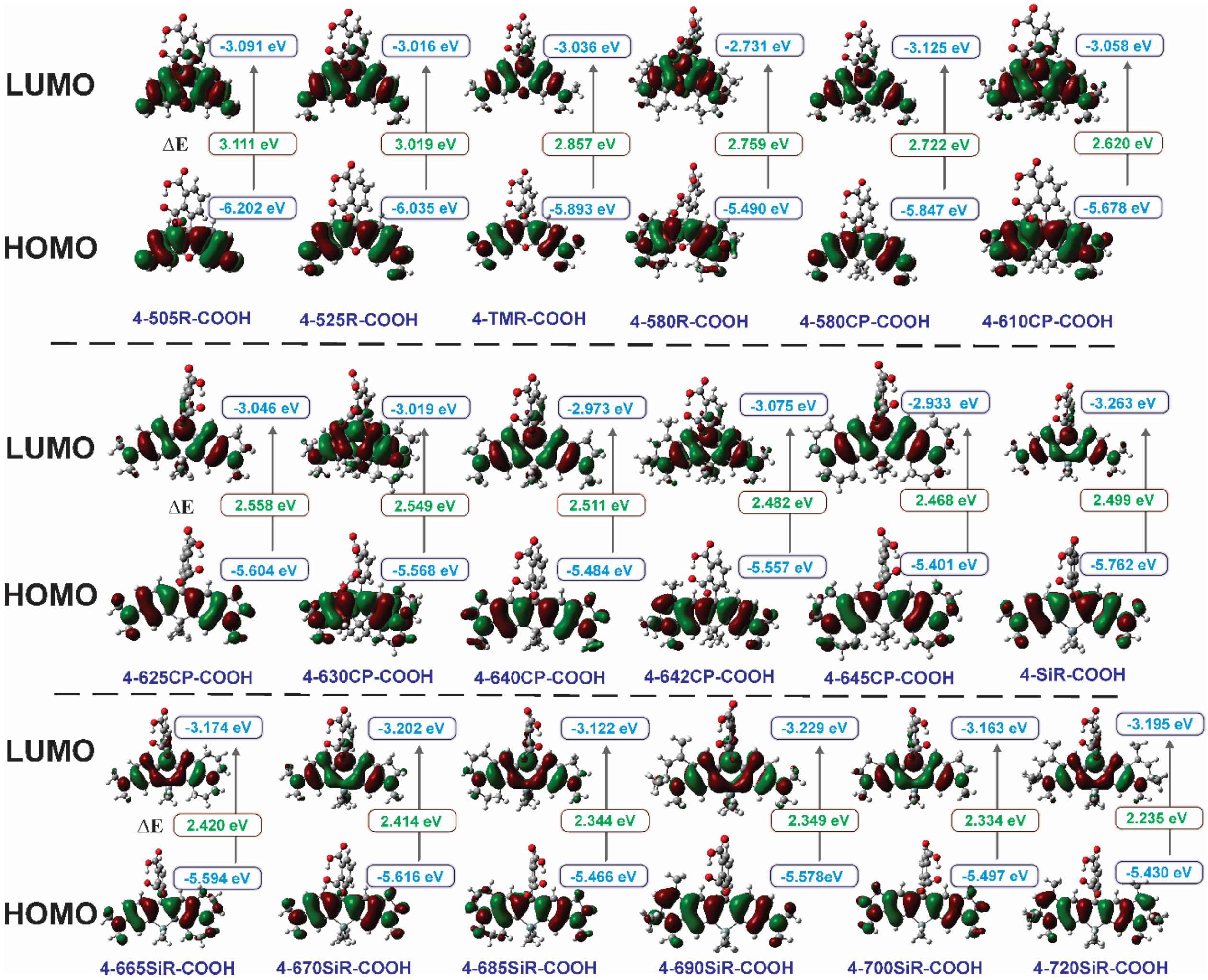

Supplementary Figure 7. Isodensity surface plots of frontier molecular orbitals of the synthesized 4-carboxyrhodamine dyes obtained by DFT calculations at B3LYP/6-311++G(d,p)/IEFPCM (H_2O) level of theory.

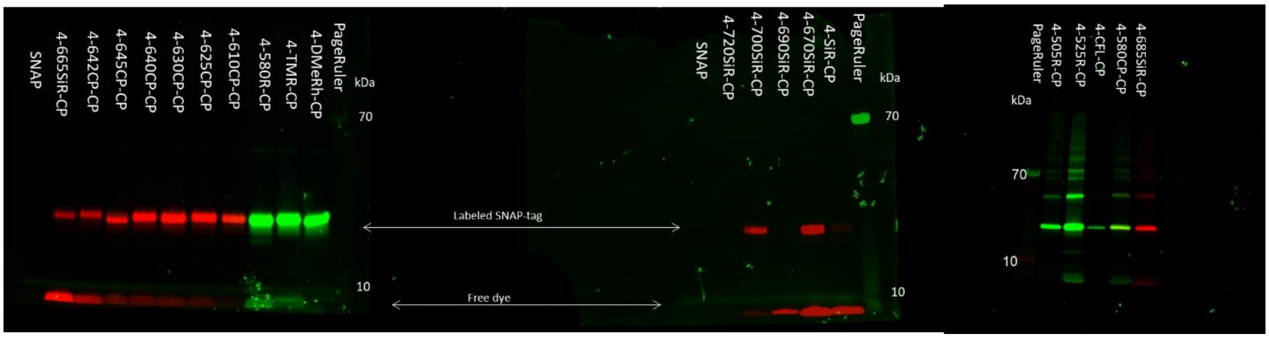

(Fluorescence)

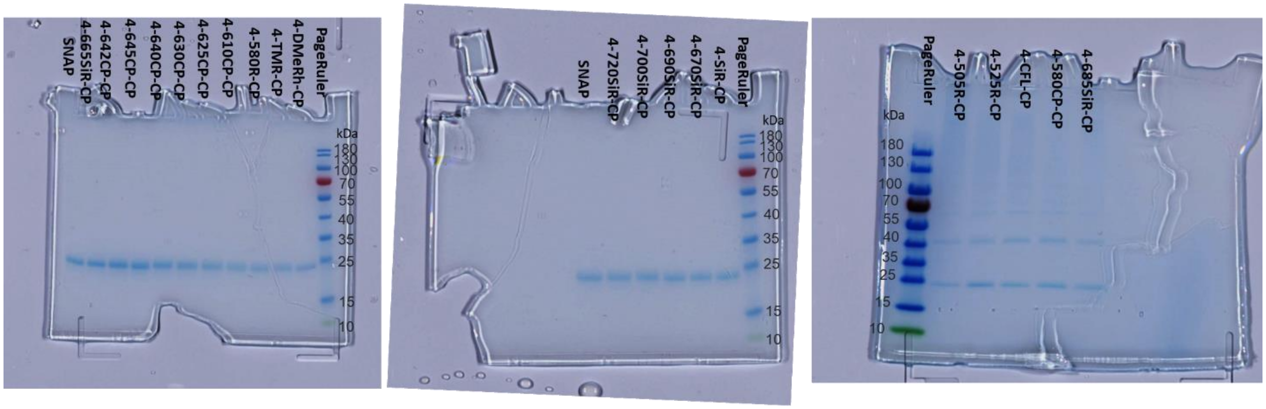

(Coomassie stain)

Supplementary Figure 8. SDS-PAGE gel images of SNAP tag protein labeled with 4-(benzyloxy)-2-chloropyrimidine (CP) derivatives **27**-**46**. Incubated for 3h at 37°C. Data acquired on Amersham Imager 600 RGB. Each experiment was repeated independently 2 times with similar results.

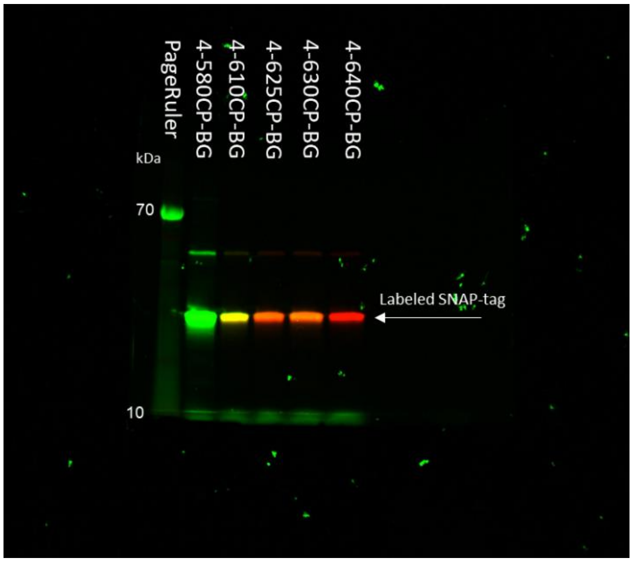

(Fluorescence)

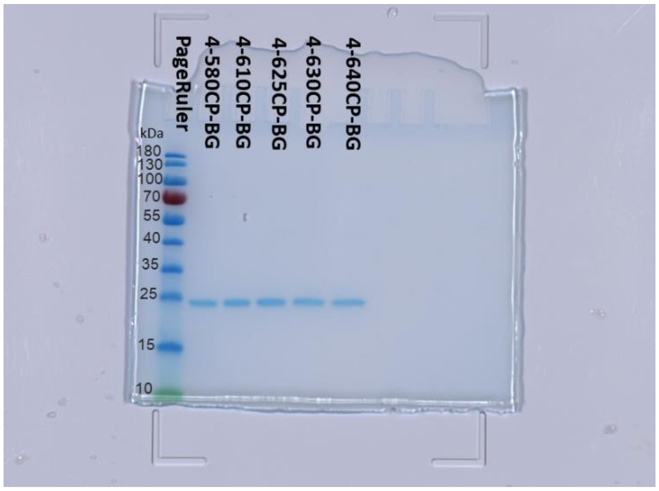

(Coomassie stain)

Supplementary Figure 9. SDS-PAGE gel images of SNAP tag protein labeled with O6-benzylguanine based (BG) SNAP-tag substrates **105**-**109**. Incubated for 3h at 37°C. Data acquired on Amersham Imager 600 RGB. Each experiment was repeated independently 2 times with similar results.

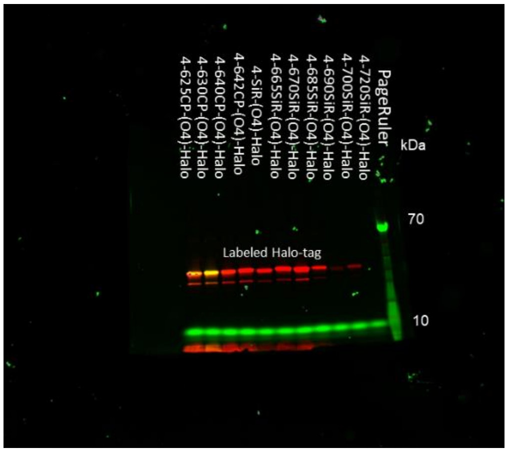

(Fluorescence

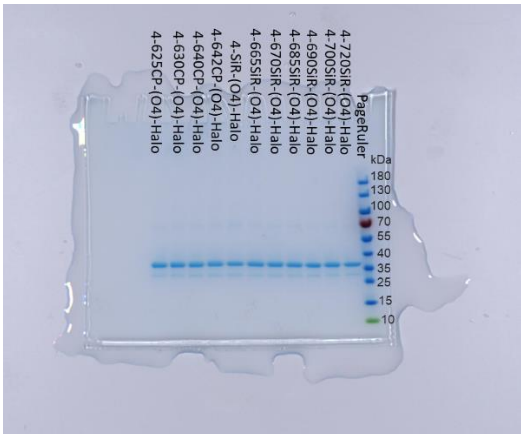

(Coomassie stain)

Supplementary Figure 10. SDS-PAGE gel images of Halo-tag protein labeled with Halo-tag substrates **68**-**79** with a SNAP tag protein. Incubated for 3h at 37°C. Data acquired on Amersham Imager 600 RGB. Each experiment was repeated independently 2 times with similar results.

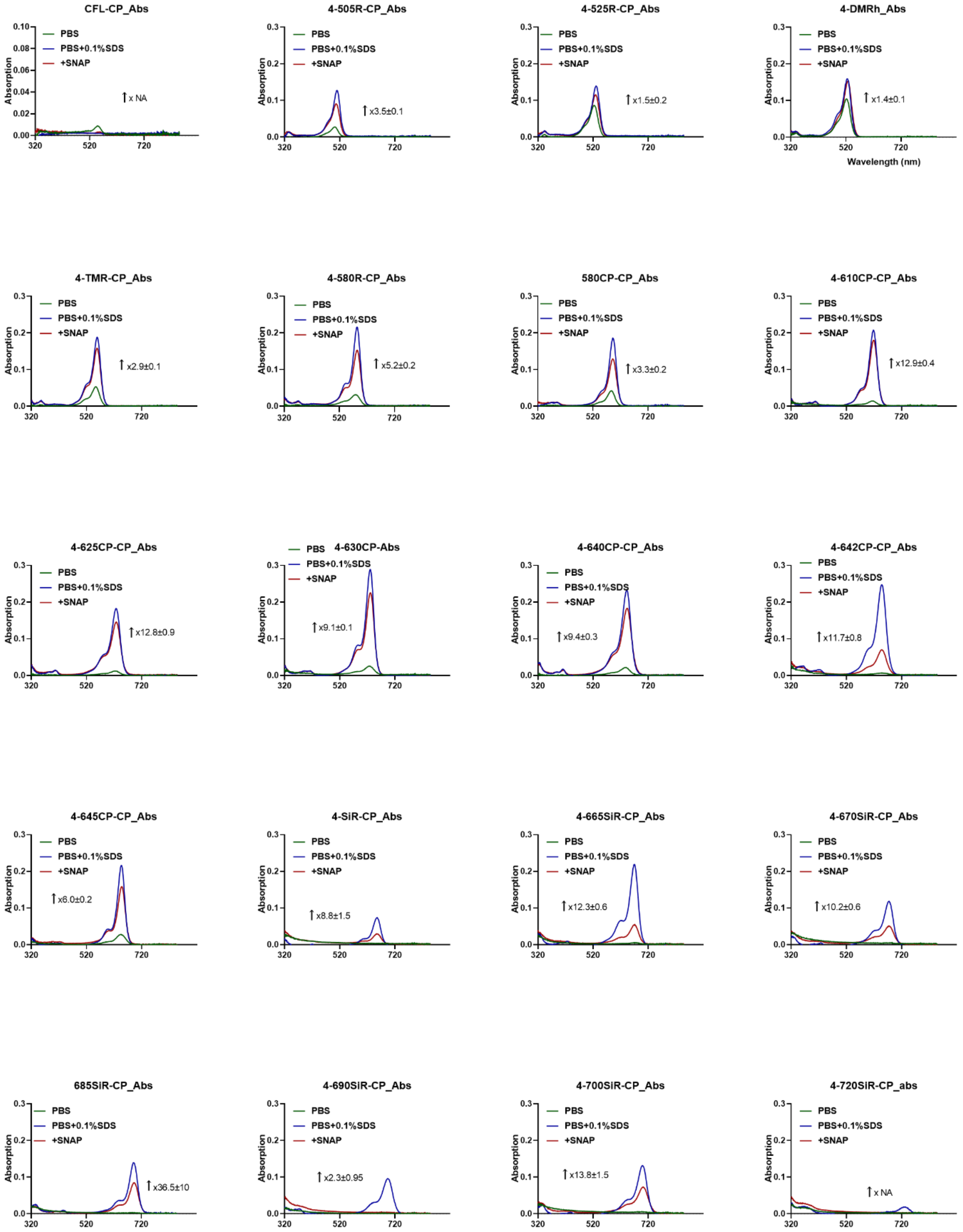

Supplementary Figure 11. Absorbance spectra of the chloropyrimidine (CP) based SNAP-tag substrates. Spectra were recorded after incubating 2.5 µM probes with 5 µM SNAP-tag protein (red), 0.1% SDS (blue) or without additives in PBS (green) at 37 °C for 3 h to ensure complete reaction. Spectra are presented as averages of three independently repeated experiments (N=3).

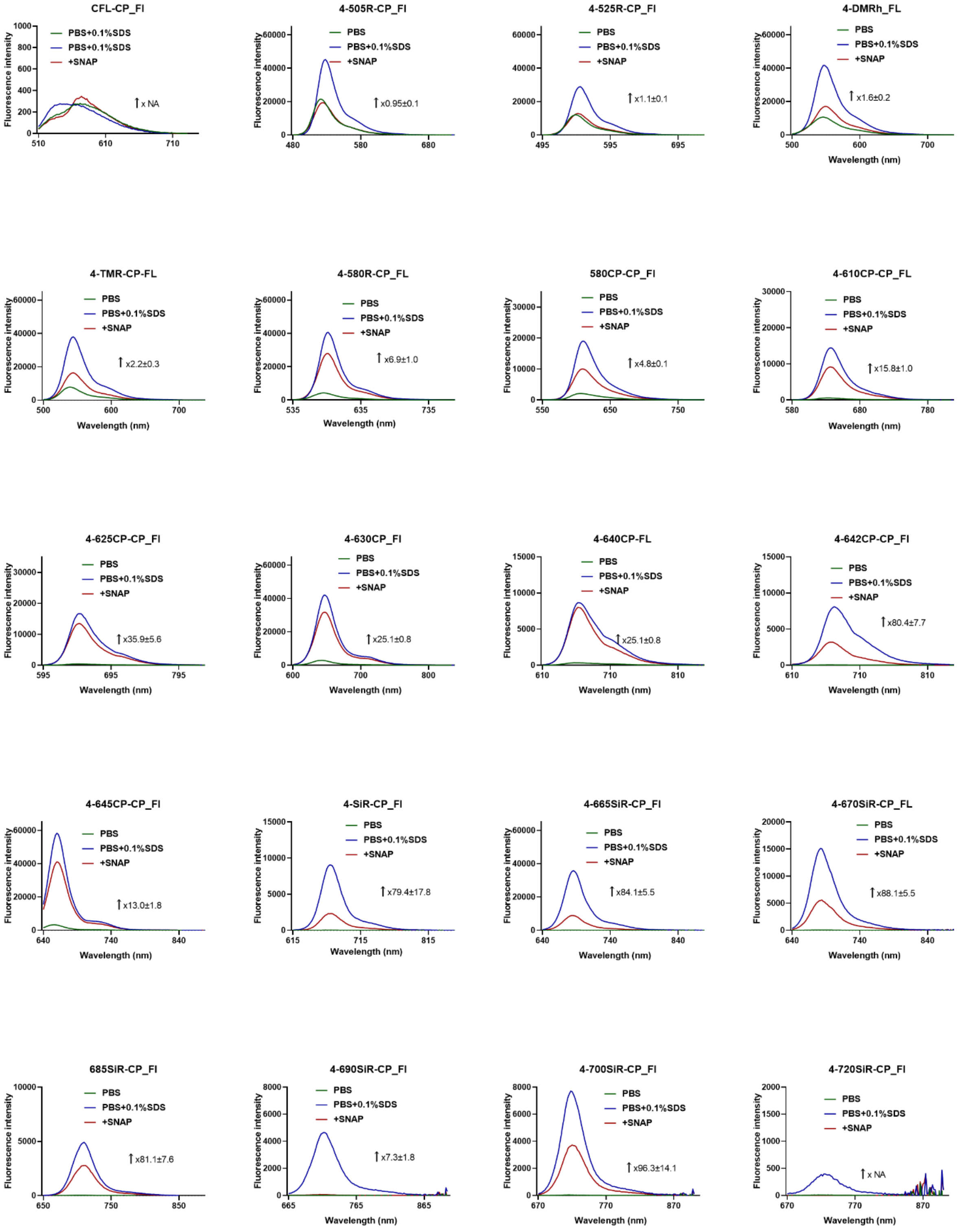

Supplementary Figure 12. Fluorescence spectra of the chloropyrimidine (CP) based SNAP-tag substrates. Spectra were recorded after incubating 2.5 µM probes with 5 µM SNAP-tag protein (red), 0.1% SDS (blue) or without additives in PBS (green) at 37 °C for 3 h to ensure complete reaction. Spectra are presented as averages of three independently repeated experiments (N=3).

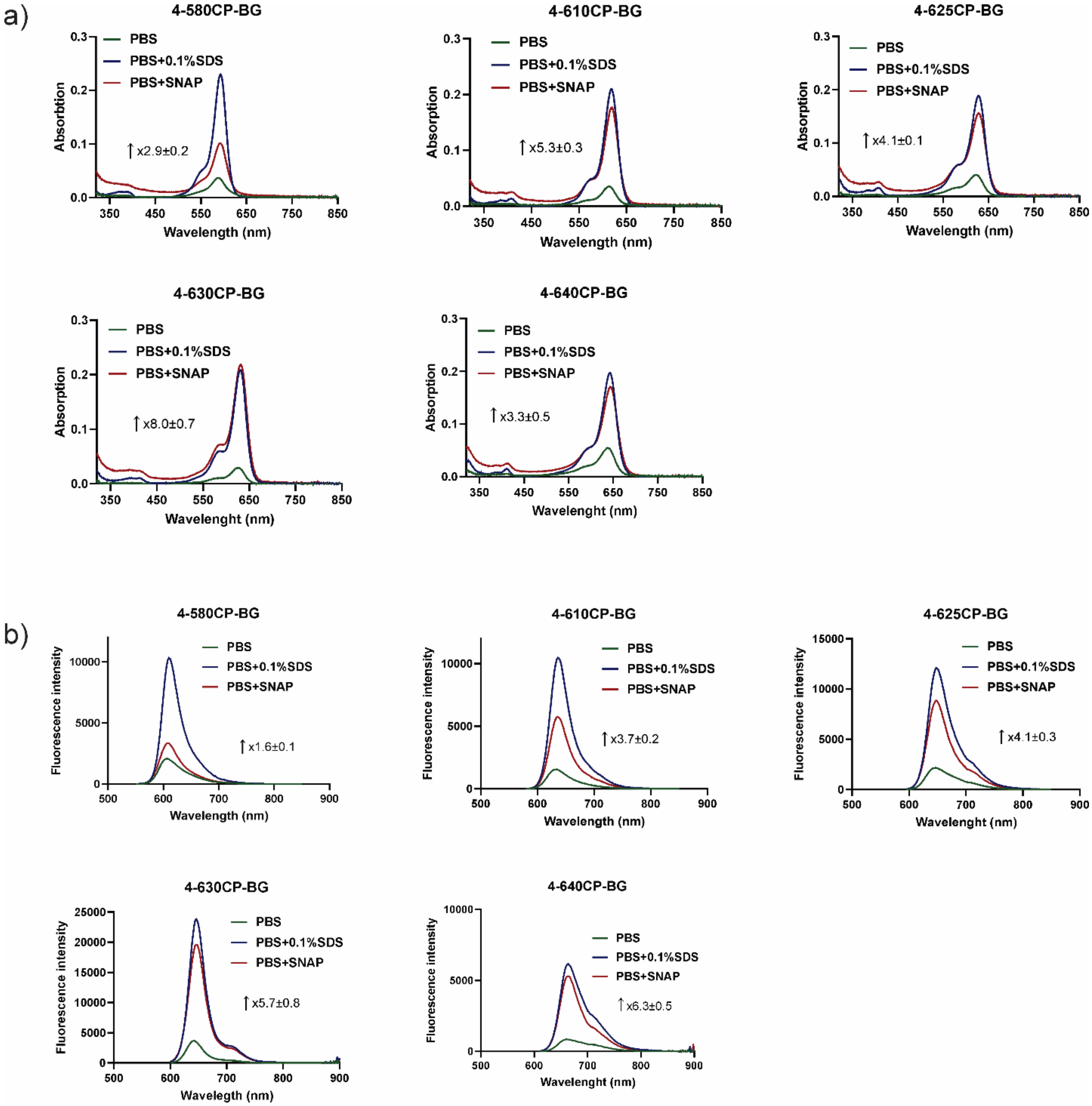

Supplementary Figure 13. Absorbance and fluorescence spectra of the O6-benzylguanine based (BG) SNAP-tag substrates. Absorbance (a) and fluorescence (b) spectra were recorded after incubating 2.5 µM probes with 5 µM Halo-tag protein (red), 0.1% SDS (blue) or without additives in PBS (green) at 37 °C for 3 h to ensure complete reaction. Spectra are presented as averages of three independently repeated experiments (N=3).

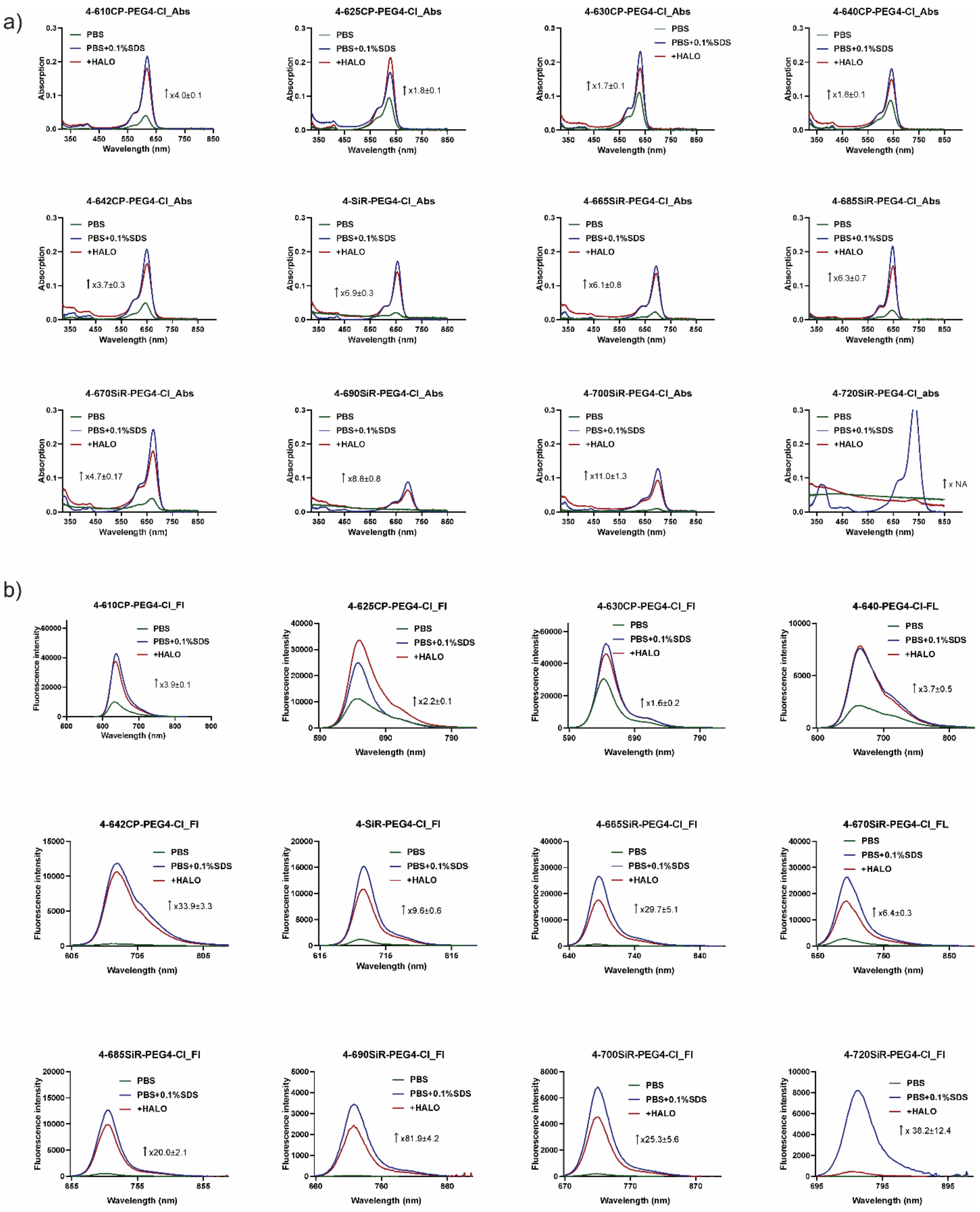

Supplementary Figure 14. Absorbance and fluorescence spectra of the Halo-tag substrates. Absorbance (a) and fluorescence (b) spectra were recorded after incubating 2.5 μM probes with 5 μM Halo-tag protein (red), 0.1% SDS (blue) or without additives in PBS (green) at 37 °C for 3 h to ensure complete reaction. Spectra are presented as averages of three independently repeated experiments (N=3).

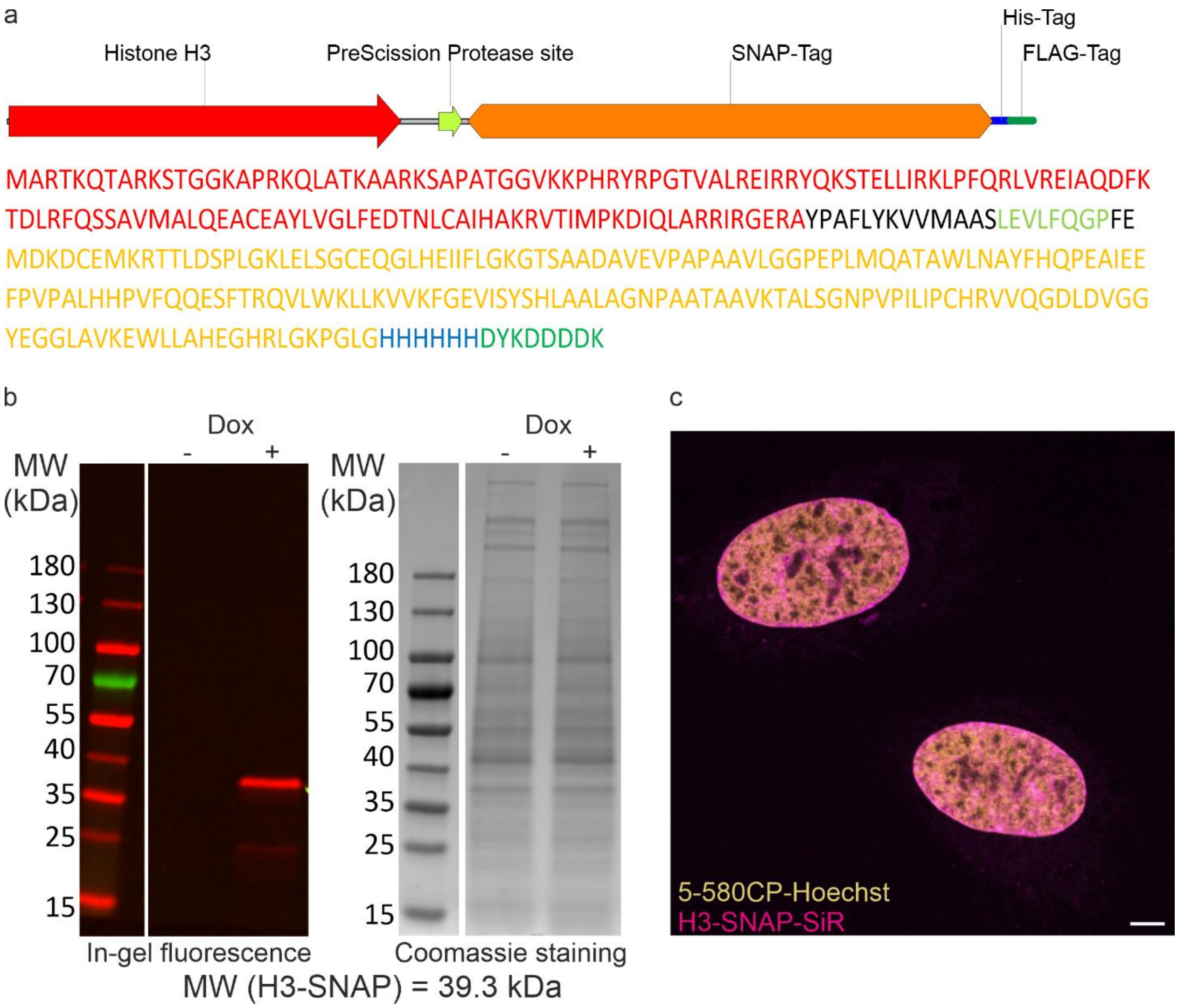

Supplementary Figure 15. Properties U-2 OS cell line expressing SNAP-tagged Histone H3. a. amino acid sequence of H3-SNAP protein. b. Inducible expression of H3-SNAP protein in U-2 OS cells. SNAP-tag labelled with 1 µM SiR-SNAP (NEB) substrate for 1h at 37°C in CelLytic™ M (Sigma-Aldrich) cell lysis reagent solution. The obtained protein sample supplemented with sample loading buffer (Bio-rad) and SDS-PAGE was performed. Protein expression induced with 0.1 µg/ml doxycycline for 24h before the lysis. SDS-PAGE experiment was performed once. c. Co-localization of H3-SNAP protein and nuclear DNA stain 5-580CP-Hoechst. Cells were stained with 100 nM SiR-SNAP (NEB) and 100 nM 5-580CP-Hoecshst in DMEM growth medium containing 10% FBS at 37 °C for 1 h, washed once with HBSS and imaged in HBSS on Abberior Expert line. Protein expression induced with 0.1 µg/ml doxycycline for 24h before imaging. Scale bar 10 µm. See supplementary materials and methods section for further details on imaging settings.

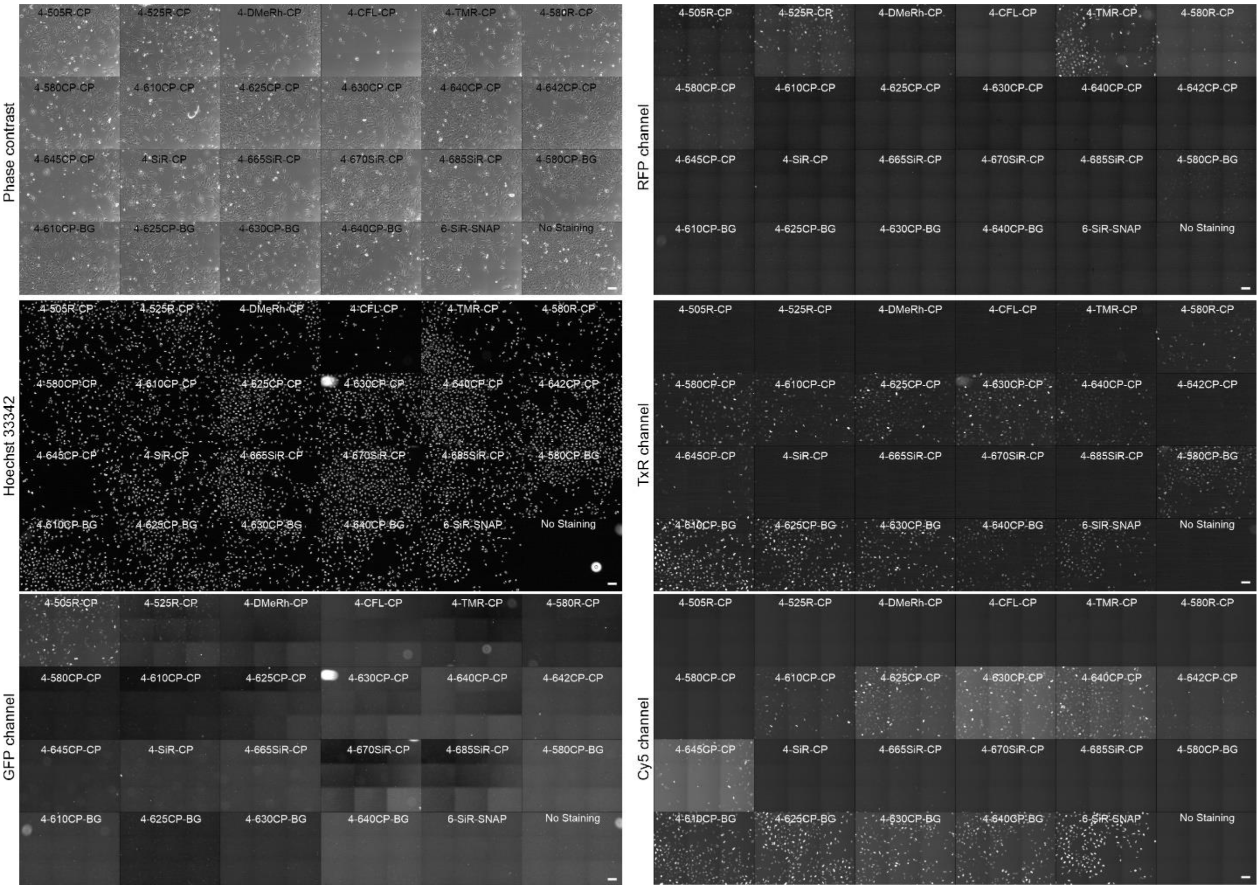

Supplementary Figure 16. Wide field fluorescence microscopy images of living U-2 OS cells expressing histone H3-SNAP protein. Cells were stained with a series of different SNAP-tag substrates at 100 nM concentration and 0.1 µg/ml Hoechst 33342 in DMEM growth medium containing 10% FBS at 37 °C for 1 h, washed once with HBSS and imaged in HBSS on Biotek Lionheart FX automated microscope. Note, DMEM growth medium shows strong autofluorescence in GFP channel and was replaced with HBSS to visualize probes in fluorescent in GFP channel. Scale bars 100 µm. See supplementary materials and methods section for further details on imaging settings.

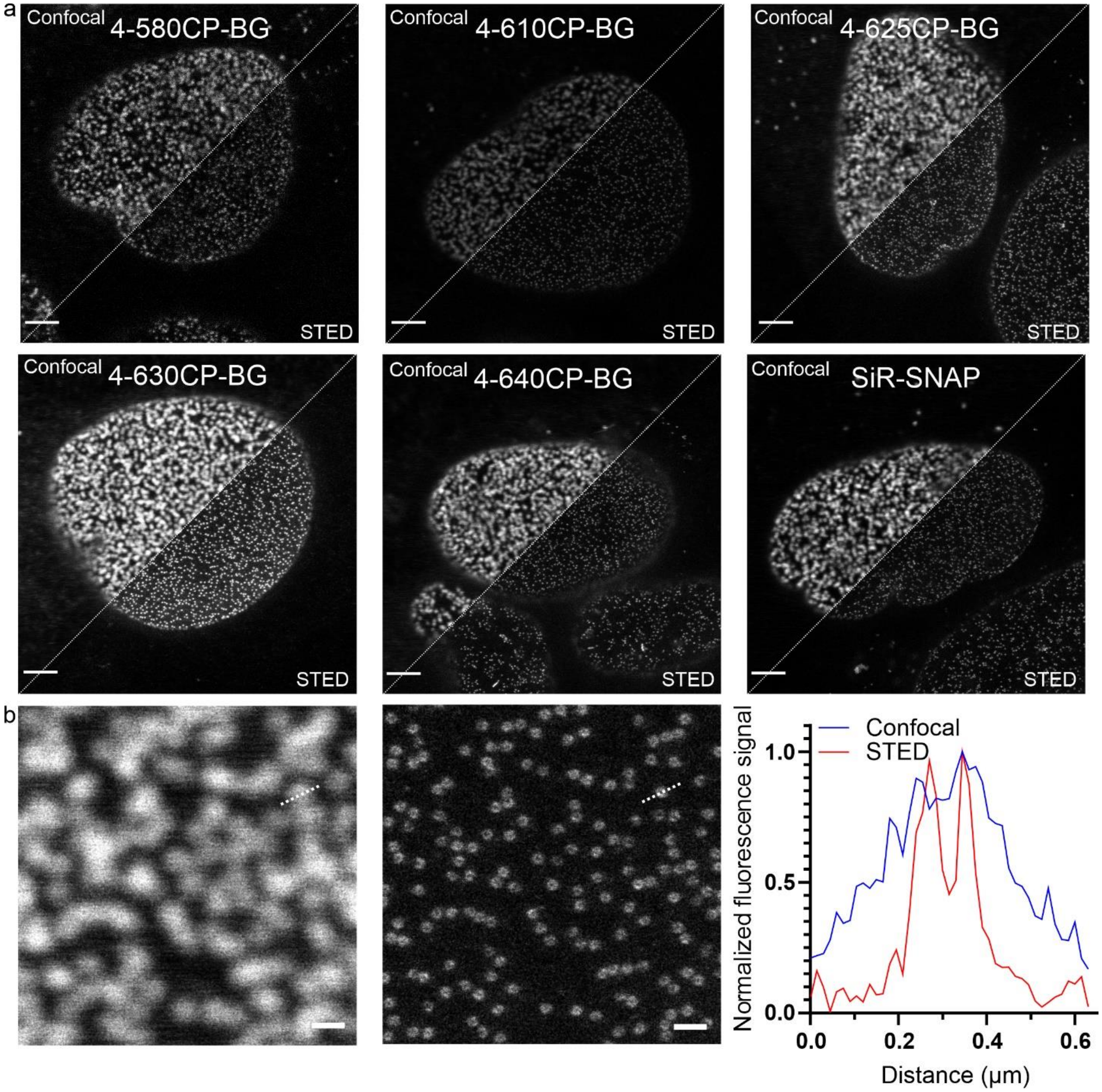

Supplementary Figure 17. STED imaging of nucleopores tagged with SNAP-tag. Performance of the new O6-Benzylguanine (BG) derivatives. a. Comparison of confocal and STED images of living U-2 OS cells expressing Nup96-SNAP stained with BG derivatives. Scale bars: 3 µm. b. Confocal (left) and STED (center) zoomed images of U-2 OS cells expressing Nup96-SNAP stained with **4-630CP-BG** probe. Line profile (right) showing normalized fluorescence signal along the dashed line in the images. Scale bar 0.5µm. Living cells were incubated with 100nM of the indicated probe in DMEM containing 10% FBS at 37 °C for 1 h, washed once with HBSS and imaged in DMEM containing 10% FBS. Images were acquired on Abberior Expert line scanning microscope equipped with 775 nm depletion laser. See supplementary materials and methods section for further details on imaging settings.

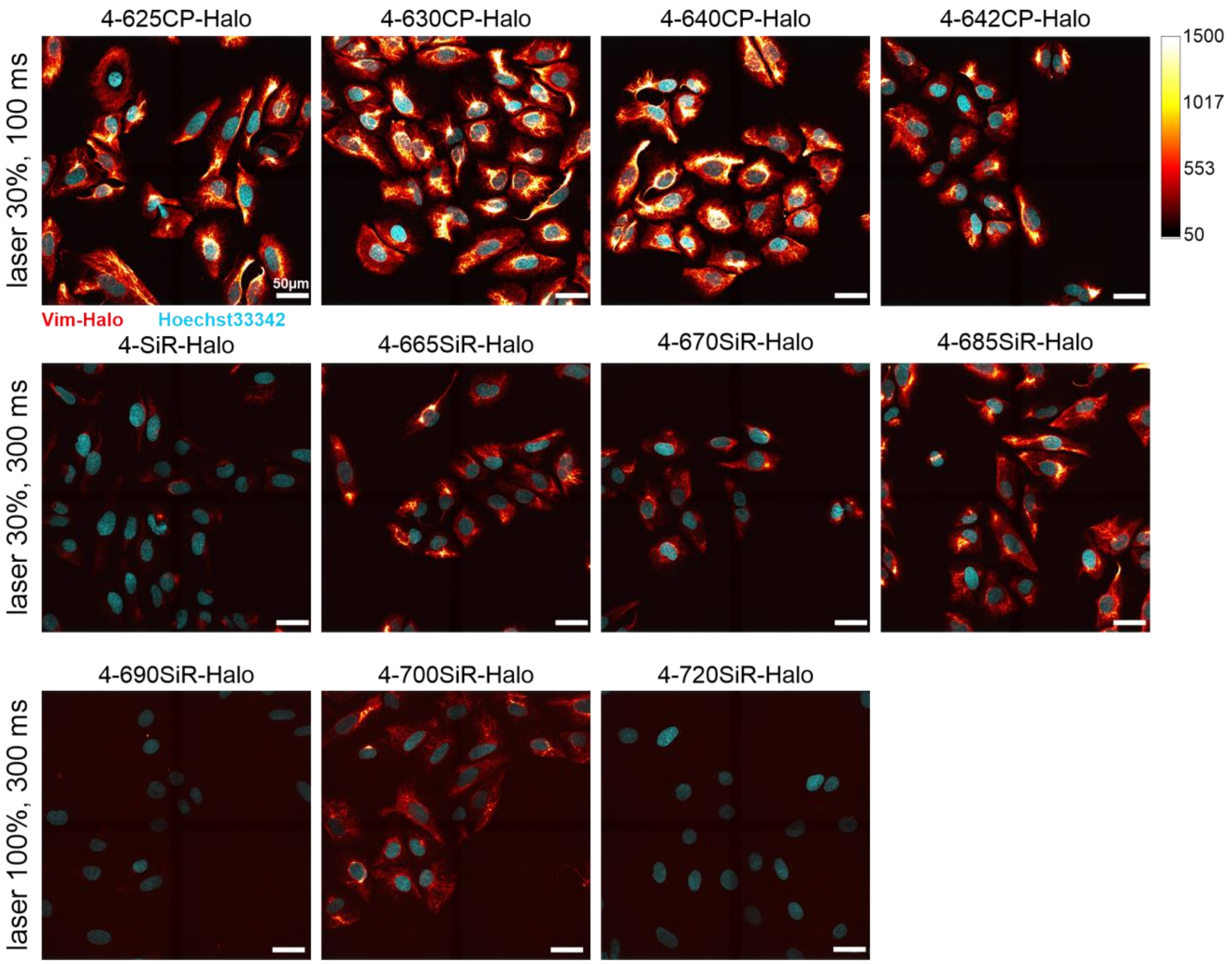

Supplementary Figure 18. Staining of U-2 OS vimentin-Halo cells with new HaloTag substrates. Cells were incubated with 100 nM dye in DMEM medium containing 10% FBS for 1h at 37°C, washed once with culture medium followed by wash with HBSS and subsequently imaged in culture medium on a spinning disk confocal microscope, using 640 nm laser for excitation and 665 nm long pass emission filter. Four fields of view were acquired as z-stacks with 200 nm step size and were merged with VisiView software. Max intensity projections are shown. Due to the different spectra of the dyes, the images had to be acquired with different settings, which are indicated in each row on the left. Note, that the observed staining intensities are only relevant for this particular imaging set-up and do not allow direct comparison of probe performance. Scale bars: 50 µm. See methods section for further details on imaging settings.

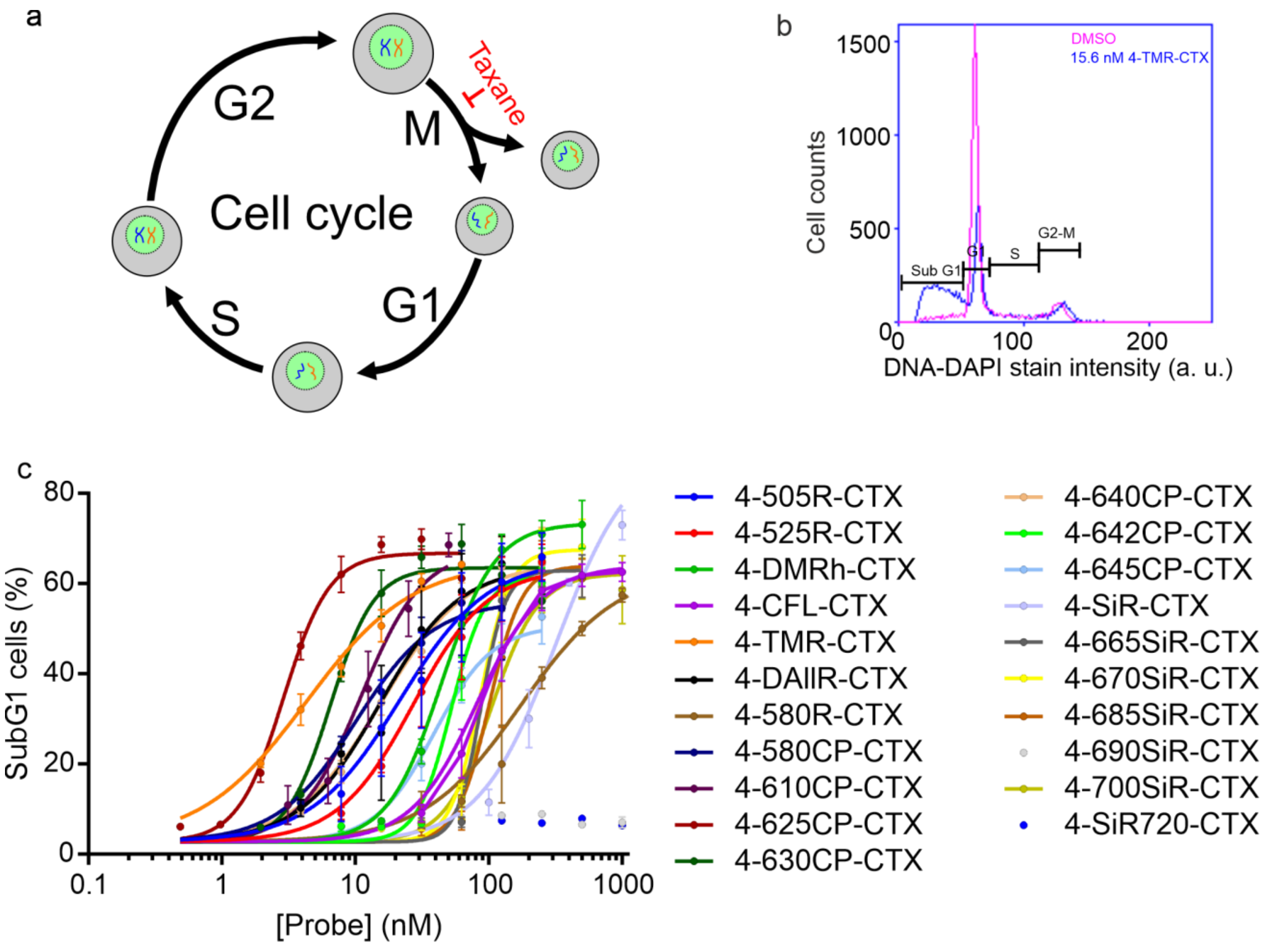

Supplementary Figure 19. Cell cycle perturbations in HeLa cells induced by fluorescent tubulin probes. a. Cytotoxicity of taxanes results from the inhibition of the cell cycle at the stage of mitosis (M). b. Representative histogram of DNA content distribution in HeLa cells treated with DMSO or 15.6 nM **4-TMR-CTX** for 24 h. The cell cycle phases are identified by the amount of DNA per cell. c. Accumulation of subG1 phase HeLa cells upon treatment with tubulin probes. Data are presented as means with standard deviations (N = 3 independent experiments, n > 9000 cells per experiment). Data were fitted to the dose response curve to obtain EC_{50}.

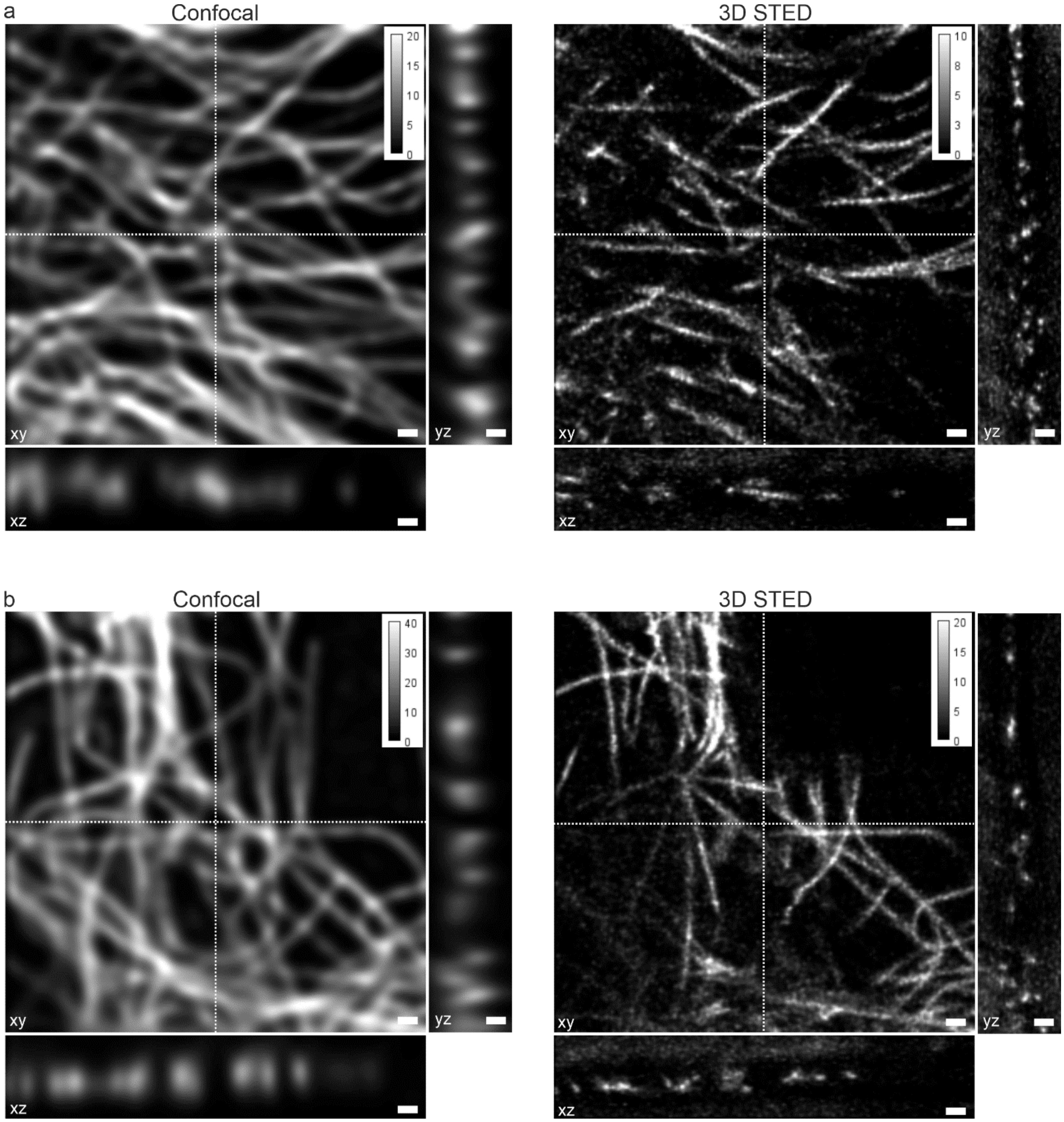

Supplementary Figure 20. Deconvolved confocal and 3D STED images of microtubules in living human fibroblasts stained for 1h at 37°C with a) 10 nM and b) 100nM **4-630CP-CTX** probe in the growth DMEM medium and imaged subsequently under no-wash conditions. Deconvolution performed using SVI Huygens software. Dashed white line indicates the position of xy, xz and yz sections. Voxel size: 40 x 40 x 40 nm. Scale bars: 0.5 µm. See supplementary materials and methods section for further details on imaging settings.

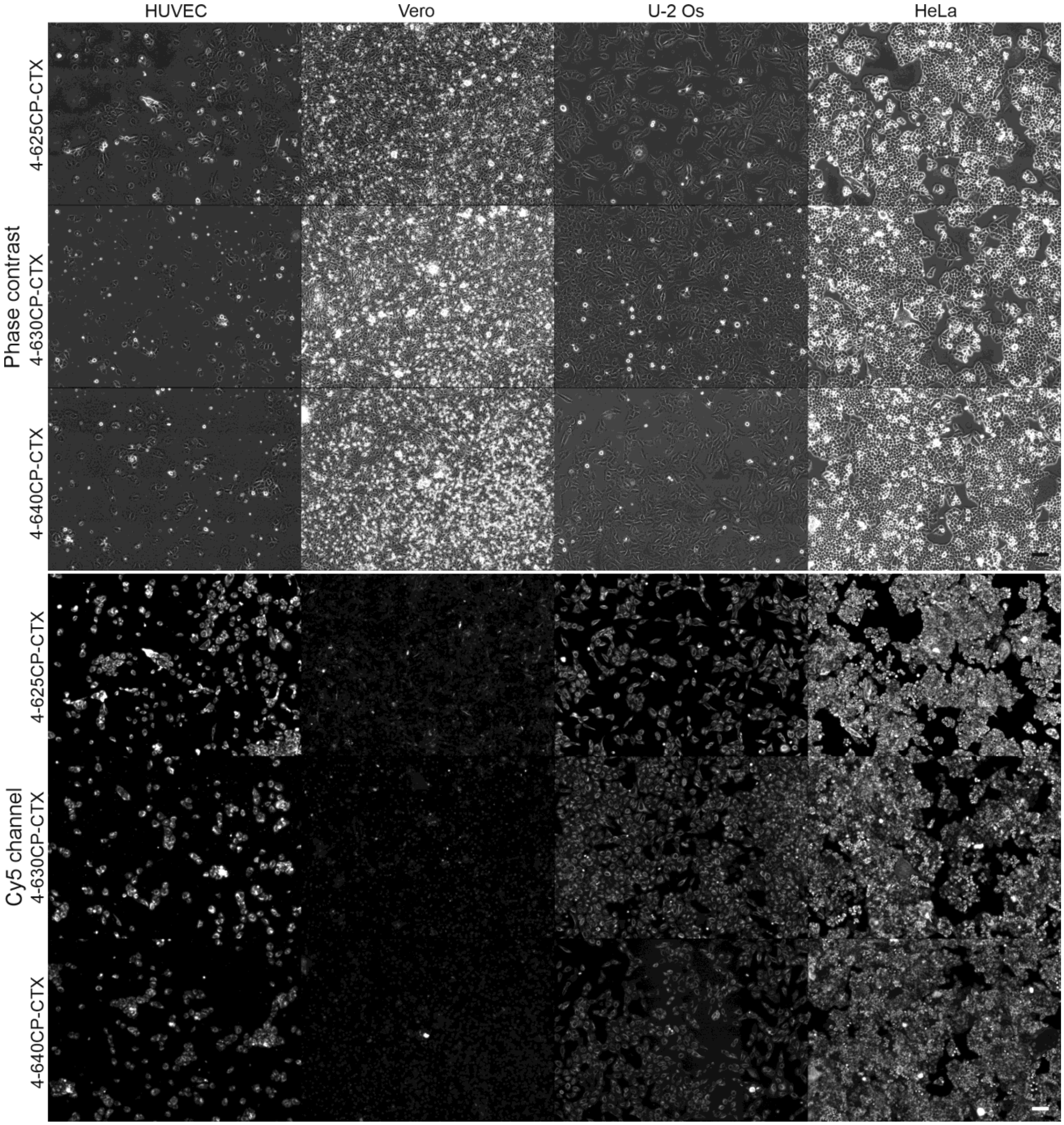

Supplementary Figure 21. Wide field fluorescence microscopy images of HUVEC, Vero, U-2 OS and HeLa cells stained with 100 nM **4-625CP-CTX**, **4-630CP-CTX** and **4-640CP-CTX** tubulin probes. Cells were stained in DMEM growth medium containing 10% FBS at 37 °C for 1 h, washed once with HBSS and imaged in DMEM growth medium containing 10% FBS on Biotek Lionheart FX automated microscope. Scale bars 100 µm. See methods section for further details on imaging settings. Note, that staining of Vero cells is the weakest due to high efflux activity of membrane pumps.

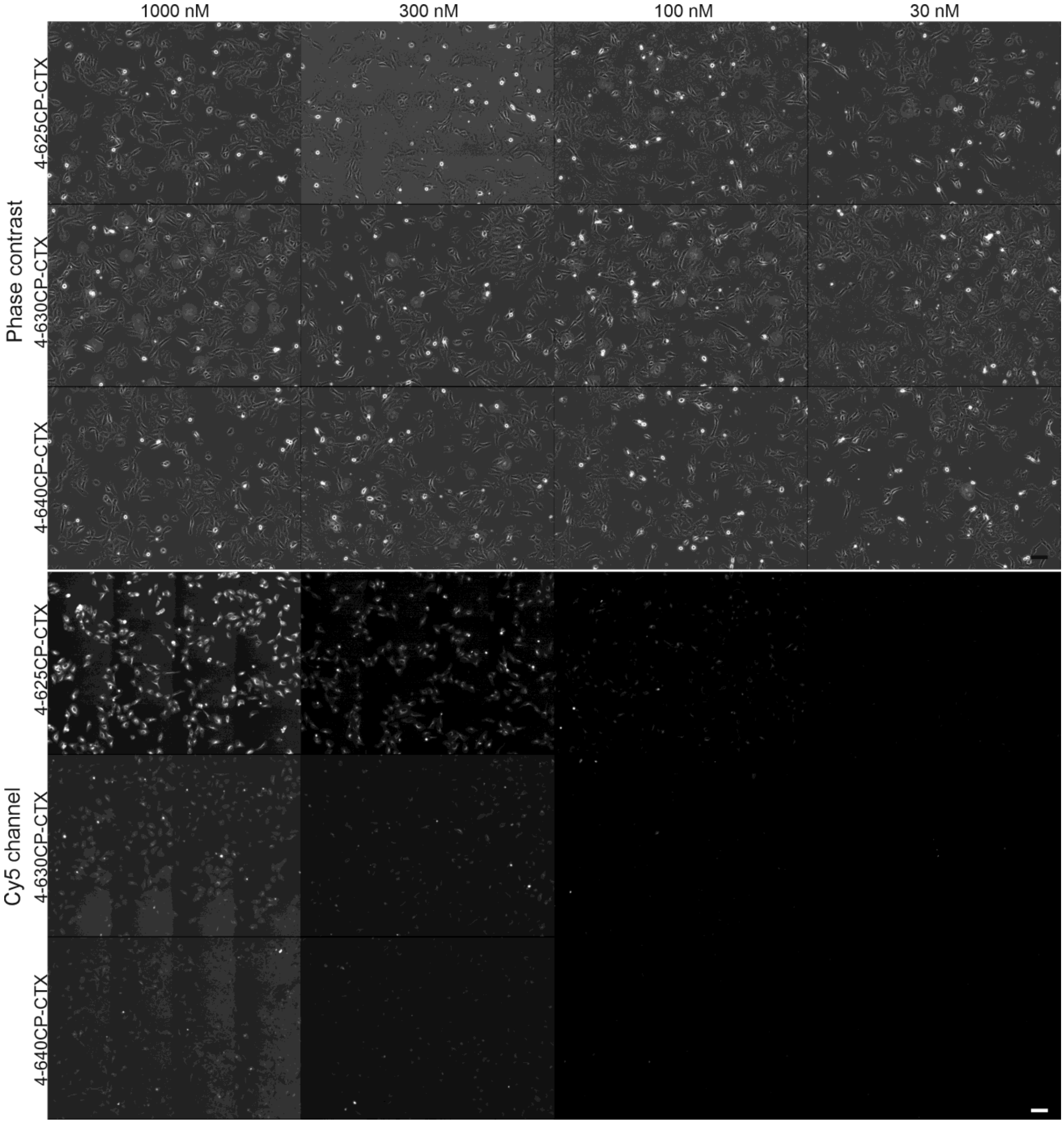

Supplementary Figure 22. Wide field fluorescence microscopy images of Vero cells stained with variable concentrations of **4-625CP-CTX**, **4-630CP-CTX** and **4-640CP-CTX** tubulin probes. Cells were stained in DMEM growth medium containing 10% FBS at 37 °C for 1 h, washed once with HBSS and imaged in DMEM growth medium containing 10% FBS on Biotek Lionheart FX automated microscope. Scale bars: 100 µm. See supplementary materials and methods section for further details on imaging settings.

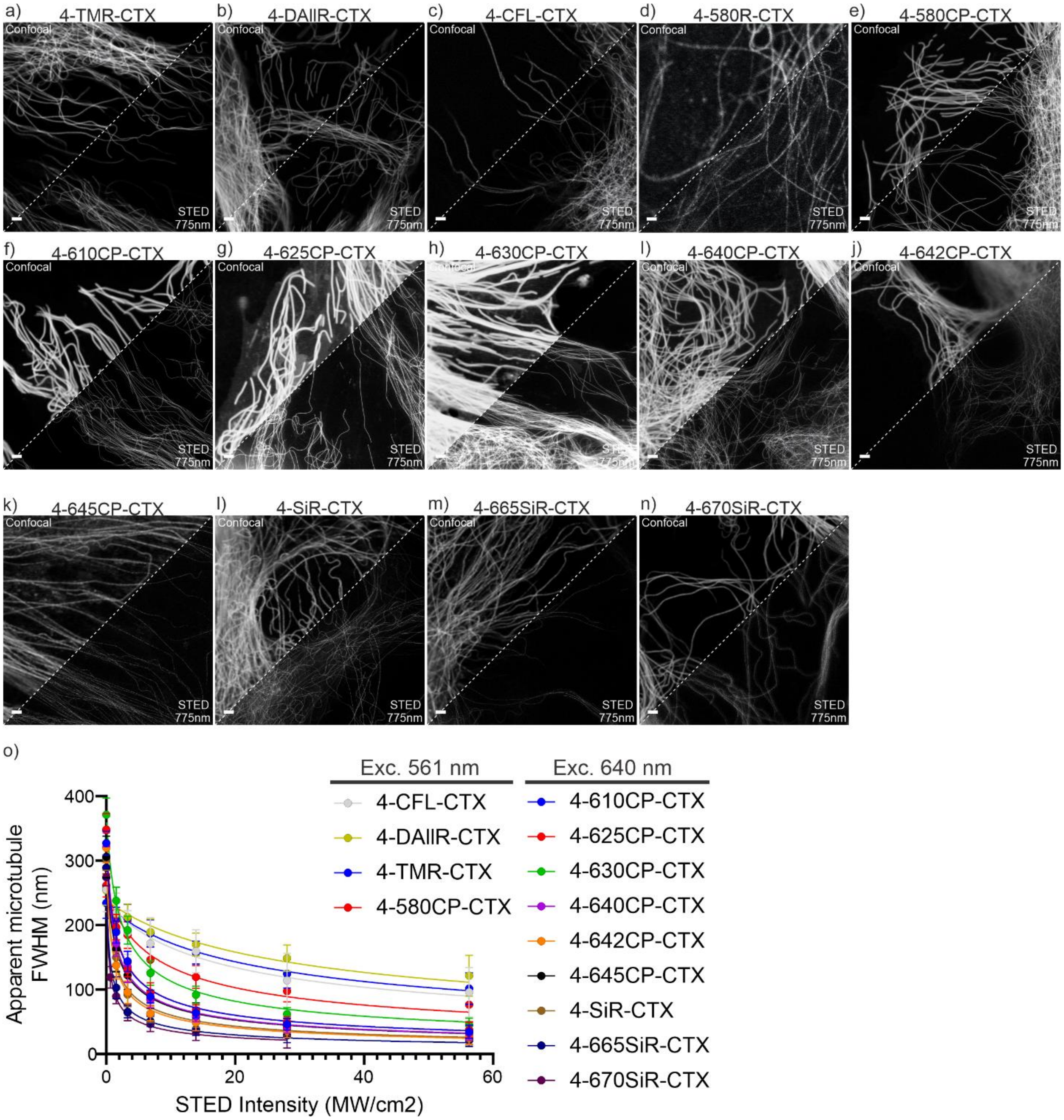

Supplementary Figure 23. Confocal and STED images of microtubules in living human fibroblasts under no-wash conditions. Dyes were excited with: a) - e) 3 mW 561 nm laser; f) - n) 0.3 mW 640 nm laser. STED images were acquired with 775 nm depletion laser using 28 MW/cm^2 intensity. o) The apparent microtubule FWHM as a function of the 775 nm STED laser intensity used for imaging of living human fibroblasts stained with the indicated tubulin probes. Data presented as mean with SD, N = 3 independent experiments, n ≥ 20 microtubules measured in total. Living human fibroblasts were incubated with 100nM of the indicated tubulin probe in growth medium containing 10% FBS at 37 °C for 1 h and imaged subsequently. Images were acquired on Abberior Expert line scanning microscope equipped with 775 nm STED laser. Pixel size: 10 x 10 nm. Scale bars: 2 µm. See supplementary materials and methods section for further details on imaging settings.

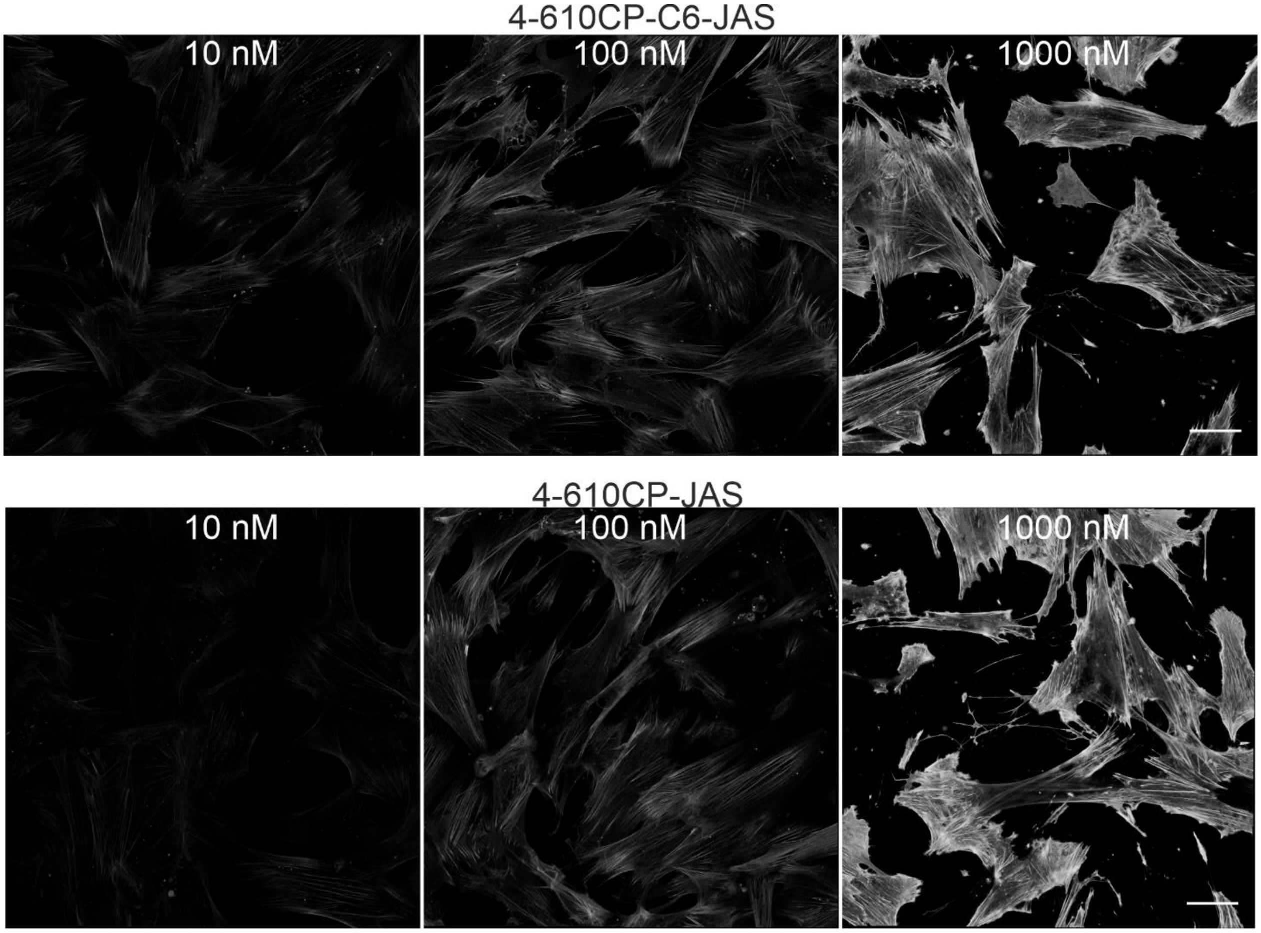

Supplementary Figure 24. Confocal microscopy images of actin in human fibroblasts stained with jasplakinolide - based probes containing different linker length probes– **4-610CP-C6-JAS** and **4-610CP-JAS**. Cells were incubated with probes at indicated concentration in DMEM growth medium containing 10% FBS at 37 °C for 1 h and subsequently imaged on Abberior Facility line without removal of the probes. Multiple fields of view were stitched with SVI Huygens software. Scale bars: 50 µm. Note, the performance of both probes is identical. See supplementary materials and methods section for further details on imaging settings.

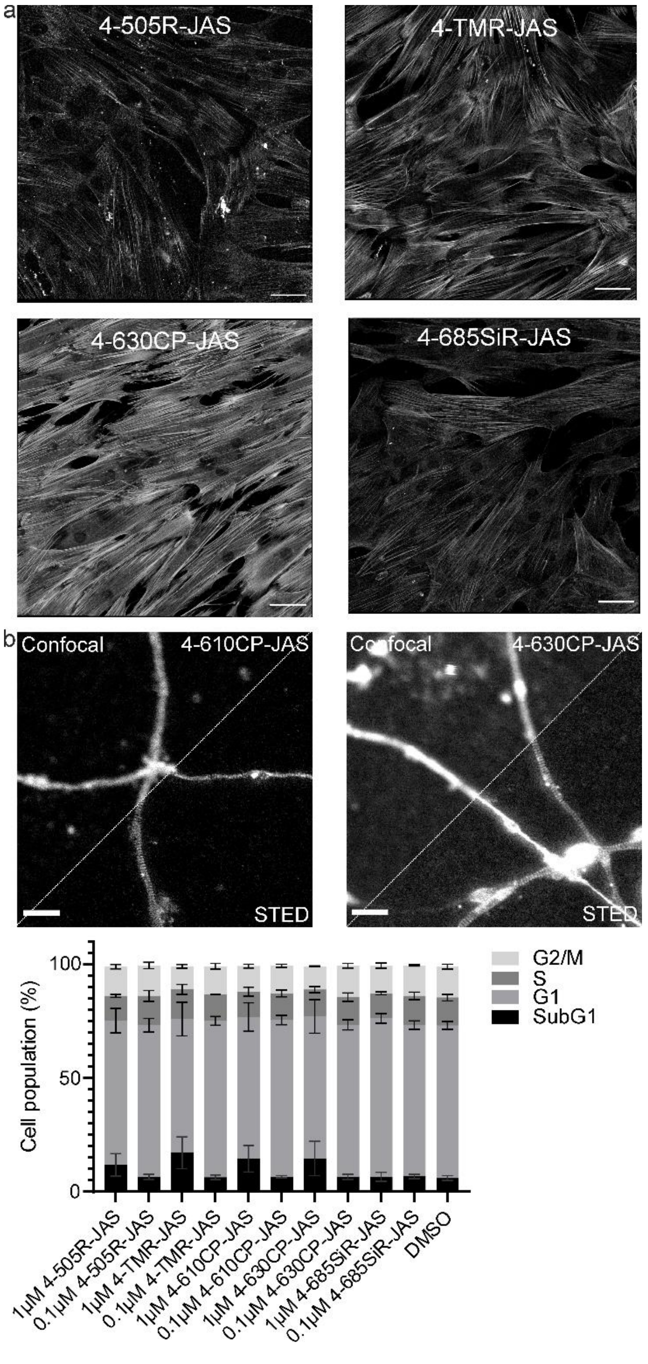

Supplementary Figure 25. Properties of the actin probes. a. Confocal microscopy images of live fibroblasts stained with 100 nM actin probes for 1h at 37°C. Cells were imaged in growth DMEM media without probe removal using Abberior Facility line microscope. Multiple fields of view were stitched with SVI Huygens software. Scale bars: 50 µm. b. Confocal and STED images of actin cytoskeleton in neurons stained with 100 nM **4-610CP-JAS** or **4-630CP-JAS** probes in growth media without probe removal. Images acquired on Abberior Expert line microscope. See methods section for further details on imaging settings. c. Cytotoxicity measurements of the actin probes. HeLa cells were incubated with the indicated concentrations of the probes at 37 °C for 24 h in a humidified 5% CO_2 incubator. Experimental data are averages of three independent experiments (N = 3, n ≥ 9000 cells) and presented as means with standard deviations.

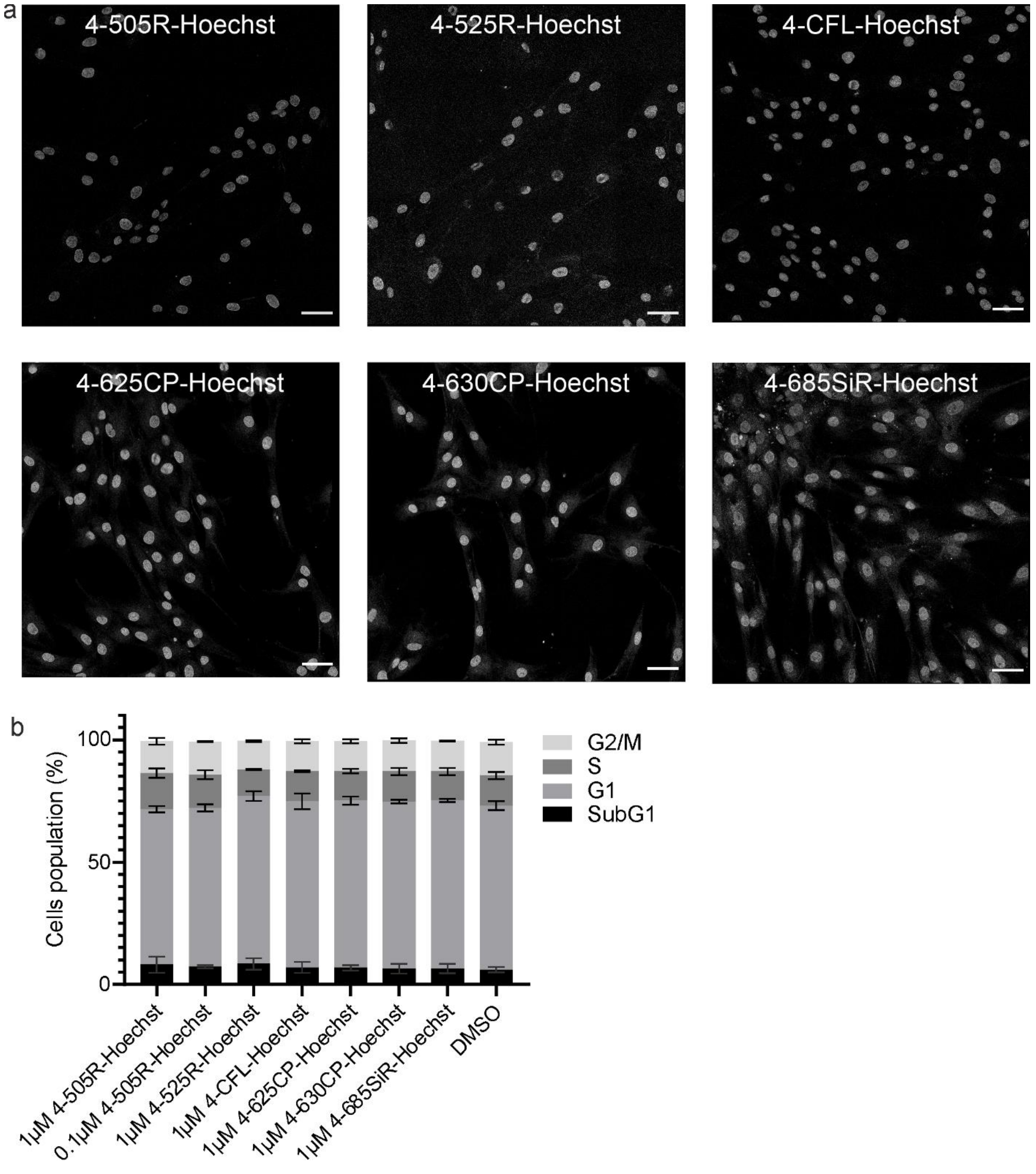

Supplementary Figure 26. Properties of DNA probes. a. Confocal microscopy images of live fibroblasts stained with 100 nM DNA probes for 1h at 37°C. Cells were washed once with HBSS and imaged in growth DMEM media on Abberior Facility line microscope. Multiple fields of view were stitched with Huygens software. Scale bars: 50 µm. See supplementary materials and methods section for further details on imaging settings. b. Cytotoxicity measurements of DNA probes. HeLa cells were incubated with the indicated concentrations of Hoechst derivatives at 37 °C for 24 h in a humidified 5% CO_2 incubator. Experimental data are averages of three independent experiments (N = 3, 9000 ≤ n ≤ 10000 cells) and presented as means with standard deviations.

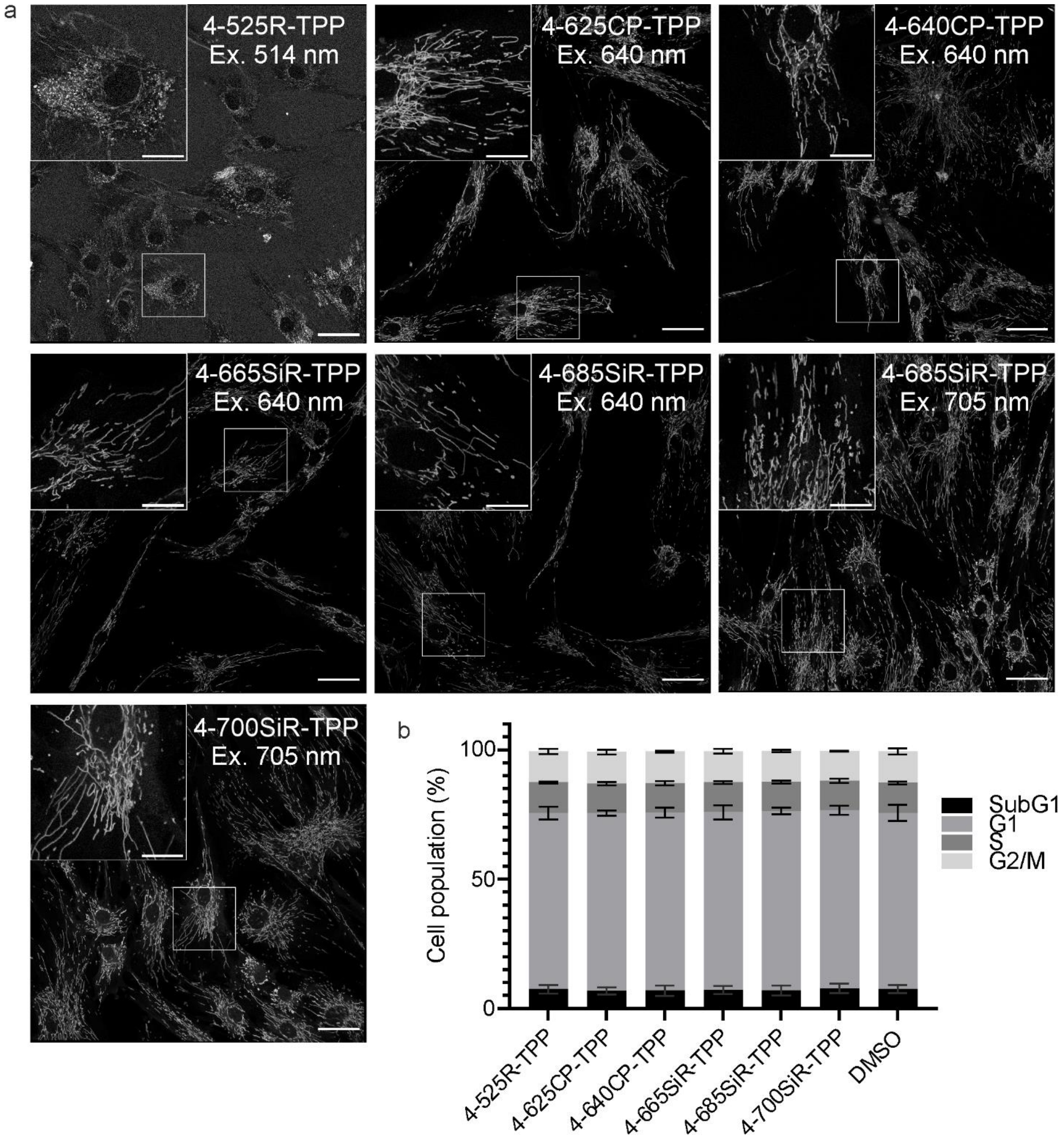

Supplementary Figure 27. Properties of probes targeting mitochondria. a. Confocal fluorescence microscopy images of human fibroblasts stained with 100nM triphenylphosphonium (TPP) mitochondrial probes. Cells were stained in DMEM growth medium containing 10% FBS at 37 °C for 1 h and imaged on Abberior Facility line without removal of the probes. Multiple fields of view were stitched with SVI Huygens software. Note, excitation laser wavelength is indicated. Scale bars 50 µm, insets – 20 µm. b. Cytotoxicity measurements of mitochondrial probes. HeLa cells were incubated with 100 nM probes at 37 °C for 24 h in a humidified 5% CO_2 incubator. Experimental data are averages of three independent experiments (N = 3, 9000 ≤ n ≤ 10000 cells) and presented as means with standard deviations. See supplementary materials and methods section for further details on imaging settings.

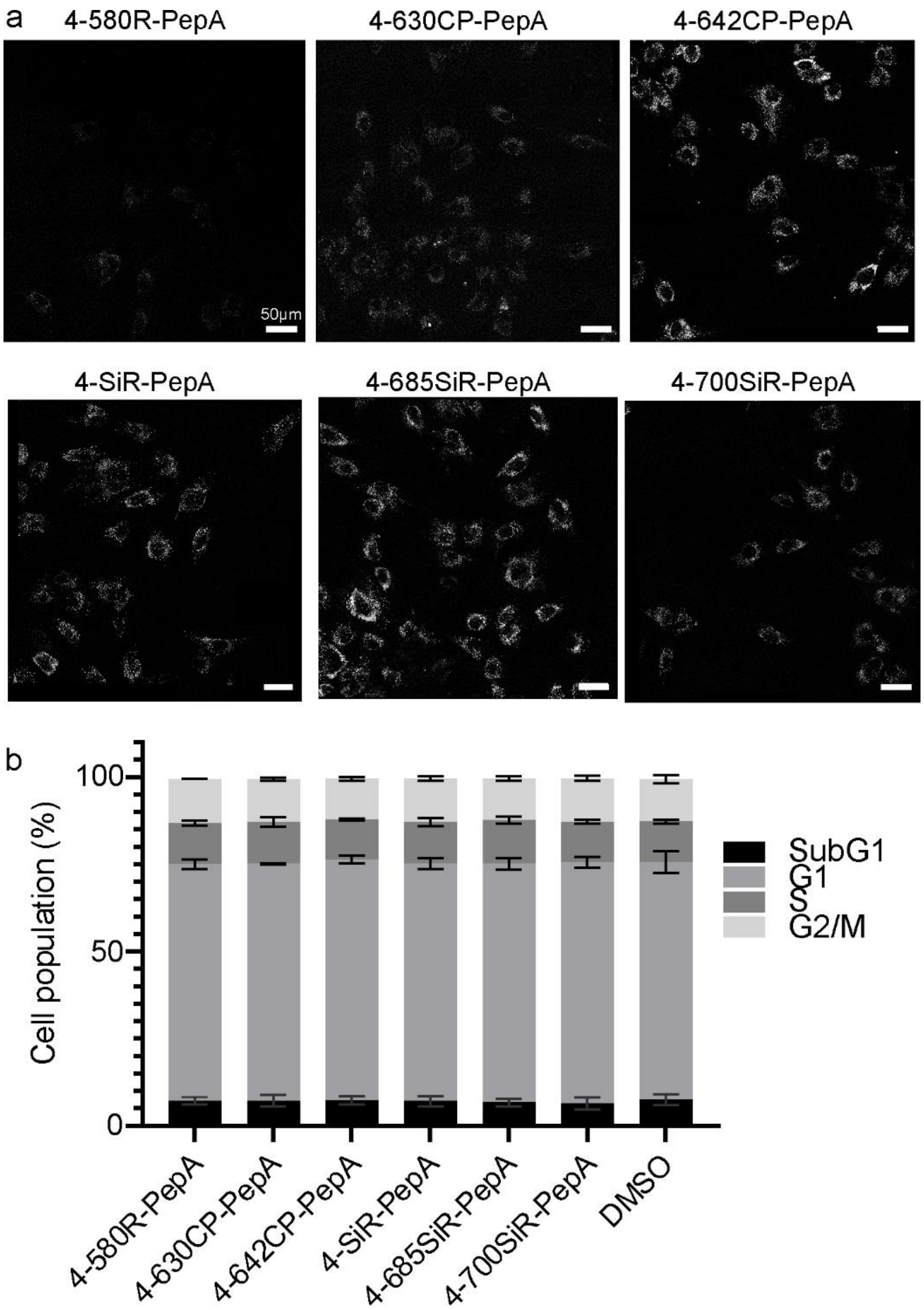

Supplementary Figure 28. Properties of lysosomal probes. a. Staining of human fibroblasts with new dye-pepstatin A conjugates. The cells were incubated with 100 nM probe in DMEM medium with FBS for 1h at 37°C, washed once with medium and once with HBSS and imaged in the same medium on Abberior facility line microscope in a confocal imaging mode. 25 fields of view were acquired and were stitched with SVI Huygens software. Note, that due to the different spectra, staining intensities cannot be compared. b. Cytotoxicity measurements of lysosomal probes. HeLa cells were incubated with 100 nM probes at 37 °C for 24 h in a humidified 5% CO_2 incubator. Experimental data are averages of three independent experiments (N = 3, 9000 ≤ n ≤ 10000 cells) and presented as means with standard deviations. See supplementary materials and methods section for further details on imaging settings.

Supplementary Tables

Supplementary Table 1. Photophysical properties of the synthesized 4-carboxyrhodamines in PBS (pH =7.4) +0.1%SDS buffer. Data represent mean ±SD (N =3).

Dye (-COOH)	λ_{max}^{abs} (nm)	λ_{max}^{em} (nm)	ε x 10^2 (m^{-1} cm^{-1})	QY (%)	τ (ns)
**4-505R (22)**	504	524	672 ± 16	98 ± 2	4.16 ± 0.02
**4-525R (23)**	526	547	821 ± 26	98 ± 3	4.39 ± 0.06
**4-DMRh (25)**	521	547	683 ± 44	20 ± 1	–
**4-TAllR (17)**	551	573	991 ± 104	90 ± 6	4.12 ± 0.04
**4-DAllR (18)**	554	574	816 ± 31	75 ± 2	3.86 ± 0.01
**4-CFL (26)**	549	574	132 ± 2.3	64 ± 4	3.59 ± 0.01
**4-TMR (3)**	554	575	778 ± 45	59 ± 1	2.99 ±0.01
**4-580R (5)**	580	598	896 ± 43	86 ± 1	4.82 ± 0.02
**4-580CP (24)**	591	613	1166 ± 55	66 ± 1	4.20 ± 0.01
**4-610CP (4)**	616	640	1055 ± 94	67 ± 1	4.23 ± 0.01
**4-DAllCP (19)**	617	640	1035 ± 185	75 ± 2	4.70 ± 0.04
**4-625CP (10)**	627	653	1032 ± 10	60 ± 1	4.19 ± 0.02
**4-630CP (11)**	629	647	1100 ± 74	77± 4	4.53 ± 0.04
**4-640CP (7)**	641	666	1092 ± 83	46 ± 1	3.79 ± 0.01
**4-642CP (12)**	648	680	1157 ± 31	40± 2	2.76 ± 0.01
**4-645CP (6)**	645	661	1086 ± 78	80 ± 3	4.61 ± 0.05
**4-SiR (2)**	655	674	733 ± 51	62 ± 1	3.95 ± 0.03
**4-665SiR (13)**	669	687	1004 ± 37	55 ± 2	3.84 ± 0.02
**4-670SiR (14)**	675	699	764 ± 78	41 ± 1	3.23 ± 0.01
**4-685SiR (15)**	693	712	1076 ± 100	44 ± 2	3.46 ± 0.04
**4-690SiR (16)**	696	724	730 ± 39	20 ± 1	1.96 ± 0.01
**4-700SiR (8)**	700	726	920 ± 26	24.6 ± 0.3	2.62 ± 0.01
**4-720SiR (9)**	732	764	636 ± 43	15 ± 1	1.74 ± 0.02

Supplementary Table 2. Measured D_{50} and K_{L-Z} values of the free dyes and respective 4-(benzyloxy)-2-chloropyrimidine (CP) probes. Data represent mean ±SD (N =3).

Dye	-COOH	-CONH-	-COOH	-CONH-
	$^{Dye}D_{50}$	$^{Probe}D_{50}$	$^{Dye}K_{L-Z}$	$^{Probe}K_{L-Z}$
**4-505R (22)**	13 ± 1	30 ± 1	4.1 ± 1.1	2.0 ± 0.3
**4-525R (23)**	8.9 ± 0.3	18.1 ± 0.3	9.4 ± 1.3	7.2 ± 0.6
**4-DMRh (25)**	38 ± 3	20.4 ± 0.5	1.0 ± 0.1	0.17 ± 0.01
**4-TAllR (17)**	20.4 ± 0.4	–[a]	3.5 ± 1.4	–[a]
**4-DAllR (18)**	15.4 ± 0.4	–[a]	9.8 ± 0.2	–[a]
**4-CFL (26)**	–[b]	–[b]	–[b]	–[b]
**4-TMR (3)**	14 ± 1	23.4 ± 0.3	8.5 ± 0.8	7.9 ± 0.5
**4-580R (5)**	3.8 ± 0.4	6.5 ± 0.2	10.22 ± 0.02	9.3 ± 2.8
**4-580CP (24)**	30.8 ± 0.7	59 ± 2	1.0 ± 0.1	0.23 ± 0.01
**4-610CP (4)**	32.1 ± 0.7	65 ± 2	0.99 ± 0.04	0.19 ± 0.08
**4-DAllCP (19)**	40.0 ± 0.4	–[a]	0.4 ± 0.1	–[a]
**4-625CP (10)**	25 ± 1	44.0 ± 0.7	5.7± 1.3	0.5 ± 0.1
**4-630CP (11)**	17.6 ± 0.6	35.0 ± 0.3	7.9 ± 2.2	1.75 ± 0.04
**4-640CP (7)**	19.1 ± 0.4	37.5 ± 0.4	6.5 ± 2.8	1.5 ± 0.2
**4-642CP (12)**	32.0 ± 0.6	65 ± 4	1.4 ± 0.4	0.5 ± 0.2
**4-645CP (6)**	8.1 ± 0.3	19.1 ±0.4	13.5 ± 2.8	12.0 ± 4.1
**4-SiR (2)**	>80	>80	0.04 ± 0.01	0.001
**4-665SiR (13)**	37.2 ± 0.6	68 ± 4	1.5 ± 0.8	0.13 ± 0.07
**4-670SiR (14)**	>80	>80	0.2 ± 0.1	0.001
**4-685SiR (15)**	29.6 ± 0.5	65 ± 3	1.6 ± 0.3	0.4 ± 0.2
**4-690SiR (16)**	>80	>80	0.06 ± 0.04	0.001
**4-700SiR (8)**	>80	>80	0.23 ± 0.04	0.001
**4-720SiR (9)**	>80	>80	0.4 ± 0.4	0.0001

[a] The corresponding 4-(benzyloxy)-2-chloropyrimidine (CP) derivatives were not synthesized. [b] The 4-CFL showed no change along increasing amount of water in 1,4-dioxane mixture and neither D_{50} nor K_{L-Z} values were obtained.

Supplementary Table 3. TDDFT 6-311++G (d,p)/IEFPCM (water) calculated vertical excitation energy and wavelength values before and after empirical correction of 0.4 eV and experimentally measured absorption maxima values of the studied dyes.

	Calculated by TDDFT		Empirically corrected			Experimentally measured	
Dye (-COOH)	$\Delta E_{H\text{-}L}$ (eV)	λ_{abs} (nm)[a]	$\Delta E_{H\text{-}L}$ (-0.4 eV)	λ_{abs} (nm)		λ_{abs} (nm)	$\Delta E_{H\text{-}L}$ (ev)[b]
**4-505R (22)**	2.888	429	2.489	498		500	2.480
**4-525R (23)**	2.783	445	2.383	520		522	2.375
**4-TMR (3)**	2.635	470	2.236	555		551	2.250
**4-580R (5)**	2.536	489	2.137	580		579	2.141
**4-580CP (24)**	2.564	483	2.164	573		585	2.119
**4-610CP (4)**	2.460	503	2.061	602		611	2.029
**4-625CP (10)**	2.416	513	2.016	615		621	1.996
**4-630CP (11)**	2.416	513	2.017	615		624	1.987
**4-640CP (7)**	2.363	525	1.964	631		636	1.949
**4-642CP (12)**	2.305	538	1.906	651		641	1.934
**4-645CP (6)**	2.347	528	1.947	637		642	1.931
**4-SiR (2)**	2.353	527	1.954	635		649	1.910
**4-665SiR (13)**	2.295	540	1.895	654		663	1.870
**4-670SiR (14)**	2.280	544	1.880	659		668	1.856
**4-685SiR (15)**	2.219	559	1.820	681		687	1.804
**4-690SiR (16)**	2.165	573	1.770	702		687	1.805
**4-700SiR (8)**	2.200	564	1.800	689		694	1.786
**4-720SiR (9)**	2.039	608	1.639	756		721	1.719

Supplementary Table 4. Properties of tubulin probes

Probe	EC_{50} (nM)	Isat (MW • cm^2)	App. $FWHM_{min}$ (nm)	Microtubule to cytosol signal ratio[b]
**4-505R-CTX (48)**	28 ± 4	n.a.	n.a.	n.a.
**4-525R-CTX (49)**	34 ± 4	n.a.	n.a.	n.a.
**4-DMRh-CTX (50)**	48 ± 3	n.a.	n.a.	n.a.
**4-DAllR-CTX (52)**	21 ± 3	16 ± 1	121 ± 32[a]	17 ± 5
**4-CFL-CTX (47)**	91 ± 7	9 ± 1	96 ± 38 [a]	27 ± 12
**4-TMR-CTX (51)**	6 ± 2	12 ± 1	102 ± 24 [a]	50 ± 25
**4-580R-CTX (53)**	239 ± 30	n.a.	n.a.	n.a.
**4-580CP-CTX (54)**	12 ± 1	3.9 ± 0.2	77 ± 26 [a]	16 ± 5
**4-610CP-CTX (55)**	15 ± 2	0.69 ± 0.02	34 ± 10 [a]	35 ± 14
**4-625CP-CTX (56)**	3.1 ± 0.2	0.61 ± 0.02	37 ± 12 [a]	16 ± 3
**4-630CP-CTX (57)**	7.0 ± 0.4	1.01 ± 0.03	45 ± 11 [a]	9 ± 2
**4-640CP-CTX (58)**	19 ± 2	0.49 ± 0.02	32 ± 11 [a]	7 ± 2
**4-642CP-CTX (59)**	61 ± 58	0.33 ± 0.01	27 ± 12 [a]	15 ± 5
**4-645CP-CTX (60)**	46 ± 5	0.61 ± 0.02	34 ± 11 [a]	17 ± 5
**4-SiR-CTX (61)**	248 ± 17	0.41 ± 0.01	24 ± 10 [a]	79 ± 29
**4-665SiR-CTX (62)**	98 ± 11	0.21 ± 0.01	21 ± 9 [a]	29 ± 10
**4-670SiR-CTX (63)**	93 ± 5	0.18 ± 0.01	29 ± 20[b]	25 ± 10
**4-685SiR-CTX (64)**	114 ± 8	n.a.	n.a.	n.a.
**4-690SiR-CTX (65)**	n.a.	n.a.	n.a.	n.a.
**4-700SiR-CTX (66)**	96 ± 33	n.a.	n.a.	n.a.
**4-720SiR-CTX (67)**	n.a.	n.a.	n.a.	n.a.

a - values calculated from STED images (775 nm laser, 100% power).

b - measured at 50% STED laser power.

All data presented as mean fitted value with SEM, except microtubule to cytosol signal ratio which is represented as mean value with SD.

Supplementary Table 5. Comparison of microtubule diameter measured in cells stained with **4-625CP-CTX** and **4-630CP-CTX** in 3D confocal and 3D STED mode. Data represents mean ± SD (N=30)

	**3D Confocal**				**3D STED**				
**Dye-CTX**	xz plane, nm		zy plane, nm		xz plane, nm		zy plane, nm		Microtubule to cytosol signal ratio
	**$FWHM_x$**	**$FWHM_z$**	**$FWHM_y$**	**$FWHM_z$**	**$FWHM_x$**	**$FWHM_z$**	**$FWHM_y$**	**$FWHM_z$**	
**4-625CP** (10 nM)	396 ± 87	700 ± 122	349 ± 78	669 ± 92	112 ± 16	89 ± 16	111 ± 20	95 ± 14	20 ± 12
**4-630CP** (10 nM)	317 ± 88	607 ± 124	299 ± 86	554 ± 79	158 ± 32	108 ± 21	150 ± 34	126 ± 22	12 ± 7

Supplementary Table 6. List of Videos and imaging parameters.

			excitation / emission				
			488 nm / 500-550 nm	561 nm / 580-620 nm	640 nm / 660-680 nm	700 nm / 720-770 nm	line
**Video 1**	100 nM 4-700SiR-TPP	human fibroblasts	10%	15%	20%	10%	2
	30 nM 4-642CP-PepA						
	30 nM 4-DAllR-CTX						
	1000 nM 4-505R-Hoe						
**Video 2**	100 nM 4-700SiR-TPP	HUVEC	10%	20%	20%	5%	1
	10 nM 4-642CP-PepA						
	10 nM 4-DAllR-CTX						
	100 nM 4-505R-Hoe						
**Video 3**	100 nM 4-700SiR-TPP	human fibroblasts	15%	50%	20%	5%	2
	30 nM 4-642CP-PepA						
	1000 nM 4-CFL-Hoe						
	1000 nM 4-505R-CTX						
**Video 4**	100 nM 4-700SiR-TPP	U-2 OS vimentin-HaloTag	25%	25%	30%	5%	2
	100 nM 4-642CP-Halo						
	30 nM 4-DAllR-CTX						
	1000 nM 4-505R-Hoe						
		line step #1		✓		✓	
		line step #2	✓		✓		

Supplementary Methods

Molecular biology and biochemical methods

Determination of absolute Quantum yields

All reported absolute fluorescence quantum yield values (Φ) were measured using a Quantaurus-QY spectrometer (model C11374-01, Hamamatsu Photonics). This instrument uses an integrating sphere to determine photons absorbed and emitted by a sample. Measurements were carried out using dilute samples in air saturated solvents at 25°C at concentrations ranging from 10^{-6} to 10^{-7} M (A < 0.1 as indicated in the user's manual) and by using 3 mL quartz cuvettes (Hamamatsu Photonics Art. No. A10095-02) provided by the instrument supplier. The fluorescence quantum yields were measured in PBS buffer (Table 3) and in PBS buffer containing 0.1% SDS (Table S1). Self-absorption corrections, if needed, were performed using the instrument software. Reported values are averages (n = 3) with standard deviation.

Determination of fluorescence lifetimes

The fluorescence decay characteristics of the solution samples in PBS (Table 3) or PBS + 0.1% SDS (Table S1) buffers at concentrations ranging from 10-6 to 10-7 M were recorded using a fluorescence lifetime measurement system (Quantaurus-Tau, Hamamatsu Photonics) in 3 mL high performance quartz glass cuvettes (Hellma Analytics Art. No. 101-10-K-40). The decay profile was registered for 53 - 100 ns interval after excitation and the experiment was continued until 10 000 peak count was reached. The instrument response function was obtained by using diluted LUDOX® TM-50 colloidal silica (Sigma Aldrich). The analysis of the obtained fluorescence decay profile was performed using the instrument software. Reported values are averages (n = 3) with standard deviation.

Reaction yield determination by absorption spectroscopy

After the purification and removal of the solvent, the obtained fluorescent probes were dissolved in a precise (700 µL) volume of the d6-DMSO solvent and were transferred to NMR tube to obtain 1H spectra. Afterwards the contents of the NMR tubes were transferred to an eppendorf and was considered as stock solution. Five 2 µL samples were taken from stock solution and were diluted in five separate eppendorf's with 98 µL of PBS + 0.1% SDS (50-fold dilution), vigorously mixed and aged for 30 min to dissolve aggregates. Then absorption of 2 µL of the diluted samples were measured on nanodrop (Nanodrop 1000, Peqlab) with 1 mm optical path. The measured absorption intensity values at the dyes absorption maxima value were averaged and concentration of the stock solution was determined according to the equation:

$$C=(dilution*A)/(\varepsilon*l) \quad (1)$$

where C –concentration of stock solution; A – sample absorption, ε - extinction coefficient of the dye in PBS containing 0.1% SDS, l – path length

Once the concentration of the stock solution is measured the mass of the obtained fluorescent conjugate could be calculated by following equation:

$$m=C*MW*V \quad (2)$$

where C –concentration of stock solution; MW – molecular weight of the compound; V- volume of stock solution.

Finally, the yield of the synthesis step could be determined by the classical equation:

$$\text{Yield }\% = \frac{\text{m}}{\text{m}_{\text{teor}}} * 100 \quad (3)$$

Where m – obtained mass of the isolated product; m_{teor} – maximal theoretical mass of the product in the reaction.

Plasmid construction

Construction and characterization of pEBTet GW_SNAP plasmid was reported previously [1,2]. This mammalian expression vector contains cytomegalovirus-type 2 tetracycline operator (tetO2)-tetO2 promoter which is “switched on” by addition of doxycycline. Histone H3 coding gene was PCR amplified from pmCherry-H3-23 template (Addgene, #55058) using following primers:

Fw_attB1_H3

GGGGACAAGTTTGTACAAAAAAGCAGGCTTAGCCACCATGGCTCGTACTAAACAGACAGC

Rw_H3_attB2

GGGGACCACTTTGTACAAGAAAGCTGGGTACGCTCTTTCTCCGCGAATGCGGC

Afterwards, purified PCR product was cloned into pEBTet GW_SNAP vector using single tube BP/LR recombination protocol. Final construct sequence has been verified by sequencing.

Maintenance and preparation of cells

Human primary dermal fibroblasts (Lonza, #CC-2511) were cultured in high-glucose DMEM (Thermo Fisher, #31053044) with 10% FBS (Thermo Fisher, #10082147) supplemented with of 1 mM Sodium pyruvate (Sigma, #S8636), 1% GlutaMax (Thermo Fisher, #35050038) and 1% Penicillin-Streptomycin (Sigma, #P0781) in a humidified 5% CO_2 incubator at 37 °C. The cells were split every 3-4 days or at confluence.

HeLa (ATCC, CCL-2) and Vero (Sigma Aldrich, #84113001-DNA-5UG) cells were cultured in high-glucose DMEM (Thermo Fisher, #31966047) with 10% FBS (BioSELL, #S0615) supplemented with 1% Penicillin-Streptomycin (Sigma, #P0781) in a humidified 5% CO_2 incubator at 37 °C. The cells were split every 3-4 days or at confluence.

U-2 OS cells (ATCC, HTB-96) and U-2 OS Vimentin-Halo cell line (in-house produced and described in ref [3]) were cultured in McCoys 5A medium (Thermo Fisher, #16600082) with 10% FBS (BioSELL, #S0615) supplemented with 1 mM Sodium pyruvate (Sigma, #S8636) and 1% of Penicillin-Streptomycin (Sigma #P0781) in a humidified 5% CO_2 incubator at 37 °C. U-2 OS CRISPR-NUP96-SNAP clone no.33 (CLS Cell Lines Service GmbH, # 300444, described in ref [4]) cells were cultivated under the same conditions, except growth medium was supplied with MEM Non-Essential Amino Acids Solution (Thermo Fisher #11140050). The cells were split every 3-4 days or at confluence.

Human umbilical vein endothelial cells (HUVEC) (Lonza, #CC-2519) cells were cultured in EMB medium (Lonza #CC-3129) and LVES (Thermo Fisher #C3008MP) supplemented with 1% of Penicillin-Streptomycin (Sigma #P0781) in a humidified 5% CO_2 incubator at 37 °C. Cells were split every 3-4 days or at confluence.

Cells were seeded in glass bottom 12-well (MatTek, #P12G-1.5-14-F) or 24-well (MatTek, #P24G-1.5-13-F) plates for wide-field imaging experiments on Biotek Lionheart FX automated microscope. Confocal and STED microscopy experiments were performed using µ-Slide 8 Well Glass Bottom dishes (Ibidi, #80827).

Inducible U-2 OS cell line expressing SNAP-tagged histone H3 was generated by transfecting cells with pEBTet H3-SNAP expression vector at ~70% confluence using Lipofectamine 2000 (Thermo Fisher, Cat. No. 11668027) following manufacturer’s recommendations. Transfected cells were cultivated in selection media composed of DMEM (Thermo Fisher, Cat. No. 31053-028) containing 10% FBS (Thermo Fisher, Cat. No. 10082139) and 1 µg/ml puromycin (Sigma, Cat. No. P9620) for approximately two weeks. Thereafter, selected cells were frozen in 10% DMSO and stored at -80°C. Expression of transgene was induced using 0.1 µg/ml doxycycline (Sigma, Cat. no. D9891) for 24 - 48 h before imaging experiment.

Preparation and staining of mouse living primary cells and tissues

Primary neuronal isolation and staining

The protocol for hippocampal neurons isolation has been described previously[5]. Neurons were collected from 8 CD1 mice (Mus musculus) equally represented both sexes which were kept at 20.8°C ambient average temperature and humidity 50.5%. Briefly, hippocampi were dissected from CD1 mice of mixed gender at postnatal day P0-P1, treated with trypsin (0,25%; Gibco, cat. 15090046) for 25 minutes at 37ºC

and subsequently manually dissociated. Cells were plated on coverslips pre-coated with poly-L-ornithine (100 μg/ml; Sigma-Aldrich, cat. P3655) and laminin (1 μg/ml; BD Bioscience, cat. 354232) and cultivated in Neurobasal medium (Gibco, cat. 21103049) supplemented with B27 serum-free supplement (2%; Gibco, cat. 17504044), GlutaMax (1x; Gibco, cat. 35050061) and Penicillin-Streptomycin (100 units/ml and 100 μg/ml respectively; Gibco, cat. 15140122).
Living neurons were incubated in culture medium containing the indicated concentration of probe at 37ºC for 30 min and imaged without washing at room temperature thereafter.

Tissue isolation and staining

Four 23-24 weeks old CD-1 mice of both genders, kept at 20.1°C ambient average temperature and 56.9% humidity, were sedated with 3% isoflurane in a sealed container and quickly euthanized by cervical dislocation. The skull and the abdomen were opened, the organs were transferred to ice cold EGTA solution[6] and sliced manually using a scalpel blade. Slices were incubated in EGTA solution containing the indicated concentration of probe on ice for 30min and imaged without washing at room temperature thereafter.

Cell cycle analysis by imaging flow cytometry and EC_{50} determination

The probes dissolved in DMSO (typically 500 - 2000× stock) were added into the media of cultured HeLa cells and incubated for 24 h at 37°C in humidified incubator with 5% CO_2. We used 6-well plates containing ~250,000 cells per well for this experiment. Afterwards, cells were processed according to the NucleoCounter® NC-3000™ two-step cell cycle analysis protocol for cells attached to T-flasks, cell culture plates or micro-carriers. We used NC-Slide A2™ slides (Chemometec, Cat. No. 942-0001) loaded with ~30 µL of the cell suspensions into a chamber of the slide. According to manufacturer's recommendations ~10,000 cells in total were measured. The collected data were analyzed with ChemoMetec NucleoView NC-3000 software, version 2.1.25.8. The experiments were repeated three times on different days and the results are presented as means with standard deviations. The EC_{50} values were determined by plotting percentage of subG1 phase cells and fitting the data in GraphPad Prism 8.4.0 to the following function:

$$Y = Y_{min} + (Y_{max} - Y_{min}) / \left(1 + \left(\frac{EC_{50}}{X}\right)^{Hill}\right) \quad (4)$$

where X – probe concentration, Y_{min} – % subG1 in sample with DMSO (no probe added), Y_{max} – the highest reachable % subG1, *Hill* - Hill coefficient determining the steepness of a dose-response curve, EC_{50} - the concentration of probe that provoking halfway of subG1 cells in a population between the baseline (Y_{min}) and maximum response (Y_{max}).

Wide field microscope and imaging parameters

For wide-field fluorescence microscopy images were acquired using Biotek Lionheart FX Automated Microscope equipped with Olympus dry 20× NA 0.45 objective and incubator set to +37°C. During acquisition cells were kept under atmosphere containing 5% of CO_2. The image acquisition parameters are listed below:

Supplementary Table 7. Parameters of image acquisition on a wide-field Lionheart FX Automated Microscope

Channel	LED	Filter cube	LED intensity (A.U.)	Integration time, ms	Camera gain
**Hoechst**	365 nm	DAPI 377/447	5	10	30
**GFP channel**	465 nm	GFP 469/525	7	75	30
**RFP channel**	523 nm	RFP	9	50	30
**TxR channel**	590 nm	Texas red	9	50	30
**Cy5 channel**	623 nm	Cy5	7	50	30

Spinning disk confocal microscope and imaging parameters

Spining disk confocal microscopy imaging was performed on an inverted microscope (Nikon Ti2) equipped with a 640 nm laser (Toptica, 150 mW) used for excitation and the oil-immersion objective Plan Apo Lambda 60x Oil NA 1.4 (MRD01605, Nikon). Emission light filtered using 665nm long pass filter. Images registered using back illuminated sCMOS camera Teledyne Photometrics Prime BSI with pixel size 6.5 x 6.5 µm corresponding to image pixel size 111 x 111 nm. All probes were used at 100 nM concentration. The image acquisition parameters are listed below:

Supplementary Table 8. Image acquisition parameters on a spining disk confocal microscope.

Probe	Laser power	Exposure (ms)
**4-625CP-(O4)Halo**	30%	100
**4-630CP-(O4)Halo**	30%	100
**4-640CP-(O4)Halo**	30%	100
**4-642CP-(O4)Halo**	30%	100
**4-SiR-(O4)Halo**	30%	300
**4-665SiR-(O4)Halo**	30%	300
**4-670SiR-(O4)Halo**	30%	300
**4-685SiR-(O4)Halo**	30%	300
**4-690SiR-(O4)Halo**	100%	300
**4-700SiR-(O4)Halo**	100%	300
**4-720SiR-(O4)Halo**	100%	300

Confocal/STED microscopes and imaging parameters

Confocal and STED images were acquired using Abberior STED Expert Line, Abberior STED Facility Line scanning (Abberior Instruments GmbH) or TCS SP8 (Leica) microscopes. STED images were acquired using Abberior STED Expert Line or Abberior STED Falicilty Line scanning (Abberior Instruments GmbH) microscopes.

TCS SP8 confocal microscope equipped with 405, 458, 476, 488, 496, 514, 561 and 633 nm excitation lasers as well as HC PL APO CS2 63x/1.40 Oil objective (Leica) was used in described study. Microscope has three Hybrid and two PMT detectors which can be tuned to any detection window in the range 400 – 800 nm. Voxel size was 0.08µm x 0.08µm x 0.2µm (in xyz respectively) and pinhole size was set to 1.0 AU. Laser powers were optimized for each sample.

Abberior STED Expert Line equipped with 561 nm and 640 nm 40 MHz pulsed excitation lasers, a pulsed 775 nm 40 MHz 3W STED laser, and an UPlanSApo 100x/1.40 Oil objective. The following detection windows were used: for the TMR/580CP channel 615 / 20 nm, and for the 610CP/SiR channel 685 / 70 nm. Pixel size was 10 – 30 nm in the xy plane was used for 2D STED images. Laser powers were optimized for each sample. 3D STED images were acquired using pinhole set to 0.8 AU, voxel size set to 40 x 40 x 40 nm, 3D STED doughnut set to 95%, with single line accumulation and xzy scanning mode.

Abberior STED Facility Line equipped with 488, 515, 561, 640 and 700 nm 40 MHz pulsed excitation lasers, a pulsed 775 nm 40 MHz 3W STED laser, and an UPlanSApo 60x/1.42 Oil objective. Microscope has two APD and two MATRIX detectors which can be tuned to any detection window in the range 400 – 800 nm. Pixel size was 30 nm in the xy plane was used for 2D STED images and 80 nm in the xy plane for large field of view images. Laser powers were optimized for each sample.

Estimation of the STED effect was performed on Abberior STED Expert line microscope by varying the STED laser power from 0 to 100% while measuring cells stained with tubulin probes. Obtained data were fitted using GraphPad Prism 8.4.0 to the following equation:

$$Y = \frac{d_{conf}}{\sqrt{1+I/I_{sat}}} \quad (5),$$

where d_{conf} – confocal resolution, I – STED laser intensity power, I_{sat} – saturating STED laser intensity power.

Supplementary Table 9. Settings used for confocal and STED imaging

Figure	Probe	Concentration	Microscope	Excitation (nm)	Pixel dwell time (µs)	Objective	Pixel size (nm)	Emission (nm)
Figure 5a and S23	**4-505R-CTX**	100 nM	Abberior facility line	488	2	60x NA 1.42 oil	10	505 – 608
	**4-DMRh-CTX**			518	2			535 – 638
	**4-525R-CTX**			518	1			528 - 638

	**4-TMR-CTX**<br>**4-DAllR-CTX**<br>**4-CFL-CTX**<br>**4-580R-CTX**<br>**4-580CP-CTX**<br>**4-610CP-CTX**<br>**4-625CP-CTX**<br>**4-630CP-CTX**<br>**4-640CP-CTX**<br>**4-642CP-CTX**<br>**4-645CP-CTX**<br>**4-SiR-CTX**<br>**4-665SiR-CTX**<br>**4-670SiR-CTX**	100 nM	Abberior expert line	561<br>561<br>561<br>561<br>561<br>640<br>640<br>640<br>640<br>640<br>640<br>640<br>640<br>640	1	100x NA 1.4 oil	10	580 – 630<br>580 – 630<br>580 – 630<br>580 – 630<br>580 – 630<br>650 – 720<br>650 – 720<br>650 – 720<br>650 – 720<br>650 – 720<br>650 – 720<br>650 – 720<br>650 – 720<br>650 – 720
	**4-685SiR-CTX**<br>**4-700SiR-CTX**<br>**4-720SiR-CTX**	100 nM	Abberior facility line	705<br>705<br>705	2	60x NA 1.42 oil	10<br>10<br>50	725 – 780<br>725 – 780<br>725 – 780
Figure 5c	**4-625CP-CTX**	10 nM	Abberior expert line	640	3	100x NA 1.4 oil	20	650 - 720
Figure 6a	**4-580CP-CTX**<br>**4-630CP**-(O4)Halo	100 nM<br>100 nM	Abberior expert line	561<br>640	2	100x NA 1.4 oil	30	580 – 630<br>650 - 720
Figure 6b	**4-580CP-Hoechst**<br>**4-625CP-BG**	100 nM<br>100 nM	Abberior expert line	561<br>640	2	100x NA 1.4 oil	20	580 – 630<br>650 - 720
Figure 6c	**4-DAllR-CTX**<br>**4-630CP-JAS**<br>**4-700SiR-TPP**	10 nM<br>10 nM<br>10 nM	Abberior facility line	561<br>640<br>705	3	60x NA 1.42 oil	30	571 – 630<br>650 - 695<br>715 - 755
Figure 6d-f	**4-505R-Hoechst**<br>**4-TMR-JAS**<br>**4-685SiR-TPP**	250 nM<br>1 µM<br>1.5 µM	TCS SP8 confocal microscope	514<br>561<br>630	1.2	63x NA 1.4 oil	80x80x20	525-550<br>570-600<br>689-740
Figure S15	**5-580CP-Hoechst**<br>**SiR-SNAP**	100 nM<br>100 nM	Abberior expert line	561<br>640	3	100x NA 1.4 oil	30	580 – 630<br>650 - 720
Figure S17	**4-580CP-BG**	100 nM	Abberior expert line	561	2	100x NA 1.4 oil	15	580 - 630
	**4-610CP-BG**	100 nM	Abberior expert line	640	2	100x NA 1.4 oil	15	650 - 720
	**4-625CP-BG**	100 nM	Abberior expert line	640	2	100x NA 1.4 oil	15	650 - 720
	**4-630CP-BG**	100 nM	Abberior expert line	640	2	100x NA 1.4 oil	15	650 - 720
	**4-640CP-BG**	100 nM	Abberior expert line	640	2	100x NA 1.4 oil	15	650 - 720
	**SiR-SNAP**	100 nM	Abberior expert line	640	2	100x NA 1.4 oil	15	650 - 720
Figure S20	**4-630CP-CTX**	10 nM	Abberior expert line	640	1	100x NA 1.4 oil	40	650 - 720
	**4-630CP-CTX**	100 nM	Abberior expert line	640	1	100x NA 1.4 oil	40	650 - 720
Figure S24	**4-610CP-JAS**	10 nM	Abberior facility line	640	3	60x NA 1.42 oil	80	650 - 760
	**4-610CP-JAS**	100 nM	Abberior facility line	640	3	60x NA 1.42 oil	80	650 - 760
	**4-610CP-JAS**	1000 nM	Abberior facility line	640	3	60x NA 1.42 oil	80	650 - 760
	**4-610CP-C6-JAS**	10 nM	Abberior facility line	640	3	60x NA 1.42 oil	80	650 - 760
	**4-610CP-C6-JAS**	100 nM	Abberior facility line	640	3	60x NA 1.42 oil	80	650 - 760
	**4-610CP-C6-JAS**	1000 nM	Abberior facility line	640	3	60x NA 1.42 oil	80	650 - 760
Figure S25	**4-TMR-JAS**	100 nM	Abberior facility line	561	3	60x NA 1.42 oil	80	571 - 681
	**4-685SiR-JAS**	100 nM	Abberior facility line	705	3	60x NA 1.42 oil	80	715 - 800
	**4-630CP-JAS**	100 nM	Abberior facility line	640	3	60x NA 1.42 oil	80	650 -760
	**4-505R-JAS**	100 nM	Abberior facility line	488	3	60x NA 1.42 oil	80	498 - 608
	**4-505R-Hoe**	100 nM	Abberior facility line	488	3	60x NA 1.42 oil	80	500 - 550

Figure S26	**4-525R-Hoe**	100 nM	Abberior facility line	518	3	60x NA 1.42 oil	80	530 - 580
	**4-CFL-Hoe**	100 nM	Abberior facility line	561	3	60x NA 1.42 oil	80	575 - 625
	**4-625CP-Hoe**	100 nM	Abberior facility line	640	3	60x NA 1.42 oil	80	650 - 700
	**4-630CP-Hoe**	100 nM	Abberior facility line	640	3	60x NA 1.42 oil	80	651 - 700
	**4-685SiR-Hoe**	100 nM	Abberior facility line	705	3	60x NA 1.42 oil	80	715 - 765
Figure S27	**4-625CP-TPP**	100 nM	Abberior facility line	640	3	60x NA 1.42 oil	80	650 - 760
	**4-640CP-TPP**	100 nM	Abberior facility line	640	3	60x NA 1.42 oil	80	650 - 760
	**4-665SiR-TPP**	100 nM	Abberior facility line	640	3	60x NA 1.42 oil	80	650 - 760
	**4-685SiR-TPP**	100 nM	Abberior facility line	640	3	60x NA 1.42 oil	80	650 - 760
	**4-525R-TPP**	100 nM	Abberior facility line	518	3	60x NA 1.42 oil	80	531 - 630
	**4-685SiR-TPP**	100 nM	Abberior facility line	705	3	60x NA 1.42 oil	80	715 - 800
	**4-700SiR-TPP**	100 nM	Abberior facility line	705	3	60x NA 1.42 oil	80	715 - 800
Figure S28	**4-580R-PepA**	100 nM	Abberior facility line	561	5	60x NA 1.42 oil	80	571- 681
	**4-630CP-PepA**	100 nM	Abberior facility line	640	3	60x NA 1.42 oil	80	650 - 760
	**4-642CP-PepA**	100 nM	Abberior facility line	640	3	60x NA 1.42 oil	80	650 - 760
	**4-SiR-PepA**	100 nM	Abberior facility line	640	3	60x NA 1.42 oil	80	650 - 760
	**4-685SiR-PepA**	100 nM	Abberior facility line	705	3	60x NA 1.42 oil	80	715 - 800
	**4-700SiR-PepA**	100 nM	Abberior facility line	705	3	60x NA 1.42 oil	80	715 - 800

4-color time-lapse movies

For recording multicolor movies, the cells were incubated with the indicated probes for 1h at 37°C and imaged without washing at 37°C with 5% CO_2 flow. In case of U-2 OS vimentin-HaloTag cell line, the cells were first incubated with **4-642CP-(O_4)Halo** substrate for 1h at 37°C, washed twice, stained with the other probes and imaged without washing.

The time-lapse series were recorded on the Abberior Facility Line microscope in the confocal imaging mode under control of Imspector software. The 2-step sequential line scanning was used: 561 nm and 700 nm channels were recorded in the first step and 488 nm and 640 nm channels were recorded during the second step. Pixel size was 100 nm, pixel dwell time – 1 µs, frame rate – 0.33 fps. 200 frames of 50×50 µm were acquired for each movie and the final videos were rendered at 20 fps.

Processing and visualization of the acquired images

All acquired or reconstructed images were processed and visualized using Fiji[7]. Line profiles were measured using the "straight line" tool with the line width set to 3 pixels. To define microtubule diameter,

line profiles were fitted with Gaussian and Lorentzian distributions for confocal and STED images respectively. For 3D STED images resolution, perpendicular microtubule profile images in xz and zy planes were cropped using Fiji BigDataViewer Plugin and fitted using 2D Gaussian distribution. Fitting routines were optimized and performed with Python3 language. Stitching and deconvolution of images were performed using SVI Huygens Essential software package.

General chemical experimental information and synthesis methods

NMR spectra were recorded at 25 °C with an Agilent 400-MR spectrometer at 400.06 MHz (^{1}H) and 100.60 MHz (^{13}C) and Varian INOVA 600 (I600) spectrometer at 599.74 MHz (^{1}H) and are reported in ppm. All ^{1}H and ^{13}C spectra are referenced to tetramethylsilane (δ = 0 ppm) using the residual signals of the solvents according to the values reported in literature[8]. Multiplicities of signals are described as follows: s = singlet, d = doublet, t = triplet, q = quartet, p = pentet, m = multiplet or overlap of non-equivalent resonances; br = broad signal. Coupling constants (J) are given in Hz. All NMR spectra were processed with MestRenova 11.0.4 software.

ESI-MS were recorded on a Varian 500-MS spectrometer (Agilent). ESI-HRMS were recorded on a MICROTOF spectrometer (Bruker) equipped with ESI ion source (Apollo) and direct injector with LC autosampler Agilent RR 1200.

Analytical LC–MS analysis was performed on an Agilent 1260 Infinity II LC/MS system equipped with an autosampler, diode array detector WR, fluorescence detector Spectra and Infinity Lab LC/MSD 6100 series quadruple with API electrospray. Analysis was done by using an Agilent Zorbax SB-C18 RRHT, 2.1 x 50 mm, 1.8 µm threaded column and SUPELCO Titan C18, 2.1 x 75 mm, 1.9 µm column with A: 25 mM $HCOONH_4$ (pH = 3.6) aqueous buffer and B: MeOH

Preparative HPLC was performed on a combined Agilent1260/1290 Infinity II preparative system equipped with a 1290 Infinity II open-bed sampler (G7169B)/fraction collector (G7159B), 1260 Infinity II preparative binary pump (G7161A), 1260 Infinity II multiple wavelength detector (G7165A) and with Agilent 5 Prep-C18, 5 µm, 100 x 50 mm preparative column.

9,9'-methylenebis(2,3,6,7-tetrahydro-1H,5H-pyrido[3,2,1-ij]quinolin-8-ol) (**SI-1**):

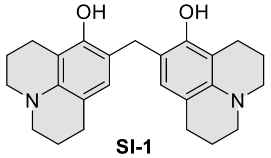

SI-1

To the ice bath cooled solution of 8-hydroxyjulolidine (1 g, 5.3 mmol) in methanol (10 mL) hydrochloric acid was added (0.5 mL, 32%) and stirred for 10 min. Formalin (1 mL, 16%) was then added to the reaction mixture and the resulting mixture was allowed to stand overnight. The mixture was poured into water (40 mL) and was neutralized with saturated Na_2CO_3 solution. The mixture was extracted with DCM (3x 50 mL), the combined extracts were dried over Na_2SO_4, filtered and solvent was removed under reduced pressure. The product was purified by flash column chromatography (Teledyne Isco RediSep Rf 40 g; gradient 2% to 40% hexane – EtOAc), fractions containing the product were evaporated to give 0.7g (68%) of off-white solid.

^{1}H NMR (400 MHz, d$_6$-DMSO) δ 8.06 (s, 2H), 6.43 (s, 2H), 3.50 (s, 2H), 2.95 (dt, *J* = 9.9, 5.2 Hz, 8H), 2.58 – 2.51 (m, 8H), 1.85 – 1.76 (m, 8H). ^{13}C NMR (101 MHz, d$_6$-DMSO) δ 149.4, 141.8, 127.3, 116.6, 112.9, 108.9, 49.6, 49.0, 29.3, 26.7, 22.2, 21.6, 21.6. ESI-MS, positive mode: m/z = 391.2 $[M+H]^+$. HRMS (ESI) calcd for $C_{25}H_{31}N_2O_2$ $[M+H]^+$ 391.2380, found 391.2375.

***2,3,6,7,12,13,16,17*-octahydro-1*H*,5*H*,9*H*,11*H*,15*H*-Xantheno[2,3,4-ij:5,6,7-i'j']diquinolizine (SI-2)**:

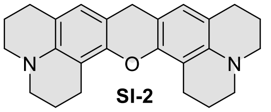

SI-2

Compound **SI-1** (0.7g, 1.8 mmol) was added to concentrated sulphuric acid (5 mL). The resulting solution was heated at 95°C for 3 hours. The reaction was allowed to cool to room temperature before being poured onto ice. The pH was adjusted to 5 with 40% NaOH solution whilst keeping the mixture cold. Then mixture was extracted with DCM (3x 100 mL) the combined extracts were dried over Na_2SO_4, filtered and the solvent was removed under reduced pressure. The product was purified by flash column chromatography (Teledyne Isco RediSep Rf 40 g; gradient 2% to 40% hexane – EtOAc), fractions containing the product were evaporated to give 0.435g (65%) of off-white solid.

^{1}H NMR (400 MHz, CD_2Cl_2) δ 6.55 (s, 2H), 3.69 (s, 2H), 3.09 – 3.03 (m, 8H), 2.80 (t, *J* = 6.7 Hz, 4H), 2.70 (t, *J* = 6.6 Hz, 4H), 2.02 – 1.92 (m, 8H). ^{13}C NMR (101 MHz, CD_2Cl_2) δ 148.6, 142.9, 126.4, 116.8, 109.7, 109.0, 50.8, 50.3, 27.7, 27.2, 23.2, 22.5, 21.9. ESI-MS, positive mode: m/z = 373.2$[M+H]^+$.HRMS (ESI) calcd for $C_{25}H_{29}N_2O$ $[M+H]^+$ 373.2274, found 373.2277.

6-Bromoindoline (**SI-3**):

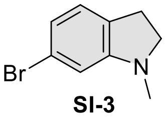

SI-3

6-Bromoindole (3.5 g, 17.8 mmol) was dissolved in glacial acetic acid (50 mL) and $NaCNBH_3$ was added carefully in small portions (intense bubbling). Once the addition was complete, paraformaldehyde (5.3 g, 178 mmol) was added and the reaction was stirred for 3-5 hours. The reaction course was monitored by TLC hex:EtOAc (8:2). Then the

reaction mixture was poured into water (200 mL) and neutralized with saturated Na_2CO_3 solution. The mixture was extracted with DCM (3x 100 mL), combined extracts were dried with Na_2SO_4, filtered and solvent was removed under reduced pressure. The product was purified by flash column chromatography (Teledyne Isco RediSep Rf 80 g; gradient 2% to 40% hexane – EtOAc), fractions containing the product were evaporated to give 2.6 g (69%) of colorless oil. This compound has been obtained previously[9].

^{1}H NMR (400 MHz, $CDCl_3$) δ 6.90 (dt, *J* = 7.7, 1.1 Hz, 1H), 6.75 (dd, *J* = 7.7, 1.7 Hz, 1H), 6.56 (d, *J* = 1.8 Hz, 1H), 3.33 (t, *J* = 8.3 Hz, 2H), 2.89 (td, *J* = 8.2, 1.1 Hz, 2H), 2.74 (s, 3H). ^{13}C NMR (101 MHz, $CDCl_3$) δ 154.9, 129.4, 125.4, 121.2, 120.2, 110.1, 56.2, 35.8, 28.4. ESI-MS, positive mode: m/z = 212.0 $[M+H]^+$. HRMS (ESI) calcd for $C_9H_{11}BrN$ $[M+H]^+$ 212.0069, found 212.0073.

1-methyl-6-(prop-1-en-2-yl)indoline (**SI-4**):

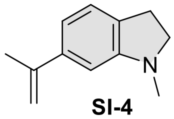

SI-4

A mixture of **SI-3** (2.6 g, 12.3 mmol), potassium isopropenyltrifluoroborate (2.2 g, 14.7 mmol, 1.2 equiv) and $Pd(dppf)Cl_2$ (301 mg, 0.37 mmol, 3 mol %) in 1,4-dioxane (45 mL) in a 100 mL round bottom flask was purged on a Schlenk line and filled with argon. Aqueous NaOH (5 mL of 2 M solution) was then injected, and the yellowish solution turned brown. The mixture was then heated up to 100 °C and stirred for 3 h, cooled down to rt, diluted with water (200 mL) and extracted with DCM (3×100 mL). The combined organic layers were washed with brine and dried over Na_2SO_4. The product was isolated by flash column chromatography (Teledyne Isco RediSep Rf 80 g; gradient 2% to 20% EtOAc – hexane, Teledyne Isco RediSep Rf 40 g, gradient 10% to 70% hexane – DCM) fractions containing the product were evaporated to give yellowish oil, yield 1.4 g (66%).

^{1}H NMR (400 MHz, $CDCl_3$) δ 7.13 – 7.01 (m, 1H), 6.82 (dd, *J* = 7.5, 1.6 Hz, 1H), 6.62 (d, *J* = 1.5 Hz, 1H), 5.38 – 5.30 (m, 1H), 5.05 (p, *J* = 1.6 Hz, 1H), 3.34 (t, *J* = 8.2 Hz, 2H), 2.96 (t, *J* = 8.2 Hz, 2H), 2.81 (s, 3H), 2.20 – 2.12 (m, 3H). ^{13}C NMR (101 MHz, $CDCl_3$) δ 144.2, 141.2, 130.1, 124.0, 115.9, 111.7, 104.8, 56.5, 36.4, 28.6, 22.3. ESI-MS, positive mode: m/z = 174.1 $[M+H]^+$. HRMS (ESI) calcd for $C_{12}H_{16}N$ $[M+H]^+$ 174.1277, found 174.1278.

1-methylindoline (**SI-5**):

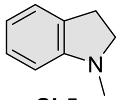

SI-5

1-Methylindole (6 g, 0.046 mol) was dissolved in AcOH (25 mL). The obtained solution was cooled in ice bath and $NaCNBH_3$ (3.5 g, 0.05 mol) was slowly added in portions (excessive bubbling of reaction mixture). After addition was complete ice bath was removed and stirring was continued for 4 h. The completion of reaction was confirmed by TLC 9:1 hexane:EtOAc. Mixture was quenched with water (25 mL). Acetic acid and water were evaporated on rotary evaporator and 1M NaOH (50 mL). Obtained mixture was extracted with DCM (3 x 100 mL). The organic phase was separated, dried

over Na_2SO_4 and evaporated. Product was purified by flash chromatography (Teledyne Isco RediSep Rf 80 g; gradient 20% to 100% hexane – DCM), fractions containing the product were evaporated to give 5.2 g (85%) of yellowish liquid. The synthesis of 1-methylindoline (**SI-5**) was preformed based on previously published procedure[10].

1-methylindoline-5-carbaldehyde (**SI-6**):

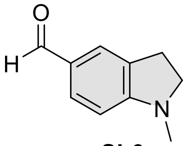

SI-6

1-Methylindoline (5.2 g, 0.039 mol) was dissolved in dry DMF (10 mL) and the solution was cooled in the ice bath. Phosphorous oxychloride (3.6 mL, 0.039 mol) was added dropwise over the period of 30 min. Then ice bath was removed and the mixture was stirred for 4h at 85°C. After cooling the mixture was quenched with an ice water (50 mL) and pH adjusted to 8 with 1M NaOH solution. The mixture was extracted with DCM (3x 100 mL), organic layers were washed with $NaHCO_3$ solution, dried over Na_2SO_4. The solvent was removed on rotary evaporator to obtain 6.2 g (99%) of yellow solid, which was used in next step without purification. The synthesis of 1-methylindoline-5-carbaldehyde (**SI-6**) was performed based on previously published procedure[11].

(1-methylindolin-5-yl)methanol (**SI-7**):

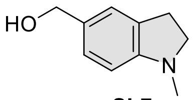

SI-7

1-methylindoline-5-carbaldehyde (**SI-6**) (6.2 g, 38.5 mmol) was dissolved in MeOH (40 mL) and solution was cooled in an ice bath. $NaBH_4$ (1.3 g, 34.2 mmol) was added in small portions. After addition was complete, reaction mixture was left to warm to room temperature. After 1h of stirring at rt water (5 mL) was added and stirring was continued for 10 min. Then MeOH was removed on rotary evaporator, water was added to the residue and mixture was extracted with EtOAc (3x 100 mL), washed with brine and dried over Na_2SO_4. The solvent was evaporated and the product was purified by flash column chromatography (Teledyne Isco RediSep Rf 80 g; gradient 20% to 80% hexane – EtOAc). White solid, yield 5.7 g (91%). This compound has been obtained previously[12].

^{1}H NMR (400 MHz, d_6-DMSO) δ 7.00 (s, 1H), 6.94 (d, J = 7.9 Hz, 1H), 6.43 (d, J = 7.9 Hz, 1H), 4.87 – 4.80 (m, 1H), 4.33 (d, J = 5.6 Hz, 2H), 3.19 (t, J = 8.2 Hz, 2H), 2.83 (t, J = 8.2 Hz, 2H), 2.66 (s, 3H). ^{13}C NMR (101 MHz, d_6-DMSO) δ 152.4, 131.6, 129.8, 126.0, 123.3, 106.5, 63.3, 55.9, 36.1, 28.2. ESI-MS, positive mode: m/z = 164.1 $[M+H]^+$. HRMS (ESI) calcd for $C_{10}H_{14}NO$ $[M+H]^+$ 164.1069, found 164.1068

2-(3-(dimethylamino)phenyl)propan-2-ol (**SI-8**):

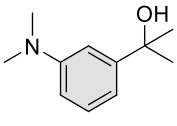

SI-8

Solution of 3-Bromo-dimethylaniline (4g, 20 mmol) in THF (50 mL) was cooled to -78°C in dry ice/acetone cooling bath. Then *n*-BuLi (8 mL, 2.5M) was added through a syringe and mixture was stirred for 30 min at -78°C followed by the addition of dry acetone (2 mL, 27 mmol). After addition was complete the reaction mixture was stirred at -78° for 15 min and then cooling bath was removed and the mixture was allowed to warm to rt. Stirring was continued for 30 min at rt. Then HCl (2mL, 1M) was added to the reaction mixture through a syringe. THF was partially evaporated, water was added and the mixture was extracted with EtOAc (4x 50 mL), washed with brine and dried over Na_2SO_4. The solvent was evaporated and the product was purified by flash column chromatography (Teledyne Isco RediSep Rf 80 g; gradient 10% to 70% hexane – EtOAc) to give 2.88 g of yellowish liquid in 80% yield. This compound has been obtained previously[13].

^{1}H NMR (400 MHz, $CDCl_3$) δ 7.23 (t, *J* = 7.9 Hz, 1H), 6.97 (t, *J* = 2.2 Hz, 1H), 6.83 (d, *J* = 7.7 Hz, 1H), 6.68 (dd, *J* = 8.2, 2.3 Hz, 1H), 2.97 (s, 6H), 1.59 (s, 6H). ^{13}C NMR (101 MHz, $CDCl_3$) δ 150.5, 150.4, 129.0, 113.4, 111.4, 109.1, 72.9, 41.0, 31.9. ESI-MS, positive mode: m/z = 180.1 $[M+H]^+$. HRMS (ESI) calcd for $C_{11}H_{18}NO$ $[M+H]^+$ 180.1383, found 180.1388.

N,N-dimethyl-3-(prop-1-en-2-yl)aniline (**SI-9**):

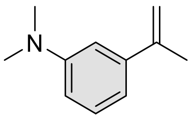

SI-9

Compound **SI-8** (2.88 g, 16.1 mmol) was dissolved in acetic acid (30 mL) and 1 mL of concentrated H_2SO_4 was added. The obtained mixture was heated to 100°C for 1h. Reaction course was monitored by TLC hex:EtOAc (8:2). Once reaction was complete mixture was poured to water and neutralized with saturated Na_2CO_3. Mixture was extracted with EtOAc (4x 100mL), washed with brine and dried over Na_2SO_4. The solvent was evaporated and product was purified by flash column chromatography (Teledyne Isco RediSep Rf 80 g; gradient 2% to 20% hexane – EtOAc) to give 2.0 g of light yellow liquid in a 77% yield. This compound has been obtained previously[13].

^{1}H NMR (400 MHz, $CDCl_3$) δ 7.26 (t, *J* = 8.1 Hz, 1H), 6.95 – 6.86 (m, 2H), 6.77 – 6.71 (m, 1H), 5.39 (dt, *J* = 1.5, 0.8 Hz, 1H), 5.11 (p, *J* = 1.5 Hz, 1H), 3.02 (s, 6H), 2.21 (dd, *J* = 1.5, 0.8 Hz, 3H). ^{13}C NMR (101 MHz, $CDCl_3$) δ 150.7, 144.4, 142.5, 129.0, 114.6, 112.2, 112.2, 110.3, 40.9, 22.2. ESI-MS, positive mode: m/z = 162.1 $[M+H]^+$. HRMS (ESI) calcd for $C_{11}H_{16}N$ $[M+H]^+$ 162.1277, found 162.1268.

2,3,6,7-tetrahydro-1H,5H-pyrido[3,2,1-ij]quinoline-9-carbaldehyde (**SI-10**):

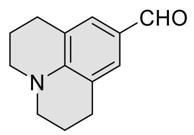

SI-10

Phosphorous oxychloride (2.14 mL, 22.9 mmol) was added dropwise to anhydrous DMF (6 mL) whilst mixture was cooled in an ice bath. The obtained mixture was stirred at room temperature for 30 minutes. Solution of julolidine (3.6 g, 20.8 mmol) in DMF (5 mL) was added and the reaction mixture was heated at 90°C for 4 hours. Then mixture was cooled and poured to 100 mL water and neutralized with saturated Na_2CO_3 solution. Obtained mixture was extracted with EtOAc (3x 100mL), washed with brine, dried over Na_2SO_4 and filtered. The solvent was evaporated and product was purified by flash column chromatography (Teledyne Isco RediSep Rf 40 g; gradient 10% to 70% hexane – EtOAc) to give 2.7 g of yellow solid in a 65% yield. This compound has been obtained previously[14].

^{1}H NMR (400 MHz, *d*$_6$-DMSO) δ 9.50 (s, 1H), 7.21 (s, 2H), 3.27 (t, *J* = 5.7 Hz, 4H), 2.69 (t, *J* = 6.3 Hz, 4H), 1.89 – 1.82 (m, 4H). ^{13}C NMR (101 MHz, *d*$_6$-DMSO) δ 189.2, 147.5, 128.8, 123.3, 119.9, 49.3, 27.0, 20.7. ESI-MS, positive mode: m/z = 202.1 $[M+H]^+$. HRMS (ESI) calcd for $C_{13}H_{16}NO$ $[M+H]^+$ 202.1226, found 202.1236.

(2,3,6,7-tetrahydro-1H,5H-pyrido[3,2,1-ij]quinolin-9-yl)methanol (**SI-11**):

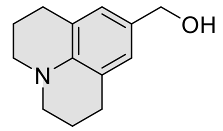

SI-11

Carbaldehyde **SI-10** (1.2 g, 5.97 mmol) was dissolved in 30 mL of MeOH and $NaBH_4$ (247 mg, 6.5 mmol) was added in small portions at room temperature over the course of 30 minutes. Once addition was complete the mixture was stirred for 1 hour. Reaction was quenched by addition of water (5 mL) and MeOH was evaporated on rotary evaporator. Water was poured on residue and the mixture was extracted with EtOAc (3x 50 mL), washed with brined, dried over Na_2SO_4 and filtered. The solvent was evaporated and product was purified by flash column chromatography (Teledyne Isco RediSep Rf 40 g; gradient 10% to 70% hexane – EtOAc) to give 0.93 g of white solid in a 77% yield. The compound easily oxidizes and we recommend to store it under inert gas at -15°C. This compound has been obtained previuosly[15].

^{1}H NMR (400 MHz, *d*$_6$-DMSO) δ 6.63 (s, 2H), 4.72 (t, *J* = 5.6 Hz, 1H), 4.23 (d, *J* = 5.6 Hz, 2H), 3.05 (t, *J* = 5.6 Hz, 4H), 2.65 (t, *J* = 6.5 Hz, 4H), 1.89 – 1.83 (m, 4H). ^{13}C NMR (101 MHz, *d*$_6$-DMSO) δ 141.6, 129.3, 125.6, 120.6, 63.1, 49.4, 27.2, 21.8. ESI-MS, positive mode: m/z = 204.2 $[M+H]^+$. HRMS (ESI) calcd for $C_{13}H_{18}NO$ $[M+H]^+$ 204.1383, found 204.1390.

8-bromo-2,3,6,7-tetrahydro-1H,5H-pyrido[3,2,1-ij]quinolone (**SI-12**):

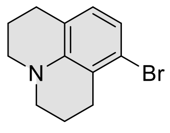

SI-12

3-Bromoaniline (6.3 mL, 58 mmol), Na_2CO_3 (24.6 g, 232 mmol) and 1-bromo-3-chloropropane (110 mL, 1.11 mol) were heated with stirring at 140°C for 48 hours. Then mixture was cooled, water (250 mL) was added and products were extracted with DCM (3x 150 mL). Combine organic extracts were dried over Na_2SO_4 and filtered. Filtrate was concentrated and excess of 1-bromo-3-chloropropane was removed. The crude mixture was dissolved in DMF (30 mL) and heated at 160°C for 48 hours. After cooling to room temperature solution of NaOH was added (200 mL, 1M) and mixture was extracted with EtOAc (3x 250 mL). The combined extracts were dried over Na_2SO_4, filtered and solvent was removed on rotary evaporator. The product was purified by flash column chromatography (Teledyne Isco RediSep Rf 80 g; gradient 1% to 10% hexane – EtOAc) to give 4.6 g of slightly yellow solid in a 31% yield. This compound has been obtained previously[16].

^{1}H NMR (400 MHz, *d$_6$*-DMSO) δ 6.69 – 6.60 (m, 2H), 3.12 – 3.06 (m, 4H), 2.63 (dt, *J* = 11.7, 6.8 Hz, 4H), 1.91 – 1.81 (m, 4H). ^{13}C NMR (101 MHz, *d$_6$*-DMSO) δ 144.5, 127.8, 122.0, 120.4, 119.5, 118.5, 49.2, 48.8, 28.0, 27.0, 21.3, 21.2. ESI-MS, positive mode: m/z = 252.1 [M+H]$^+$. HRMS (ESI) calcd for $C_{12}H_{15}BrN$, [M+H]$^+$ 252.0382, found 252.0389.

8-(prop-1-en-2-yl)-2,3,6,7-tetrahydro-1H,5H-pyrido[3,2,1-ij]quinoline (**SI-13**):

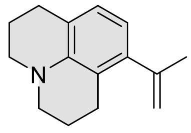

SI-13

A mixture of **SI-12** (2.5 g, 9.9 mmol), potassium isopropenyltrifluoroborate (1.75 g, 11.9 mmol, 1.2 equiv) and Pd(dppf)Cl_2 (245 mg, 0.3 mmol, 3 mol %) in 1,4-dioxane (45 mL) in a 100 mL round bottom flask was purged on a Schlenk line and filled with argon. Aqueous NaOH (5 mL of 2 M solution) was then injected, and the yellowish solution turned brown. The mixture was then heated up to 100 °C and stirred for 3 h, cooled down to rt, diluted with water (200 mL) and extracted with DCM (3× 100 mL). The combined organic layers were washed with brine and dried over Na_2SO_4. The product was isolated by flash column chromatography (Teledyne Isco RediSep Rf 80 g; gradient 1% to 10% EtOAc – hexane, Teledyne Isco RediSep Rf 40 g, gradient 10% to 70% hexane – DCM) resulting in light yellow oil, yield 1.7 g (82%).

^{1}H NMR (400 MHz, *d$_6$*-DMSO) δ 6.64 (d, *J* = 7.5 Hz, 1H), 6.21 (d, *J* = 7.5 Hz, 1H), 5.09 (dq, *J* = 3.0, 1.5 Hz, 1H), 4.69 (dq, *J* = 2.6, 0.8 Hz, 1H), 3.08 (q, *J* = 5.8 Hz, 4H), 2.61 (dt, *J* = 14.5, 6.8 Hz, 4H), 1.91 (dd, *J* = 1.5, 0.9 Hz, 3H), 1.88 – 1.79 (m, 4H). ^{13}C NMR (101 MHz, *d$_6$*-DMSO) δ 146.4, 142.9, 141.6, 126.5, 120.1, 117.8, 115.3, 114.2, 50.1, 49.7, 27.8, 25.4, 24.8, 22.1. ESI-MS, positive mode: m/z = 214.2 [M+H]$^+$. HRMS (ESI) calcd for $C_{15}H_{20}N$ [M+H]$^+$ 214.1590, found 214.1592.

7-bromo-2,2,4-trimethyl-1,2-dihydroquinoline (**SI-14**):

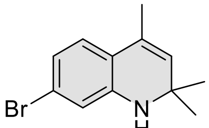
SI-14

3-Bromoaniline (8.65 mL, 80 mmol), mesityl oxide (1.3 mL, 160 mmol), I_2 (254 mg, 1 mmol) was dissolved in toluene (40 mL) and refluxed with stirring for 8h. Then another portion of I_2 (254 mg, 1 mmol) was added and reflux was continued for another 16h. Reaction course was monitored by TLC (hexane:EtOAc 9:1). Once reaction was complete the solvent and the excess of mesityl oxide was evaporated and the product was purified by flash column chromatography (Teledyne Isco RediSep Rf 120 g; gradient 1% to 10% EtOAc – hexane) to give 8.5 g of yellow oil in 42% yield. This compound has been obtained previously[15].

^{1}H NMR (400 MHz, d_6-DMSO) δ 6.83 (d, J = 7.9 Hz, 1H), 6.59 (d, J = 2.0 Hz, 1H), 6.54 (dd, J = 8.1, 2.0 Hz, 1H), 6.11 (s, 1H), 5.33 – 5.27 (m, 1H), 1.86 (d, J = 1.5 Hz, 3H), 1.19 (s, 6H). ^{13}C NMR (101 MHz, d_6-DMSO) δ 145.7, 128.6, 126.8, 124.9, 120.9, 119.2, 117.4, 114.0, 51.4, 31.0, 18.1. ESI-MS, positive mode: m/z = 252.1 $[M+H]^+$. HRMS (ESI) calcd for $C_{12}H_{15}BrN$ $[M+H]^+$ 252.0382, found 252.0385.

7-bromo-1,2,2,4-tetramethyl-1,2-dihydroquinoline (**SI-15**):

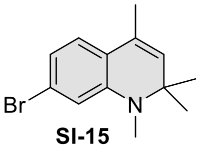
SI-15

A mixture of **SI-14** (5g, 19.8 mmol) CH_3I (2.5 mL, 40 mmol) and K_2CO_3 (5.5g, 40 mmol) in MeCN (50mL) was stirred at 50°C overnight. Reaction course was monitored by TLC (hexane:EtOAc 95:5). Reaction Mixture was cooled and filtered. Filtrate was collected and evaporated, product was purified by flash column chromatography (Teledyne Isco RediSep Rf 120 g; gradient 0% to 10% EtOAc – hexane) to give 3.6g of yellow oil in 68% yield. This compound has been obtained previously[17].

^{1}H NMR (400 MHz, CD_2Cl_2) δ 6.87 (d, J = 8.0 Hz, 1H), 6.71 (dd, J = 8.0, 1.9 Hz, 1H), 6.60 (d, J = 1.9 Hz, 1H), 5.31 (q, J = 1.4 Hz, 2H), 2.76 (s, 3H), 1.94 (d, J = 1.4 Hz, 3H), 1.29 (s, 6H). ^{13}C NMR (101 MHz, CD_2Cl_2) δ 146.5, 130.2, 127.3, 124.2, 122.2, 122.0, 118.3, 113.0, 56.5, 30.6, 27.1, 18.1. ESI-MS, positive mode: m/z = 266.1 $[M+H]^+$. HRMS (ESI) calcd for $C_{13}H_{17}NBr$ $[M+H]^+$ 266.0539, found 266.0535.

1,2,2,4-tetramethyl-7-(prop-1-en-2-yl)-1,2-dihydroquinoline (**SI-16**):

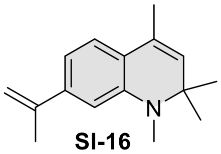
SI-16

A mixture of **SI-15** (1.5 g, 5.6 mmol), potassium isopropenyltrifluoroborate (1.0 g, 6.8 mmol, 1.2 eq) and Pd(dppf)Cl_2 (137 mg, 0.17 mmol, 3 mol %) in 1,4-dioxane (45 mL) in a 100 mL round bottom flask was purged on a Schlenk line and filled with argon. Aqueous NaOH (5 mL of 2 M solution) was then injected, and the yellowish solution turned brown. The mixture was then heated up to 100°C and stirred overnight, cooled down to rt, diluted with water (200 mL) and extracted with EtOAc (3× 100 mL). The combined organic layers were washed with brine and dried

over Na_2SO_4. The product was isolated by flash column chromatography (, Büchi Reveleris HP silica 40 g, isocratic conditions hexane:EtOAc:DCM 96:2:2) to give 0.7 g of light yellow oil in a 55% yield.

^{1}H NMR (400 MHz, CD_2Cl_2) δ 7.00 (d, *J* = 7.8 Hz, 1H), 6.75 (dd, *J* = 7.8, 1.8 Hz, 1H), 6.61 (d, *J* = 1.8 Hz, 1H), 5.35 (dd, *J* = 1.7, 0.8 Hz, 1H), 5.31 (q, *J* = 1.6 Hz, 1H), 5.03 (p, *J* = 1.5 Hz, 1H), 2.82 (s, 3H), 2.14 (dd, *J* = 1.5, 0.8 Hz, 3H), 1.97 (d, *J* = 1.4 Hz, 4H), 1.29 (s, 6H). ^{13}C NMR (101 MHz, CD_2Cl_2) δ 145.7, 144.7, 141.9, 130.7, 128.4, 123.4, 123.3, 114.1, 111.7, 108.3, 56.8, 31.0, 27.4, 22.2, 18.8. ESI-MS, positive mode: m/z = 228.2 $[M+H]^+$. HRMS (ESI) calcd for $C_{16}H_{22}N$ $[M+H]^+$ 228.1747, found 228.1749.

Bis(6-bromo-1-methylindolin-5-yl)methane (**SI-17**):

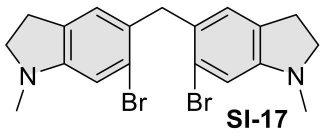

SI-17

Compound **SI-3** (2.1 g, 10 mmol) was dissolved in 20 mL of glacial acetic acid then formaldehyde solution was added (4.5 mL, 37%). Reaction mixture was heated to 40°C for 15-20 min. The color of mixture changed from colorless to green/yellow. Reaction completion was confirmed by TLC (Hexane:EtOAc 9:1). Reaction mixture was cooled, poured into water and neutralized with NaOH solution. Mixture was extracted with DCM (3x 50 mL), washed with brine, dried over Na_2SO_4 and filtered. Filtrate was concentrated on rotary evaporator, the mixture was deposited on celite and product was purified by flash column chromatography (Büchi Reveleris HP silica 40 g, gradient 5% to50% hexane – EtOAc) to give 1.35 g of white solid in a 62% yield. This compound has been obtained previously[18].

^{1}H NMR (400 MHz, *d$_6$*-DMSO) δ 6.70 (s, 2H), 6.68 (s, 2H), 3.84 (s, 2H), 3.24 (t, *J* = 8.2 Hz, 4H), 2.76 (t, *J* = 8.2 Hz, 4H), 2.68 (s, 6H). ^{13}C NMR (101 MHz, *d$_6$*-DMSO) δ 153.2, 130.1, 126.8, 125.7, 122.3, 110.2, 55.6, 40.0, 35.5, 27.7. ESI-MS, positive mode: m/z = 437.0 $[M+H]^+$. HRMS (ESI) calcd for $C_{19}H_{21}N_2Br_2$ $[M+H]^+$ 437.0046, found 437.0042.

Bis(7-bromo-1,2,2,4-tetramethyl-1,2-dihydroquinolin-6-yl)methane (**SI-18**):

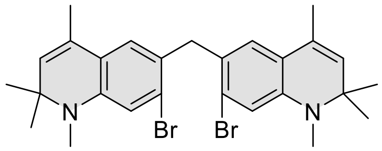

SI-18

Compound **SI-15** (3.7 g, 13.9 mmol) was dissolved in AcOH (30 mL) and solution of formaldehyde (5.6 mL, 37%) was added. The reaction mixture was stirred at 60°C for 30 min. Reaction course was followed by TLC (Hexane:EtOAc 95:5). Once reaction was complete reaction mixture was cooled, poured into water and neutralized with NaOH solution. Mixture was extracted with DCM (3x 50 mL), washed with brine, dried over Na_2SO_4 and filtered. Filtrate was concentrated on rotary evaporator, the mixture was deposited on celite and product was purified by flash column chromatography (Büchi Reveleris HP silica 80 g, gradient 0% to 10% hexane – EtOAc) to give 2.9 g of off-white solid in a 76% yield. This compound has been obtained previuosly[9].

^1H NMR (400 MHz, $CDCl_3$) δ 6.77 (s, 2H), 6.71 (s, 2H), 5.28 (q, *J* = 1.5 Hz, 2H), 3.98 (s, 2H), 2.77 (s, 6H), 1.84 (d, *J* = 1.4 Hz, 6H), 1.28 (s, 12H). ^{13}C NMR (101 MHz, $CDCl_3$) δ 144.8, 130.6, 127.9, 126.9, 125.3, 124.7, 123.0, 114.5, 56.5, 40.0, 30.8, 27.2, 18.5. ESI-MS, positive mode: m/z = 589.0 $[M+H]^+$. HRMS (ESI) calcd for $C_{26}H_{37}Br_2N_2Si$ $[M+H]^+$ 587.1087, found 587.1014.

3-bromo-4-((8-bromo-2,3,6,7-tetrahydro-1H,5H-pyrido[3,2,1-ij]quinolin-9-yl)methyl)-N,N-dimethylaniline (**SI-19**):

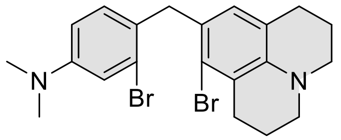

SI-19

Compound **SI-12** (1.5g, 5.95mmol), (2-bromo-4-(dimethylamino)phenyl)methanol[17] (1.37 g, 5.95 mmol) and $BF_3 \cdot OEt_2$ (1.5 mL, 11.9 mmol) were dissolved in DCM (40 mL), and the mixture was stirred at room temperature for 5h. The reaction was quenched with water and the solution was extracted with DCM (3x 50mL). The organic layer was washed with brine, dried over Na_2SO_4, filtered and filtrate was evaporated to dryness. The residue was purified by flash column chromatography (Teledyne Isco RediSep Rf 80 g; gradient 5% to 50% EtOAc – hexane + 5% constant DCM additive) to give 1.85 g of white solid in a 67% yield.

^1H NMR (400 MHz, $CDCl_3$) δ 6.93 (d, *J* = 2.6 Hz, 1H), 6.82 (d, *J* = 8.6 Hz, 1H), 6.58 (dd, *J* = 8.6, 2.7 Hz, 1H), 6.45 (s, 1H), 3.94 (s, 2H), 3.14 – 3.04 (m, 4H), 2.91 (s, 6H), 2.80 (t, *J* = 6.7 Hz, 2H), 2.62 (t, *J* = 6.6 Hz, 2H), 2.06 – 1.85 (m, 4H). ^{13}C NMR (101 MHz, $CDCl_3$) δ 150.7, 143.7, 131.1, 129.0, 127.6, 127.1, 126.0, 125.8, 121.7, 121.4, 116.5, 112.3, 50.6, 50.0, 41.4, 40.8, 30.0, 28.1, 22.8, 22.5. ESI-MS, positive mode: m/z = 465.0 $[M+H]^+$. HRMS (ESI) calcd for $C_{21}H_{25}Br_2N_2$ $[M+H]^+$ 465.0360, found 465.0360.

3-bromo-4-((6-bromo-1-methylindolin-5-yl)methyl)-N,N-dimethylaniline (**SI-20**):

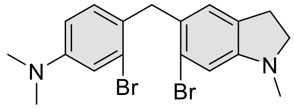

SI-20

Compound **SI-3** (2.5 g, 11.8 mmol), (2-bromo-4-(dimethylamino)phenyl)methanol[17] (2.7 g, 11.8 mmol) and $BF_3 \cdot OEt_2$ (2.95 mL, 23.6 mmol) were dissolved in DCM (100 mL), and the mixture was stirred at room temperature for 5h. The reaction was quenched with water and the solution was extracted with DCM (3x 100mL). The organic layer was washed with brine, dried over Na_2SO_4, filtered and filtrate was evaporated to dryness. The residue was purified by flash column chromatography (Teledyne Isco RediSep Rf 80 g; gradient 2% to 40% EtOAc – hexane + 5% constant DCM additive) to give 3.8 g of white solid in a 76% yield. This compound has been obtained previuosly[17].

^1H NMR (400 MHz, CD_2Cl_2) δ 6.93 (d, *J* = 2.7 Hz, 1H), 6.84 (d, *J* = 8.5 Hz, 1H), 6.70 (s, 1H), 6.64 (s, 1H), 6.59 (dd, *J* = 8.6, 2.7 Hz, 1H), 3.96 (s, 2H), 3.29 (t, *J* = 8.2 Hz, 2H), 2.91 (s, 6H), 2.81 (td, *J* = 8.2, 1.1 Hz, 2H), 2.72 (s, 3H). ^{13}C NMR (101 MHz, CD_2Cl_2) δ 153.8, 150.8, 131.2, 131.0, 128.3, 127.5, 126.5, 125.9, 123.4,

116.5, 112.3, 111.1, 56.8, 40.8, 40.7, 36.3, 28.9. ESI-MS, positive mode: m/z = 425.0 $[M+H]^+$. HRMS (ESI) calcd for $C_{18}H_{21}Br_2N_2$ $[M+H]^+$ 425.0046, found 425.0045.

3-bromo-4-((7-bromo-1,2,2,4-tetramethyl-1,2-dihydroquinolin-6-yl)methyl)-N,N-dimethylaniline (**SI-21**):

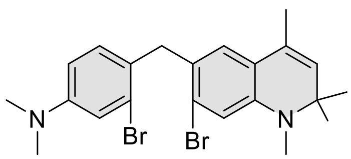

SI-21

Compound **SI-15** (1.5 g, 5.6 mmol), (2-bromo-4-(dimethylamino)phenyl)methanol[17] (1.3 g, 5.6 mmol) and $BF_3{\cdot}OEt_2$ (1.4 mL, 11.2 mmol) were dissolved in DCM (50 mL), and the mixture was stirred at room temperature for 2h. The reaction was quenched with water and the solution was extracted with DCM (3x 100mL). The organic layer was washed with brine, dried over Na_2SO_4, filtered and filtrate was evaporated to dryness. The residue was purified by flash column chromatography (Teledyne Isco RediSep Rf 80 g; gradient 2% to 40% EtOAc – hexane + 5% constant DCM additive) to give 1.6 g of yellow glassy solid material in a 60% yield.

^{1}H NMR (400 MHz, CD_2Cl_2) δ 6.93 (d, *J* = 2.6 Hz, 1H), 6.81 (d, *J* = 8.6 Hz, 1H), 6.78 (s, 1H), 6.68 (s, 1H), 6.58 (dd, *J* = 8.6, 2.7 Hz, 1H), 5.31 (q, *J* = 1.5 Hz, 1H), 3.96 (s, 2H), 2.90 (s, 6H), 2.76 (s, 3H), 1.85 (d, *J* = 1.4 Hz, 3H), 1.29 (s, 6H). ^{13}C NMR (101 MHz, CD_2Cl_2) δ 150.7, 145.5, 131.2, 130.9, 127.9, 127.5, 126.7, 126.0, 125.8, 125.1, 123.3, 116.6, 114.6, 112.3, 57.0, 40.8, 40.4, 31.2, 27.6, 18.7. ESI-MS, positive mode: m/z = 479.1 [M+H]+. HRMS (ESI) calcd for $C_{22}H_{27}N_2Br_2$ $[M+H]^+$ 477.0536, found 477.0527.

(6-bromo-1-methylindolin-5-yl)methanol (**SI-22**):

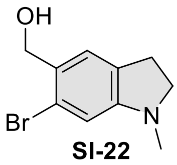

SI-22

Phosphorus oxychloride (0.88 mL, 9.4 mmol) was added slowly into anhydrous DMF (5 mL) at 0 °C under argon and stired for 30 minutes. Then solution of 1-methyl-6-bromoindoline (1 g, 4.72 mmol) in DMF (5 mL) was added to the mixture and stirring was continued overnight at 80°C. Then mixture was cooled and quenched with $NaHCO_3$ solution (30 mL), extracted with EtOAc (3x50 mL), organic layer was separated and dried over Na_2SO_4. The solvent was fully evaporated and obtained aldehyde was redissolved in MeOH (40 mL). The obtained solution was cooled in an ice bathe and solid $NaBH_4$ (178 mg, 4.72 mmol) was added in 3 portions. Ice bath was removed and stirring was continued for 1 hour. Completion of the reaction was confirmed by TLC 8:2 hexane:EtOAc. After reaction was complete methanol was evaporated on rotary evaporator and water (20 mL) was added to the residue. The obtained mixture was extracted with EtOAc (3x50 mL), organic layers were separated, dried over Na_2SO_4. Solvent was evaporated on rotary evaporator to obtain 1.0 g (88%) of crude **SI-22**. Synthesis of (6-bromo-1-methylindolin-5-yl)methanol (**SI-22**)was performed based on previously published procedure[17].

8-bromo-9-((6-bromo-1-methylindolin-5-yl)methyl)-2,3,6,7-tetrahydro-1H,5H-pyrido[3,2,1-ij]quinolone (SI-23):

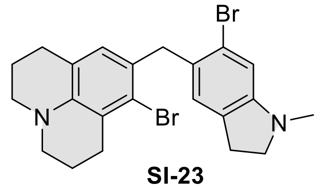

SI-23

Compound **SI-22** (1.0 g, 4.0 mmol), compound **SI-12** (0.97 g, 4.0 mmol) and $BF_3{\cdot}OEt_2$ (1.0 mL, 8 mmol) were dissolved in DCM (50 mL), and the mixture was stirred at room temperature for 2h. The reaction was quenched with water and the solution was extracted with DCM (3x 50mL). The organic layer was washed with brine, dried over Na_2SO_4, filtered and filtrate was evaporated to dryness. The residue was purified by flash column chromatography (Teledyne Isco RediSep Rf 80 g; gradient 5% to 40% EtOAc – hexane) to give 1.37 g of white solid in a 72% yield.

^1H NMR (400 MHz, $CDCl_3$) δ 6.71 (s, 1H), 6.66 (s, 1H), 6.48 (s, 1H), 3.97 (s, 2H), 3.29 (t, J = 8.1 Hz, 2H), 3.13 – 3.06 (m, 4H), 2.87 – 2.80 (m, 4H), 2.74 (s, 3H), 2.65 (t, J = 6.5 Hz, 2H), 2.04 – 1.91 (m, 4H). ^{13}C NMR (101 MHz, $CDCl_3$) δ 153.1, 143.1, 130.2, 128.7, 128.5, 127.2, 126.2, 125.7, 123.2, 121.4, 120.9, 111.0, 56.5, 50.2, 49.7, 41.4, 36.2, 29.5, 28.5, 27.6, 22.4, 22.1. ESI-MS, positive mode: m/z = 477.0 $[M+H]^+$. HRMS (ESI) calcd for C22H25Br2N2 $[M+H]^+$ 477.0360, found 477.0357.

9-oxo-9H-xanthene-3,6-diyl bis(trifluoromethanesulfonate) (SI-24):

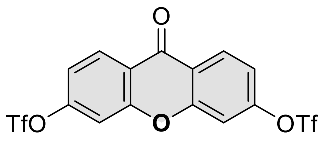

SI-24

Dry pyridine (8.9 mL, 110 mmol) was added to a stirred suspension of 3,6-dihydroxy-9H-xanthen-9- one (2.5 g, 11 mmol) in dry dichloromethane (50 mL) at 0 °C. The reaction mixture was stirred for another 15 min at this temperature, and Tf_2O (5.55 mL, 33.0 mmol) was added dropwise during 20 min. The mixture was allowed to warm to room temperature and stirred for additional 2h. After no starting material was observed (TLC), water (30 mL) was added, and the organic layer was separated, washed with aq HCl (1 M, 3 × 30 mL), brine (30 mL) and dried over MgSO4. The solvent was removed under reduced pressure and dried under vacuum to give 5.3g (99%) of pure product as white solid. This compound has been obtained previuosly[19].

^1H NMR (400 MHz, $CDCl_3$) δ 8.44 (dd, J = 8.9, 0.4 Hz, 2H), 7.49 (d, J = 2.3 Hz, 2H), 7.35 (dd, J = 8.8, 2.3 Hz, 2H).^{13}C NMR (101 MHz, $CDCl_3$) δ 174.7, 156.7, 153.5, 129.8, 121.5, 118.8 (q, $^1J_{C\text{-}F}$ = 318 Hz, $-CF_3$), 118.3, 111.6. ESI-MS, positive mode: m/z = 514.9 $[M+Na]^+$. HRMS (ESI) calcd for $C_{15}H_6F_6O_8S_2Na$ $[M+H]^+$ 514.9300, found 514.9294.

9,9-dimethyl-10-oxo-9,10-dihydroanthracene-2,7-diyl bis(trifluoromethanesulfonate) (**SI-25**):

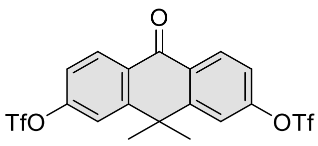

SI-25

Dry pyridine (4.8 mL, 59 mmol) was added to a stirred suspension of 3,6-dihydroxy-10,10-dimethyl-9(10H)-anthracenone[20] (1.5 g, 5.9 mmol) in dry dichloromethane (50 mL) at 0°C. The reaction mixture was stirred for another 15 min at this temperature, and Tf_2O (3.0 mL, 17.7 mmol) was added dropwise during 20 min. The mixture was allowed to warm to room temperature and stirred for additional 2h. After no starting material was observed (TLC), water (30 mL) was added, and the organic layer was separated, washed with aq HCl (1 M, 3 × 30 mL), brine (30 mL) and dried over MgSO4. The solvent was removed under reduced pressure and dried under vacuum to give 3.0g (99%) of pure product as white solid. This compound has been obtained previously[21].

^{1}H NMR (400 MHz, $CDCl_3$) δ 8.46 (d, *J* = 8.8 Hz, 2H), 7.58 (d, *J* = 2.4 Hz, 2H), 7.38 (dd, *J* = 8.8, 2.4 Hz, 2H), 1.78 (s, 6H). ^{13}C NMR (101 MHz, $CDCl_3$) δ 180.9, 153.3, 152.6, 130.9, 129.4, 120.5, 120.0, 118.9 (q, $^1J_{C-F}$ = 319 Hz, -CF_3), 38.7, 33.0. ESI-MS, positive mode: m/z = 519.0 $[M+H]^+$. HRMS (ESI) calcd for $C_{18}H_{12}F_6O_7S_2$ $[M+H]^+$ 519.0001, found 518.9996.

6-(dimethylamino)-9-oxo-9H-xanthen-3-yl trifluoromethanesulfonate (**SI-26**):

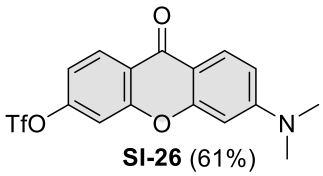

SI-26 (61%)

N,N-Dimethylamine (1.83 mL, 3.66 mmol, 2M in THF) and compound **SI-24** (0.6g, 1.22 mmol) were dissolved in dry DMSO (6 mL). Mixture was stirred and heated in a sealed vial at 90°C overnight. Reaction mixture was cooled, poured into water and extracted with EtOAc (4x 50 mL), washed with brine, dried over Na_2SO_4 and filtered. Filtrate was concentrated on rotary evaporator, the residue was deposited on celite and products were purified by flash column chromatography (Büchi Reveleris HP silica 40 g, gradient 5% to 50% DCM – EtOAc) to give 288 mg of target compound in 61% yield as off-white solid.

^{1}H NMR (400 MHz, $CDCl_3$) δ 8.38 (d, *J* = 8.8 Hz, 1H), 8.13 (d, *J* = 9.1 Hz, 1H), 7.34 (d, *J* = 2.3 Hz, 1H), 7.22 (dd, *J* = 8.8, 2.4 Hz, 1H), 6.74 (dd, *J* = 9.1, 2.4 Hz, 1H), 6.48 (d, *J* = 2.5 Hz, 1H), 3.13 (s, 6H). ^{13}C NMR (101 MHz, $CDCl_3$) δ 174.3, 158.5, 156.6, 155.3, 152.3, 129.2, 128.2, 122.3, 118.8 (q, $^1J_{C-F}$ = 319 Hz, -CF_3), 116.6, 111.5, 110.8, 110.3, 96.8, 40.3. ESI-MS, positive mode: m/z = 388.0 $[M+H]^+$. HRMS (ESI) calcd for $C_{16}H_{13}NO_5F_3S$ $[M+H]^+$ 388.0461, found 388.0464.

3-(dimethylamino)-6-hydroxy-9H-xanthen-9-one (**SI-27**):

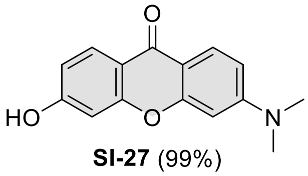

SI-27 (99%)

To a solution of **SI-26** (288 mg, 0.74 mmol) in *i*-PrOH (10 mL) solution of NaOH (1 mL, 1M) was added and the obtained mixture was stirred at 60°C for 1h. Reaction mixture was cooled, neutralized with HCl (1 mL, 1M), diluted with water and cooled in an ice bath. The formed precipitate was filtered and dried to give 187 mg of pure compound as light yellow solid in a quantitative yield.

^{1}H NMR (400 MHz, d_6-DMSO) δ 10.7 (s, 1H), 7.9 (dd, *J* = 21.0, 8.8 Hz, 2H), 6.9 – 6.7 (m, 3H), 6.6 (d, *J* = 1.9 Hz, 1H), 3.1 (s, 6H). ^{13}C NMR (101 MHz, d_6-DMSO) δ 173.5, 162.9, 157.6, 157.3, 154.5, 127.5, 126.9, 114.3, 113.1, 110.4, 109.6, 101.9, 96.6, 39.7. ESI-MS, positive mode: m/z = 256.3 $[M+H]^+$. HRMS (ESI) calcd for $C_{15}H_{13}NO_3Na$ $[M+Na]^+$ 279.0865, found 279.0861.

3,7-bis(dimethylamino)-5,5-dimethyldibenzo[b,e]silin-10(5H)-one (**K2**):

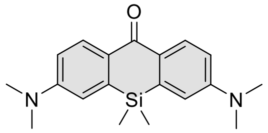

3-Bromodimethylaniline was dissolved in acetic acid (40 mL) and 37% formaldehyde solution (10 mL) was added to the mixture. Then mixture was heated to 65°C on an oil bath. After 1h reaction mixture was cooled to room temperature. After cooling acetic acid was evaporated and $NaHCO_3$ solution (50 mL) was added. The mixture was extracted with EtOAc (3x 100 mL), organic layers were separated, washed with brine, dried over Na_2SO_4. The intermediate compound was purified by flash column chromatography (Teledyne Isco RediSep Rf 80 g; gradient 5% to 80% EtOAc – hexane) to give 6.4 g (61%) of white solid, which was further dissolved in anhydrous THF (250 mL) flushed with argon and cooled to -78°C in dry ice/acetone bath. Then 1,4M solution of *s*-BuLi (27.7 mL, 38.75 mmol, 2.5 eq) was slowly added to the reaction mixture. After addition was complete mixture was stirred at -78°C for 1.5h and then $SiMe_2Cl_2$ (2.8 mL, 0.02325 mmol, 1.5 eq) was added. After addition was complete cooling bath was removed and mixture was left to warm to room temperature and stirring was continued for additional 2h. Then reaction was quenched with 1M HCl (5mL), THF was removed on rotary evaporator and solution of $NaHCO_3$ (50 mL) was added to the residue and mixture was extracted with EtOAc (4x 100mL), organic layers were separated, washed with brine, dried over Na_2SO_4 and solvent was removed under vacuum. The obtained residue was dissolved in acetone (100 mL) and cooled to -15°C. Then powdered $KMnO_4$ (4.9 g, 0.031, 2eq) were added in small portions over the period of 3 hours, after addition was complete stirring was continued for 2 more hours. Then the reaction was quenched by pouring it to cold (-78°C) DCM (200 mL). Celite was added to the mixture, stirred and filtered through another thin layer of celite and washed with DCM. Filtrate was concentrated on a rotary evaporator, the mixture was deposited on celite and product was purified by flash column chromatography (Büchi Reveleris HP silica 80 g, gradient 10% to 60% DCM – EtOAc) to yield 2.21 g (44%)

of yellow crystals. The synthesis of 3,7-bis(dimethylamino)-5,5-dimethyldibenzo[b,e]silin-10(5H)-one (**K2**)was performed based on previously published procedure[13].

^{1}H NMR (400 MHz, $CDCl_3$) δ 8.48 (d, 2 H, J = 8.9 Hz), 6.81 (dd, 2 H, J = 9.0 Hz, 2.4 Hz), 6.78 (d, 2 H, J =2.4 Hz), 3.18 (s, 12 H), 0.32 (s, 6 H).^{13}C NMR (125MHz, $CDCl_3$) δ 185.2, 151.5, 140.2, 131.3, 129.2, 114.2, 113.4, 40.1, -0.3. ESI-MS, positive mode: m/z = 325.2 $[M+H]^+$

3,6-bis(dimethylamino)-9H-xanthen-9-one (**K3**):

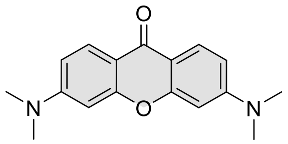

The Pyronin Y (4.0g, 13.2 mol) was dissolved in NMP (50 mL) transferred to Ace pressure tube and sodium phosphate tribasic dodecahydrate (10 g, 26.4 mmol), and water (3 mL) was added. The vial was sealed and heated at 115°C for 2 hours. Mixture was cooled and another portion of sodium phosphate tribasic dodecahydrate (5 g, 13.2 mmol), I_2 (3.35 g, 13.2 mmol) and water (2 mL) was added and then mixture was left at 115°C overnight. After cooling to rt reaction mixture was filtered through celite, celite was washed with DCM:MeOH (8:2). The filtrate was concentrated on rotavapor, NMP was removed by vacuum distillation. The obtained residue was dissolved in DCM:MeOH and celite was added followed by removal of the solvent. The product was purified by flash column chromatography (Büchi Reveleris HP silica 120g; gradient 10% to 80% hexane – EtOAc with a constant 10% DCM additive), fractions containing the product were evaporated to give 2.15 g (58%) of light brown solid. The synthesis of 3,6-bis(dimethylamino)-9H-xanthen-9-one (**K3**) was performed based on previously published procedure[22].

^{1}H NMR (400 MHz, $CDCl_3$) δ 8.01 (d, *J* = 9.0 Hz, 2H), 6.58 (d, *J* = 9.0 Hz, 2H), 6.38 (s, 2H), 3.12 (s, 12H). ^{13}C NMR (125 MHz, $CDCl_3$) δ 175.3, 158.4, 154.2, 127.8, 111.4, 109.5, 96.2, 40.2. ESI-MS, positive mode: m/z = 283.2 $[M+H]^+$.

3,6-bis(dimethylamino)-10,10-dimethylanthracen-9(10H)-one (**K4**):

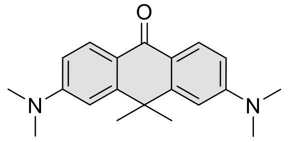

Solution consisting of **SI-9** (2.0g, 0.0124 mol, 1 eq) and 4-(dimethylamino)benzenemethanol (1.87 g, 0.0124 mol, 1 eq) in dry DCM was cooled to -78°C in dry ice/acetone cooling bath. Then 1 M BCl_3 (14.9 mL, 0.0149 mmol, 1.2 eq) was injected slowly and the cooling bath was removed allowing the reaction mixture to warm to room temperature. After stirring overnight at room temperature reaction was complete (TLC control, hexane : EtOAc 8:2) and the solvent was evaporated. To the crude residue ~25 g of polyphosphoric acid (prepared by phosphoric acid + P_2O_5 method) was added and the reaction mixture was heated to 110°C. Heating was continued for 3h and then mixture was poured on ice and neutralized with concentrated NaOH solution. The mixture was extracted with DCM, washed with brine and dried over

Na_2SO_4. The organic solvent was evaporated and the residue was dissolved in acetone (50 mL) and cooled to -15°C. Then powdered $KMnO_4$ (3.92 g, 0.0248 mmol, 2 eq) were added in small portions over the period of 3 hours, after addition was complete stirring was continued for 2 more hours. Then the reaction was quenched by pouring it to cold (-78°C) DCM (50 mL). Celite was added to the mixture, stirred and filtered through another thin layer of celite and washed with DCM. Filtrate was concentrated on a rotary evaporator, the mixture was deposited on celite and product was purified by flash column chromatography (Büchi Reveleris HP silica 80 g, gradient 10% to 60% DCM – EtOAc) to yield 1.45 g (38%) of yellow solid. The synthesis of 3,6-bis(dimethylamino)-10,10-dimethylanthracen-9(10H)-one (**K4**) was performed based on previously published procedure[13].

^{1}H NMR (400 MHz, CDCl3): δ 8.24 (d, *J* = 8.6 Hz, 2H), 6.71-6.88 (m, 4H), 3.16 (s, 12H) 1.78 (s, 6H). ^{13}C NMR (125 MHz, $CDCl_3$) δ 181.1, 153.2, 128.9, 119.8, 111.3, 107.6, 40.1, 37.9, 34.2. ESI-MS, positive mode: m/z = 309.3 $[M+H]^+$

Compound **K5**:

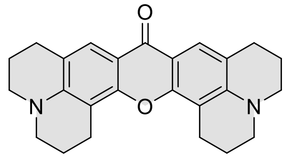

K5

Compound **SI-1** (0.7 g, 1.8 mmol) was added to concentrated H_2SO_4 (10 mL) and was heated to 90°C for 3 hours. The reaction was cooled and poured onto ice and neutralized with NaOH solution while maintaining temperature with addition of ice, the pH was adjusted to 8-9. The mixture was extracted with DCM (4x 50 mL), the combined organic extracts were dried over Na_2SO_4, filtered and solvent was removed under reduced pressure. The residue was dissolved in NMP (20 mL) transferred to Ace pressure tube and Cs_2CO_3 (1.2 g, 3.6 mmol), I_2 (0.46 g, 1.8 mmol) and water (1 mL) was added. The vial was sealed and heated at 115°C for 2 hours, during the time mixture color changed from brown to purple and back to brown. Mixture was cooled and another portion of Cs_2CO_3 (0.59 g, 1.8 mmol) and I_2 (0.46 g, 1.8 mmol) was added, same was repeated 2h afterwards and then mixture was left at 115°C overnight. After cooling to rt reaction mixture was filtered through celite, celite was washed with DCM:MeOH (8:2). The filtrate was concentrated on rotavapor, NMP was removed by vacuum distillation. The obtained residue was dissolved in DCM:MeOH and celite was added followed by removal of the solvent. The product was purified by flash column chromatography (Büchi Reveleris HP silica 40g; gradient 5% to 40% DCM – EtOAc), fractions containing the product were evaporated to give 0.35g (50% in 2 steps) of brown solid. This compound has been obtained previuosly[23].

^{1}H NMR (400 MHz, $CDCl_3$) δ 7.74 (s, 2H), 3.26 (s, 8H), 2.90 (t, *J* = 6.0 Hz, 4H), 2.81 (t, *J* = 6.0 Hz, 4H), 2.09 – 1.91 (m, 8H). ^{13}C NMR (101 MHz, $CDCl_3$) δ 174.8, 153.4, 147.3, 123.5, 118.4, 110.6, 105.6, 50.3, 49.7,

27.6, 21.8, 21.0, 20.7. ESI-MS, positive mode: m/z = 409.2 $[M+Na]^+$. HRMS (ESI) calcd for $C_{25}H_{26}N_2O_2Na$ $[M+Na]^+$ 409.1886, found 409.1879.

Compound **K6:**

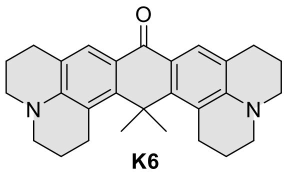

K6

Solution consisting of **SI-11** (500 mg, 2.5 mmol, 1 eq) and **SI-13** (530 mg, 2.5 mmol, 1 eq) in dry DCM was cooled to -78°C in dry ice/acetone cooling bath. Then BF_3-OEt_2 (1.17 mL, 9.3 mmol) was injected at once and the cooling bath was removed allowing the reaction mixture to warm to room temperature. After stirring for 1h at room temperature reaction was complete (TLC control, hexane : EtOAc 8:2) and the solvent was evaporated. To the crude residue ~40g of polyphosphoric acid (prepared by phosphoric acid + P_2O_5 method) was added and the reaction mixture was heated to 145°C. Heating was continued for 3-4h till the reaction was complete and then mixture was poured on ice and neutralized with concentrated NaOH solution. The mixture was extracted with DCM, washed with brine and dried over Na_2SO_4. The organic solvent was evaporated and the residue was dissolved in acetone (50 mL). The solution was cooled to -40°C (controlled dry ice/acetone bath) and $KMnO_4$ (395 mg, 2.5 mmol) was added all at once. The temperature of the cooling bath was allowed to slowly rise to -15°C in the course of 1h. TLC (8:2:1 - hexane:EtOAc:DCM) showed incomplete reaction. Reaction mixture was cooled to -40°C and another portion of $KMnO_4$ (100 mg, 0.65 mmol) was introduced. Again, temperature of the cooling bath was allowed to slowly rise to -15°C in the course of 1h. Once reaction was complete the reaction was quenched by pouring to cold (-78°C) DCM (100 mL). Celite was added to the mixture, stirred and filtered through another thin layer of celite and washed with DCM. Filtrate was concentrated on a rotary evaporator, the mixture was deposited on celite and product was purified by flash column chromatography (Büchi Reveleris HP silica 40 g, gradient 10% to 60% DCM – EtOAc; Büchi Reveleris HP silica 24g, gradient 20% to 100% Hexane – EtOAc with a constant 5% DCM additive) and lyophilized from MeCN/H_2O mixture to give 154 mg (15% in 3 steps) of yellow solid.

^{1}H NMR (400 MHz, CD_2Cl_2) δ 7.76 (s, 2H), 3.29 (q, *J* = 6.1, 5.7 Hz, 8H), 2.91 (t, *J* = 5.8 Hz, 4H), 2.78 (t, *J* = 6.2 Hz, 4H), 1.95 (q, *J* = 5.2 Hz, 8H), 1.91 (s, 6H). ^{13}C NMR (101 MHz, CD_2Cl_2) δ 181.8, 152.2, 148.6, 126.0, 121.2, 118.4, 118.0, 51.3, 50.7, 37.2, 32.2, 28.7, 28.6, 22.4, 22.4. ESI-MS, positive mode: m/z = 413.3 $[M+H]^+$. HRMS (ESI) calcd for $C_{28}H_{33}N_2O$ $[M+H]^+$ 413.2587, found 413.2586.

1,9,11,11-tetramethyl-2,3,7,8,9,11-hexahydrobenzo[1,2-f:5,4-f']diindol-5(1H)-one (**K7**):

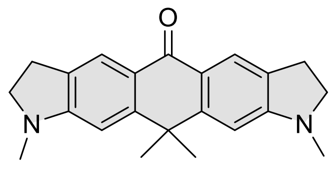

K7

Solution consisting of **SI-4** (500 mg, 2.89 mmol, 1eq) and **SI-7** (470 mg, 2.89 mmol, 1 eq) in dry DCM was cooled to -78°C in dry ice/acetone cooling bath. Then BF_3-OEt_2 (544 µL, 4.33 mmol, 1.5 eq) was injected at once and the cooling bath was removed allowing the reaction mixture to warm to room temperature. After stirring for 1h at room temperature reaction was complete (TLC control, hexane : EtoAc 8:2) and the solvent was evaporated. To the crude residue ~25g of polyphosphoric acid (prepared by phosphoric acid + P_2O_5 method) was added and the reaction mixture was heated to 110°C. Heating was continued for 1.5h and then mixture was poured on ice and neutralized with concentrated NaOH solution. The mixture was extracted with DCM, washed with brine and dried over Na_2SO_4. The organic solvent was evaporated and the residue was dissolved in acetone (50 mL). The solution was cooled to -15°C (*i*-PrOH:water 1:1, dry ice) and $KMnO_4$ (460 mg, 2.91 mmol) was added in small portions. After 1h, TLC (8:2 - hexane:EtOAc) showed incomplete reaction and another portion of $KMnO_4$ (460 mg, 2.91 mmol) was introduced in small portions. Once reaction was complete the reaction was quenched by pouring to cold (-78°C) DCM (100 mL). Celite was added to the mixture, stirred and filtered through another thin layer of celite, washed with DCM. Filtrate was concentrated on rotary evaporator, the mixture was deposited on celite and product was purified by flash column chromatography (Büchi Reveleris HP silica 40g; gradient 2% to 40% DCM – EtOAc) to give 288 mg (30% in 3 steps) of yellow solid.

^{1}H NMR (400 MHz, $CDCl_3$) δ 8.06 (t, *J* = 1.3 Hz, 2H), 6.47 (s, 2H), 3.49 (t, *J* = 8.3 Hz, 4H), 3.03 (td, *J* = 8.3, 1.3 Hz, 4H), 2.90 (s, 6H), 1.68 (s, 6H). ^{13}C NMR (101 MHz, $CDCl_3$) δ 181.1, 156.4, 152.6, 129.4, 123.2, 121.2, 102.0, 55.3, 38.7, 34.7, 33.5, 27.7. ESI-MS, positive mode: m/z = 355.2 $[M+Na]^+$. HRMS (ESI) calcd for $C_{22}H_{24}N_2ONa$ $[M+Na]^+$ 355.1781, found 355.1781.

1,9,11,11-tetramethyl-2,3,7,8,9,11-hexahydrosilino[3,2-f:5,6-f']diindol-5(1H)-one (**K8**):

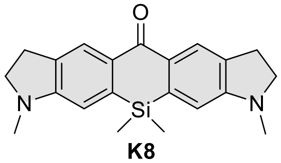

K8

In a 250 mL round-bottom flask, a degassed solution of **SI-17** (1.7 g, 3.9 mmol) in anhydrous THF (50 mL) was cooled to -78°C (dry ice – acetone cooling bath). *s*-Butyllithium (7.0 mL of 1.4M in cyclohexane, 9.75 mmol) was introduced through a needle to the reaction mixture and stirring was continued at -78°C for 1.5h. Then Cl_2SiMe_2 (470 µL, 3.9 mmol, 2 eq) was slowly injected to the reaction mixture and stirred for 10 min at -78°C before the cooling bath was removed and mixture was allowed to slowly warm to room temperature. Stirring was continued for 1h at rt then it was quenched with 1 mL of saturated NH_4Cl solution. THF was partially evaporated on rotary evaporator. Water was added to the residue and

mixture was extracted with DCM (4x 50 mL), washed with brine, dried over Na_2SO_4 and filtered. Filtrate was concentrated on rotary evaporator and obtained crude compound was dissolved in 50 mL of acetone. Obtained solution was cooled to -15°C (*i*-PrOH:H_2O 1:1 dry ice cooling bath) and $KMnO_4$ (1.2g ,7.8 mmol) was added in small portions in the course of 3-4h. Reaction course was monitored by TLC (9:1 DCM:EtOAc). Once reaction was complete it was quenched by pouring reaction mixture to -78°C cooled DCM (100 mL). Celite was added to the mixture, stirred and filtered through another thin layer of celite, washed with DCM. Filtrate was concentrated on rotary evaporator, the mixture was deposited on celite and product was purified by flash column chromatography (Büchi Reveleris HP silica 40 g, gradient 2% to 20% DCM – EtOAc) to give 300 mg (22% in 2 steps) of yellow solid. This compound has been obtained previuosly[18].

^{1}H NMR (400 MHz, $CDCl_3$) δ 8.21 (s, 2H), 6.51 (s, 2H), 3.47 (t, *J* = 8.4 Hz, 4H), 3.05 (td, *J* = 8.7, 1.0 Hz, 4H), 2.90 (s, 6H), 0.45 (s, 6H). ^{13}C NMR (101 MHz, $CDCl_3$) δ 185.2, 154.9, 140.1, 132.3, 131.7, 126.2, 108.0, 55.0, 34.7, 28.2, -0.9. ESI-MS, positive mode: m/z = 349.2 $[M+H]^+$. HRMS (ESI) calcd for $C_{21}H_{25}N_2OSi$ $[M+H]^+$ 349.1731, found 349.1733.

1**,2,2,4,8,10,10,11,13,13-decamethyl-2,10,11,13-tetrahydrosilino[3,2-g:5,6-g']diquinolin-6(1H)-one** (**K9**):

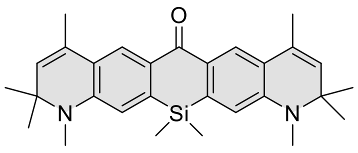

K9

In a 250 mL round-bottom flask, a degassed solution of **SI-18** (2 g, 3.7 mmol) in anhydrous THF (50 mL) was cooled to -78°C (dry ice – acetone cooling bath). *s*-Butyllithium (6.6 mL of 1.4M in cyclohexane, 9.25 mmol) was introduced through a needle to the reaction mixture and stirring was continued at -78°C for 1.5h. Then Cl_2SiMe_2 (445 µL, 3.7 mmol, 2 eq) was slowly injected to the reaction mixture and stirred for 10 min at -78°C before the cooling bath was removed and mixture was allowed to slowly warm to room temperature. Stirring was continued for 1h at rt then it was quenched with 1 mL of saturated NH_4Cl solution. THF was partially evaporated on rotary evaporator. Water was added to the residue and mixture was extracted with DCM (4x 50 mL), washed with brine, dried over Na_2SO_4 and filtered. Filtrate was concentrated on rotary evaporator and obtained crude compound was dissolved in 50 mL of acetone. Obtained solution was cooled to -20°C (Ethylene glycol+10% EtOH dry ice cooling bath) and $KMnO_4$ (1.75 g, 11.1 mmol) was added in small portions in the course of 3-4h. Reaction course was monitored by TLC (8:2 hexane:EtOAc). Once reaction was complete it was quenched by pouring reaction mixture to -78°C cooled DCM (100 mL). Celite was added to the mixture, stirred and filtered through another thin layer of celite, washed with DCM. Filtrate was concentrated on rotary evaporator, the mixture was deposited on celite and product was purified by flash column chromatography (Büchi Reveleris HP silica 40 g, gradient 5% to 70% hexane– EtOAc) to give 590 mg (35% in 2 steps) of yellow solid.

^{1}H NMR (400 MHz, $CDCl_3$) δ 8.19 (s, 2H), 6.57 (s, 2H), 5.32 (q, *J* = 1.4 Hz, 2H), 2.94 (s, 6H), 2.08 (d, *J* = 1.4 Hz, 6H), 1.38 (s, 12H), 0.46 (s, 6H). ^{13}C NMR (101 MHz, $CDCl_3$) δ 185.3, 146.9, 140.8, 129.9, 129.9, 128.2, 124.9, 123.4, 112.5, 57.3, 31.2, 28.7, 18.8, -0.9. ESI-MS, positive mode: m/z = 457.3 $[M+H]^+$. HRMS (ESI) calcd for $C_{29}H_{36}N_2OSi$ $[M+H]^+$ 457.2670, found 457.2671.

8-(dimethylamino)-1,10,10-trimethyl-1,2,3,10-tetrahydro-5H-naphtho[2,3-f]indol-5-one (**K10**):

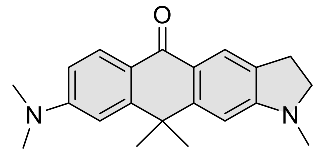

K10

Solution consisting of **SI-9** (1 g, 6.2 mmol, 1eq) and **SI-7** (1.02 g, 6.2 mmol, 1 eq) in dry DCM was cooled to -78°C in dry ice/acetone cooling bath. Then BF_3-OEt_2 (1.17 mL, 9.3 mmol) was injected at once and the cooling bath was removed allowing the reaction mixture to warm to room temperature. After stirring for 1h at room temperature reaction was complete (TLC control, hexane : EtOAc 8:2) and the solvent was evaporated. To the crude residue ~40g of polyphosphoric acid (prepared by phosphoric acid + P_2O_5 method) was added and the reaction mixture was heated to 110°C. Heating was continued for 2h and then mixture was poured on ice and neutralized with concentrated NaOH solution. The mixture was extracted with DCM, washed with brine and dried over Na_2SO_4. The organic solvent was evaporated and the residue was dissolved in acetone (100 mL). The solution was cooled to -15°C (*i*-PrOH:water 1:1, dry ice) and $KMnO_4$ (980 mg, 6.2 mmol) was added in small portions. After 1h, TLC (8:2 - hexane:EtOAc) showed incomplete reaction and another portion of $KMnO_4$ (490 mg, 3.1 mmol) was introduced in small portions. Once reaction was complete the reaction was quenched by pouring to cold (-78°C) DCM (100 mL). Celite was added to the mixture, stirred and filtered through another thin layer of celite, washed with DCM. Filtrate was concentrated on rotary evaporator, the mixture was deposited on celite and product was purified by flash column chromatography (Büchi Reveleris HP silica 40g, gradient 2% to 50% DCM – EtOAc; Büchi Reveleris HP silica 24g, gradient 20% to 100% Hexane – EtOAc) to give 635 mg (32% in 3 steps) of yellow solid.

^{1}H NMR (400 MHz, CD_2Cl_2) δ 8.14 (d, *J* = 8.7 Hz, 1H), 7.96 (s, 1H), 6.79 – 6.73 (m, 2H), 6.51 (s, 1H), 3.50 (t, *J* = 8.3 Hz, 2H), 3.09 (s, 6H), 3.03 (t, *J* = 8.3 Hz, 2H), 2.91 (s, 3H), 1.69 (s, 6H).^{13}C NMR (101 MHz, CD_2Cl_2) δ 181.0, 157.1, 153.6, 153.1, 152.7, 130.0, 129.1, 123.1, 121.4, 120.3, 111.3, 108.4, 102.5, 55.7, 40.6, 38.9, 34.9, 33.9, 28.0.ESI-MS, positive mode: m/z = 321.2 $[M+H]^+$. HRMS (ESI) calcd for $C_{21}H_{25}N_2O$ $[M+H]^+$ 321.1961, found 321.1961.

12-(dimethylamino)-14,14-dimethyl-2,3,5,6,7,14-hexahydro-1H,9H-naphtho[2,3-f]pyrido[3,2,1-ij]quinolin-9-one (**K11**):

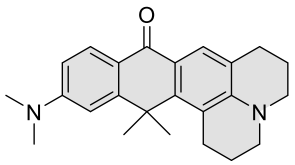

K11

Solution consisting of **SI-9** (395 mg, 2.46 mmol, 1 eq) and **SI-11** (500 mg, 2.46 mmol, 1 eq) in dry DCM was cooled to -78°C in dry ice/acetone cooling bath. Then BF_3-OEt_2 (465 µL, 3.69 mmol) was injected at once and the cooling bath was removed allowing the reaction mixture to warm to room temperature. After stirring for 1h at room temperature reaction was complete (TLC control, hexane : EtOAc 8:2) and the solvent was evaporated. To the crude residue ~25 g of polyphosphoric acid (prepared by phosphoric acid + P_2O_5 method) was added and the reaction mixture was heated to 140°C. Heating was continued for 2h and then mixture was poured on ice and neutralized with concentrated NaOH solution. The mixture was extracted with DCM, washed with brine and dried over Na_2SO_4. The residue was dissolved in NMP (20 mL) transferred to Ace pressure tube and Na_3PO_4-$6H_20$ (2.3 g, 4.92 mmol), I_2 (625 mg, 2.46 mmol) and water (0.5 mL) was added. The vial vas sealed and heated at 110°C for 2 hours, during the time mixture color changed from brown to blue and back to brown. Mixture was cooled and another portion of Na_3PO_4-$6H_20$ (1.15 g, 2.46 mmol) and I_2 (625 mg, 2.46 mmol) was added and then mixture was left at 115°C overnight. After cooling to rt reaction mixture was filtered through celite, celite was washed with DCM:MeOH (8:2). The filtrate was concentrated on rotary evaporator, NMP was removed by vacuum distillation. The obtained residue was dissolved in DCM:MeOH and celite was added followed by removal of the solvent. The product was purified by flash column chromatography (Büchi Reveleris HP silica 40g; gradient 20% to 80% Hexane – EtOAc), fractions containing the product were evaporated to give 230 mg(26% in 3 steps) of yellow solid.

^{1}H NMR (400 MHz, CD_2Cl_2) δ 8.07 (d, *J* = 8.8 Hz, 1H), 7.87 (s, 1H), 6.74 (dd, *J* = 8.8, 2.5 Hz, 1H), 6.70 (d, *J* = 2.5 Hz, 1H), 3.32 – 3.27 (m, 4H), 3.08 (s, 6H), 3.04 – 3.00 (m, 2H), 2.82 – 2.78 (m, 2H), 1.99 – 1.93 (m, 4H), 1.82 (s, 6H). ^{13}C NMR (101 MHz, CD_2Cl_2) δ 181.4, 156.8, 153.8, 148.5, 146.9, 128.4, 126.9, 121.5, 119.7, 119.4, 118.8, 111.3, 109.0, 51.3, 50.7, 40.6, 38.4, 31.6, 28.7, 28.3, 22.5, 22.3. ESI-MS, positive mode: m/z = 361.2 $[M+H]^+$. HRMS (ESI) calcd for $C_{24}H_{28}N_2O$ $[M+H]^+$ 361.2274, found 361.2268.

9-(dimethylamino)-1,2,2,4,11,11-hexamethyl-2,11-dihydronaphtho[2,3-g]quinolin-6(1H)-one (**K12**):

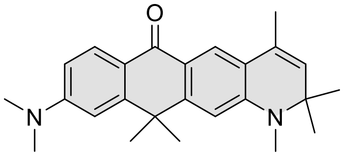

K12

Solution consisting of 4-(dimethylamino)benzyl alcohol (432 mg, 2.86 mmol, 1 eq) and **SI-16** (650 mg, 2.86 mmol, 1 eq) in dry DCM was cooled in an ice bath and BF_3-OEt_2 (540 µL, 4.3 mmol) was injected at once. The cooling bath was removed allowing the reaction mixture to warm to room temperature. After stirring for 3h at room temperature the reaction was complete (TLC control,

hexane : EtOAc 8:2) and the solvent was evaporated. To the crude residue ~40g of polyphosphoric acid (prepared by phosphoric acid + P_2O_5 method) was added and the reaction mixture was heated to 120°C. Heating was continued for 2h till the reaction was complete and then the mixture was poured on ice and neutralized with concentrated NaOH solution. The mixture was extracted with DCM, washed with brine and dried over Na_2SO_4. The organic solvent was evaporated and the residue was dissolved in acetone (50 mL). The solution was cooled to -40°C (controlled dry ice/acetone bath) and $KMnO_4$ (452 mg, 2.86 mmol) was added all at once. The temperature of the cooling bath was allowed to slowly rise to -25°C and was held at this temperature for 2h. Then mixture was allowed to warm to -15 °C and stirred for additional 1h. Reaction course was monitored by TLC (7:2:1 - hexane:EtOAc:DCM). Once reaction was complete it was quenched by pouring mixture to cold (-78°C) DCM (100 mL). Celite was added to the mixture, stirred and filtered through another thin layer of celite, washed with DCM. Filtrate was concentrated on rotary evaporator, the mixture was deposited on celite and product was purified by flash column chromatography (Büchi Reveleris HP silica 40 g, gradient 10% to 70% hexane – EtOAc with a constant 5% DCM additive) to give 225mg (21% in 3 steps) of yellow solid.

^1H NMR (400 MHz, CD_2Cl_2) δ 8.15 (d, *J* = 9.0 Hz, 1H), 7.91 (s, 1H), 6.79 – 6.74 (m, 2H), 6.57 (s, 1H), 5.36 – 5.33 (m, 1H), 3.09 (s, 6H), 2.95 (s, 3H), 2.07 (d, *J* = 1.4 Hz, 3H), 1.70 (s, 6H), 1.38 (s, 6H). ^{13}C NMR (101 MHz, CD_2Cl_2) δ 181.0, 153.7, 153.2, 152.8, 149.1, 130.2, 129.1, 127.9, 122.2, 121.7, 120.4, 120.0, 111.3, 108.5, 106.8, 57.9, 40.6, 38.6, 33.9, 31.6, 28.9, 19.0. ESI-MS, positive mode: m/z = 397.2 $[M+Na]^+$. HRMS (ESI) calcd for $C_{25}H_{30}N_2ONa$ $[M+Na]^+$ 397.2250, found 397.2249.

12-(dimethylamino)-14,14-dimethyl-2,3,5,6,7,14-hexahydro-1H,9H-benzo[5,6]silino[2,3-f]pyrido[3,2,1-ij]quinolin-9-one (**K13**):

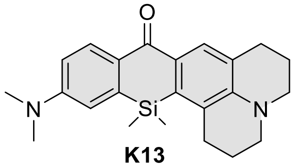

K13

In a 250 mL round-bottom flask, a degassed solution of **SI-19** (1.1 g, 2.37 mmol) in anhydrous THF (50 mL) was cooled to -78°C (dry ice – acetone cooling bath). *s*-Butyllithium (4.2 mL of 1.4M in cyclohexane, 5.92 mmol) was introduced through a needle to the reaction mixture and stirring was continued at -78°C for 1.5h. Then Cl_2SiMe_2 (362 µL, 3.0 mmol,) was slowly injected to the reaction mixture and stirred for 10 min at -78°C before the cooling bath was removed and mixture was allowed to slowly warm to room temperature. Stirring was continued for 1h at rt then it was quenched with 1 mL of saturated NH_4Cl solution. THF was partially evaporated on rotary evaporator. Water was added to the residue and mixture was extracted with DCM (4x 50 mL), washed with brine, dried over Na_2SO_4 and filtered. Filtrate was concentrated on rotary evaporator and obtained crude compound was dissolved in 50 mL of acetone. Obtained solution was cooled to -40°C (controlled dry ice/acetone bath) and $KMnO_4$ (395 mg, 2.5 mmol)

was added all at once. The temperature of the cooling bath was allowed to slowly rise to -15°C in the course of 1h. TLC (8:2:1 - hexane:EtOAc:DCM) showed incomplete reaction. Reaction mixture was cooled to -40°C and another portion of $KMnO_4$ (300 mg, 1.95 mmol) was introduced. Again, temperature of the cooling bath was allowed to slowly rise to -15°C in the course of 1-2h. Once reaction was complete the reaction was quenched by pouring to cold (-78°C) DCM (100 mL). Celite was added to the mixture, stirred and filtered through another thin layer of celite, washed with DCM. Filtrate was concentrated on rotary evaporator, the mixture was deposited on celite and product was purified by flash column chromatography (Büchi Reveleris HP silica 40 g, gradient 5% to 80% hexane – EtOAc +5% constant DCM additive) to give 258 mg (29% in 3 steps) of yellow solid.

^{1}H NMR (400 MHz, CD_2Cl_2) δ 8.24 (dd, *J* = 8.6, 0.7 Hz, 1H), 7.98 (d, *J* = 0.5 Hz, 1H), 6.84 – 6.79 (m, 2H), 3.32 – 3.26 (m, 4H), 3.08 (s, 6H), 2.96 – 2.90 (m, 2H), 2.84 – 2.78 (m, 2H), 2.05 – 1.92 (m, 4H), 0.52 (s, 6H). ^{13}C NMR (101 MHz, CD_2Cl_2) δ 185.4, 152.2, 145.8, 141.9, 135.6, 131.2, 131.2, 130.2, 129.7, 129.3, 125.3, 123.4, 114.7, 113.5, 50.9, 50.4, 40.4, 29.5, 28.8, 22.5, 22.2, 0.1. ESI-MS, positive mode: m/z = 377.2 $[M+H]^+$. HRMS (ESI) calcd for $C_{23}H_{29}N_2OSi$ $[M+H]^+$ 377.2044, found 377.2038.

8-(dimethylamino)-1,10,10-trimethyl-1,2,3,10-tetrahydro-5H-benzo[5,6]silino[3,2-f]indol-5-one (**K14**):

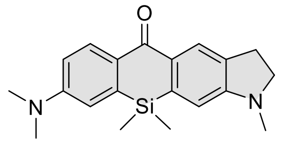

K14

In a 250 mL round-bottom flask, a degassed solution of **SI-19** (1.0 g, 2.36 mmol) in anhydrous THF (50 mL) was cooled to -78°C (dry ice – acetone cooling bath). *s*-Butyllithium (4.2 mL of 1.4M in cyclohexane, 5.92 mmol) was introduced through a needle to the reaction mixture and stirring was continued at -78°C for 1.5h. Then Cl_2SiMe_2 (355 µL, 2.95 mmol,) was slowly injected to the reaction mixture and stirred for 10 min at -78°C before the cooling bath was removed and mixture was allowed to slowly warm to room temperature. Stirring was continued for 1h at rt then it was quenched with 1 mL of saturated NH_4Cl solution. THF was partially evaporated on rotary evaporator. Water was added to the residue and mixture was extracted with DCM (4x 50 mL), washed with brine, dried over Na_2SO_4 and filtered. Filtrate was concentrated on rotary evaporator and obtained crude compound was dissolved in 50 mL of acetone. Obtained solution was cooled to -40°C (controlled dry ice/acetone bath) and $KMnO_4$ (553 mg, 3.5 mmol) was added all at once. The temperature of the cooling bath was allowed to slowly rise to -15°C in the course of 1h. TLC (8:2:1 - hexane:EtOAc:DCM) showed incomplete reaction. Reaction mixture was cooled to -40°C and another portion of $KMnO_4$ (240 mg, 1.52 mmol) was introduced. Again, temperature of the cooling bath was allowed to slowly rise to -15°C in the course of 1-2h. Once reaction was complete the reaction was quenched by pouring to cold (-78°C) DCM (100 mL). Celite was added to the mixture, stirred and filtered through another thin layer of celite, washed with DCM. Filtrate was concentrated on rotary

evaporator, the mixture was deposited on celite and product was purified by flash column chromatography (Büchi Reveleris HP silica 40 g, gradient 5% to 50% hexane – EtOAc +5% constant DCM additive) to give 270 mg (34% in 2 steps) of yellow solid. This compound has been obtained previuosly[17].

^{1}H NMR (400 MHz, CD_2Cl_2) δ 8.28 (dd, *J* = 8.4, 0.9 Hz, 1H), 8.13 (dt, *J* = 1.3, 0.7 Hz, 1H), 6.84 – 6.81 (m, 2H), 6.54 (s, 1H), 3.47 (t, *J* = 8.5 Hz, 2H), 3.08 (s, 6H), 3.04 (td, *J* = 8.4, 1.1 Hz, 2H), 2.90 (s, 3H), 0.45 (s, 6H). ^{13}C NMR (101 MHz, CD_2Cl_2) δ 185.1, 155.5, 152.1, 140.8, 140.6, 132.9, 131.9, 131.7, 130.1, 126.2, 114.8, 113.5, 108.5, 55.3, 40.4, 34.9, 28.6, -0.8. ESI-MS, positive mode: m/z = 337.2 [M+H]+. HRMS (ESI) calcd for $C_{20}H_{25}N_2OSi$ $[M+H]^+$ 337.1731, found 337.1731.

Compound K15:

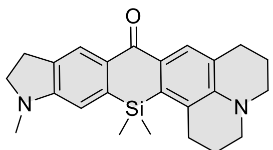

K15

In a 250 mL round-bottom flask, a degassed solution of **SI-23** (1.1 g, 2.3 mmol) in anhydrous THF (50 mL) was cooled to -78°C (dry ice – acetone cooling bath). *s*-Butyllithium (4.1 mL of 1.4M in cyclohexane, 5.75 mmol) was introduced through a needle to the reaction mixture and stirring was continued at -78°C for 1.5h. Then Cl_2SiMe_2 (360 µL, 2.9 mmol,) was slowly injected to the reaction mixture and stirred for 10 min at -78°C before the cooling bath was removed and mixture was allowed to slowly warm to room temperature. Stirring was continued for 1h at rt then it was quenched with 1 mL of saturated NH_4Cl solution. THF was partially evaporated on rotary evaporator. Water was added to the residue and mixture was extracted with DCM (4x 50 mL), washed with brine, dried over Na_2SO_4 and filtered. Filtrate was concentrated on rotary evaporator and obtained crude compound was dissolved in 70 mL of acetone. Obtained solution was cooled to -40°C (controlled dry ice/acetone bath) and $KMnO_4$ (360 mg, 2.3 mmol) was added all at once. The temperature of the cooling bath was allowed to slowly rise to -15°C in the course of 2h. TLC (8:2 - hexane:EtOAc) showed incomplete reaction. Reaction mixture was cooled to -40°C and another portion of $KMnO_4$ (320 mg, 2 mmol) was introduced. Again, temperature of the cooling bath was allowed to slowly rise to -25°C in the course of 1-2h, in the meantime additional amount of $KMnO_4$ (50 mg, 0.32 mmol) was added. Once reaction was complete the reaction was quenched by pouring to cold (-78°C) DCM (100 mL). Celite was added to the mixture, stirred and filtered through another thin layer of celite, washed with DCM. Filtrate was concentrated on rotary evaporator, the mixture was deposited on celite and product was purified by flash column chromatography (Büchi Reveleris HP silica 40 g, gradient 2% to 40% hexane – EtOAc +5% constant DCM additive) to give 335 mg (37% in 2 steps) of yellow solid.

^{1}H NMR (400 MHz, CD_2Cl_2) δ 8.07 (s, 1H), 7.97 (s, 1H), 6.52 (s, 1H), 3.46 (t, *J* = 8.2 Hz, 2H), 3.30 – 3.25 (m, 4H), 3.03 (td, *J* = 8.3, 1.3 Hz, 2H), 2.92 (t, *J* = 6.1 Hz, 2H), 2.89 (s, 3H), 2.81 (t, *J* = 6.3 Hz, 2H), 2.04 – 1.93 (m, 4H), 0.51 (s, 6H). ^{13}C NMR (101 MHz, CD_2Cl_2) δ 185.3, 155.6, 145.7, 141.6, 135.5, 132.8, 131.5,

130.2, 129.3, 125.7, 125.2, 123.3, 108.3, 55.4, 50.9, 50.4, 35.0, 29.4, 28.7, 28.6, 22.5, 22.2, 0.0. ESI-MS, positive mode: m/z = 411.2 $[M+Na]^+$. HRMS (ESI) calcd for $C_{24}H_{28}N_2O_5iNa$ $[M+Na]^+$ 411.1863, found 411.1853.

9-(dimethylamino)-1,2,2,4,11,11-hexamethyl-2,11-dihydrobenzo[5,6]silino[3,2-g]quinolin-6(1H)-one (**K16**):

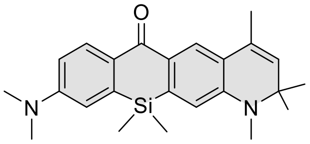

K16

In a 250 mL round-bottom flask, a degassed solution of **SI-21** (1.6 g, 3.35 mmol) in anhydrous THF (50 mL) was cooled to -78°C (dry ice – acetone cooling bath). *s*-Butyllithium (6.0 mL of 1.4M in cyclohexane, 8.37 mmol) was introduced through a needle to the reaction mixture and stirring was continued at -78°C for 1.5h. Then Cl_2SiMe_2 (506 µL, 4.2 mmol,) was slowly injected to the reaction mixture and stirred for 10 min at -78°C before the cooling bath was removed and mixture was allowed to slowly warm to room temperature. Stirring was continued for 1h at rt then it was quenched with 1 mL of saturated NH_4Cl solution. THF was partially evaporated on rotary evaporator. Water was added to the residue and mixture was extracted with DCM (4x 50 mL), washed with brine, dried over Na_2SO_4 and filtered. Filtrate was concentrated on rotary evaporator and obtained crude compound was dissolved in 50 mL of acetone. Obtained solution was cooled to -40°C (controlled dry ice/acetone bath) and $KMnO_4$ (530 mg, 3.35 mmol) was added all at once. The temperature of the cooling bath was allowed to slowly rise to -15°C in the course of 2h. TLC (8:2:1 - hexane:EtOAc:DCM) showed incomplete reaction. Reaction mixture was cooled to -40°C and another portion of $KMnO_4$ (320 mg, 2 mmol) was introduced. Again, temperature of the cooling bath was allowed to slowly rise to -15°C in the course of 1-2h. Once reaction was complete the reaction was quenched by pouring to cold (-78°C) DCM (100 mL). Celite was added to the mixture, stirred and filtered through another thin layer of celite, washed with DCM. Filtrate was concentrated on rotary evaporator, the mixture was deposited on celite and product was purified by flash column chromatography (Büchi Reveleris HP silica 40 g, gradient 5% to 50% hexane – EtOAc +5% constant DCM additive) to give 418 mg (32% in 2 steps) of yellow solid.

^1H NMR (400 MHz, CD_2Cl_2) δ 8.29 (dd, *J* = 8.5, 0.8 Hz, 1H), 8.08 (s, 1H), 6.85 – 6.79 (m, 2H), 6.60 (s, 1H), 5.36 (d, *J* = 1.3 Hz, 1H), 3.08 (s, 6H), 2.94 (s, 3H), 2.07 (d, *J* = 1.4 Hz, 3H), 1.37 (s, 6H), 0.46 (s, 6H). ^{13}C NMR (101 MHz, CD_2Cl_2) δ 185.1, 152.1, 147.5 (2 peaks based on HMBC), 141.3, 140.9, 131.6, 130.7, 130.1, 128.3, 124.9, 123.9, 114.9, 113.5, 113.2, 57.7, 40.4, 31.5, 28.8, 19.0, -0.8. ESI-MS, positive mode: m/z = 391.3 $[M+H]^+$. HRMS (ESI) calcd for C24H31N2OSi $[M+H]^+$ 391.2200, found 391.2196.

3,6-bis(diallylamino)-9H-xanthen-9-one (**K17**):

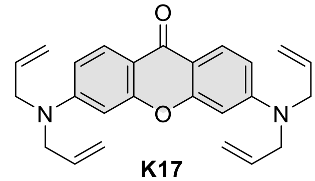

K17

Diallylamine (2.5 mL, 20.3 mmol) and compound **SI-24** (1 g, 2.03 mmol) were dissolved in dry DMSO (6 mL). Mixture was stirred and heated in a sealed vial at 100°C for 24h. Reaction course was monitored by TLC (hexane:EtOAc 3:7). Once reaction was finished mixture was poured to water and extracted with EtOAc (4x 50 mL), washed with brine, dried over Na_2SO_4 and filtered. Filtrate was concentrated on rotary evaporator, the residue was deposited on celite and products were purified by flash column chromatography (Büchi Reveleris HP silica 40 g, gradient 5% to 40% hexane – EtOAc +10% constant DCM additive) to give 400 mg of target compound in 51% yield as yellow solid.

^{1}H NMR (400 MHz, d_6-DMSO) δ 7.84 (d, *J* = 9.0 Hz, 2H), 6.74 (dd, *J* = 9.0, 2.4 Hz, 2H), 6.53 (d, *J* = 2.4 Hz, 2H), 5.97 – 5.80 (m, 4H), 5.23 – 5.10 (m, 8H), 4.06 (d, *J* = 4.7 Hz, 8H). ^{13}C NMR (101 MHz, d_6-DMSO) δ 173.0, 157.4, 152.7, 133.3, 126.8, 116.2, 111.0, 109.4, 97.1, 52.5. ESI-MS, positive mode: m/z = 409.2 $[M+Na]^+$. HRMS (ESI) calcd for $C_{25}H_{26}N_2O_2Na$ $[M+Na]^+$ 409.1886, found 409.1881.

3,6-bis(allyl(methyl)amino)-9H-xanthen-9-one (**K18**):

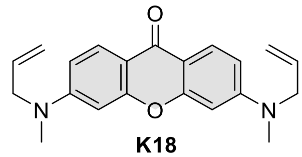

K18

N-Allylmethylamine (1.95 mL, 20.3 mmol) and compound **SI-24** (1 g, 2.03 mmol) were dissolved in dry DMSO (6 mL). Mixture was stirred and heated in a sealed vial at 100°C for 24h. Reaction course was monitored by TLC (hexane:EtOAc 3:7). Once reaction was finished mixture was poured to water and extracted with EtOAc (4x 50 mL), washed with brine, dried over Na_2SO_4 and filtered. Filtrate was concentrated on rotary evaporator, the residue was deposited on celite and products were purified by flash column chromatography (Büchi Reveleris HP silica 40 g, gradient 20% to 80% hexane – EtOAc +5% constant DCM additive) to give 325 mg of target compound in 48% yield as yellow solid.

^{1}H NMR (400 MHz, d_6-DMSO) δ 7.86 (d, *J* = 9.0 Hz, 2H), 6.76 (dd, *J* = 9.0, 2.4 Hz, 2H), 6.53 (d, *J* = 2.4 Hz, 2H), 5.85 (ddt, *J* = 17.1, 10.4, 4.8 Hz, 2H), 5.21 – 5.04 (m, 4H), 4.17 – 4.00 (m, 4H), 3.05 (s, 6H). ^{13}C NMR (101 MHz, d_6-DMSO) δ 173.1, 157.5, 153.4, 132.9, 126.8, 116.1, 110.9, 109.3, 96.8, 53.9, 38.1. ESI-MS, positive mode: m/z = 335.2 $[M+H]^+$. HRMS (ESI) calcd for $C_{21}H_{23}N_2O_2$ $[M+H]^+$ 335.1754, found 335.1754.

3,6-bis(allyl(methyl)amino)-10,10-dimethylanthracen-9(10H)-one (**K19**):

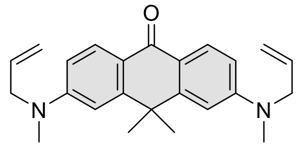

K19

N-Allylmethylamine (1.85 mL, 19.3 mmol) and compound **SI-25** (1 g, 1.93 mmol) were dissolved in dry DMSO (6 mL). Mixture was stirred and heated in a sealed vial at 100°C for 24h. Reaction course was monitored by TLC (hexane:EtOAc 3:7). Once reaction was finished mixture was poured into water

and extracted with EtOAc (4x 50 mL), washed with brine, dried over Na_2SO_4 and filtered. Filtrate was concentrated on rotary evaporator, the residue was deposited on celite and products were purified by flash column chromatography (Büchi Reveleris HP silica 40 g, gradient 5% to 40% hexane – EtOAc +5% constant DCM additive) to give 257 mg of target compound in 37% yield as yellow solid.

^1H NMR (400 MHz, d_6-DMSO) δ 7.96 (d, J = 8.9 Hz, 2H), 6.85 (d, J = 2.5 Hz, 2H), 6.77 (dd, J = 8.9, 2.5 Hz, 2H), 5.92 – 5.81 (m, 2H), 5.19 – 5.11 (m, 4H), 4.10 (d, J = 5.0 Hz, 4H), 3.06 (s, 6H), 1.64 (s, 6H). ^{13}C NMR (101 MHz, d_6-DMSO) δ 179.4, 152.1, 152.1, 133.4, 128.1, 118.8, 116.2, 110.8, 108.2, 53.9, 38.0, 37.7, 33.3. ESI-MS, positive mode: m/z = 361.4 $[M+H]^+$. HRMS (ESI) calcd for $C_{24}H_{29}N_2O$ $[M+H]^+$ 361.2279, found 361.2283.

3-(dimethylamino)-6-methoxy-9H-xanthen-9-one (**K20**):

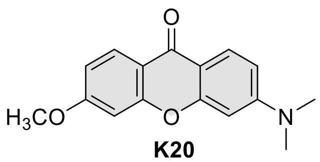

K20

Iodomethane (174 µL, 2.8 mmol) was added to a stirred suspension of K_2CO_3 (193 mg, 1.4 mmol) and **SI-27** (180 mg, 0.7 mmol) in acetone (10 mL). The reaction mixture was sealed with septa and stirred for 2h at 45°C. Then acetone was evaporated on rotary evaporator, water was added and mixture was extracted with DCM (3x 25 mL), washed with brine, dried over Na_2SO_4 and filtered. The filtrate was concentrated on rotary evaporator to give 370 mg of pure compound as a white solid in a quantitative yield.

^1H NMR (400 MHz, $CDCl_3$) δ 8.20 (d, J = 8.8 Hz, 1H), 8.13 (d, J = 9.0 Hz, 1H), 6.88 (dd, J = 8.8, 2.4 Hz, 1H), 6.81 (d, J = 2.4 Hz, 1H), 6.70 (dd, J = 9.0, 2.5 Hz, 1H), 6.47 (d, J = 2.4 Hz, 1H), 3.90 (s, 3H), 3.10 (s, 6H). ^{13}C NMR (101 MHz, $CDCl_3$) δ 175.4, 164.3, 158.4, 158.0, 154.7, 128.1, 127.9, 116.3, 112.2, 111.8, 109.6, 100.3, 97.0, 55.8, 40.3. ESI-MS, positive mode: m/z = 292.1 $[M+Na]^+$. HRMS (ESI) calcd for $C_{16}H_{15}NO_3Na$ $[M+Na]^+$ 292.0944, found 292.0943.

3,6-dimethoxy-10,10-dimethylanthracen-9(10H)-one (**K21**):

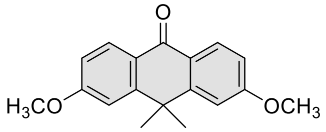

K21 (94%)

Iodomethane (445 µL, 7.1 mmol) was added to a stirred suspension of K_2CO_3 (650 mg, 4.7 mmol) and 3,6-dihydroxy-10,10-dimethyl-9(10H)-anthracenone[13] (300 mg, 1.18 mmol) in acetone (20 mL). The reaction mixture was sealed with septa and stirred overnight at 50°C. Then acetone was evaporated on rotary evaporator, water was added and mixture was extracted with EtOAc (3x 25 mL), washed with brine, dried over Na_2SO_4 and filtered. The filtrate was concentrated on rotary evaporator, the residue was deposited on celite and product was purified by flash column chromatography (Büchi Reveleris

HP silica 40 g, gradient 2% to 50% hexane – EtOAc) to give 312 mg of target compound in 94% yield as white solid. This compound has been obtained previuosly[24].

^{1}H NMR (400 MHz, $CDCl_3$) δ 8.34 (d, *J* = 8.8 Hz, 2H), 7.10 (d, *J* = 2.4 Hz, 2H), 6.97 (dd, *J* = 8.8, 2.5 Hz, 2H), 3.92 (s, 6H), 1.71 (s, 6H). ^{13}C NMR (101 MHz, $CDCl_3$) δ 181.9, 163.6, 152.8, 130.1, 124.0, 112.8, 111.7, 55.6, 38.3, 33.3. ESI-MS, positive mode: m/z = 305.1 $[M+Na]^+$. HRMS (ESI) calcd for $C_{18}H_{19}O_3$ $[M+H]^+$ 283.1329, found 283.1331.

General procedure **A** for the compounds **2-26**:

In a 50 mL round-bottom flask, a degassed solution of 3-bromophthalic acid (100 mg, 0.408 mmol, 1 eq) in anhydrous THF (5 mL) was cooled to -78°C (dry ice – acetone cooling bath). *n*-butyllithium (640 µL, 1.6M in hexane, 1.02 mmol, 2.5 eq) was introduced through a needle to the reaction mixture dropwise in 3-5 min, the color of reaction mixture starts to change to light orange once ~ the last 0.5 eq are being introduced .Once addition was complete mixture color changed to orange and stirring was continued at -78°C for 20 min. Meantime a corresponding ketone **K2**-**K21** (0.136 mmol, 0.33 eq) was dissolved in minimal amount of THF (1-30 mL, varies depending on ketone) and slowly injected to the reaction mixture with a syringe. The mixture was stirred for additional 10 min. at -78°C and then cooling bath was removed and reaction mixture was allowed to warm to room temperature. Stirring was continued for 30 min at rt and then 1 mL of glacial acetic acid (1 mL, 17.5 mmol) was injected with a syringe resulting in immediate change of color. After stirring for 5 min the THF was partially removed on rotary evaporator and water (20 mL) and solution of HCl was added (2 mL, 1M) and mixture was extracted with DCM or DCM/MeOH (9:1) mixture (5x 25 mL). The combined organic extracts were dried over Na_2SO_4 and filtered. Filtrate was concentrated on rotary evaporator, the residue was deposited on celite and products were purified by flash column chromatography.

NOTE: reaction is highly sensitive to the ratio of the used reagents. Even small amount of water can affect the concentration of alkyllithium reagents, therefore we highly recommend to use a freshly distilled anhydrous THF and if needed predetermine exact n-BuLi concentration prior usage by titration.

General procedure **B** for the compounds **2-26**:

In a 50 mL round-bottom flask, a degassed solution of 3-bromophthalic acid (200 mg, 0.816 mmol, 1 eq) in anhydrous THF (5 mL) was cooled to -78°C (dry ice – acetone cooling bath). *n*-Butyllithium (816 µL, 2.5M in hexane, 2.04 mmol, 2.5 eq) was introduced through a needle to the reaction mixture dropwise in 3-5 min, the color of reaction mixture starts to change to light orange once ~ the last 0.5 eq are being introduced .Once addition was complete mixture color changed to orange and stirring was continued at -

78°C for 20 min. Meantime a corresponding ketone **K5**-**K7** (0.136 mmol, 0.17 eq) was partially dissolved in THF (30 mL) and the obtained suspension was slowly injected to the reaction mixture with a syringe. The mixture was stirred for additional 10 min. at -78°C and then cooling bath was removed and reaction mixture was allowed to warm to room temperature. During the warm up stage, the suspension slowly turns into solution. Stirring was continued for 1h at rt and then 1 mL of glacial acetic acid (1 mL, 17.5 mmol) was introduced resulting in immediate change of color. After stirring for 5 min the THF was partially removed on rotary evaporator and water (20 mL) and solution of HCl was added (2 mL, 1M) and mixture was extracted with DCM or DCM/MeOH (9:1) mixture (5x 25 mL). The combined organic extracts were dried over Na_2SO_4 and filtered. Filtrate was concentrated on rotary evaporator, the residue was deposited on celite and products were purified by flash column chromatography.

4-SiR-COOH (**2**):

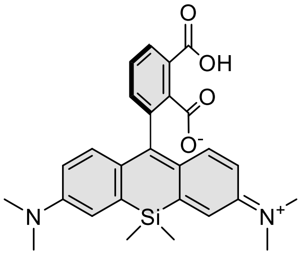

4-SiR-COOH (**2**)

Purified by flash column chromatography (Büchi Reveleris HP silica 40 g, gradient 5% to 60% hexane – EtOAc). Obtained 58 mg of target compound in 90% yield as a greenish solid by general method A. Analysis data conforms to previously published data[25].

^{1}H NMR (400 MHz, d_5-Pyridine) δ 8.17 (dd, *J* = 7.7, 0.9 Hz, 1H), 7.77 (t, *J* = 7.7 Hz, 1H), 7.54 (dd, *J* = 7.7, 0.9 Hz, 1H), 7.18 (d, *J* = 2.9 Hz, 2H), 7.04 (d, *J* = 9.0 Hz, 2H), 6.54 (dd, *J* = 9.0, 2.9 Hz, 2H), 2.85 (s, 12H), 0.73 (s, 3H), 0.65 (s, 3H). ^{1}H NMR (400 MHz, CD_3OD) δ 8.12 (dd, *J* = 7.8, 1.2 Hz, 1H), 7.75 (t, *J* = 7.7 Hz, 1H), 7.46 (dd, *J* = 7.7, 1.2 Hz, 1H), 7.34 (d, *J* = 2.8 Hz, 2H), 7.02 (d, *J* = 9.5 Hz, 2H), 6.80 (dd, *J* = 9.5, 2.8 Hz, 2H), 3.28 (s, 12H), 0.64 (s, 3H), 0.56 (s, 3H). ^{13}C NMR (101 MHz, d_5-Pyridine) δ 170.1, 169.4, 150.4 (overlapped with pyridine, visible in HMBC spectra), 157.1, 137.4, 135.6, 134.9, 132.3, 129.7, 129.2, 127.0, 124.2 (overlapped with pyridine, visible in HMBC spectra), 117.4, 114.6, 92.3, 40.4, 0.8, -0.8. ^{13}C NMR (101 MHz, CD_3OD) δ 170.8, 168.7, 168.7, 154.3, 147.2, 140.4, 133.2, 131.9, 131.5, 130.5, 121.8, 118.9, 116.1, 115.4, 41.2, -0.5, -1.8. ESI-MS, positive mode: m/z = 473.2 $[M+H]^+$. HRMS (ESI) calcd for $C_{27}H_{29}N_2O_4Si$ $[M+H]^+$ 473.1891, found 473.1890.

4-TMR-COOH (**3**):

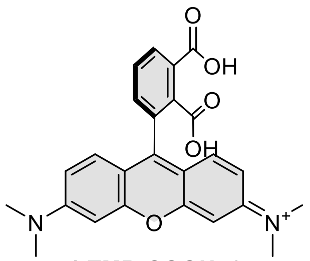

4-TMR-COOH (**3**)

Purified by flash column chromatography (Büchi Reveleris HP silica 24 g, gradient 5% to 50% DCM – DCM:MeOH (1:1) +0.1 constant additive of HCOOH). The compound was dissolved in MeCN and filtered through 0.45 µm PTFE filter and lyophilized from MeCN/H_2O mixture to obtain 53 mg of target compound in 84% yield as a dark red solid by general method A. Analysis data conforms to previously published data[25].

^{1}H NMR (400 MHz, CD_3OD) δ 8.25 (dd, *J* = 7.8, 1.2 Hz, 1H), 7.82 (t, *J* = 7.8 Hz, 1H), 7.60 (dd, *J* = 7.8, 1.2 Hz, 1H), 7.21 (d, *J* = 9.5 Hz, 2H), 7.08 (dd, *J* = 9.5, 2.4 Hz, 2H), 6.87 (d, *J* = 2.4 Hz, 2H), 3.29 (s, 12H). ^{13}C NMR (101 MHz, CD_3OD) δ 170.5, 168.6, 159.0, 159.0, 157.0, 136.8, 134.1, 132.8, 132.5, 132.2, 132.0, 131.0, 115.5, 115.2, 97.4, 41.0. ESI-MS, positive mode: m/z = 431.2 $[M+H]^+$. HRMS (ESI) calcd for $C_{25}H_{23}N_2O_5$ $[M+H]^+$ 431.1601, found 431.1606.

<u>Gram scale experimental procedure of </u>**4-TMR-COOH** (**3**) synthesis:

In a 500 mL round-bottom flask, a degassed solution of 3-bromophthalic acid (4.0 g, 16.3 mmol, 1 eq) in anhydrous THF (100 mL) was cooled to -78°C (dry ice – acetone cooling bath). *n*-Butyllithium (25.1 mL, 2.5M in hexane, 40.2 mmol, 2.5 eq) was introduced through a needle to the reaction mixture dropwise in 3-5 min, the color of reaction mixture starts to change to light orange once ~ the last 0.5 eq were being introduced .Once addition was complete mixture color changed to orange and stirring was continued at -78°C for 20 min. In meantime reaction mixture became cloudy and some of the lithium organics started to crash out of the solution. Ketone **K3** (1.5 g, 5.4 mmol, 0.33 eq) was dissolved in THF (70 mL) and slowly injected to the reaction mixture with a syringe. The mixture was stirred for additional 10 min. at -78°C and then cooling bath was removed and reaction mixture was allowed to warm to room temperature. Stirring was continued for 1h at rt and then glacial acetic acid (5 mL, 76.2 mmol) was injected with a syringe resulting in immediate change of color to dark violet. After stirring for 5 min the THF was partially removed on rotary evaporator and water (70 mL) and solution of HCl was added (15 mL, 1M) and mixture was extracted with DCM/MeOH (9:1) mixture (7x 100 mL). The combined organic extracts were dried over Na_2SO_4 and filtered. Filtrate was concentrated on rotary evaporator, the residue was deposited on celite and products were purified by flash column chromatography (Büchi Reveleris HP silica 80 g, gradient 5% to 50% DCM – DCM:MeOH (1:1) +0.1% constant additive of formic acid). The compound was dissolved in 50 mL MeCN and filtered through 0.45 µm PTFE filter. Acetonitrile was evaporated on rotary evaporator to yield 1.7 g compound in 74% yield as a dark violet solid.

*NOTE: The gram scale conditions were not fully optimized. The amount of solvent was reduced to minimal level and some crashing out of reactants from the reaction mixture was evident, thus we recommend to use larger volumes of THF for the ArLi generation and for dissolving **K3** ketone. Increase of solvent might result in better yield.*

4-610CP-COOH (**4**):

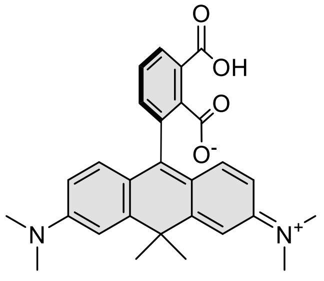

4-610CP-COOH (**4**)

Purified by flash column chromatography (Büchi Reveleris HP silica 24 g, gradient 20% to 100% DCM – DCM:MeOH 9:1) The compound was dissolved in MeCN and filtered through 0.45 µm PTFE filter and lyophilized from MeCN/H_2O mixture to give 45 mg of target compound in 72% yield as a dark violet solid by general method A. Analysis data conforms to previously published data[25].

^{1}H NMR (400 MHz, CD_3OD) δ 8.17 (dd, *J* = 7.8, 1.2 Hz, 1H), 7.77 (t, *J* = 7.8 Hz, 1H), 7.53 (dd, *J* = 7.7, 1.2 Hz, 1H), 7.23 (d, *J* = 2.5 Hz, 2H), 7.09 (d, *J* = 9.4 Hz, 2H), 6.84 (dd, *J* = 9.4, 2.5 Hz, 2H), 3.34 (s, 12H), 1.86 (s, 3H), 1.72 (s, 3H). ^{13}C NMR (101 MHz, CD_3OD) δ 170.6, 168.9, 158.3, 157.9, 138.9, 136.6, 135.9, 134.2, 131.8, 131.7, 130.6, 122.0, 114.0, 112.1, 48.6, 43.2, 41.0, 36.2, 32.3. ESI-MS, positive mode: m/z = 457.2 $[M+H]^+$. HRMS (ESI) calcd for $C_{28}H_{29}N_2O_4$ $[M+H]^+$ 457.2122, found 457.2119.

4-580R-COOH (**5**):

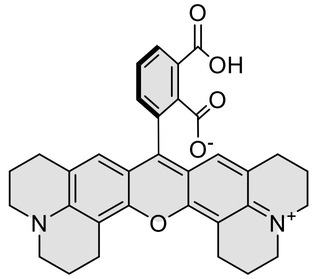

4-580R-COOH (**5**)

Purified by flash column chromatography (Büchi Reveleris HP silica 24 g, gradient 5% to 60% DCM – DCM:MeOH 1:1 + constant 0.1% HCOOH additive). The compound was dissolved in MeCN and filtered through 0.45 µm PTFE filter and lyophilized from MeCN/H_2O mixture to obtain 27 mg of target compound in 37% yield general method A and 56 mg in 77% yield by general method B as deep violet solid.

^{1}H NMR (400 MHz, *d*$_6$-DMSO, + CF_3COOD) δ 8.08 (dd, *J* = 7.8, 1.2 Hz, 1H), 7.77 (t, *J* = 7.7 Hz, 1H), 7.54 (dd, *J* = 7.7, 1.2 Hz, 1H), 6.57 (s, 2H), 3.56 – 3.44 (m, 8H), 2.96 (td, *J* = 6.2, 2.4 Hz, 4H), 2.69 – 2.56 (m, 4H), 2.02 – 1.93 (m, 4H), 1.83 (t, *J* = 6.0 Hz, 4H). ^{13}C NMR (101 MHz, *d*$_6$-DMSO + CF_3COOD, open form) δ 168.1, 167.3, 163.2, 151.4, 151.0, 135.3, 133.4, 131.2, 130.8, 130.5, 130.2, 126.2, 123.8, 119.9, 117.0, 114.1, 112.5, 105.1, 50.6, 50.1, 27.1, 20.3, 19.5, 19.4. ESI-MS, positive mode: m/z = 535.3 $[M+H]^+$. HRMS (ESI) calcd for C33H31N2O5 $[M+H]^+$ 535.2227, found 535.2224

4-645CP-COOH (**6**):

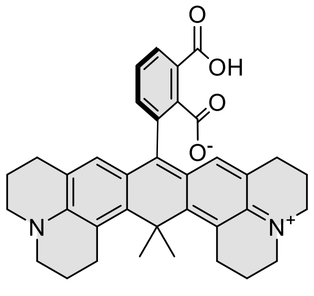

4-645CP-COOH (6)

Purified by flash column chromatography (Büchi Reveleris HP silica 24 g, gradient 5% to 40% DCM – DCM:MeOH 1:1 + constant 0.1% HCOOH additive). The compound was dissolved in MeCN and filtered through 0.45 µm PTFE filter and lyophilized from MeCN/H_2O mixture to obtain 9 mg of target compound in 12% yield general method A and 25 mg in 33% yield by general method B as dark blue solid.

^1H NMR (400 MHz, *d6*-DMSO + CF_3COOD) δ 8.02 (dd, *J* = 7.8, 1.2 Hz, 1H), 7.71 (t, *J* = 7.7 Hz, 1H), 7.44 (dd, *J* = 7.7, 1.2 Hz, 1H), 6.38 (s, 2H), 3.50 (dt, *J* = 21.3, 5.6 Hz, 8H), 3.10 – 2.88 (m, *J* = 5.4 Hz, 4H), 2.50 – 2.36 (m, 4H), 2.13 – 1.90 (m, 7H), 1.89 (s, 3H), 1.85-1.73 (m, 4H). ^{13}C NMR (101 MHz, *d6*-DMSO + CF_3COOD, open form) δ 168.3, 167.7, 163.4, 159.2, 154.1, 152.7, 135.8, 135.4, 133.5, 133.1, 130.7, 130.3, 129.8, 122.4, 121.7, 118.7, 117.5, 114.6, 51.8, 51.3, 39.6, 31.3, 30.5, 27.6, 27.5, 20.5, 20.4. ESI-MS, positive mode: m/z = 561.2 $[M+H]^+$. HRMS (ESI) calcd for $C_{36}H_{37}N_2O_4$ $[M+H]^+$ 561.2748, found 561.2737.

4-640CP-COOH (**7**):

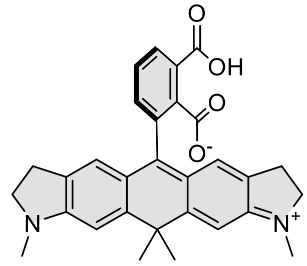

4-640CP-COOH (7)

Purified by flash column chromatography (Interchim puriflash 15 µm silica 25 g, gradient 5% to 30% DCM – DCM:MeOH 1:1 + constant 0.1% HCOOH additive). The compound was dissolved in MeCN and filtered through 0.45 µm PTFE filter and lyophilized from MeCN/H_2O mixture to obtain 21 mg of target compound in 32% yield general method A and 35 mg in 54% yield by general method B as blue solid.

^1H NMR (400 MHz, CD_3OD) δ 8.16 (dd, *J* = 7.9, 1.3 Hz, 1H), 7.75 (t, *J* = 7.7 Hz, 1H), 7.49 (dd, *J* = 7.7, 1.2 Hz, 1H), 7.02 (s, 2H), 6.66 (s, 2H), 3.84 (t, *J* = 8.0 Hz, 4H), 3.21 (s, 6H), 3.04 – 2.89 (m, 4H), 1.82 (s, 3H), 1.69 (s, 3H).^{13}C NMR (101 MHz, CD_3OD) δ 170.8, 168.9, 160.8, 160.6, 159.2, 136.8, 136.7, 134.4, 133.1, 131.6, 131.5, 130.7, 130.1, 122.8, 105.9, 55.9, 43.9, 36.0, 33.6, 32.3, 26.8. ESI-MS, positive mode: m/z = 481.3 $[M+H]^+$. HRMS (ESI) calcd for $C_{30}H_{29}N_2O_4$ $[M+H]^+$ 481.2122, found 481.2117

4-SiR700-COOH (**8**):

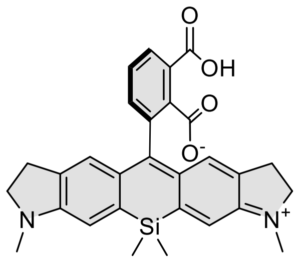

4-SiR700-COOH (**8**)

Purified by flash column chromatography (Büchi Reveleris HP silica 40 g, gradient 20% to 80% DCM – DCM:MeOH 9:1). Lyophilized from MeCN/H_2O mixture to give 46 mg of target compound in 69% yield as a yellow-greenish solid by general method A. Analysis data conforms to previously published data[26].

^{1}H NMR (400 MHz, *d*$_6$-DMSO) δ 7.71 (t, *J* = 7.5 Hz, 1H), 7.65 (d, *J* = 7.4 Hz, 1H), 7.18 (d, *J* = 7.6 Hz, 1H), 6.78 (s, 2H), 6.53 (s, 2H), 3.27 – 3.16 (m, 4H), 2.85 – 2.67 (m, 10H), 0.58 (s, 3H), 0.49 (s, 3H). ^{13}C NMR (101 MHz, *d*$_6$-DMSO) δ 168.5, 167.2, 156.6, 152.4, 134.9, 133.6, 133.3, 132.5, 132.1, 127.9, 125.1, 122.5, 120.7, 110.0, 90.5, 54.9, 35.3, 28.0, -0.2, -0.5. ESI-MS, positive mode: m/z = 497.2 $[M+H]^+$. HRMS (ESI) calcd for $C_{29}H_{29}N_2O_4Si$ $[M+H]^+$ 497.1891, found 497.1889.

4-SiR720-COOH (**9**):

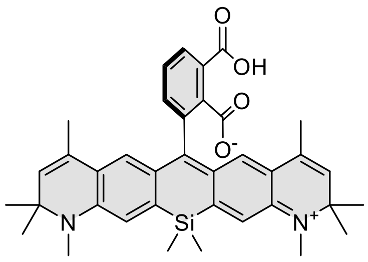

4-SiR720-COOH (**9**)

Purified by flash column chromatography (Büchi Reveleris HP silica 24g, gradient 5% to 50% DCM – DCM:MeOH 9:1). Lyophilized from MeCN/H_2O mixture and second time from 1,4-dioxane to give 55 mg of target compound (6:1 ratio complex with 1,4-dioxane as determined by NMR) in a 65% yield as a yellow solid by general method A.

^{1}H NMR (400 MHz, *d*$_6$-DMSO) δ 7.83 (t, *J* = 7.5 Hz, 1H), 7.78 (dd, *J* = 7.5, 1.1 Hz, 1H), 7.45 (dd, *J* = 7.6, 1.1 Hz, 1H), 6.75 (s, 2H), 6.38 (s, 2H), 5.35 (d, *J* = 1.6 Hz, 2H), 2.81 (s, 6H), 1.56 (d, *J* = 1.4 Hz, 6H), 1.25 (s, 6H), 1.24 (s, 6H), 0.62 (s, 3H), 0.51 (s, 3H). ^{13}C NMR (101 MHz, *d*$_6$-DMSO) δ 168.0, 167.0, 154.8, 143.8, 136.1 (2 peaks based on HMBC), 134.4, 132.8, 131.2, 130.5, 128.4, 126.1, 122.8, 122.1, 120.8, 114.2, 90.8, 56.2, 30.5, 27.7, 27.6, 17.4, 0.2, -1.6. ESI-MS, positive mode: m/z = 605.3 $[M+H]^+$. HRMS (ESI) calcd for $C_{37}H_{41}N_2O_4Si$ $[M+H]^+$ 605.2830, found 605.2828.

4-625CP-COOH (**10**):

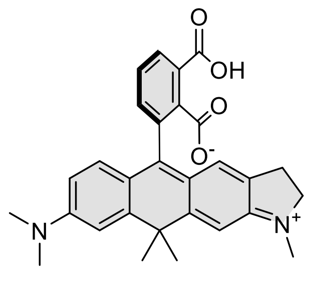

4-625CP-COOH (**10**)

Purified by flash column chromatography (Büchi Reveleris HP silica 40g, gradient 5% to 80% DCM – DCM:MeOH 9:1). Lyophilized from MeCN/H_2O mixture to give 45 mg of target compound in 71% yield as a violet solid by general method A.

^{1}H NMR (400 MHz, CD_3OD) δ 8.16 (dd, *J* = 7.8, 1.2 Hz, 1H), 7.75 (t, *J* = 7.8 Hz, 1H), 7.51 (dd, *J* = 7.7, 1.2 Hz, 1H), 7.15 (d, *J* = 2.6 Hz, 1H), 7.13 (s, 1H), 6.97 (d, *J* = 9.3 Hz, 1H), 6.77 – 6.71 (m, 2H), 3.93 (t, *J* = 7.7 Hz, 2H), 3.29 (s, 3H),

3.26 (s, 6H), 3.06 – 2.97 (m, 2H), 1.84 (s, 3H), 1.71 (s, 3H). ^{13}C NMR (101 MHz, CD_3OD) δ 170.7, 168.9, 162.9, 161.8, 160.1, 156.6, 156.3, 137.5, 136.7, 136.3, 134.3, 134.1, 131.7, 131.6, 130.8, 130.6, 123.6, 121.3, 113.1, 111.3, 107.0, 56.4, 43.6, 40.7, 36.1, 33.8, 32.3, 26.5. ESI-MS, positive mode: m/z = 469.3 $[M+H]^+$. HRMS (ESI) calcd for $C_{29}H_{29}N_2O_4$ $[M+H]^+$ 469.2122, found 469.2118

4-630CP-COOH (**11**):

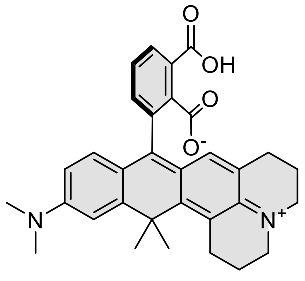

4-630CP-COOH (**11**)

Purified by flash column chromatography (Büchi Reveleris HP silica 24g, gradient 5% to 80% DCM – DCM:MeOH 9:1). Lyophilized from MeCN/H_2O mixture to give 42 mg of target compound in 62% yield as a violet solid by general method A.

^{1}H NMR (400 MHz, d_6-DMSO) δ 7.63 – 7.55 (m, 2H), 6.95 (dd, J = 7.1, 1.4 Hz, 1H), 6.78 (d, J = 2.6 Hz, 1H), 6.55 (dd, J = 8.9, 2.5 Hz, 1H), 6.39 (d, J = 8.8 Hz, 1H), 6.09 (s, 1H), 3.13 (dt, J = 15.4, 6.4 Hz, 4H), 2.95 – 2.86 (m, 8H), 2.46 – 2.33 (m, 2H), 1.87 (s, 5H), 1.80 (s, 3H), 1.72 (dd, J = 8.6, 4.5 Hz, 2H). ^{13}C NMR (101 MHz, d_6-DMSO) δ 169.1, 168.2, 157.2, 151.0, 150.1, 145.0, 140.7, 135.3, 128.2, 128.2, 127.9, 126.1, 124.8, 121.9, 121.6, 120.8, 117.5, 116.9, 112.4, 110.0, 88.9, 50.4, 49.7, 40.5, 37.8, 33.1, 32.4, 28.0, 27.9, 22.3, 21.6. ESI-MS, positive mode: m/z = 509.1 $[M+H]^+$. HRMS (ESI) calcd for $C_{32}H_{33}N_2O_4$ $[M+H]^+$ 509.2435, found 509.2423.

4-642CP-COOH (12):

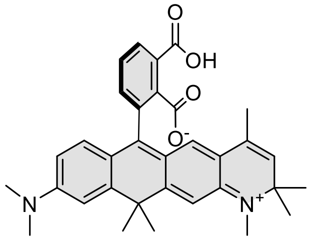

4-642CP-COOH (**12**)

Purified by flash column chromatography (Büchi Reveleris HP silica 40g, gradient 5% to 80% DCM – DCM:MeOH 9:1). Lyophilized from MeCN/H_2O mixture to give 48 mg of target compound in 66% yield as a blue solid by general method A.

^{1}H NMR (400 MHz, d_6-DMSO) δ 7.76 – 7.70 (m, 2H), 7.15 (dd, J = 7.0, 1.6 Hz, 1H), 6.91 (d, J = 2.6 Hz, 1H), 6.67 (s, 1H), 6.59 (dd, J = 8.9, 2.6 Hz, 1H), 6.48 (d, J = 8.8 Hz, 1H), 6.15 (s, 1H), 5.33 (d, J = 1.6 Hz, 1H), 2.93 (s, 6H), 2.84 (s, 3H), 1.81 (s, 3H), 1.70 (s, 3H), 1.54 (d, J = 1.3 Hz, 3H), 1.26 (s, 6H). ^{13}C NMR (101 MHz, d_6-DMSO) δ 168.1, 167.1, 155.9, 150.6, 146.2, 146.1, 145.2, 135.1, 132.4, 132.4, 130.7, 128.4, 128.0, 125.5, 122.2, 121.2, 121.0, 118.1, 117.4, 111.8, 109.1, 107.0, 87.2, 56.3, 40.0, 38.0, 34.5, 33.0, 30.7, 27.9, 27.6, 17.5. ESI-MS, positive mode: m/z = 523.3 [M+H]+. HRMS (ESI) calcd for $C_{33}H_{35}N_2O_4$ $[M+H]^+$ 523.2591, found 523.2590.

4-SiR665-COOH (**13**):

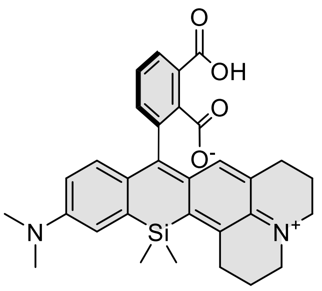

4-SiR665-COOH (**13**)

Purified by flash column chromatography (Büchi Reveleris HP silica 40g, gradient 20% to 80% DCM – DCM:MeOH 9:1). Lyophilized from MeCN/H_2O mixture to give 46 mg of target compound in 65% yield as a light blue solid by general method A.

^{1}H NMR (400 MHz, *d*$_6$-DMSO) δ 7.7 (t, *J* = 7.5 Hz, 1H), 7.6 (dd, *J* = 7.5, 1.1 Hz, 1H), 7.2 (dd, *J* = 7.7, 1.1 Hz, 1H), 6.9 (d, *J* = 2.7 Hz, 1H), 6.7 (dd, *J* = 9.0, 2.7 Hz, 1H), 6.6 (d, *J* = 8.9 Hz, 1H), 6.3 (s, 1H), 3.2 (t, *J* = 5.8 Hz, 2H), 3.1 – 3.1 (m, 2H), 2.9 (s, 8H), 2.5 – 2.4 (m, 2H), 2.0 – 1.9 (m, 2H), 1.8 – 1.7 (m, 2H), 0.7 (s, 3H), 0.6 (s, 3H). ^{13}C NMR (101 MHz, *d*$_6$-DMSO) δ 168.5, 167.1, 157.0, 149.2, 142.1, 135.6, 134.8, 133.2, 130.2, 129.9, 129.5, 127.8, 127.0, 126.2, 125.6, 125.1, 122.9, 120.5, 115.9, 114.1, 90.7, 49.4, 48.9, 39.8, 28.9, 27.7, 21.6, 21.1, 0.8, 0.7. ESI-MS, positive mode: m/z = 525.3 $[M+H]^+$. HRMS (ESI) calcd for $C_{31}H_{33}N_2O_4Si$ $[M+H]^+$ 525.2204, found 525.2202.

4-SiR670-COOH (**14**):

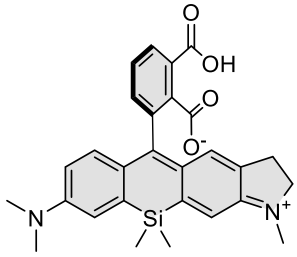

4-SiR670-COOH (**14**)

Purified by flash column chromatography (Büchi Reveleris HP silica 40g, gradient 10% to 80% DCM – DCM:MeOH 9:1). Lyophilized from MeCN/H_2O mixture to give 48 mg of target compound in 73% yield as a yellowish-green solid by general method A.

^{1}H NMR (400 MHz, *d*$_6$-DMSO) δ 7.7 (t, *J* = 7.6 Hz, 1H), 7.7 (dd, *J* = 7.4, 1.0 Hz, 1H), 7.2 (dd, *J* = 7.7, 1.0 Hz, 1H), 7.0 (dd, *J* = 2.2, 1.1 Hz, 1H), 6.8 (s, 1H), 6.7 – 6.6 (m, 2H), 6.5 (s, 1H), 3.2 (dt, *J* = 16.1, 8.2 Hz, 2H), 2.9 (s, 6H), 2.7 (s, 5H), 0.6 (s, 3H), 0.5 (s, 3H). ^{13}C NMR (101 MHz, *d*$_6$-DMSO) δ 168.3, 167.2, 156.0, 152.5, 149.1, 135.3, 134.8, 133.8, 133.5, 132.4, 132.0, 130.5, 127.9, 127.6, 125.4, 122.6, 121.1, 116.1, 113.8, 110.2, 90.5, 54.9, 39.8, 35.3, 28.0, -0.1, -0.8. ESI-MS, positive mode: m/z = 485.2 $[M+H]^+$. HRMS (ESI) calcd for $C_{28}H_{29}N_2O_4Si$ $[M+H]^+$ 485.1891, found 485.1886.

4-685SiR-COOH (**15**):

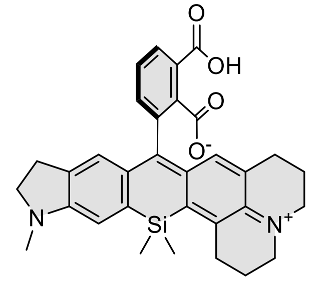

4-685SiR-COOH (**15**)

Purified by flash column chromatography (Büchi Reveleris HP silica 40g, gradient 1% to 15% DCM – DCM:MeOH). Lyophilized from MeCN/H_2O mixture and second time from 1,4-dioxane to give 40 mg of target compound (12:1 ratio complex with 1,4-dioxane as determined by NMR) in 54% yield as a light-blue solid by general method A.

^{1}H NMR (400 MHz, d_6-DMSO) δ 7.69 (t, J = 7.5 Hz, 1H), 7.64 (dd, J = 7.5, 1.2 Hz, 1H), 7.13 (dd, J = 7.5, 1.2 Hz, 1H), 6.75 (s, 1H), 6.50 (s, 1H), 6.30 (s, 1H), 3.26 – 3.07 (m, 6H), 2.90 (t, J = 6.3 Hz, 2H), 2.83 – 2.67 (m, 5H), 2.50 – 2.37 (m, 2H), 1.99 – 1.90 (m, 2H), 1.80 – 1.70 (m, 2H), 0.65 (s, 3H), 0.54 (s, 3H). ^{13}C NMR (101 MHz, d_6-DMSO) δ 168.7, 167.0, 157.4, 152.5, 142.0, 135.0, 133.8, 132.9, 132.7, 131.3, 129.8, 129.6, 127.9, 126.1, 125.5, 125.1, 123.0, 121.8, 120.3, 109.8, 90.9, 54.9, 49.4, 48.9, 35.3, 28.8, 28.0, 27.7, 21.6, 21.1, 0.8, 0.7. ESI-MS, positive mode: m/z = 537.1 $[M+H]^+$. HRMS (ESI) calcd for $C_{32}H_{33}N_2O_4Si$ $[M+H]^+$ 537.2204, found 537.2209.

4-SiR690-COOH (**16**):

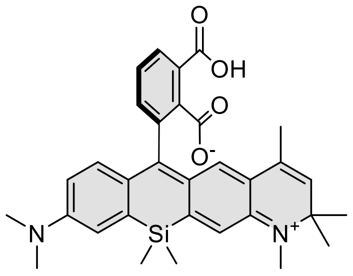

4-SiR690-COOH (**16**)

Purified by flash column chromatography (Büchi Reveleris HP silica 24g, gradient 20% to 80% hexane – EtOAc). Lyophilized from MeCN/H_2O mixture to give 47 mg of target compound in 65% yield as a yellowish-green solid by general method A.

^{1}H NMR (400 MHz, d_6-DMSO) δ 7.8 (t, J = 7.6 Hz, 1H), 7.7 (dd, J = 7.5, 1.0 Hz, 1H), 7.4 (dd, J = 7.7, 1.0 Hz, 1H), 7.0 (dd, J = 2.4, 0.9 Hz, 1H), 6.8 (s, 1H), 6.7 – 6.6 (m, 2H), 6.4 (s, 1H), 5.3 (d, J = 1.6 Hz, 1H), 2.9 (s, 6H), 2.8 (s, 3H), 1.6 (d, J = 1.4 Hz, 3H), 1.2 (d, J = 4.8 Hz, 6H), 0.6 (s, 3H), 0.5 (s, 3H). ^{13}C NMR (101 MHz, d_6-DMSO) δ 168.5, 167.6, 155.5, 149.7, 144.3, 136.4, 136.4, 134.9, 133.9, 131.7, 131.1, 131.0, 128.6, 127.9, 126.6, 126.2, 123.4, 122.2, 121.3, 116.9, 114.6, 114.0, 91.0, 56.7, 40.2, 30.9, 28.1, 28.0, 17.9, 0.6, -1.0. ESI-MS, positive mode: m/z = 539.2 $[M+H]^+$. HRMS (ESI) calcd for $C_{32}H_{35}N_2O_4Si$ $[M+H]^+$ 539.2361, found 539.2357.

4-TAllR-COOH (**17**):

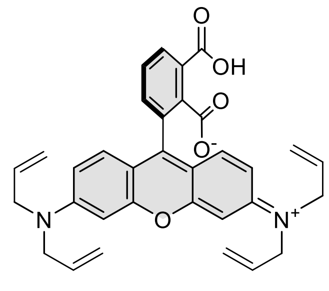

4-TAllR-COOH (17)

Purified by flash column chromatography (Büchi Reveleris HP silica 24g, gradient 1% to 25% DCM – MeOH). Lyophilized from MeCN/H_2O mixture to give 46 mg of target compound in 63% yield as a pink solid by general method A.

^{1}H NMR (400 MHz, CD_3OD) δ 8.11 (dd, *J* = 7.8, 1.2 Hz, 1H), 7.64 (t, *J* = 7.7 Hz, 1H), 7.38 (dd, *J* = 7.6, 1.2 Hz, 1H), 7.22 (d, *J* = 9.4 Hz, 2H), 6.99 – 6.93 (m, 2H), 6.90 (d, *J* = 2.5 Hz, 2H), 5.93 (ddt, *J* = 17.1, 10.0, 4.7 Hz, 4H), 5.28 – 5.18 (m, 8H), 4.28 – 4.17 (m, 8H). ^{13}C NMR (101 MHz, CD_3OD) δ 172.7, 170.8, 158.7, 157.7, 139.1, 135.3, 135.2, 133.8, 133.1, 132.8, 132.4, 132.4, 130.1, 117.7, 115.0, 114.7, 98.2, 54.5. ESI-MS, positive mode: m/z = 535.2 $[M+H]^+$. HRMS (ESI) calcd for $C_{33}H_{31}N_2O_5$ $[M+H]^+$ 535.2227, found 535.2224.

4-DAllR-COOH (**18**):

Purified by flash column chromatography (Büchi Reveleris HP silica 40g, gradient 1% to 25% DCM – MeOH). Lyophilized from MeCN/H_2O mixture to give 49 mg of target compound in 75% yield as a pink solid by general method A.

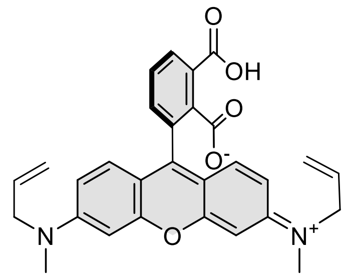

4-DAllR-COOH (18)

^{1}H NMR (400 MHz, CD_3OD) δ 8.19 (dd, *J* = 7.8, 1.2 Hz, 1H), 7.78 (t, *J* = 7.8 Hz, 1H), 7.56 (dd, *J* = 7.7, 1.2 Hz, 1H), 7.18 (d, *J* = 9.5 Hz, 2H), 7.05 (dd, *J* = 9.5, 2.5 Hz, 2H), 6.92 (d, *J* = 2.4 Hz, 2H), 5.89 (ddt, *J* = 17.2, 10.0, 4.8 Hz, 2H), 5.25 – 5.11 (m, 4H), 4.31 – 4.20 (m, 4H), 3.26 (s, 6H). ^{13}C NMR (101 MHz, CD_3OD) δ 170.4, 168.7, 159.2, 158.9, 157.4, 136.7, 134.1, 133.0, 132.5, 132.2, 132.2, 132.1, 131.1, 117.8, 115.8, 115.5, 97.8, 56.2, 39.6. ESI-MS, positive mode: m/z = 483.2 [M+H]+. HRMS (ESI) calcd for $C_{29}H_{27}N_2O_5$ $[M+H]^+$ 483.1914, found 483.1910.

4-DAllCP-COOH 19:

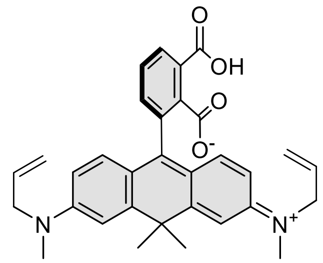

4-DAllCP-COOH (19)

Purified by flash column chromatography (Büchi Reveleris HP silica 40g, gradient 10% to 100% DCM – DCM:MeOH 9:1). Lyophilized from MeCN/H_2O mixture to give 50 mg of target compound in 73% yield as a dark violet solid by general method A.

^{1}H NMR (400 MHz, CD_3OD) δ 8.03 (dd, *J* = 7.6, 1.0 Hz, 1H), 7.72 (t, *J* = 7.7 Hz, 1H), 7.22 (dd, *J* = 7.7, 1.0 Hz, 1H), 7.00 (d, *J* = 2.6 Hz, 2H), 6.71 (d, *J* =

9.0 Hz, 2H), 6.62 (dd, *J* = 9.0, 2.6 Hz, 2H), 5.87 (ddt, *J* = 17.1, 10.3, 4.9 Hz, 2H), 5.19 – 5.11 (m, 4H), 4.09 – 4.02 (m, 4H), 3.06 (s, 6H), 1.82 (s, 3H), 1.71 (s, 3H). ^{13}C NMR (101 MHz, CD_3OD) δ 172.6, 169.6, 152.8, 150.6, 134.8, 134.3, 133.4, 132.0, 131.6, 131.3, 129.2, 128.0, 120.0, 116.7, 113.1, 111.0, 56.0, 40.4, 38.8, 35.7, 33.1. ESI-MS, positive mode: m/z = 509.2 $[M+H]^+$. HRMS (ESI) calcd for $C_{32}H_{33}N_2O_4$ $[M+H]^+$ 509.2435, found 509.2424.

Compound **20**:

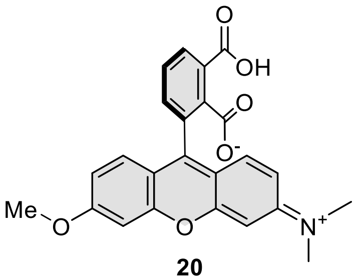

20

Purified by flash column chromatography (Büchi Reveleris HP silica 40g, gradient 1% to 20% DCM – DCM:MeOH 1:1 +constant 0.1% additive of HCOOH). Lyophilized from MeCN/H_2O mixture to give 43 mg of target compound in 76% yield as a white solid by general method A.

^{1}H NMR (400 MHz, *d₆*-DMSO) δ 7.80 (d, *J* = 4.3 Hz, 2H), 7.32 (t, *J* = 4.3 Hz, 1H), 6.93 – 6.86 (m, 1H), 6.70 (s, 2H), 6.61 – 6.43 (m, 3H), 3.81 (s, 3H), 2.95 (s, 6H). ^{13}C NMR (101 MHz, d_6-DMSO) δ 167.1, 166.9, 161.0, 153.3, 152.1, 152.0, 151.8, 135.5, 132.8, 129.1, 129.0, 128.4, 125.7, 122.5, 111.6, 111.0, 109.3, 105.2, 100.8, 97.9, 82.8, 55.6, 39.8. ESI-MS, negative: m/z = 416.1 $[M-H]^-$. HRMS (ESI) calcd for $C_{24}H_{18}NO_6$ $[M-H]^-$ 416.1140, found 416.1153.

Compound **21**:

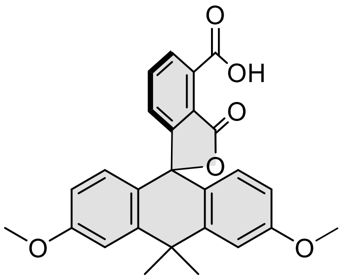

21

Purified by flash column chromatography (Büchi Reveleris HP silica 40g, gradient 20% to 100% DCM – DCM:MeOH 9:1). Lyophilized from MeCN/H_2O mixture and second time from 1,4-dioxane to give 53 mg of target compound (1:1 ratio complex with 1,4-dioxane as determined by NMR) in 75% yield as a white solid by general method A.

^{1}H NMR (400 MHz, d_6-DMSO) δ 7.79 – 7.71 (m, 2H), 7.28 (d, *J* = 2.6 Hz, 2H), 7.15 (dd, *J* = 7.2, 1.5 Hz, 1H), 6.83 (dd, *J* = 8.8, 2.6 Hz, 2H), 6.65 (d, *J* = 8.8 Hz, 2H), 3.80 (s, 6H), 1.83 (s, 3H), 1.73 (s, 3H). ^{13}C NMR (101 MHz, d_6-DMSO) δ 167.8, 166.9, 159.8, 155.9, 146.5, 135.6, 132.4, 128.8, 128.7, 125.5, 122.6, 121.6, 113.7, 111.6, 85.1, 55.3, 38.1, 34.1, 33.3. ESI-MS, negative mode: m/z = 429.1 $[M-H]^-$. HRMS (ESI) calcd for $C_{26}H_{21}O_6$ $[M-H]^-$ 429.1344, found 429.1342.

Compound **22**:

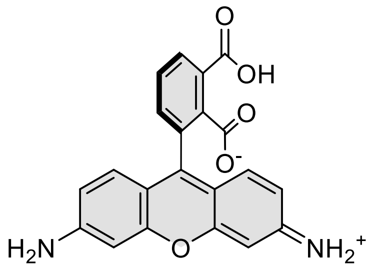

4-505R-COOH (22)

1,3-dimethylbarbituric acid (100 mg, 0.64 mmol) and $Pd(PPh_3)_4$ (15 mg, 0.013 mmol) were added to a solution of **4-TAllR-COOH** (**17**) (50 mg, 0.094 mmol) in MeCN (10 mL). The reaction mixture was purged with argon and the vial was sealed. The mixture was stirred at 45°C. The reaction course was monitored by LC/MS analysis and reaction was complete in 10h of heating. The vial was opened and water (10 mL), MeCN (5 mL) and HCOOH (200 µL) were added to dissolve the formed precipitants. The obtained mixture was filtered through 0.45 µm PTFE filter to remove palladium. The solution was concentrated and dissolved in minimum amount of MeCN/H_20 mixture and purified by the preparative HPLC (preparative column: Agilent 5 Prep-C18, 5 µm, 100 x 50 mm; solvent A: H2O + 0.2% v/v HCOOH, solvent B MeCN; temperature 25 °C, gradient A:B - 2 min 80:20 isocratic, 2-15 min 80:20 to 20:80 gradient, 15-20 20:80 isocratic). Fractions containing the product were collected, solvent was removed and obtained residue was lyophilized from 50:50 MeCN:H_2O mixture to obtain 21 mg of product as orange-brown solid in 60% yield.

^{1}H NMR (400 MHz, CD_3OD) δ 8.21 (dd, *J* = 7.8, 1.2 Hz, 1H), 7.81 (t, *J* = 7.8 Hz, 1H), 7.60 (dd, *J* = 7.7, 1.2 Hz, 1H), 7.11 (d, *J* = 9.1 Hz, 2H), 6.84 (dd, *J* = 9.0, 2.1 Hz, 2H), 6.81 (d, *J* = 2.1 Hz, 2H). ^{13}C NMR (101 MHz, CD_3OD) δ 170.4, 168.7, 161.6, 159.7, 157.6, 136.7, 134.0, 133.6, 132.4, 132.1, 131.0, 117.9, 115.1, 98.3. ESI-MS, positive mode: m/z = 375.3 $[M+H]^+$. HRMS (ESI) calcd for $C_{21}H_{15}N_2O_5$ $[M+H]^+$ 375.0975, found 375.0965.

4-525R-COOH (**23**):

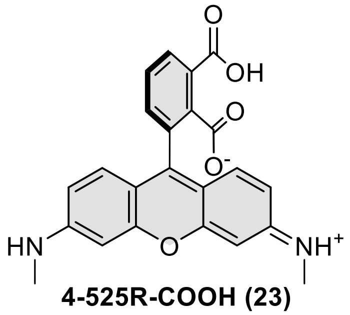

4-525R-COOH (23)

1,3-dimethylbarbituric acid (50 mg, 0.32 mmol) and $Pd(PPh_3)_4$ (10 mg, 0.0086 mmol) were added to a solution of **4-DAllR-COOH** (**18**) (65 mg, 0.135 mmol) in MeCN (10 mL). The reaction mixture was purged with argon and the vial was sealed. The mixture was stirred at 40°C overnight. The reaction completion was confirmed by LC/MS analysis. The vial was and opened and acetonitrile was evaporated to dryness on rotary evaporator. The crude mixture was deposited on celite and product was purified by flash column chromatography (Büchi Reveleris HP silica 40g, gradient 2% to 80% DCM –MeOH). The compound was dissolved in MeCN and filtered through 0.45 µm PTFE filter and lyophilized from MeCN/H_2O mixture and

second time from 1,4-dioxane to give 39 mg of target compound (13:1 ratio complex with 1,4-dioxane as determined by NMR) in 71% yield.

^{1}H NMR (400 MHz, *d_6*-DMSO) δ 7.62 (t, *J* = 7.5 Hz, 1H), 7.53 (d, *J* = 7.4 Hz, 1H), 7.04 (d, *J* = 7.5 Hz, 1H), 6.47 (d, *J* = 8.5 Hz, 2H), 6.43 (s, 2H), 6.39 – 6.33 (m, 4H), 2.71 (s, 6H). ^{13}C NMR (101 MHz, *d_6*-DMSO) δ 168.6, 167.8, 153.0, 152.4, 151.0, 134.0, 128.5, 128.4, 127.9, 123.3, 110.2, 110.2, 106.8, 96.1, 29.5. ESI-MS, positive mode: m/z = 403.1 $[M+H]^+$. HRMS (ESI) calcd for $C_{23}H_{19}N_2O_5$ $[M+H]^+$ 403.1288, found 403.1288.

4-580CP-COOH (**24**):

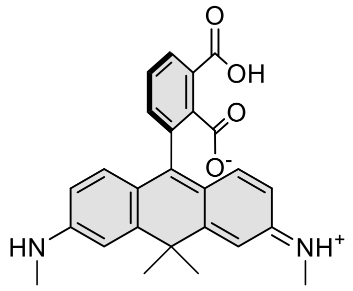

4-580CP-COOH (**24**)

1,3-dimethylbarbituric acid (50 mg, 0.32 mmol) and $Pd(PPh_3)_4$ (10 mg, 0.0086 mmol) were added to a solution of **4-DAllCP-COOH** (**19**) (55 mg, 0.108 mmol) in MeCN (10 mL). The reaction mixture was purged with argon and the vial was sealed. The mixture was stirred at 40°C overnight. The reaction completion was confirmed by LC/MS analysis. The vial was opened and acetonitrile was evaporated to dryness on rotary evaporator. The crude mixture was deposited on celite and product was purified by flash column chromatography (Büchi Reveleris HP silica 40g, gradient 2% to 40% DCM –MeOH). The compound was dissolved in MeCN and filtered through 0.45 µm PTFE filter and lyophilized from MeCN/H_2O mixture and second time from 1,4-dioxane to give 41 mg of target compound (5:1 ratio complex with 1,4-dioxane as determined by NMR) in 85% yield. Analysis data conforms to previously published data[25].

^{1}H NMR (400 MHz, CD_3OD) δ 8.17 (dd, *J* = 7.9, 1.1 Hz, 1H), 7.76 (t, *J* = 7.7 Hz, 1H), 7.52 (dd, *J* = 7.7, 1.2 Hz, 1H), 7.11 (d, *J* = 2.2 Hz, 2H), 7.03 (d, *J* = 9.2 Hz, 2H), 6.64 (dd, *J* = 9.2, 2.2 Hz, 2H), 3.06 (s, 6H), 1.81 (s, 3H), 1.68 (s, 3H). ^{13}C NMR (101 MHz, cd_3od) δ 170.6, 168.9, 162.5, 159.4, 158.6, 139.6, 136.6, 136.0, 134.2, 131.7, 131.7, 130.6, 122.1, 112.1 (visible from HSQC), 42.7, 35.8, 31.9, 30.1. ESI-MS, positive mode: m/z = 429.2 $[M+H]^+$. HRMS (ESI) calcd for $C_{26}H_{25}N_2O_4$ $[M+H]^+$ 429.1809, found 429.1807.

4-DMRh-COOH (**25**):

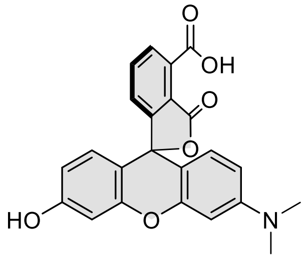

4-DMRh-COOH (**25**)

Compound **20** (60 mg, 0.144 mmol) was suspended in dry 1,2-dichloroethane (20 mL) in ace pressure tube and 6 ml of 1M BBr_3 (6 mmol) solution in DCM was added. The tube was sealed and stirred at 55°C (silicon oil bath temperature) for 24h. Reaction completion was confirmed by LC/MS analysis. The mixture was cooled to room temperature and the tube was carefully opened and poured into water (20 mL) and the mixture was extracted

with DCM/MeOH (9:1) mixture (7x 25 mL). The extracts were combined, dried over Na_2SO_4 and filtered. Filtrate was evaporated and the obtained crude mixture was dissolved in MeCN/H_2O + 0.2 HCOOH 1:1 mixture and purified by the preparative HPLC (preparative column: Agilent 5 Prep-C18, 5 µm, 100 x 50 mm; solvent A: H2O + 0.2% v/v HCOOH, solvent B MeCN; temperature 25 °C, gradient A:B - 2 min 80:20 isocratic, 2-15 min 80:20 to 20:80 gradient, 15-20 20:80 isocratic). Fractions containing the product were collected, solvent was removed and obtained residue was lyophilized from 50:50 MeCN:H_2O mixture to obtain 41 mg of product as red solid in 70% yield.

^{1}H NMR (400 MHz, CD_3OD) δ 8.24 (dd, *J* = 7.9, 1.2 Hz, 1H), 7.84 (t, *J* = 7.8 Hz, 1H), 7.63 (dd, *J* = 7.7, 1.2 Hz, 1H), 7.37 – 7.26 (m, 3H), 7.07 (dd, *J* = 9.7, 2.3 Hz, 2H), 7.03 (dd, *J* = 9.1, 2.3 Hz, 1H), 3.40 (s, 6H). ^{13}C NMR (101 MHz, CD_3OD) δ 170.3, 169.3, 168.7, 160.7, 160.1, 158.9, 158.5, 136.5, 133.9, 133.7, 133.4, 132.6, 132.4, 132.1, 131.2, 118.5, 118.2, 117.7, 116.5, 103.3, 97.6, 41.6. ESI-MS, negative mode: m/z = 402.1 [M-H]$^-$. HRMS (ESI) calcd for $C_{23}H_{16}NO_6$ [M-H]$^-$ 402.0983, found 402.0983.

4-CFL-COOH (**26**):

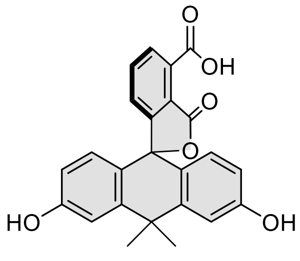

4-CFL-COOH (**26**)

Compound **21** (70 mg, 0.163 mmol) was suspended in dry 1,2-dichloroethane (20 mL) in ace pressure tube and 6 ml of 1M BBr_3 (6 mmol) solution in DCM was added. The tube was sealed and stirred at 55°C (silicon oil bath temperature) for 72h. Reaction completion was confirmed by LC/MS analysis. The mixture was cooled to room temperature and the tube was carefully opened and poured into water (20 mL) and the mixture was extracted with DCM/MeOH (9:1) mixture (7x 25 mL). The extracts were combined, dried over Na_2SO_4 and filtered. Filtrate was evaporated and the obtained crude mixture was deposited on celite and purified by flash column chromatography (Büchi Reveleris HP silica 40g, gradient 2% to 25% DCM –MeOH) to give 43 mg in 65% yield as white solid.

^{1}H NMR (400 MHz, *d$_6$*-DMSO) δ 9.94 (s, 2H), 7.72 – 7.58 (m, 2H), 7.07 (dd, *J* = 9.9, 1.9 Hz, 3H), 6.62 (dd, *J* = 8.6, 2.4 Hz, 2H), 6.50 (d, *J* = 8.6 Hz, 2H), 1.72 (s, 3H), 1.62 (s, 3H). ^{13}C NMR (101 MHz, *d$_6$*-DMSO) δ 168.1, 167.6, 158.1, 155.8, 146.5, 140.7, 135.2, 128.8, 128.1, 124.6, 121.5, 121.4, 114.9, 112.6, 85.5, 37.6, 34.4, 33.1. ESI-MS, positive mode: m/z = 403.1 [M+H]$^+$. HRMS (ESI) calcd. for $C_{24}H_{19}O_6$ [M+H]$^+$ 403.1176, found 403.1170.

General procedure for the synthesis of compounds **27**-**46**:

Into a solution of corresponding rhodamine dye (5 µmol, 1 eq; **2-16** and **22-26**) and DIPEA (5 µL) in DMSO (100 µL) a solution of HATU (6.5 µmol, 1.3 eq) in DMSO (100 µL) was added and the mixture was mixed for 1 min. Then a solution of 4-((4-(aminomethyl)benzyl)oxy)-6-chloropyrimidin-2-amine (7.5 µmol ,1.5 eq) in DMSO (100 µL) was added at once. Reactions were usually over after 30 min and their course was monitored by LC/MS analysis. Once reaction was finished it was quenched with 20 µL of formic acid and diluted with water and acetonitrile to 2 mL volume and was further purified by the means of preparative HPLC (preparative column: Agilent 5 Prep-C18, 5 µm, 100 x 50 mm).

4-CFL-CP (**27**): JB603

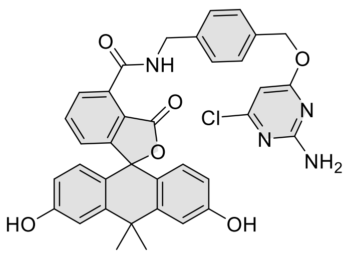

Compound was purified by preparative HPLC (solvent A: H_2O + 0.2% v/v HCOOH, solvent B: acetonitrile; temperature 25 °C, gradient A:B - 3 min 70:30 isocratic, 4-20 min 70:30 to 0:100 gradient, 20-25 min 0:100 isocratic, 40 mL/min flow). Fractions containing the product were collected, evaporated and lyophilized from acetonitrile: water mixture. Yield 42% (1.4 mg) of white solid.

^{1}H NMR (400 MHz, d_6-DMSO) δ 9.72 (s, 2H), 9.49 (t, *J* = 5.8 Hz, 1H), 7.80 (dd, *J* = 7.5, 1.0 Hz, 1H), 7.72 (t, *J* = 7.6 Hz, 1H), 7.49 (d, *J* = 8.3 Hz, 2H), 7.44 (d, *J* = 8.3 Hz, 2H), 7.13 – 7.04 (m, 5H), 6.62 (d, *J* = 1.4 Hz, 4H), 6.14 (s, 1H), 5.32 (s, 2H), 4.59 (d, *J* = 5.8 Hz, 2H), 1.73 (s, 3H), 1.64 (s, 3H). ESI-MS, positive mode: m/z = 671.2 $[M+Na]^+$. HRMS (ESI) calcd for $C_{36}H_{29}ClN_4O_6Na$ $[M+Na]^+$ 671.1668, found 671.1651.

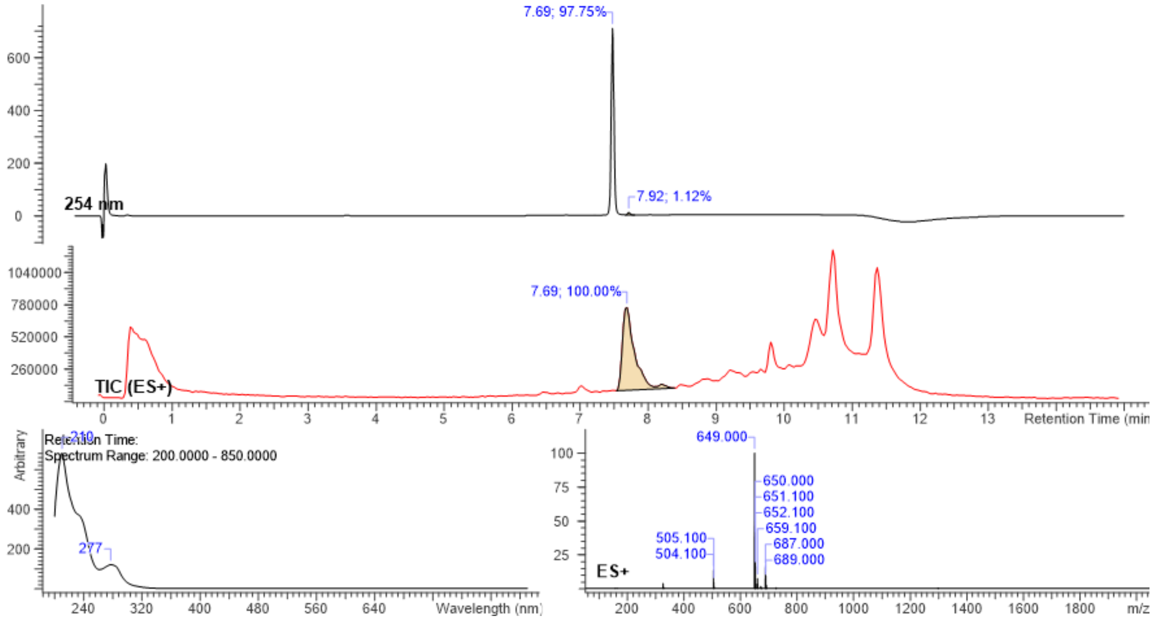

Supplementary Figure 29. LC/MS analysis results of compound **27**.

4-505R-CP (**28**):

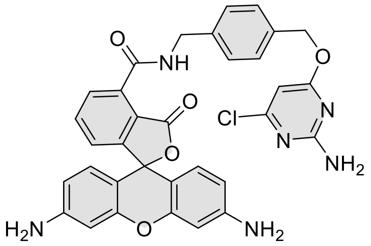

Compound was purified by preparative HPLC (solvent A: H_2O + 10mM NH_4COOH pH = 3.6, solvent B: acetonitrile; temperature 25 °C, gradient A:B - 3 min 80:20 isocratic, 4-20 min 80:20 to 0:100 gradient, 20-25 min 0:100 isocratic, 40 mL/min flow). Fractions containing the product were collected, evaporated and lyophilized from acetonitrile water mixture. The obtained solid was dissolved in 700 µL of d_6-DMSO. The samples from DMSO solution were diluted x100 in PBS +0.1%SDS and concentration was determined spectroscopically with Nanodrop. Determined stock concentration was 4.1 mM which constitutes to 58% yield (1.8 mg).

^{1}H NMR (400 MHz, d_6-DMSO) δ 9.52 (t, J = 5.8 Hz, 1H), 7.85 (dd, J = 7.5, 1.1 Hz, 1H), 7.78 (t, J = 7.6 Hz, 1H), 7.48 (d, J = 8.2 Hz, 2H), 7.43 (d, J = 8.3 Hz, 2H), 7.26 (dd, J = 7.6, 1.1 Hz, 1H), 7.10 (s, 2H), 6.45 (d, J = 8.6 Hz, 2H), 6.38 (d, J = 2.2 Hz, 2H), 6.29 (dd, J = 8.5, 2.2 Hz, 2H), 6.14 (s, 1H), 5.58 (s, 4H), 5.31 (s, 2H), 4.58 (d, J = 5.7 Hz, 2H). ESI-MS, positive mode: m/z = 621.2 $[M+H]^+$. HRMS (ESI) calcd for $C_{33}H_{31}ClN_6O_5$ $[M+H]^+$ 621.1648, found 621.1639.

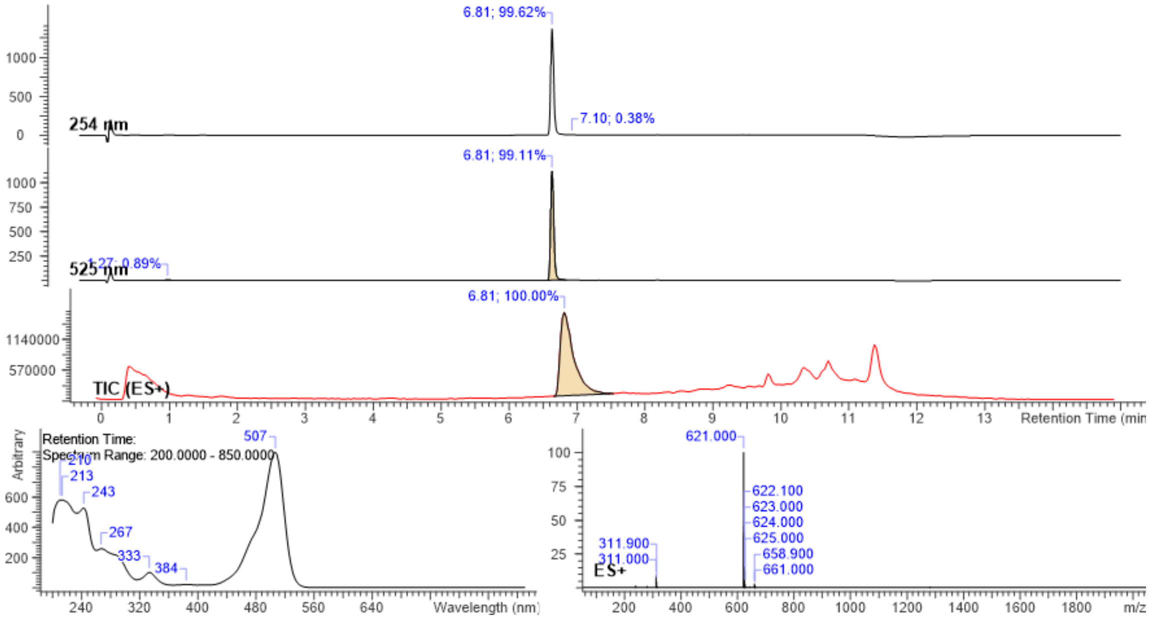

Supplementary Figure 30. LC/MS analysis results of compound **28**.

4-525R-CP (**29**):

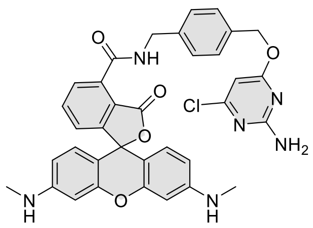

Compound was purified by preparative HPLC (solvent A: H_2O + 10mM NH_4COOH pH = 3.6, solvent B: acetonitrile; temperature 25 °C, gradient A:B - 3 min 80:20 isocratic, 4-20 min 80:20 to 0:100 gradient, 20-25 min 0:100 isocratic, 40 mL/min flow). Fractions containing the product were collected, evaporated and lyophilized from acetonitrile water mixture. The obtained solid was dissolved in 700 µL of d_6-DMSO. The samples from DMSO solution were diluted x100 in PBS +0.1%SDS and concentration was determined spectroscopically with Nanodrop. Determined stock concentration was 3.5 mM which constitutes to 49% yield (1.6 mg).

^{1}H NMR (400 MHz, d_6-DMSO) δ 9.53 (t, J = 5.8 Hz, 1H), 7.86 (dd, J = 7.5, 1.1 Hz, 1H), 7.78 (t, J = 7.6 Hz, 1H), 7.48 (d, J = 8.2 Hz, 2H), 7.43 (d, J = 8.3 Hz, 2H), 7.25 (dd, J = 7.6, 1.0 Hz, 1H), 7.14 – 7.06 (m, 2H), 6.54 – 6.47 (m, 2H), 6.37 – 6.25 (m, 4H), 6.18 (q, J = 4.9 Hz, 2H), 6.14 (s, 1H), 5.31 (s, 2H), 4.59 (d, J = 5.8 Hz, 2H), 2.69 (d, J = 4.9 Hz, 6H). ESI-MS, positive mode: m/z = 649.2 $[M+H]^+$. HRMS (ESI) calcd for $C_{35}H_{30}ClN_6O_5$ $[M+H]^+$ 649.1961, found 649.1955.

LC/MS:

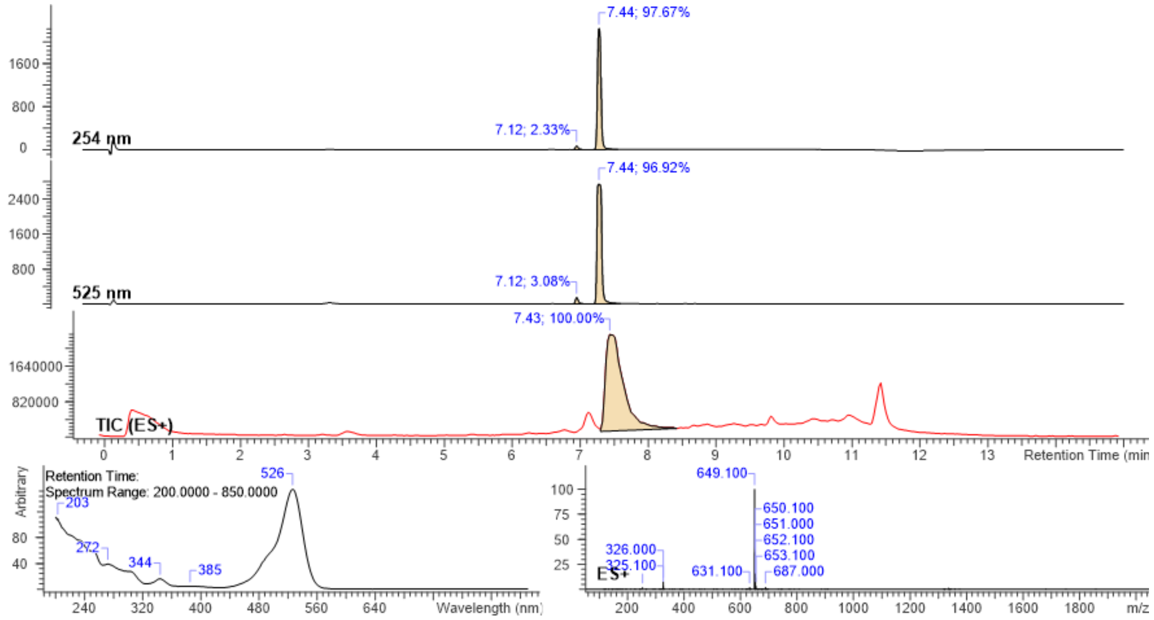

Supplementary Figure 31. LC/MS analysis results of compound **29**.

4-DMRh-CP (30):

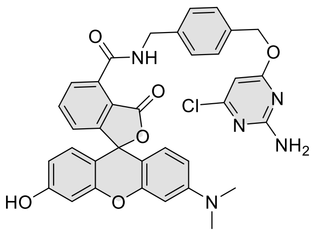

Compound was purified by preparative HPLC (solvent A: H_2O + 0.2% v/v HCOOH, solvent B: acetonitrile; temperature 25 °C, gradient A:B - 3 min 70:30 isocratic, 4-20 min 70:30 to 0:100 gradient, 20-25 min 0:100 isocratic, 40 mL/min flow). Fractions containing the product were collected, evaporated and lyophilized from acetonitrile: water mixture. Yield 52% (1.7 mg) of light red solid.

^{1}H NMR (400 MHz, d_6-DMSO) δ 10.13 (br s, 1H), 9.50 (t, J = 5.7 Hz, 1H), 7.84 (dd, J = 7.6, 1.0 Hz, 1H), 7.76 (t, J = 7.6 Hz, 1H), 7.47 (d, J = 8.3 Hz, 2H), 7.41 (d, J = 8.3 Hz, 2H), 7.26 (dd, J = 7.7, 1.1 Hz, 1H), 7.08 (s, 2H), 6.67 (d, J = 8.7 Hz, 1H), 6.65 – 6.60 (m, 2H), 6.53 – 6.47 (m, 3H), 6.13 (s, 1H), 5.30 (s, 2H), 4.57 (d, J = 5.7 Hz, 2H), 2.93 (s, 6H). ESI-MS, positive mode: m/z = 650.2 $[M+H]^+$. HRMS (ESI) calcd for $C_{35}H_{29}N_5O_6Cl$ $[M+H]^+$ 650.1801, found 650.1785.

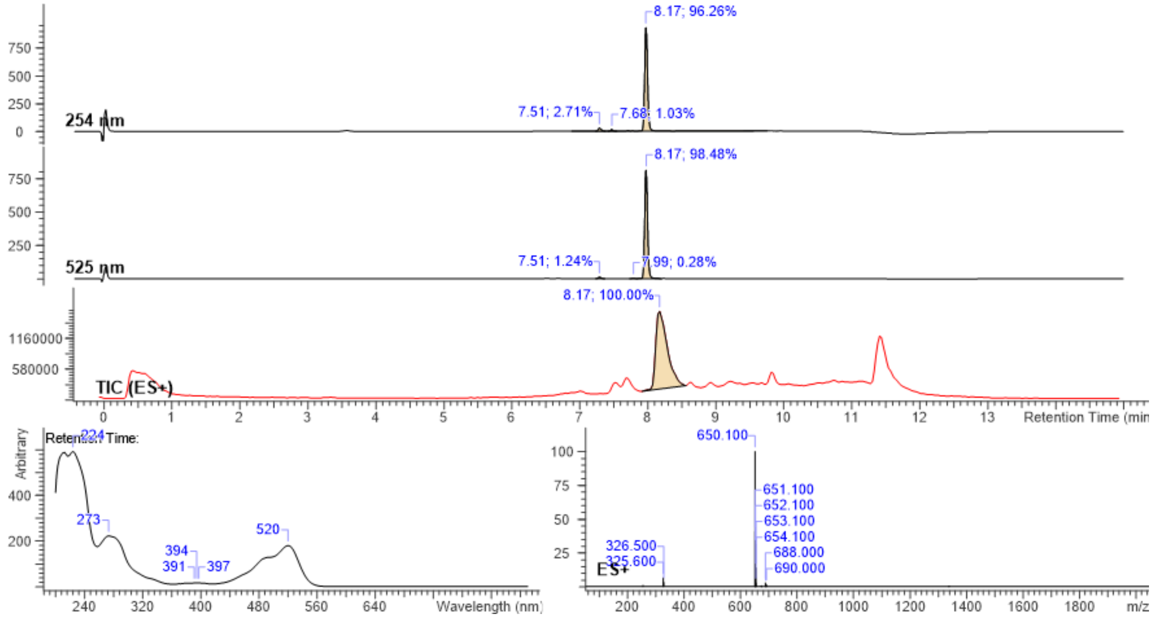

Supplementary Figure 32. LC/MS analysis results of compound **30**.

4-TMR-CP (**31**):

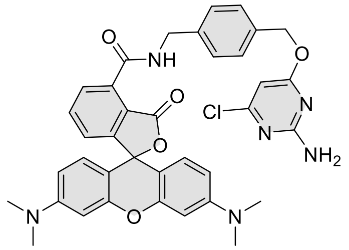

Compound was purified by preparative HPLC (solvent A: H_2O + 10mM NH_4COOH pH = 3.6, solvent B: acetonitrile; temperature 25 °C, gradient A:B - 3 min 80:20 isocratic, 4-20 min 80:20 to 0:100 gradient, 20-25 min 0:100 isocratic, 40 mL/min flow). Fractions containing the product were collected, evaporated and lyophilized from acetonitrile water mixture. The obtained solid was dissolved in 700 µL of d_6-DMSO. The samples from DMSO solution were diluted x100 in PBS +0.1%SDS and concentration was determined spectroscopically with Nanodrop. Determined stock concentration was 5.5 mM which constitutes to 77% yield (2.6 mg).

^{1}H NMR (400 MHz, , d_6-DMSO) δ 9.51 (t, *J* = 5.8 Hz, 1H), 7.85 (dd, *J* = 7.5, 1.0 Hz, 1H), 7.77 (t, *J* = 7.6 Hz, 1H), 7.47 (d, *J* = 8.3 Hz, 2H), 7.42 (d, *J* = 8.4 Hz, 2H), 7.23 (dd, *J* = 7.7, 1.0 Hz, 1H), 7.09 (s, 2H), 6.66 – 6.59 (m, 2H), 6.52 – 6.40 (m, 4H), 6.13 (s, 1H), 5.30 (s, 2H), 4.58 (d, *J* = 5.7 Hz, 2H), 2.93 (s, 12H). ESI-MS, positive mode: m/z = 677.2 $[M+H]^+$. HRMS (ESI) calcd for $C_{37}H_{34}N_6O_5Cl$ $[M+H]^+$ 677.2274, found 677.2262.

LC/MS:

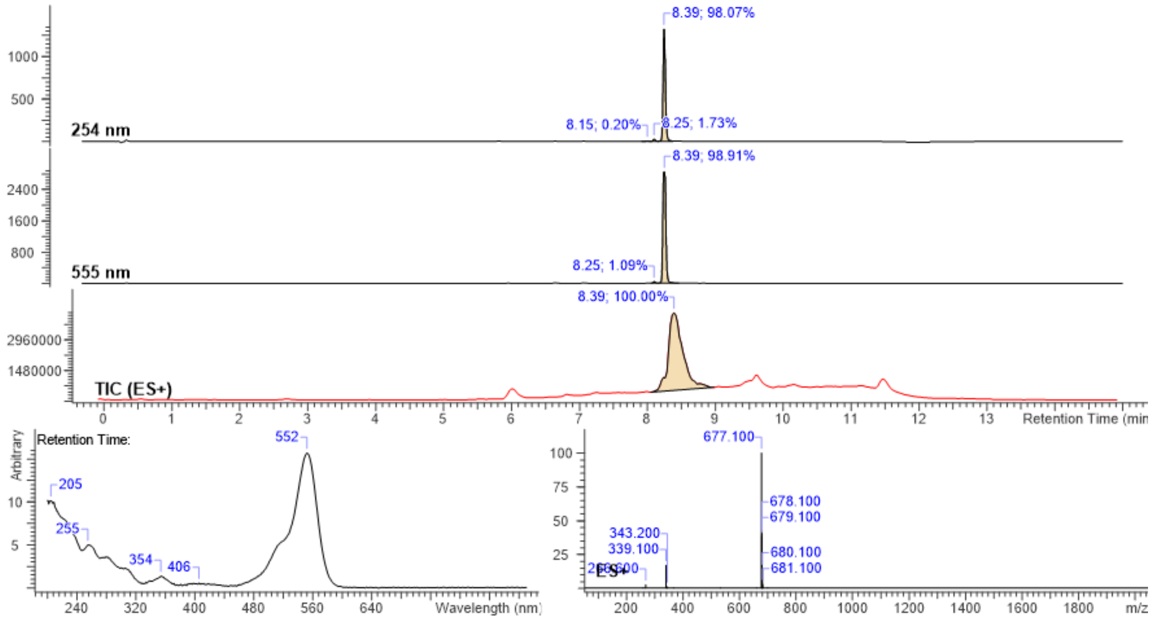

Supplementary Figure 33. LC/MS analysis results of compound **31**.

4-580R-CP (**32**):

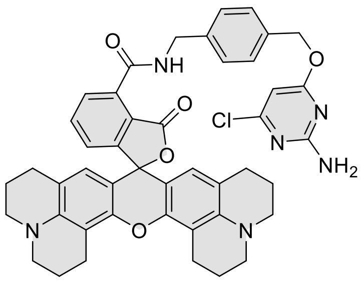

Compound was purified by preparative HPLC (solvent A: H_2O + 10mM NH_4COOH pH = 3.6, solvent B: acetonitrile; temperature 25 °C, gradient A:B - 3 min 80:20 isocratic, 4-20 min 80:20 to 0:100 gradient, 20-25 min 0:100 isocratic, 40 mL/min flow). Fractions containing the product were collected, evaporated and lyophilized from acetonitrile water mixture. The obtained solid was dissolved in 700 µL of d_6-DMSO. The samples from DMSO solution were diluted x100 in PBS +0.1%SDS and concentration was determined spectroscopically with Nanodrop. Determined stock concentration was 3.2 mM which constitutes to 45% yield (1.6 mg). ^{1}H NMR (400 MHz, d_6-DMSO) δ 9.65 (t, *J* = 5.6 Hz, 1H), 7.88 (dd, *J* = 7.5, 0.9 Hz, 1H), 7.76 (t, *J* = 7.6 Hz, 1H), 7.48 (d, *J* = 8.1 Hz, 2H), 7.42 (d, *J* = 8.2 Hz, 2H), 7.27 (dd, *J* = 7.7, 0.8 Hz, 1H), 7.10 (s, 2H), 6.14 (s, 1H), 6.12 (s, 2H), 5.31 (s, 2H), 4.58 (d, *J* = 5.8 Hz, 2H), 3.13 (s, 8H), 2.85 (t, *J* = 6.4 Hz, 4H), 2.49 – 2.35 (m, 4H), 1.99 – 1.91 (m, 4H), 1.78 (p, *J* = 6.1 Hz, 4H). ESI-MS, positive mode: m/z = 781.3 $[M+H]^{+\cdot}$ HRMS (ESI) calcd for $C_{45}H_{42}ClN_6O_5$ $[M+H]^+$ 781.2900, found 781.2896.

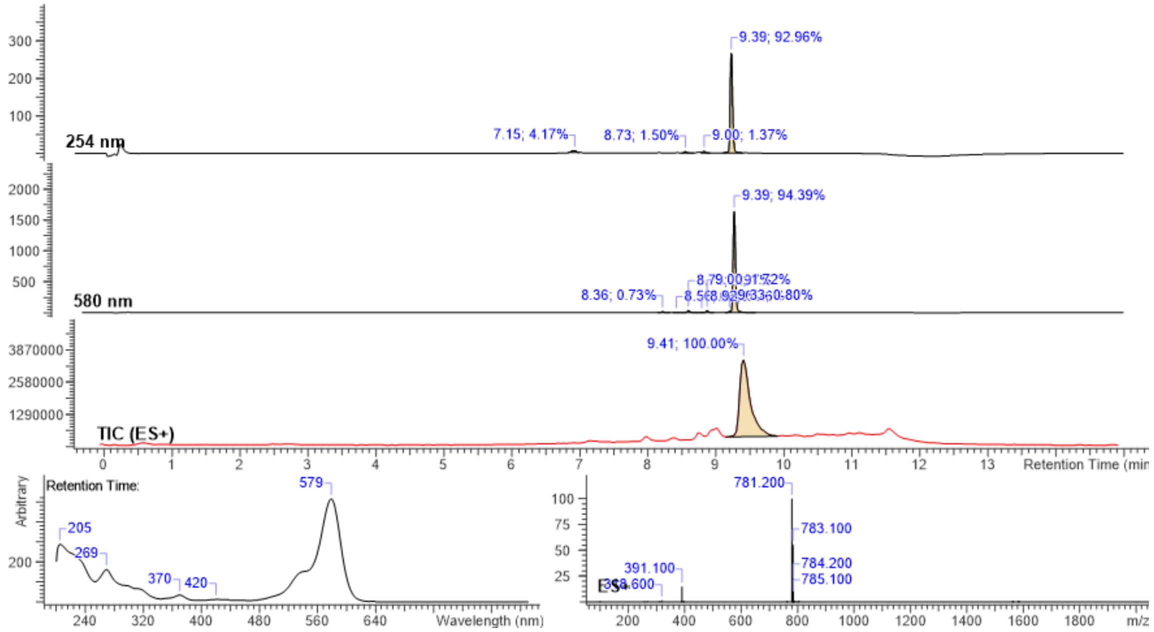

Supplementary Figure 34. LC/MS analysis results of compound **32**.

4-580CP-CP (**33**):

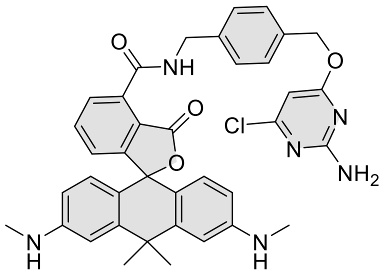

Compound was purified by preparative HPLC (solvent A: H_2O + 10mM NH_4COOH pH = 3.6, solvent B: acetonitrile; temperature 25 °C, gradient A:B - 3 min 80:20 isocratic, 4-20 min 80:20 to 0:100 gradient, 20-25 min 0:100 isocratic, 40 mL/min flow). Fractions containing the product were collected, evaporated and lyophilized from acetonitrile water mixture. The obtained solid was dissolved in 700 µL of d_6-DMSO. The samples from DMSO solution were diluted x100 in PBS +0.1%SDS and concentration was determined spectroscopically with Nanodrop. Determined stock concentration was 5.4 mM which constitutes to 76% yield (2.7 mg).

^{1}H NMR (400 MHz, d_6-DMSO) δ 9.58 (t, *J* = 5.8 Hz, 1H), 7.79 (dd, *J* = 7.5, 1.0 Hz, 1H), 7.70 (t, *J* = 7.6 Hz, 1H), 7.48 (d, *J* = 8.3 Hz, 2H), 7.42 (d, *J* = 8.3 Hz, 2H), 7.09 (s, 2H), 7.06 (dd, *J* = 7.7, 1.0 Hz, 1H), 6.74 (d, *J* = 2.3 Hz, 2H), 6.46 (d, *J* = 8.6 Hz, 2H), 6.36 (dd, *J* = 8.7, 2.3 Hz, 2H), 6.13 (s, 1H), 5.83 (q, *J* = 5.0 Hz, 2H), 5.30 (s, 2H), 4.58 (d, *J* = 5.7 Hz, 2H), 2.68 (d, *J* = 4.9 Hz, 6H), 1.74 (s, 3H), 1.64 (s, 3H). ESI-MS, positive mode: m/z = 675.3 $[M+H]^{+\cdot}$ HRMS (ESI) calcd for $C_{38}H_{36}ClN_6O_4$ $[M+H]^+$ 675.2481, found 675.2468.

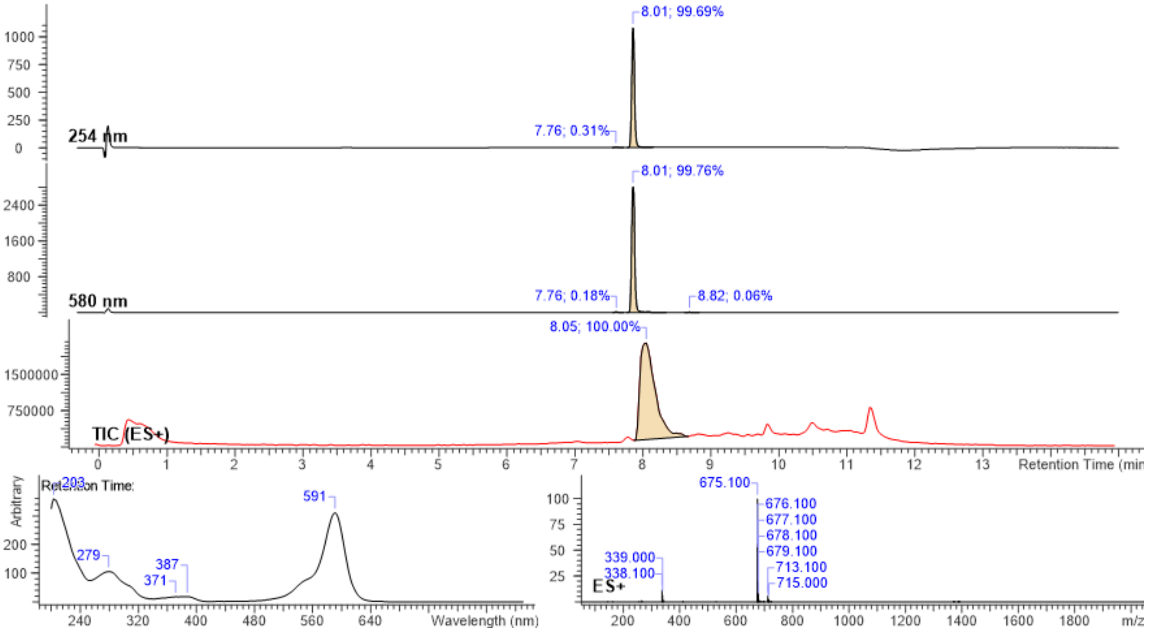

Supplementary Figure 35. LC/MS analysis results of compound **33**.

4-610CP-CP (**34**):

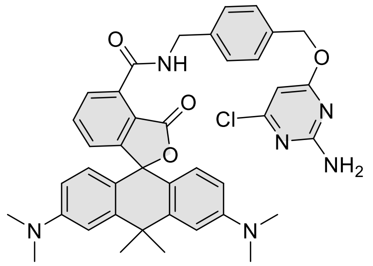

Compound was purified by preparative HPLC (solvent A: H_2O + 10mM NH_4COOH pH = 3.6, solvent B: acetonitrile; temperature 25 °C, gradient A:B - 3 min 80:20 isocratic, 4-20 min 80:20 to 0:100 gradient, 20-25 min 0:100 isocratic, 40 mL/min flow). Fractions containing the product were collected, evaporated and lyophilized from acetonitrile water mixture. The obtained solid was dissolved in 700 µL of d_6-DMSO. The samples from DMSO solution were diluted x100 in PBS +0.1%SDS and concentration was determined spectroscopically with Nanodrop. Determined stock concentration was 4.6 mM which constitutes to 65% yield (2.1 mg)

^{1}H NMR (400 MHz, *d*$_6$-DMSO) δ 9.58 (t, *J* = 5.8 Hz, 1H), 7.80 (dd, *J* = 7.5, 1.0 Hz, 1H), 7.70 (t, *J* = 7.6 Hz, 1H), 7.49 (d, *J* = 8.2 Hz, 2H), 7.44 (d, *J* = 8.3 Hz, 2H), 7.10 (s, 2H), 7.05 (dd, *J* = 7.7, 1.0 Hz, 1H), 6.95 – 6.86 (m, 2H), 6.69 – 6.48 (m, 4H), 6.14 (s, 1H), 5.32 (s, 2H), 4.60 (d, *J* = 5.8 Hz, 2H), 2.94 (s, 12H), 1.82 (s, 3H), 1.72 (s, 3H). ESI-MS, positive mode: m/z = 703.3 $[M+H]^+$. HRMS (ESI) calcd for $C_{40}H_{40}ClN_6O_4$ $[M+H]^+$ 703.2794, found 703.2788.

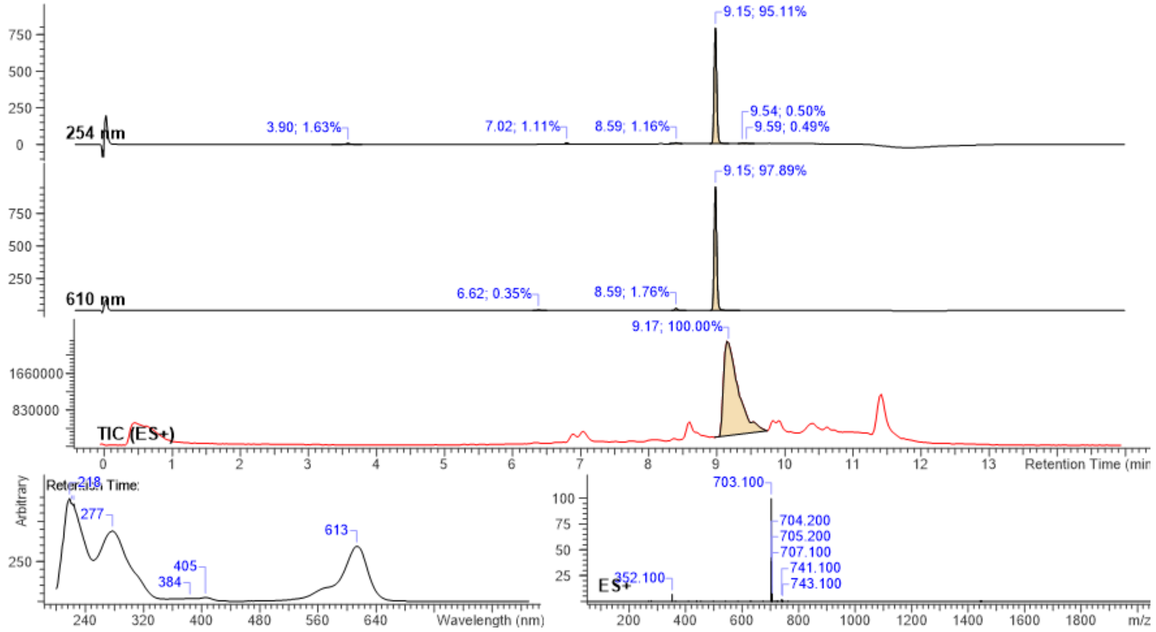

Supplementary Figure 36. LC/MS analysis results of compound **34**.

4-625CP-CP (**35**):

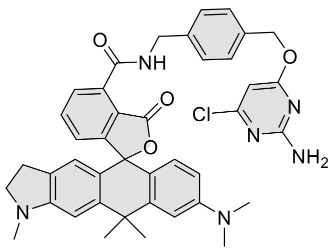

Compound was purified by preparative HPLC (solvent A: H_2O + 10mM NH_4COOH pH = 3.6, solvent B: acetonitrile; temperature 25 °C, gradient A:B - 3 min 80:20 isocratic, 4-20 min 80:20 to 0:100 gradient, 20-25 min 0:100 isocratic, 40 mL/min flow). Fractions containing the product were collected, evaporated and lyophilized from acetonitrile water mixture. The obtained solid was dissolved in 700 µL of d_6-DMSO. The samples from DMSO solution were diluted x100 in PBS +0.1%SDS and concentration was determined spectroscopically with Nanodrop. Determined stock concentration was 3.8 mM which constitutes to 53% yield (1.9 mg)

^{1}H NMR (400 MHz, d_6-DMSO) δ 9.59 (t, *J* = 5.8 Hz, 1H), 7.80 (dd, *J* = 7.5, 1.0 Hz, 1H), 7.70 (t, *J* = 7.6 Hz, 1H), 7.50 (d, *J* = 8.1 Hz, 2H), 7.44 (d, *J* = 8.1 Hz, 2H), 7.10 (s, 2H), 7.05 (dd, *J* = 7.7, 0.9 Hz, 1H), 6.89 (d, *J* = 2.1 Hz, 1H), 6.76 (s, 1H), 6.58 – 6.53 (m, 2H), 6.39 (s, 1H), 6.14 (s, 1H), 5.32 (s, 2H), 4.60 (d, *J* = 5.8 Hz, 2H), 3.28 – 3.20 (m, 2H), 2.93 (s, 6H), 2.79 (s, 3H), 2.77 – 2.66 (m, 2H), 1.79 (s, 3H), 1.71 (s, 3H). ESI-MS, positive mode: m/z = 715.2 $[M+H]^+$. HRMS (ESI) calcd for $C_{41}H_{40}ClN_6O_4$ $[M+H]^+$ 715.2794, found 715.2782.

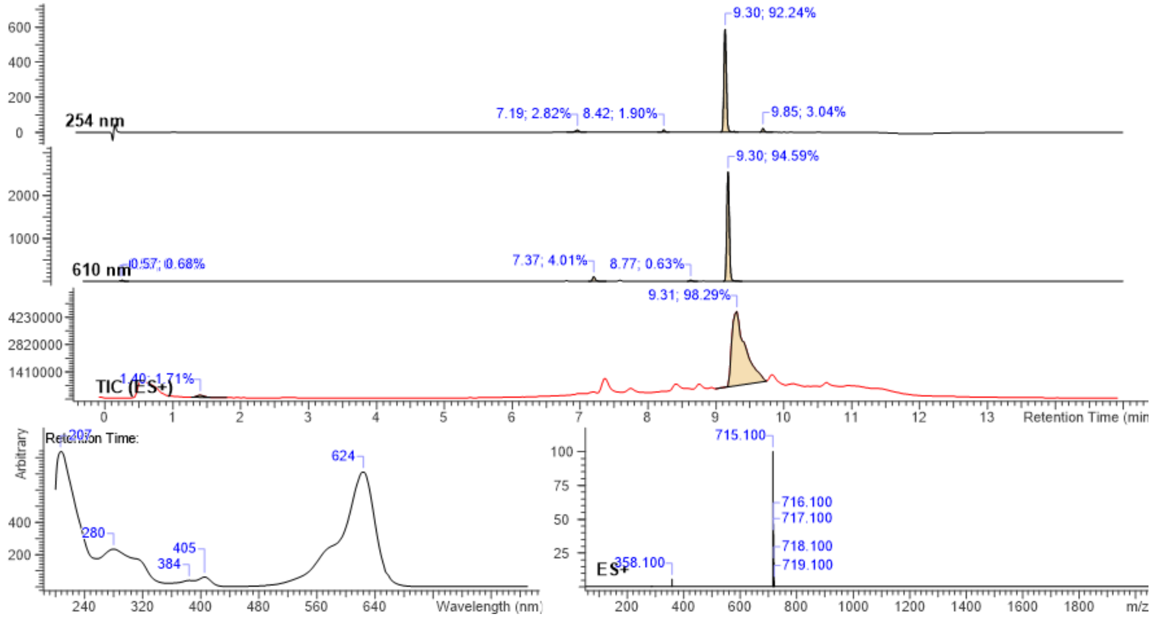

Supplementary Figure 37. LC/MS analysis results of compound **35**.

4-630CP-CP (**36**):

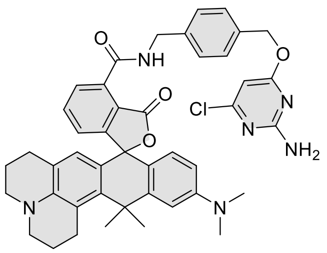

Compound was purified by preparative HPLC (solvent A: H_2O + 10mM NH_4COOH pH = 3.6, solvent B: acetonitrile; temperature 25 °C, gradient A:B - 3 min 80:20 isocratic, 4-20 min 80:20 to 0:100 gradient, 20-25 min 0:100 isocratic , 40 mL/min flow). Fractions containing the product were collected, evaporated and lyophilized from acetonitrile water mixture. The obtained solid was dissolved in 700 µL of d_6-DMSO. The samples from DMSO solution were diluted x100 in PBS +0.1%SDS and concentration was determined spectroscopically with Nanodrop. Determined stock concentration was 3.1 mM which constitutes to 44% yield (1.7 mg)

^{1}H NMR (400 MHz, d_6-DMSO) δ 9.62 (t, J = 5.8 Hz, 1H), 7.79 (dd, J = 7.5, 1.0 Hz, 1H), 7.68 (t, J = 7.6 Hz, 1H), 7.50 (d, J = 8.3 Hz, 2H), 7.44 (d, J = 8.3 Hz, 2H), 7.10 (s, 2H), 7.01 (dd, J = 7.7, 1.0 Hz, 1H), 6.79 (d, J = 2.5 Hz, 1H), 6.56 (dd, J = 8.9, 2.5 Hz, 1H), 6.50 (d, J = 8.9 Hz, 1H), 6.17 (s, 1H), 6.15 (s, 1H), 5.32 (s, 2H), 4.60 (d, J = 5.8 Hz, 2H), 3.20 – 3.12 (m, 4H), 2.98 – 2.93 (m, 2H), 2.92 (s, 6H), 2.47 – 2.36 (m, 2H), 1.95 – 1.89 (m, 2H), 1.89 (s, 3H), 1.83 (s, 3H), 1.79 – 1.72 (m, 2H). ESI-MS, positive mode: m/z = 755.3 $[M+H]^+$. HRMS (ESI) calcd for $C_{44}H_{44}ClN_6O_4$ $[M+H]^+$ 755.3107, found 755.3100.

LC/MS:

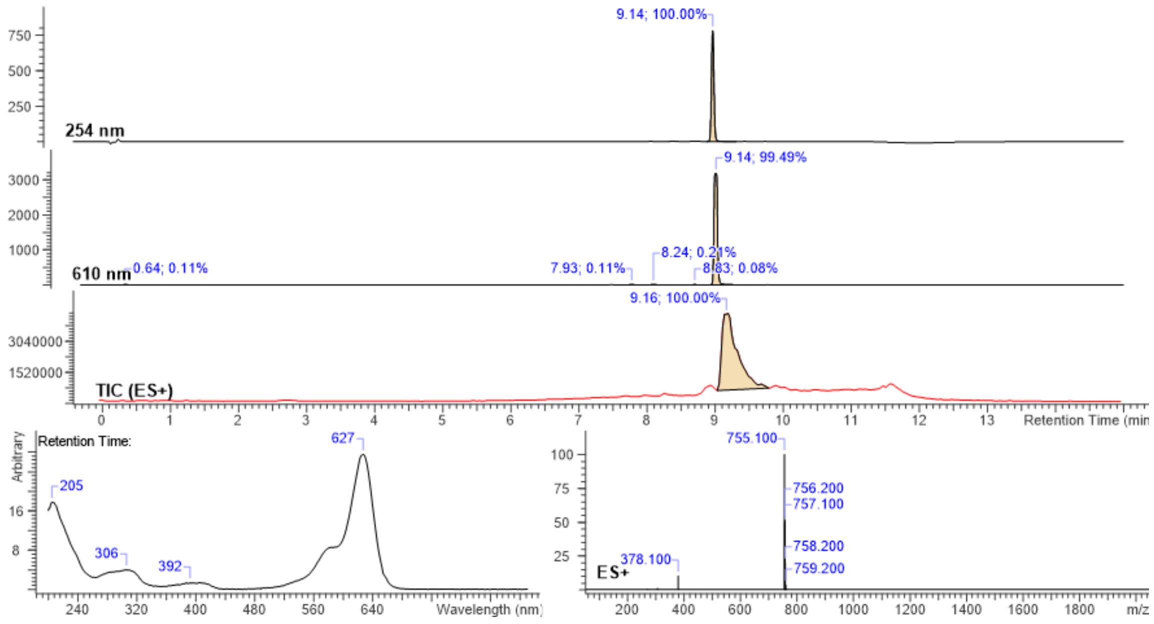

Supplementary Figure 38. LC/MS analysis results of compound **36**.

4-640CP-CP (**37**):

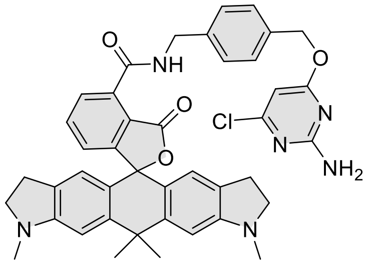

Compound was purified by preparative HPLC (solvent A: H_2O + 10mM NH_4COOH pH = 3.6, solvent B: acetonitrile; temperature 25 °C, gradient A:B - 3 min 80:20 isocratic, 4-20 min 80:20 to 0:100 gradient, 20-25 min 0:100 isocratic , 40 mL/min flow). Fractions containing the product were collected, evaporated and lyophilized from acetonitrile water mixture. The obtained solid was dissolved in 700 µL of d_6-DMSO. The samples from DMSO solution were diluted x100 in PBS +0.1%SDS and concentration was determined spectroscopically with Nanodrop. Determined stock concentration was 4.6 mM which constitutes to 65% yield (2.4 mg).

^{1}H NMR (400 MHz, d_6-DMSO) δ 9.61 (t, J = 5.8 Hz, 1H), 7.79 (dd, J = 7.5, 1.0 Hz, 1H), 7.70 (t, J = 7.6 Hz, 1H), 7.50 (d, J = 8.2 Hz, 2H), 7.44 (d, J = 8.2 Hz, 2H), 7.10 (s, 2H), 7.05 (dd, J = 7.7, 0.9 Hz, 1H), 6.74 (s, 2H), 6.36 (s, 2H), 6.14 (s, 1H), 5.32 (s, 2H), 4.60 (d, J = 5.8 Hz, 2H), 3.27 – 3.18 (m, 4H), 2.78 (s, 6H), 2.75 – 2.60 (m, 4H), 1.76 (s, 3H), 1.69 (s, 3H). ESI-MS, positive mode: m/z = 727.3 $[M+H]^+$. HRMS (ESI) calcd for $C_{42}H_{40}ClN_6O_4$ $[M+H]^+$ 727.2794, found 727.2786.

LC/MS:

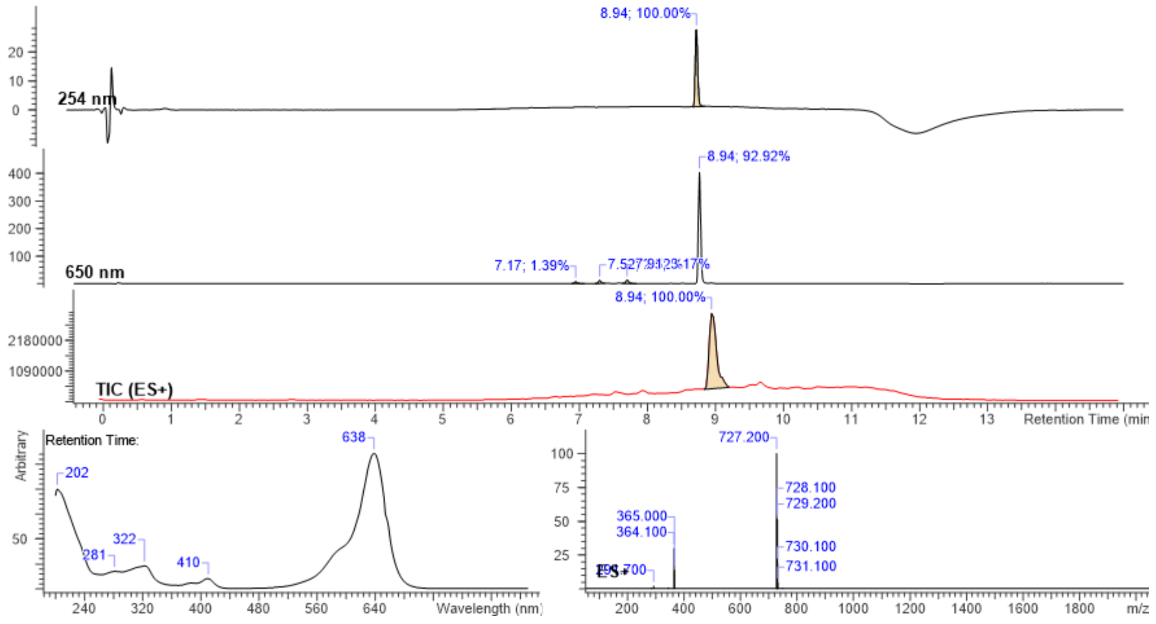

Supplementary Figure 39. LC/MS analysis results of compound **37**.

4-642CP-CP (**38**):

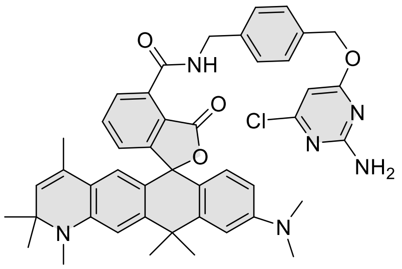

Compound was purified by preparative HPLC (solvent A: H_2O + 10mM NH_4COOH pH = 3.6, solvent B: acetonitrile; temperature 25 °C, gradient A:B - 3 min 30:70 isocratic, 4-20 min 30:70 to 0:100 gradient, 20-25 min 0:100 isocratic, 40 mL/min flow). Fractions containing the product were collected, evaporated and lyophilized from acetonitrile water mixture. The obtained solid was dissolved in 700 µL of d_6-DMSO. The samples from DMSO solution were diluted x100 in PBS +0.1%SDS and concentration was determined spectroscopically with Nanodrop. Determined stock concentration was 5.1 mM which constitutes to 72% yield (2.7 mg).

^{1}H NMR (400 MHz, *d*$_{6}$-DMSO) δ 9.62 (t, *J* = 5.8 Hz, 1H), 7.85 (dd, *J* = 7.5, 1.0 Hz, 1H), 7.74 (t, *J* = 7.6 Hz, 1H), 7.49 (d, *J* = 8.2 Hz, 2H), 7.43 (d, *J* = 8.4 Hz, 2H), 7.15 – 7.07 (m, 3H), 6.91 (d, *J* = 2.3 Hz, 1H), 6.67 (s, 1H), 6.60 – 6.54 (m, 2H), 6.18 (s, 1H), 6.14 (s, 1H), 5.34 – 5.29 (m, 3H), 4.60 (t, *J* = 5.9 Hz, 2H), 2.94 (s, 6H), 2.84 (s, 3H), 1.81 (s, 3H), 1.71 (s, 3H), 1.55 (d, *J* = 1.4 Hz, 3H), 1.26 (d, *J* = 4.4 Hz, 6H). ESI-MS, positive mode: m/z = 769.3 $[M+H]^+$. HRMS (ESI) calcd for $C_{45}H_{46}ClN_6O$ $[M+H]^+$ 769.3264, found 769.3261.

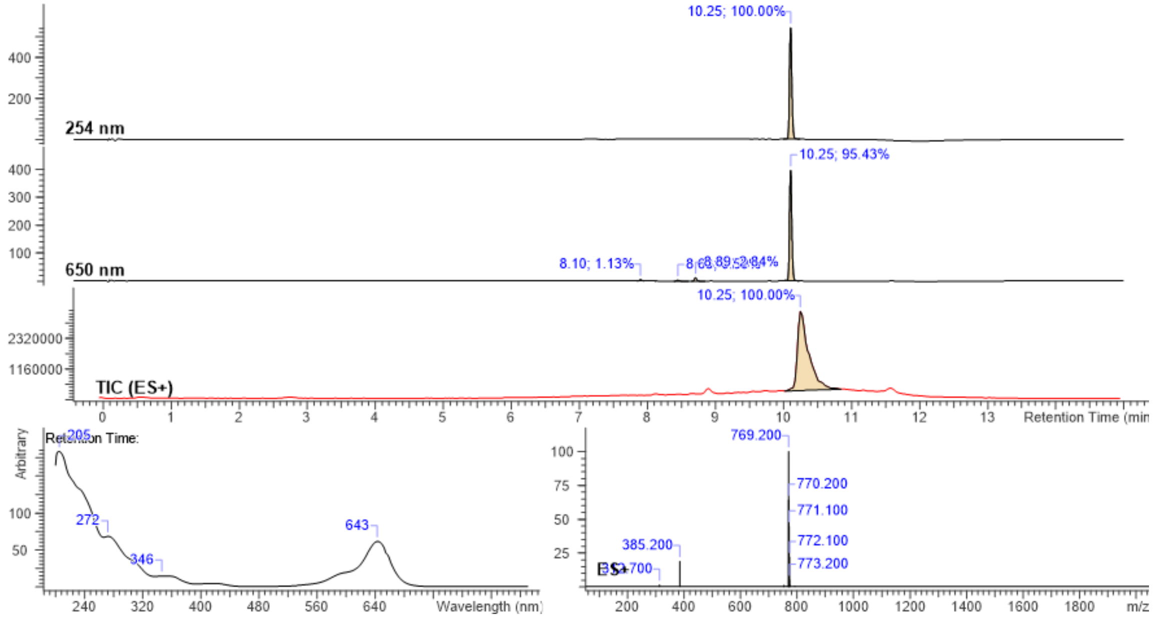

Supplementary Figure 40. LC/MS analysis results of compound **38**.

4-645CP-CP (**39**):

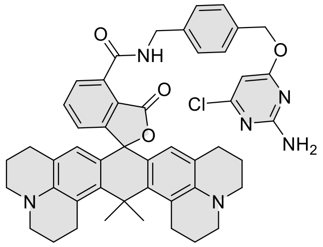

Compound was purified by preparative HPLC (solvent A: H_2O + 10 mM NH_4COOH pH = 3.6, solvent B: acetonitrile; temperature 25 °C, gradient A:B - 3 min 40:60 isocratic, 4-20 min 40:60 to 0:100 gradient, 20-25 min 0:100 isocratic, 40 mL/min flow). Fractions containing the product were collected, evaporated and lyophilized from acetonitrile water mixture. The obtained solid was dissolved in 700 µL of d_6-DMSO. The samples from DMSO solution were diluted x100 in PBS +0.1%SDS and concentration was determined spectroscopically with Nanodrop. Determined stock concentration was 2.9 mM which constitutes to 40% yield (1.6 mg).

^{1}H NMR (400 MHz, d_6-DMSO) δ 9.66 (t, J = 5.8 Hz, 1H), 7.79 (dd, J = 7.5, 1.0 Hz, 1H), 7.66 (d, J = 7.6 Hz, 1H), 7.50 (d, J = 8.3 Hz, 2H), 7.44 (d, J = 8.2 Hz, 2H), 7.10 (s, 2H), 6.98 (dd, J = 7.7, 1.0 Hz, 1H), 6.14 (s, 1H), 6.06 (s, 2H), 5.32 (s, 2H), 4.60 (d, J = 5.8 Hz, 2H), 3.19 – 3.10 (m, 8H), 2.84 (dd, J = 7.3, 4.2 Hz, 4H), 2.47 – 2.34 (m, 4H), 1.98 (s, 3H), 1.92 (s, 3H), 1.89 (dd, J = 6.8, 4.9 Hz, 4H), 1.74 (tq, J = 9.4, 5.9, 5.5 Hz, 5H). ESI-MS, positive mode: m/z = 807.3 $[M+H]^+$. HRMS (ESI) calcd for $C_{48}H_{48}ClN_6O_4$ $[M+H]^+$ 807.3420, found 807.3399.

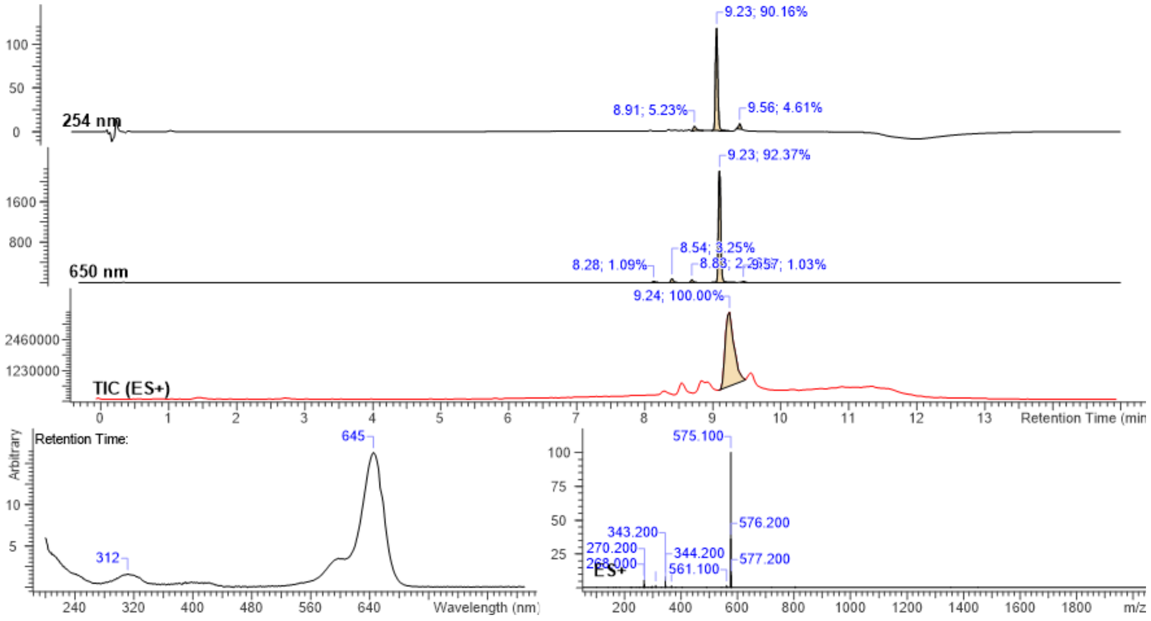

Supplementary Figure 41. LC/MS analysis results of compound **39**.

4-SiR-CP (**40**):

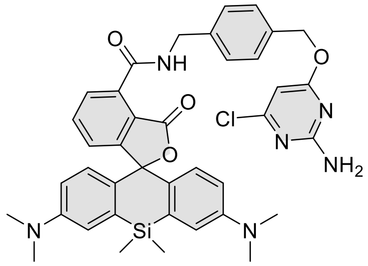

Compound was purified by preparative HPLC (solvent A: H_2O + 10 mM NH_4COOH pH = 3.6, solvent B: acetonitrile; temperature 25 °C, gradient A:B - 3 min 40:60 isocratic, 4-20 min 40:60 to 0:100 gradient, 20-25 min 0:100 isocratic, 40 mL/min flow). Fractions containing the product were collected, evaporated and lyophilized from acetonitrile water mixture. The obtained solid was dissolved in 700 µL of d_6-DMSO. The samples from DMSO solution were diluted x100 in PBS +0.1%SDS and concentration was determined spectroscopically with Nanodrop. Determined stock concentration was 4.3 mM which constitutes to 61% yield (2.2 mg).

^{1}H NMR (400 MHz, d_6-DMSO) δ 9.50 – 9.43 (m, 1H), 7.83 – 7.75 (m, 2H), 7.47 (d, J = 8.3 Hz, 2H), 7.42 (d, J = 8.5 Hz, 2H), 7.28 (dd, J = 5.6, 3.1 Hz, 1H), 7.09 (s, 4H), 6.73 (q, J = 8.6 Hz, 4H), 6.14 (s, 1H), 5.31 (s, 2H), 4.56 (d, J = 5.8 Hz, 2H), 2.95 (s, 12H), 0.63 (s, 3H), 0.53 (s, 3H). ESI-MS, positive mode: m/z = 719.3 $[M+H]^+$. HRMS (ESI) calcd for $C_{39}H_{40}ClN_6O_4Si$ $[M+H]^+$ 719.2563, found 719.2543.

LC/MS:

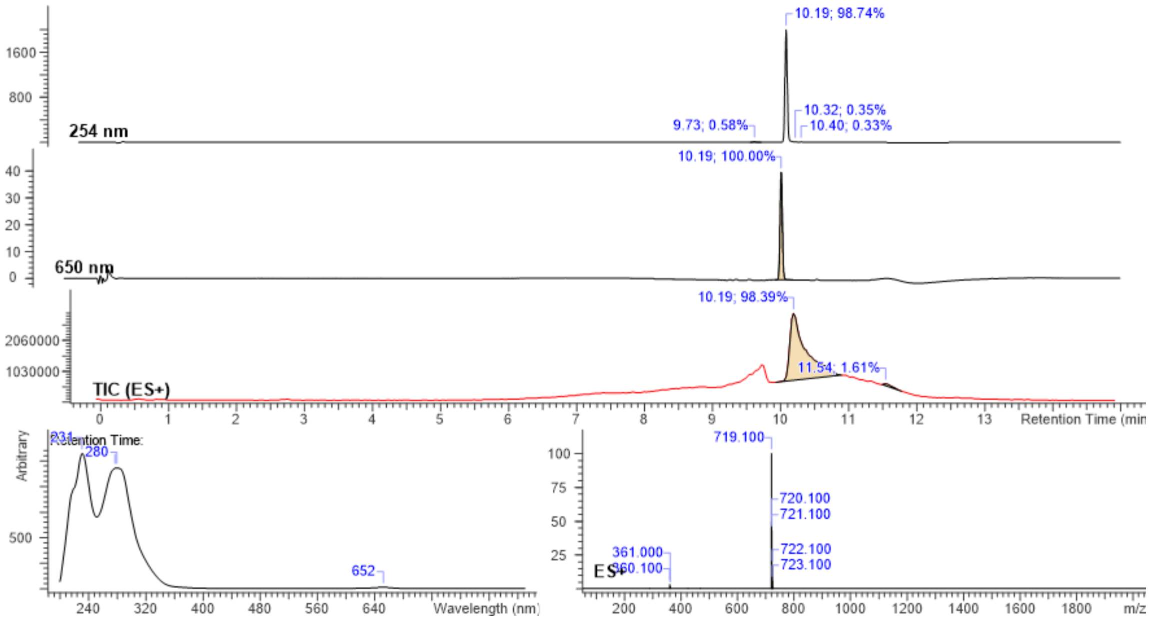

Supplementary Figure 42. LC/MS analysis results of compound **40**.

4-665SiR-CP (**41**):

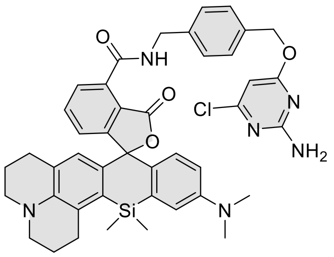

Compound was purified by preparative HPLC (solvent A: H_2O + 10 mM NH_4COOH pH = 3.6, solvent B: acetonitrile; temperature 25 °C, gradient A:B - 3 min 30:70 isocratic, 4-20 min 30:70 to 0:100 gradient, 20-25 min 0:100 isocratic, 40 mL/min flow). Fractions containing the product were collected, evaporated and lyophilized from acetonitrile water mixture. The obtained solid was dissolved in 700 µL of d_6-DMSO. The samples from DMSO solution were diluted x100 in PBS +0.1%SDS and concentration was determined spectroscopically with Nanodrop. Determined stock concentration was 4.3 mM which constitutes to 61% yield (2.2 mg).

^{1}H NMR (400 MHz, d_6-DMSO) δ 9.51 (t, J = 5.8 Hz, 1H), 7.74 – 7.67 (m, 2H), 7.46 (d, J = 8.3 Hz, 2H), 7.41 (d, J = 8.3 Hz, 2H), 7.12 (dd, J = 7.2, 1.5 Hz, 1H), 7.09 (s, 2H), 6.92 (d, J = 2.7 Hz, 1H), 6.68 (d, J = 9.0 Hz, 1H), 6.63 (dd, J = 9.1, 2.7 Hz, 1H), 6.33 (s, 1H), 6.13 (s, 1H), 5.30 (s, 2H), 4.56 (d, J = 5.8 Hz, 2H), 3.15 (t, J = 5.8 Hz, 2H), 3.10 (t, J = 5.5 Hz, 3H), 2.89 (s, 8H), 2.46 – 2.38 (m, 2H), 2.01 – 1.86 (m, 4H), 1.74 (q, J = 6.0 Hz, 4H), 0.65 (s, 3H), 0.54 (s, 3H). ESI-MS, positive mode: m/z = 771.2 $[M+H]^+$. HRMS (ESI) calcd for $C_{43}H_{44}ClN_6O_4Si$ $[M+H]^+$ 771.2876, found 771.2865.

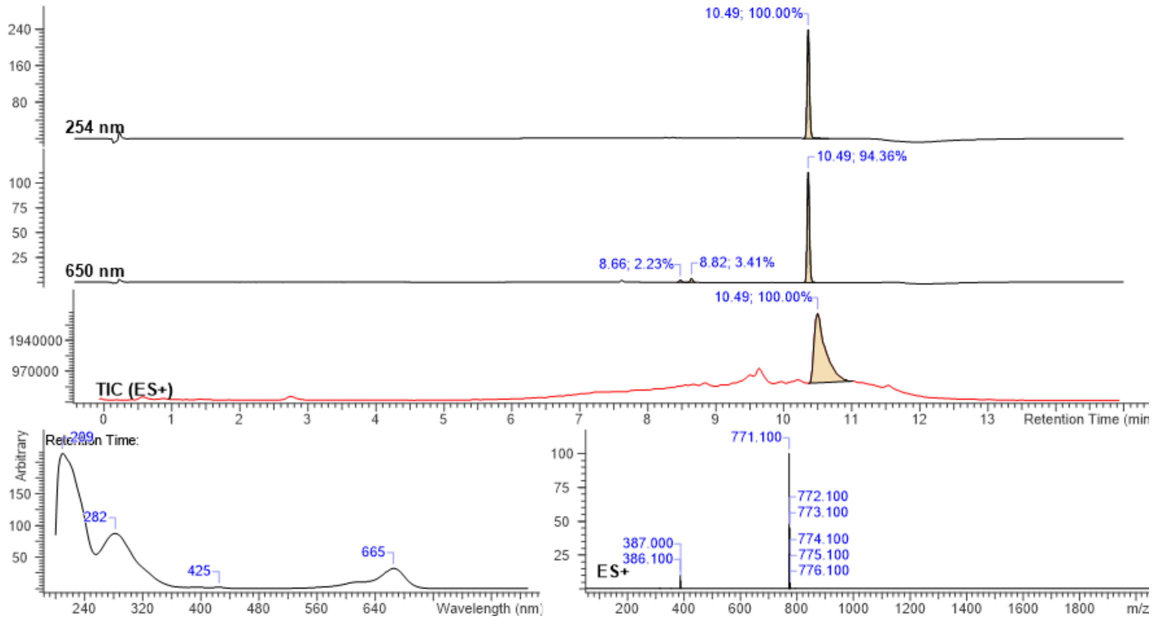

Supplementary Figure 43. LC/MS analysis results of compound **41**.

4-670SiR-CP (**42**):

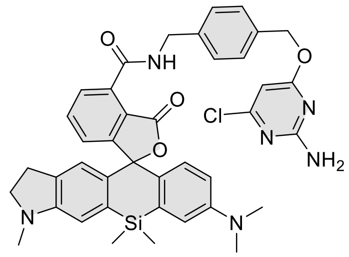

Compound was purified by preparative HPLC (solvent A: H_2O + 10 mM NH_4COOH pH = 3.6, solvent B: acetonitrile; temperature 25 °C, gradient A:B - 3 min 30:70 isocratic, 4-20 min 30:70 to 0:100 gradient, 20-25 min 0:100 isocratic, 40 mL/min flow). Fractions containing the product were collected, evaporated and lyophilized from acetonitrile water mixture. The obtained solid was dissolved in 700 µL of d_6-DMSO. The samples from DMSO solution were diluted x100 in PBS +0.1%SDS and concentration was determined spectroscopically with Nanodrop. Determined stock concentration was 4.7 mM which constitutes to 66% yield (2.3 mg).

^{1}H NMR (400 MHz, d_6-DMSO) δ 9.49 (t, *J* = 5.8 Hz, 1H), 7.78 – 7.70 (m, 2H), 7.46 (d, *J* = 8.3 Hz, 2H), 7.41 (d, *J* = 8.2 Hz, 2H), 7.20 (dd, *J* = 6.6, 2.1 Hz, 1H), 7.09 (s, 2H), 6.96 (d, *J* = 2.8 Hz, 1H), 6.79 (s, 1H), 6.70 (d, *J* = 9.0 Hz, 1H), 6.62 (dd, *J* = 9.0, 2.8 Hz, 1H), 6.58 (s, 1H), 6.13 (s, 1H), 5.30 (s, 2H), 4.56 (d, *J* = 5.8 Hz, 2H), 3.24 – 3.15 (m, 2H), 2.89 (s, 6H), 2.81 – 2.65 (m, 5H), 0.58 (s, 3H), 0.49 (s, 3H). ESI-MS, positive mode: m/z = 731.3 $[M+H]^+$. HRMS (ESI) calcd for $C_{40}H_{40}ClN_6O_4Si$ $[M+H]^+$ 731.2563, found 731.2552.

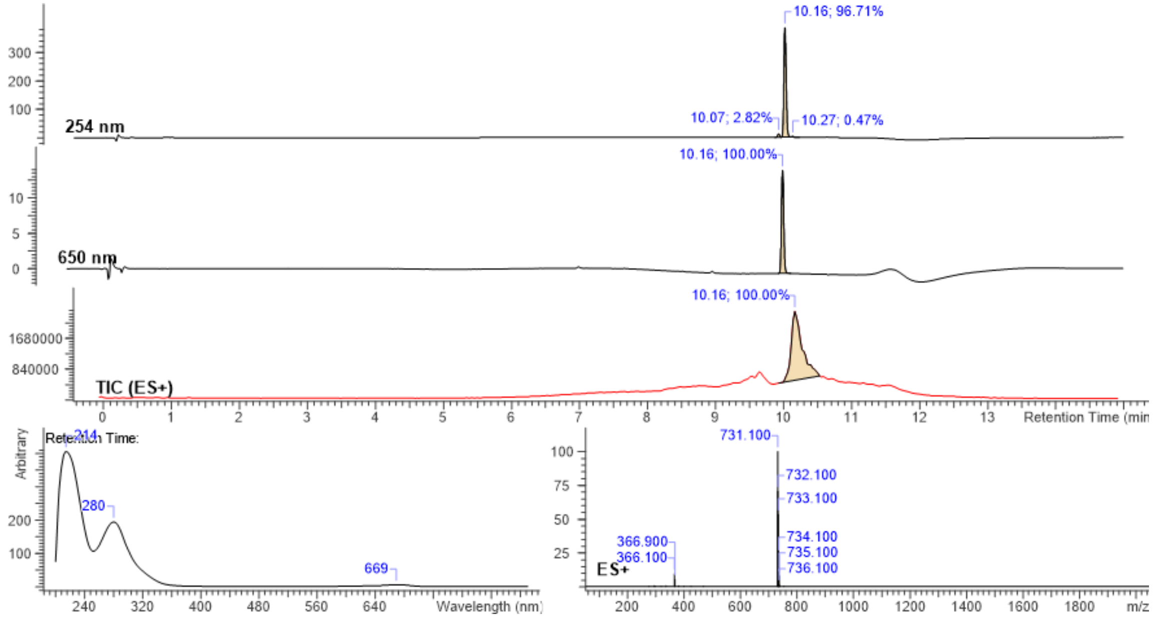

Supplementary Figure 44. LC/MS analysis results of compound **42**.

4-685SiR-CP (43):

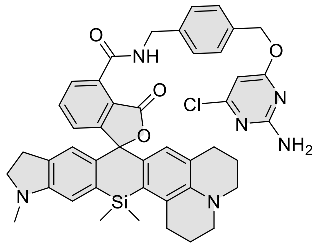

Compound was purified by preparative HPLC (solvent A: H_2O + 10 mM NH_4COOH pH = 3.6, solvent B: acetonitrile; temperature 25 °C, gradient A:B - 3 min 40:60 isocratic, 4-20 min 40:60 to 0:100 gradient, 20-25 min 0:100 isocratic, 40 mL/min flow). Fractions containing the product were collected, evaporated and lyophilized from acetonitrile water mixture. The obtained solid was dissolved in 700 µL of d_6-DMSO. The samples from DMSO solution were diluted x100 in PBS +0.1%SDS and concentration was determined spectroscopically with Nanodrop. Determined stock concentration was 3.5 mM which constitutes to 49% yield (1.9 mg).

^{1}H NMR (400 MHz, d_6-DMSO) δ 9.52 (t, *J* = 5.8 Hz, 1H), 7.72 (dd, *J* = 7.5, 1.3 Hz, 1H), 7.68 (t, *J* = 7.5 Hz, 1H), 7.47 (d, *J* = 8.2 Hz, 2H), 7.42 (d, *J* = 8.2 Hz, 2H), 7.12 – 7.05 (m, 3H), 6.73 (s, 1H), 6.55 (s, 1H), 6.32 (s, 1H), 6.13 (s, 1H), 5.30 (s, 2H), 4.56 (d, *J* = 5.8 Hz, 2H), 3.23 – 3.08 (m, 6H), 2.89 (t, *J* = 6.4 Hz, 2H), 2.84 – 2.58 (m, 7H), 2.47 – 2.36 (m, 2H), 1.94 (p, *J* = 6.1 Hz, 3H), 1.77 – 1.70 (m, 2H), 0.63 (s, 3H), 0.52 (s, 3H). ESI-MS, positive mode: m/z = 783.3 $[M+H]^+$. HRMS (ESI) calcd for $C_{44}H_{44}ClN_6O_4Si$ $[M+H]^+$ 783.2876, found 783.2866.

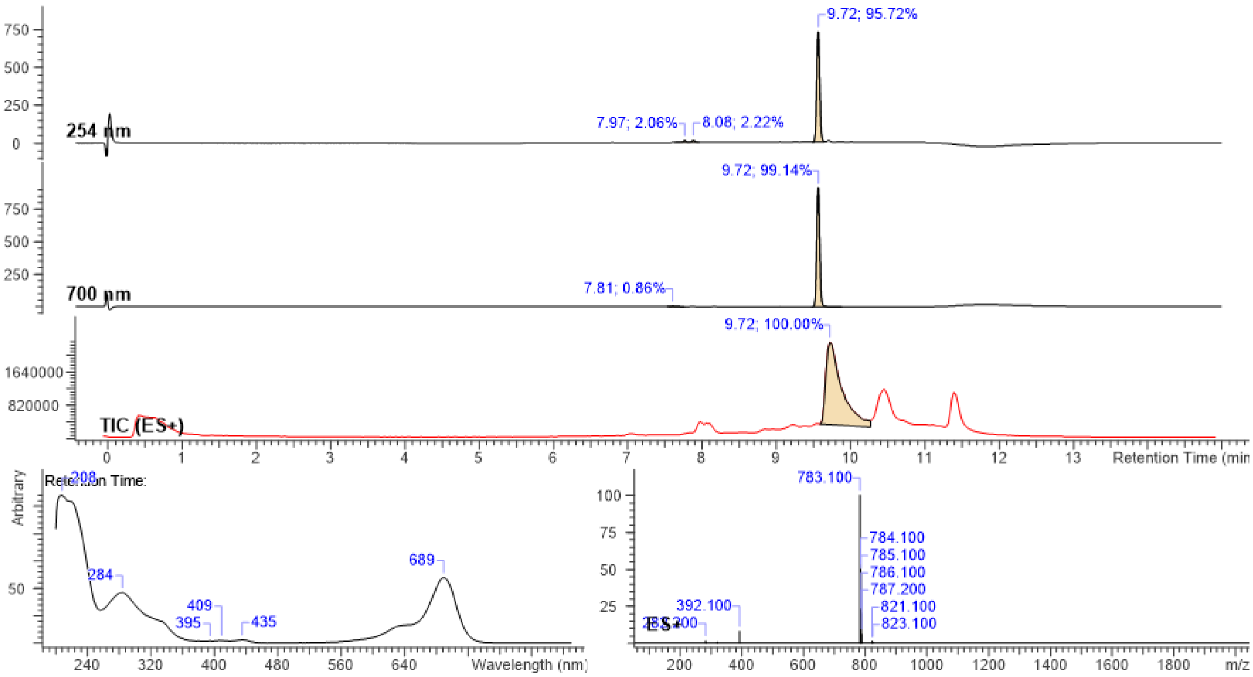

Supplementary Figure 45. LC/MS analysis results of compound **43**.

4-690SiR-CP (**44**):

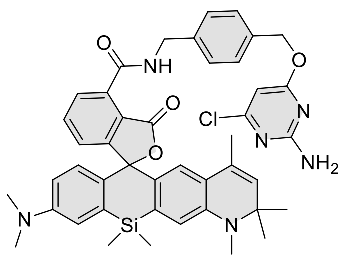

Compound was purified by preparative HPLC (solvent A: H_2O + 10 mM NH_4COOH pH = 3.6, solvent B: acetonitrile; temperature 25 °C, gradient A:B - 3 min 30:70 isocratic, 4-20 min 30:70 to 0:100 gradient, 20-25 min 0:100 isocratic, 40 mL/min flow). Fractions containing the product were collected, evaporated and lyophilized from acetonitrile water mixture. The obtained solid was dissolved in 700 µL of d_6-DMSO. The samples from DMSO solution were diluted x100 in PBS +0.1%SDS and concentration was determined spectroscopically with Nanodrop. Determined stock concentration was 2.9 mM which constitutes to 41% yield (1.6 mg).

^{1}H NMR (400 MHz, d_6-DMSO) δ 9.48 (t, *J* = 5.7 Hz, 1H), 7.86 – 7.79 (m, 2H), 7.46 (d, *J* = 8.4 Hz, 2H), 7.41 (d, *J* = 8.5 Hz, 2H), 7.39 – 7.35 (m, 1H), 7.10 (s, 2H), 7.01 (d, *J* = 2.8 Hz, 1H), 6.76 (s, 1H), 6.70 (d, *J* = 8.9 Hz, 1H), 6.61 (dd, *J* = 9.0, 2.8 Hz, 1H), 6.41 (s, 1H), 6.14 (s, 1H), 5.34 (d, *J* = 1.5 Hz, 1H), 5.30 (s, 2H), 4.61 – 4.51 (m, 2H), 2.91 (s, 6H), 2.81 (s, 3H), 1.57 (d, *J* = 1.4 Hz, 3H), 1.24 (d, *J* = 4.1 Hz, 7H), 0.63 (s, 3H), 0.51 (s, 3H). ESI-MS, positive mode: m/z = 785.3 $[M+H]^+$. HRMS (ESI) calcd for $C_{44}H_{46}ClN_6O_4Si$ $[M+H]^+$ 785.3033, found 785.3025.

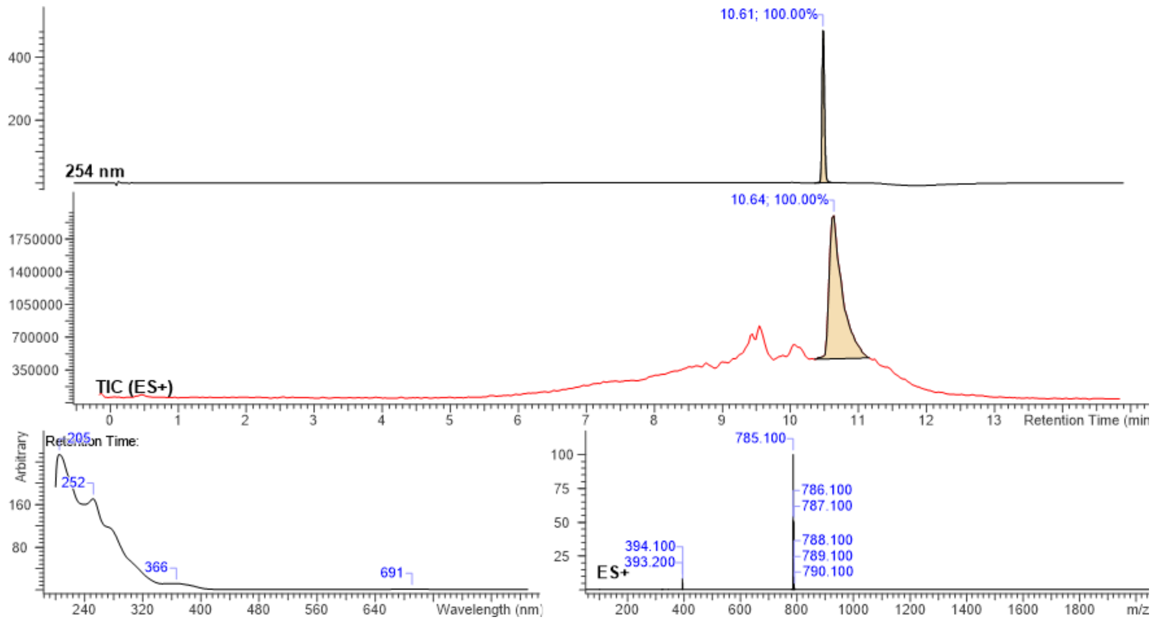

Supplementary Figure 46. LC/MS analysis results of compound **44**.

4-700SiR-CP (**45**):

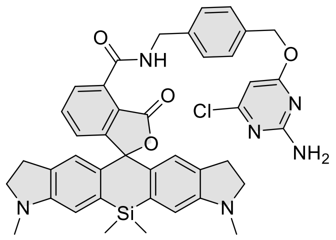

Compound was purified by preparative HPLC (solvent A: H_2O + 10 mM NH_4COOH pH = 3.6, solvent B: acetonitrile; temperature 25 °C, gradient A:B - 3 min 30:70 isocratic, 4-20 min 30:70 to 0:100 gradient, 20-25 min 0:100 isocratic, 40 mL/min flow). Fractions containing the product were collected, evaporated and lyophilized from acetonitrile water mixture. The obtained solid was dissolved in 700 µL of d_6-DMSO. The samples from DMSO solution were diluted x100 in PBS +0.1%SDS and concentration was determined spectroscopically with Nanodrop. Determined stock concentration was 3.3 mM which constitutes to 46% yield (1.7 mg).

^{1}H NMR (400 MHz, d_6-DMSO) δ 9.52 (t, J = 5.8 Hz, 1H), 7.78 – 7.71 (m, 2H), 7.49 (d, J = 8.2 Hz, 2H), 7.43 (d, J = 8.2 Hz, 2H), 7.17 (dd, J = 7.1, 1.6 Hz, 1H), 7.10 (s, 2H), 6.78 (s, 2H), 6.59 (s, 2H), 6.14 (s, 1H), 5.32 (s, 2H), 4.58 (d, J = 5.8 Hz, 2H), 3.25 – 3.18 (m, 4H), 2.76 (s, 10H), 0.57 (s, 3H), 0.49 (s, 3H). ESI-MS, positive mode: m/z = 743.3 $[M+H]^+$. HRMS (ESI) calcd for $C_{41}H_{40}ClN_6O_4Si$ $[M+H]^+$ 743.2563, found 743.2551.

LC/MS:

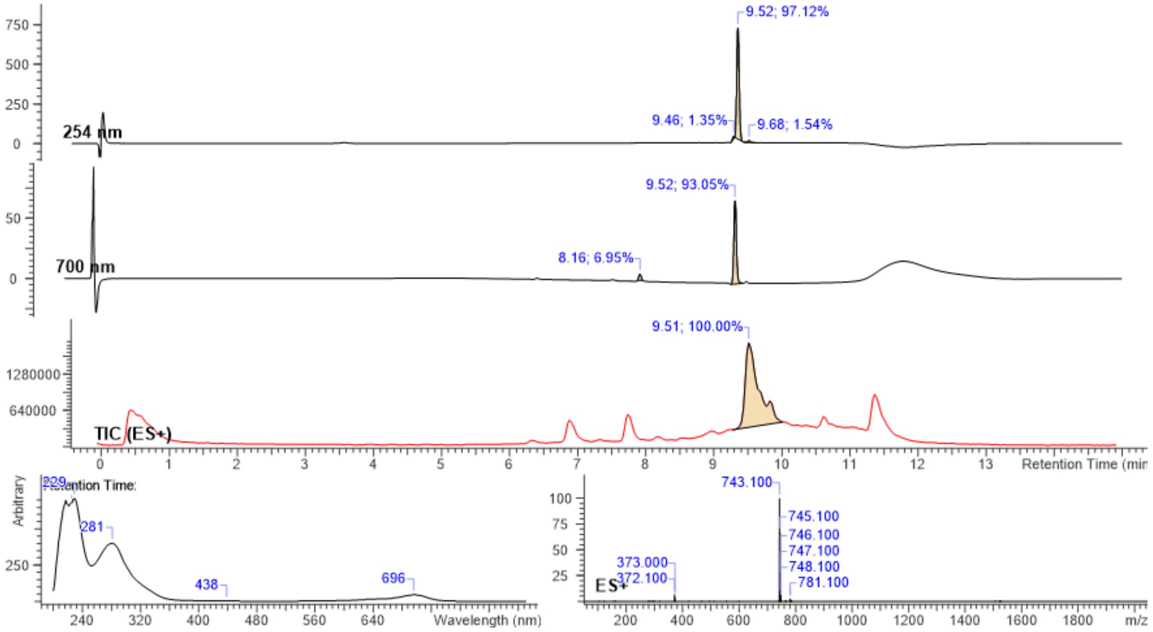

Supplementary Figure 47. LC/MS analysis results of compound **45**.

4-720SiR-CP (**46**):

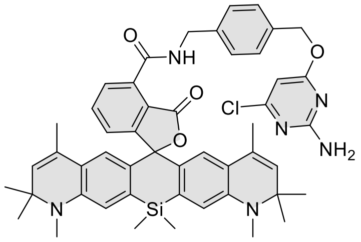

Compound was purified by preparative HPLC (solvent A: H_2O + 10 mM NH_4COOH pH = 3.6, solvent B: acetonitrile; temperature 25 °C, gradient A:B - 3 min 30:70 isocratic, 4-20 min 30:70 to 0:100 gradient, 20-25 min 0:100 isocratic, 40 mL/min flow). Fractions containing the product were collected, evaporated and lyophilized from acetonitrile water mixture. The obtained solid was dissolved in 700 µL of d_6-DMSO. The samples from DMSO solution were diluted x100 in PBS +0.1%SDS and concentration was determined spectroscopically with Nanodrop. Determined stock concentration was 4.9 mM which constitutes to 69% yield (2.9 mg).

^{1}H NMR (400 MHz, *d*$_6$-DMSO) δ 9.48 (t, *J* = 5.8 Hz, 1H), 7.88 – 7.83 (m, 2H), 7.48 (t, *J* = 4.3 Hz, 1H), 7.45 (d, *J* = 8.5 Hz, 2H), 7.40 (d, *J* = 8.4 Hz, 2H), 7.09 (s, 2H), 6.77 (s, 2H), 6.40 (s, 2H), 6.13 (s, 1H), 5.33 (d, *J* = 1.5 Hz, 2H), 5.30 (s, 2H), 4.55 (d, *J* = 5.8 Hz, 2H), 2.81 (s, 6H), 1.56 (d, *J* = 1.4 Hz, 6H), 1.24 (d, *J* = 2.4 Hz, 12H), 0.63 (s, 3H), 0.50 (s, 3H). ESI-MS, positive mode: m/z = 851.3 $[M+H]^+$. HRMS (ESI) calcd for $C_{49}H_{52}ClN_6O_4Si$ $[M+H]^+$ 851.3502, found 851.3495.

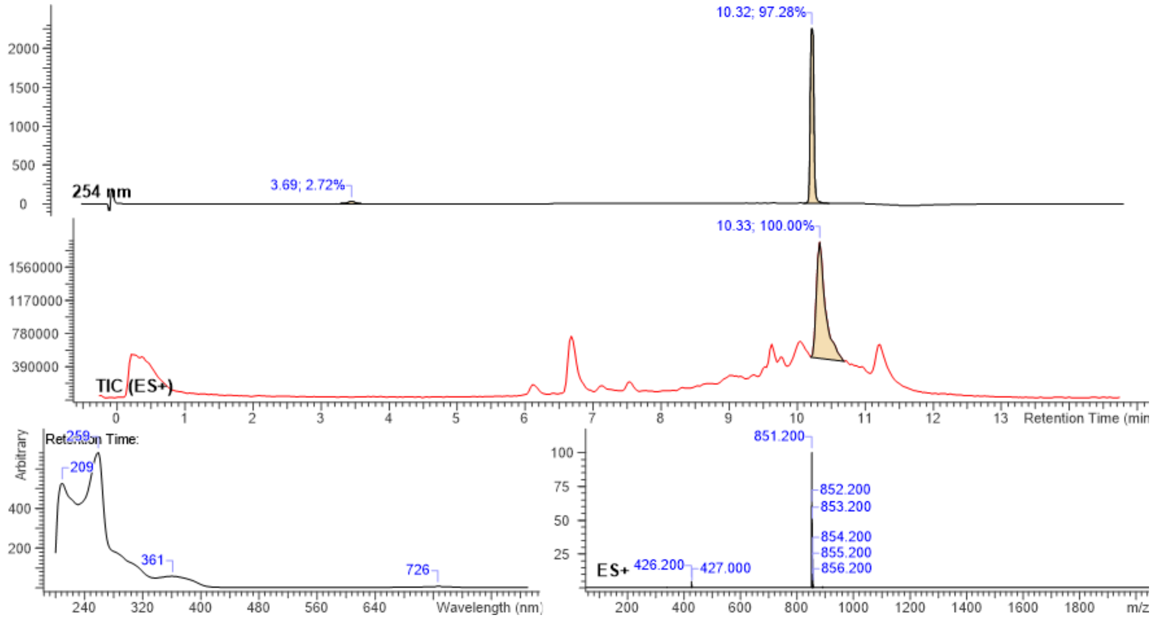

Supplementary Figure 48. LC/MS analysis results of compound **46**.

General procedure for the synthesis of compounds **47**-**67**:

Into a solution of corresponding rhodamine dye (5 µmol, 1 eq; **2-17** and **22-26**) and DIPEA (5 µL) in DMSO (100 µL) a solution of HATU (6.5 µmol, 1.3 eq) in DMSO (100 µL) was added and the mixture was mixed for 1 min. Then a solution of **CTX-C8-NH_2**[25] (7.5 µmol ,1.5 eq) in DMSO (100 µL) was added at once. Reactions were usually over after 30 min and their course was monitored by LC/MS analysis. Once reaction was finished it was quenched with 20 µL of formic acid and diluted with water and acetonitrile to 2 mL volume and was further purified by the means of preparative HPLC (preparative column: Agilent 5 Prep-C18, 5 µm, 100 x 50 mm).

4-CFL-CTX (**47**):

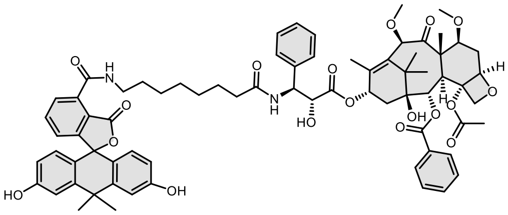

Compound was purified by preparative HPLC (solvent A: H_2O +0.2% HCOOH, solvent B: acetonitrile; temperature 25 °C, gradient A:B - 3 min 70:30 isocratic, 4-20 min 70:30 to 0:100 gradient, 20-25 min 0:100 isocratic, 40 mL/min flow). Fractions containing the product were collected, evaporated and lyophilized from acetonitrile water mixture to obtain product in 38% yield (2.4 mg).

^{1}H NMR (400 MHz, d_6-DMSO) δ 9.72 (br s, 2H), 9.01 (t, *J* = 5.6 Hz, 1H), 8.39 (d, *J* = 9.1 Hz, 1H), 8.02 – 7.96 (m, 2H), 7.76 (dd, *J* = 7.5, 1.0 Hz, 1H), 7.71 (d, *J* = 7.6 Hz, 1H), 7.69 – 7.65 (m, 1H), 7.59 (t, *J* = 7.5 Hz, 2H), 7.41 – 7.30 (m, 4H), 7.21 (t, *J* = 7.1 Hz, 1H), 7.08 – 7.06 (m, 2H), 6.65 – 6.53 (m, 4H), 5.96 (dd, *J* = 8.5, 4.2 Hz, 2H), 5.39 (d, *J* = 7.2 Hz, 1H), 5.29 (dd, *J* = 9.0, 5.8 Hz, 1H), 4.96 (dd, *J* = 9.9, 2.0 Hz, 1H), 4.71 (s, 1H), 4.65 (s, 1H), 4.43 (t, *J* = 6.2 Hz, 1H), 4.02 (s, 2H), 3.76 (dd, *J* = 10.7, 6.5 Hz, 1H), 3.63 (d, *J* = 7.2 Hz, 1H), 3.38 – 3.35 (m, overlapped with water peak, 2H), 3.30 (s, overlapped with water peak, 3H), 3.21 (s, 3H), 2.68 – 2.63 (m, 1H), 2.25 (s, 3H), 2.18 (t, *J* = 7.3 Hz, 2H), 2.01 – 1.78 (m, 6H), 1.73 (s, 3H), 1.64 (s, 3H), 1.61 – 1.41 (m, 8H), 1.31 (m, 6H), 1.03 (s, 3H), 0.97 (s, 3H). ESI-MS, positive mode: m/z = 1283.5 $[M+Na]^+$. HRMS (ESI) calcd for $C_{72}H_{80}N_2O_{18}Na$ $[M+Na]^+$ 1283.5298, found 1283.5292.

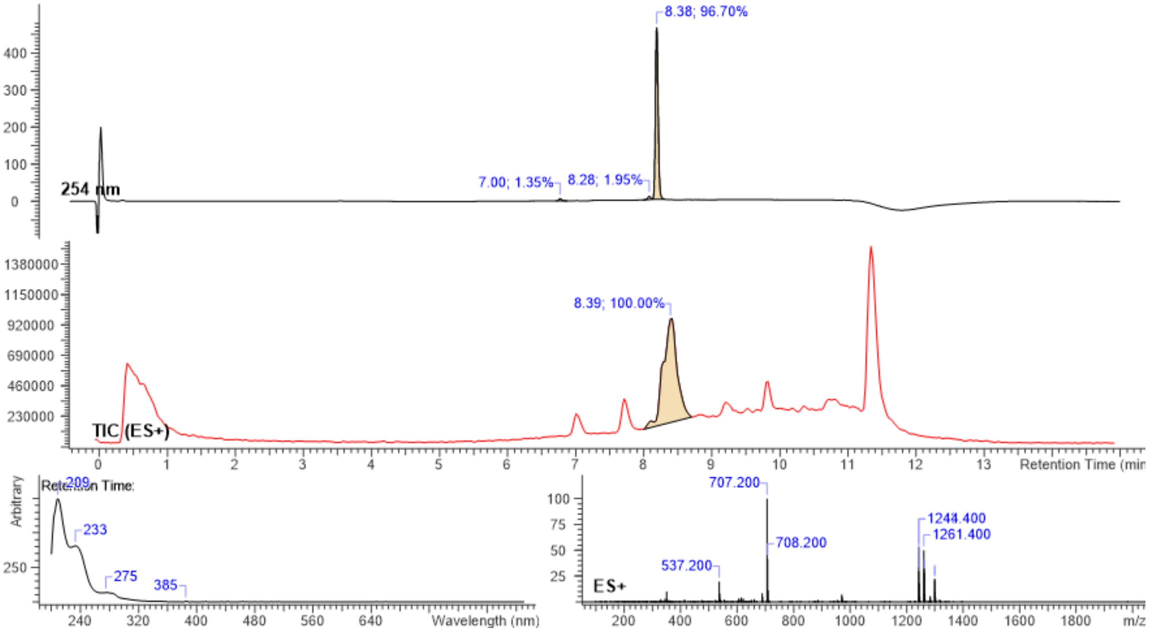

Supplementary Figure 49. LC/MS analysis results of compound **47**.

4-505R-CTX (**48**):

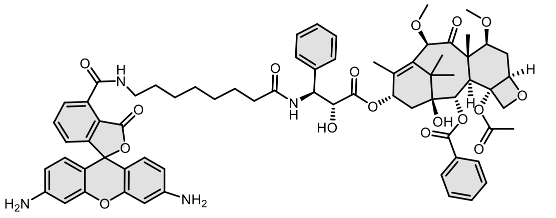

Compound was purified by preparative HPLC (solvent A: 10mM NH_4COOH pH = 3.6, solvent B: acetonitrile; temperature 25 °C, gradient A:B - 3 min 70:30 isocratic, 4-20 min 70:30 to 0:100 gradient, 20-25 min 0:100 isocratic, 40 mL/min flow). Fractions containing the product were collected, evaporated and lyophilized from acetonitrile water mixture. The obtained solid was dissolved in 700 µL of d_6-DMSO. The samples from DMSO solution were diluted x100 in PBS +0.1%SDS and concentration was determined spectroscopically with Nanodrop. Determined stock concentration was 2.1 mM which constitutes to 29% yield (1.8 mg).

^{1}H NMR (600 MHz, DMSO-d_6) δ 9.06 (t, J = 5.6 Hz, 1H), 8.43 (d, J = 9.0 Hz, 1H), 7.99 – 7.97 (m, 2H), 7.82 (d, J = 7.4 Hz, 1H), 7.76 (t, J = 7.6 Hz, 1H), 7.68 (t, J = 7.7 Hz, 1H), 7.61 – 7.58 (m, 2H), 7.37 (t, J = 7.5 Hz, 2H), 7.32 (d, J = 7.6 Hz, 2H), 7.25 – 7.19 (m, 2H), 6.43 (d, J = 8.5 Hz, 2H), 6.38 (d, J = 2.2 Hz, 2H), 6.29 (dd, J = 8.5, 2.2 Hz, 2H), 6.03 (s, 1H), 5.94 (t, J = 9.2 Hz, 1H), 5.59 (s, 4H), 5.39 (d, J = 7.1 Hz, 1H), 5.28 (dd, J = 9.0, 5.9 Hz, 1H), 4.96 (d, J = 9.6 Hz, 1H), 4.70 (s, 1H), 4.65 (s, 1H), 4.42 (d, J = 6.0 Hz, 1H), 4.02 (s, 2H), 3.75 (dd, J = 10.6, 6.6 Hz, 1H), 3.63 (d, J = 7.1 Hz, 1H), 3.38 – 3.37 (m, overlapped with water peak, 2H), 3.30 (s, overlapped with water peak, 3H), 3.21 (s, 3H), 2.68 – 2.63 (m, 1H), 2.24 (s, 3H), 2.17 (t, J = 7.2 Hz, 2H), 1.98 – 1.94 (m, 1H), 1.88 – 1.83 (m, 4H), 1.57 – 1.49 (m, 8H), 1.37 – 1.25 (m, 6H), 1.03 (s, 3H), 0.97 (s, 3H). ESI-MS, positive mode: m/z = 1233.3 $[M+H]^+$. HRMS (ESI) calcd for $C_{69}H_{77}N_4O_{17}$ $[M+H]^+$ 1233.5278, found 1233.5272.

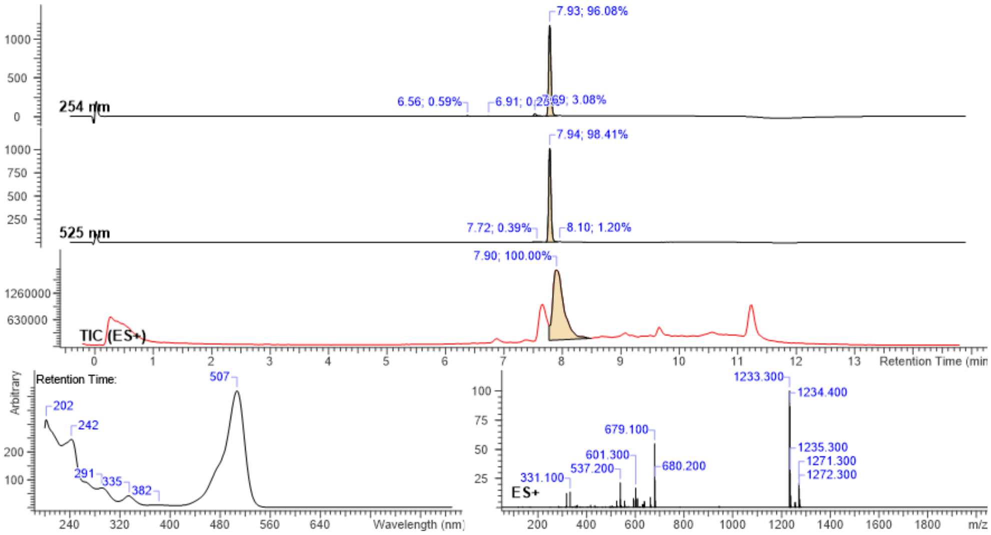

Supplementary Figure 50. LC/MS analysis results of compound **48**.

4-525R-CTX (**49**):

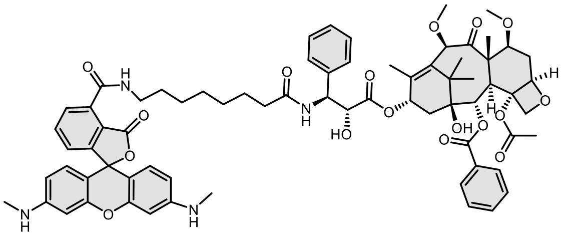

Compound was purified by preparative HPLC (solvent A: H_2O 10mM NH_4COOH pH = 3.6, solvent B: acetonitrile; temperature 25 °C, gradient A:B - 3 min 70:30 isocratic, 4-20 min 70:30 to 0:100 gradient, 20-25 min 0:100 isocratic, 40 mL/min flow). Fractions containing the product were collected, evaporated and lyophilized from acetonitrile water mixture. The obtained solid was dissolved in 700 µL of d_6-DMSO. The samples from DMSO solution were diluted x100 in PBS +0.1%SDS and concentration was determined spectroscopically with Nanodrop. Determined stock concentration was 2.6 mM which constitutes to 37% yield (2.3 mg).

^1H NMR (600 MHz, d_6-DMSO) δ 9.07 (t, J = 5.6 Hz, 1H), 8.43 (d, J = 2.9 Hz, 1H), 7.98 (d, J = 7.6 Hz, 2H), 7.83 (d, J = 7.5 Hz, 1H), 7.76 (t, J = 7.6 Hz, 1H), 7.68 (t, J = 7.7 Hz, 1H), 7.62 – 7.58 (m, 2H), 7.36 (t, J = 7.5 Hz, 2H), 7.32 (d, J = 7.7 Hz, 2H), 7.24 – 7.18 (m, 2H), 6.48 (d, J = 8.5 Hz, 2H), 6.43 – 6.23 (m, 4H), 6.18 (q, J = 5.0 Hz, 2H), 6.03 (br s, 1H), 5.94 (t, J = 9.4 Hz, 1H), 5.39 (d, J = 7.1 Hz, 1H), 5.28 (dd, J = 9.0, 5.9 Hz, 1H), 4.95 (d, J = 9.7 Hz, 1H), 4.70 (s, 1H), 4.65 (s, 1H), 4.42 (s, 1H), 4.02 (s, 2H), 3.75 (dd, J = 10.7, 6.6 Hz, 1H), 3.63 (d, J = 7.1 Hz, 1H), 3.37 – 3.37 (m, overlapped with water peak, 2H), 3.30 (s, overlapped with water peak, 3H), 3.21 (s, 3H), 2.69 (d, J = 4.9 Hz, 6H), 2.66 – 2.60 (m, 1H), 2.24 (s, 3H), 2.17 (t, J = 7.6 Hz, 2H), 1.98 – 1.82 (m, 5H), 1.57 – 1.48 (m, 8H), 1.38 – 1.26 (m, 6H), 1.03 (s, 3H), 0.97 (s, 3H). ESI-MS, positive mode: m/z = 1261.6 $[M+H]^+$. HRMS (ESI) calcd for $C_{71}H_{81}N_4O_{17}$ $[M+H]^+$ 1261.5591, found 1261.5587.

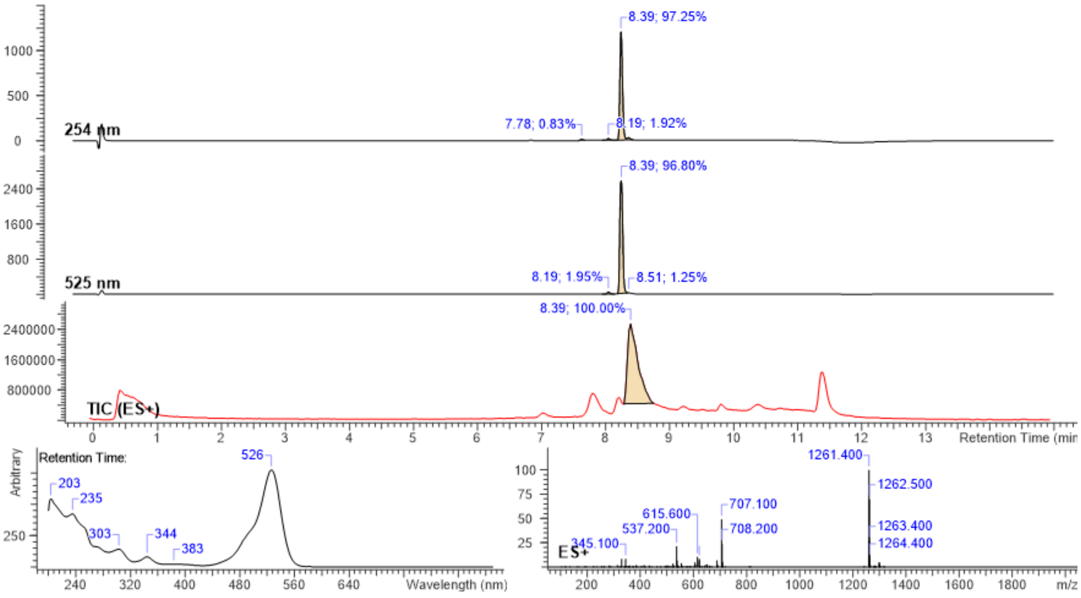

Supplementary Figure 51. LC/MS analysis results of compound **49**.

4-DMRh-CTX (**50**):

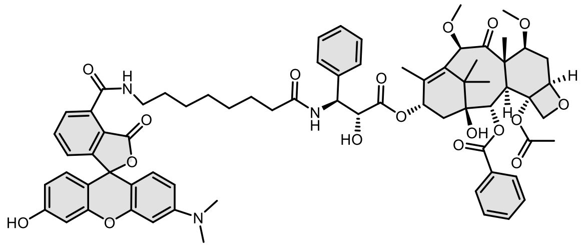

Compound was purified by preparative HPLC (solvent A: H_2O +0.2% HCOOH, solvent B: acetonitrile; temperature 25 °C, gradient A:B - 3 min 70:30 isocratic, 4-20 min 70:30 to 0:100 gradient, 20-25 min 0:100 isocratic, 40 mL/min flow). Fractions containing the product were collected, evaporated and lyophilized from acetonitrile water mixture to obtain product in 46% yield (2.9 mg).

^{1}H NMR (400 MHz, d_6-DMSO) δ 10.12 (s, 1H), 9.05 (t, J = 5.5 Hz, 1H), 8.39 (d, J = 9.0 Hz, 1H), 8.00 – 7.95 (m, 2H), 7.83 (dd, J = 7.5, 1.0 Hz, 1H), 7.75 (t, J = 7.6 Hz, 1H), 7.68 (t, J = 7.3 Hz, 1H), 7.59 (t, J = 7.5 Hz, 2H), 7.38 – 7.30 (m, 4H), 7.25 (dd, J = 7.6, 1.0 Hz, 1H), 7.23 – 7.19 (m, 1H), 6.67 (d, J = 8.7 Hz, 1H), 6.65 – 6.60 (m, 2H), 6.55 – 6.48 (m, 3H), 5.99 – 5.92 (m, 2H), 5.39 (d, J = 7.2 Hz, 1H), 5.28 (dd, J = 9.0, 5.8 Hz, 1H), 4.95 (d, J = 9.4 Hz, 1H), 4.70 (s, 1H), 4.65 (s, 1H), 4.42 (t, J = 6.4 Hz, 1H), 4.02 (s, 2H), 3.75 (dd, J = 10.6, 6.6 Hz, 1H), 3.63 (d, J = 7.1 Hz, 1H), 3.31 – 3.27 (m, 5H), 3.21 (s, 3H), 2.95 (s, 6H), 2.69 – 2.64 (m, 1H), 2.24 (s, 3H), 2.17 (t, J = 7.4 Hz, 2H), 2.00 – 1.94 (m, 1H), 1.89 – 1.80 (m, 4H), 1.62 – 1.45 (m, 8H), 1.40 – 1.25 (m, 6H), 1.03 (s, 3H), 0.97 (s, 3H). ESI-MS, positive mode: m/z = 1262.5 [M+H]$^+$. HRMS (ESI) calcd for $C_{71}H_{80}N_3O_{18}$ [M+H]$^+$ 1262.5431, found 1262.5426.

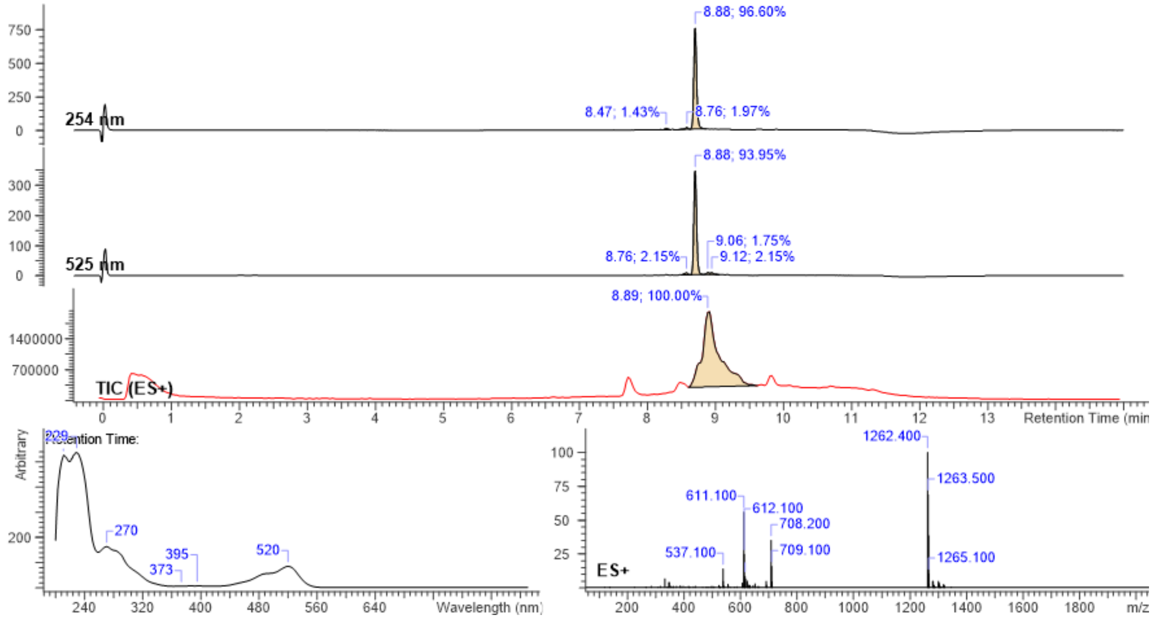

Supplementary Figure 52. LC/MS analysis results of compound **50**.

4-TMR-CTX (**51**):

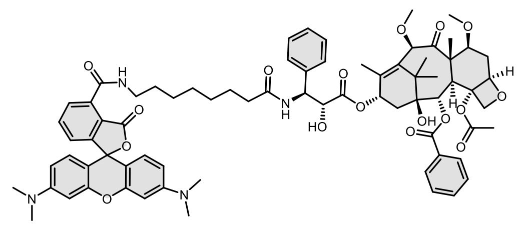

Compound was purified by preparative HPLC (solvent A: H_2O +0.2% HCOOH, solvent B: MeOH; temperature 25 °C, gradient A:B - 3 min 70:30 isocratic, 4-20 min 70:30 to 0:100 gradient, 20-25 min 0:100 isocratic, 40 mL/min flow). Fractions containing the product were collected, evaporated and lyophilized from acetonitrile water mixture. The obtained solid was dissolved in 700 µL of d_6-DMSO. The samples from DMSO solution were diluted x100 in PBS +0.1%SDS and concentration was determined spectroscopically with Nanodrop. Determined stock concentration was 3.4 mM which constitutes to 48% yield (3.1 mg).

^{1}H NMR (400 MHz, d_6-DMSO) δ 9.06 (t, *J* = 5.5 Hz, 1H), 8.38 (d, *J* = 9.1 Hz, 1H), 7.98 (d, *J* = 7.0 Hz, 2H), 7.84 (dd, *J* = 7.5, 1.1 Hz, 1H), 7.76 (t, *J* = 7.6 Hz, 1H), 7.70 – 7.64 (m, 1H), 7.59 (t, *J* = 7.5 Hz, 2H), 7.35 (ddd, *J* = 15.1, 8.2, 6.8 Hz, 4H), 7.24 – 7.19 (m, 2H), 6.61 (dd, *J* = 9.4, 1.6 Hz, 2H), 6.53 – 6.45 (m, 4H), 5.94 (dd, *J* = 8.1, 5.4 Hz, 2H), 5.39 (d, *J* = 7.0 Hz, 1H), 5.32 – 5.28 (m, 1H), 4.95 (d, *J* = 9.1 Hz, 1H), 4.70 (s, 1H), 4.65 (s, 1H), 4.43 (dd, *J* = 6.9, 5.8 Hz, 1H), 4.02 (s, 2H), 3.75 (dd, *J* = 10.9, 6.4 Hz, 1H), 3.63 (d, *J* = 7.1 Hz, 1H), 3.31 – 3.28 (m, 5H), 3.21 (s, 3H), 2.94 (s, 12H), 2.69 – 2.61 (m, 1H), 2.25 (s, 3H), 2.18 (t, *J* = 7.8 Hz, 2H), 1.96 – 1.81 (m, 5H), 1.53 (q, *J* = 6.7, 5.7 Hz, 8H), 1.30 – 1.26 (m, 6H), 1.03 (s, 3H), 0.97 (s, 3H). ESI-MS, positive mode: m/z = 1289.6 $[M+H]^+$. HRMS (ESI) calcd for $C_{73}H_{85}N_4O_{17}$ $[M+H]^+$ 1289.5904, found 1289.5919.

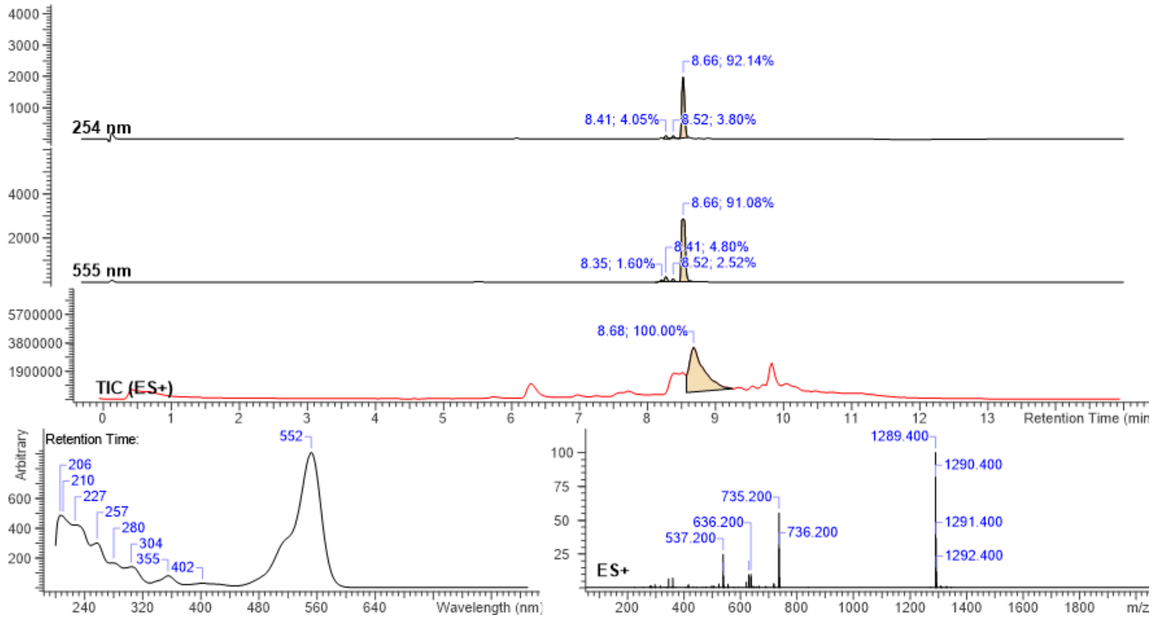

Supplementary Figure 53. LC/MS analysis results of compound **51**.

4-DAllR-CTX (52):

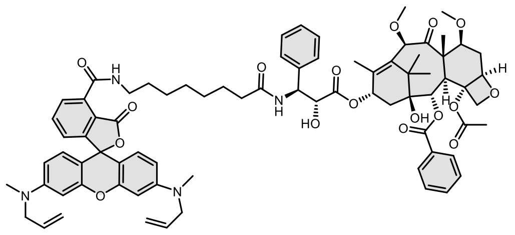

Compound was purified by preparative HPLC (solvent A: H_2O +0.2% HCOOH, solvent B: MeOH; temperature 25 °C, gradient A:B - 3 min 70:30 isocratic, 4-20 min 70:30 to 0:100 gradient, 20-25 min 0:100 isocratic, 40 mL/min flow). Fractions containing the product were collected, evaporated and lyophilized from acetonitrile water mixture. The obtained solid was dissolved in 700 µL of d_6-DMSO. The samples from DMSO solution were diluted x100 in PBS +0.1%SDS and concentration was determined spectroscopically with Nanodrop. Determined stock concentration was 3.9 mM which constitutes to 55% yield (3.8 mg).

^{1}H NMR (400 MHz, DMSO-d_6) δ 9.05 (t, J = 5.6 Hz, 1H), 8.38 (d, J = 9.0 Hz, 1H), 8.01 – 7.95 (m, 2H), 7.84 (dd, J = 7.6, 1.1 Hz, 1H), 7.76 (t, J = 7.6 Hz, 1H), 7.68 (tt, J = 7.4, 1.5 Hz, 1H), 7.59 (t, J = 7.5 Hz, 2H), 7.39 – 7.31 (m, 4H), 7.26 (dd, J = 7.7, 1.0 Hz, 1H), 7.21 (tt, J = 7.2, 1.5 Hz, 1H), 6.60 – 6.55 (m, 2H), 6.50 – 6.45 (m, 4H), 5.94 (t, J = 8.3 Hz, 2H), 5.87 – 5.76 (m, 2H), 5.39 (d, J = 7.2 Hz, 1H), 5.29 (dd, J = 9.0, 5.8 Hz, 1H), 5.14 – 5.07 (m, 4H), 4.95 (dd, J = 9.7, 2.0 Hz, 1H), 4.70 (s, 1H), 4.65 (s, 1H), 4.43 (t, J = 5.8 Hz, 1H), 4.02 (s, 2H), 3.98 (d, J = 4.9 Hz, 4H), 3.75 (dd, J = 10.6, 6.5 Hz, 1H), 3.63 (d, J = 7.2 Hz, 1H), 3.39 – 3.36 (m, 2H), 3.30 (s, 3H), 3.21 (s, 3H), 2.95 (s, 6H), 2.70 – 2.61 (m, 1H), 2.25 (s, 3H), 2.18 (t, J = 7.3 Hz, 2H), 2.00 – 1.86 (m, 2H), 1.83 (s, 3H), 1.57 – 1.48 (m, 8H), 1.39 – 1.26 (m, 6H), 1.03 (s, 3H), 0.97 (s, 3H). ESI-MS, positive mode: m/z = 1341.6 $[M+H]^+$. HRMS (ESI) calcd for $C_{77}H_{89}N_4O_{17}$ $[M+H]^+$ 1341.6217, found 1341.6212.

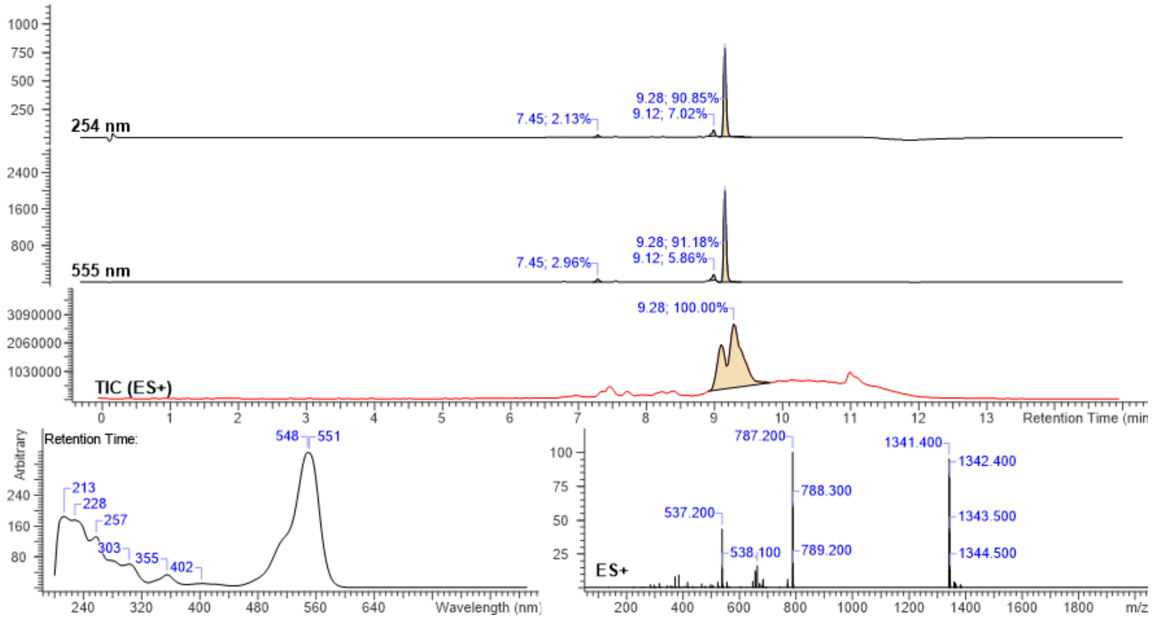

Supplementary Figure 54. LC/MS analysis results of compound **52**.

4-580R-CTX (**53**):

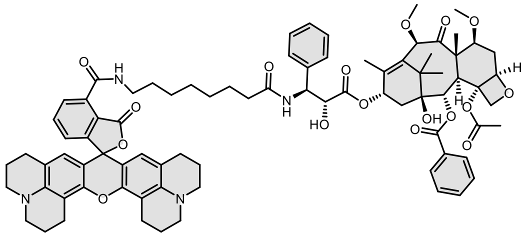

Compound was purified by preparative HPLC (solvent A: H_2O 10mM NH_4COOH pH = 3.6, solvent B: MeOH; temperature 25 °C, gradient A:B - 3 min 60:40 isocratic, 4-20 min 60:40 to 0:100 gradient, 20-25 min 0:100 isocratic, 40 mL/min flow). Fractions containing the product were collected, evaporated and lyophilized from acetonitrile water mixture. The obtained solid was dissolved in 700 µL of d_6-DMSO. The samples from DMSO solution were diluted x100 in PBS +0.1% SDS and concentration was determined spectroscopically with Nanodrop. Determined stock concentration was 3.4 mM which constitutes to 23% yield (1.6 mg).

^{1}H NMR (400 MHz, d_6-DMSO) δ 9.18 (t, *J* = 5.5 Hz, 1H), 8.40 (d, *J* = 9.0 Hz, 1H), 8.00 – 7.94 (m, 2H), 7.85 (dd, *J* = 7.6, 0.9 Hz, 1H), 7.73 (t, *J* = 7.6 Hz, 1H), 7.66 (t, *J* = 7.3 Hz, 1H), 7.58 (t, *J* = 7.5 Hz, 2H), 7.39 – 7.27 (m, 4H), 7.24 (dd, *J* = 7.7, 1.0 Hz, 1H), 7.19 (t, *J* = 7.1 Hz, 1H), 6.10 (s, 2H), 5.99 (d, *J* = 7.0 Hz, 1H), 5.93 (t, *J* = 9.2 Hz, 1H), 5.37 (d, *J* = 7.2 Hz, 1H), 5.26 (dd, *J* = 9.0, 5.7 Hz, 1H), 4.94 (d, *J* = 10.9 Hz, 1H), 4.69 (s, 1H), 4.63 (s, 1H), 4.40 (t, *J* = 6.1 Hz, 1H), 4.01 (s, 2H), 3.74 (dd, *J* = 10.6, 6.6 Hz, 1H), 3.62 (d, *J* = 7.2 Hz, 1H), 3.35 – 3.33 (m, 2H), 3.29 (s, 3H), 3.20 (s, 3H), 3.16 (t, *J* = 5.6 Hz, 2H), 3.11 (t, *J* = 5.6 Hz, 2H), 2.84 (t, *J* = 6.6 Hz, 2H), 2.64 – 2.59 (m, 1H), 2.46 – 2.38 (m, 2H), 2.23 (s, 3H), 2.16 (t, *J* = 7.2 Hz, 2H), 2.02 – 1.89 (m, 5H), 1.88 – 1.66 (m, 8H), 1.59 – 1.41 (m, 8H), 1.40 – 1.17 (m, 14H), 1.02 (s, 3H), 0.96 (s, 3H). ESI-MS, positive mode: m/z = 1393.7 [M+H]$^+$. HRMS (ESI) calcd for $C_{81}H_{93}N_4O_{17}$ [M+H]$^+$ 1393.6530, found 1393.6527.

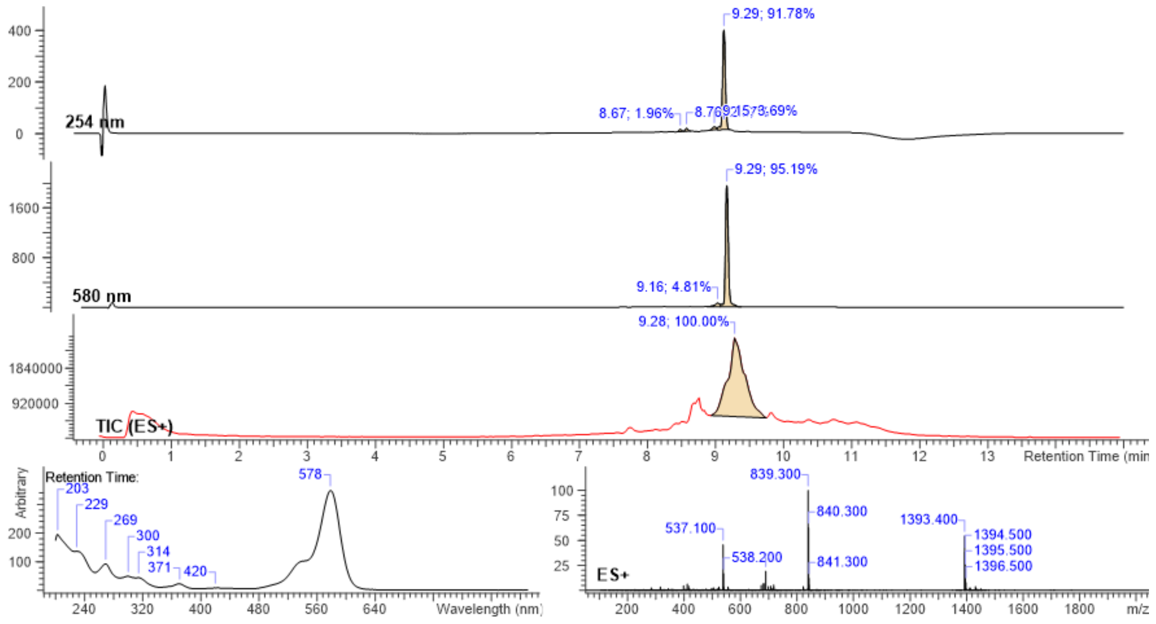

Supplementary Figure 55. LC/MS analysis results of compound **53**.

4-580CP-CTX (**54**):

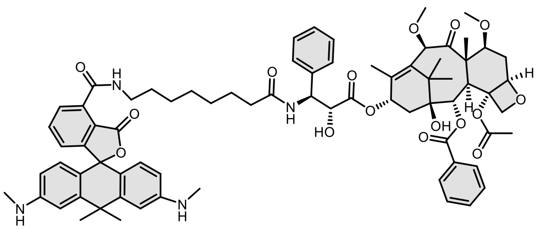

Compound was purified by preparative HPLC (solvent A: H_2O 10mM NH_4COOH pH = 3.6, solvent B: MeOH; temperature 25 °C, gradient A:B - 3 min 60:40 isocratic, 4-20 min 60:40 to 0:100 gradient, 20-25 min 0:100 isocratic, 40 mL/min flow). Fractions containing the product were collected, evaporated and lyophilized from acetonitrile water mixture. The obtained solid was dissolved in 700 µL of d_6-DMSO. The samples from DMSO solution were diluted x100 in PBS +0.1% SDS and concentration was determined spectroscopically with Nanodrop. Determined stock concentration was 3.8 mM which constitutes to 53% yield (3.4 mg).

^{1}H NMR (400 MHz, d_6-DMSO) δ 9.13 (t, *J* = 5.5 Hz, 1H), 8.38 (d, *J* = 9.1 Hz, 1H), 8.01 – 7.94 (m, 2H), 7.81 – 7.77 (m, 1H), 7.72 – 7.65 (m, 2H), 7.59 (t, *J* = 7.4 Hz, 2H), 7.40 – 7.29 (m, 4H), 7.21 (t, *J* = 7.1 Hz, 1H), 7.05 (dd, *J* = 7.7, 1.0 Hz, 1H), 6.76 (d, *J* = 2.3 Hz, 2H), 6.45 (d, *J* = 8.6 Hz, 2H), 6.38 (dd, *J* = 8.7, 2.3 Hz, 2H), 5.98 – 5.91 (m, 2H), 5.85 (d, *J* = 5.1 Hz, 2H), 5.39 (d, *J* = 7.1 Hz, 1H), 5.32 – 5.27 (m, 1H), 4.95 (d, *J* = 9.4 Hz, 1H), 4.71 (s, 1H), 4.65 (s, 1H), 4.43 (t, *J* = 6.4 Hz, 1H), 4.02 (s, 2H), 3.75 (dd, *J* = 10.6, 6.5 Hz, 1H), 3.63 (d, *J* = 7.1 Hz, 1H), 3.35 – 3.33 (m, 2H), 3.30 (s, 3H), 3.21 (s, 3H), 2.70 (d, *J* = 4.7 Hz, 6H), 2.66 – 2.62 (m, 1H), 2.25 (s, 3H), 2.17 (t, *J* = 7.5 Hz, 2H), 2.00 – 1.94 (m, 1H), 1.90 – 1.80 (m, 4H), 1.75 (s, 3H), 1.65 (s, 3H), 1.61 – 1.44 (m, 8H), 1.39 – 1.25 (m, 6H), 1.03 (s, 3H), 0.97 (s, 3H). ESI-MS, positive mode: m/z = 1287.6 $[M+H]^+$. HRMS (ESI) calcd for $C_{74}H_{87}N_4O_{16}$ $[M+H]^+$ 1287.6112, found 1287.6094.

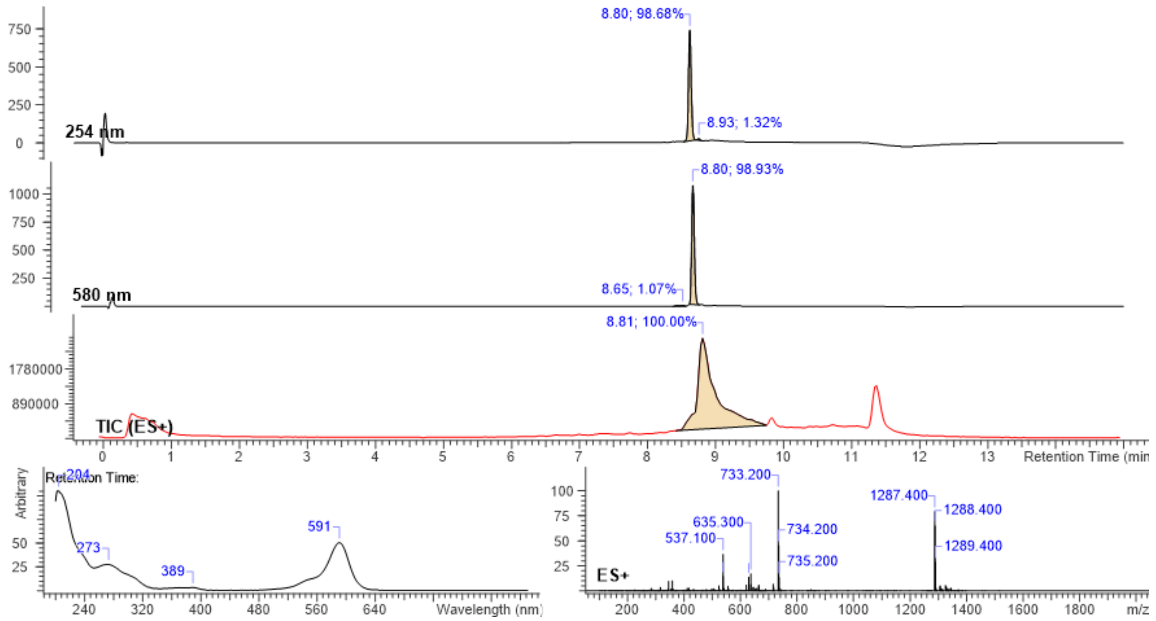

Supplementary Figure 56. LC/MS analysis results of compound **54**.

4-610CP-CTX (**55**):

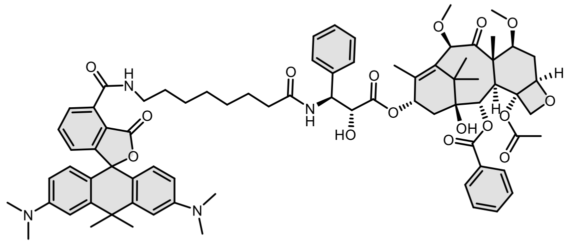

Compound was purified by preparative HPLC (solvent A: 10mM NH_4COOH pH = 3.6, solvent B: MeOH; temperature 25 °C, gradient A:B - 3 min 60:40 isocratic, 4-20 min 60:40 to 0:100 gradient, 20-25 min 0:100 isocratic, 40 mL/min flow). Fractions containing the product were collected, evaporated and lyophilized from acetonitrile water mixture. The obtained solid was dissolved in 700 µL of d_6-DMSO. The samples from DMSO solution were diluted x100 in PBS +0.1% SDS and concentration was determined spectroscopically with Nanodrop. Determined stock concentration was 3.4 mM which constitutes to 48% yield (3.1 mg). Analysis data conforms to previously published data[25].

^{1}H NMR (400 MHz, d_6-DMSO) δ 9.09 (t, *J* = 5.5 Hz, 1H), 8.37 (d, *J* = 9.0 Hz, 1H), 7.98 – 7.91 (m, 2H), 7.75 (dd, *J* = 7.5, 1.0 Hz, 1H), 7.68 – 7.61 (m, 2H), 7.61 – 7.51 (m, 2H), 7.36 – 7.26 (m, 4H), 7.18 (tt, *J* = 7.1, 1.6 Hz, 1H), 7.01 (dd, *J* = 7.7, 1.0 Hz, 1H), 6.88 (d, *J* = 2.0 Hz, 2H), 6.60 – 6.49 (m, 4H), 6.00 – 5.87 (m, 2H), 5.36 (d, *J* = 7.1 Hz, 1H), 5.26 (dd, *J* = 9.1, 5.8 Hz, 1H), 4.92 (dd, *J* = 9.6, 2.1 Hz, 1H), 4.68 (s, 1H), 4.62 (s, 1H), 4.40 (t, *J* = 6.2 Hz, 1H), 3.99 (s, 2H), 3.72 (dd, *J* = 10.6, 6.6 Hz, 1H), 3.60 (d, *J* = 7.1 Hz, 1H), 3.34 – 3.31 (m, 2H), 3.27 (s, 3H), 3.18 (s, 3H), 2.91 (s, 12H), 2.69 – 2.57 (m, 1H), 2.21 (s, 3H), 2.15 (t, *J* = 7.3 Hz, 2H), 1.97 – 1.91 (m, 1H), 1.88 – 1.82 (m, 1H), 1.80 (s, 3H), 1.79 (s, 3H), 1.69 (s, 3H), 1.56 – 1.43 (m, 8H), 1.37 – 1.23 (m, 6H), 1.00 (s, 3H), 0.94 (s, 3H). ESI-MS, positive mode: m/z = 1315.6 $[M+H]^+$. HRMS (ESI) calcd for $C_{76}H_{91}N_4O_{16}$ $[M+H]^+$ 1315.6425, found 1315.6409.

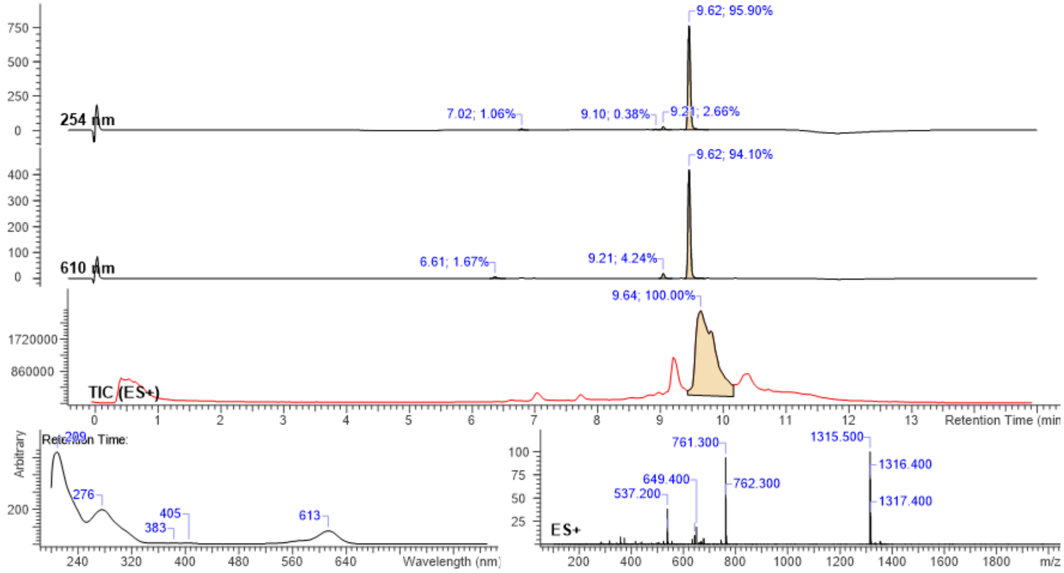

Supplementary Figure 57. LC/MS analysis results of compound **55**.

4-625CP-CTX (**56**):

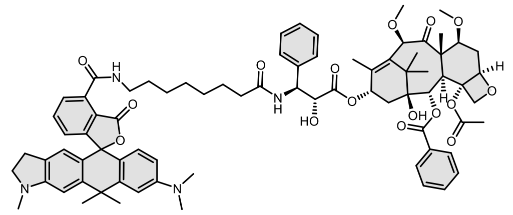

Compound was purified by preparative HPLC (solvent A: 10mM NH_4COOH pH = 3.6, solvent B: MeOH; temperature 25 °C, gradient A:B - 3 min 60:40 isocratic, 4-20 min 60:40 to 0:100 gradient, 20-25 min 0:100 isocratic, 40 mL/min flow). Fractions containing the product were collected, evaporated and lyophilized from acetonitrile water mixture. The obtained solid was dissolved in 700 µL of d_6-DMSO. The samples from DMSO solution were diluted x100 in PBS +0.1% SDS and concentration was determined spectroscopically with Nanodrop. Determined stock concentration was 2.8 mM which constitutes to 39% yield (2.6 mg).

^{1}H NMR (400 MHz, d_6-DMSO) δ 9.13 (t, *J* = 5.6 Hz, 1H), 8.41 (d, *J* = 9.4 Hz, 1H), 8.00 – 7.94 (m, 2H), 7.78 (dd, *J* = 7.6, 1.0 Hz, 1H), 7.68 (td, *J* = 7.5, 1.9 Hz, 2H), 7.59 (t, *J* = 7.5 Hz, 2H), 7.40 – 7.29 (m, 4H), 7.21 (t, *J* = 7.2 Hz, 1H), 7.03 (dd, *J* = 7.7, 1.0 Hz, 1H), 6.89 (d, *J* = 2.4 Hz, 1H), 6.76 (s, 1H), 6.57 (dd, *J* = 9.0, 2.3 Hz, 1H), 6.53 (d, *J* = 8.8 Hz, 1H), 6.37 (s, 1H), 5.96 (t, *J* = 13.8 Hz, 2H), 5.39 (d, *J* = 7.1 Hz, 1H), 5.29 (dd, *J* = 9.0, 5.8 Hz, 1H), 4.95 (d, *J* = 9.2 Hz, 1H), 4.71 (s, 1H), 4.65 (s, 1H), 4.43 (s, 1H), 4.02 (s, 2H), 3.76 (dd, *J* = 10.6, 6.6 Hz, 1H), 3.63 (d, *J* = 7.1 Hz, 1H), 3.35 (s, 2H), 3.30 (s, 3H), 3.27 – 3.23 (m, 2H), 3.21 (s, 3H), 2.93 (s, 6H), 2.79 (s, 3H), 2.76 – 2.64 (m, 3H), 2.24 (s, 3H), 2.18 (t, *J* = 7.4 Hz, 2H), 1.98 – 1.94 (m, 1H), 1.92 – 1.82 (m, 4H), 1.79 (s, 3H), 1.70 (s, 3H), 1.65 – 1.48 (m, 8H), 1.41 – 1.27 (m, 6H), 1.03 (s, 3H), 0.97 (s, 3H). ESI-MS, positive mode: m/z = 1327.6 $[M+H]^+$. HRMS (ESI) calcd for $C_{77}H_{91}N_4O_{1}6$ $[M+H]^+$ 1327.6425, found 1327.6433.

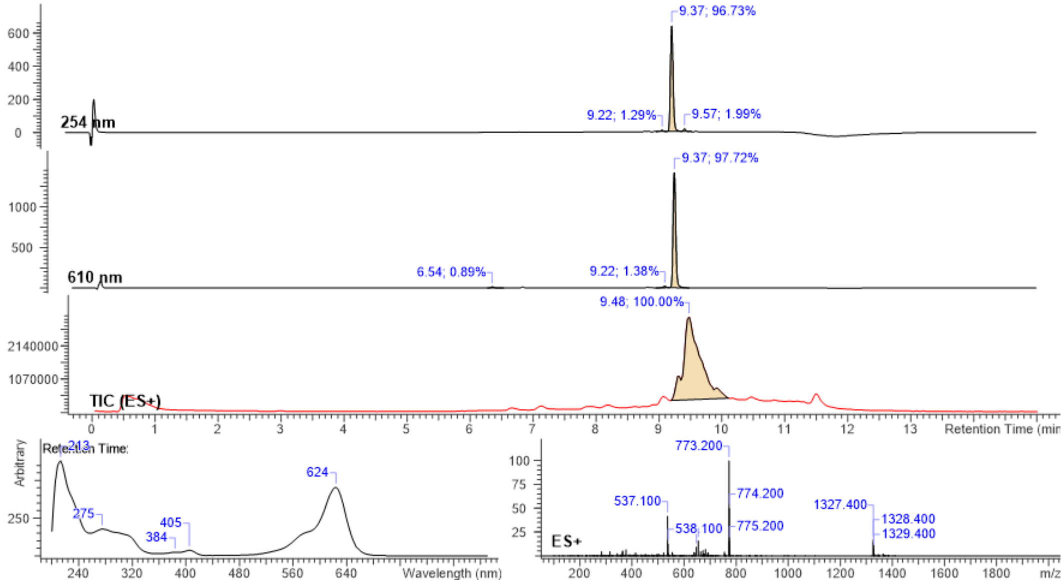

Supplementary Figure 58. LC/MS analysis results of compound **56**.

4-630CP-CTX (**57**):

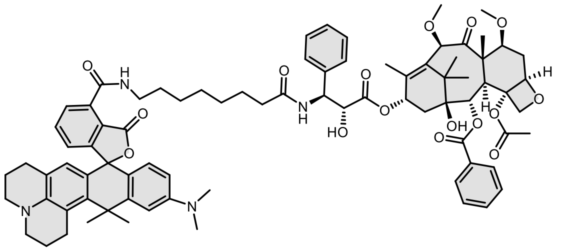

Compound was purified by preparative HPLC (solvent A: 10mM NH_4COOH pH = 3.6, solvent B: MeOH; temperature 25 °C, gradient A:B - 3 min 50:50 isocratic, 4-20 min 50:50 to 0:100 gradient, 20-25 min 0:100 isocratic, 40 mL/min flow). Fractions containing the product were collected, evaporated and lyophilized from acetonitrile water mixture. The obtained solid was dissolved in 700 µL of d_6-DMSO. The samples from DMSO solution were diluted x100 in PBS +0.1% SDS and concentration was determined spectroscopically with Nanodrop. Determined stock concentration was 1.9 mM which constitutes to 27% yield (1.8 mg).

^{1}H NMR (400 MHz, d_6-DMF) δ 9.69 (t, J = 5.4 Hz, 1H), 8.46 (d, J = 9.2 Hz, 1H), 8.15 – 8.07 (m, 3H), 7.79 (t, J = 7.6 Hz, 1H), 7.71 (t, J = 7.4 Hz, 1H), 7.62 (t, J = 7.5 Hz, 2H), 7.50 (d, J = 7.3 Hz, 2H), 7.41 (t, J = 7.7 Hz, 2H), 7.27 (t, J = 7.3 Hz, 1H), 7.15 (dd, J = 7.7, 1.0 Hz, 1H), 6.93 (d, J = 2.5 Hz, 1H), 6.65 – 6.61 (m, 1H), 6.58 (d, J = 8.8 Hz, 1H), 6.31 (s, 1H), 6.17 (t, J = 9.2 Hz, 1H), 6.06 (d, J = 5.9 Hz, 1H), 5.60 (d, J = 7.1 Hz, 1H), 5.56 (dd, J = 9.1, 5.1 Hz, 1H), 5.03 (s, 1H), 4.87 (s, 1H), 4.68 (t, J = 4.9 Hz, 1H), 4.13 (q, J = 8.1 Hz, 2H), 3.92 (dd, J = 10.6, 6.5 Hz, 1H), 3.82 (d, J = 7.1 Hz, 1H), 3.48 – 3.45 (m, 2H), 3.41 (s, 3H), 3.31 (s, 3H), 3.27 – 3.15 (m, 4H), 3.09 – 3.03 (m, 1H), 3.00 (s, 6H), 2.54 – 2.38 (m, 5H), 2.34 – 2.08 (m, 6H), 2.07 – 1.91 (m, 8H), 1.91 (s, 3H), 1.82 – 1.75 (m, 2H), 1.72 – 1.52 (m, 8H), 1.45 – 1.29 (m, 6H), 1.19 (s, 3H), 1.15 (s, 3H). ESI-MS, positive mode: m/z = 1367.7 $[M+H]^+$. HRMS (ESI) calcd for $C_{80}H_{95}N_4O_{16}$ $[M+H]^+$ 1367.6738, found 1367.6745.

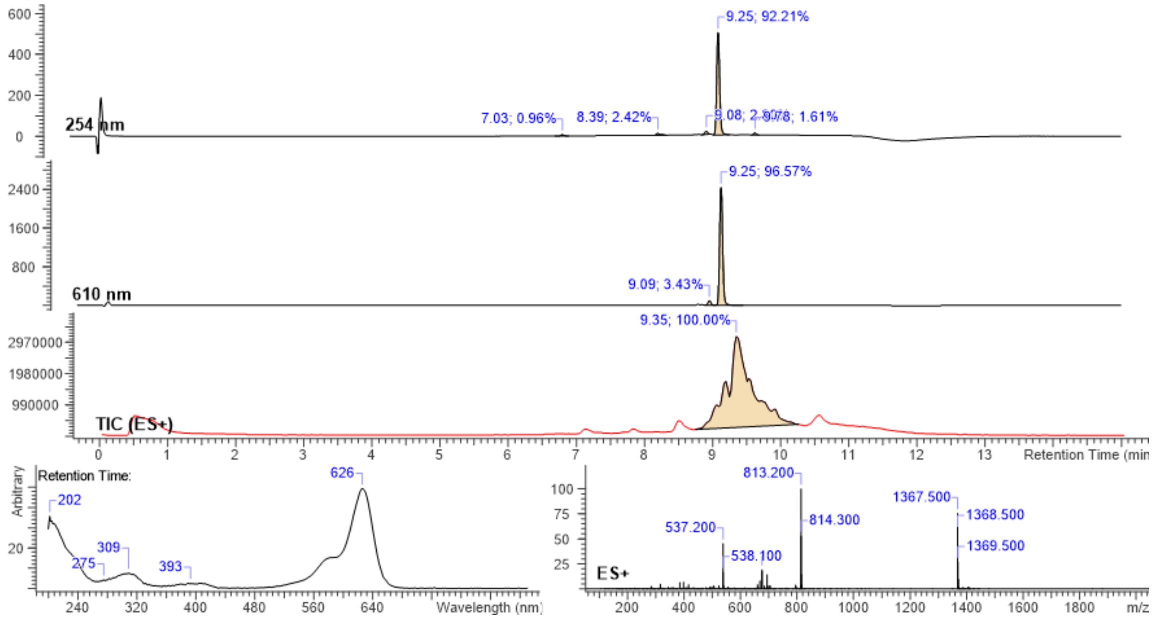

Supplementary Figure 59. LC/MS analysis results of compound **57**.

4-640CP-CTX (**58**):

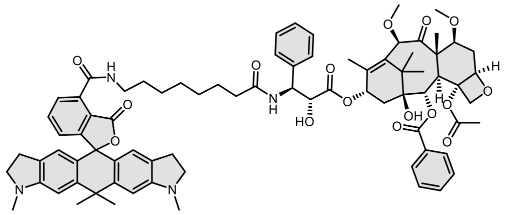

Compound was purified by preparative HPLC (solvent A: 10mM NH_4COOH pH = 3.6, solvent B: MeOH; temperature 25 °C, gradient A:B - 3 min 50:50 isocratic, 4-20 min 50:50 to 0:100 gradient, 20-25 min 0:100 isocratic, 40 mL/min flow). Fractions containing the product were collected, evaporated and lyophilized from acetonitrile water mixture. The obtained solid was dissolved in 700 µL of d_6-DMSO. The samples from DMSO solution were diluted x100 in PBS +0.1% SDS and concentration was determined spectroscopically with Nanodrop. Determined stock concentration was 2.8 mM which constitutes to 39% yield (2.6 mg).

^{1}H NMR (400 MHz, d_6-DMSO) δ 9.15 (t, J = 5.6 Hz, 1H), 8.45 (d, J = 9.0 Hz, 1H), 8.00 – 7.96 (m, 2H), 7.77 (dd, J = 7.5, 1.0 Hz, 1H), 7.68 (t, J = 7.6 Hz, 2H), 7.59 (dd, J = 8.2, 6.7 Hz, 2H), 7.35 (ddd, J = 14.8, 8.2, 6.7 Hz, 4H), 7.23 – 7.18 (m, 1H), 7.03 (dd, J = 7.7, 1.0 Hz, 1H), 6.74 (s, 2H), 6.34 (s, 2H), 6.07 (d, J = 6.7 Hz, 1H), 5.95 (t, J = 9.0 Hz, 1H), 5.39 (d, J = 7.1 Hz, 1H), 5.28 (dd, J = 9.0, 5.9 Hz, 1H), 4.95 (dd, J = 9.6, 2.1 Hz, 1H), 4.70 (s, 1H), 4.65 (s, 1H), 4.43 (t, J = 5.7 Hz, 1H), 4.02 (s, 2H), 3.76 (dd, J = 10.6, 6.6 Hz, 1H), 3.63 (d, J = 7.1 Hz, 1H), 3.34 – 3.33 (m, 2H), 3.30 (s, 3H), 3.27 – 3.17 (m, 7H), 2.78 (s, 6H), 2.75 – 2.60 (m, 5H), 2.24 (s, 3H), 2.18 (t, J = 7.4 Hz, 2H), 1.98 – 1.83 (m, 5H), 1.76 (s, 3H), 1.69 (s, 3H), 1.60 – 1.46 (m, 8H), 1.39 – 1.25 (m, 6H), 1.03 (s, 3H), 0.97 (s, 3H). ESI-MS, positive mode: m/z = 1339.6 $[M+H]^+$. HRMS (ESI) calcd for $C_{78}H_{91}N_4O_{16}$ $[M+H]^+$ 1339.6425, found 1339.6428.

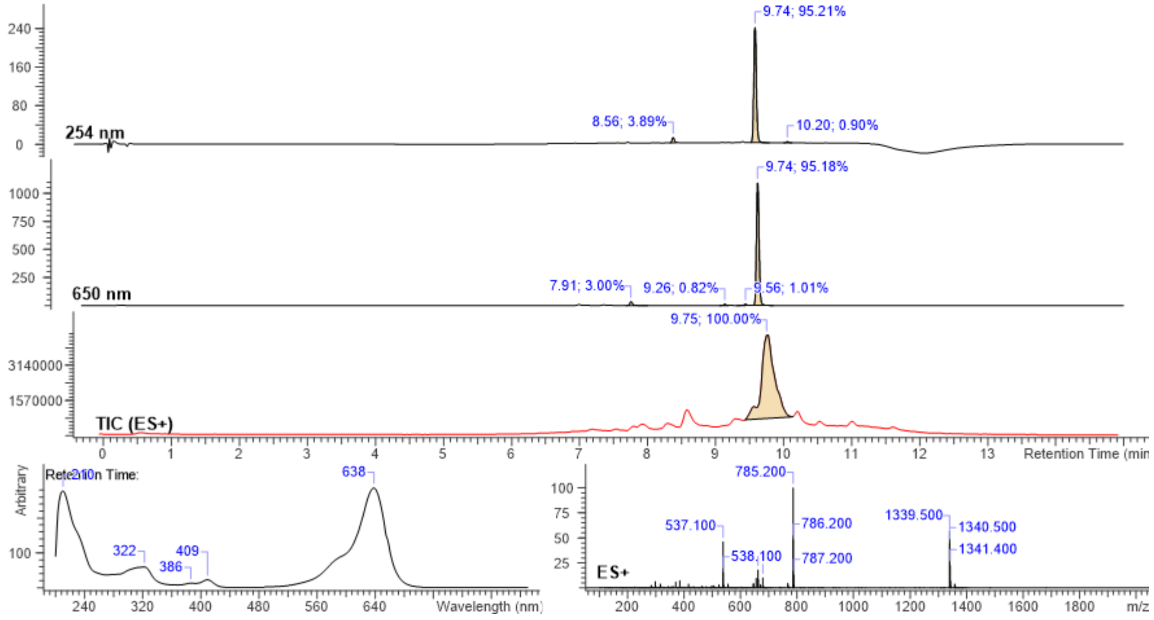

Supplementary Figure 60. LC/MS analysis results of compound **58**.

4-642CP-CTX (**59**):

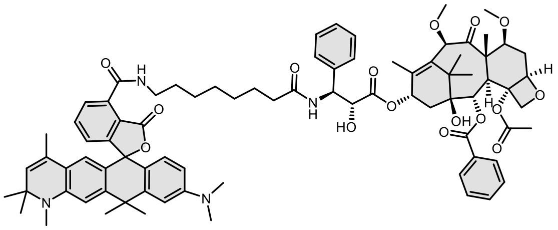

Compound was purified by preparative HPLC (solvent A: 10mM NH_4COOH pH = 3.6, solvent B: MeOH; temperature 25 °C, gradient A:B - 3 min 50:50 isocratic, 4-20 min 50:50 to 0:100 gradient, 20-25 min 0:100 isocratic, 40 mL/min flow). Fractions containing the product were collected, evaporated and lyophilized from acetonitrile water mixture. The obtained solid was dissolved in 700 µL of d_6-DMSO. The samples from DMSO solution were diluted x100 in PBS +0.1% SDS and concentration was determined spectroscopically with Nanodrop. Determined stock concentration was 3.4 mM which constitutes to 48% yield (3.3 mg). ^{1}H NMR (400 MHz, d_6-DMSO) δ 9.17 (t, J = 5.6 Hz, 1H), 8.40 (d, J = 9.0 Hz, 1H), 8.01 – 7.94 (m, 2H), 7.85 – 7.82 (m, 1H), 7.76 – 7.63 (m, 3H), 7.59 (t, J = 7.4 Hz, 2H), 7.41 – 7.28 (m, 4H), 7.20 (t, J = 7.1 Hz, 1H), 7.10 (dd, J = 7.7, 1.0 Hz, 1H), 6.91 (s, 1H), 6.67 (s, 1H), 6.56 (d, J = 1.3 Hz, 2H), 6.16 (s, 1H), 6.01 – 5.91 (m, 2H), 5.38 (d, J = 7.1 Hz, 1H), 5.32 (d, J = 1.5 Hz, 1H), 5.28 (dd, J = 9.0, 5.8 Hz, 1H), 4.95 (d, J = 9.2 Hz, 1H), 4.70 (s, 1H), 4.65 (s, 1H), 4.42 (t, J = 4.9 Hz, 1H), 4.02 (s, 2H), 3.75 (dd, J = 10.6, 6.6 Hz, 2H), 3.63 (d, J = 7.1 Hz, 1H), 3.36 – 3.36 (m, 2H), 3.30 (s, 3H), 3.21 (s, 3H), 2.94 (s, 6H), 2.84 (s, 3H), 2.68 – 2.63 (m, 1H), 2.24 (s, 3H), 2.18 (t, J = 7.3 Hz, 2H), 2.02 – 1.77 (m, 8H), 1.70 (s, 3H), 1.69 – 1.39 (m, 11H), 1.37 – 1.19 (m, 12H), 1.03 (s, 3H), 0.97 (s, 3H). ESI-MS, positive mode: m/z = 1381.7 $[M+H]^+$. HRMS (ESI) calcd for $C_{81}H_{97}N_4O_{16}$ $[M+H]^+$ 1381.6894, found 1381.6904.

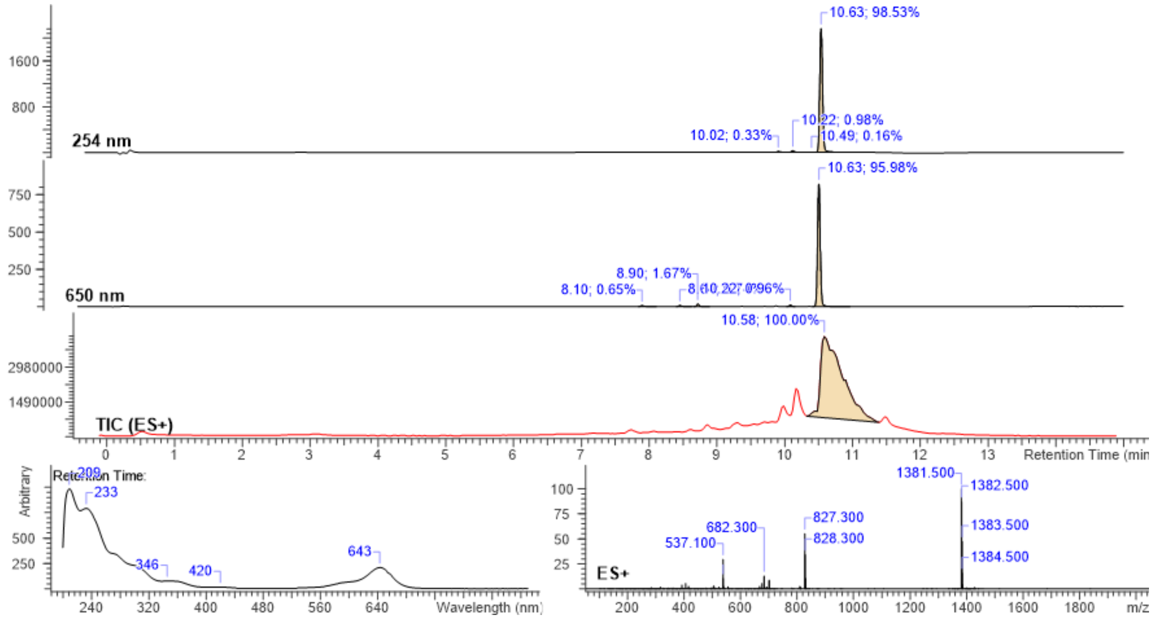

Supplementary Figure 61. LC/MS analysis results of compound **59**.

4-645CP-CTX (**60**):

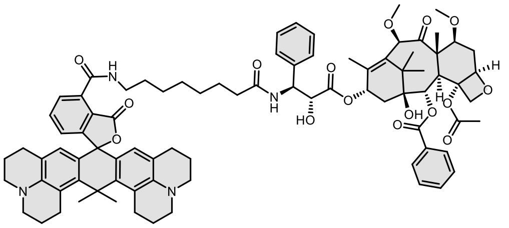

Compound was purified by preparative HPLC (solvent A: 10mM NH_4COOH pH = 3.6, solvent B: MeOH; temperature 25 °C, gradient A:B - 3 min 50:50 isocratic, 4-20 min 50:50 to 0:100 gradient, 20-25 min 0:100 isocratic, 40 mL/min flow). Fractions containing the product were collected, evaporated and lyophilized from acetonitrile water mixture. The obtained solid was dissolved in 700 µL of d_6-DMSO. The samples from DMSO solution were diluted x100 in PBS +0.1% SDS and concentration was determined spectroscopically with Nanodrop. Determined stock concentration was 1.6 mM which constitutes to 22% yield (1.6 mg).

^{1}H NMR (400 MHz, d_6-DMSO) δ 9.75 (t, *J* = 5.3 Hz, 1H), 8.51 (d, *J* = 8.8 Hz, 1H), 8.13 – 8.08 (m, 2H), 7.77 (t, *J* = 7.6 Hz, 2H), 7.70 (tt, *J* = 7.4, 1.2 Hz, 1H), 7.62 (t, *J* = 7.4 Hz, 2H), 7.50 (d, *J* = 7.1 Hz, 2H), 7.41 (t, *J* = 7.7 Hz, 2H), 7.27 (tt, *J* = 7.3, 1.2 Hz, 1H), 7.10 (dd, *J* = 7.7, 1.0 Hz, 1H), 6.20 (s, 2H), 6.16 (d, *J* = 9.0 Hz, 2H), 5.60 (d, *J* = 7.1 Hz, 1H), 5.55 (dd, *J* = 9.1, 5.1 Hz, 1H), 5.05 – 4.99 (m, 2H), 4.87 (s, 1H), 4.68 (d, *J* = 5.2 Hz, 1H), 4.16 – 4.09 (m, 2H), 3.92 (dd, *J* = 10.6, 6.6 Hz, 1H), 3.82 (d, *J* = 7.2 Hz, 1H), 3.46 (d, *J* = 6.2 Hz, 2H), 3.41 (s, 3H), 3.31 (s, 3H), 3.27 – 3.06 (m, 8H), 2.90 (s, 16H), 2.61 – 2.32 (m, 9H), 2.32 – 2.22 (m, 3H), 2.18 – 2.13 (m, 1H), 2.06 (s, 3H), 2.00 (s, 3H), 1.98 (d, *J* = 1.4 Hz, 3H), 1.98 – 1.89 (m, 4H), 1.77 (td, *J* = 11.6, 10.8, 5.5 Hz, 4H), 1.62 (d, *J* = 23.5 Hz, 7H), 1.45 – 1.28 (m, 6H), 1.18 (s, 3H), 1.14 (s, 3H). ESI-MS, positive mode: m/z = 1419.7 $[M+H]^+$. HRMS (ESI) calcd for $C_{84}H_{99}N_4O_{16}$ $[M+H]^+$ 1419.7051, found 1419.7039.

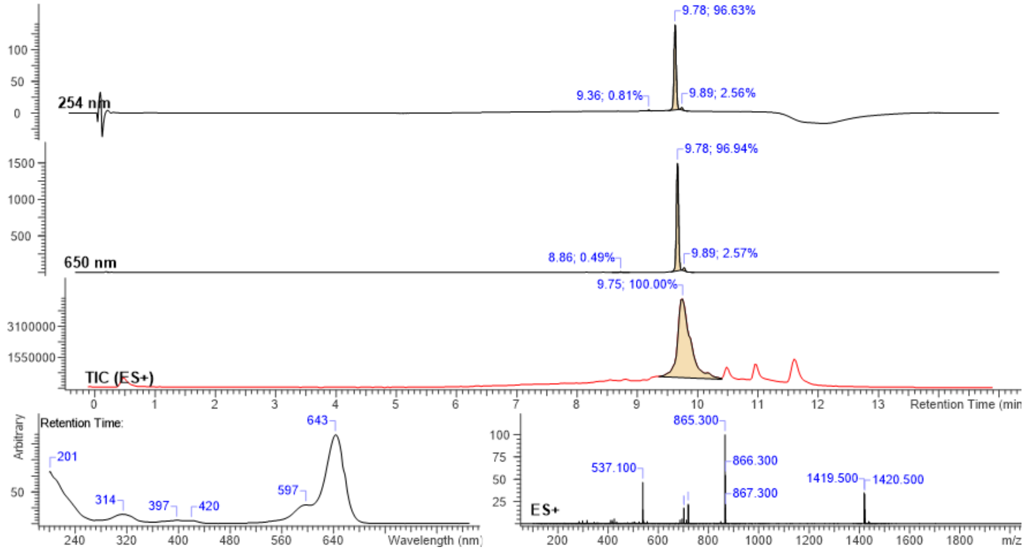

Supplementary Figure 62. LC/MS analysis results of compound **60**.

4-SiR-CTX (**61**):

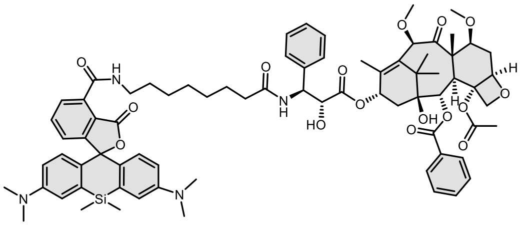

Compound was purified by preparative HPLC (solvent A: H_2O +0.2% HCOOH, solvent B: MeOH; temperature 25 °C, gradient A:B - 3 min 50:50 isocratic, 4-20 min 50:50 to 0:100 gradient, 20-25 min 0:100 isocratic, 40 mL/min flow). Fractions containing the product were collected, evaporated and lyophilized from acetonitrile water mixture.to give product in 55% yield (3.7 mg) as a light blue solid.

^{1}H NMR (400 MHz, d_6-DMSO) δ 9.02 (t, *J* = 5.5 Hz, 1H), 8.37 (d, *J* = 9.0 Hz, 1H), 7.98 (d, *J* = 6.9 Hz, 2H), 7.77 – 7.74 (m, 2H), 7.69 – 7.64 (m, 1H), 7.59 (t, *J* = 7.5 Hz, 2H), 7.39 – 7.30 (m, 4H), 7.26 – 7.21 (m, 2H), 7.00 (d, *J* = 2.8 Hz, 2H), 6.70 (dd, *J* = 9.0, 1.2 Hz, 2H), 6.64 (ddd, *J* = 8.9, 2.8, 1.4 Hz, 2H), 5.95 (dd, *J* = 8.5, 6.4 Hz, 2H), 5.39 (d, *J* = 7.1 Hz, 1H), 5.29 (dd, *J* = 9.1, 5.8 Hz, 1H), 4.95 (d, *J* = 9.5 Hz, 1H), 4.70 (s, 1H), 4.65 (s, 1H), 4.43 (dd, *J* = 6.9, 5.8 Hz, 1H), 4.02 (s, 2H), 3.75 (dd, *J* = 10.6, 6.5 Hz, 1H), 3.63 (d, *J* = 7.1 Hz, 1H), 3.30 (s, 3H), 3.30 – 3.27 (m, 2H), 3.21 (s, 3H), 2.92 (s, 12H), 2.72 – 2.59 (m, 1H), 2.25 (s, 3H), 2.18 (t, *J* = 7.4 Hz, 2H), 2.01 – 1.93 (m, 1H), 1.90 – 1.84 (m, 1H), 1.83 (s, 3H), 1.57 – 1.46 (m, 8H), 1.37 – 1.20 (m, 6H), 1.03 (s, 3H), 0.97 (s, 3H), 0.62 (s, 3H), 0.52 (s, 3H). ESI-MS, positive mode: m/z = 1353.6 $[M+Na]^+$. HRMS (ESI) calcd for $C_{75}H_{90}N_4O_{16}SiNa$ $[M+Na]^+$ 1353.6013, found 1353.6003.

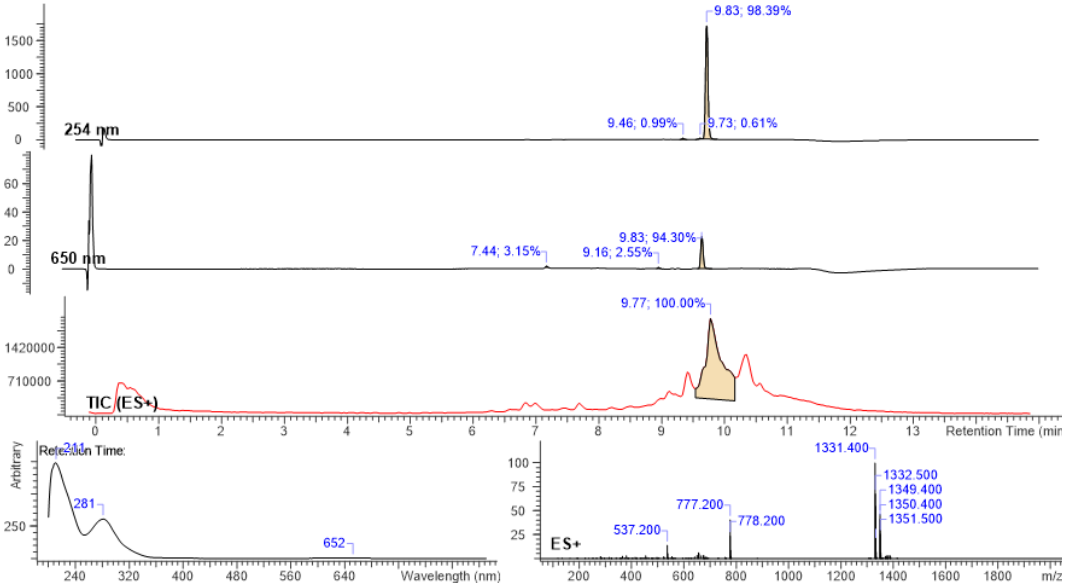

Supplementary Figure 63. LC/MS analysis results of compound **61**.

4-665SiR-CTX (**62**):

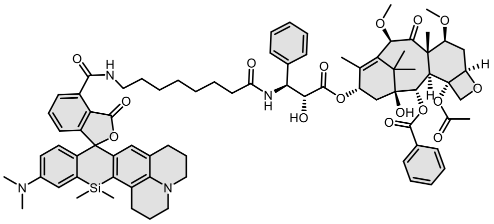

Compound was purified by preparative HPLC (solvent A: 10mM NH_4COOH pH = 3.6, solvent B: MeOH; temperature 25 °C, gradient A:B - 3 min 50:50 isocratic, 4-20 min 50:50 to 0:100 gradient, 20-25 min 0:100 isocratic, 40 mL/min flow). Fractions containing the product were collected, evaporated and lyophilized from acetonitrile water mixture. The obtained solid was dissolved in 700 µL of d_6-DMSO. The samples from DMSO solution were diluted x100 in PBS +0.1% SDS and concentration was determined spectroscopically with Nanodrop. Determined stock concentration was 2.4 mM which constitutes to 34% yield (2.3 mg).

^{1}H NMR (400 MHz, d_6-DMSO) δ 9.06 (t, J = 5.5 Hz, 1H), 8.41 (d, J = 9.0 Hz, 1H), 8.01 – 7.93 (m, 2H), 7.75 – 7.63 (m, 3H), 7.59 (t, J = 7.4 Hz, 2H), 7.35 (ddd, J = 15.2, 8.3, 6.9 Hz, 4H), 7.21 (t, J = 7.1 Hz, 1H), 7.13 – 7.09 (m, 1H), 6.93 (d, J = 2.6 Hz, 1H), 6.70 – 6.62 (m, 2H), 6.33 (s, 1H), 6.00 (d, J = 6.7 Hz, 1H), 5.95 (t, J = 9.3 Hz, 2H), 5.39 (d, J = 7.1 Hz, 1H), 5.29 (dd, J = 9.0, 5.8 Hz, 1H), 4.95 (d, J = 9.5 Hz, 1H), 4.70 (s, 1H), 4.65 (s, 1H), 4.43 (t, J = 6.1 Hz, 1H), 4.02 (s, 2H), 3.75 (dd, J = 10.6, 6.6 Hz, 1H), 3.63 (d, J = 7.2 Hz, 1H), 3.31 – 3.24 (m, 5H), 3.21 (s, 3H), 3.16 (t, J = 5.8 Hz, 2H), 3.11 (t, J = 5.3 Hz, 2H), 3.02 – 2.78 (m, 8H), 2.68 – 2.62 (m, 1H), 2.44 (dd, J = 14.8, 8.3 Hz, 2H), 2.24 (s, 3H), 2.18 (t, J = 7.3 Hz, 2H), 1.97 – 1.73 (m, 8H), 1.58 – 1.44 (m, 7H), 1.38 – 1.24 (m, 6H), 1.03 (s, 3H), 0.97 (s, 3H), 0.66 (s, 3H), 0.55 (s, 3H). ESI-MS, positive mode: m/z = 1383.7 [M+H]$^+$. HRMS (ESI) calcd for $C_{79}H_{95}N_4O_{16}Si$ [M+H]$^+$ 1383.6507, found 1383.6513.

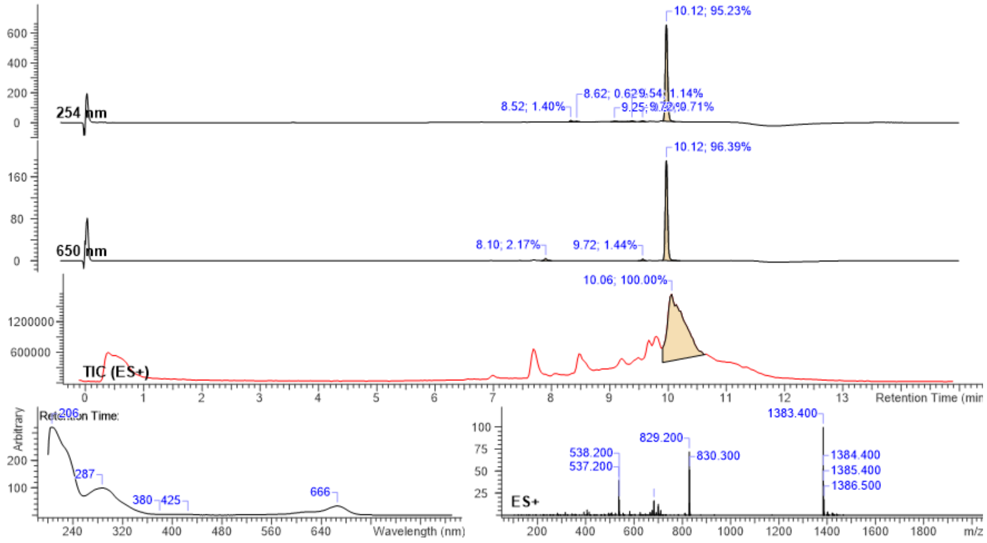

Supplementary Figure 64. LC/MS analysis results of compound **62**.

4-670SiR-CTX (**63**):

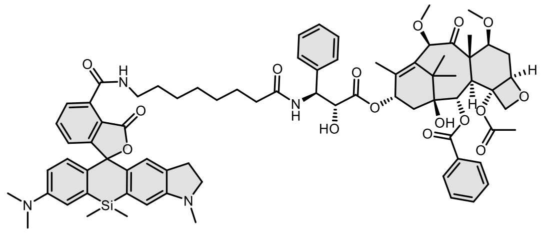

Compound was purified by preparative HPLC (solvent A: H_2O +0.2% HCOOH, solvent B: MeOH; temperature 25 °C, gradient A:B - 3 min 50:50 isocratic, 4-20 min 50:50 to 0:100 gradient, 20-25 min 0:100 isocratic, 40 mL/min flow). Fractions containing the product were collected, evaporated and lyophilized from acetonitrile water mixture to give product in 34% yield (2.3 mg).

^{1}H NMR (400 MHz, d_6-DMSO) δ 9.03 (t, *J* = 5.5 Hz, 1H), 8.38 (d, *J* = 9.0 Hz, 1H), 8.01 – 7.94 (m, 2H), 7.77 – 7.71 (m, 2H), 7.67 (tt, *J* = 7.2, 1.4 Hz, 1H), 7.59 (t, *J* = 7.4 Hz, 2H), 7.40 – 7.30 (m, 4H), 7.24 – 7.17 (m, 2H), 6.98 (d, *J* = 2.8 Hz, 1H), 6.80 (s, 1H), 6.70 (dd, *J* = 9.0, 1.0 Hz, 1H), 6.64 (ddd, *J* = 9.1, 2.8, 1.6 Hz, 1H), 6.57 (s, 1H), 5.99 – 5.91 (m, 2H), 5.39 (d, *J* = 7.1 Hz, 1H), 5.29 (dd, *J* = 9.0, 5.8 Hz, 1H), 4.95 (d, *J* = 9.4 Hz, 1H), 4.70 (s, 1H), 4.65 (s, 1H), 4.43 (t, *J* = 6.3 Hz, 1H), 4.02 (s, 2H), 3.75 (dd, *J* = 10.6, 6.6 Hz, 1H), 3.63 (d, *J* = 7.1 Hz, 1H), 3.31 – 3.28 (m, 5H), 3.26 – 3.16 (m, 5H), 2.91 (s, 6H), 2.80 – 2.63 (m, 6H), 2.25 (s, 3H), 2.18 (t, *J* = 7.4 Hz, 2H), 1.97 (d, *J* = 6.3 Hz, 1H), 1.90 – 1.80 (m, 4H), 1.60 – 1.45 (m, 8H), 1.38 – 1.25 (m, 6H), 1.03 (s, 3H), 0.97 (s, 3H), 0.60 (s, 3H), 0.50 (s, 3H). ESI-MS, positive mode: m/z = 1343.6 $[M+H]^+$. HRMS (ESI) calcd for $C_{76}H_{91}N_4O_{16}Si$ $[M+H]^+$ 1343.6194, found 1343.6195.

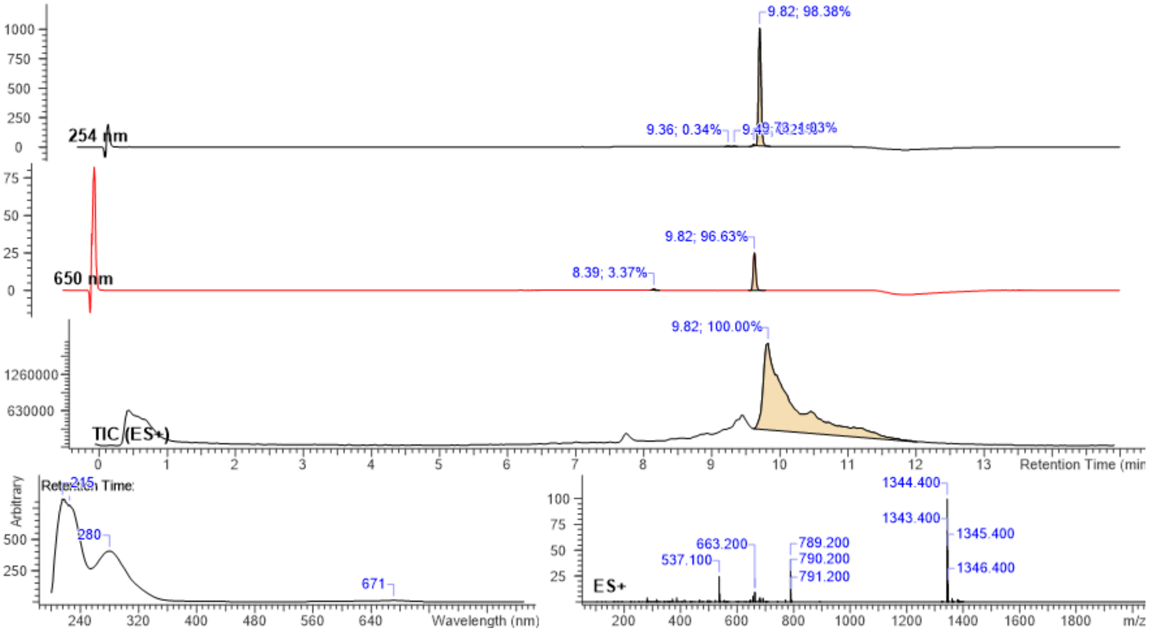

Supplementary Figure 65. LC/MS analysis results of compound **63**.

4-685SiR-CTX (**64**):

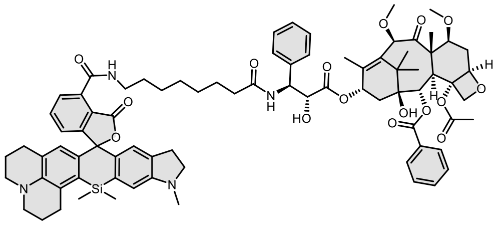

Compound was purified by preparative HPLC (solvent A: H_2O +0.2% HCOOH, solvent B: MeOH; temperature 25 °C, gradient A:B - 3 min 40:60 isocratic, 4-20 min 40:60 to 0:100 gradient, 20-25 min 0:100 isocratic, 40 mL/min flow). Fractions containing the product were collected, evaporated and lyophilized from acetonitrile water mixture. The obtained solid was dissolved in 700 µL of d_6-DMSO. The samples from DMSO solution were diluted x100 in PBS +0.1% SDS and concentration was determined spectroscopically with Nanodrop. Determined stock concentration was 2.8 mM which constitutes to 39% yield (2.7 mg).

^{1}H NMR (400 MHz, d_6-DMSO) δ 9.06 (t, J = 5.5 Hz, 1H), 8.42 (d, J = 9.0 Hz, 1H), 8.00 – 7.93 (m, 2H), 7.73 – 7.62 (m, 3H), 7.58 (t, J = 7.5 Hz, 2H), 7.39 – 7.26 (m, 4H), 7.19 (tt, J = 7.1, 1.4 Hz, 1H), 7.06 (dd, J = 7.4, 1.3 Hz, 1H), 6.73 (s, 1H), 6.53 (s, 1H), 6.31 (s, 1H), 6.02 (d, J = 4.8 Hz, 1H), 5.96 – 5.90 (m, 1H), 5.37 (d, J = 7.1 Hz, 1H), 5.27 (dd, J = 8.9, 5.9 Hz, 1H), 4.94 (d, J = 9.6 Hz, 1H), 4.69 (s, 1H), 4.63 (s, 1H), 4.41 (t, J = 5.0 Hz, 1H), 4.01 (s, 2H), 3.74 (dd, J = 10.6, 6.6 Hz, 1H), 3.62 (d, J = 7.2 Hz, 1H), 3.29 – 3.28 (m, 5H), 3.22 – 3.08 (m, 9H), 2.89 (t, J = 6.3 Hz, 2H), 2.80 – 2.62 (m, 6H), 2.46 – 2.38 (m, 2H), 2.23 (s, 3H), 2.16 (t, J = 7.4 Hz, 2H), 1.99 – 1.79 (m, 7H), 1.77 – 1.70 (m, 2H), 1.61 – 1.41 (m, 8H), 1.35 – 1.23 (m, 6H), 1.02 (s, 3H), 0.96 (s, 3H), 0.63 (s, 3H), 0.52 (s, 3H). ESI-MS, positive mode: m/z = 1395.6 [M+H]$^+$. HRMS (ESI) calcd for $C_{80}H_{95}N_4O_{16}Si$ [M+H]$^+$ 1395.6507, found 1395.6494.

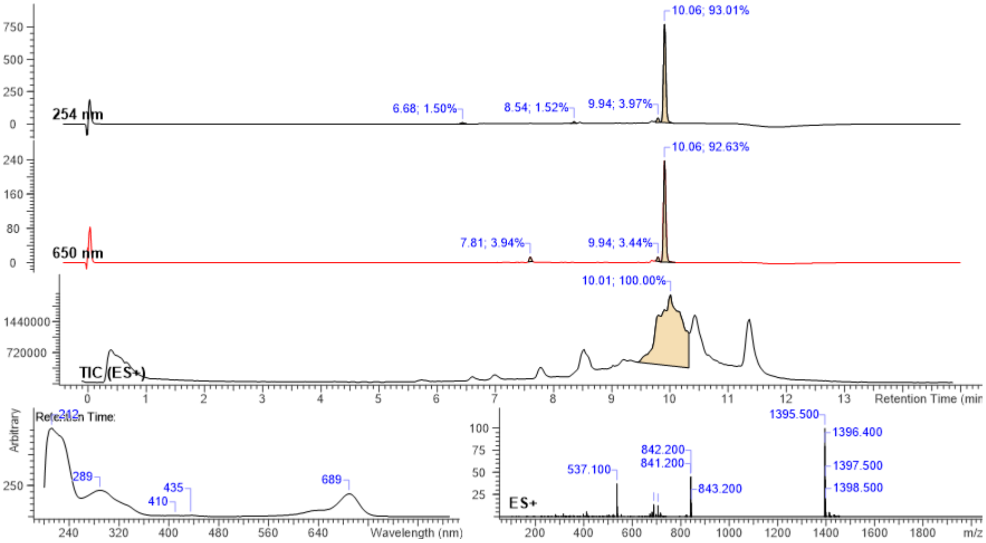

Supplementary Figure 66. LC/MS analysis results of compound **64**.

4-690SiR-CTX (**65**):

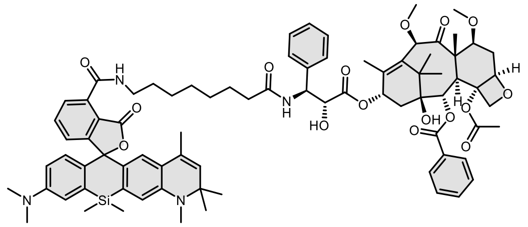

Compound was purified by preparative HPLC (solvent A: H_2O +0.2% HCOOH, solvent B: MeOH; temperature 25 °C, gradient A:B - 3 min 30:70 isocratic, 4-20 min 30:70 to 0:100 gradient, 20-25 min 0:100 isocratic, 40 mL/min flow). Fractions containing the product were collected, evaporated and lyophilized from acetonitrile water mixture to give product in 52% yield (3.6 mg).

^{1}H NMR (400 MHz, d_6-DMSO) δ 9.01 (t, *J* = 5.1 Hz, 1H), 8.37 (d, *J* = 9.0 Hz, 1H), 8.01 – 7.95 (m, 2H), 7.83 – 7.76 (m, 2H), 7.69 – 7.64 (m, 1H), 7.59 (t, *J* = 7.4 Hz, 2H), 7.41 – 7.27 (m, 5H), 7.20 (tt, *J* = 7.1, 1.4 Hz, 1H), 7.01 (d, *J* = 2.8 Hz, 1H), 6.76 (s, 1H), 6.69 (dd, *J* = 8.9, 0.9 Hz, 1H), 6.62 (ddd, *J* = 9.1, 2.9, 1.3 Hz, 1H), 6.41 (s, 1H), 5.99 – 5.90 (m, 2H), 5.39 (d, *J* = 7.1 Hz, 1H), 5.34 (d, *J* = 1.5 Hz, 1H), 5.29 (dd, *J* = 9.0, 5.8 Hz, 1H), 4.95 (d, *J* = 9.6 Hz, 1H), 4.70 (s, 1H), 4.65 (s, 1H), 4.42 (t, *J* = 6.3 Hz, 1H), 4.02 (s, 2H), 3.75 (dd, *J* = 10.6, 6.6 Hz, 1H), 3.63 (d, *J* = 7.1 Hz, 1H), 3.31 – 3.24 (m, 5H), 3.21 (s, 3H), 2.91 (s, 6H), 2.81 (s, 3H), 2.69 – 2.62 (m, 1H), 2.25 (s, 3H), 2.17 (t, *J* = 7.4 Hz, 2H), 1.99 – 1.82 (m, 5H), 1.57 (d, *J* = 1.4 Hz, 3H), 1.56 – 1.40 (m, 8H), 1.35 – 1.21 (m, 12H), 1.03 (s, 3H), 0.97 (s, 3H), 0.63 (s, 3H), 0.51 (s, 3H). ESI-MS, positive mode: m/z = 1397.7 $[M+H]^+$. HRMS (ESI) calcd for $C_{80}H_{97}N_4O_{16}Si$ $[M+H]^+$ 1397.6663, found 1397.6662.

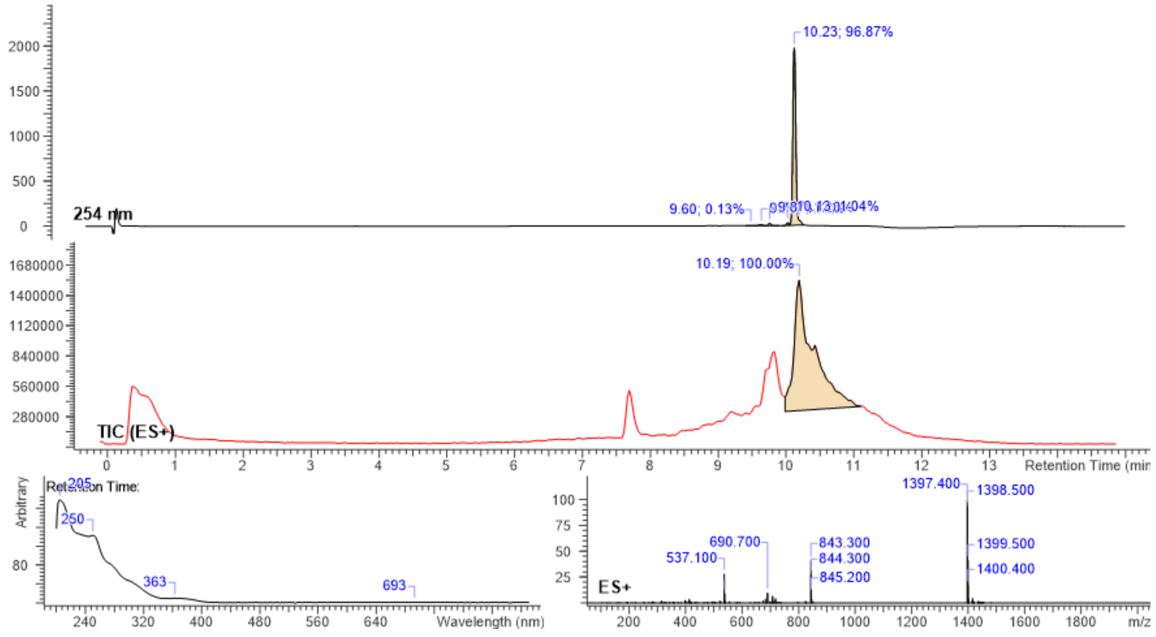

Supplementary Figure 67. LC/MS analysis results of compound **65**.

4-700SiR-CTX (**66**):

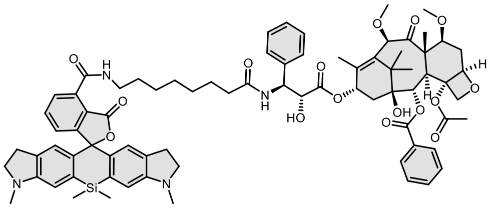

Compound was purified by preparative HPLC (solvent A: H_2O +0.2% HCOOH, solvent B: MeOH; temperature 25 °C, gradient A:B - 3 min 30:70 isocratic, 4-20 min 30:70 to 0:100 gradient, 20-25 min 0:100 isocratic, 40 mL/min flow). Fractions containing the product were collected, evaporated and lyophilized from acetonitrile water mixture. The obtained solid was dissolved in 700 µL of d_6-DMSO. The samples from DMSO solution were diluted x100 in PBS +0.1% SDS and concentration was determined spectroscopically with Nanodrop. Determined stock concentration was 3.3 mM which constitutes to 46% yield (3.2 mg).

^{1}H NMR (400 MHz, d_6-DMSO) δ 9.03 (t, J = 5.5 Hz, 1H), 8.38 (d, J = 9.0 Hz, 1H), 7.99 – 7.94 (m, 2H), 7.73 – 7.64 (m, 3H), 7.58 (t, J = 7.5 Hz, 2H), 7.33 (ddd, J = 15.2, 8.3, 6.9 Hz, 4H), 7.19 (tt, J = 7.1, 1.5 Hz, 1H), 7.14 (dd, J = 6.8, 1.9 Hz, 1H), 6.76 (s, 2H), 6.56 (s, 2H), 5.98 – 5.90 (m, 2H), 5.37 (d, J = 7.1 Hz, 1H), 5.28 (dd, J = 9.0, 5.8 Hz, 1H), 4.94 (d, J = 9.5 Hz, 1H), 4.69 (s, 1H), 4.63 (s, 1H), 4.41 (t, J = 6.3 Hz, 1H), 4.01 (s, 2H), 3.74 (dd, J = 10.6, 6.6 Hz, 1H), 3.62 (d, J = 7.1 Hz, 1H), 3.30 – 3.25 (m, 5H), 3.24 – 3.14 (m, 7H), 2.83 – 2.63 (m, 11H), 2.23 (s, 3H), 2.16 (t, J = 7.3 Hz, 2H), 1.99 – 1.82 (m, 5H), 1.59 – 1.45 (m, 8H), 1.38 – 1.24 (m, 6H), 1.02 (s, 3H), 0.96 (s, 3H), 0.56 (s, 3H), 0.47 (s, 3H). ESI-MS, positive mode: m/z = 1377.6 $[M+Na]^+$. HRMS (ESI) calcd for $C_{77}H_{91}N_4O_{16}Si$ $[M+H]^+$ 1355.6194, found 1355.6190.

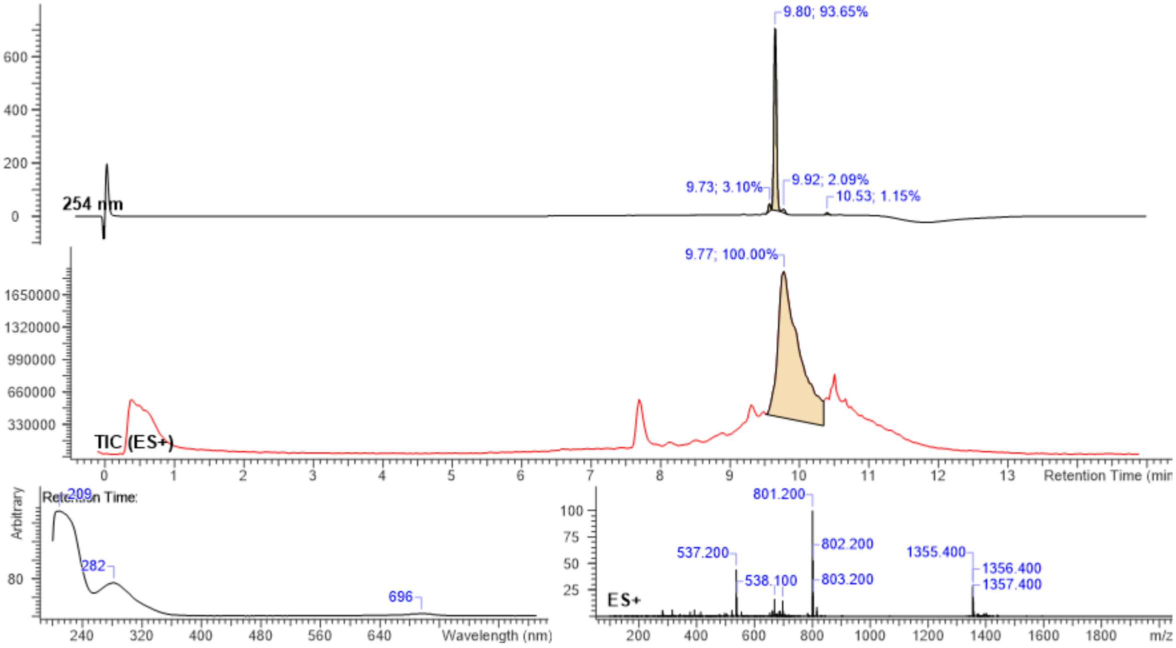

Supplementary Figure 68. LC/MS analysis results of compound **66**.

4-720SiR-CTX (**67**):

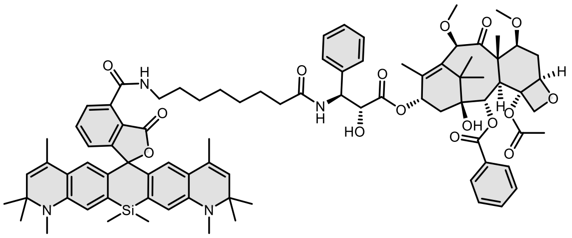

Compound was purified by preparative HPLC (solvent A: H_2O +0.2% HCOOH, solvent B: MeOH; temperature 25 °C, gradient A:B - 3 min 30:70isocratic, 4-20 min 30:70 to 0:100 gradient, 20-25 min 0:100 isocratic, 40 mL/min flow). Fractions containing the product were collected, evaporated and lyophilized from acetonitrile water mixture to give product in 49% yield (3.6 mg).

^{1}H NMR (400 MHz, d_6-DMSO) δ 9.02 (t, J = 5.5 Hz, 1H), 8.39 (d, J = 9.0 Hz, 1H), 8.01 – 7.95 (m, 2H), 7.87 – 7.80 (m, 2H), 7.67 (tt, J = 7.3, 1.3 Hz, 1H), 7.59 (t, J = 7.4 Hz, 2H), 7.44 (t, J = 4.4 Hz, 1H), 7.40 – 7.28 (m, 4H), 7.20 (tt, J = 7.0, 1.6 Hz, 1H), 6.76 (s, 2H), 6.41 (s, 2H), 5.99 – 5.91 (m, 2H), 5.39 (d, J = 7.1 Hz, 1H), 5.34 (d, J = 1.5 Hz, 2H), 5.28 (dd, J = 9.0, 5.8 Hz, 1H), 4.95 (dd, J = 9.6, 1.8 Hz, 1H), 4.70 (s, 1H), 4.65 (s, 1H), 4.42 (t, J = 6.2 Hz, 1H), 4.02 (s, 2H), 3.75 (dd, J = 10.6, 6.6 Hz, 1H), 3.63 (d, J = 7.3 Hz, 1H), 3.31 – 3.24 (m, 5H), 3.21 (s, 3H), 2.81 (s, 6H), 2.69 – 2.64 (m, 1H), 2.24 (s, 3H), 2.19 – 2.13 (m, 2H), 1.98 – 1.82 (m, 5H), 1.57 (d, J = 1.1 Hz, 6H), 1.55 – 1.39 (m, 8H), 1.36 – 1.21 (m, 18H), 1.03 (s, 3H), 0.97 (s, 3H), 0.63 (s, 3H), 0.50 (s, 3H). ESI-MS, positive mode: m/z = 1464.7 $[M+H]^+$. HRMS (ESI) calcd for $C_{85}H_{103}N_4O_{16}Si$ $[M+H]^+$ 1463.7133, found 1463.7131.

LC/MS:

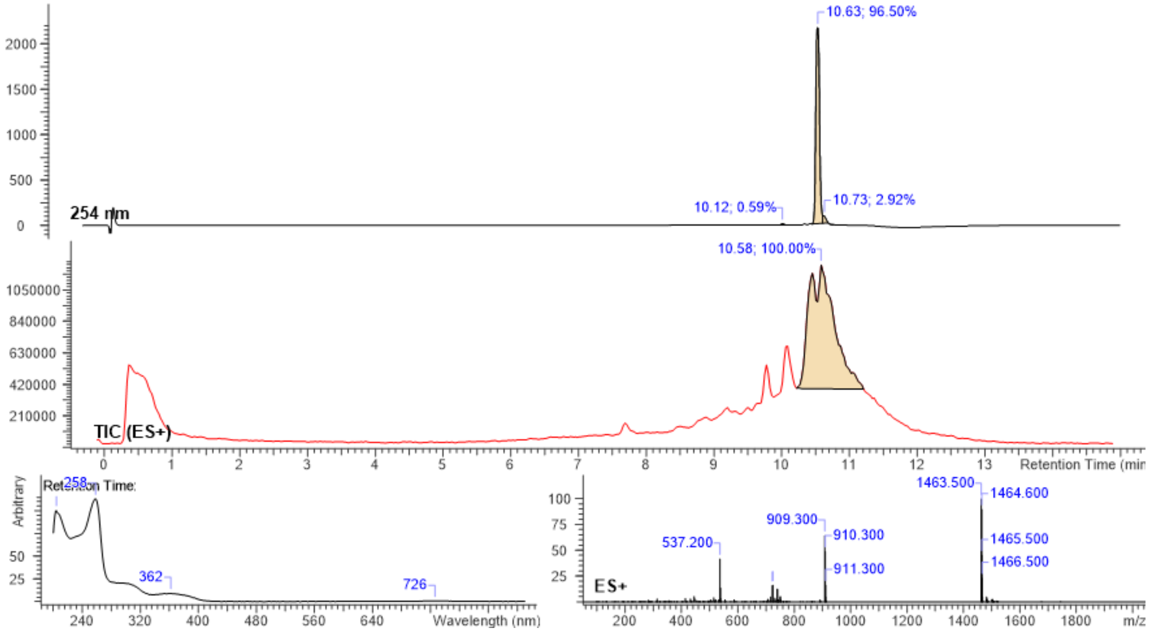

Supplementary Figure 69. LC/MS analysis results of compound **67**.

General procedure for the synthesis of compounds **68**-**79**:

Into a solution of corresponding rhodamine dye (5 µmol, 1 eq; **2**, **4** and **7-16**) and DIPEA (5 µL) in DMSO (100 µL) a solution of HATU (6.5 µmol, 1.3 eq) in DMSO (100 µL) was added and the mixture was mixed for 1 min. Then a solution of **Cl-$(CH_2)_6$-PEG_4-NH_2**[27] (7.5 µmol ,1.5 eq) in DMSO (100 µL) was added at once. Reactions were usually over after 30 min and their course was monitored by LC/MS analysis. Once reaction was finished it was quenched with 20 µL of formic acid and diluted with water and acetonitrile to 2 mL volume and was further purified by the means of preparative HPLC (preparative column: Agilent 5 Prep-C18, 5 µm, 100 x 50 mm).

4-610CP-Halo (**68**):

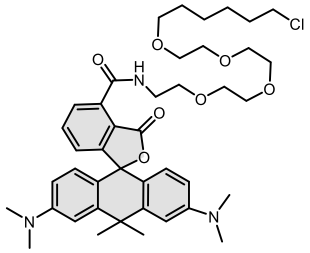

Compound was purified by preparative HPLC (solvent A: H_2O +0.2% HCOOH, solvent B: MeOH; temperature 25 °C, gradient A:B - 3 min 70:30 isocratic, 4-20 min 70:30 to 0:100 gradient, 20-25 min 0:100 isocratic, 40 mL/min flow). Fractions containing the product were collected, evaporated and lyophilized from acetonitrile water mixture. The obtained solid was dissolved in 700 µL of d_6-DMSO. The samples from DMSO solution were diluted x100 in PBS +0.1% SDS and concentration was determined spectroscopically with Nanodrop. Determined stock concentration was 4.8 mM which constitutes to 68% yield (2.5 mg).

^{1}H NMR (400 MHz, CD_3OD) δ 8.10 (dd, *J* = 7.6, 0.9 Hz, 1H), 7.76 (t, *J* = 7.7 Hz, 1H), 7.14 (dd, *J* = 7.8, 1.0 Hz, 1H), 7.01 – 6.96 (m, 2H), 6.66 – 6.53 (m, 4H), 3.81 – 3.47 (m, 18H), 3.40 (t, *J* = 6.5 Hz, 2H), 2.99 (s, 12H), 1.88 (s, 3H), 1.80 – 1.66 (m, 5H), 1.57 – 1.49 (m, 2H), 1.45 – 1.32 (m, 4H). ESI-MS, positive mode: m/z = 750.4 $[M+H]^+$. HRMS (ESI) calcd for $C_{42}H_{57}ClN_3O_7$ $[M+H]^+$ 750.3880, found 750.3885.

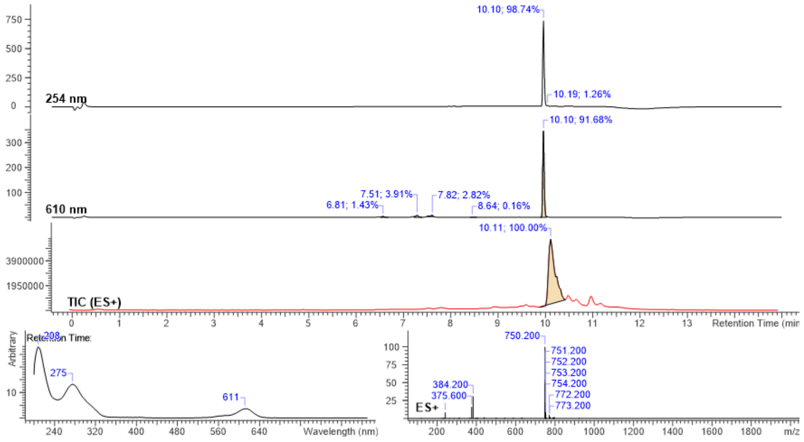

Supplementary Figure 70. LC/MS analysis results of compound **68**.

4-625CP-Halo (69):

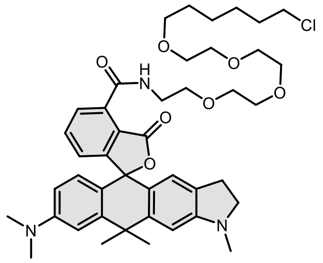

Compound was purified by preparative HPLC (solvent A: H_2O +0.2% HCOOH, solvent B: MeOH; temperature 25 °C, gradient A:B - 3 min 70:30 isocratic, 4-20 min 70:30 to 0:100 gradient, 20-25 min 0:100 isocratic, 40 mL/min flow). Fractions containing the product were collected, evaporated and lyophilized from acetonitrile water mixture. The obtained solid was dissolved in 700 µL of d_6-DMSO. The samples from DMSO solution were diluted x100 in PBS +0.1% SDS and concentration was determined spectroscopically with Nanodrop. Determined stock concentration was 4.4 mM which constitutes to 62% yield (2.4 mg).

^{1}H NMR (400 MHz, , d_6-DMSO) δ 9.34 (t, *J* = 5.5 Hz, 1H), 7.81 (dd, *J* = 7.5, 0.9 Hz, 1H), 7.70 (t, *J* = 7.6 Hz, 1H), 7.05 (dd, *J* = 7.7, 1.0 Hz, 1H), 6.89 (d, *J* = 2.4 Hz, 1H), 6.76 (s, 1H), 6.57 (dd, *J* = 8.9, 2.4 Hz, 1H), 6.53 (d, *J* = 8.8 Hz, 1H), 6.37 (s, 1H), 3.71 – 3.40 (m, 18H), 3.35 (t, *J* = 6.5 Hz, 2H), 3.28 – 3.19 (m, 2H), 2.93 (s, 6H), 2.79 (s, 3H), 2.77 – 2.66 (m, 2H), 1.79 (s, 3H), 1.74 – 1.63 (m, 5H), 1.47 (p, *J* = 6.7 Hz, 2H), 1.39 – 1.26 (m, 4H). ESI-MS, positive mode: m/z = 784.4 $[M+Na]^+$. HRMS (ESI) calcd for $C_{43}H_{56}ClN_3O_7Na$ $[M+Na]^+$ 784.3699, found 784.3687.

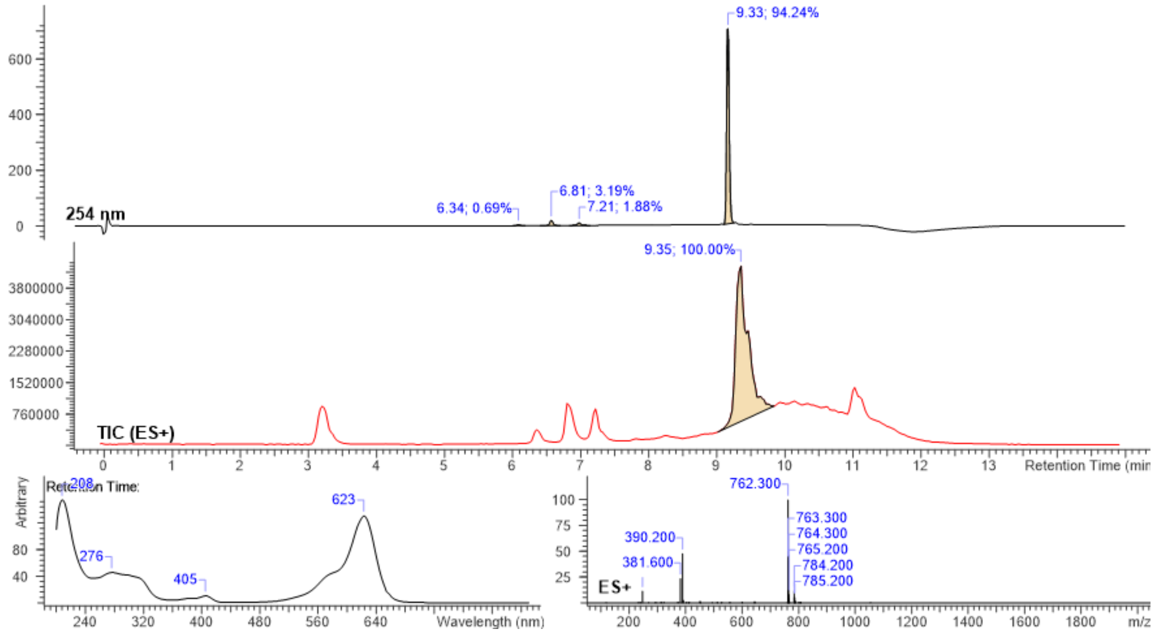

Supplementary Figure 71. LC/MS analysis results of compound **69**.

4-630CP-Halo (**70**):

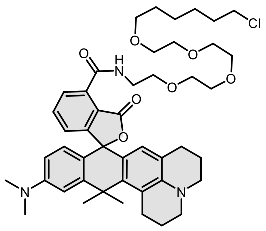

Compound was purified by preparative HPLC (solvent A: H_2O + 10mM NH_4COOH pH = 3.6, solvent B: MeOH; temperature 25 °C, gradient A:B - 3 min 70:30 isocratic, 4-20 min 70:30 to 0:100 gradient, 20-25 min 0:100 isocratic, 40 mL/min flow). Fractions containing the product were collected, evaporated and lyophilized from acetonitrile water mixture. The obtained solid was dissolved in 700 µL of d_6-DMSO. The samples from DMSO solution were diluted x100 in PBS +0.1% SDS and concentration was determined spectroscopically with Nanodrop. Determined stock concentration was 3.0 mM which constitutes to 42% yield (1.7 mg).

^{1}H NMR (400 MHz, d_6-DMSO) δ 9.38 (t, J = 5.5 Hz, 1H), 7.81 (dd, J = 7.5, 1.0 Hz, 1H), 7.68 (t, J = 7.6 Hz, 1H), 7.02 (dd, J = 7.7, 1.0 Hz, 1H), 6.80 (d, J = 2.5 Hz, 1H), 6.56 (dd, J = 9.0, 2.5 Hz, 1H), 6.47 (d, J = 8.8 Hz, 1H), 6.14 (s, 1H), 3.87 – 3.37 (m, 18H), 3.35 (t, J = 6.5 Hz, 2H), 3.24 – 3.07 (m, 4H), 2.92 (s, 8H), 2.49 – 2.35 (m, 2H), 2.03 – 1.85 (m, 5H), 1.82 (s, 3H), 1.79 – 1.63 (m, 4H), 1.47 (p, J = 6.8 Hz, 2H), 1.39 – 1.24 (m, 4H). ESI-MS, positive mode: m/z = 824.4 $[M+Na]^+$. HRMS (ESI) calcd for $C_{46}H_{60}ClN_3O_7Na$ $[M+Na]^+$ 824.4012, found 824.3995.

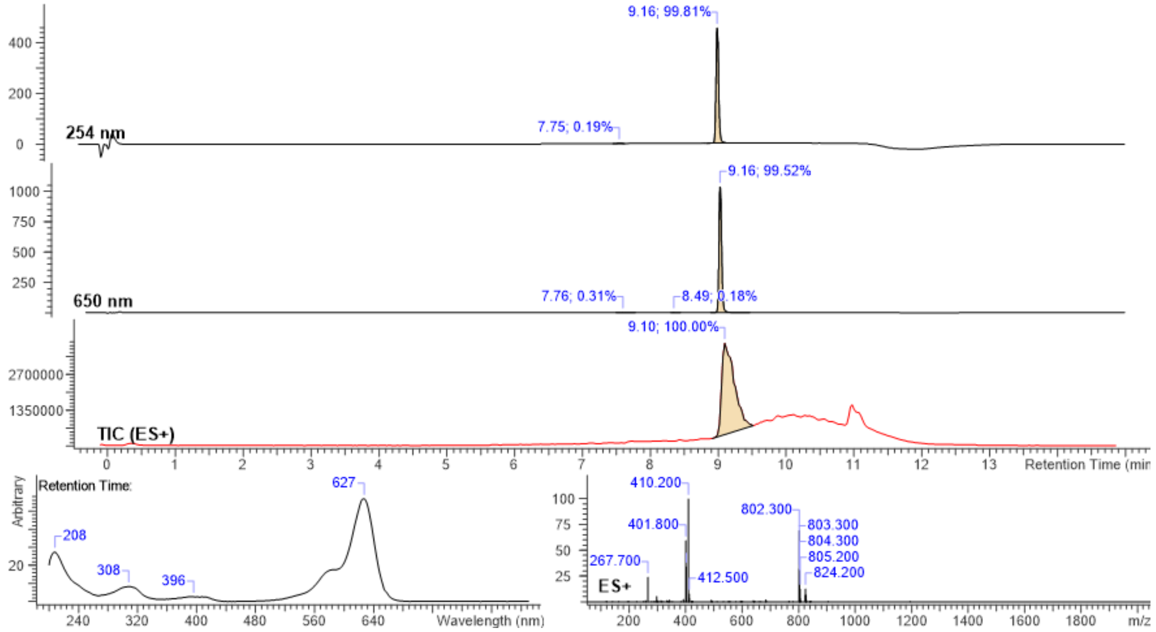

Supplementary Figure 72. LC/MS analysis results of compound **70**.

4-640CP-Halo (**71**):

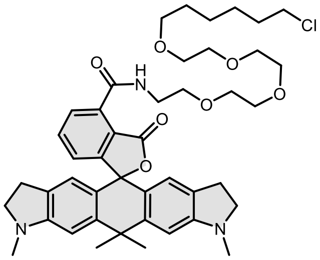

Compound was purified by preparative HPLC (solvent A: H_2O + 10mM NH_4COOH pH = 3.6, solvent B: MeOH; temperature 25 °C, gradient A:B - 3 min 60:40 isocratic, 4-20 min 60:40 to 0:100 gradient, 20-25 min 0:100 isocratic, 40 mL/min flow). Fractions containing the product were collected, evaporated and lyophilized from acetonitrile water mixture. The obtained solid was dissolved in 700 µL of d_6-DMSO. The samples from DMSO solution were diluted x100 in PBS +0.1% SDS and concentration was determined spectroscopically with Nanodrop. Determined stock concentration was 3.5 mM which constitutes to 47% yield (1.8 mg).

^{1}H NMR (400 MHz, d_6-DMSO) δ 9.36 (t, *J* = 5.5 Hz, 1H), 7.81 (dd, *J* = 7.5, 1.0 Hz, 1H), 7.69 (t, *J* = 7.6 Hz, 1H), 7.05 (dd, *J* = 7.7, 1.0 Hz, 1H), 6.74 (s, 2H), 6.34 (s, 2H), 3.73 – 3.38 (m, 18H), 3.35 (t, *J* = 6.5 Hz, 2H), 3.27 – 3.18 (m, 4H), 2.78 (s, 6H), 2.75 – 2.62 (m, 4H), 1.76 (s, 3H), 1.73 – 1.61 (m, 5H), 1.46 (p, *J* = 7.0 Hz, 2H), 1.38 – 1.28 (m, 4H). ESI-MS, positive mode: m/z = 796.4 $[M+Na]^+$. HRMS (ESI) calcd for $C_{44}H_{56}ClN_3O_7Na$ $[M+Na]^+$ 796.3699, found 796.3669.

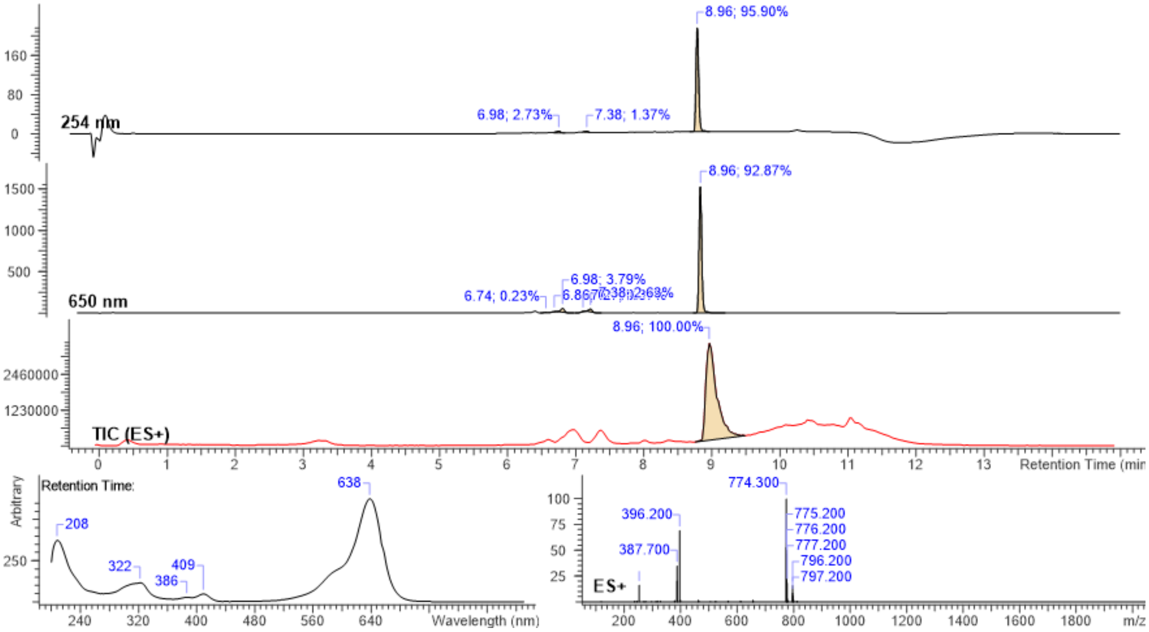

Supplementary Figure 73. LC/MS analysis results of compound **71**.

4-642CP-Halo (**72**):

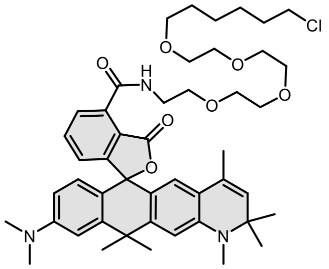

Compound was purified by preparative HPLC (solvent A: H_2O + 10mM NH_4COOH pH = 3.6, solvent B: MeOH; temperature 25 °C, gradient A:B - 3 min 30:70 isocratic, 4-20 min 30:70 to 0:100 gradient, 20-25 min 0:100 isocratic, 40 mL/min flow). Fractions containing the product were collected, evaporated and lyophilized from acetonitrile water mixture. The obtained solid was dissolved in 700 µL of d_6-DMSO. The samples from DMSO solution were diluted x100 in PBS +0.1% SDS and concentration was determined spectroscopically with Nanodrop. Determined stock concentration was 4.7 mM which constitutes to 66% yield (2.7 mg).

^{1}H NMR (400 MHz, d_6-DMSO) δ 9.38 (t, J = 5.5 Hz, 1H), 7.88 (dd, J = 7.6, 1.0 Hz, 1H), 7.74 (t, J = 7.7 Hz, 1H), 7.12 (dd, J = 7.7, 1.0 Hz, 1H), 6.91 (s, 1H), 6.67 (s, 1H), 6.56 (s, 2H), 6.16 (s, 1H), 5.33 (d, J = 1.6 Hz, 1H), 3.65 – 3.42 (m, 18H), 3.35 (t, J = 6.5 Hz, 2H), 2.94 (s, 6H), 2.84 (s, 3H), 1.81 (s, 3H), 1.73 – 1.64 (m, 5H), 1.55 (d, J = 1.4 Hz, 3H), 1.47 (p, J = 6.7 Hz, 2H), 1.38 – 1.25 (m, 10H). ESI-MS, positive mode: m/z = 838.4 $[M+Na]^+$. HRMS (ESI) calcd for $C_{47}H_{62}ClN_3O_7Na$ $[M+Na]^+$ 838.4169, found 838.4147.

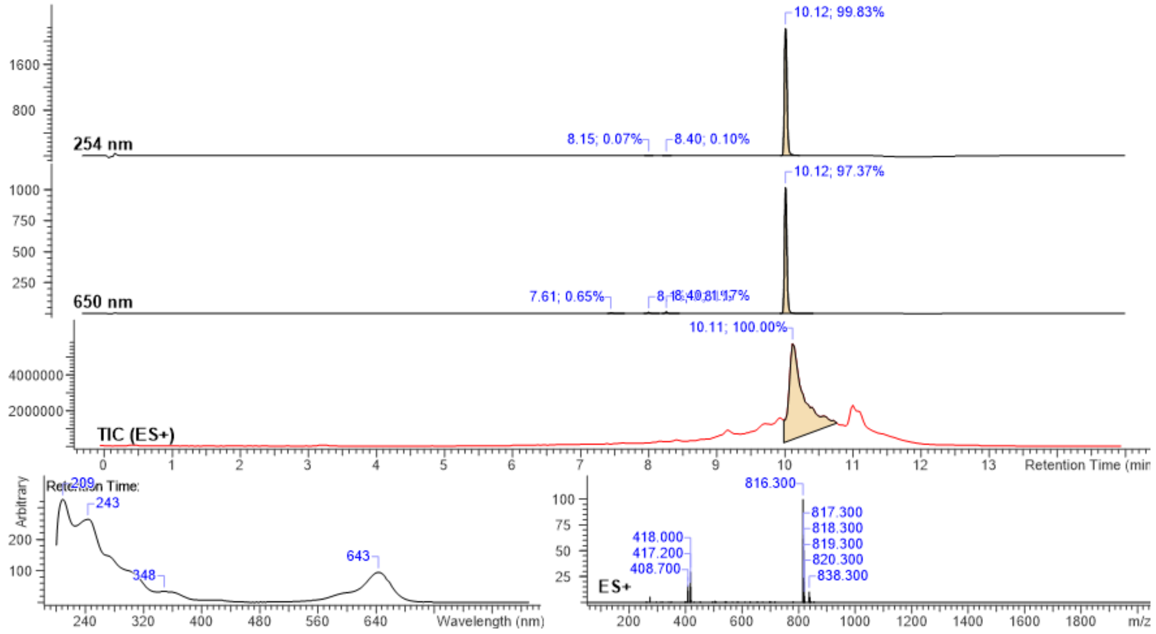

Supplementary Figure 74. LC/MS analysis results of compound **72**.

4-SiR-Halo (**73**):

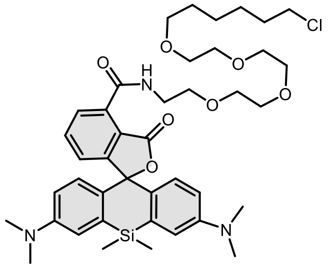

Compound was purified by preparative HPLC (solvent A: H_2O + 10mM NH_4COOH pH = 3.6, solvent B: MeOH; temperature 25 °C, gradient A:B - 3 min 30:70 isocratic, 4-20 min 30:70 to 0:100 gradient, 20-25 min 0:100 isocratic, 40 mL/min flow). Fractions containing the product were collected, evaporated and lyophilized from acetonitrile water mixture to give product in 70% yield (2.7 mg) as a light blue solid.

^{1}H NMR (400 MHz, d_6-DMSO) δ 9.22 (t, *J* = 5.5 Hz, 1H), 7.82 – 7.74 (m, 2H), 7.26 (dd, *J* = 7.2, 1.5 Hz, 1H), 7.00 (d, *J* = 2.7 Hz, 2H), 6.69 (d, *J* = 9.0 Hz, 2H), 6.64 (dd, *J* = 9.0, 2.8 Hz, 2H), 3.72 – 3.37 (m, 18H), 3.35 (t, *J* = 6.6 Hz, 2H), 2.92 (s, 12H), 1.68 (p, *J* = 6.7 Hz, 2H), 1.46 (p, *J* = 6.8 Hz, 2H), 1.35 – 1.21 (m, 4H), 0.62 (s, 3H), 0.52 (s, 3H). ESI-MS, positive mode: m/z = 788.3 $[M+Na]^+$. HRMS (ESI) calcd for $C_{41}H_{56}ClN_3O_7SiNa$ $[M+Na]^+$ 788.3468, found 788.3463.

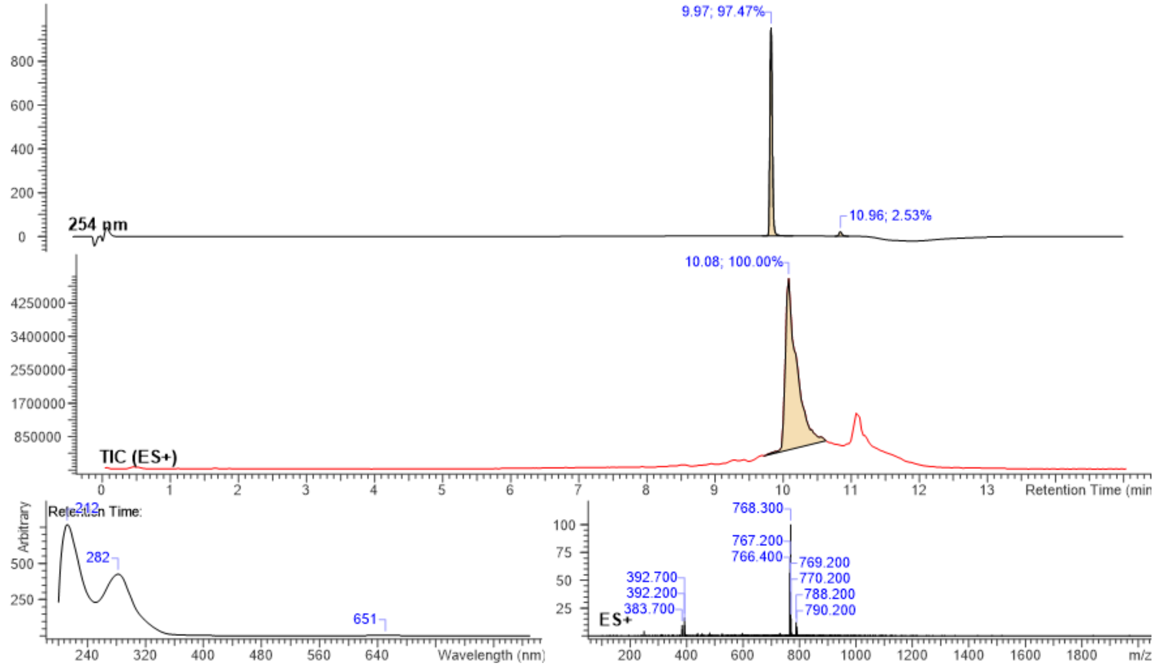

Supplementary Figure 75. LC/MS analysis results of compound **73**.

4-665SiR-Halo (**74**):

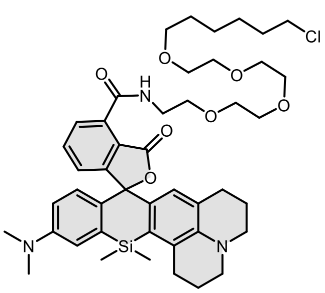

Compound was purified by preparative HPLC (solvent A: H_2O + 10mM NH_4COOH pH = 3.6, solvent B: MeOH; temperature 25 °C, gradient A:B - 3 min 30:70 isocratic, 4-20 min 30:70 to 0:100 gradient, 20-25 min 0:100 isocratic, 40 mL/min flow). Fractions containing the product were collected, evaporated and lyophilized from acetonitrile water mixture. The obtained solid was dissolved in 700 µL of d_6-DMSO. The samples from DMSO solution were diluted x100 in PBS +0.1% SDS and concentration was determined spectroscopically with Nanodrop. Determined stock concentration was 2.5 mM which constitutes to 35% yield (1.4 mg).

^{1}H NMR (400 MHz, *d$_6$*-DMSO) δ 9.26 (t, *J* = 5.5 Hz, 1H), 7.75 (d, *J* = 1.1 Hz, 1H), 7.71 (t, *J* = 7.6 Hz, 1H), 7.13 (dd, *J* = 7.6, 1.2 Hz, 1H), 6.93 (d, *J* = 2.5 Hz, 1H), 6.69 – 6.63 (m, 2H), 6.33 (s, 1H), 3.68 – 3.40 (m, 18H), 3.35 (t, *J* = 6.5 Hz, 2H), 3.16 (t, *J* = 5.8 Hz, 2H), 3.12 (t, *J* = 5.1 Hz, 2H), 2.96 – 2.85 (m, 8H), 2.47 – 2.40 (m, 2H), 1.98 – 1.93 (m, 2H), 1.79 – 1.74 (m, 2H), 1.68 (p, *J* = 6.7 Hz, 2H), 1.46 (p, *J* = 7.0 Hz, 2H), 1.36 – 1.27 (m, 4H), 0.66 (s, 3H), 0.55 (s, 3H). ESI-MS, positive mode: m/z = 840.4 $[M+Na]^+$. HRMS (ESI) calcd for $C_{45}H_{60}ClN_3O_7SiNa$ $[M+Na]^+$ 840.3781, found 840.3779.

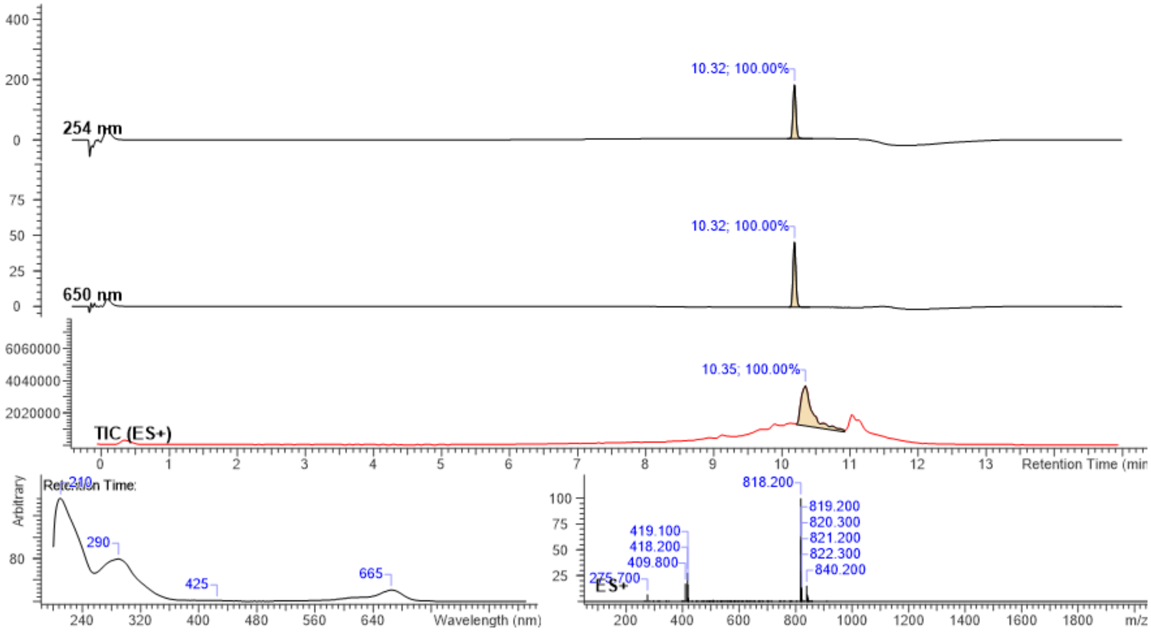

Supplementary Figure 76. LC/MS analysis results of compound **74**.

4-670SiR-Halo (**75**):

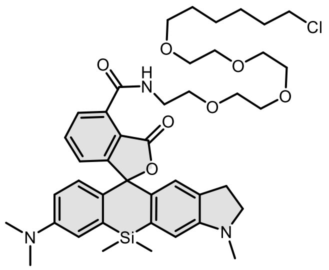

Compound was purified by preparative HPLC (solvent A: H_2O + 0.2% HCOOH, solvent B: MeOH; temperature 25 °C, gradient A:B - 3 min 40:60 isocratic, 4-20 min 40:60 to 0:100 gradient, 20-25 min 0:100 isocratic, 40 mL/min flow). Fractions containing the product were collected, evaporated and lyophilized from acetonitrile water mixture to give product in 45% yield (1.75 mg) as a colorless solid.

^{1}H NMR (400 MHz, d_6-DMSO) δ 9.24 (t, J = 5.5 Hz, 1H), 7.79 (dd, J = 7.5, 1.3 Hz, 1H), 7.75 (t, J = 7.4 Hz, 1H), 7.21 (dd, J = 7.3, 1.4 Hz, 1H), 6.98 (d, J = 2.7 Hz, 1H), 6.80 (s, 1H), 6.69 (d, J = 9.0 Hz, 1H), 6.64 (dd, J = 9.1, 2.8 Hz, 1H), 6.57 (s, 1H), 3.62 – 3.42 (m, 18H), 3.35 (t, J = 6.5 Hz, 2H), 3.26 – 3.18 (m, 2H), 2.91 (s, 6H), 2.82 – 2.70 (m, 5H), 1.69 (p, J = 6.7 Hz, 2H), 1.46 (p, J = 6.8 Hz, 2H), 1.32 – 1.20 (m, 4H), 0.60 (s, 3H), 0.50 (s, 3H). ESI-MS, positive mode: m/z = 800.3 [M+Na]$^+$. HRMS (ESI) calcd for $C_{42}H_{56}ClN_3O_7SiNa$ [M+Na]$^+$ 800.3468, found 800.3464.

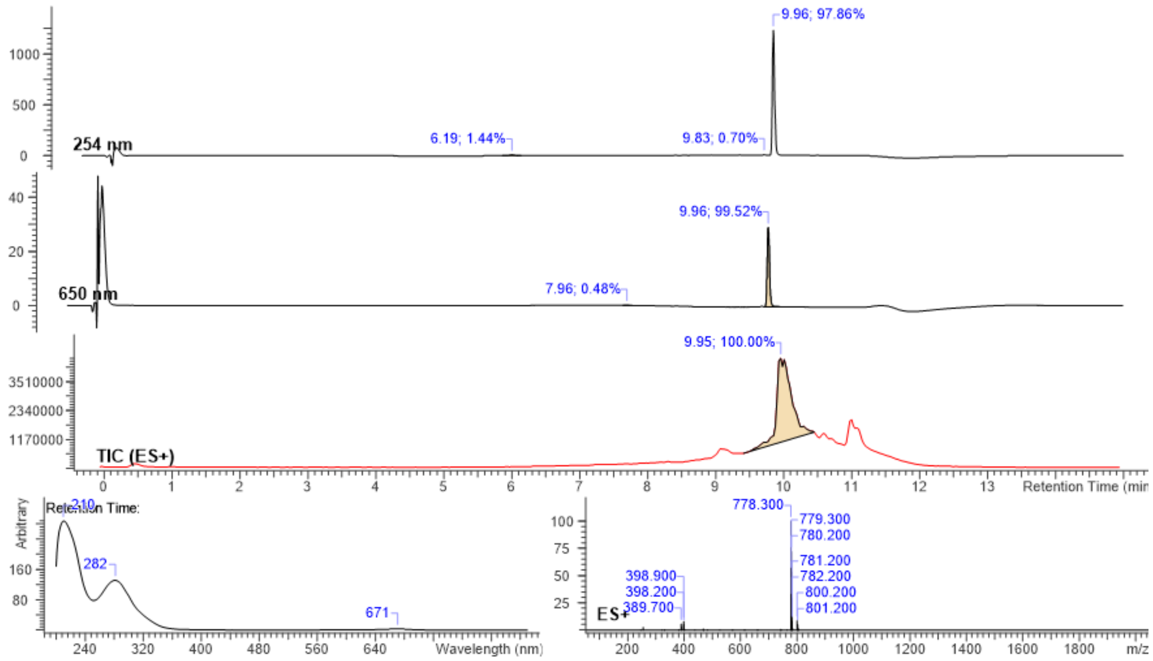

Supplementary Figure 77. LC/MS analysis results of compound **75**.

4-685SiR-Halo (**76**):

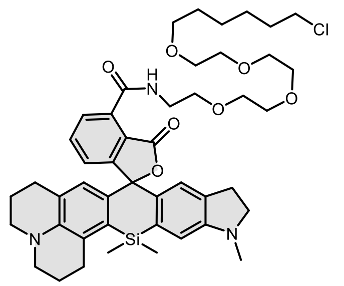

Compound was purified by preparative HPLC (solvent A: H_2O + 0.2% HCOOH pH = 3.6, solvent B: MeOH; temperature 25 °C, gradient A:B - 3 min 40:60 isocratic, 4-20 min 40:60 to 0:100 gradient, 20-25 min 0:100 isocratic, 40 mL/min flow). Fractions containing the product were collected, evaporated and lyophilized from acetonitrile water mixture. The obtained solid was dissolved in 700 µL of d_6-DMSO. The samples from DMSO solution were diluted x100 in PBS +0.1% SDS and concentration was determined spectroscopically with Nanodrop. Determined stock concentration was 3.4 mM which constitutes to 48% yield (2.0 mg).

^{1}H NMR (400 MHz, d_6-DMSO) δ 9.29 (t, J = 5.5 Hz, 1H), 7.75 (dd, J = 7.4, 0.9 Hz, 1H), 7.69 (t, J = 7.6 Hz, 1H), 7.09 (dd, J = 7.6, 1.0 Hz, 1H), 6.74 (s, 1H), 6.54 (s, 1H), 6.31 (s, 1H), 3.63 – 3.42 (m, 18H), 3.34 (t, J = 6.8 Hz, 2H), 3.23 – 3.15 (m, 4H), 3.13 – 3.08 (m, 2H), 2.90 (t, J = 6.4 Hz, 2H), 2.76 (s, 5H), 2.48 – 2.38 (m, 2H), 1.94 (p, J = 6.3 Hz, 2H), 1.80 – 1.72 (m, 2H), 1.67 (p, J = 6.9 Hz, 2H), 1.46 (p, J = 6.8 Hz, 2H), 1.35 – 1.24 (m, 4H), 0.64 (s, 3H), 0.53 (s, 3H). ESI-MS, positive mode: m/z = 852.4 $[M+Na]^+$. HRMS (ESI) calcd for $C_{46}H_{60}ClN_3O_7SiNa$ $[M+Na]^+$ 852.3781, found 852.3770.

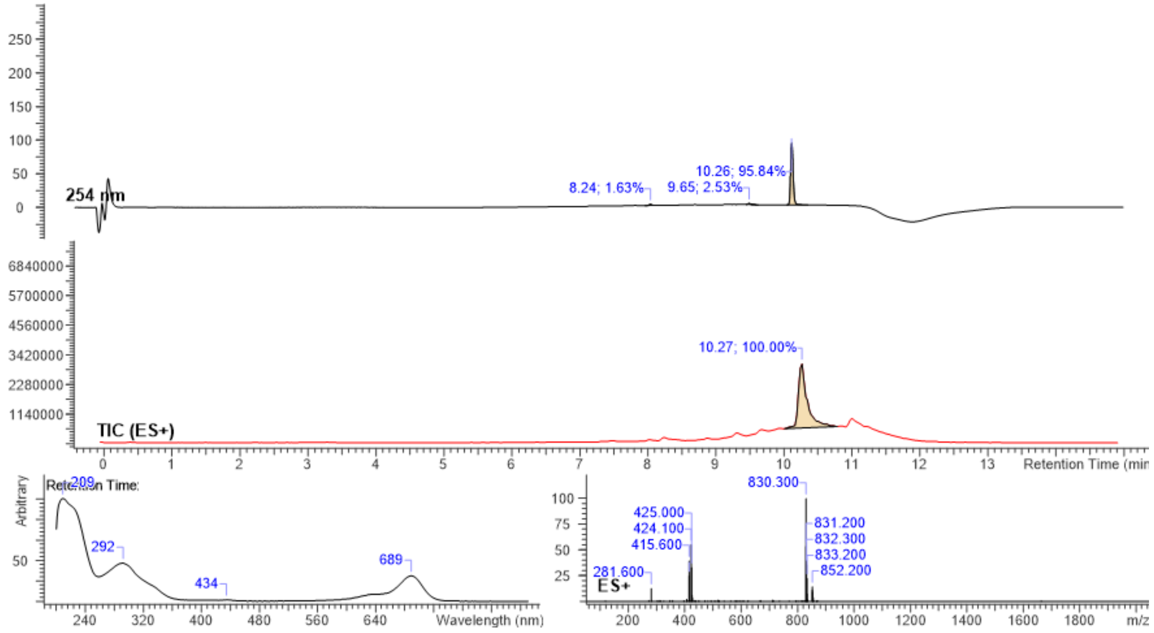

Supplementary Figure 78. LC/MS analysis results of compound **76**.

4-690SiR-Halo (**77**):

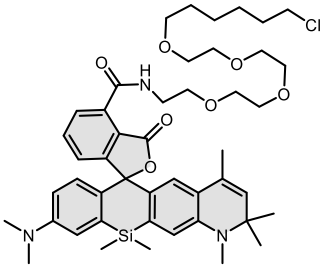

Compound was purified by preparative HPLC (solvent A: H_2O + 0.2% HCOOH, solvent B: MeOH; temperature 25 °C, gradient A:B - 3 min 40:60 isocratic, 4-20 min 40:60 to 0:100 gradient, 20-25 min 0:100 isocratic, 40 mL/min flow). Fractions containing the product were collected, evaporated and lyophilized from acetonitrile water mixture to give product in 67% yield (2.8 mg) as a colorless solid.

^{1}H NMR (400 MHz, d_6-DMSO) δ 9.22 (t, J = 5.5 Hz, 1H), 7.86 – 7.79 (m, 2H), 7.37 (dd, J = 7.1, 1.6 Hz, 1H), 7.01 (d, J = 2.8 Hz, 1H), 6.76 (s, 1H), 6.68 (d, J = 8.9 Hz, 1H), 6.61 (dd, J = 9.0, 2.8 Hz, 1H), 6.40 (s, 1H), 5.35 (d, J = 1.5 Hz, 1H), 3.65 – 3.40 (m, 18H), 3.35 (t, J = 6.5 Hz, 2H), 2.92 (s, 6H), 2.81 (s, 3H), 1.68 (p, J = 7.0, 6.5 Hz, 2H), 1.57 (d, J = 1.4 Hz, 3H), 1.46 (p, J = 7.0 Hz, 2H), 1.35 – 1.26 (m, 4H), 1.25 (s, 3H), 1.24 (s, 3H), 0.63 (s, 3H), 0.51 (s, 3H). ESI-MS, positive mode: m/z = 854.4 $[M+Na]^+$. HRMS (ESI) calcd for $C_{46}H_{62}ClN_3O_7SiNa$ $[M+Na]^+$ 854.3938, found 854.3935.

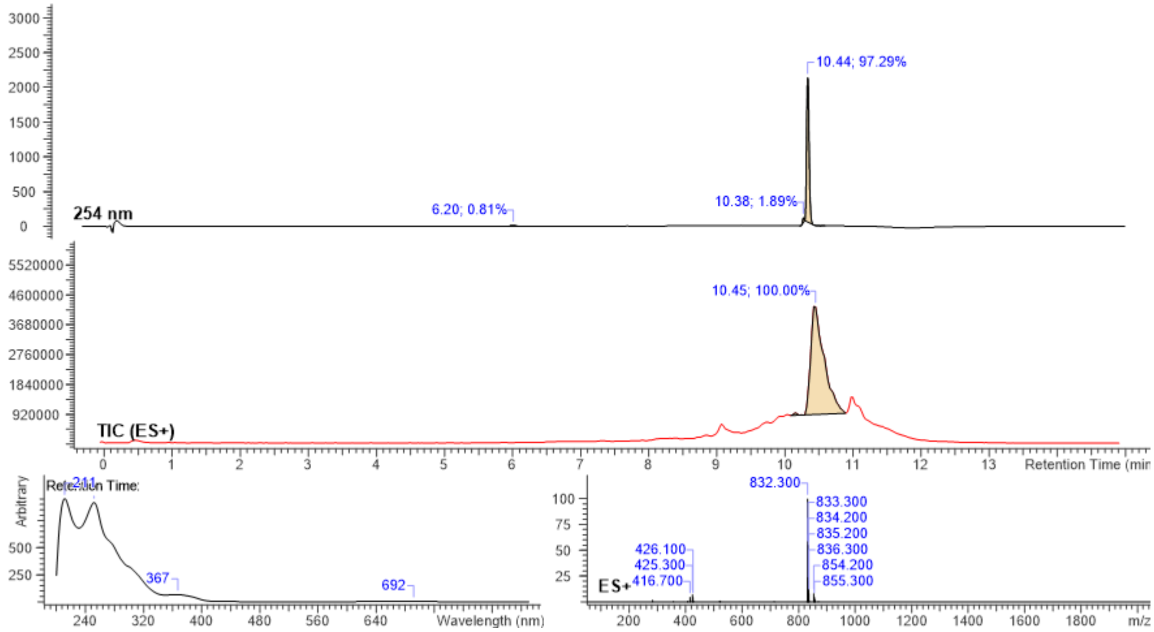

Supplementary Figure 79. LC/MS analysis results of compound **77**.

4-700SiR-Halo (**78**):

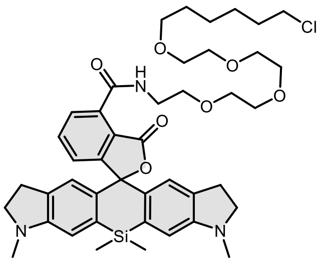

Compound was purified by preparative HPLC (solvent A: H_2O + 0.2% HCOOH pH = 3.6, solvent B: MeOH; temperature 25 °C, gradient A:B - 3 min 40:60 isocratic, 4-20 min 40:60 to 0:100 gradient, 20-25 min 0:100 isocratic, 40 mL/min flow). Fractions containing the product were collected, evaporated and lyophilized from acetonitrile water mixture. The obtained solid was dissolved in 700 µL of d_6-DMSO. The samples from DMSO solution were diluted x100 in PBS +0.1% SDS and concentration was determined spectroscopically with Nanodrop. Determined stock concentration was 2.7 mM which constitutes to 38% yield (1.5 mg).

^{1}H NMR (400 MHz, d_6-DMSO) δ 9.26 (t, J = 5.5 Hz, 1H), 7.77 (dd, J = 7.5, 1.2 Hz, 1H), 7.72 (t, J = 7.5 Hz, 1H), 7.16 (dd, J = 7.5, 1.2 Hz, 1H), 6.78 (s, 2H), 6.57 (s, 2H), 3.69 – 3.38 (m, 18H), 3.35 (t, J = 6.5 Hz, 2H), 3.27 – 3.17 (m, 4H), 2.87 – 2.65 (m, 10H), 1.68 (p, J = 6.8 Hz, 2H), 1.46 (p, J = 6.8 Hz, 2H), 1.34 – 1.23 (m, 4H), 0.57 (s, 3H), 0.49 (s, 3H). ESI-MS, positive mode: m/z = 812.3 $[M+Na]^+$. HRMS (ESI) calcd for $C_{43}H_{56}ClN_3O_7SiNa$ $[M+Na]^+$ 812.3468, found 812.3462.

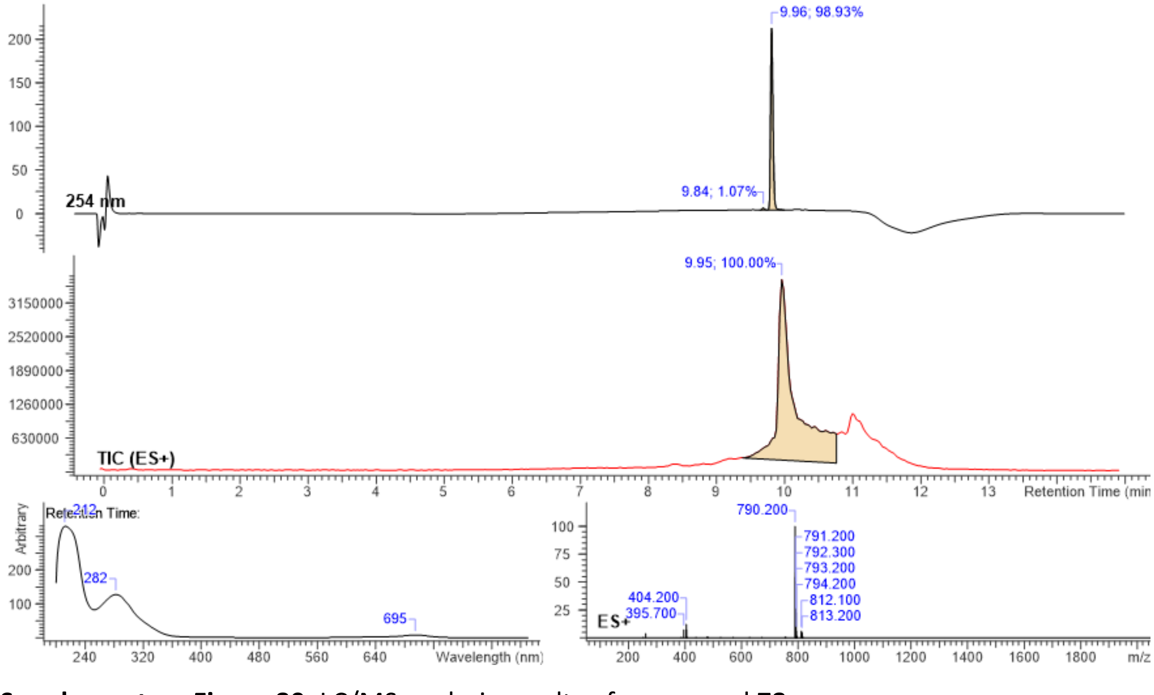

Supplementary Figure 80. LC/MS analysis results of compound **78**.

4-720SiR-Halo (**79**):

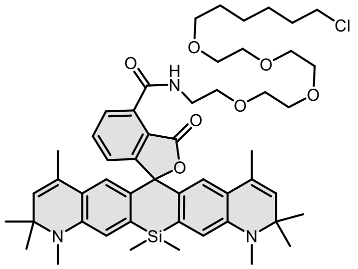

Compound was purified by preparative HPLC (solvent A: H_2O + 0.2% HCOOH, solvent B: MeOH; temperature 25 °C, gradient A:B - 3 min 40:60 isocratic, 4-20 min 40:60 to 0:100 gradient, 20-25 min 0:100 isocratic, 40 mL/min flow). Fractions containing the product were collected, evaporated and lyophilized from acetonitrile water mixture to give product in 58% yield (2.6 mg) as a colorless solid.

^{1}H NMR (400 MHz, d_6-DMSO) δ 9.23 (t, *J* = 5.5 Hz, 1H), 7.88 (dd, *J* = 7.6, 1.7 Hz, 1H), 7.85 (t, *J* = 7.5, 7.1 Hz, 1H), 7.47 (dd, *J* = 7.1, 1.6 Hz, 1H), 6.76 (s, 2H), 6.39 (s, 2H), 5.34 (d, *J* = 1.5 Hz, 2H), 3.61 – 3.41 (m, 18H), 3.34 (t, *J* = 6.8 Hz, 2H), 2.81 (s, 6H), 1.68 (p, *J* = 6.8 Hz, 2H), 1.57 (d, *J* = 1.4 Hz, 6H), 1.46 (p, *J* = 6.8 Hz, 2H), 1.35 – 1.26 (m, 4H), 1.25 (s, 6H), 1.24 (s, 6H), 0.63 (s, 3H), 0.50 (s, 3H). ESI-MS, positive mode: m/z = 920.4 [M+Na]$^+$. HRMS (ESI) calcd for $C_{51}H_{68}ClN_3O_7SiNa$ [M+H]$^+$ 898.4588, found 898.4570.

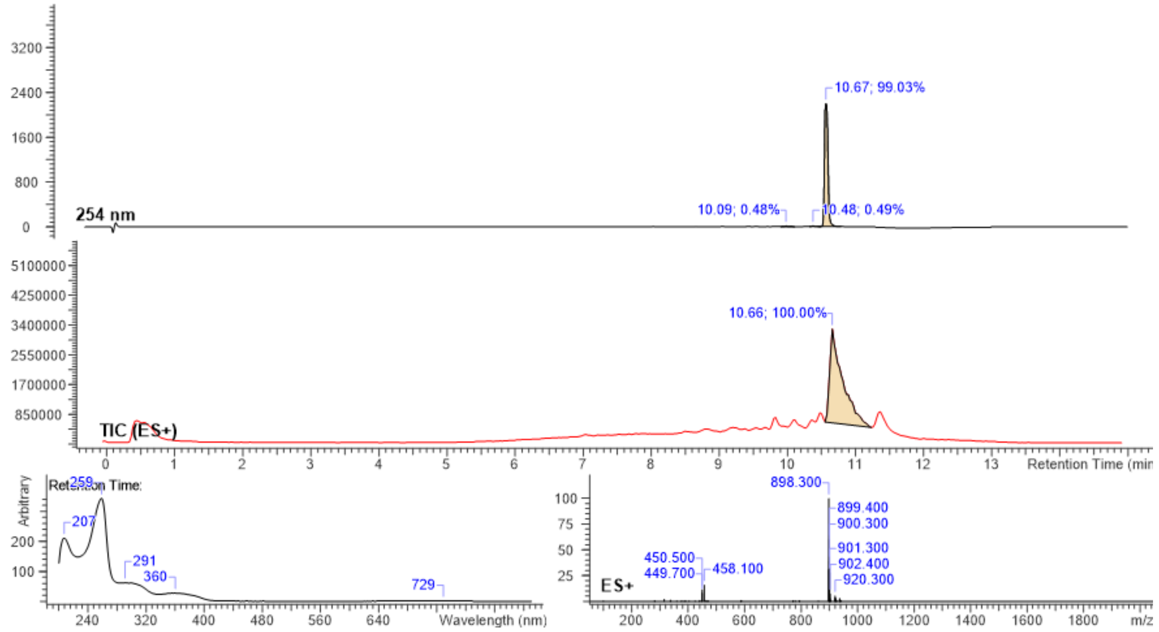

Supplementary Figure 81. LC/MS analysis results of compound **79**.

General procedure for the synthesis of compounds **80**-**86**:

Into a solution of corresponding rhodamine dye (5 µmol, 1 eq; **5**, **7, 8, 10, 13, 15, 23**) and DIPEA (5 µL) in DMSO (100 µL) a solution of HATU (6.5 µmol, 1.3 eq) in DMSO (100 µL) was added and a mixture was mixed for 1 min. Then a solution of (6-aminohexyl)triphenylphosphonium bromide hydrobromide (7.5 µmol ,1.5 eq, Sigma Aldrich) in DMSO (100 µL +5 µL DIPEA) was added at once. Reactions were usually over after 30 min and their course was monitored by LC/MS analysis. Once reaction was finished it was quenched with 20 µL of formic acid and diluted with water and acetonitrile to 2 mL volume and was further purified by the means of preparative HPLC (preparative column: Agilent 5 Prep-C18, 5 µm, 100 x 50 mm).

4-525R-TPP (80):

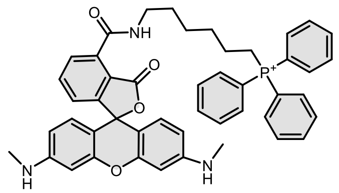

Compound was purified by preparative HPLC (solvent A: H_2O + 10mM NH_4COOH pH = 3.6, solvent B: MeCN; temperature 25 °C, gradient A:B - 3 min 70:30 isocratic, 4-20 min 70:30 to 0:100 gradient, 20-25 min 0:100 isocratic, 40 mL/min flow). Fractions containing the product were collected, evaporated and lyophilized from acetonitrile water mixture. The obtained solid was dissolved in 700 µL of d_6-DMSO. The samples from DMSO solution were diluted x100 in PBS +0.1% SDS and concentration was determined spectroscopically with Nanodrop. Determined stock concentration was 4.0 mM which constitutes to 56% yield (2.1 mg).

^{1}H NMR (400 MHz, CD_3OD) δ 7.87 – 7.74 (m, 11H), 7.69 – 7.61 (m, 6H), 7.37 (dd, *J* = 7.6, 1.2 Hz, 1H), 7.14 (d, *J* = 9.1 Hz, 2H), 6.76 (d, *J* = 2.2 Hz, 2H), 6.73 (dd, *J* = 9.2, 2.2 Hz, 2H), 3.45 – 3.36 (m, 4H), 3.01 (s, 6H), 1.67 – 1.51 (m, 8H). ESI-MS, positive mode: m/z = 746.3 $[M]^+$. HRMS (ESI) calcd for $C_{47}H_{45}N_3O_4P$ $[M]^+$ 746.3142, found 746.3141.

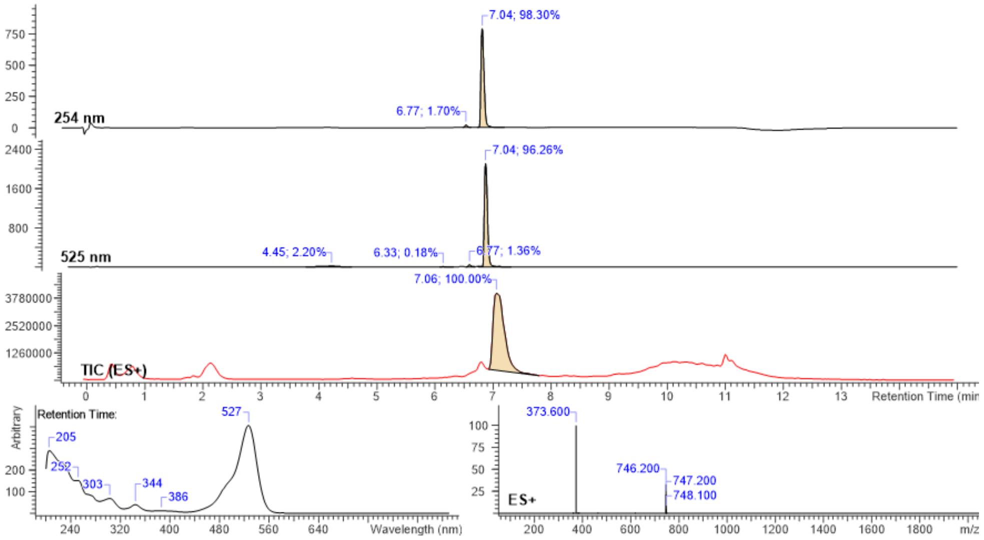

Supplementary Figure 82. LC/MS analysis results of compound **80**.

4-580R-TPP (81):

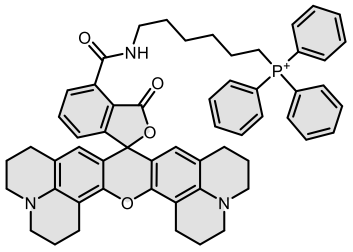

Compound was purified by preparative HPLC (solvent A: H_2O + 10mM NH_4COOH pH = 3.6, solvent B: MeCN; temperature 25°C, gradient A:B - 3 min 70:30 isocratic, 4-20 min 70:30 to 0:100 gradient, 20-25 min 0:100 isocratic, 40 mL/min flow). Fractions containing the product were collected, evaporated and lyophilized from acetonitrile water mixture. The obtained solid was dissolved in 700 µL of d_6-DMSO. The samples from DMSO solution were diluted x100 in PBS +0.1% SDS and concentration was determined spectroscopically with Nanodrop. Determined stock concentration was 1.9 mM which constitutes to 27% yield (1.2 mg).

^{1}H NMR (400 MHz, CD_3OD) δ 7.86 – 7.75 (m, 10H), 7.68 – 7.63 (m, 5H), 7.55 (t, *J* = 7.7 Hz, 1H), 7.30 (dd, *J* = 7.6, 1.3 Hz, 1H), 6.82 (s, 2H), 3.54 (t, *J* = 5.8 Hz, 4H), 3.50 – 3.44 (m, 4H), 3.43 – 3.33 (m, 4H), 3.06 (t, *J* = 6.2 Hz, 4H), 2.63 (q, *J* = 6.2 Hz, 4H), 2.09 (p, *J* = 6.3 Hz, 4H), 1.94 – 1.83 (m, 4H), 1.67 – 1.51 (m, 8H). ESI-MS, positive mode: m/z = 878.4 $[M]^+$. HRMS (ESI) calcd for $C_{57}H_{57}N_3O_4P$ $[M]^+$ 878.4081, found 878.4091.

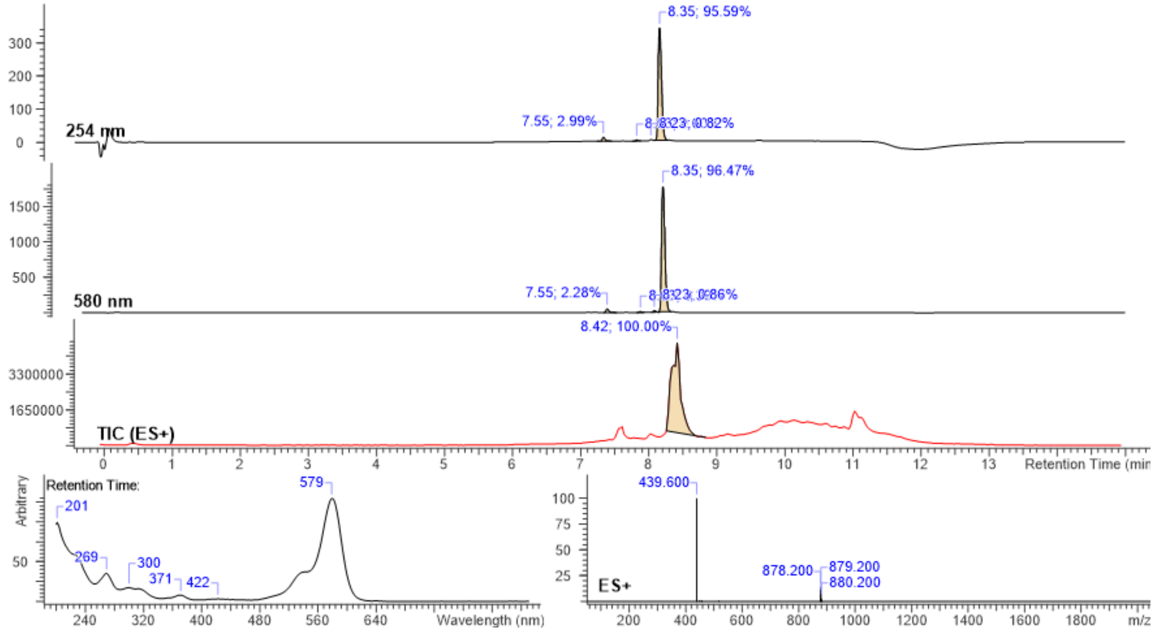

Supplementary Figure 83. LC/MS analysis results of compound **81**.

4-625-TPP (82):

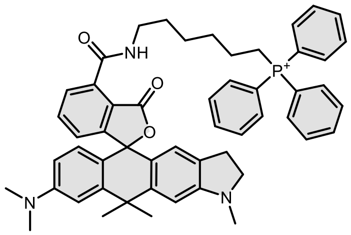

Compound was purified by preparative HPLC (solvent A: H_2O + 10mM NH_4COOH pH = 3.6, solvent B: MeCN; temperature 25°C, gradient A:B - 3 min 70:30 isocratic, 4-20 min 70:30 to 0:100 gradient, 20-25 min 0:100 isocratic, 40 mL/min flow). Fractions containing the product were collected, evaporated and lyophilized from acetonitrile water mixture. The obtained solid was dissolved in 700 µL of d_6-DMSO. The samples from DMSO solution were diluted x100 in PBS +0.1% SDS and concentration was determined spectroscopically with Nanodrop. Determined stock concentration was 2.4 mM which constitutes to 34% yield (1.4 mg).

^{1}H NMR (400 MHz, CD_3OD) δ 7.96 (dd, *J* = 7.6, 1.0 Hz, 1H), 7.80 – 7.71 (m, 10H), 7.65 – 7.58 (m, 6H), 7.15 (dd, *J* = 7.8, 1.0 Hz, 1H), 7.00 (d, *J* = 2.4 Hz, 1H), 6.81 (s, 1H), 6.59 (d, *J* = 8.8 Hz, 1H), 6.55 (dd, *J* = 8.9, 2.4 Hz, 1H), 6.41 (s, 1H), 3.52 – 3.41 (m, 4H), 3.36 – 3.32 (m, 2H), 3.01 (s, 6H), 2.88 (s, 3H), 2.72 (t, *J* = 8.2 Hz, 2H), 1.86 (s, 3H), 1.75 (s, 3H), 1.73 – 1.61 (m, 8H). ESI-MS, positive mode: m/z = 812.4 $[M]^+$. HRMS (ESI) calcd for $C_{53}H_{55}N_3O_3P$ $[M]^+$ 812.3976, found 812.3964.

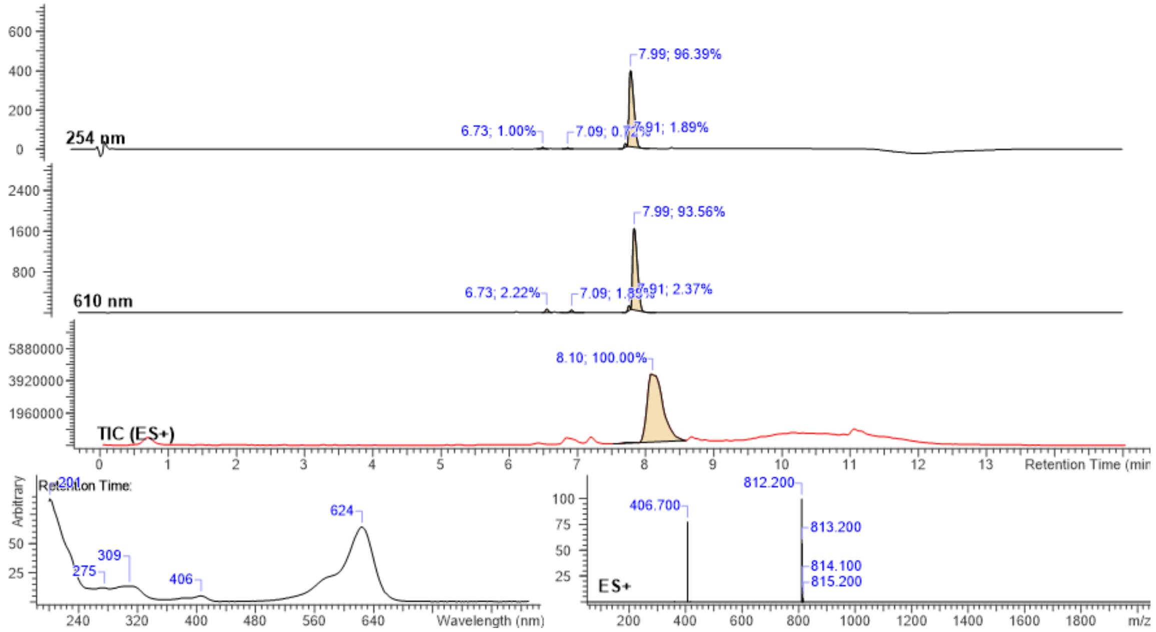

Supplementary Figure 84. LC/MS analysis results of compound **82**.

4-640-TPP (83):

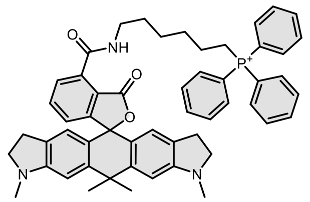

Compound was purified by preparative HPLC (solvent A: H_2O + 10 mM NH_4COOH pH = 3.6, solvent B: MeCN; temperature 25°C, gradient A:B - 3 min 40:60 isocratic, 4-20 min 40:60 to 0:100 gradient, 20-25 min 0:100 isocratic, 40 mL/min flow). Fractions containing the product were collected, evaporated and lyophilized from acetonitrile water mixture. The obtained solid was dissolved in 700 µL of d_6-DMSO. The samples from DMSO solution were diluted x100 in PBS +0.1% SDS and concentration was determined spectroscopically with Nanodrop. Determined stock concentration was 2.9 mM which constitutes to 41% yield (1.7 mg).

^1H NMR (400 MHz, CD_3OD) δ 7.90 (dd, *J* = 7.6, 1.0 Hz, 1H), 7.86 – 7.73 (m, 10H), 7.68 (t, *J* = 7.7 Hz, 1H), 7.65 – 7.60 (m, 5H), 7.15 (dd, *J* = 7.7, 1.1 Hz, 1H), 6.82 (s, 2H), 6.48 (s, 2H), 3.51 – 3.43 (m, 4H), 3.40 (td, *J* = 8.2, 2.3 Hz, 4H), 2.93 (s, 6H), 2.77 – 2.72 (m, 4H), 1.82 (s, 3H), 1.73 (s, 3H), 1.65 (s, 8H). ESI-MS, positive mode: m/z = 824.4 $[M]^+$. HRMS (ESI) calcd for $C_{54}H_{55}N_3O_3P$ $[M]^+$ 824.3976, found 824.3950.

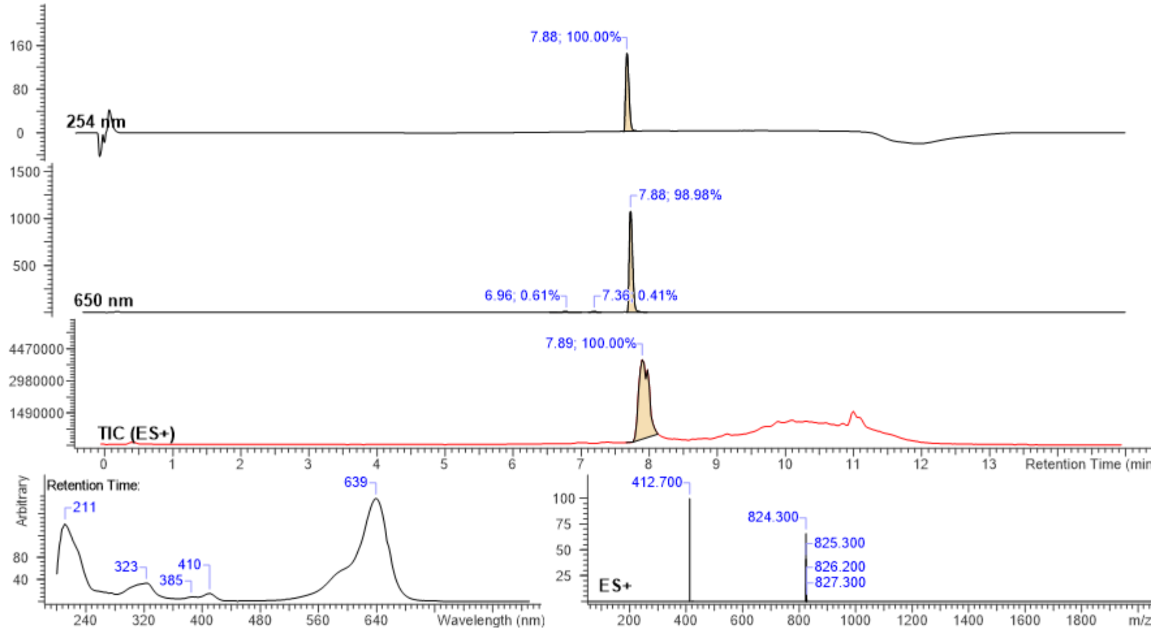

Supplementary Figure 85. LC/MS analysis results of compound **83**.

4-665SiR-TPP (84):

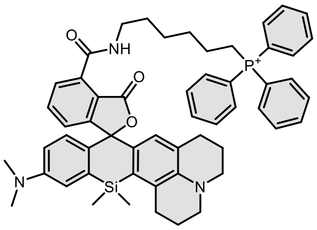

Compound was purified by preparative HPLC (solvent A: H_2O + 10 mM NH_4COOH pH = 3.6, solvent B: MeCN; temperature 25°C, gradient A:B - 3 min 40:60 isocratic, 4-20 min 40:60 to 0:100 gradient, 20-25 min 0:100 isocratic, 40 mL/min flow). Fractions containing the product were collected, evaporated and lyophilized from acetonitrile water mixture. The obtained solid was dissolved in 700 µL of d_6-DMSO. The samples from DMSO solution were diluted x100 in PBS +0.1% SDS and concentration was determined spectroscopically with Nanodrop. Determined stock concentration was 2.2 mM which constitutes to 31% yield (1.3 mg).

^{1}H NMR (400 MHz CD_3OD) δ 7.90 (dd, *J* = 7.6, 1.0 Hz, 1H), 7.82 – 7.73 (m, 11H), 7.65 – 7.60 (m, 5H), 7.27 (dd, *J* = 7.8, 1.0 Hz, 1H), 7.02 (d, *J* = 2.8 Hz, 1H), 6.68 (d, *J* = 8.9 Hz, 1H), 6.62 – 6.59 (m, 1H), 6.32 (s, 1H), 3.50 – 3.41 (m, 4H), 3.23 (t, *J* = 5.9 Hz, 2H), 3.17 (t, *J* = 5.8 Hz, 2H), 3.01 (dt, *J* = 8.2, 4.0 Hz, 2H), 2.97 (s, 6H), 2.42 (t, *J* = 6.4 Hz, 2H), 2.07 – 2.02 (m, 2H), 1.84 – 1.79 (m, 2H), 1.76 – 1.65 (m, 8H), 0.69 (s, 3H), 0.54 (s, 3H). ESI-MS, positive mode: m/z = 868.3 $[M]^+$. HRMS (ESI) calcd for $C_{55}H_{59}N_3O_3PSi$ $[M]^+$ 868.4058, found 868.4038.

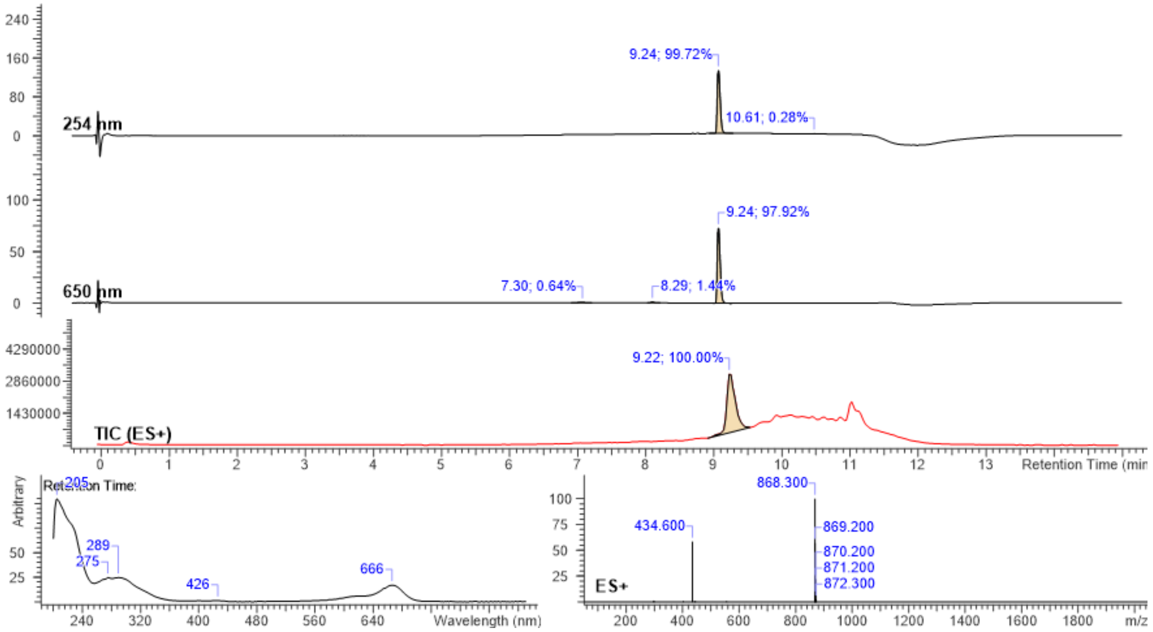

Supplementary Figure 86. LC/MS analysis results of compound **84**.

4-685SiR-TPP (85):

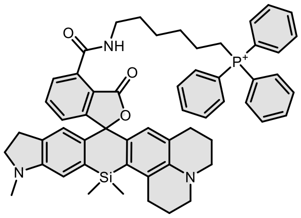

Compound was purified by preparative HPLC (solvent A: H_2O + 10 mM NH_4COOH pH = 3.6, solvent B: MeCN; temperature 25°C, gradient A:B - 3 min 40:60 isocratic, 4-20 min 40:60 to 0:100 gradient, 20-25 min 0:100 isocratic, 40 mL/min flow). Fractions containing the product were collected, evaporated and lyophilized from acetonitrile water mixture. The obtained solid was dissolved in 700 µL of d_6-DMSO. The samples from DMSO solution were diluted x100 in PBS +0.1% SDS and concentration was determined spectroscopically with Nanodrop. Determined stock concentration was 2.5 mM which constitutes to 35% yield (1.5 mg).

^{1}H NMR (400 MHz, CD_3OD) δ 7.85 (dd, *J* = 7.6, 0.9 Hz, 1H), 7.83 – 7.66 (m, 11H), 7.66 – 7.61 (m, 5H), 7.19 (dd, *J* = 7.8, 0.9 Hz, 1H), 6.77 (s, 1H), 6.55 (s, 1H), 6.33 (s, 1H), 3.50 – 3.43 (m, 4H), 3.28 – 3.21 (m, 4H), 3.18 – 3.14 (m, 2H), 3.00 (t, *J* = 6.3 Hz, 2H), 2.82 (s, 6H), 2.71 (t, *J* = 8.2 Hz, 2H), 2.40 (t, *J* = 6.3 Hz, 2H), 2.05 – 2.02 (m, 2H), 1.84 – 1.66 (m, 10H), 0.67 (s, 3H), 0.54 (s, 3H). ESI-MS, positive mode: m/z = 880.4 $[M]^+$. HRMS (ESI) calcd for $C_{56}H_{59}N_3O_3PSi$ $[M]^+$ 880.4058, found 880.4039.

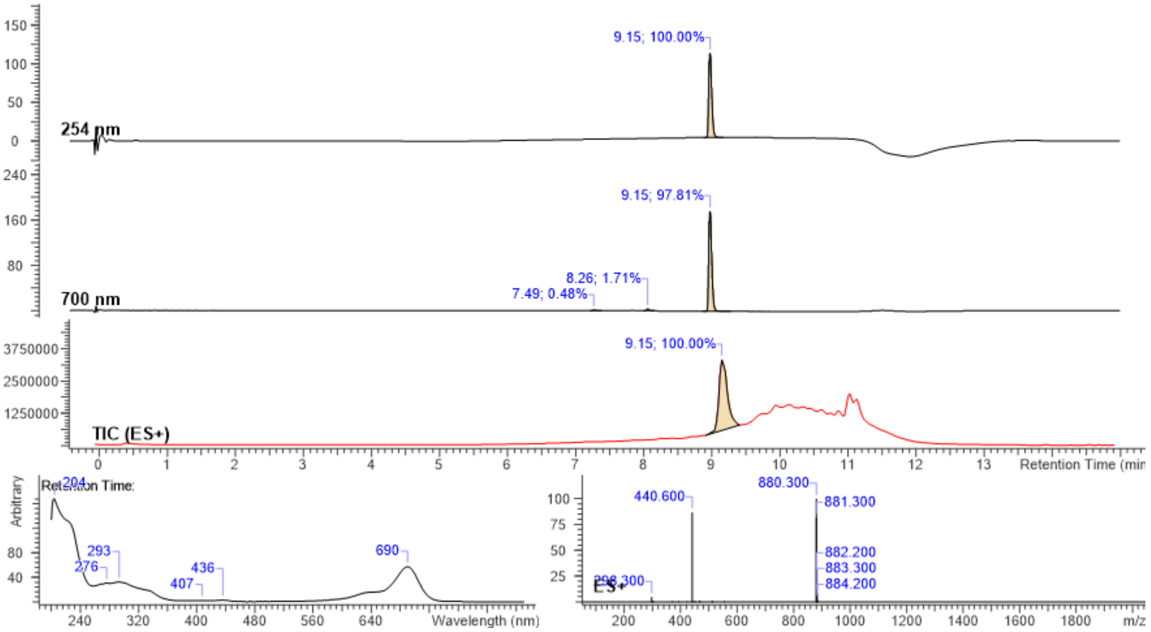

Supplementary Figure 87. LC/MS analysis results of compound **85**.

4-700SiR-TPP (86):

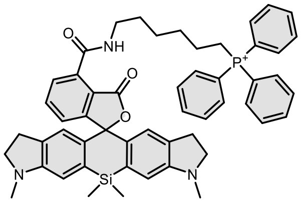

Compound was purified by preparative HPLC (solvent A: H_2O + 10 mM NH_4COOH pH = 3.6, solvent B: MeCN; temperature 25°C, gradient A:B - 3 min 40:60 isocratic, 4-20 min 40:60 to 0:100 gradient, 20-25 min 0:100 isocratic, 40 mL/min flow). Fractions containing the product were collected, evaporated and lyophilized from acetonitrile water mixture. The obtained solid was dissolved in 700 µL of d_6-DMSO. The samples from DMSO solution were diluted x100 in PBS +0.1% SDS and concentration was determined spectroscopically with Nanodrop. Determined stock concentration was 2.3 mM which constitutes to 32% yield (1.3 mg).

^{1}H NMR (400 MHz, CD_3OD) δ 7.88 (dd, *J* = 7.6, 0.9 Hz, 1H), 7.84 – 7.71 (m, 11H), 7.65 – 7.61 (m, 5H), 7.25 (dd, *J* = 7.8, 1.0 Hz, 1H), 6.79 (s, 2H), 6.57 (d, *J* = 0.5 Hz, 2H), 3.51 – 3.42 (m, 4H), 3.24 (h, *J* = 8.4 Hz, 4H), 2.82 (s, 6H), 2.72 (td, *J* = 8.2, 1.1 Hz, 4H), 1.67 (s, 8H), 0.60 (s, 3H), 0.47 (s, 3H). ESI-MS, positive mode: m/z = 840.2 $[M]^+$. HRMS (ESI) calcd for $C_{53}H_{55}N_3O_3PSi$ $[M]^+$ 840.3745, found 840.3745.

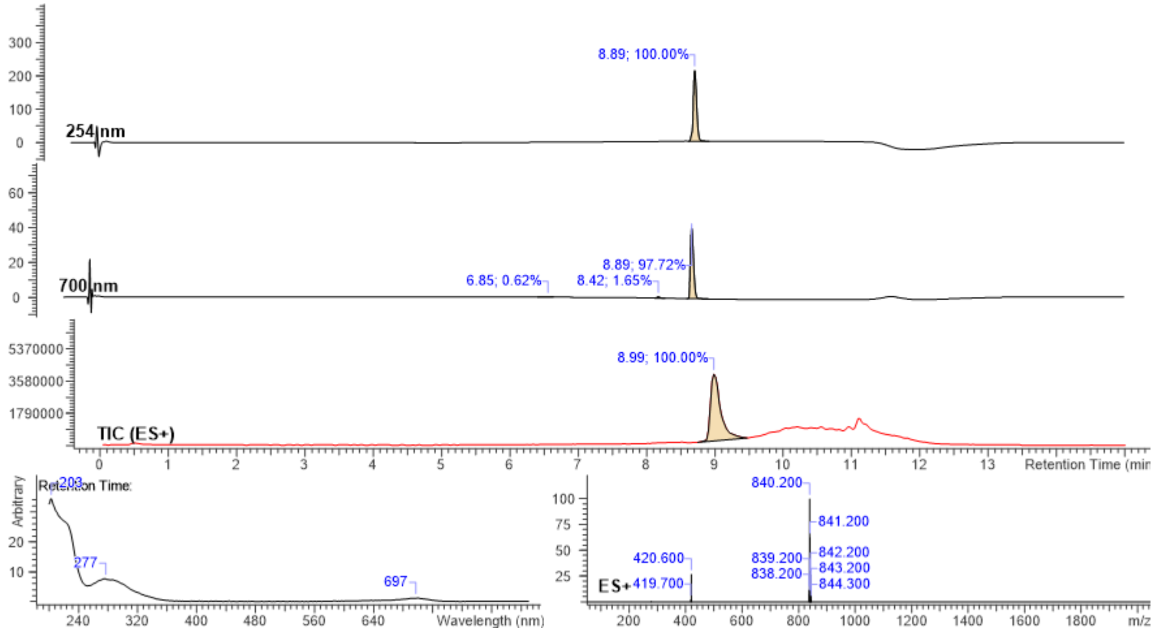

Supplementary Figure 88. LC/MS analysis results of compound **86**.

General procedure for the synthesis of compounds **87**-**92**:

Into a solution of corresponding rhodamine dye (5 µmol, 1 eq; **10**, **11, 15, 22, 23, 26**) and DIPEA (5 µL) in DMSO (100 µL) a solution of HATU (6.5 µmol, 1.3 eq) in DMSO (100 µL) was added and the mixture was mixed for 1 min. Then a solution of **Hoechst-C$_4$-NH$_2$**[28] (7.5 µmol ,1.5 eq) in DMSO (100 µL +5 µL DIPEA) was added at once. Reactions were usually over after 30 min and their course was monitored by LC/MS analysis. Once reaction was finished it was quenched with 20 µL of formic acid and diluted with water and acetonitrile to 2 mL volume and was further purified by the means of preparative HPLC (preparative column: Agilent 5 Prep-C18, 5 µm, 100 x 50 mm).

4-CFL-Hoechst (**87**):

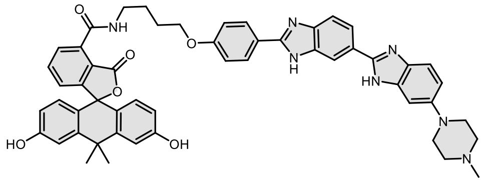

Compound was purified by preparative HPLC (solvent A: H_2O + 0.2% HCOOH, solvent B: MeCN; temperature 25 °C, gradient A:B - 3 min 80:20 isocratic, 4-20 min 80:20 to 0:100 gradient, 20-25 min 0:100 isocratic, 40 mL/min flow). Fractions containing the product were collected, evaporated and lyophilized from acetonitrile water mixture to give product in 48% yield (2.1 mg) as a colorless solid.

^{1}H NMR (400 MHz, *d*$_6$-DMSO) δ 12.93 (br s, 1H), 12.55 (br s, 1H), 9.70 (br s, 2H), 9.06 (t, *J* = 5.7 Hz, 1H), 8.43 – 8.17 (br m, 2H), 8.17 – 8.06 (m, 3H), 7.99 (br s, 1H), 7.77 (dd, *J* = 7.5, 1.1 Hz, 1H), 7.71 (t, *J* = 7.5 Hz, 1H), 7.47 (br s, 1H), 7.16 (d, *J* = 9.0 Hz, 2H), 7.08 (dd, *J* = 7.6, 1.2 Hz, 2H), 6.94 (d, *J* = 8.6 Hz, 2H), 6.69 – 6.46 (m, 4H), 4.16 (t, *J* = 6.4 Hz, 2H), 3.46 (q, *J* = 6.7 Hz, 2H), 3.17-3.09 (m, 4H), 2.61-2.51 (m, 4H), 2.28 (s, 3H), 1.94 (p, *J* = 7.1 Hz, 2H), 1.80 (p, *J* = 7.2 Hz, 2H), 1.73 (s, 3H), 1.64 (s, 3H). ESI-MS, positive mode: m/z = 880.4 [M+H]$^+$. HRMS (ESI) calcd for $C_{53}H_{50}N_7O_6$ [M+H]$^+$ 880.3817, found 880.3807.

LC/MS:

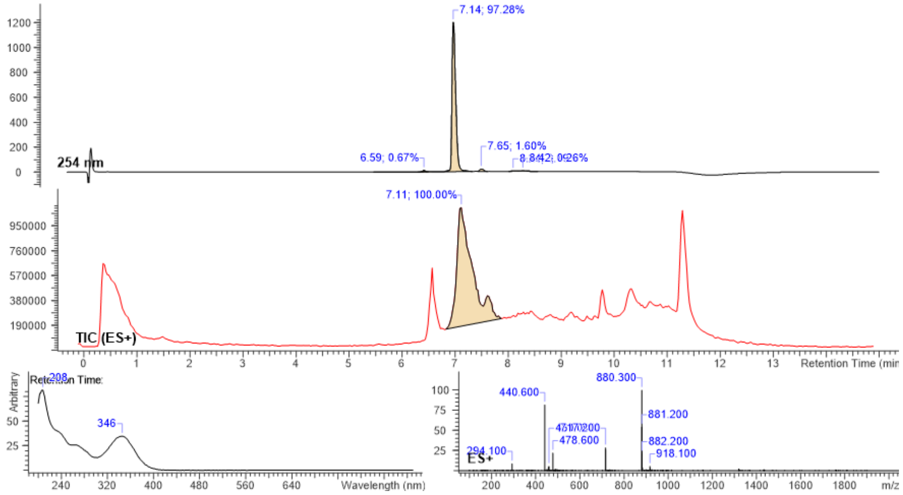

Supplementary Figure 89. LC/MS analysis results of compound **87**.

4-505R-Hoechst (**88**):

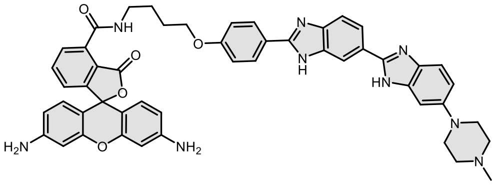

Compound was purified by preparative HPLC (solvent A: H_2O + 10 mM NH_4COOH pH = 3.6, solvent B: MeCN; temperature 25°C, gradient A:B - 3 min 80:20 isocratic, 4-20 min 80:20 to 0:100 gradient, 20-25 min 0:100 isocratic, 40 mL/min flow). Fractions containing the product were collected, evaporated and lyophilized from acetonitrile water mixture. The obtained solid was dissolved in 700 µL of d_6-DMSO. The samples from DMSO solution were diluted x100 in PBS +0.1% SDS and concentration was determined spectroscopically with Nanodrop. Determined stock concentration was 3.8 mM which constitutes to 53% yield (2.2 mg).

^{1}H NMR (400 MHz, d_6-DMSO) δ 9.10 (t, J = 5.6 Hz, 1H), 8.15 (d, J = 8.8 Hz, 2H), 7.99 (dd, J = 8.5, 1.6 Hz, 1H), 7.82 (d, J = 1.2 Hz, 1H), 7.77 (t, J = 7.5 Hz, 1H), 7.66 (d, J = 8.4 Hz, 1H), 7.44 (d, J = 8.7 Hz, 1H), 7.25 (dd, J = 7.5, 1.2 Hz, 1H), 7.15 (d, J = 8.9 Hz, 2H), 7.00 (s, 2H), 6.95 – 6.89 (m, 1H), 6.45 (d, J = 8.6 Hz, 2H), 6.38 (d, J = 2.2 Hz, 2H), 6.30 (dd, J = 8.5, 2.2 Hz, 2H), 5.59 (s, 4H), 4.15 (t, J = 6.3 Hz, 2H), 3.44 (q, J = 6.2 Hz, 2H), 3.13 (t, J = 4.9 Hz, 4H), 2.57 – 2.51 (m, 4H), 2.25 (s, 3H), 1.92 (p, J = 7.5 Hz, 2H), 1.78 (p, J = 7.3 Hz, 2H). ESI-MS, positive mode: m/z = 852.4 $[M+H]^+$. HRMS (ESI) calcd for $C_{50}H_{46}N_9O_5$ $[M+H]^+$ 852.3616, found 852.3612.

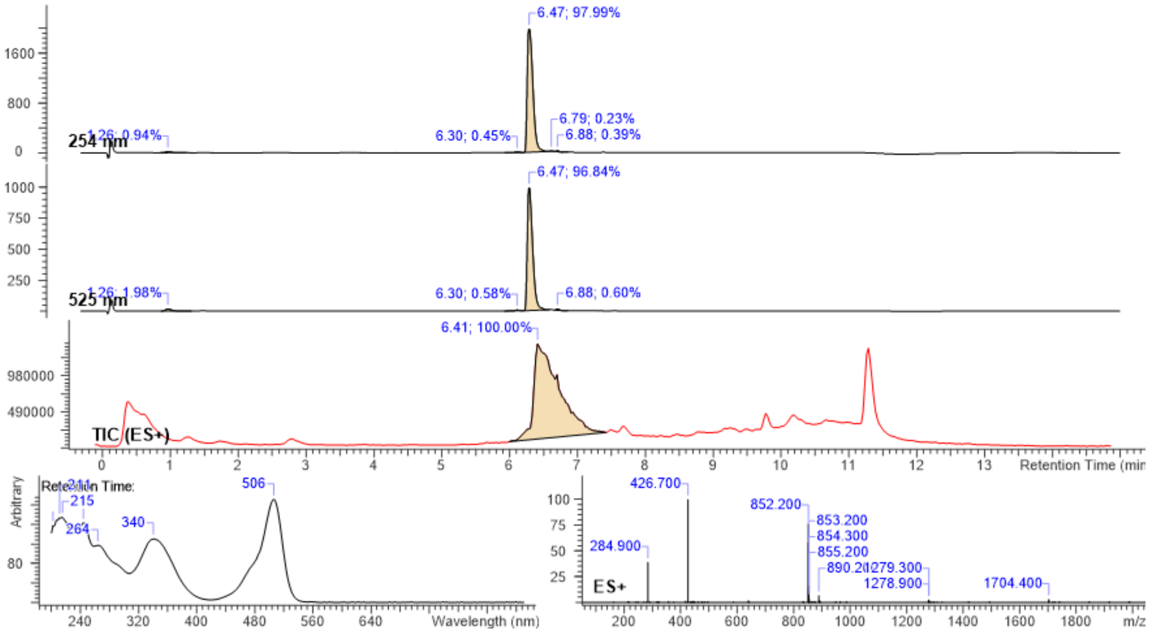

Supplementary Figure 90. LC/MS analysis results of compound **88**.

4-525R-Hoechst (**89**):

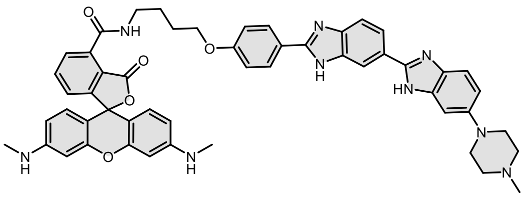

Compound was purified by preparative HPLC (solvent A: H_2O + 10 mM NH_4COOH pH = 3.6, solvent B: MeCN; temperature 25°C, gradient A:B - 3 min 70:30 isocratic, 4-20 min 70:30 to 0:100 gradient, 20-25 min 0:100 isocratic, 40 mL/min flow). Fractions containing the product were collected, evaporated and lyophilized from acetonitrile water mixture. The obtained solid was dissolved in 700 µL of d_6-DMSO. The samples from DMSO solution were diluted x100 in PBS +0.1% SDS and concentration was determined spectroscopically with Nanodrop. Determined stock concentration was 3.1 mM which constitutes to 44% yield (1.9 mg).

^{1}H NMR (400 MHz, d_6-DMSO + CF_3COOD) δ 9.11 (t, J = 5.6 Hz, 1H), 8.27 (s, 1H), 8.15 (d, J = 8.8 Hz, 2H), 7.99 (d, J = 8.5 Hz, 1H), 7.83 (dd, J = 7.5, 1.1 Hz, 1H), 7.77 (t, J = 7.6 Hz, 1H), 7.66 (d, J = 8.4 Hz, 1H), 7.44 (d, J = 8.0 Hz, 1H), 7.24 (dd, J = 7.6, 1.1 Hz, 1H), 7.15 (d, J = 9.0 Hz, 2H), 6.99 (s, 1H), 6.93 (dd, J = 8.8, 2.2 Hz, 1H), 6.50 (d, J = 8.5 Hz, 2H), 6.36 – 6.29 (m, 4H), 6.18 (q, J = 4.9 Hz, 2H), 4.15 (t, J = 6.3 Hz, 2H), 3.45 (p, J = 6.8 Hz, 2H), 3.13 (t, J = 5.0 Hz, 4H), 2.69 (d, J = 4.7 Hz, 6H), 2.54 – 2.51 (m, 4H), 2.25 (s, 3H), 1.96 – 1.89 (m, 2H), 1.83 – 1.76 (m, 2H). ESI-MS, positive mode: m/z = 880.4 $[M+H]^+$. HRMS (ESI) calcd for $C_{52}H_{50}N_9O_5$ $[M+H]^+$ 880.3929, found 880.3935.

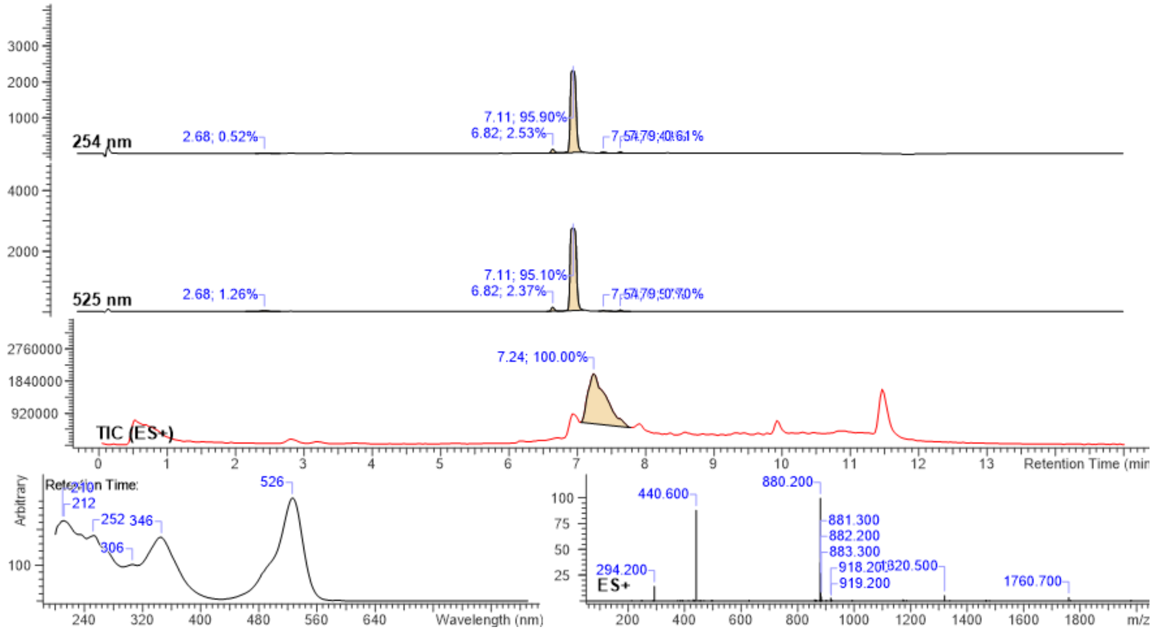

Supplementary Figure 91. LC/MS analysis results of compound **89**.

4-625CP-Hoechst (**90**):

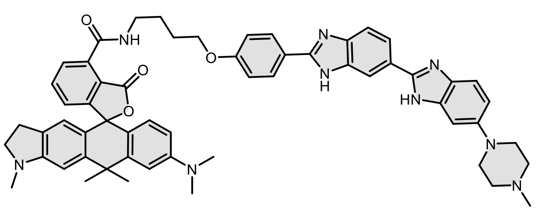

Compound was purified by preparative HPLC (solvent A: H_2O + 10 mM NH_4COOH pH = 3.6, solvent B: MeCN; temperature 25°C, gradient A:B - 3 min 70:30 isocratic, 4-20 min 70:30 to 0:100 gradient, 20-25 min 0:100 isocratic, 40 mL/min flow). Fractions containing the product were collected, evaporated and lyophilized from acetonitrile water mixture. The obtained solid was dissolved in 700 µL of d_6-DMSO. The samples from DMSO solution were diluted x100 in PBS +0.1% SDS and concentration was determined spectroscopically with Nanodrop. Determined stock concentration was 3.5 mM which constitutes to 49% yield (2.3 mg).

^{1}H NMR (400 MHz, d_6-DMSO + CF_3COOD) δ 9.17 (t, *J* = 5.6 Hz, 1H), 8.28 (s, 1H), 8.15 (d, *J* = 8.8 Hz, 2H), 7.99 (d, *J* = 8.5 Hz, 1H), 7.77 (dd, *J* = 7.5, 1.0 Hz, 1H), 7.72 – 7.63 (m, 2H), 7.44 (s, 1H), 7.16 (d, *J* = 9.0 Hz, 2H), 7.04 (dd, *J* = 7.7, 1.0 Hz, 1H), 6.99 – 6.87 (m, 3H), 6.76 (s, 1H), 6.59 – 6.53 (m, 2H), 6.39 (s, 1H), 4.16 (t, *J* = 6.4 Hz, 2H), 3.46 (q, *J* = 6.6 Hz, 2H), 3.25 – 3.22 (m, 2H), 3.13 (t, *J* = 4.7 Hz, 4H), 2.93 (s, 6H), 2.79 (s, 3H), 2.77 – 2.68 (m, 2H), 2.56 – 2.51 (m, 4H), 2.25 (s, 3H), 1.98 – 1.92 (m, 2H), 1.85 – 1.77 (m, 5H), 1.71 (s, 3H). ESI-MS, positive mode: m/z = 946.5 $[M+H]^+$. HRMS (ESI) calcd for $C_{58}H_{60}N_9O_4$ $[M+H]^+$ 946.4763, found 946.4743.

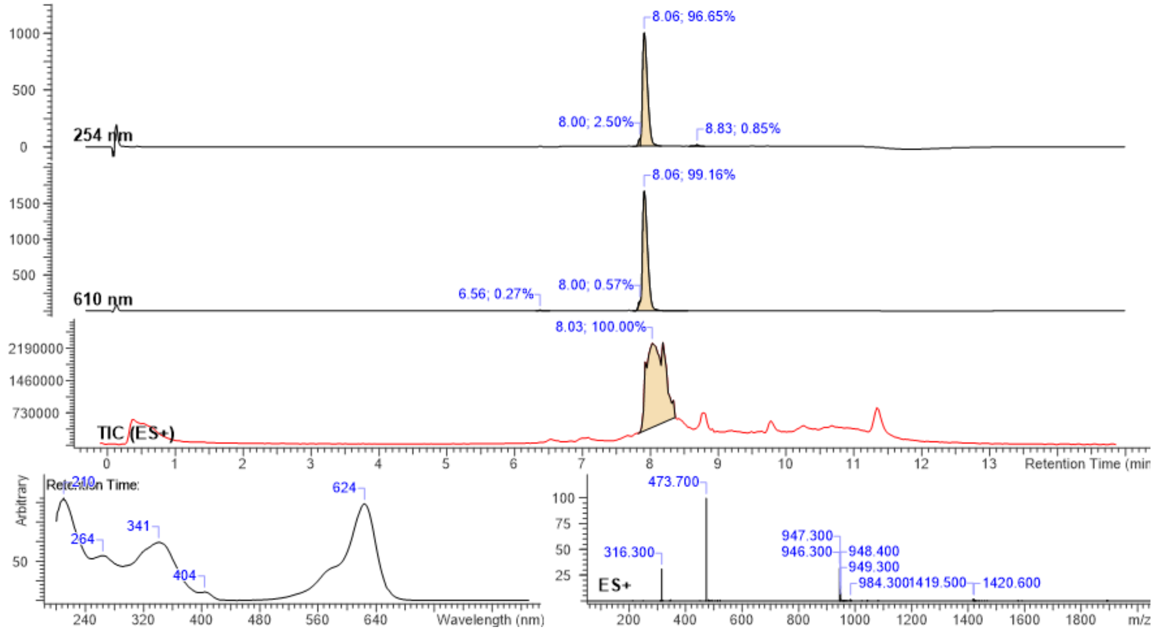

Supplementary Figure 92. LC/MS analysis results of compound **90**.

4-630CP-Hoechst (91):

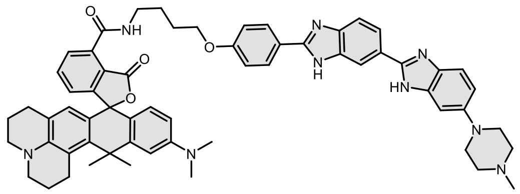

Compound was purified by preparative HPLC (solvent A: H_2O + 10 mM NH_4COOH pH = 3.6, solvent B: MeCN; temperature 25°C, gradient A:B - 3 min 70:30 isocratic, 4-20 min 70:30 to 0:100 gradient, 20-25 min 0:100 isocratic, 40 mL/min flow). Fractions containing the product were collected, evaporated and lyophilized from acetonitrile water mixture. The obtained solid was dissolved in 700 µL of d_6-DMSO. The samples from DMSO solution were diluted x100 in PBS +0.1% SDS and concentration was determined spectroscopically with Nanodrop. Determined stock concentration was 2.3 mM which constitutes to 32% yield (1.6 mg).

^{1}H NMR (400 MHz, d_6-DMSO) δ 12.93 (s, 1H), 12.57 (s, 1H), 9.20 (t, *J* = 5.6 Hz, 1H), 8.34 (s, 1H), 8.22 (s, 1H), 8.15 (d, *J* = 8.6 Hz, 3H), 8.03 – 7.96 (m, 1H), 7.76 (dd, *J* = 7.5, 1.0 Hz, 1H), 7.71 (d, *J* = 8.6 Hz, 1H), 7.67 (t, *J* = 7.6 Hz, 1H), 7.60 (d, *J* = 8.2 Hz, 1H), 7.16 (d, *J* = 8.8 Hz, 2H), 7.00 (dd, *J* = 7.7, 1.0 Hz, 1H), 6.94 (d, *J* = 7.2 Hz, 1H), 6.79 (d, *J* = 2.6 Hz, 1H), 6.57 (dd, *J* = 9.0, 2.5 Hz, 1H), 6.50 (d, *J* = 8.9 Hz, 1H), 6.16 (s, 1H), 4.17 (t, *J* = 6.4 Hz, 2H), 3.46 (q, *J* = 6.7 Hz, 2H), 3.19 – 3.12 (m, 8H), 2.97 – 2.89 (m, 8H), 2.62 – 2.56 (m, 4H), 2.47 – 2.36 (m, 2H), 2.31 (s, 3H), 1.98 – 1.90 (m, 4H), 1.89 (s, 3H), 1.83 (s, 3H), 1.82 – 1.71 (m, 4H). ESI-MS, positive mode: m/z = 986.5 $[M+H]^+$. HRMS (ESI) calcd for $C_{61}H_{64}N_9O_4Si$ $[M+H]^+$ 986.5076, found 986.5056.

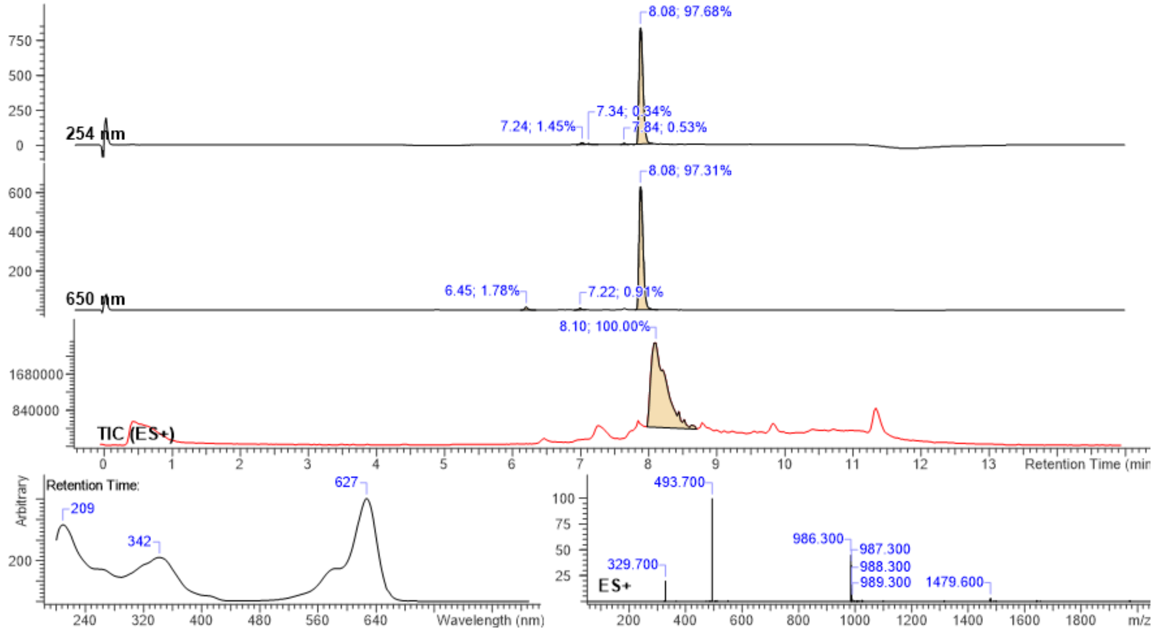

Supplementary Figure 93. LC/MS analysis results of compound **91**.

4-685SiR-Hoechst (**92**):

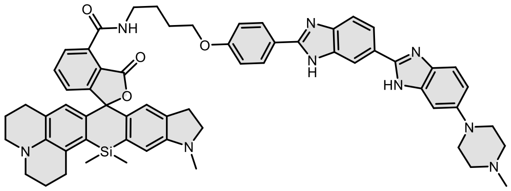

Compound was purified by preparative HPLC (solvent A: H_2O + 10 mM NH_4COOH pH = 3.6, solvent B: MeCN; temperature 25°C, gradient A:B - 3 min 70:30 isocratic, 4-20 min 70:30 to 0:100 gradient, 20-25 min 0:100 isocratic, 40 mL/min flow). Fractions containing the product were collected, evaporated and lyophilized from acetonitrile water mixture. The obtained solid was dissolved in 700 µL of d_6-DMSO. The samples from DMSO solution were diluted x100 in PBS +0.1% SDS and concentration was determined spectroscopically with Nanodrop. Determined stock concentration was 4.1 mM which constitutes to 58% yield (2.9 mg).

^{1}H NMR (400 MHz, *d*$_6$-DMSO) δ 12.95 (s, 1H), 12.56 (s, 1H), 9.11 (t, *J* = 5.7 Hz, 1H), 8.29 (s, 2H), 8.15 (d, *J* = 8.8 Hz, 2H), 7.99 (d, *J* = 8.4 Hz, 1H), 7.74 – 7.64 (m, 3H), 7.45 (s, 2H), 7.16 (d, *J* = 9.0 Hz, 2H), 7.09 (dd, *J* = 6.9, 1.8 Hz, 1H), 6.93 (d, *J* = 8.4 Hz, 1H), 6.75 (s, 1H), 6.57 (s, 1H), 6.34 (s, 1H), 4.16 (t, *J* = 6.3 Hz, 2H), 3.44 (q, *J* = 6.2 Hz, 2H), 3.22 – 3.10 (m, 10H), 2.90 (t, *J* = 6.3 Hz, 2H), 2.81 – 2.70 (m, 5H), 2.56 – 2.51 (m, 4H), 2.48 – 2.39 (m, 2H), 2.25 (s, 3H), 1.98 – 1.90 (m, 4H), 1.84 – 1.72 (m, 4H), 0.64 (s, 3H), 0.54 (s, 3H). ESI-MS, positive mode: m/z = 1014.5 $[M+H]^+$. HRMS (ESI) calcd for $C_{61}H_{64}N_9O_4Si$ $[M+H]^+$ 1014.4845, found 1014.4810.

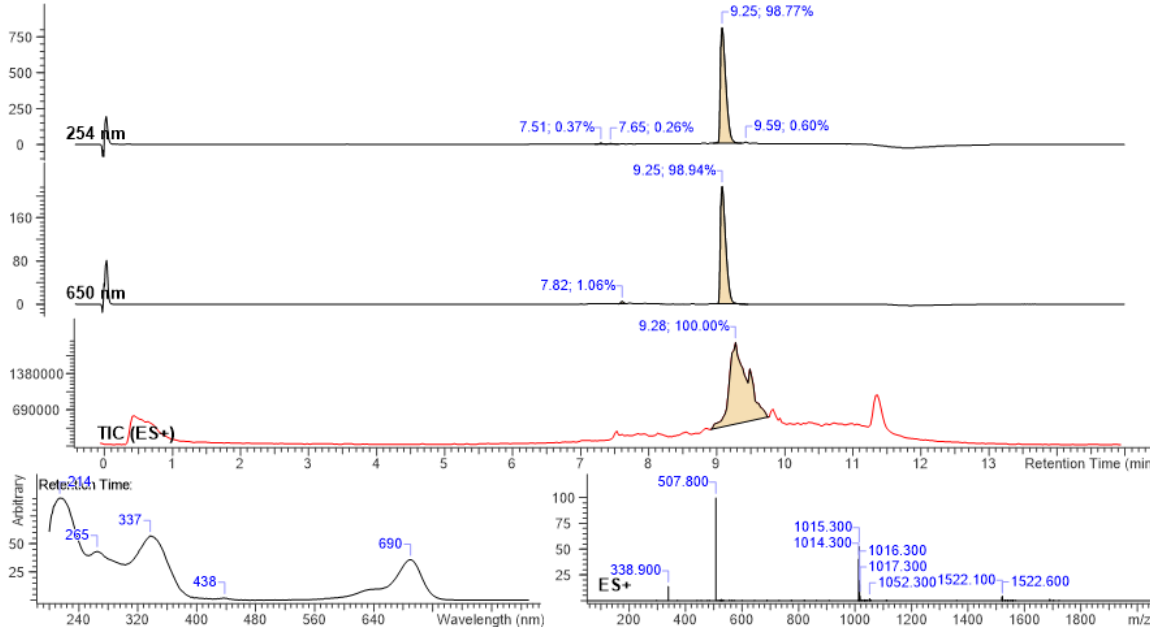

Supplementary Figure 94. LC/MS analysis results of compound **92**.

PepA-C6-NHBoc:

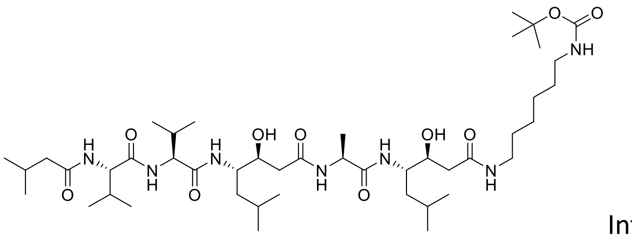

Into a solution of Pepstatin A (50 mg, 73 µmol, 1 eq;) and DIPEA (50 µL) in DMSO (1000 µL) a solution of TSTU (27 mg ,88 µmol, 1.2 eq) in DMSO (100 µL) was added and the mixture was mixed for 1-2 hours. Then a solution of N-(tert-Butoxycarbonyl)-1,6-diaminohexane (24 mg,110 µmol ,1.5 eq) in DMSO (100 µL +5 µL DIPEA) was added at once. Reaction was stirred for 1-2 hours the course of reaction was monitored by LC/MS analysis. Once reaction was finished it was quenched with 50 µL of formic acid and was further purified by the means of preparative HPLC (preparative column: Agilent 5 Prep-C18, 5 µm, 100 x 50 mm), collected fractions were collected and evaporated. The residue was dissolved in MeCN/H_2O mixture and lyophilized to give 35 mg (54% yield) of colorless solid.

^{1}H NMR (400 MHz, d_6-DMSO) δ 7.92 (d, *J* = 7.3 Hz, 1H), 7.82 (d, *J* = 8.8 Hz, 1H), 7.78 (d, *J* = 8.8 Hz, 1H), 7.67 (t, *J* = 5.6 Hz, 1H), 7.46 (d, *J* = 8.8 Hz, 1H), 7.32 (d, *J* = 9.1 Hz, 1H), 6.75 (t, *J* = 5.0 Hz, 1H), 4.84 (t, *J* = 5.7 Hz, 2H), 4.27 – 4.10 (m, 3H), 3.90 – 3.71 (m, 4H), 3.05 – 2.96 (m, 2H), 2.91 – 2.85 (m, 2H), 2.20 – 1.87 (m, 9H), 1.60 – 1.47 (m, 3H), 1.46 – 0.96 (m, 30H), 0.95 – 0.66 (m, 24H). ^{13}C NMR (101 MHz, d_6-DMSO) δ 172.2, 171.6, 171.1, 170.8, 170.7, 170.6, 155.6, 77.3, 69.2, 69.0, 58.0, 57.8, 50.7, 50.4, 48.4, 44.4, 38.6, 38.4, 30.3, 30.1, 29.4, 29.1, 28.3, 26.1, 26.0, 25.7, 25.2, 24.2, 23.5, 23.3, 22.3, 22.3, 21.9, 21.6, 19.3, 19.3, 18.4, 18.3, 18.2. ESI-MS, positive mode: m/z = 906.6 $[M+Na]^+$. HRMS (ESI) calcd for $C_{45}H_{85}N_7O_{10}Na$ $[M+Na]^+$ 906.6250, found 906.6252.

<u>General procedure for the synthesis of compounds **93**-**99**:</u>

PepA-C6-NH-Boc (35 mg, 39 µmol) was dissolved in formic acid (1 mL) and was stirred for 3 hours at room temperature. The excess of formic acid was evaporated on rotary evaporator MeCN was added couple of times and evaporated to ensure complete removal of formic acid. Finally, residue was dissolved in MeCN/H_2O mixture and lyophilized to afford **PePA-C6-NH$_2$** quantitatively as a colorless solid. The obtained compound was further without any additional purifications.

Into a solution of corresponding rhodamine dye (5 µmol, 1 eq; **2**, **5**, **8**, **11**, **12**, **15**, **23**) and DIPEA (5 µL) in DMSO (100 µL) a solution of HATU (6.5 µmol, 1.3 eq) in DMSO (100 µL) was added and the mixture was mixed for 1 min. Then a solution of **PepA-C6-NH$_2$** (7.5 µmol ,1.5 eq) in DMSO (100 µL +5 µL DIPEA) was added at once. Reactions were usually over after 30 min and their course was monitored by LC/MS

analysis. Once reaction was finished it was quenched with 20 µL of formic acid and diluted with water and acetonitrile to 2 mL volume and was further purified by the means of preparative HPLC (preparative column: Agilent 5 Prep-C18, 5 µm, 100 x 50 mm).

4-525R-PepA (93):

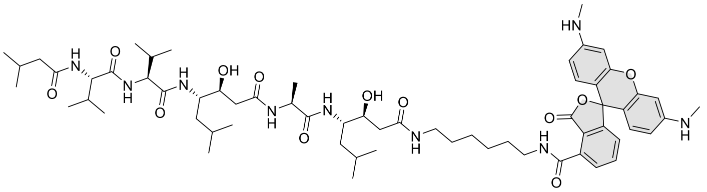

Compound was purified by preparative HPLC (solvent A: H_2O + 10 mM NH_4COOH pH = 3.6, solvent B: MeCN; temperature 25°C, gradient A:B - 3 min 70:30 isocratic, 4-20 min 70:30 to 0:100 gradient, 20-25 min 0:100 isocratic, 40 mL/min flow). Fractions containing the product were collected, evaporated and lyophilized from acetonitrile water mixture. The obtained solid was dissolved in 700 µL of d_6-DMSO. The samples from DMSO solution were diluted x100 in PBS +0.1% SDS and concentration was determined spectroscopically with Nanodrop. Determined stock concentration was 2.2 mM which constitutes to 31% yield (1.8 mg).

^{1}H NMR (400 MHz, d_6-DMSO) δ 9.05 (t, J = 5.6 Hz, 1H), 7.96 (s, 1H), 7.90 (d, J = 7.4 Hz, 1H), 7.82 – 7.74 (m, 3H), 7.70 – 7.65 (m, 1H), 7.45 (d, J = 8.9 Hz, 1H), 7.30 (s, 1H), 7.22 – 7.18 (m, 1H), 6.53 – 6.43 (m, 2H), 6.44 – 6.21 (m, 4H), 6.16 (d, J = 5.1 Hz, 2H), 4.85 – 4.79 (m, 2H), 4.24 – 4.10 (m, 3H), 3.80 (s, 4H), 3.06 – 2.93 (m, 4H), 2.68 (d, J = 4.8 Hz, 6H), 2.11 – 1.89 (m, 9H), 1.68 – 1.07 (m, 23H), 0.84 – 0.76 (m, 24H). ESI-MS, positive mode: m/z = 1168.7 [M+H]$^+$. HRMS (ESI) calcd for $C_{63}H_{94}N_9O_{12}$ [M+H]$^+$ 1168.7016, found 1168.6960.

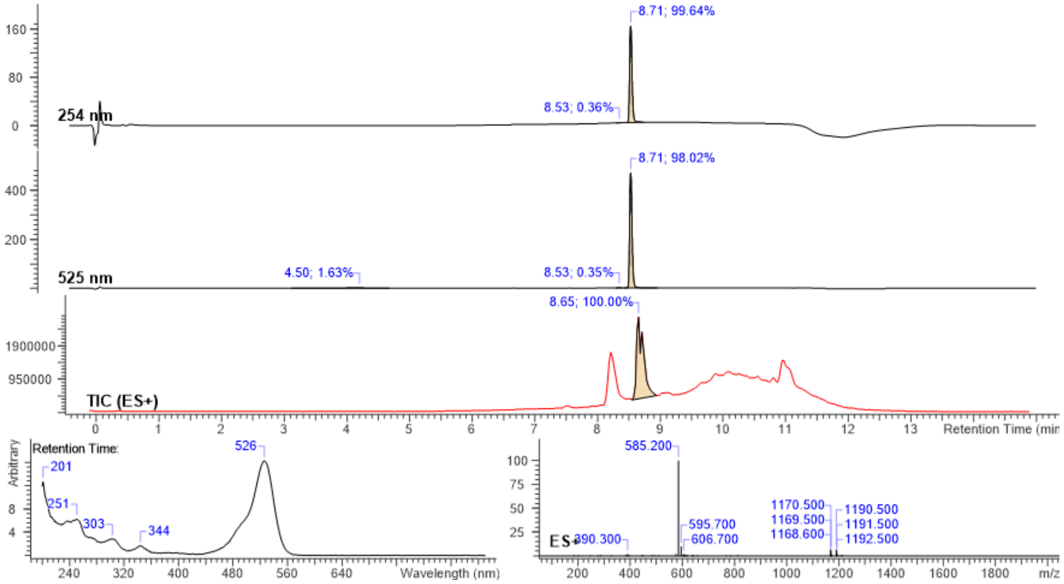

Supplementary Figure 95. LC/MS analysis results of compound **93**.

4-580R-PepA (**94**):

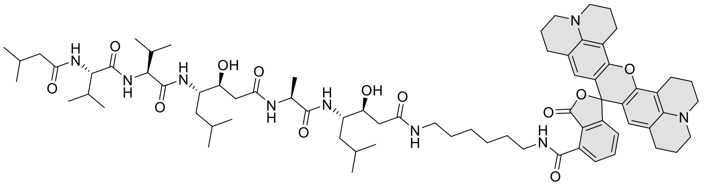

Compound was purified by preparative HPLC (solvent A: H_2O + 10 mM NH_4COOH pH = 3.6, solvent B: MeCN; temperature 25°C, gradient A:B - 3 min 70:30 isocratic, 4-20 min 70:30 to 0:100 gradient, 20-25 min 0:100 isocratic, 40 mL/min flow). Fractions containing the product were collected, evaporated and lyophilized from acetonitrile water mixture. The obtained solid was dissolved in 700 µL of d_6-DMSO. The samples from DMSO solution were diluted x100 in PBS +0.1% SDS and concentration was determined spectroscopically with Nanodrop. Determined stock concentration was 2.7 mM which constitutes to 38% yield (2.5 mg).

^{1}H NMR (400 MHz, d_6-DMSO) δ 9.19 (t, *J* = 5.6 Hz, 1H), 8.33 (s, 1H), 7.92 (d, *J* = 7.3 Hz, 1H), 7.87 – 7.71 (m, 4H), 7.47 (d, *J* = 8.8 Hz, 1H), 7.33 (d, *J* = 9.1 Hz, 1H), 7.25 (d, *J* = 7.6 Hz, 1H), 6.11 (s, 2H), 4.85 (dd, *J* = 10.0, 4.9 Hz, 2H), 4.26 – 4.10 (m, 3H), 3.87 – 3.72 (m, 4H), 3.17 (t, *J* = 5.5 Hz, 4H), 3.13 (t, *J* = 5.6 Hz, 4H), 3.03 (dt, *J* = 14.7, 7.0 Hz, 4H), 2.85 (t, *J* = 6.6 Hz, 4H), 2.49 – 2.37 (m, 4H), 2.20 – 1.70 (m, 17H), 1.68 – 1.06 (m, 23H), 0.92 – 0.57 (m, 24H). ESI-MS, positive mode: m/z = 1300.8 $[M+H]^+$. HRMS (ESI) calcd for $C_{73}H_{106}N_9O_{12}$ $[M+H]^+$ 1300.7955, found 1300.7953.

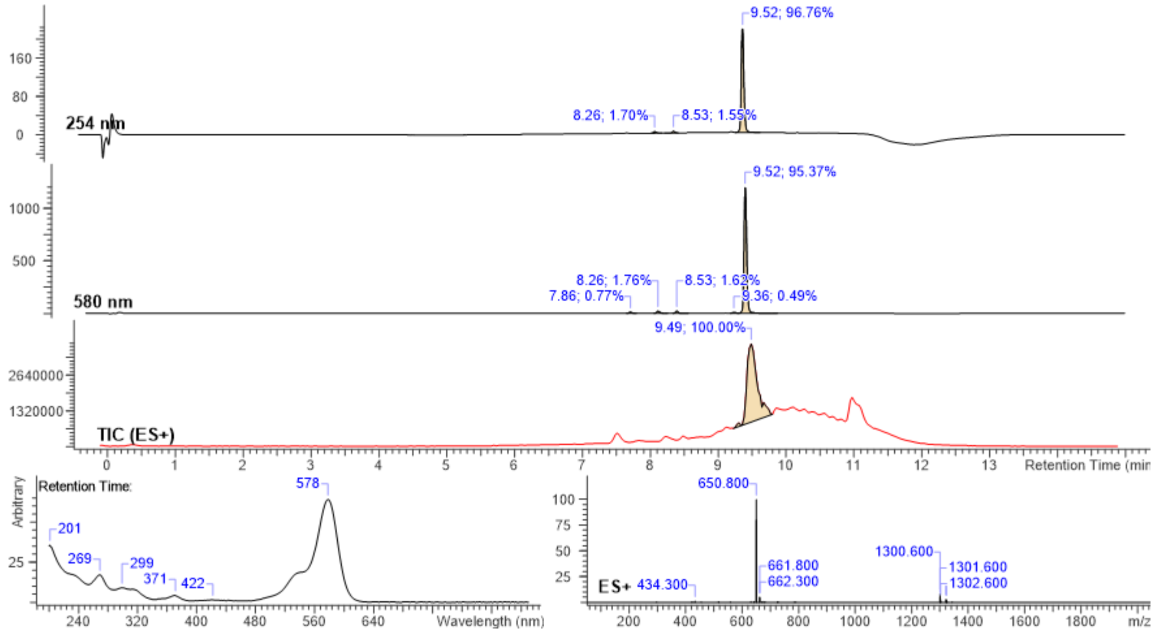

Supplementary Figure 96. LC/MS analysis results of compound **94**.

4-630CP-PepA (**95**):

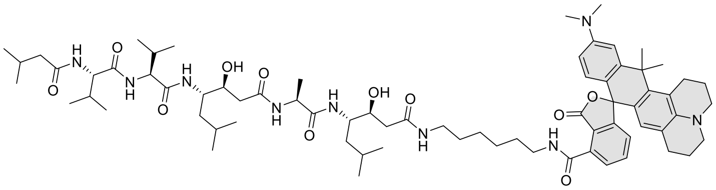

Compound was purified by preparative HPLC (solvent A: H_2O + 10 mM NH_4COOH pH = 3.6, solvent B: MeCN; temperature 25°C, gradient A:B - 3 min 70:30 isocratic, 4-20 min 70:30 to 0:100 gradient, 20-25 min 0:100 isocratic, 40 mL/min flow). Fractions containing the product were collected, evaporated and lyophilized from acetonitrile water mixture. The obtained solid was dissolved in 700 µL of d_6-DMSO. The samples from DMSO solution were diluted x100 in PBS +0.1% SDS and concentration was determined spectroscopically with Nanodrop. Determined stock concentration was 2.0 mM which constitutes to 28% yield (1.8 mg).

^{1}H NMR (400 MHz, d_6-DMSO) δ 9.16 (t, J = 5.6 Hz, 1H), 7.92 (d, J = 7.4 Hz, 1H), 7.83 – 7.63 (m, 5H), 7.46 (d, J = 8.8 Hz, 1H), 7.33 (d, J = 9.2 Hz, 1H), 7.00 (dd, J = 7.7, 0.9 Hz, 1H), 6.79 (d, J = 2.6 Hz, 1H), 6.57 (dd, J = 9.0, 2.5 Hz, 1H), 6.48 (d, J = 8.8 Hz, 1H), 6.14 (s, 1H), 4.85 (dd, J = 9.7, 5.0 Hz, 2H), 4.26 – 4.10 (m, 3H), 3.87 – 3.73 (m, 4H), 3.40 – 3.35 (m, 2H), 3.22 – 3.11 (m, 4H), 3.04 (p, J = 7.1 Hz, 2H), 3.01 – 2.78 (m, 8H), 2.48 – 2.36 (m, 2H), 2.31 – 1.82 (m, 16H), 1.82 (s, 3H), 1.77 – 1.67 (m, 2H), 1.65 – 1.17 (m, 21H), 0.89 – 0.70 (m, 24H). ESI-MS, positive mode: m/z = 1274.9 $[M+H]^+$. HRMS (ESI) calcd for $C_{72}H_{108}N_9O_{11}$ $[M+H]^+$ 1274.8163, found 1274.8162.

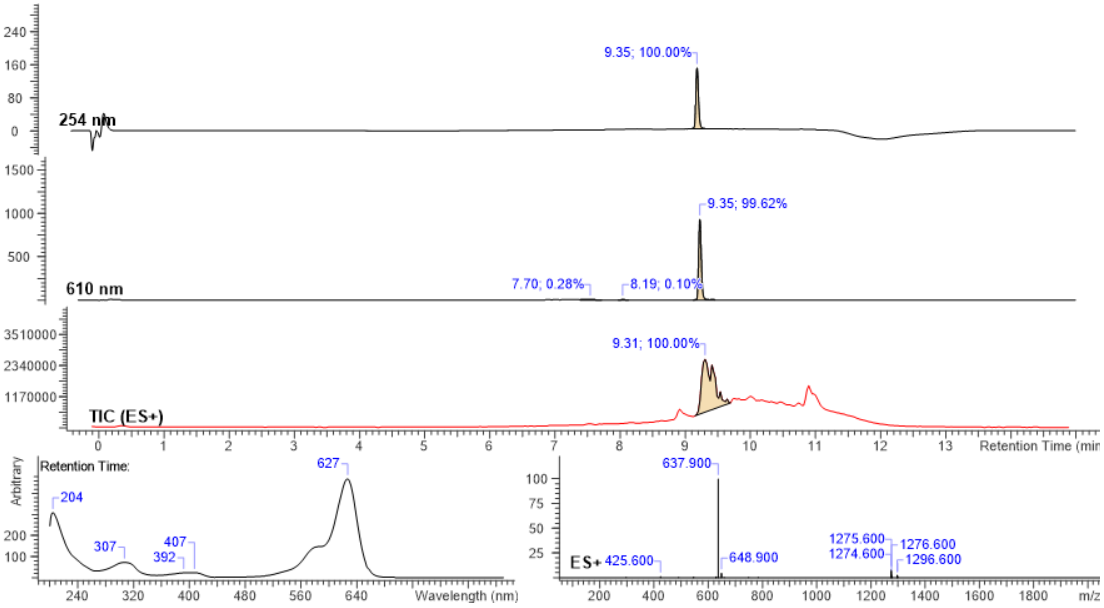

Supplementary Figure 97. LC/MS analysis results of compound **95**.

4-642CP-PepA (**96**):

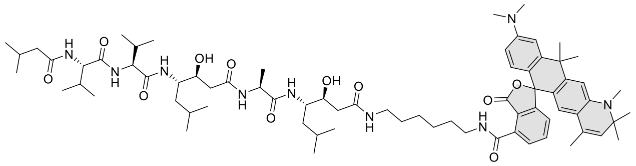

Compound was purified by preparative HPLC (solvent A: H_2O + 10 mM NH_4COOH pH = 3.6, solvent B: MeOH; temperature 25°C, gradient A:B - 3 min 70:30 isocratic, 4-20 min 70:30 to 0:100 gradient, 20-25 min 0:100 isocratic, 40 mL/min flow). Fractions containing the product were collected, evaporated and lyophilized from acetonitrile water mixture. The obtained solid was dissolved in 700 µL of d_6-DMSO. The samples from DMSO solution were diluted x100 in PBS +0.1% SDS and concentration was determined spectroscopically with Nanodrop. Determined stock concentration was 3.0 mM which constitutes to 42% yield (3.1 mg).

^{1}H NMR (400 MHz, d_6-DMSO) δ 9.17 (t, *J* = 5.6 Hz, 1H), 7.92 (d, *J* = 7.4 Hz, 1H), 7.84 – 7.67 (m, 5H), 7.47 (d, *J* = 8.9 Hz, 1H), 7.33 (d, *J* = 9.1 Hz, 1H), 7.10 (d, *J* = 7.6 Hz, 1H), 6.91 (s, 1H), 6.67 (s, 1H), 6.57 (s, 2H), 6.16 (s, 1H), 5.33 (d, *J* = 1.6 Hz, 1H), 4.85 (dd, *J* = 8.8, 5.1 Hz, 2H), 4.25 (t, *J* = 7.2 Hz, 1H), 4.18 (dd, *J* = 8.8, 7.3 Hz, 1H), 4.12 (dd, *J* = 8.9, 7.2 Hz, 1H), 3.85 – 3.72 (m, 4H), 3.38 – 3.35 (m, 2H), 3.04 (p, *J* = 6.7 Hz, 2H), 2.94 (s, 6H), 2.84 (s, 3H), 2.12 – 1.92 (m, 9H), 1.81 (s, 3H), 1.70 (s, 3H), 1.62 – 1.18 (m, 32H), 0.86 – 0.77 (m, 24H). ESI-MS, positive mode: m/z = 1288.9 $[M+H]^+$. HRMS (ESI) calcd for $C_{73}H_{110}N_9O_{11}$ $[M+H]^+$ 1288.8319, found 1288.8329.

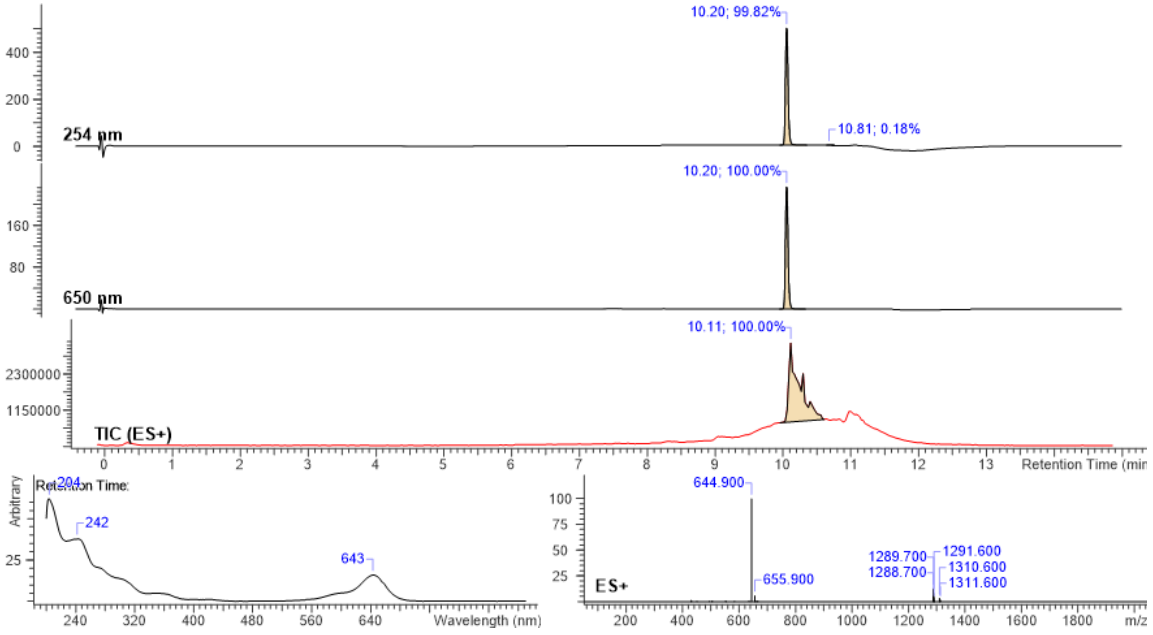

Supplementary Figure 98. LC/MS analysis results of compound **96**.

4-SiR-PepA (97):

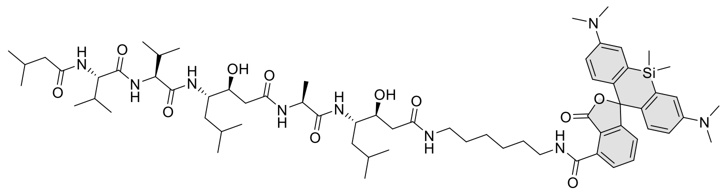

Compound was purified by preparative HPLC (solvent A: H_2O + 0.2% HCOOH, solvent B: MeCN; temperature 25 °C, gradient A:B - 3 min 40:60 isocratic, 4-20 min 40:60 to 0:100 gradient, 20-25 min 0:100 isocratic, 40 mL/min flow). Fractions containing the product were collected, evaporated and lyophilized from acetonitrile water mixture to give product in 45% yield (2.8 mg) as a light blue solid.

^{1}H NMR (400 MHz, d_6-DMSO) δ 9.01 (t, J = 5.5 Hz, 1H), 7.91 (d, J = 7.4 Hz, 1H), 7.86 – 7.73 (m, 4H), 7.70 (t, J = 5.6 Hz, 1H), 7.46 (d, J = 8.8 Hz, 1H), 7.33 (d, J = 9.1 Hz, 1H), 7.24 (t, J = 4.4 Hz, 1H), 7.00 (d, J = 2.8 Hz, 2H), 6.70 (d, J = 9.0 Hz, 2H), 6.64 (dd, J = 9.0, 2.8 Hz, 2H), 4.84 (dd, J = 8.4, 5.0 Hz, 2H), 4.25 (t, J = 7.2 Hz, 1H), 4.18 (dd, J = 8.8, 7.3 Hz, 1H), 4.12 (dd, J = 8.9, 7.2 Hz, 1H), 3.85 – 3.73 (m, 4H), 3.30 – 3.20 (m, 2H), 3.10 – 3.01 (m, 2H), 2.92 (s, 12H), 2.13 – 1.90 (m, 9H), 1.72 – 1.03 (m, 23H), 0.88 – 0.77 (m, 24H), 0.62 (s, 3H), 0.52 (s, 3H). ESI-MS, positive mode: m/z = 1260.7 $[M+Na]^+$. HRMS (ESI) calcd for $C_{67}H_{103}N_9O_{11}SiNa$ $[M+Na]^+$ 1260.7439, found 1260.7410.

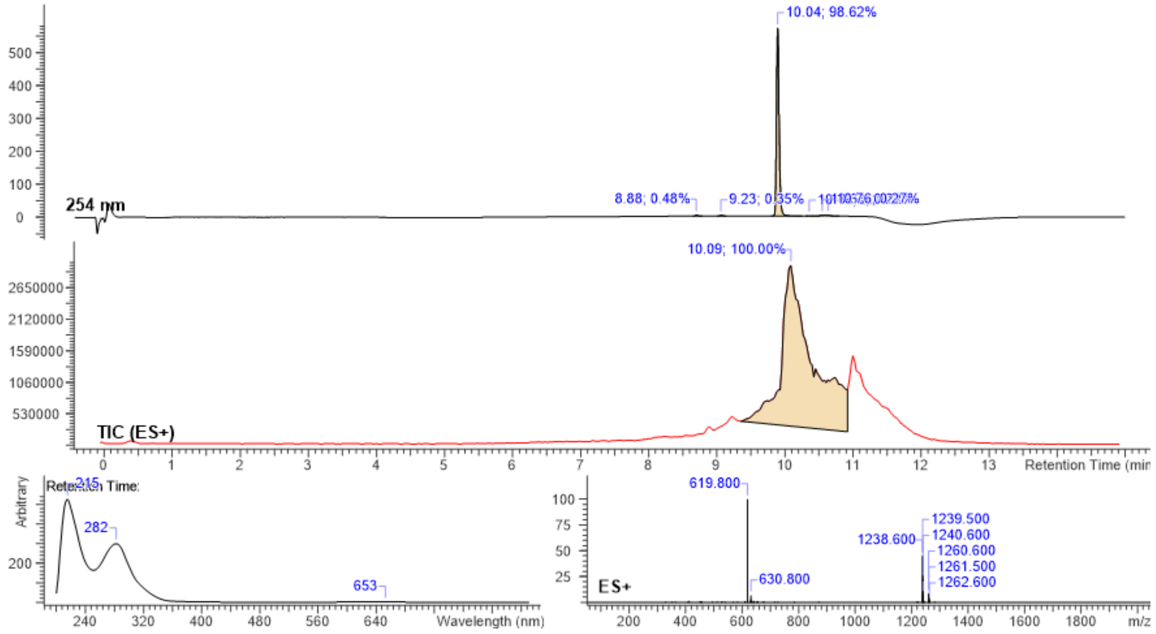

Supplementary Figure 99. LC/MS analysis results of compound **97**.

4-685SiR-PepA (98):

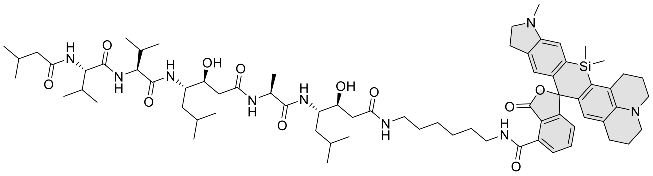

Compound was purified by preparative HPLC (solvent A: H_2O +0.2% HCOOH, solvent B: MeCN; temperature 25°C, gradient A:B - 3 min 70:30 isocratic, 4-20 min 70:30 to 0:100 gradient, 20-25 min 0:100 isocratic, 40 mL/min flow). Fractions containing the product were collected, evaporated and lyophilized from acetonitrile water mixture. The obtained solid was dissolved in 700 µL of d_6-DMSO. The samples from DMSO solution were diluted x100 in PBS +0.1% SDS and concentration was determined spectroscopically with Nanodrop. Determined stock concentration was 3.2 mM which constitutes to 45% yield (2.9 mg).

^{1}H NMR (400 MHz, d_6-DMSO) δ 9.07 (t, J = 5.6 Hz, 1H), 7.92 (d, J = 7.2 Hz, 1H), 7.82 (d, J = 8.8 Hz, 1H), 7.78 (d, J = 9.0 Hz, 1H), 7.75 – 7.63 (m, 3H), 7.47 (d, J = 8.8 Hz, 1H), 7.34 (d, J = 9.1 Hz, 1H), 7.08 (dd, J = 7.3, 1.5 Hz, 1H), 6.74 (s, 1H), 6.55 (s, 1H), 6.32 (s, 1H), 4.89 – 4.81 (m, 2H), 4.25 (t, J = 7.2 Hz, 1H), 4.18 (dd, J = 8.8, 7.3 Hz, 1H), 4.12 (dd, J = 9.0, 7.2 Hz, 1H), 3.87 – 3.73 (m, 4H), 3.45 – 3.38 (m, 2H), 3.24 – 3.09 (m, 6H), 3.08 – 3.00 (m, 2H), 2.90 (t, J = 6.1 Hz, 2H), 2.82 – 2.66 (m, 5H), 2.43 (dd, J = 16.1, 6.4 Hz, 2H), 2.35 – 1.87 (m, 13H), 1.80 – 1.72 (m, 2H), 1.59 – 1.18 (m, 21H), 1.04 – 0.69 (m, 24H), 0.64 (s, 3H), 0.53 (s, 3H). ESI-MS, positive mode: m/z = 1302.7 $[M+H]^+$. HRMS (ESI) calcd for $C_{72}H_{108}N_9O_{11}Si$ $[M+H]^+$ 1302.7932, found 1302.7932.

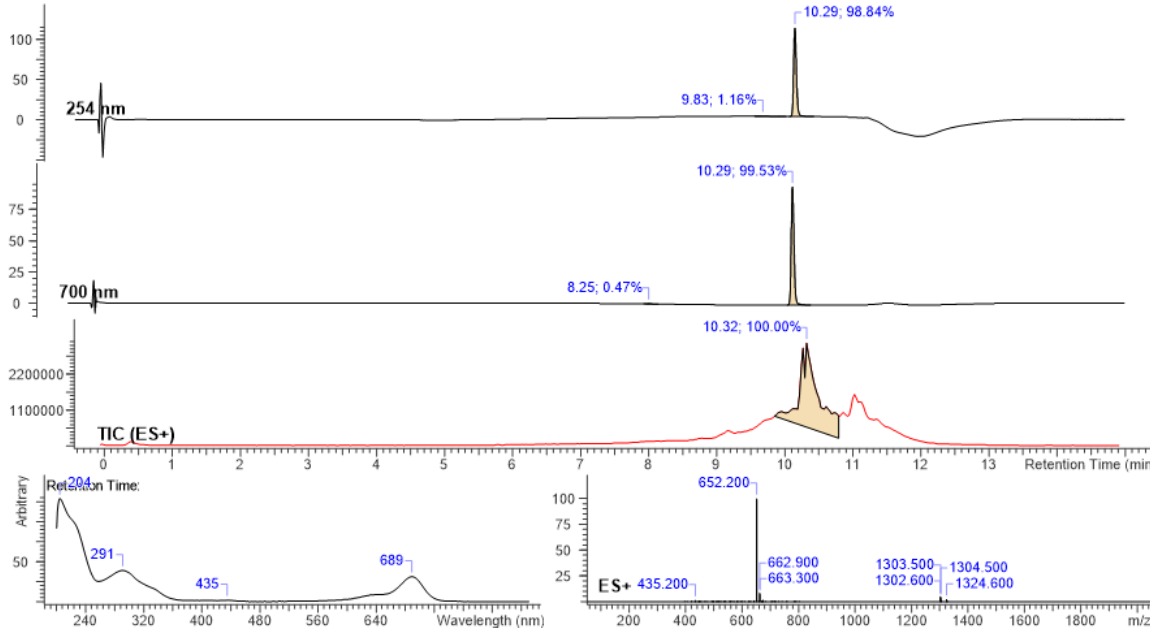

Supplementary Figure 100. LC/MS analysis results of compound **98**.

4-700SiR-PepA (**99**):

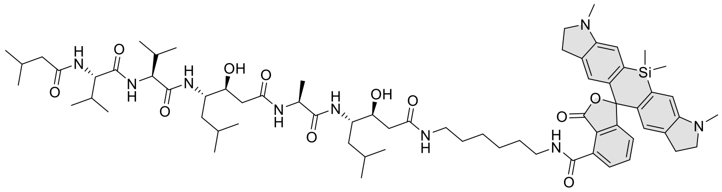

Compound was purified by preparative HPLC (solvent A: H_2O +0.2% HCOOH, solvent B: MeCN; temperature 25°C, gradient A:B - 3 min 70:30 isocratic, 4-20 min 70:30 to 0:100 gradient, 20-25 min 0:100 isocratic, 40 mL/min flow). Fractions containing the product were collected, evaporated and lyophilized from acetonitrile water mixture. The obtained solid was dissolved in 700 µL of d_6-DMSO. The samples from DMSO solution were diluted x100 in PBS +0.1% SDS and concentration was determined spectroscopically with Nanodrop. Determined stock concentration was 2.8 mM which constitutes to 39% yield (2.5 mg).

^{1}H NMR (400 MHz, d_6-DMSO) δ 9.04 (t, J = 5.6 Hz, 1H), 7.92 (d, J = 7.4 Hz, 1H), 7.82 (d, J = 8.8 Hz, 1H), 7.78 (d, J = 9.0 Hz, 1H), 7.75 – 7.67 (m, 3H), 7.46 (d, J = 8.8 Hz, 1H), 7.33 (d, J = 9.2 Hz, 1H), 7.15 (dd, J = 6.5, 2.2 Hz, 1H), 6.78 (s, 2H), 6.58 (s, 2H), 4.84 (dd, J = 8.8, 5.0 Hz, 2H), 4.25 (t, J = 7.2 Hz, 1H), 4.18 (t, J = 9.4, 7.3 Hz, 1H), 4.12 (dd, J = 9.4, 7.1 Hz, 1H), 3.86 – 3.74 (m, 4H), 3.29 – 3.14 (m, 6H), 3.07 – 2.99 (m, 2H), 2.86 – 2.62 (m, 10H), 2.13 – 1.90 (m, 9H), 1.64 – 1.08 (m, 23H), 0.88 – 0.74 (m, 24H), 0.57 (s, 3H), 0.49 (s, 3H). ESI-MS, positive mode: m/z = 1284.7 $[M+Na]^+$. HRMS (ESI) calcd for $C_{69}H_{103}N_9O_{11}SiNa$ $[M+Na]^+$ 1284.7439, found 1284.7433.

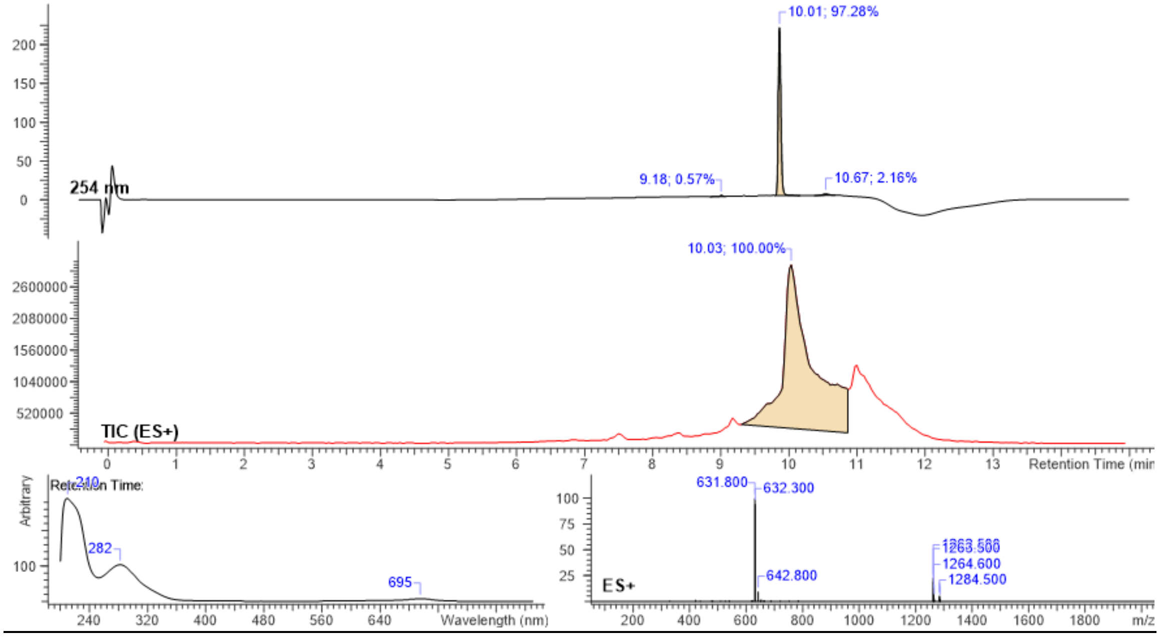

Supplementary Figure 101. LC/MS analysis results of compound **99**.

General procedure for the synthesis of compounds **100**-**104**:

Into a solution of corresponding rhodamine dye (1 µmol, 2.7 eq; **3**, **4**, **11**, **15**, **22**), **des-bromo-des-methyl-Lys-jasplakinolide**[29] (250 µg, 0.37 µmol ,1.0 eq, unless stated otherwise) and DIPEA (5 µL) in DMSO (50 µL) a solution of HATU (1.3 µmol, 1.3 eq) in DMSO (20 µL) was added and the mixture was mixed for 30 min. Reactions were usually over after 30 min and their course was monitored by LC/MS analysis. Once reaction was finished it was quenched with 10 µL of formic acid and diluted with water and acetonitrile to 1 mL volume and was further purified by the means of preparative HPLC (preparative column: Agilent 5 Prep-C18, 5 µm, 100 x 50 mm).

4-505R-JAS (100):

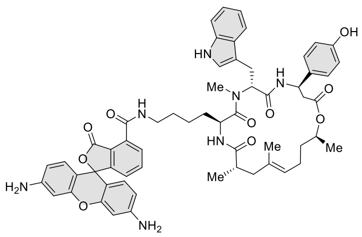

Compound was purified by preparative HPLC (solvent A: H_2O 10 mM NH_4COOH pH = 3.6, solvent B: MeCN; temperature 25°C, gradient A:B - 3 min 70:30 isocratic, 4-20 min 70:30 to 0:100 gradient, 20-25 min 0:100 isocratic, 40 mL/min flow). Fractions containing the product were collected, evaporated and lyophilized from acetonitrile water mixture. The obtained solid was dissolved in 600 µL of *d₆*-DMSO. The samples from DMSO solution were diluted x100 in PBS +0.1% SDS and concentration was determined spectroscopically with Nanodrop. Determined stock concentration was 0.31 mM which constitutes to 50% yield (192 µg).

^{1}H NMR (600 MHz, d_6-DMSO) δ 10.81 (s, 1H), 9.30 (s, 1H), 9.03 (t, *J* = 5.7 Hz, 1H), 8.65 (d, *J* = 8.8 Hz, 1H), 7.85 (dd, *J* = 7.5, 1.0 Hz, 1H), 7.76 (t, *J* = 7.6 Hz, 1H), 7.69 – 7.67 (m, 1H), 7.33 (d, *J* = 8.0 Hz, 1H), 7.25 (dd, *J* = 7.7, 1.0 Hz, 1H), 7.13 (d, *J* = 8.7 Hz, 2H), 7.09 (d, *J* = 2.3 Hz, 1H), 7.04 (t, *J* = 7.2 Hz, 1H), 6.95 (t, *J* = 7.5 Hz, 1H), 6.69 (d, *J* = 8.6 Hz, 2H), 6.53 (s, 1H), 6.43 (dd, *J* = 8.5, 6.9 Hz, 2H), 6.38 (d, *J* = 2.2 Hz, 2H), 6.29 (dd, *J* = 8.4, 2.2 Hz, 2H), 5.59 (s, 4H), 5.53 (dd, *J* = 11.5, 5.0 Hz, 1H), 5.46 (d, *J* = 9.5 Hz, 1H), 5.18 (t, *J* = 11.3 Hz, 1H), 4.92 (t, *J* = 7.0 Hz, 1H), 4.67 (h, *J* = 6.5 Hz, 1H), 4.58 (td, *J* = 8.8, 3.8 Hz, 1H), 3.19 – 3.01 (m, 6H), 2.93 (dd, *J* = 15.0, 4.9 Hz, 1H), 2.68 (dd, *J* = 14.8, 11.4 Hz, 1H), 2.62 – 2.57 (m, 2H), 2.40 – 2.37 (m, 1H), 2.18 – 2.13 (m, 1H), 1.84 (q, *J* = 16.0, 12.4 Hz, 2H), 1.70 (d, *J* = 15.0 Hz, 1H), 1.48 (s, 3H), 1.38 – 1.31 (m, 2H), 1.16 (d, *J* = 6.3 Hz, 3H), 0.98 – 0.92 (m, 2H), 0.88 – 0.79 (m, 5H), 0.76 – 0.67 (m, 2H). ESI-MS, positive mode: m/z = 1030.4 $[M+H]^+$. HRMS (ESI) calcd for $C_{59}H_{64}N_7O_{10}$ $[M+H]^+$ 1030.4709, found 1030.4714.

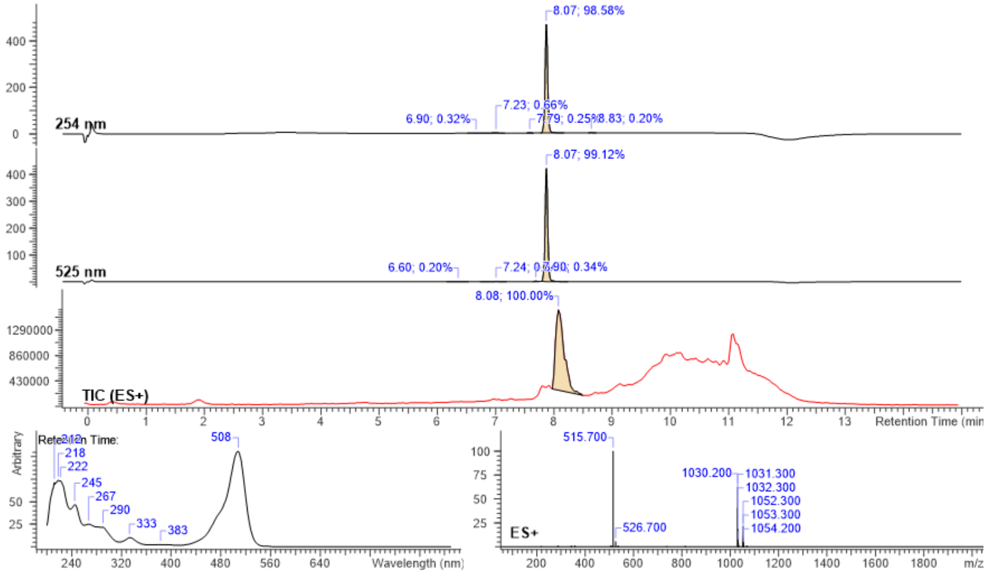

Supplementary Figure 102. LC/MS analysis results of compound **100**.

4-TMR-JAS (101):

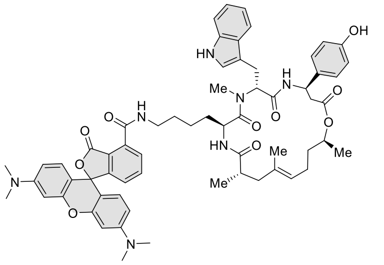

Compound was purified by preparative HPLC (solvent A: H_2O + 10 mM NH_4COOH pH = 3.6 B: MeCN; temperature 25°C, gradient A:B - 3 min 70:30 isocratic, 4-20 min 70:30 to 0:100 gradient, 20-25 min 0:100 isocratic, 40 mL/min flow). Fractions containing the product were collected, evaporated and lyophilized from acetonitrile water mixture. The obtained solid was dissolved in 600 µL of d_6-DMSO. The samples from DMSO solution were diluted x100 in PBS +0.1% SDS and concentration was determined spectroscopically with Nanodrop. Determined stock concentration was 0.26 mM which constitutes to 42% yield (169 µg).

^{1}H NMR (600 MHz, d_6-DMSO) δ 10.82 (d, *J* = 2.3 Hz, 1H), 9.30 (s, 1H), 8.97 (t, *J* = 5.6 Hz, 1H), 8.66 (d, *J* = 8.9 Hz, 1H), 7.85 (dd, *J* = 7.5, 1.0 Hz, 1H), 7.76 (t, *J* = 7.6 Hz, 1H), 7.71 (d, *J* = 8.7 Hz, 1H), 7.69 (d, *J* = 8.0 Hz, 1H), 7.34 (d, *J* = 8.3 Hz, 1H), 7.24 (dd, *J* = 7.7, 1.0 Hz, 1H), 7.13 (d, *J* = 8.7 Hz, 2H), 7.10 (d, *J* = 2.4 Hz, 1H), 7.05 (t, *J* = 7.3 Hz, 1H), 6.96 (t, *J* = 7.7 Hz, 1H), 6.70 (d, *J* = 8.6 Hz, 2H), 6.62 (dd, *J* = 8.8, 3.0 Hz, 2H), 6.52 – 6.44 (m, 4H), 5.54 (dd, *J* = 11.6, 4.9 Hz, 1H), 5.21 – 5.16 (m, 1H), 4.92 (t, *J* = 7.0 Hz, 1H), 4.69 – 4.65 (m, 1H), 4.57 (td, *J* = 9.0, 3.8 Hz, 1H), 3.28 – 3.23 (m, 2H), 3.08 – 3.00 (m, 5H), 2.94 (d, *J* = 3.3 Hz, 12H), 2.68 (dd, *J* = 14.7, 11.6 Hz, 1H), 2.63 – 2.55 (m, 2H), 2.45 (d, *J* = 21.7 Hz, 2H), 2.39 (p, *J* = 1.8 Hz, 1H), 2.18 – 2.13 (m, 1H), 1.89 – 1.79 (m, 2H), 1.66 (d, *J* = 15.2 Hz, 2H), 1.48 (s, 3H), 1.39 – 1.34 (m, 2H), 1.16 (d, *J* = 6.3 Hz, 3H), 0.99 – 0.92 (m, 2H), 0.80 (d, *J* = 6.8 Hz, 3H). ESI-MS, positive mode: m/z = 1086.5 $[M+H]^+$. HRMS (ESI) calcd for $C_{63}H_{72}N_7O_{10}$ $[M+H]^+$ 1086.5335, found 1086.5297.

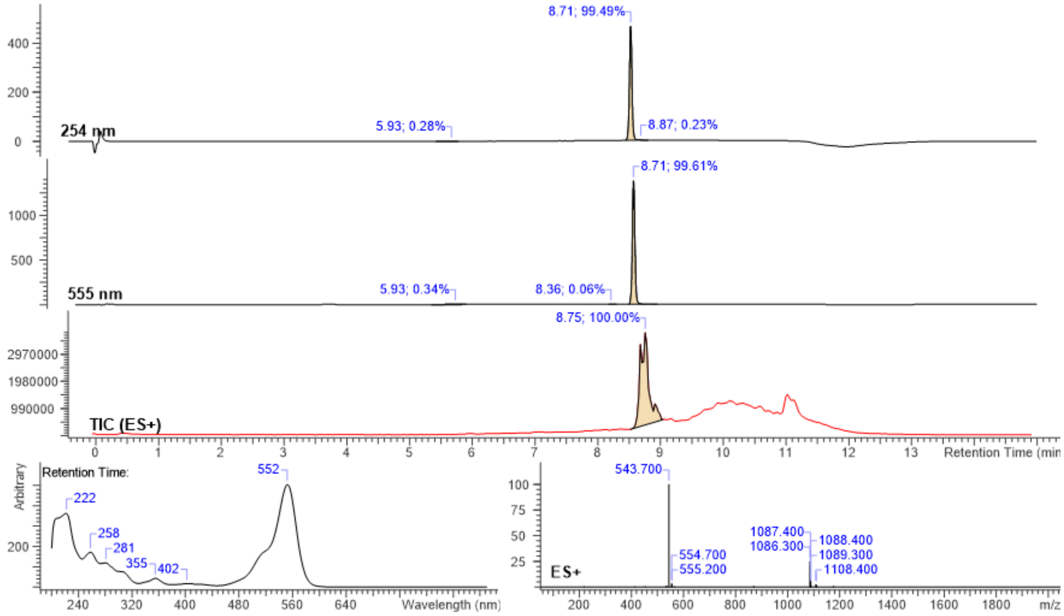

Supplementary Figure 103. LC/MS analysis results of compound **101**.

4-610CP-JAS (**102**):

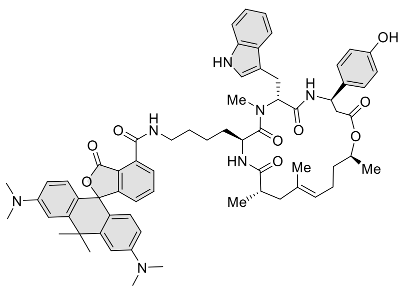

The reaction was carried from 100 µg of **des-bromo-des-methyl-Lys-jasplakinolide**[29] (0.15 µmol). Compound was purified by preparative HPLC (solvent A: H_2O + 10 mM NH_4COOH pH = 3.6, solvent B: MeCN; temperature 25°C, gradient A:B - 3 min 70:30 isocratic, 4-20 min 60:400 to 0:100 gradient, 20-25 min 0:100 isocratic, 40 mL/min flow). Fractions containing the product were collected, evaporated and lyophilized from acetonitrile water mixture. The obtained solid was dissolved in 100 µL of d_6-DMSO. The samples from DMSO solution were diluted x100 in PBS +0.1% SDS and concentration was determined spectroscopically with Nanodrop. Determined stock concentration was 0.42 mM which constitutes to 28% yield (46 µg).

The amount of product was not sufficient to obtain reasonable ^{1}H NMR spectra. Therefore, copies of LC/MS and HRMS are provided to support molecular structure and purity of the product. ESI-MS, positive mode: m/z = 1112.5 $[M+H]^+$. HRMS (ESI) calcd for $C_{66}H_{78}N_7O_9$ $[M+H]^+$ 1112.5856, found 1112.5848.

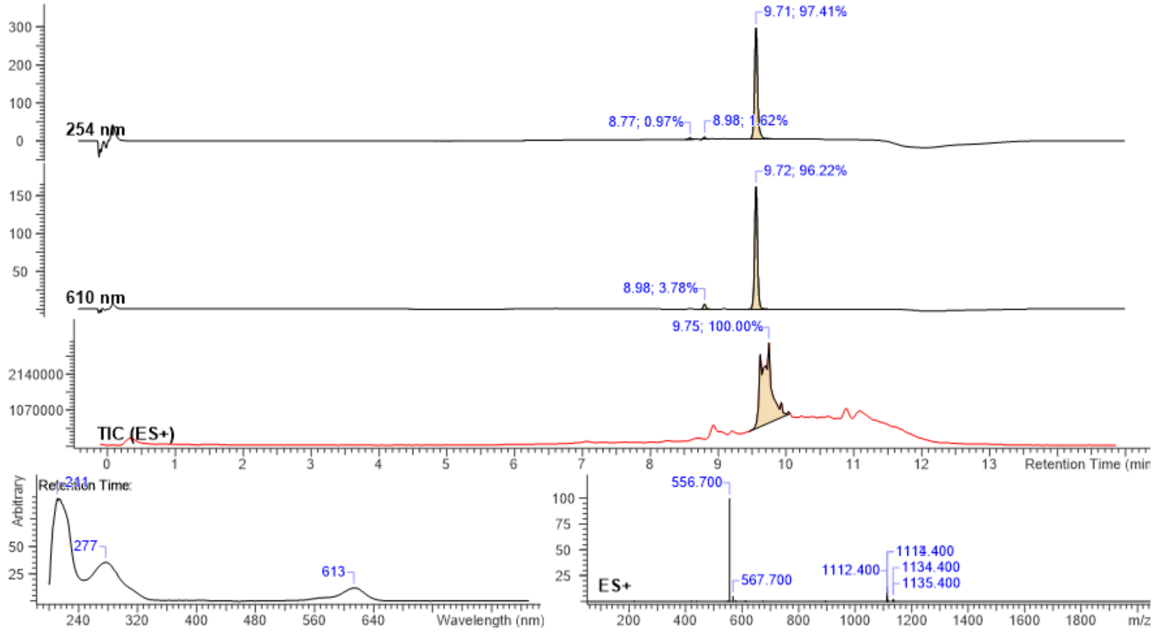

Supplementary Figure 104. LC/MS analysis results of compound **102**.

4-630CP-JAS (**103**):

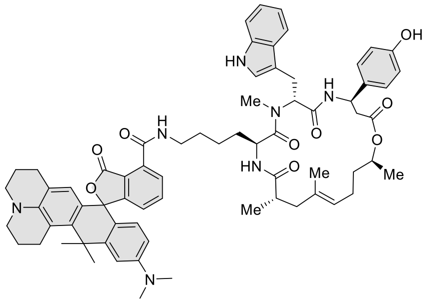

Compound was purified by preparative HPLC (solvent A: H_2O + 10 mM NH_4COOH pH = 3.6 B: MeCN; temperature 25°C, gradient A:B - 3 min 70:30 isocratic, 4-20 min 70:30 to 0:100 gradient, 20-25 min 0:100 isocratic, 40 mL/min flow). Fractions containing the product were collected, evaporated and lyophilized from acetonitrile water mixture. The obtained solid was dissolved in 600 µL of d_6-DMSO. The samples from DMSO solution were diluted x100 in PBS +0.1% SDS and concentration was determined spectroscopically with Nanodrop. Determined stock concentration was 0.28 mM which constitutes to 45% yield (194 µg).

^{1}H NMR (600 MHz, d_6-DMSO) δ 10.82 (s, 1H), 9.30 (s, 1H), 9.16 – 9.08 (m, 1H), 8.66 (dd, *J* = 8.8, 4.5 Hz, 1H), 7.79 (dd, *J* = 9.9, 7.4 Hz, 1H), 7.72 (dd, *J* = 8.7, 4.2 Hz, 1H), 7.70 – 7.63 (m, 2H), 7.34 (d, *J* = 8.1 Hz, 1H), 7.13 (d, *J* = 8.5 Hz, 2H), 7.10 (d, *J* = 2.3 Hz, 1H), 7.05 (t, *J* = 7.5 Hz, 1H), 7.00 (d, *J* = 7.8 Hz, 1H), 6.96 (t, *J* = 7.5 Hz, 1H), 6.80 (d, *J* = 2.5 Hz, 1H), 6.69 (d, *J* = 8.5 Hz, 2H), 6.55 – 6.49 (m, 2H), 6.14 (d, *J* = 2.8 Hz, 1H), 5.54 (dd, *J* = 11.6, 5.0 Hz, 1H), 5.18 (t, *J* = 11.3 Hz, 1H), 4.92 (t, *J* = 7.4 Hz, 1H), 4.67 (h, *J* = 6.8, 6.1, 6.0 Hz, 1H), 4.61 – 4.56 (m, 1H), 3.26 – 3.23 (m, 2H), 3.15 (t, *J* = 17.9, 6.2 Hz, 4H), 3.10 – 3.03 (m, 4H), 2.96 – 2.87 (m, 7H), 2.68 (dd, *J* = 14.6, 11.5 Hz, 1H), 2.62 – 2.58 (m, 2H), 2.54 – 2.52 (m, 4H), 2.47 (q, *J* = 1.9 Hz, 2H), 2.39 (q, *J* = 1.9 Hz, 1H), 2.18 – 2.13 (m, 1H), 1.96 – 1.86 (m, 5H), 1.83 (s, 3H), 1.54 – 1.43 (m, 5H), 1.40 – 1.34 (m, 2H), 1.21 (s, 3H), 1.16 (d, *J* = 6.3 Hz, 3H), 0.99 – 0.95 (m, 2H), 0.85 – 0.80 (m, 4H). ESI-MS, positive mode: m/z = 1164.6 $[M+H]^+$. HRMS (ESI) calcd for $C_{70}H_{82}N_7O_9$ $[M+H]^+$ 1164.6169, found 1164.6155.

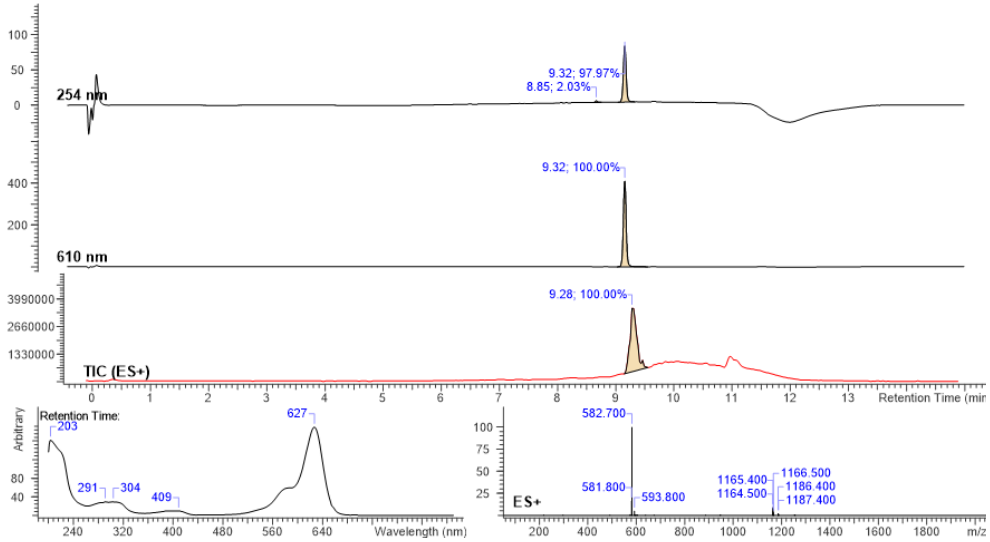

Supplementary Figure 105. LC/MS analysis results of compound **103**.

4-685SiR-JAS (**104**):

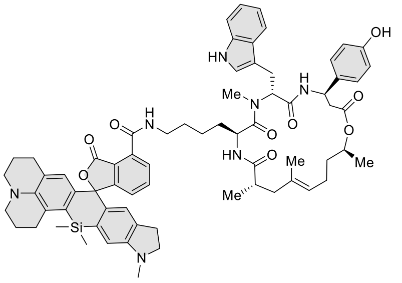

Compound was purified by preparative HPLC (solvent A: H_2O + 10 mM NH_4COOH pH = 3.6 B: MeCN; temperature 25°C, gradient A:B - 3 min 70:30 isocratic, 4-20 min 70:30 to 0:100 gradient, 20-25 min 0:100 isocratic, 40 mL/min flow). Fractions containing the product were collected, evaporated and lyophilized from acetonitrile water mixture. The obtained solid was dissolved in 600 µL of d_6-DMSO. The samples from DMSO solution were diluted x100 in PBS +0.1% SDS and concentration was determined spectroscopically with Nanodrop. Determined stock concentration was 0.24 mM which constitutes to 39% yield (172 µg).

^{1}H NMR (600 MHz, d_6-DMSO) δ 10.81 (s, 1H), 9.30 (s, 1H), 9.07 (t, *J* = 5.7 Hz, 1H), 8.65 (d, *J* = 8.8 Hz, 1H), 7.75 (dd, *J* = 7.5, 1.0 Hz, 1H), 7.70 – 7.64 (m, 3H), 7.34 (d, *J* = 8.1 Hz, 1H), 7.13 (d, *J* = 8.6 Hz, 2H), 7.11 – 7.08 (m, 2H), 7.04 (t, *J* = 7.8 Hz, 1H), 6.95 (t, *J* = 7.6 Hz, 1H), 6.75 (s, 1H), 6.69 (d, *J* = 8.6 Hz, 2H), 6.55 (d, *J* = 5.5 Hz, 1H), 6.33 (d, *J* = 9.6 Hz, 1H), 5.53 (dd, *J* = 11.5, 5.1 Hz, 1H), 5.18 (t, *J* = 12.0, 11.6 Hz, 1H), 4.92 (t, *J* = 7.2 Hz, 1H), 4.67 (h, *J* = 6.4 Hz, 1H), 4.59 (td, *J* = 8.8, 4.1 Hz, 1H), 3.24 – 3.08 (m, 11H), 3.06 (s, 3H), 2.95 (d, *J* = 5.0 Hz, 1H), 2.92 – 2.89 (m, 2H), 2.76 (d, *J* = 1.0 Hz, 3H), 2.73 – 2.65 (m, 3H), 2.62 – 2.58 (m, 2H), 2.47 – 2.42 (m, 2H), 2.38 (p, *J* = 1.9 Hz, 1H), 2.18 – 2.14 (m, 1H), 1.96 – 1.92 (m, 2H), 1.87 – 1.80 (m, 2H), 1.75 (q, *J* = 6.1 Hz, 2H), 1.69 (d, *J* = 15.8 Hz, 1H), 1.48 (s, 3H), 1.40 – 1.34 (m, 2H), 1.28 – 1.26 (m, 2H), 1.21 (s, 3H), 1.16 (d, *J* = 6.4 Hz, 4H), 0.95 (q, *J* = 6.9 Hz, 2H), 0.75 – 0.68 (m, 2H), 0.64 (s, 3H), 0.53 (s, 3H). ESI-MS, positive mode: m/z = 1192.5 $[M+H]^+$. HRMS (ESI) calcd for $C_{70}H_{82}N_7O_9Si$ $[M+H]^+$ 1192.5901, found 1192.5901.

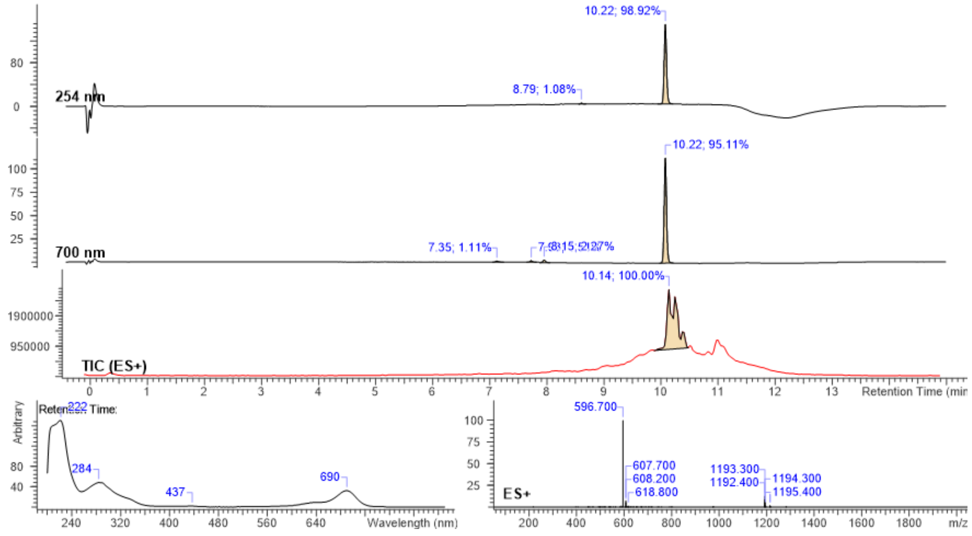

Supplementary Figure 106. LC/MS analysis results of compound **104**.

General procedure for the synthesis of compounds **105**-**109**:

Into a solution of corresponding rhodamine dye (5 µmol, 1 eq;**4, 7, 10, 11, 24**) and DIPEA (5 µL) in DMSO (100 µL) a solution of HATU (6.5 µmol, 1.3 eq) in DMSO (100 µL) was added and the mixture was mixed for 1 min. Then a solution of O6-[4-(aminomethyl)benzyl]guanine (7.5 µmol ,1.5 eq, Sigma Aldrich) in DMSO (100 µL +5 µL DIPEA) was added at once. Reactions were usually over after 30 min and their course was monitored by LC/MS analysis. Once reaction was finished it was quenched with 20 µL of formic acid and diluted with water and acetonitrile to 2 mL volume and was further purified by the means of preparative HPLC (preparative column: Agilent 5 Prep-C18, 5 µm, 100 x 50 mm).

4-580CP-BG (**105**):

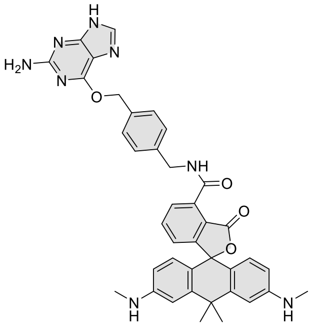

Compound was purified by preparative HPLC (solvent A: H_2O + 10 mM NH_4COOH pH = 3.6 B: MeCN; temperature 25°C, gradient A:B - 3 min 70:30 isocratic, 4-20 min 70:30 to 0:100 gradient, 20-25 min 0:100 isocratic, 40 mL/min flow). Fractions containing the product were collected, evaporated and lyophilized from acetonitrile water mixture. The obtained solid was dissolved in 700 µL of d_6-DMSO. The samples from DMSO solution were diluted x100 in PBS +0.1% SDS and concentration was determined spectroscopically with Nanodrop. Determined stock concentration was 4.2 mM which constitutes to 59% yield (2.0 mg).

^{1}H NMR (400 MHz, d_6-DMSO) δ 12.42 (s, 1H), 9.58 (t, J = 5.7 Hz, 1H), 8.48 (s, 1H), 7.81 – 7.79 (m, 1H), 7.70 (t, J = 7.6 Hz, 1H), 7.50 (s, 4H), 7.06 (dd, J = 7.7, 1.0 Hz, 1H), 6.75 (d, J = 2.3 Hz, 2H), 6.48 (d, J = 8.6 Hz, 2H), 6.37 (dd, J = 8.7, 2.3 Hz, 2H), 6.28 (s, 2H), 5.85 (q, J = 5.1 Hz, 2H), 5.48 (s, 2H), 4.60 (d, J = 5.8 Hz, 2H), 2.70 (d, J = 5.0 Hz, 6H), 1.75 (s, 3H), 1.66 (s, 3H). ESI-MS, positive mode: m/z = 703.3 $[M+Na]^+$. HRMS (ESI) calcd for $C_{39}H_{36}N_8O_4Na$ $[M+Na]^+$ 703.2752, found 703.2726.

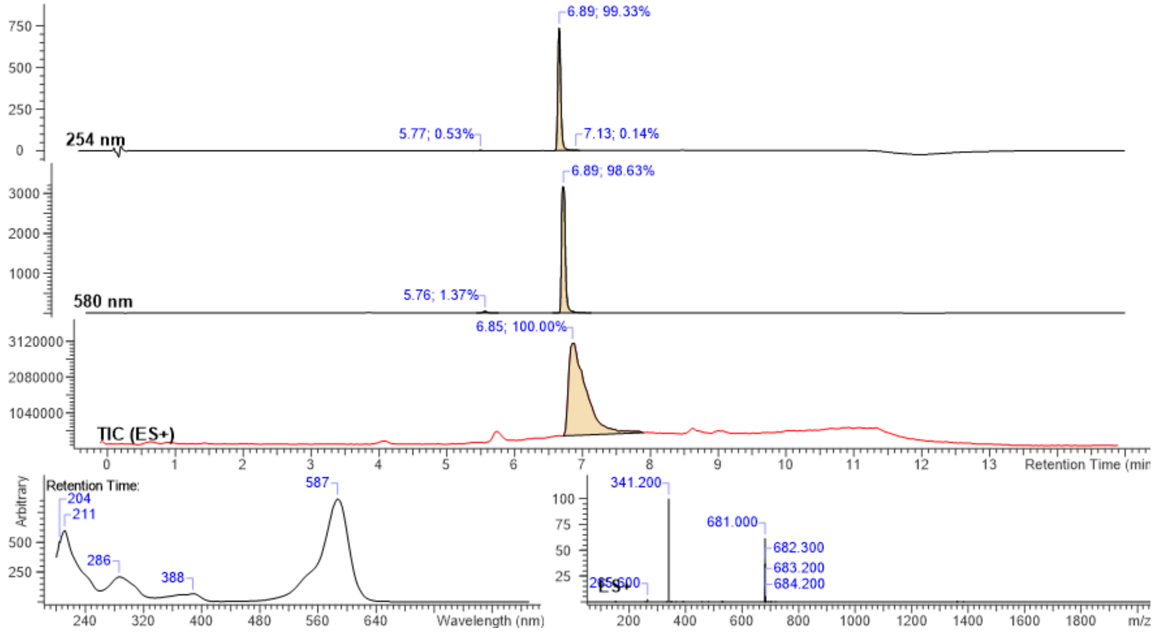

Supplementary Figure 107. LC/MS analysis results of compound **105**.

4-610CP-BG (**106**):

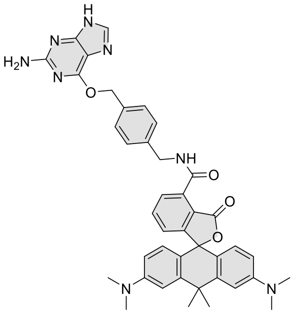

Compound was purified by preparative HPLC (solvent A: H_2O + 10 mM NH_4COOH pH = 3.6 B: MeCN; temperature 25°C, gradient A:B - 3 min 70:30 isocratic, 4-20 min 70:30 to 0:100 gradient, 20-25 min 0:100 isocratic, 40 mL/min flow). Fractions containing the product were collected, evaporated and lyophilized from acetonitrile water mixture. The obtained solid was dissolved in 700 µL of d_6-DMSO. The samples from DMSO solution were diluted x100 in PBS +0.1% SDS and concentration was determined spectroscopically with Nanodrop. Determined stock concentration was 3.2 mM which constitutes to 45% yield (1.6 mg).

^{1}H NMR (400 MHz, d_6-DMSO) δ 12.43 (s, 1H), 9.57 (t, J = 5.8 Hz, 1H), 8.53 (s, 1H), 7.80 (dd, J = 7.5, 1.0 Hz, 1H), 7.70 (t, J = 7.6 Hz, 1H), 7.50 (s, 4H), 7.05 (dd, J = 7.6, 1.0 Hz, 1H), 6.92 – 6.89 (m, 2H), 6.61 – 6.55 (m, 4H), 6.28 (s, 2H), 5.49 (s, 2H), 4.60 (d, J = 5.8 Hz, 2H), 2.94 (s, 12H), 1.82 (s, 3H), 1.72 (s, 3H). ESI-MS, positive mode: m/z = 731.3 $[M+Na]^+$. HRMS (ESI) calcd for $C_{41}H_{40}N_8O_4Na$ $[M+Na]^+$ 731.3065, found 731.3059.

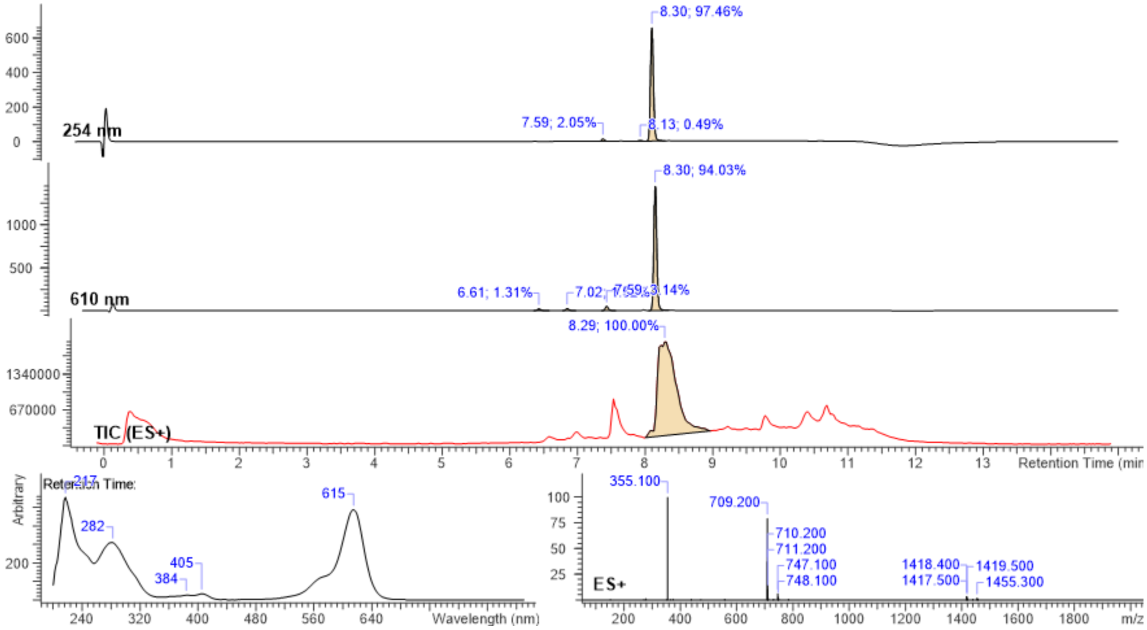

Supplementary Figure 108. LC/MS analysis results of compound **106**.

4-625CP-BG (**107**):

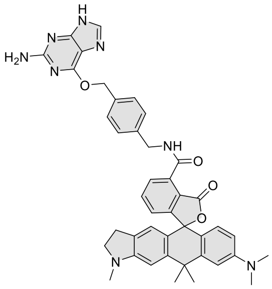

Compound was purified by preparative HPLC (solvent A: H_2O + 10 mM NH_4COOH pH = 3.6 B: MeCN; temperature 25°C, gradient A:B - 3 min 70:30 isocratic, 4-20 min 70:30 to 0:100 gradient, 20-25 min 0:100 isocratic, 40 mL/min flow). Fractions containing the product were collected, evaporated and lyophilized from acetonitrile water mixture. The obtained solid was dissolved in 700 µL of d_6-DMSO. The samples from DMSO solution were diluted x100 in PBS +0.1% SDS and concentration was determined spectroscopically with Nanodrop. Determined stock concentration was 3.8 mM which constitutes to 53% yield (1.9 mg).

^{1}H NMR (400 MHz, d_6-DMSO) δ 9.59 (t, J = 5.8 Hz, 1H), 7.83 – 7.75 (m, 2H), 7.69 (t, J = 7.6 Hz, 1H), 7.51 (s, 4H), 7.04 (dd, J = 7.7, 1.0 Hz, 1H), 6.89 (s, 1H), 6.76 (s, 1H), 6.60 – 6.52 (m, 2H), 6.40 (s, 1H), 6.27 (s, 2H), 5.49 (s, 2H), 4.61 (d, J = 5.8 Hz, 2H), 3.27 – 3.23 (m, 2H), 2.93 (s, 6H), 2.79 (s, 3H), 2.76 – 2.67 (m, 2H), 1.79 (s, 3H), 1.71 (s, 3H). ESI-MS, positive mode: m/z = 721.3 $[M+H]^+$. HRMS (ESI) calcd for $C_{42}H_{41}N_8O_4$ $[M+H]^+$ 721.3245, found 721.3238.

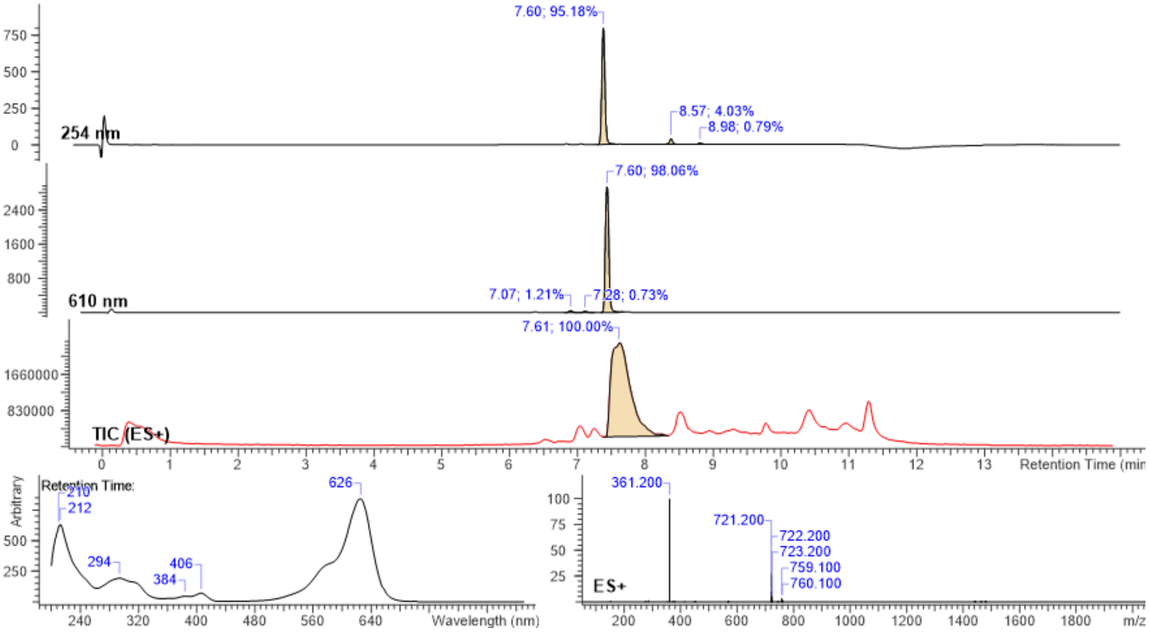

Supplementary Figure 109. LC/MS analysis results of compound **107**.

4-630CP-BG (**108**):

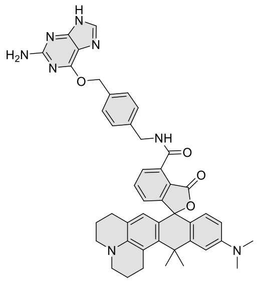

Compound was purified by preparative HPLC (solvent A: H_2O + 10 mM NH_4COOH pH = 3.6 B: MeCN; temperature 25°C, gradient A:B - 3 min 70:30 isocratic, 4-20 min 70:30 to 0:100 gradient, 20-25 min 0:100 isocratic, 40 mL/min flow). Fractions containing the product were collected, evaporated and lyophilized from acetonitrile water mixture. The obtained solid was dissolved in 700 µL of d_6-DMSO. The samples from DMSO solution were diluted x100 in PBS +0.1% SDS and concentration was determined spectroscopically with Nanodrop. Determined stock concentration was 2.3 mM which constitutes to 32% yield (1.2 mg).

^{1}H NMR (400 MHz, d_6-DMSO) δ 12.39 (s, 1H), 9.61 (t, J = 5.7 Hz, 1H), 7.78 (dd, J = 7.5, 1.0 Hz, 2H), 7.67 (t, J = 7.6 Hz, 1H), 7.51 (s, 4H), 7.01 (dd, J = 7.7, 1.0 Hz, 1H), 6.79 (d, J = 2.5 Hz, 1H), 6.56 (dd, J = 8.9, 2.5 Hz, 1H), 6.50 (d, J = 8.9 Hz, 1H), 6.28 (s, 2H), 6.17 (s, 1H), 5.49 (s, 2H), 4.61 (d, J = 5.8 Hz, 2H), 3.20 – 3.11 (m, 4H), 2.99 – 2.86 (m, 8H), 2.47 – 2.36 (m, 2H), 1.94 – 1.89 (m, 2H), 1.89 (s, 3H), 1.83 (s, 3H), 1.79 – 1.72 (m, 2H). ESI-MS, positive mode: m/z = 761.3 [M+H]+. HRMS (ESI) calcd for $C_{45}H_{45}N_8O_4$ $[M+H]^+$ 761.3558, found 761.3545.

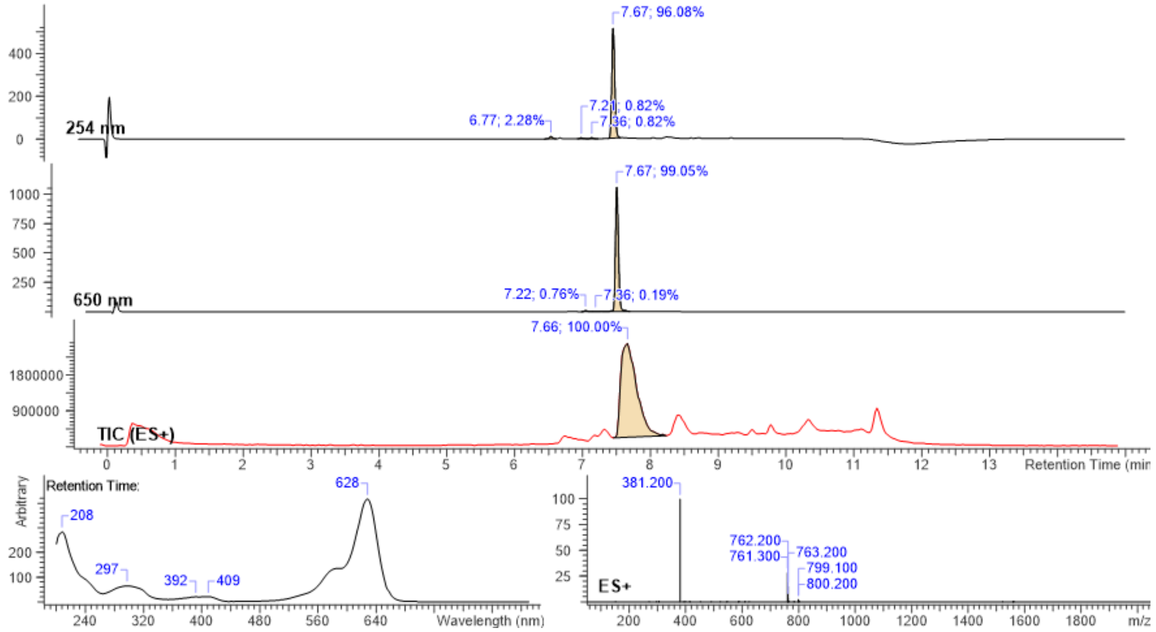

Supplementary Figure 110. LC/MS analysis results of compound **108**.

4-640CP-BG (**109**):

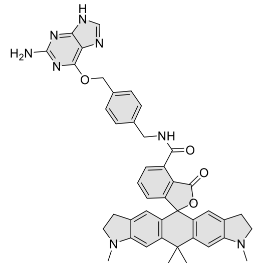

Compound was purified by preparative HPLC (solvent A: H_2O + 10 mM NH_4COOH pH = 3.6 B: MeCN; temperature 25°C, gradient A:B - 3 min 70:30 isocratic, 4-20 min 70:30 to 0:100 gradient, 20-25 min 0:100 isocratic, 40 mL/min flow). Fractions containing the product were collected, evaporated and lyophilized from acetonitrile water mixture. The obtained solid was dissolved in 700 µL of d_6-DMSO. The samples from DMSO solution were diluted x100 in PBS +0.1% SDS and concentration was determined spectroscopically with Nanodrop. Determined stock concentration was 4.0 mM which constitutes to 56% yield (2.0 mg).

^{1}H NMR (400 MHz, d_6-DMSO) δ 9.60 (t, *J* = 5.8 Hz, 1H), 7.82 (s, 1H), 7.79 (dd, *J* = 7.5, 1.0 Hz, 1H), 7.69 (t, *J* = 7.6 Hz, 1H), 7.51 (s, 4H), 7.04 (dd, *J* = 7.7, 1.0 Hz, 1H), 6.74 (s, 2H), 6.37 (s, 2H), 6.26 (s, 2H), 5.49 (s, 2H), 4.61 (d, *J* = 5.8 Hz, 2H), 3.28 – 3.18 (m, 4H), 2.78 (s, 6H), 2.76 – 2.64 (m, 4H), 1.76 (s, 3H), 1.69 (s, 3H). ESI-MS, positive mode: m/z = 733.3 [M+H]+. HRMS (ESI) calcd for $C_{43}H_{41}N_8O_4$ $[M+H]^+$ 733.3245, found 733.3236.

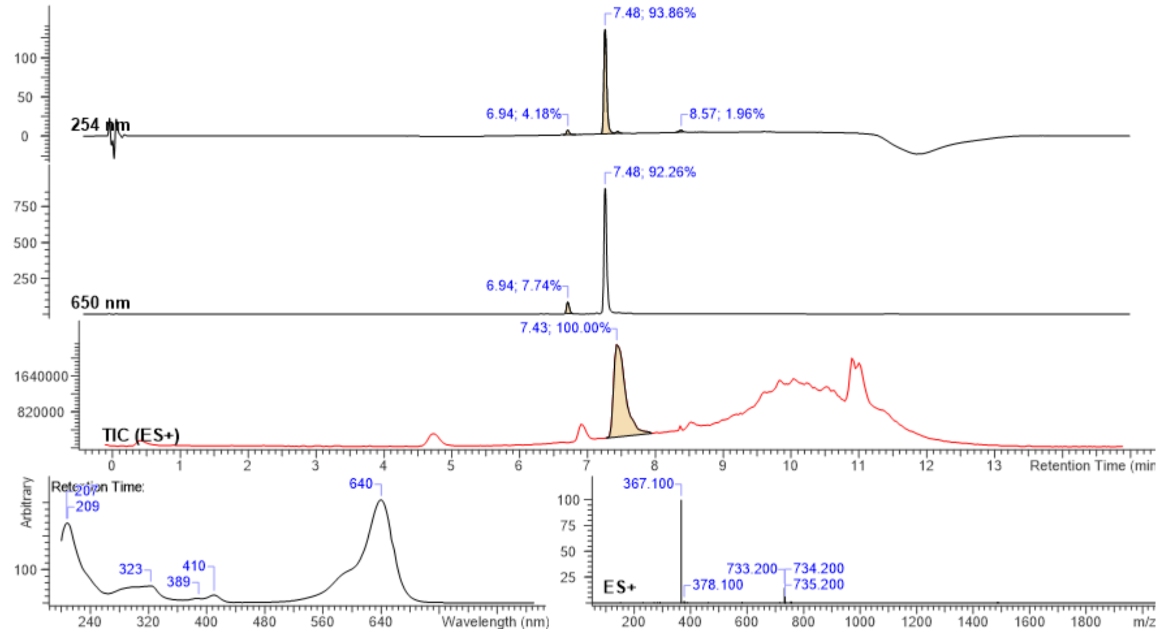

Supplementary Figure 110. LC/MS analysis results of compound **108**.

Copies of NMR spectra

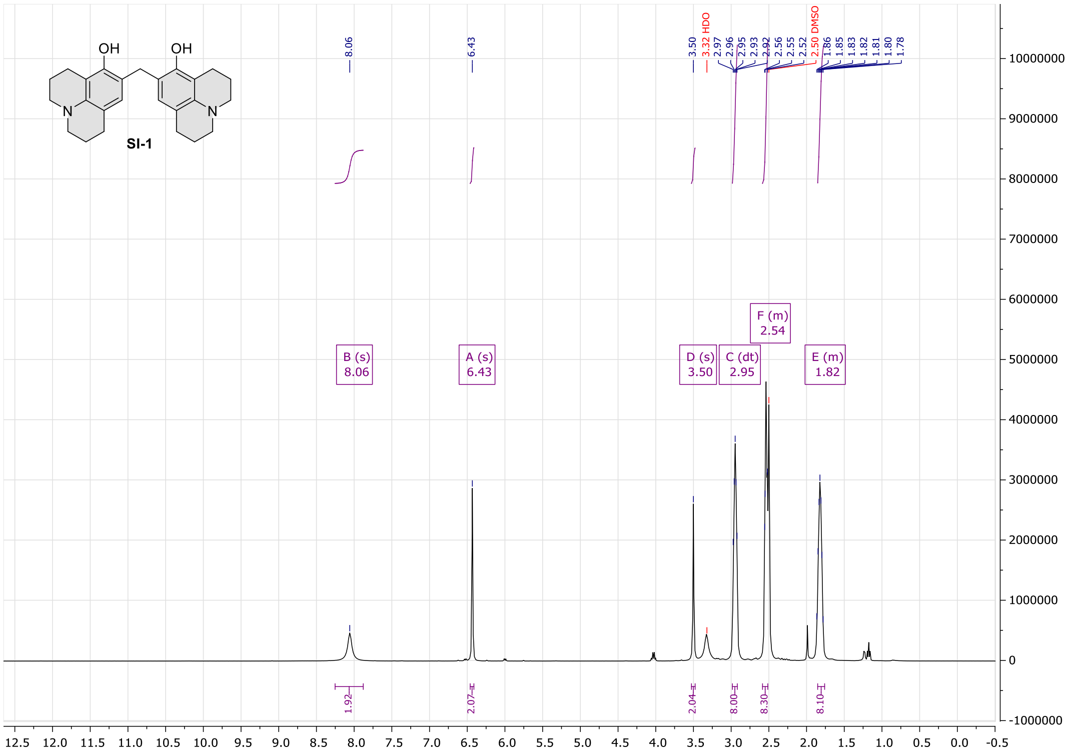

Supplementary Figure 111. ^{1}H NMR of compound **SI-1** (400 MHz, d_6-DMSO).

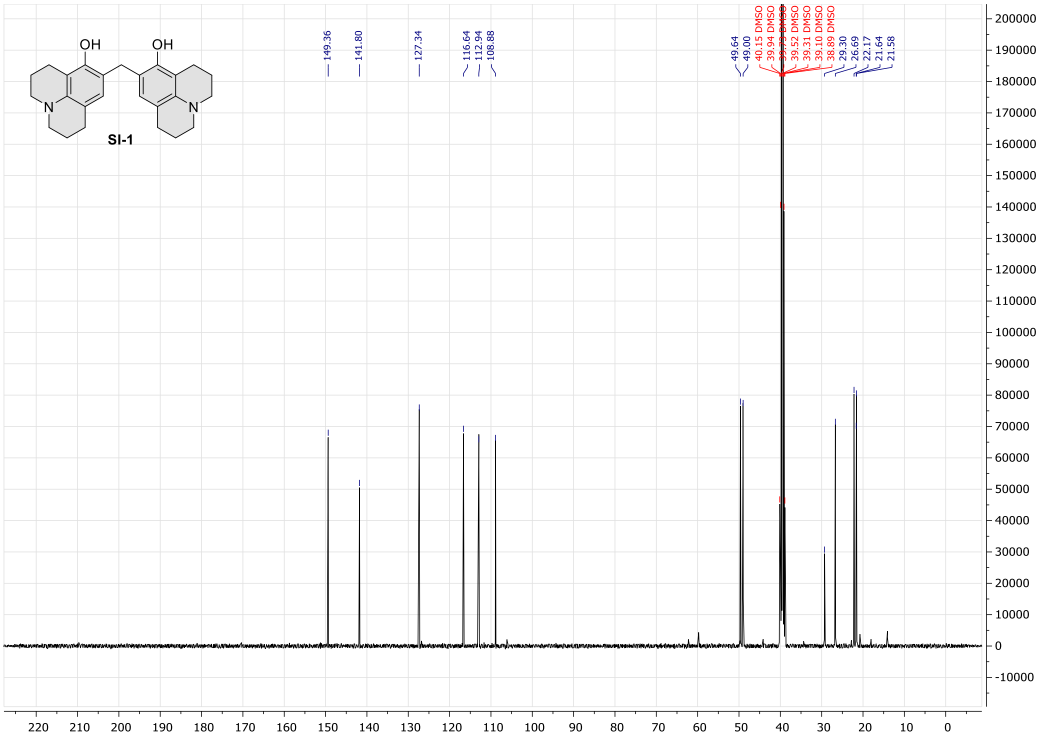

Supplementary Figure 112. ^{13}C NMR of compound **SI-1** (100 MHz, d_6-DMSO).

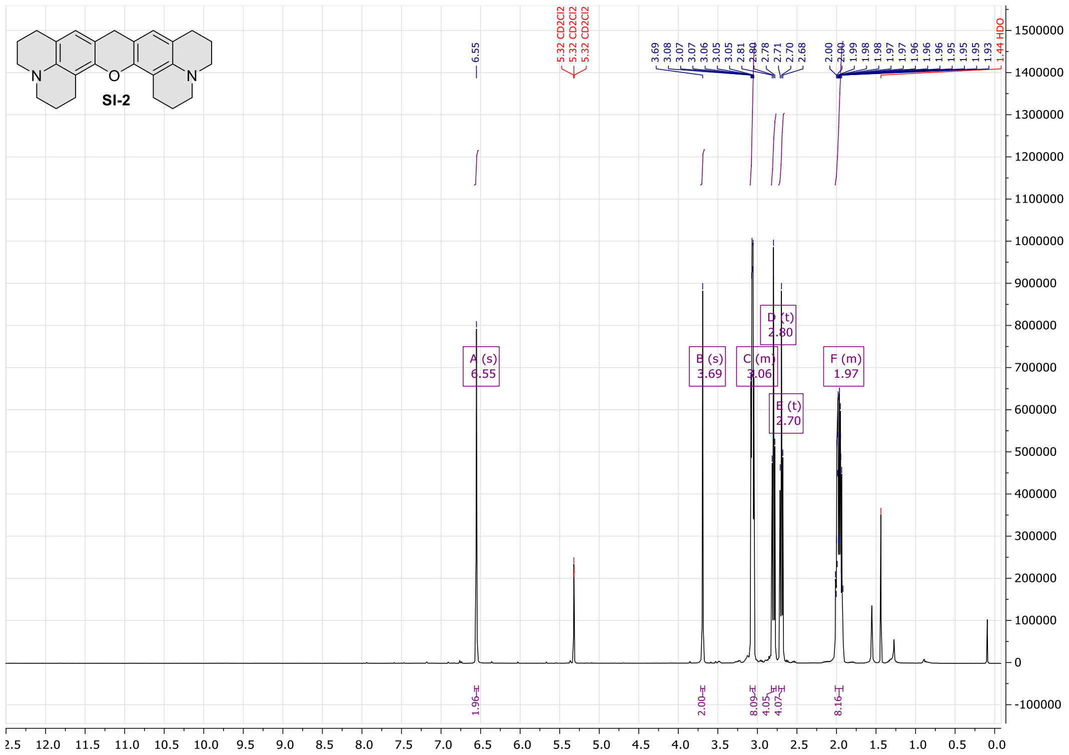

Supplementary Figure 113. ^{1}H NMR of compound **SI-2** (400 MHz, CD_2Cl_2).

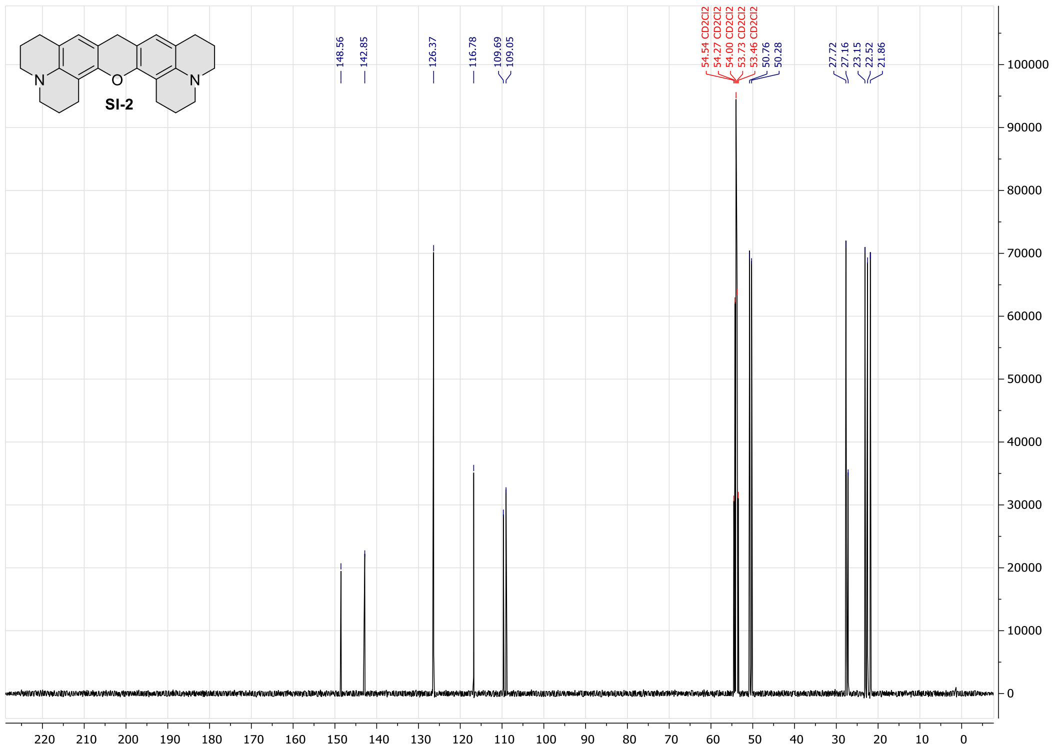

Supplementary Figure 114. ^{13}C NMR of compound **SI-2** (100 MHz, CD_2Cl_2).

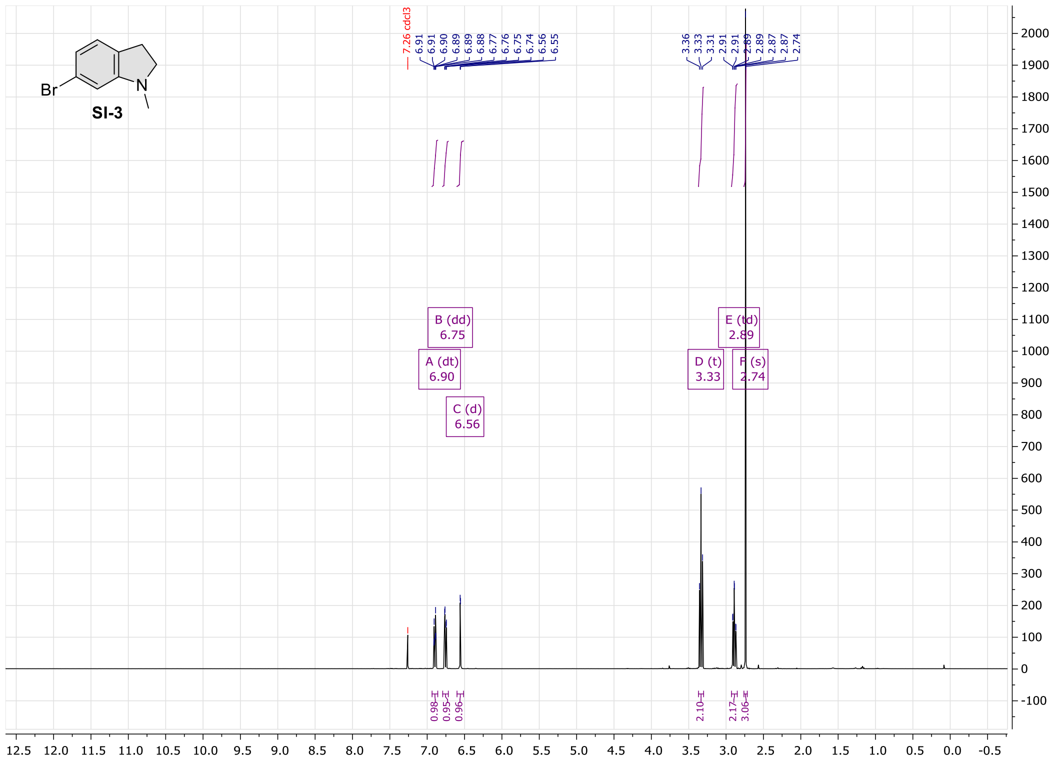

Supplementary Figure 115. ^{1}H NMR of compound **SI-3** (400 MHz, $CDCl_3$).

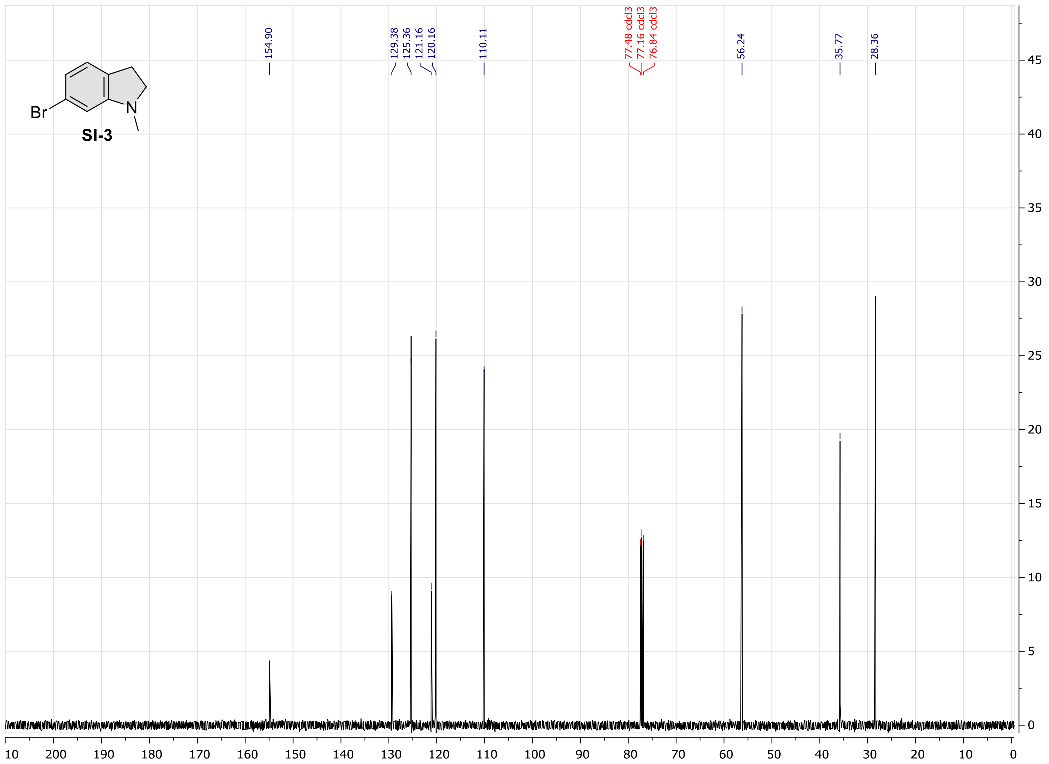

Supplementary Figure 116. ^{13}C NMR of compound **SI-3** (100 MHz, $CDCl_3$).

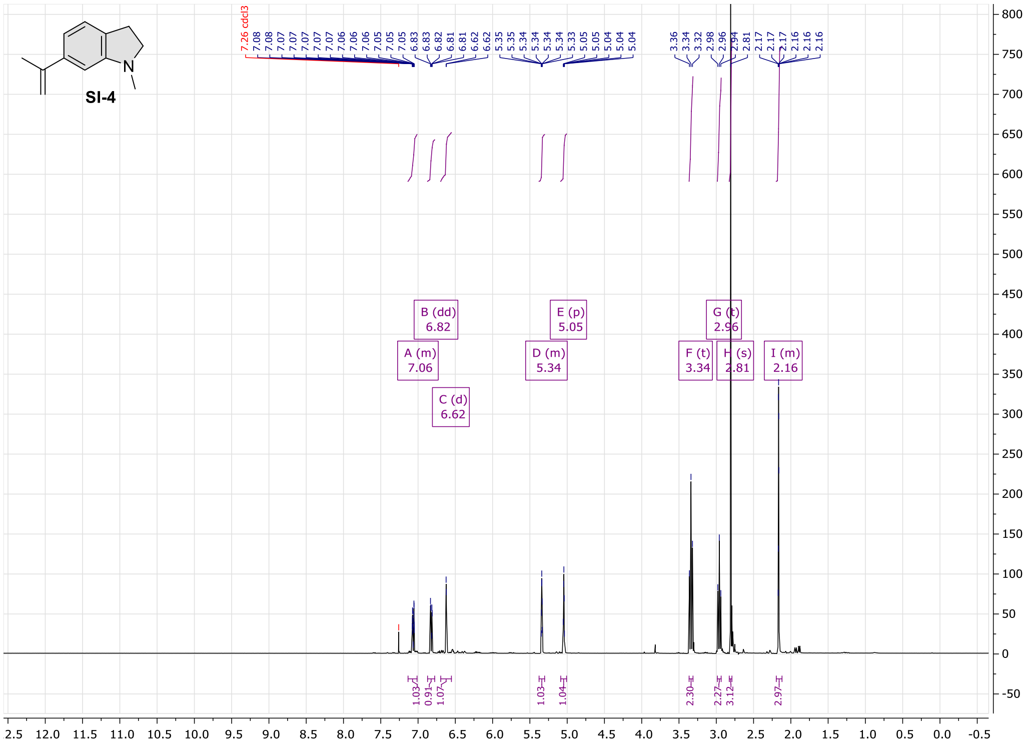

Supplementary Figure 117. ^{1}H NMR of compound **SI-4** (400 MHz, $CDCl_3$).

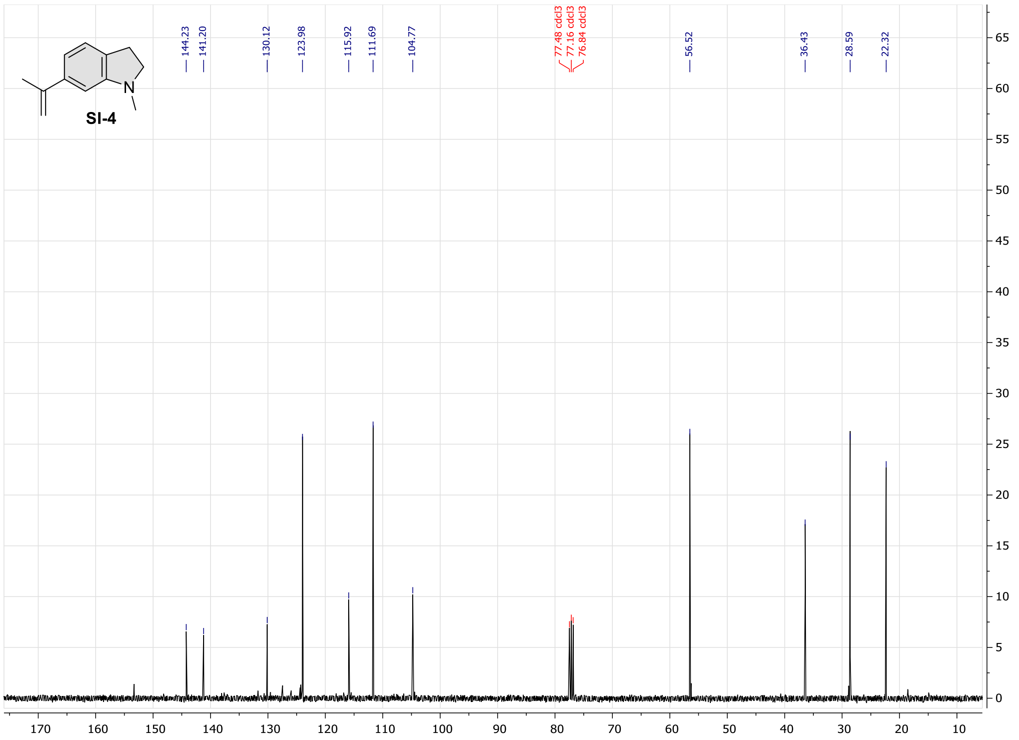

Supplementary Figure 118. ^{13}C NMR of compound **SI-4** (100 MHz, $CDCl_3$).

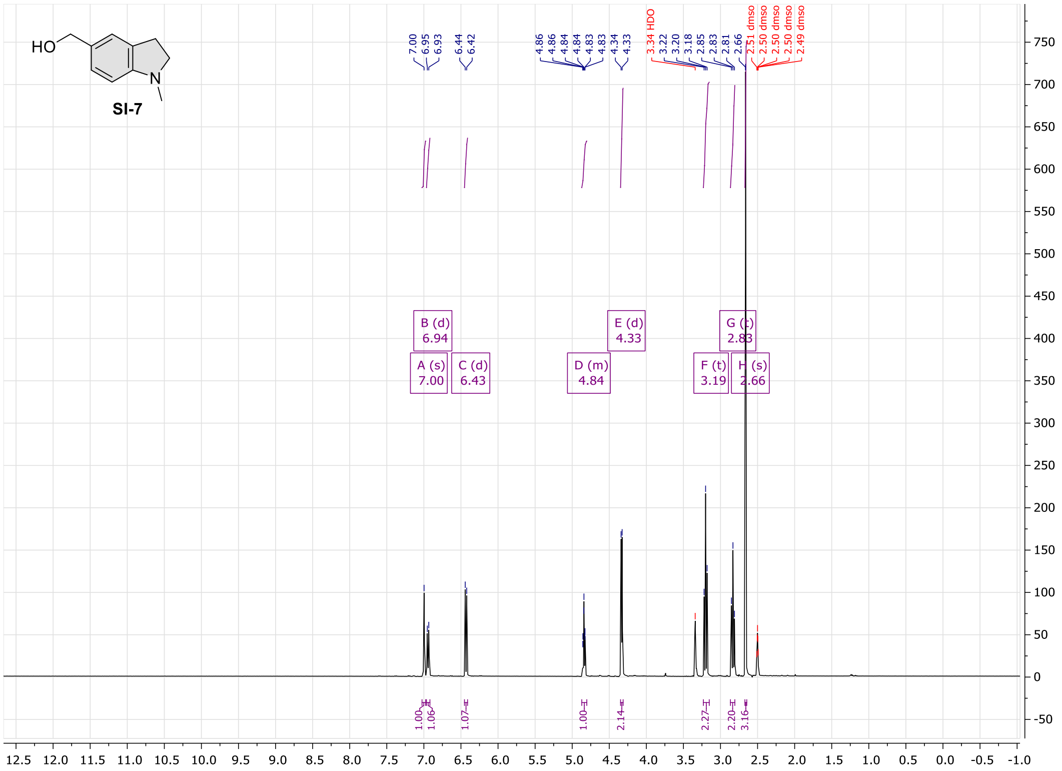

Supplementary Figure 119. ^{1}H NMR of compound **SI-7** (400 MHz, d_6-DMSO).

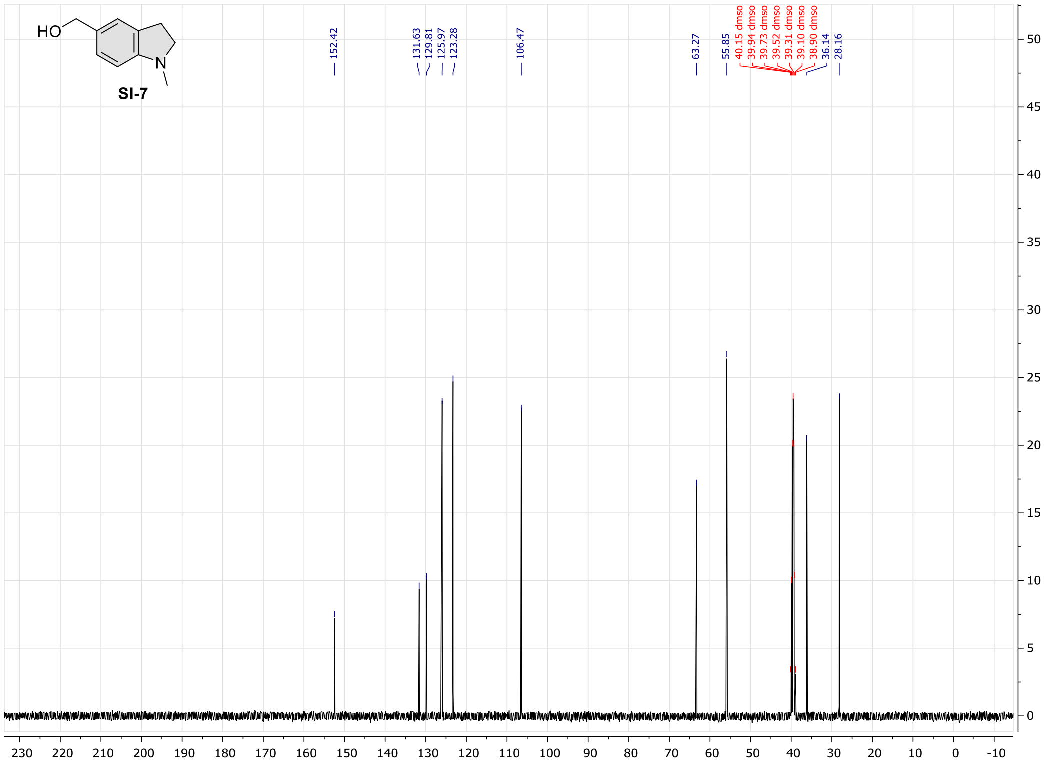

Supplementary Figure 120. ^{13}C NMR of compound **SI-7** (100 MHz, d_6-DMSO).

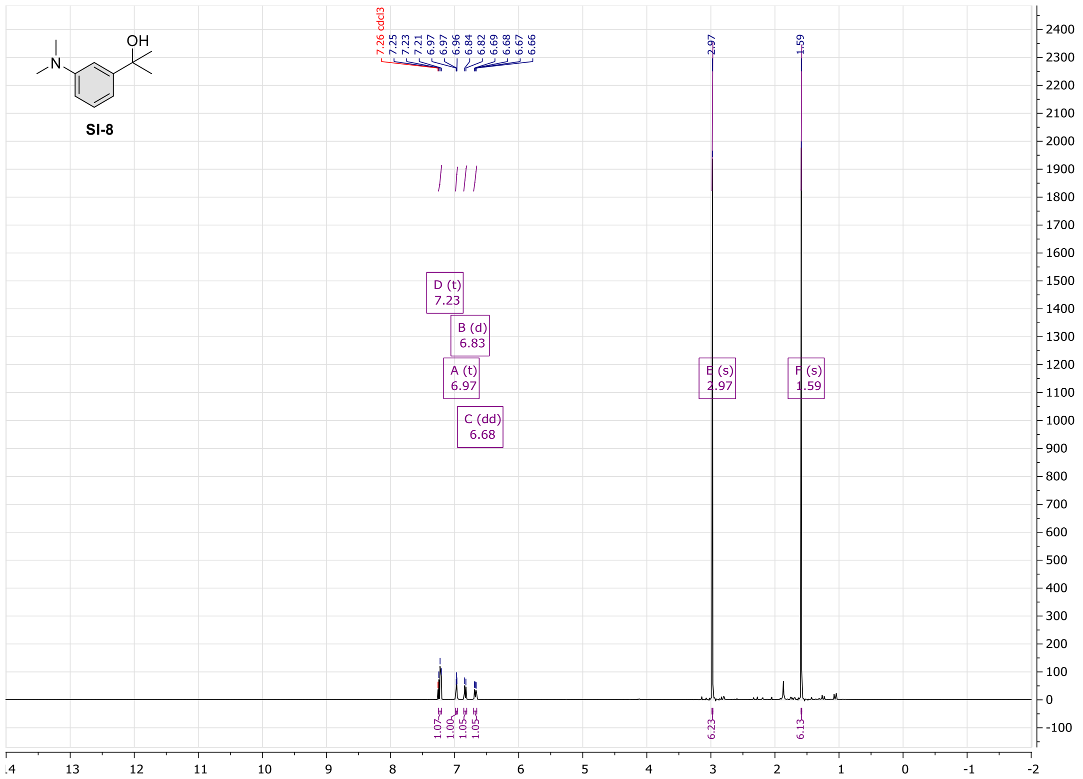

Supplementary Figure 121. ^{1}H NMR of compound **SI-8** (400 MHz, $CDCl_3$).

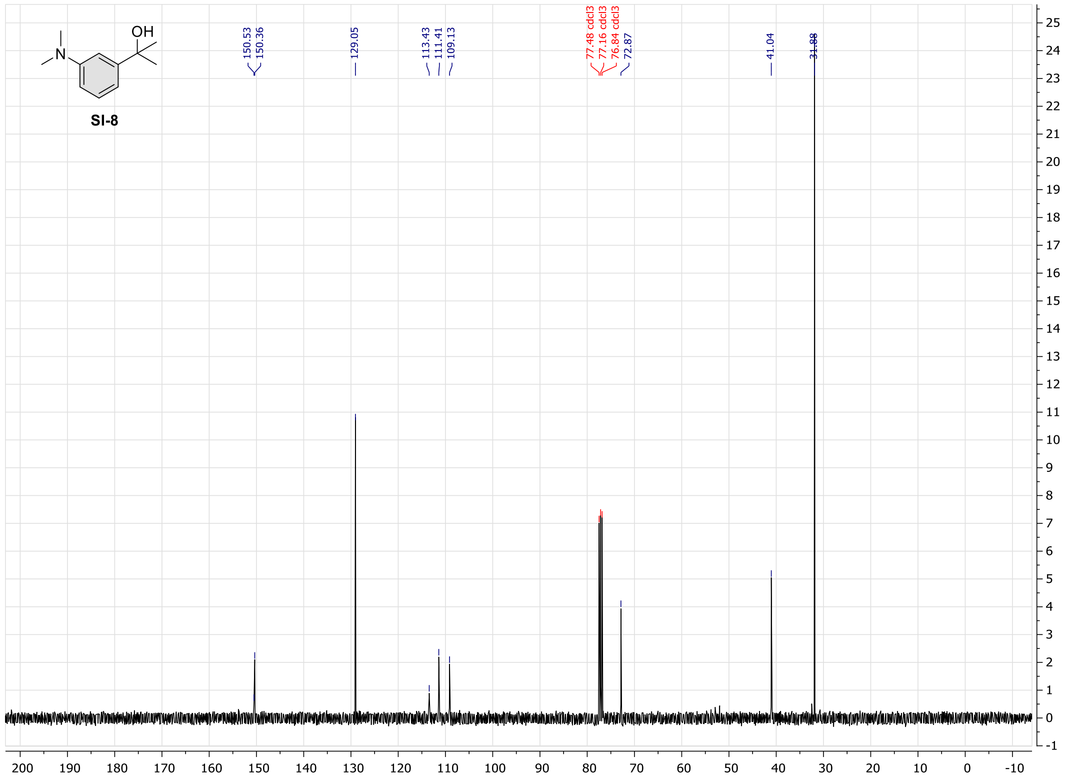

Supplementary Figure 122. ^{13}C NMR of compound **SI-8** (100 MHz, $CDCl_3$).

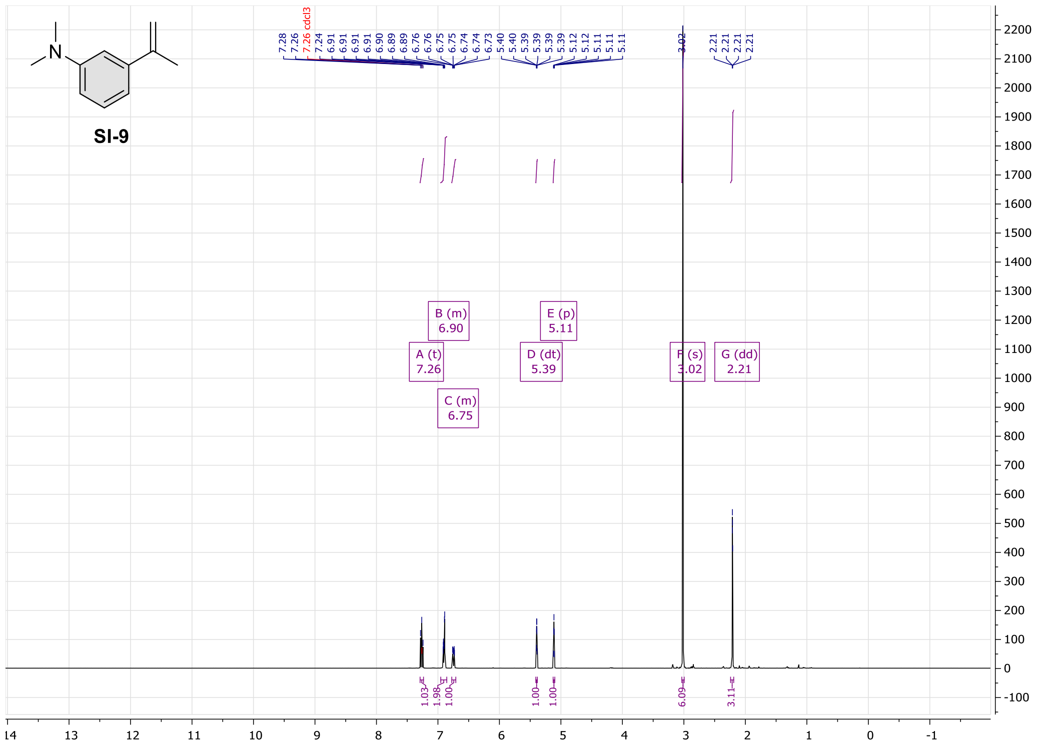

Supplementary Figure 123. ^{1}H NMR of compound **SI-9** (400 MHz, $CDCl_3$).

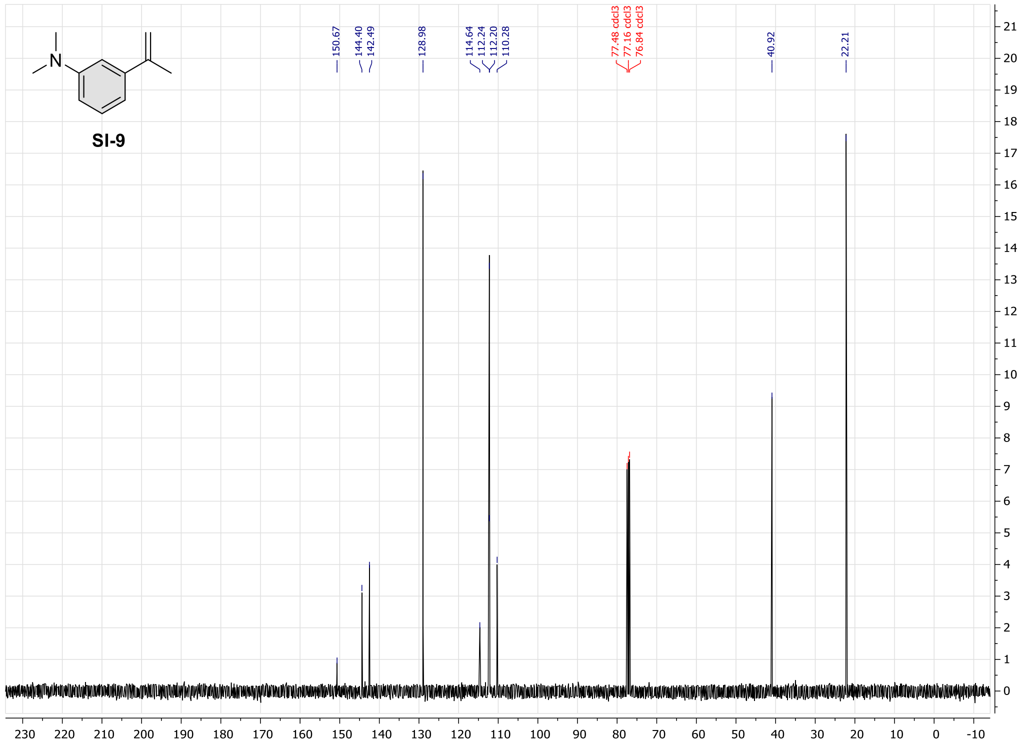

Supplementary Figure 124. ^{13}C NMR of compound **SI-9** (100 MHz, $CDCl_3$).

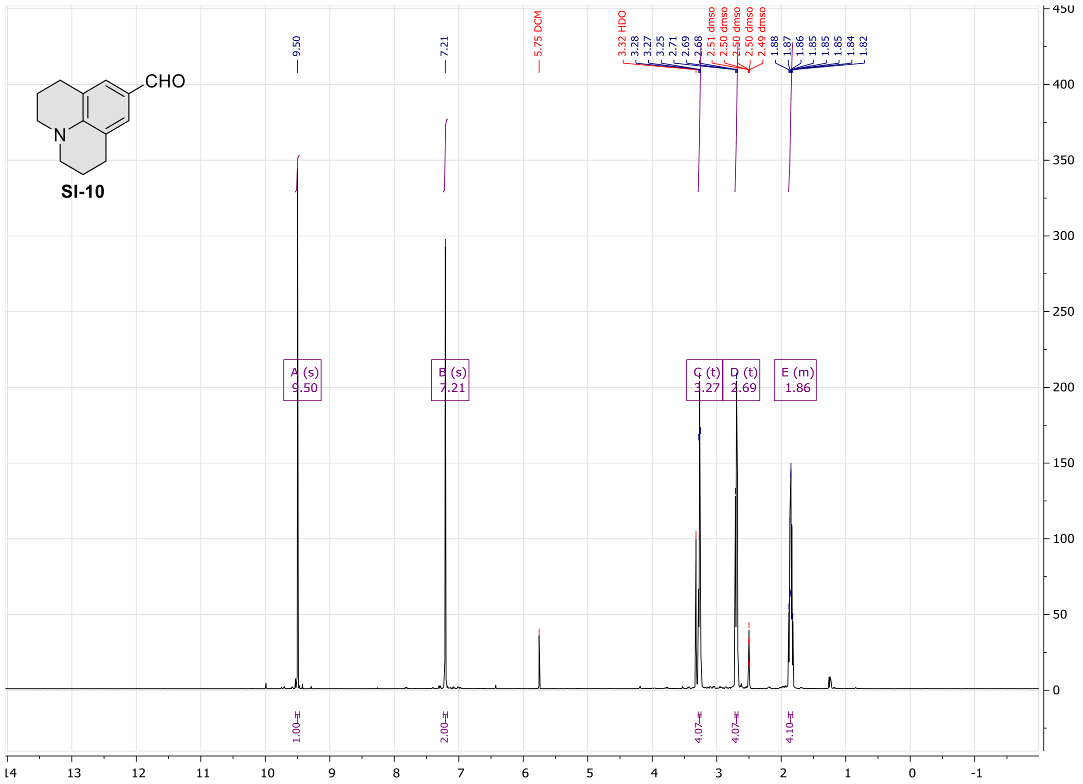

Supplementary Figure 125. ^{1}H NMR of compound **SI-10** (400 MHz, d_6-DMSO).

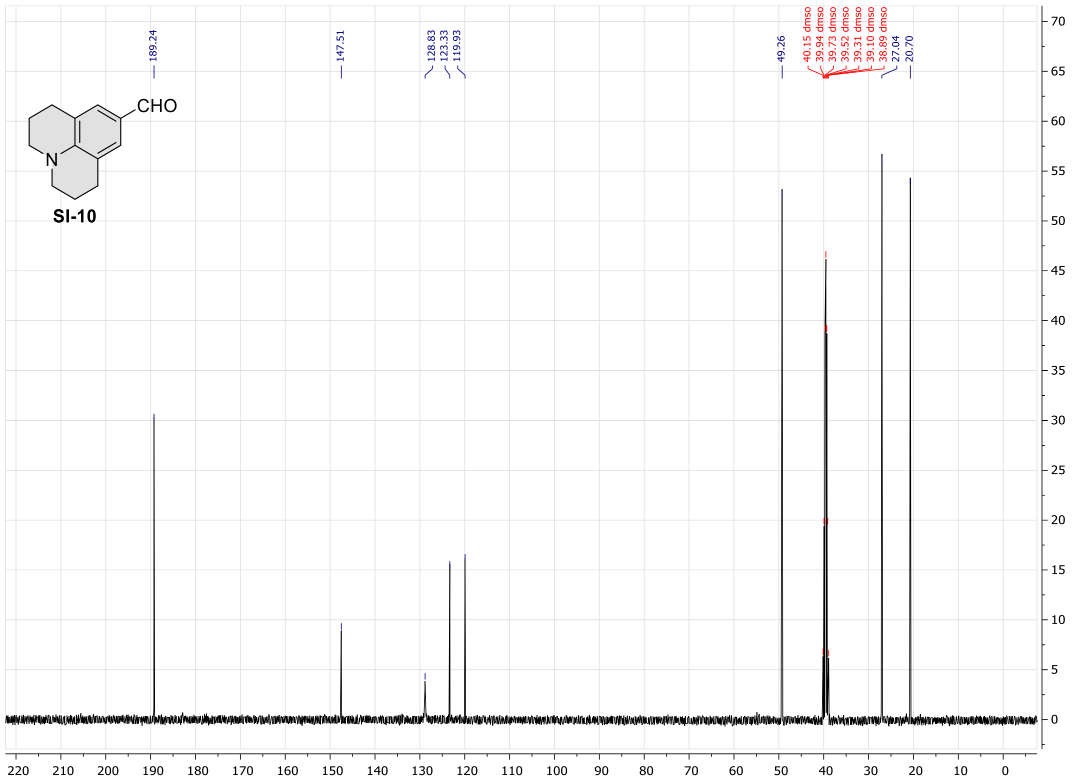

Supplementary Figure 126. ^{13}C NMR of compound **SI-10** (100 MHz, d_6-DMSO).

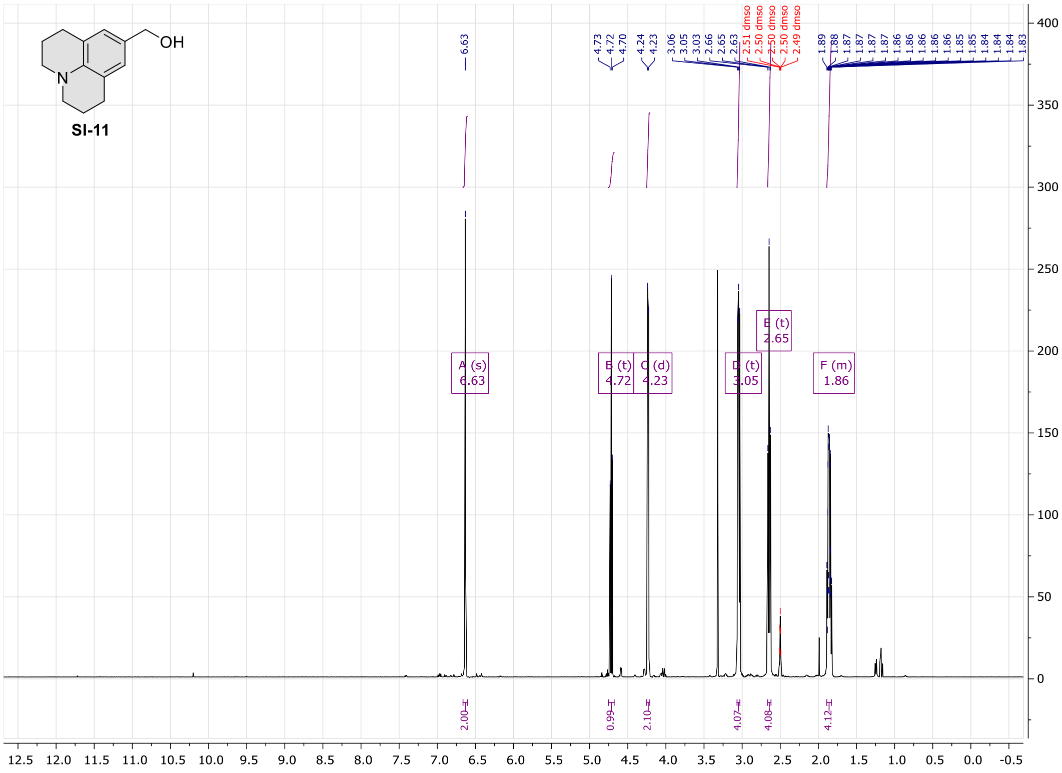

Supplementary Figure 127. ^{1}H NMR of compound **SI-11** (400 MHz, d_6-DMSO).

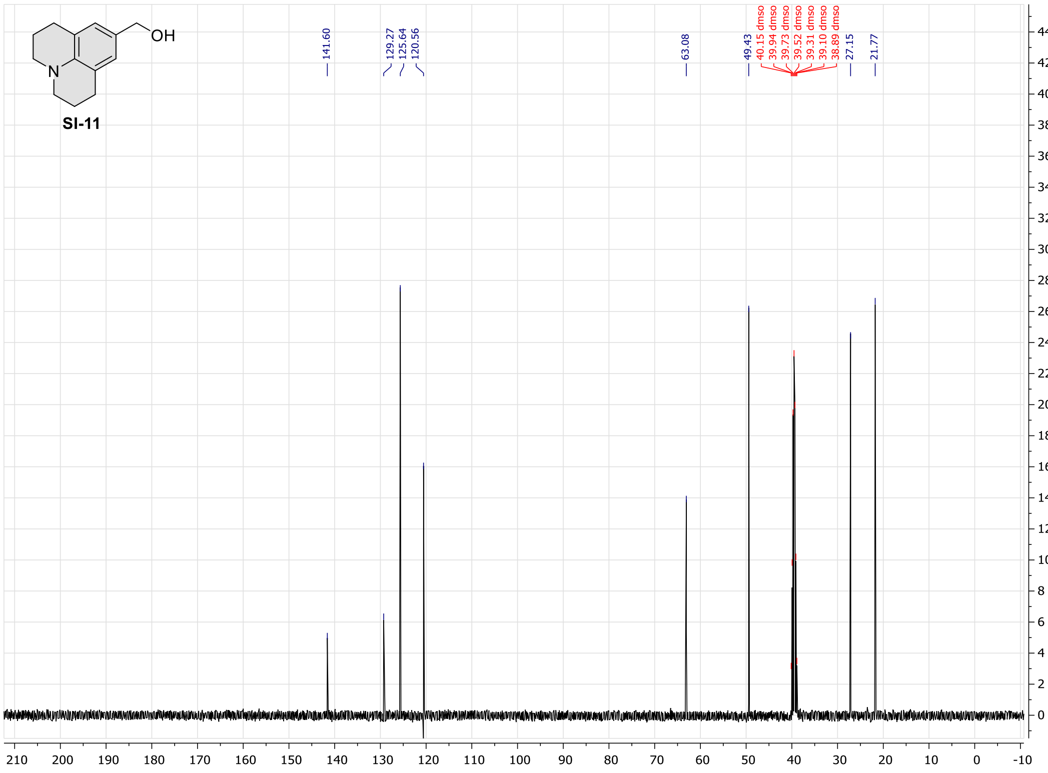

Supplementary Figure 128. ^{13}C NMR of compound **SI-11** (100 MHz, d_6-DMSO).

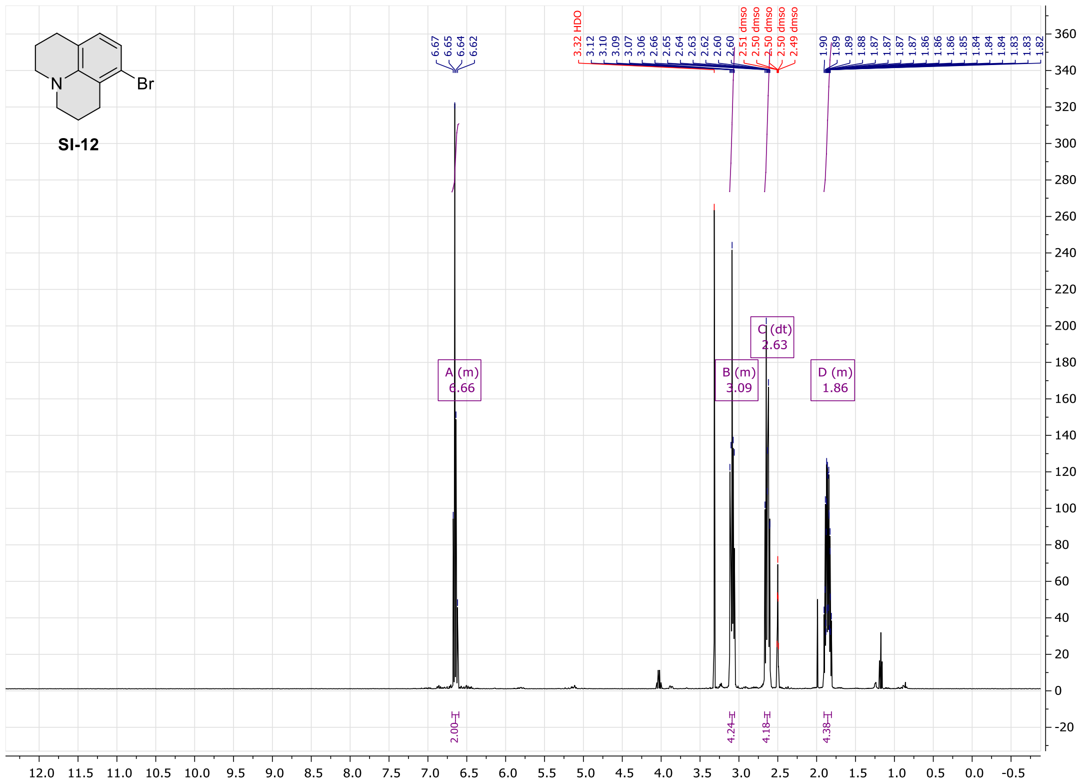

Supplementary Figure 129. ^{1}H NMR of compound **SI-12** (400 MHz, d_6-DMSO).

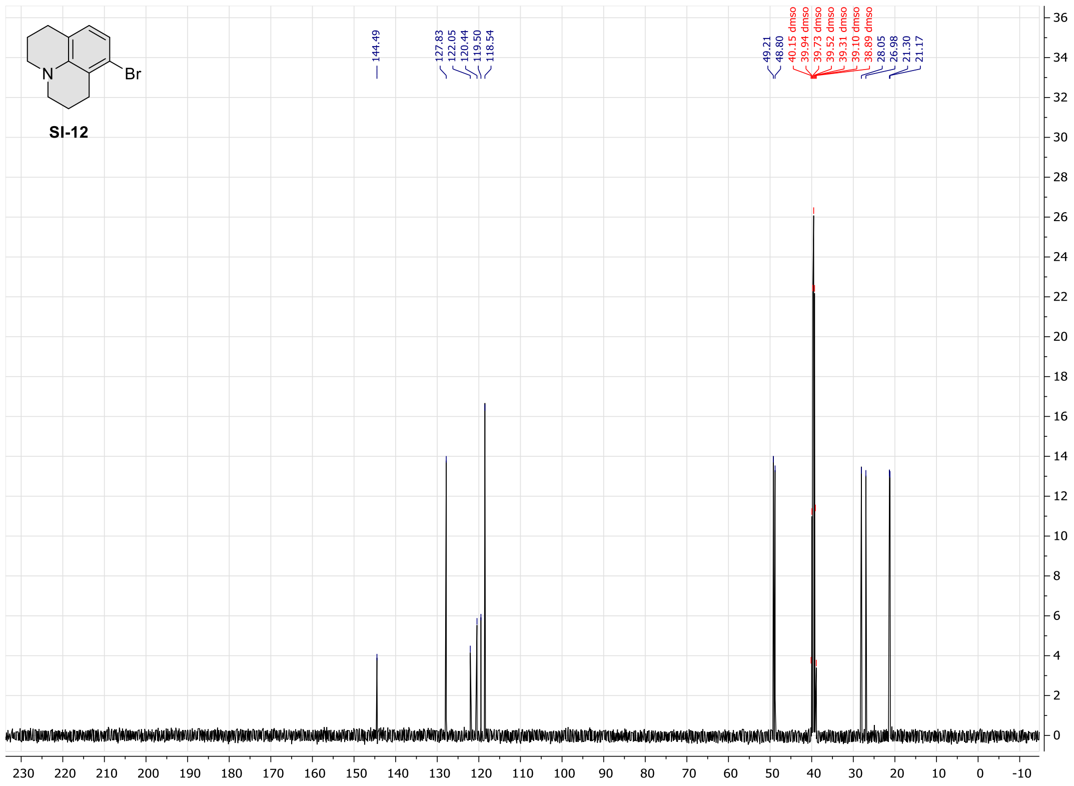

Supplementary Figure 130. ^{13}C NMR of compound **SI-12** (100 MHz, d_6-DMSO).

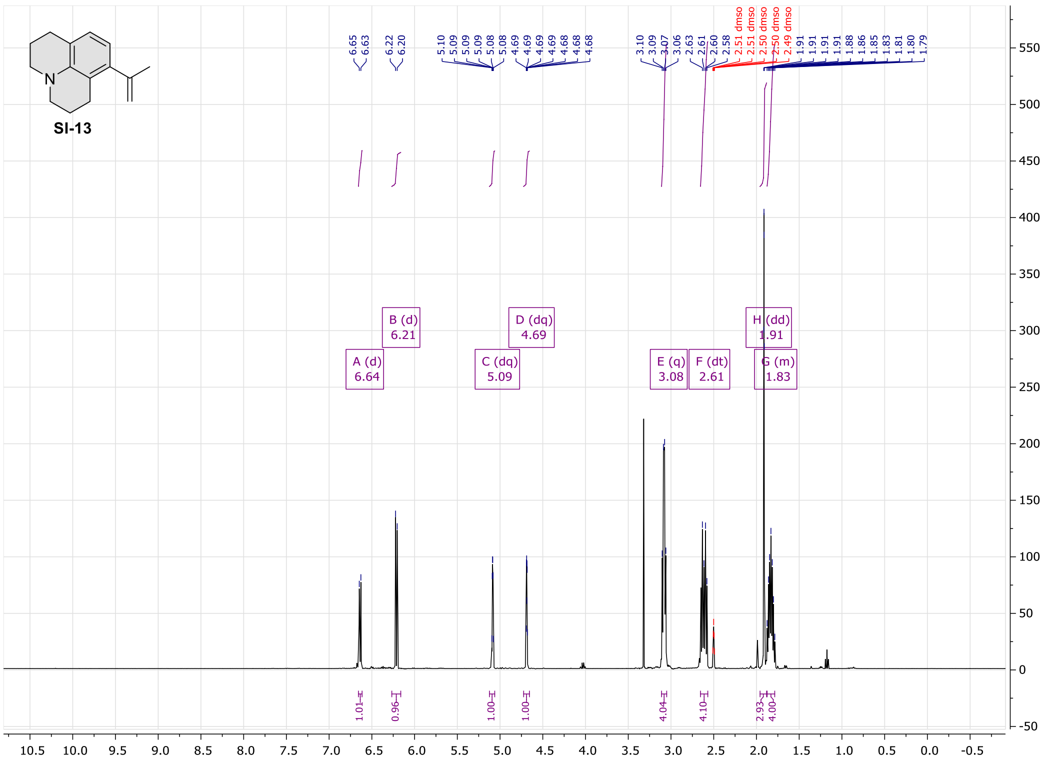

Supplementary Figure 131. ^{1}H NMR of compound **SI-13** (400 MHz, d_6-DMSO).

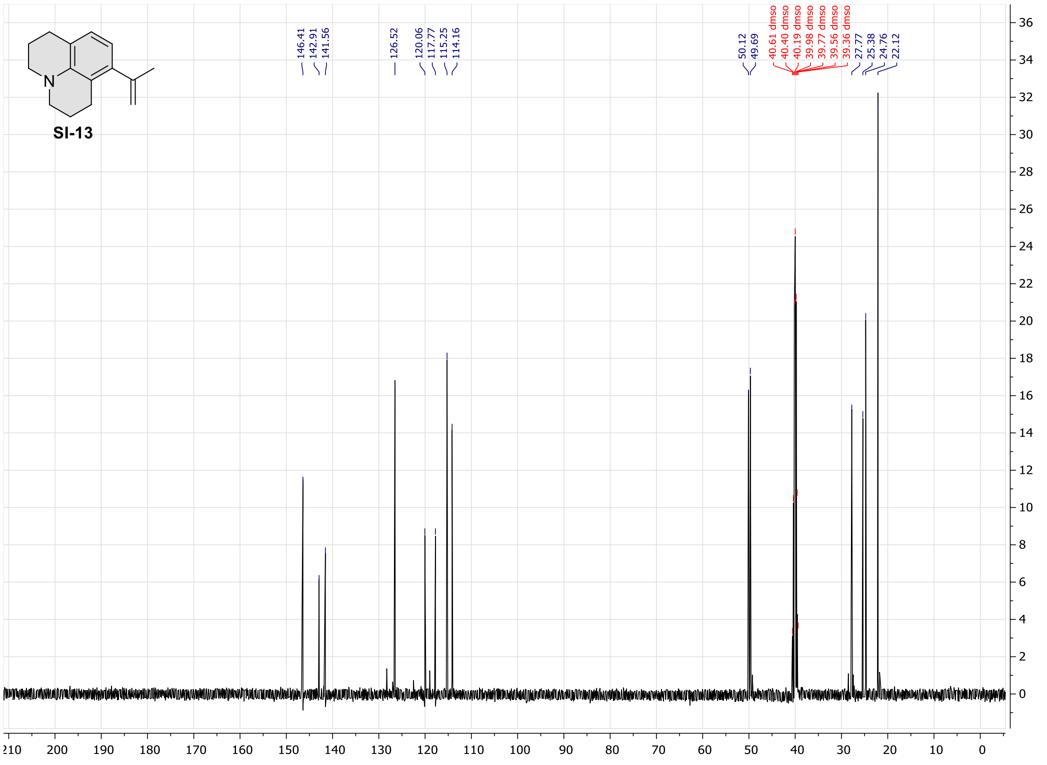

Supplementary Figure 132. ^{13}C NMR of compound **SI-13** (100 MHz, d_6-DMSO).

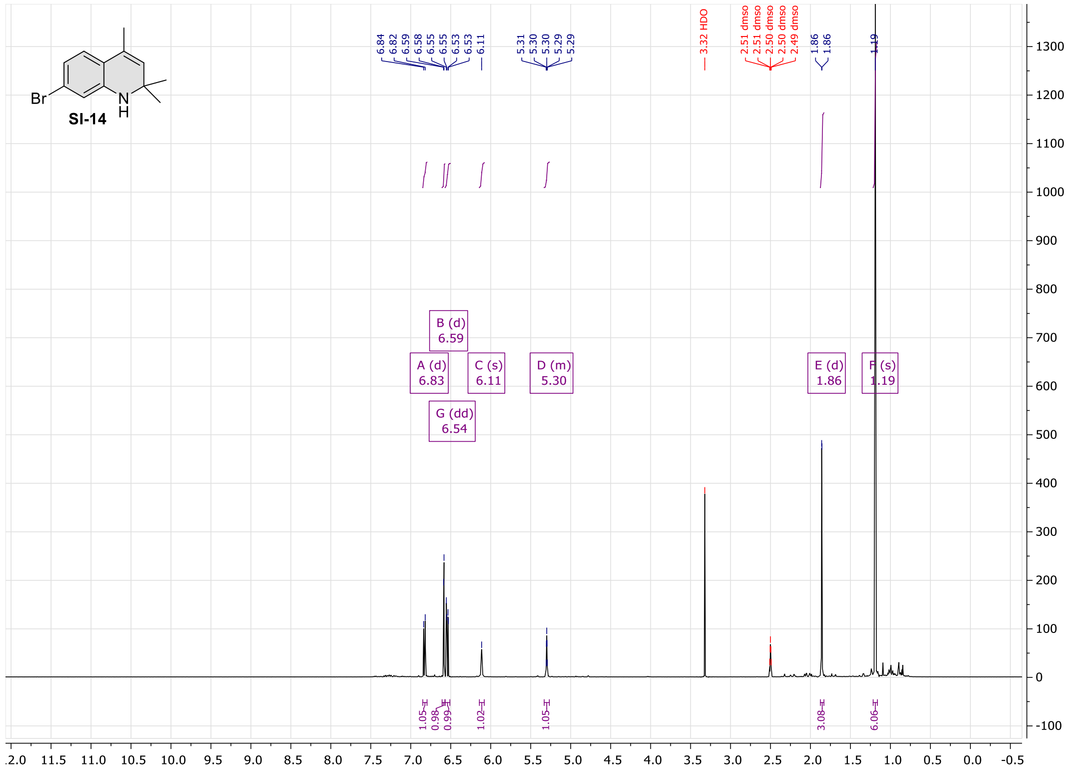

Supplementary Figure 133. ¹H NMR of compound **SI-14** (400 MHz, d_6-DMSO)**.**

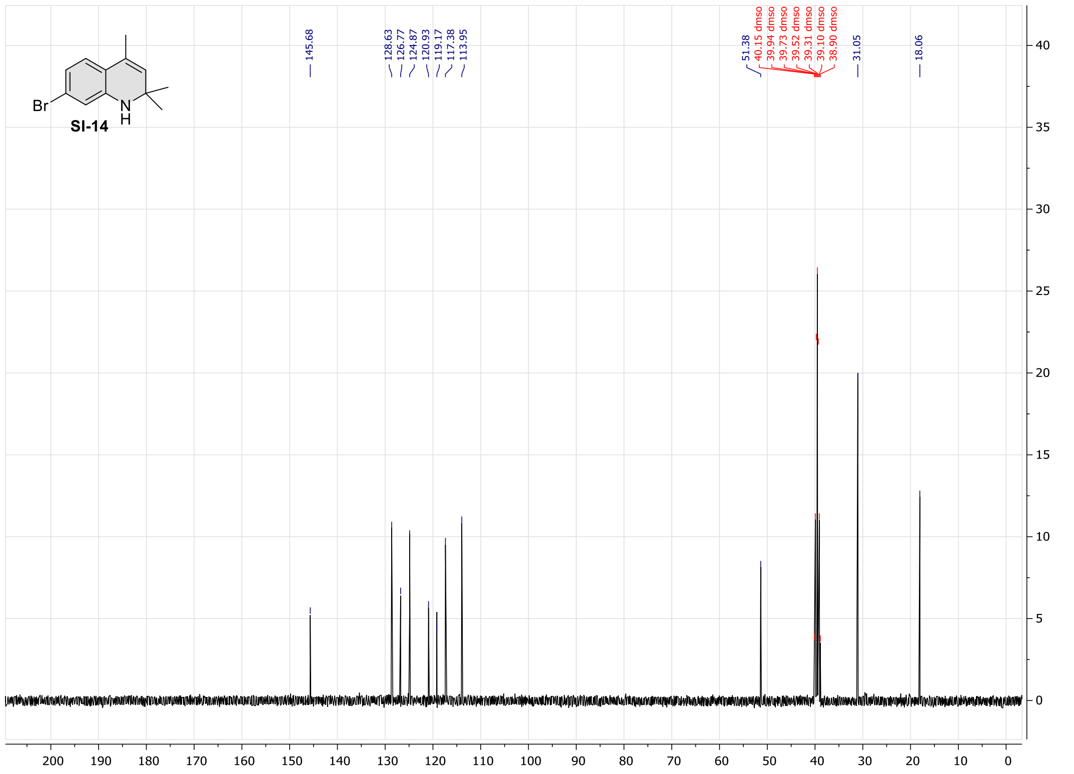

Supplementary Figure 134. ¹³C NMR of compound **SI-14** (100 MHz, d_6-DMSO).

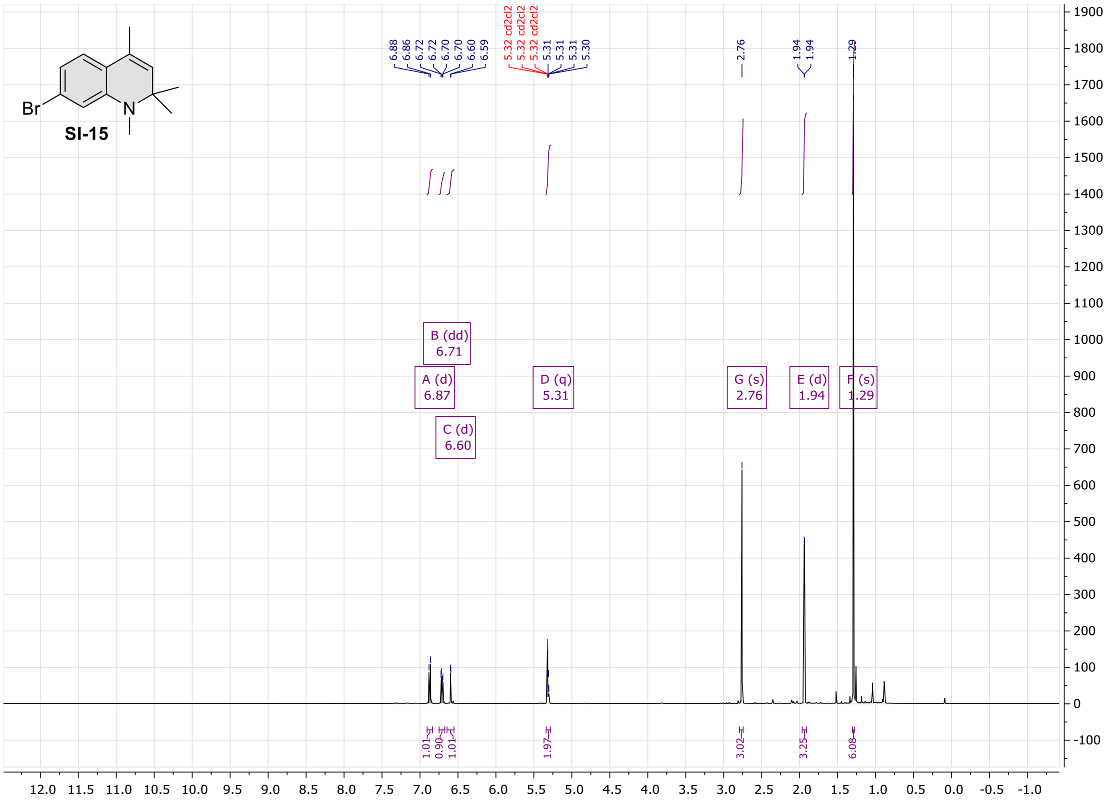

Supplementary Figure 135. ^{1}H NMR of compound **SI-15** (400 MHz, CD_2Cl_2).

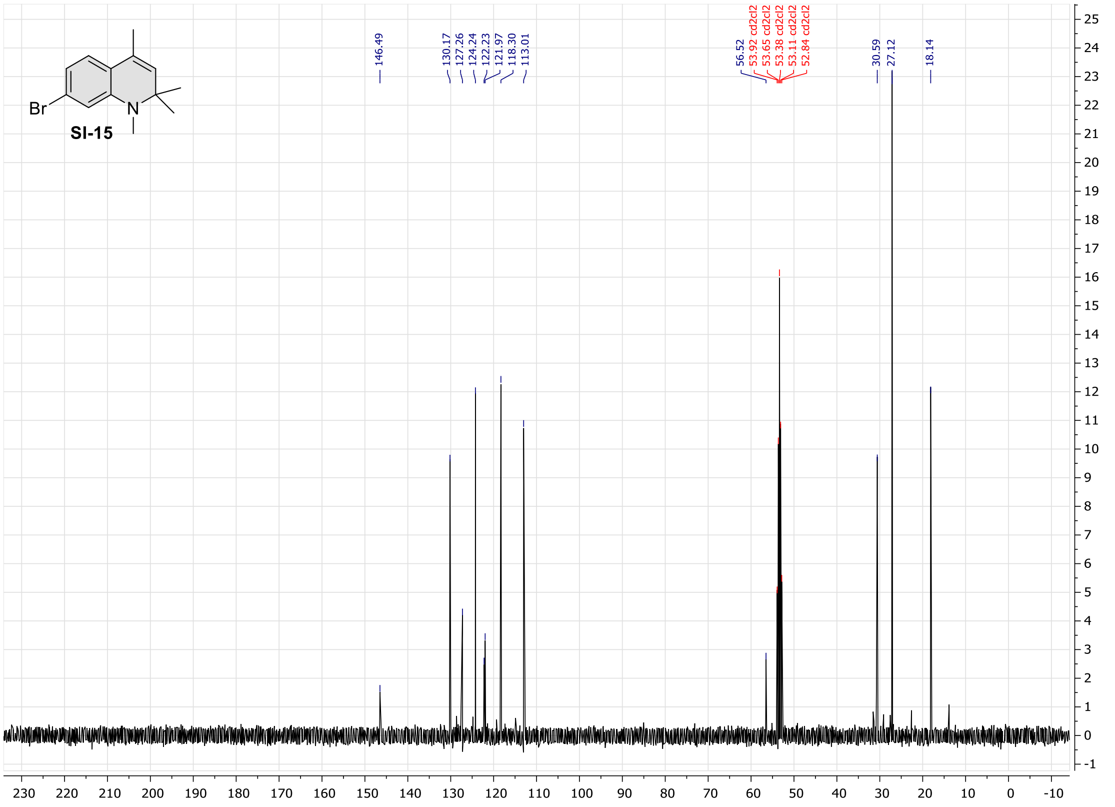

Supplementary Figure 136. ^{13}C NMR of compound **SI-15** (100 MHz, CD_2Cl_2).

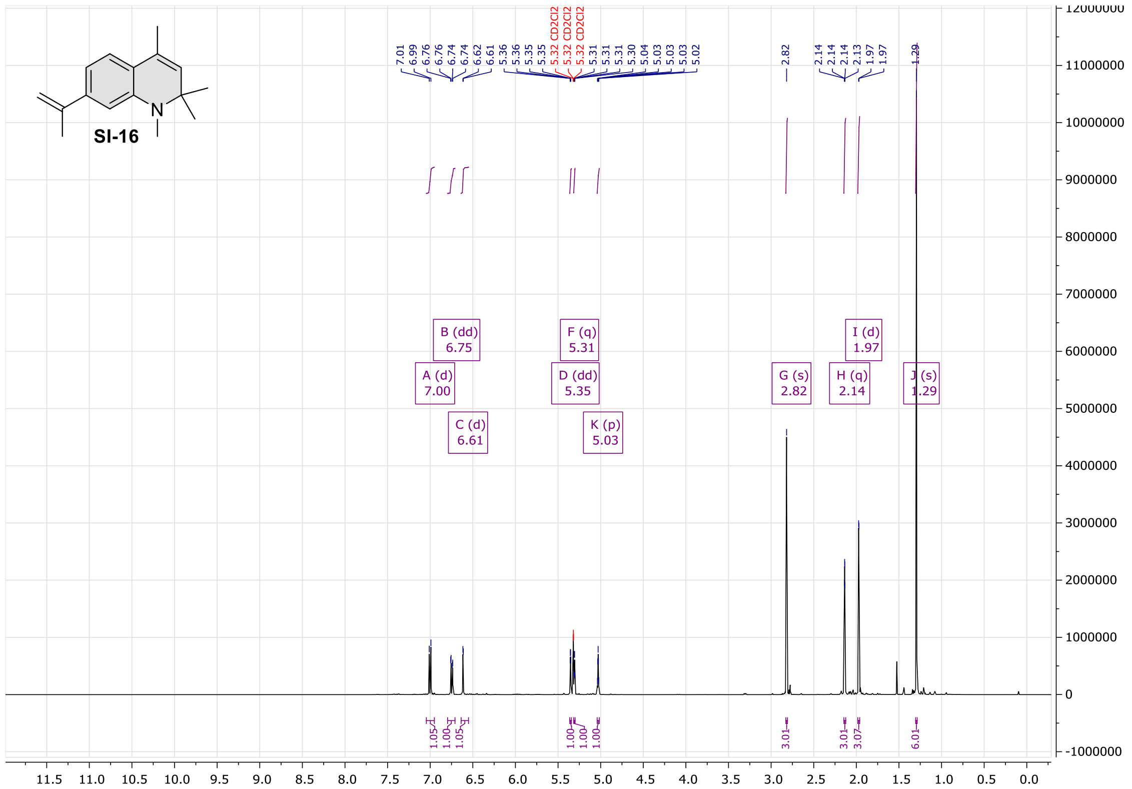

Supplementary Figure 137. ^{1}H NMR of compound **SI-16** (400 MHz, CD_2Cl_2).

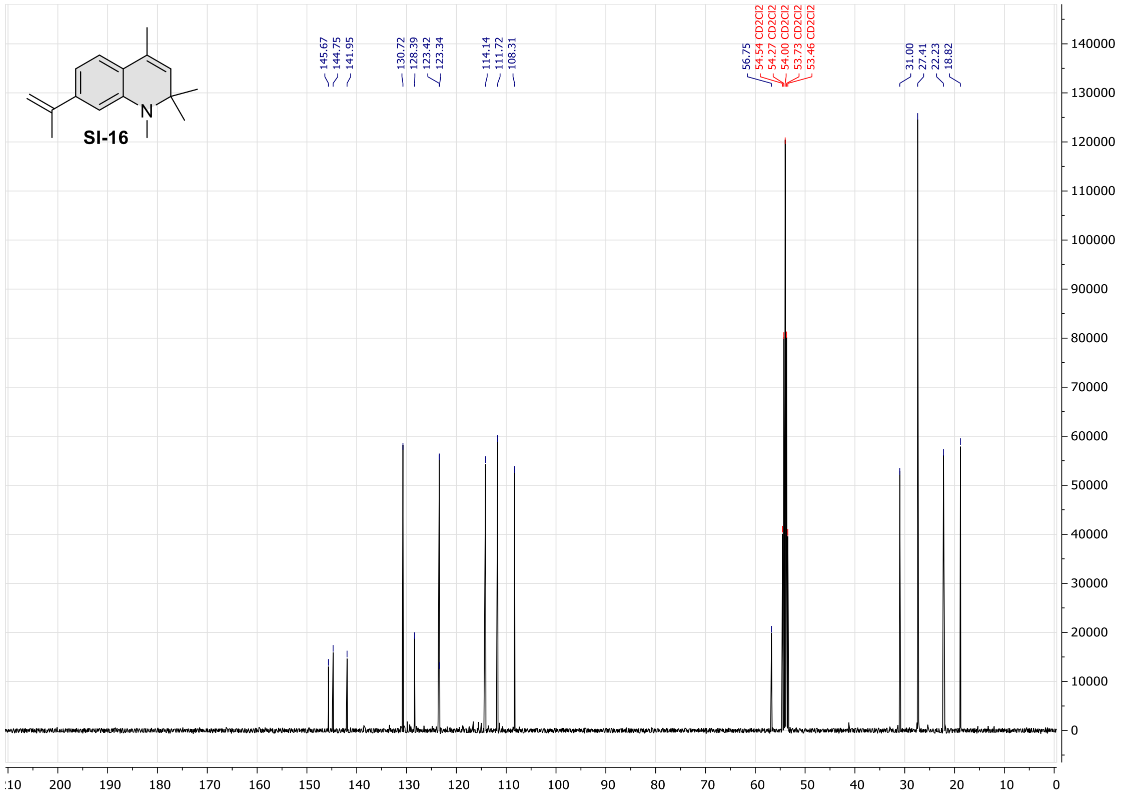

Supplementary Figure 138. ^{13}C NMR of compound **SI-16** (100 MHz, CD_2Cl_2).

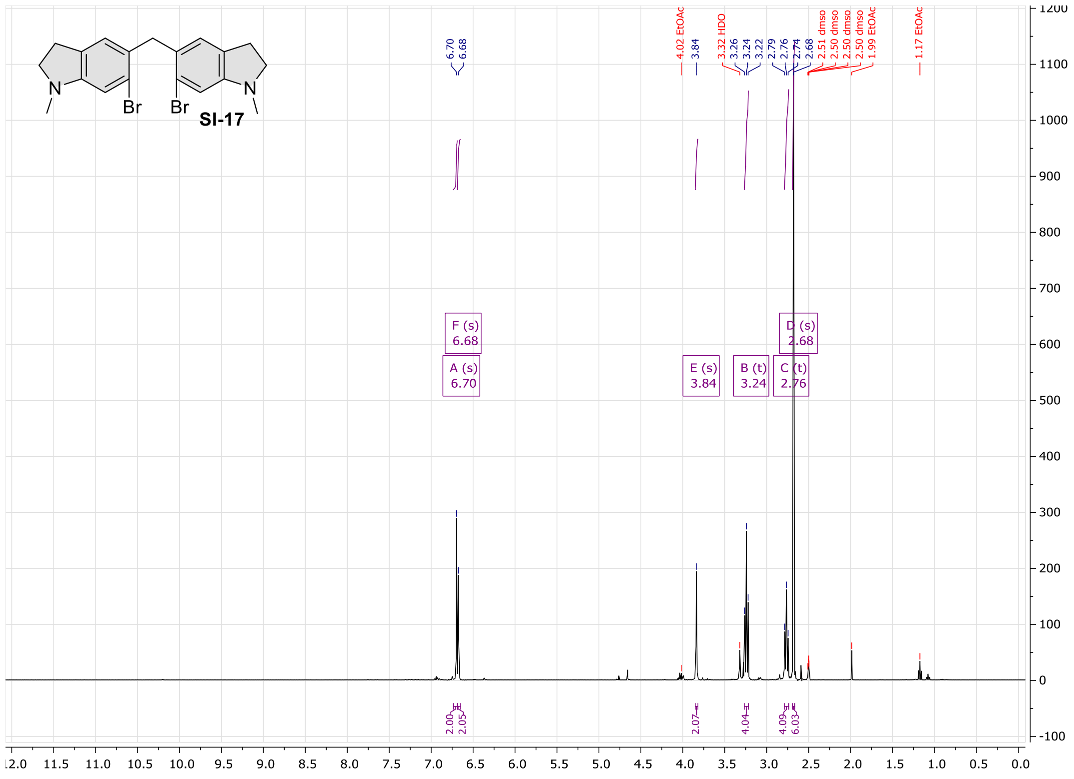

Supplementary Figure 139. ^{1}H NMR of compound **SI-17** (400 MHz, d_6-DMSO).

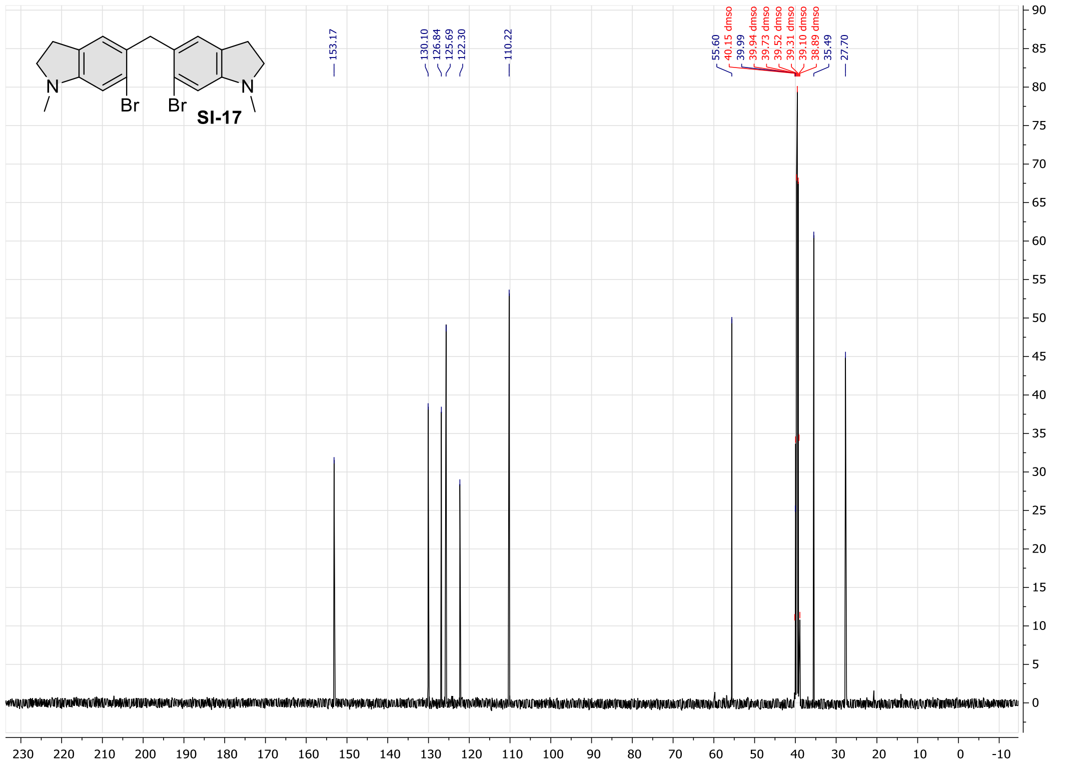

Supplementary Figure 140. ^{13}C NMR of compound **SI-17** (100 MHz, d_6-DMSO).

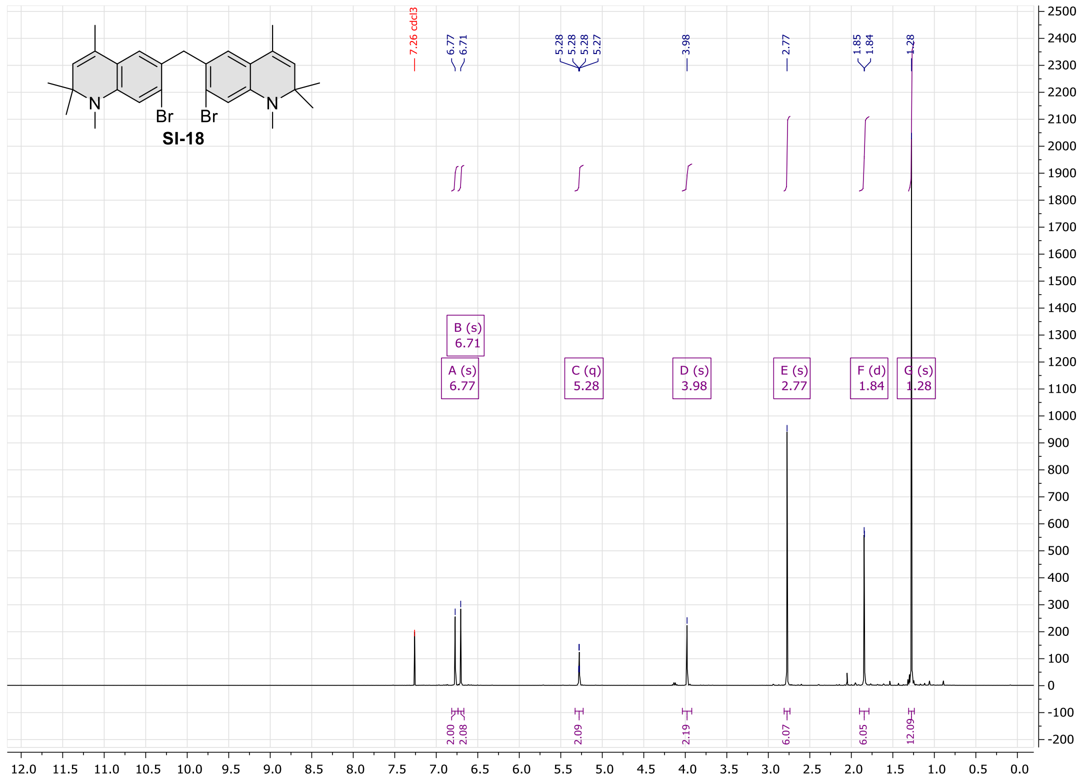

Supplementary Figure 141. ^{1}H NMR of compound **SI-18** (400 MHz, $CDCl_3$).

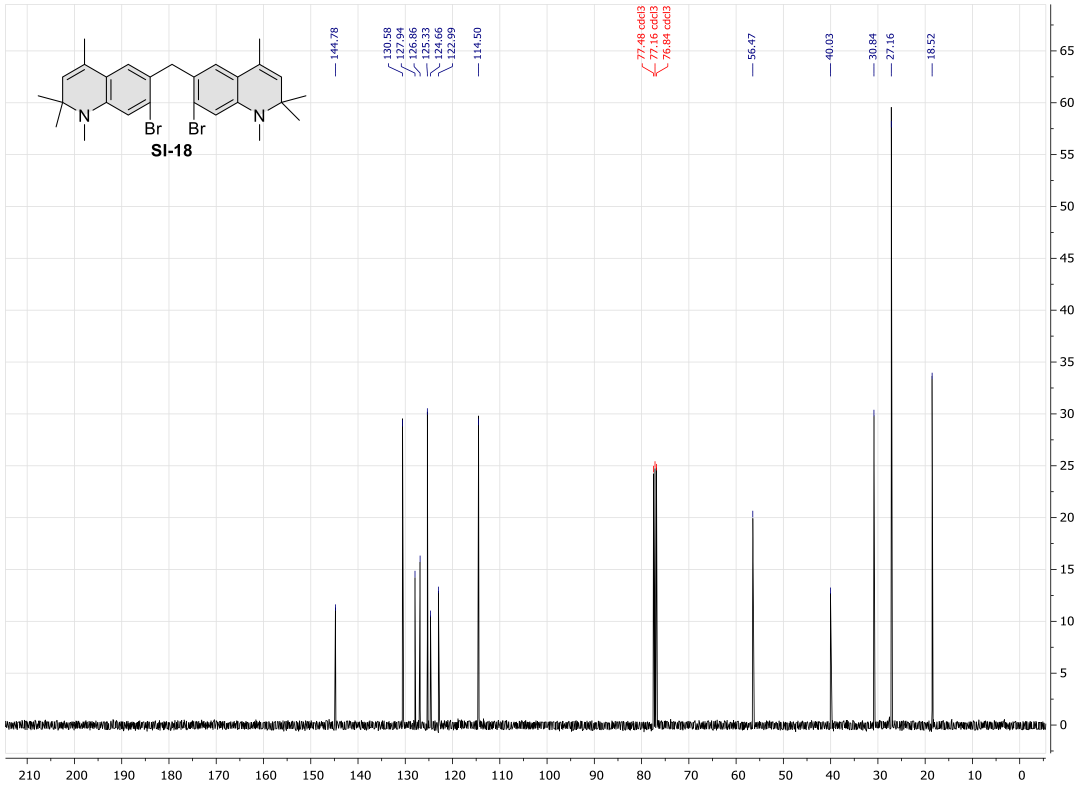

Supplementary Figure 142. ^{13}C NMR of compound **SI-18** (100 MHz, $CDCl_3$).

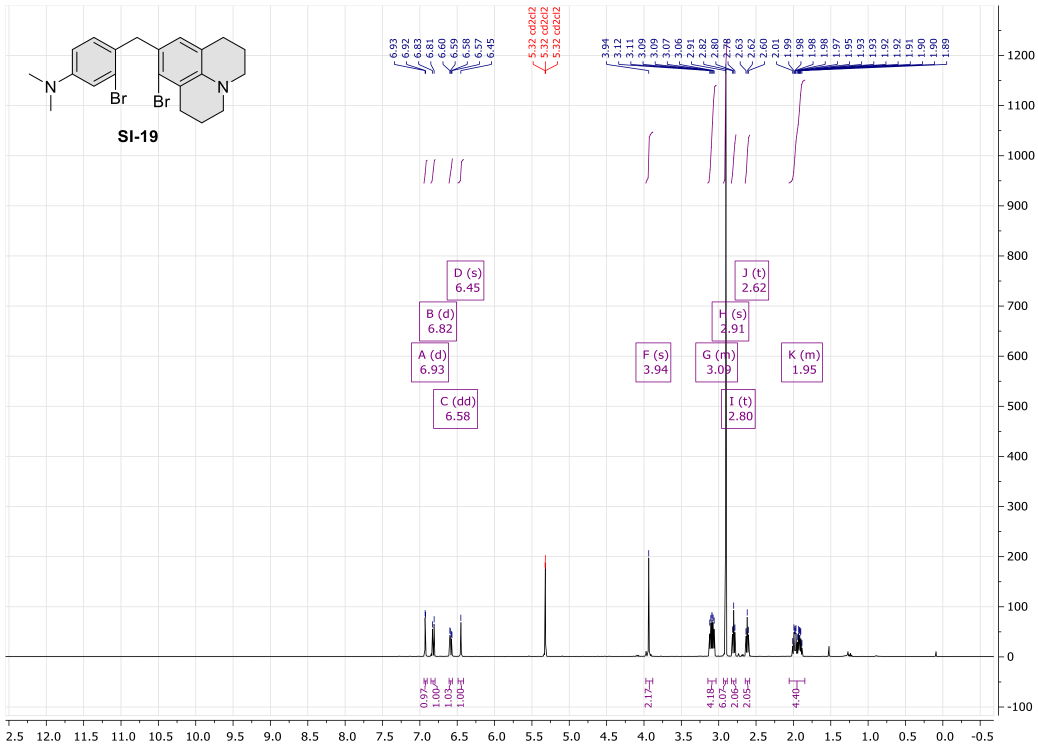

Supplementary Figure 143. ^{1}H NMR of compound **SI-19** (400 MHz, CD_2Cl_2).

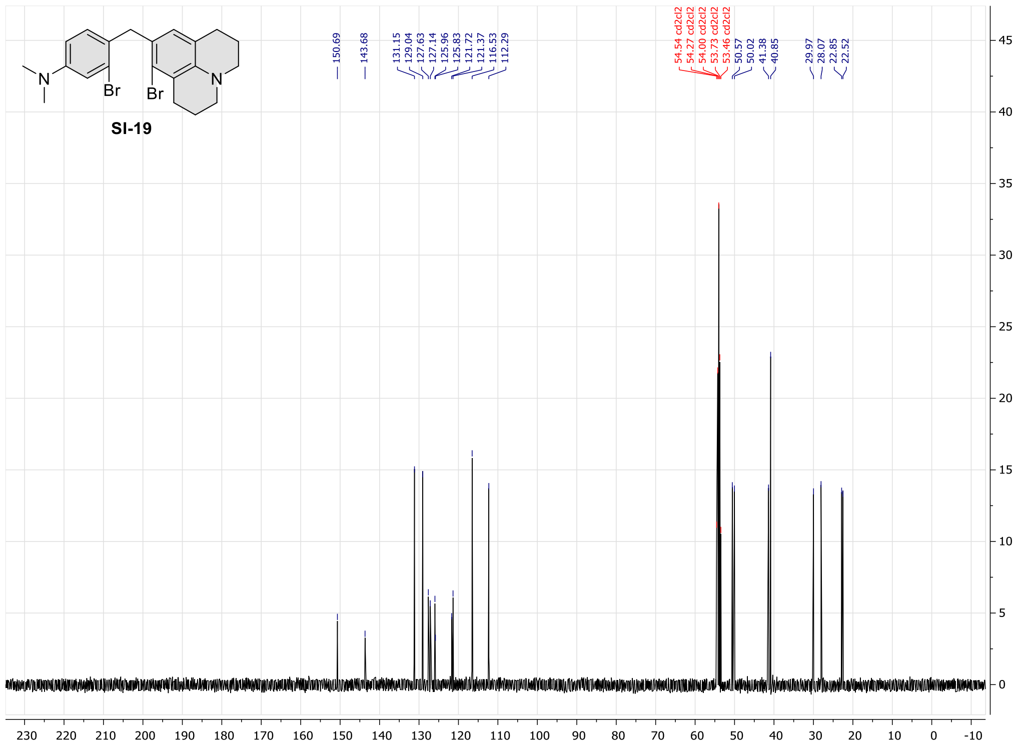

Supplementary Figure 144. ^{13}C NMR of compound **SI-19** (100 MHz, CD_2Cl_2).

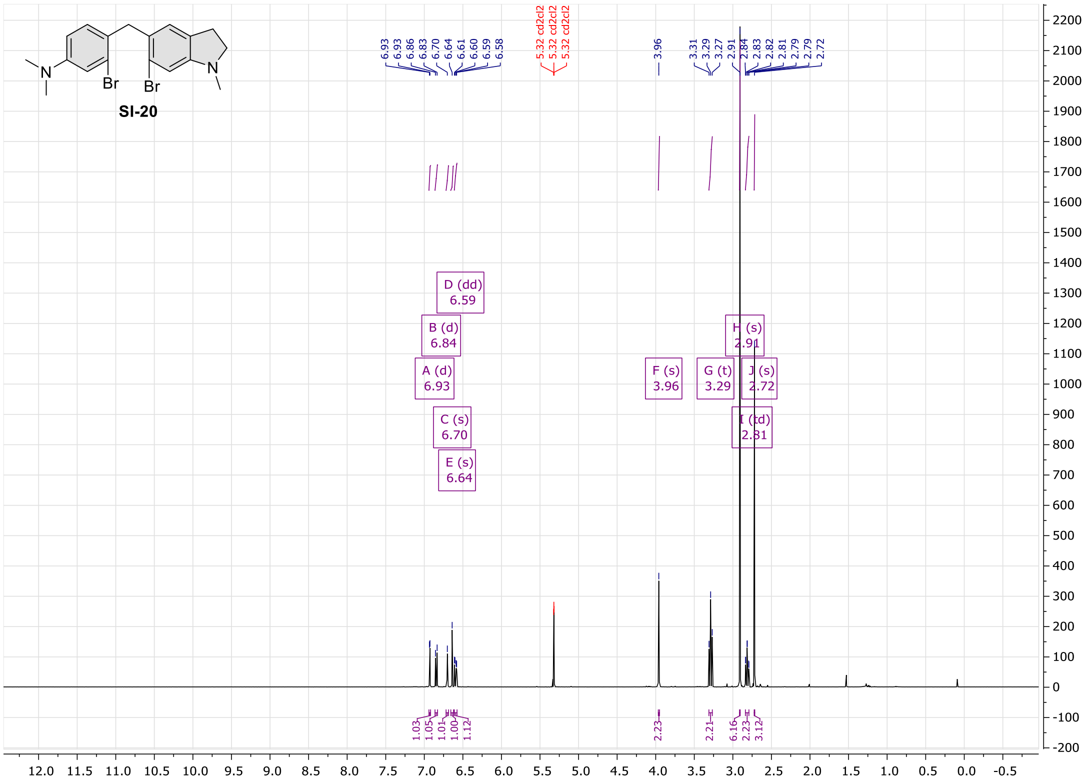

Supplementary Figure 145. ^{1}H NMR of compound **SI-20** (400 MHz, CD_2Cl_2).

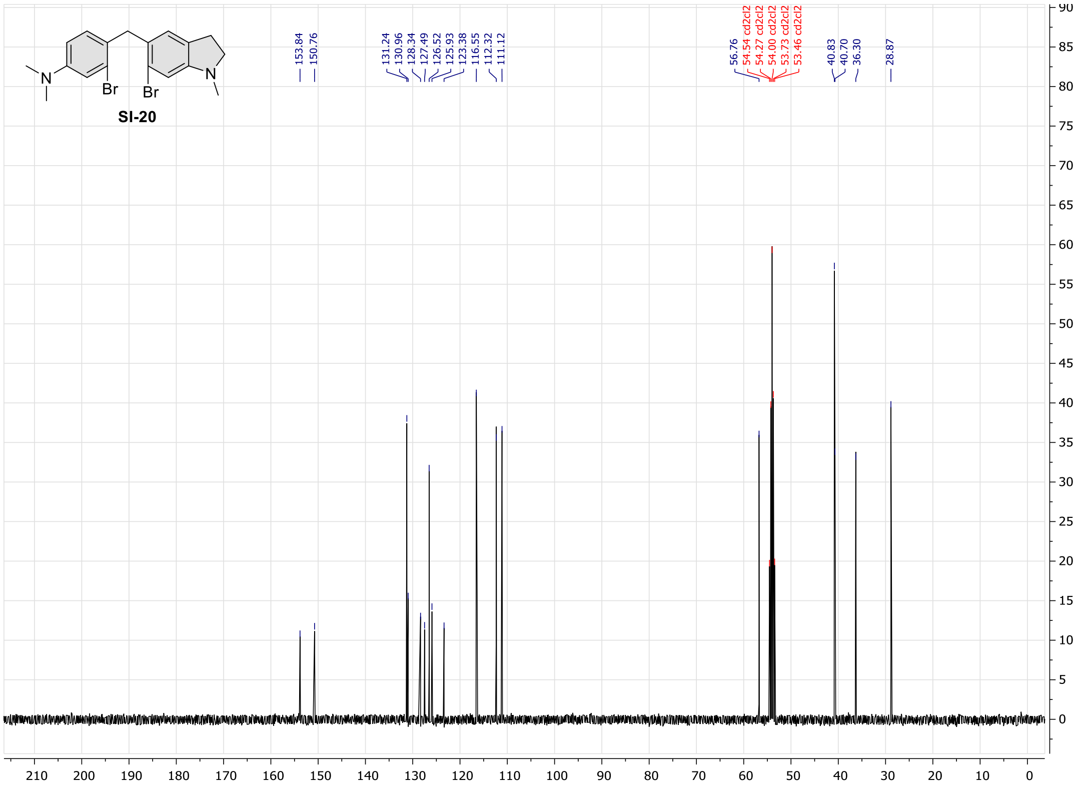

Supplementary Figure 146. ^{13}C NMR of compound **SI-20** (100 MHz, CD_2Cl_2).

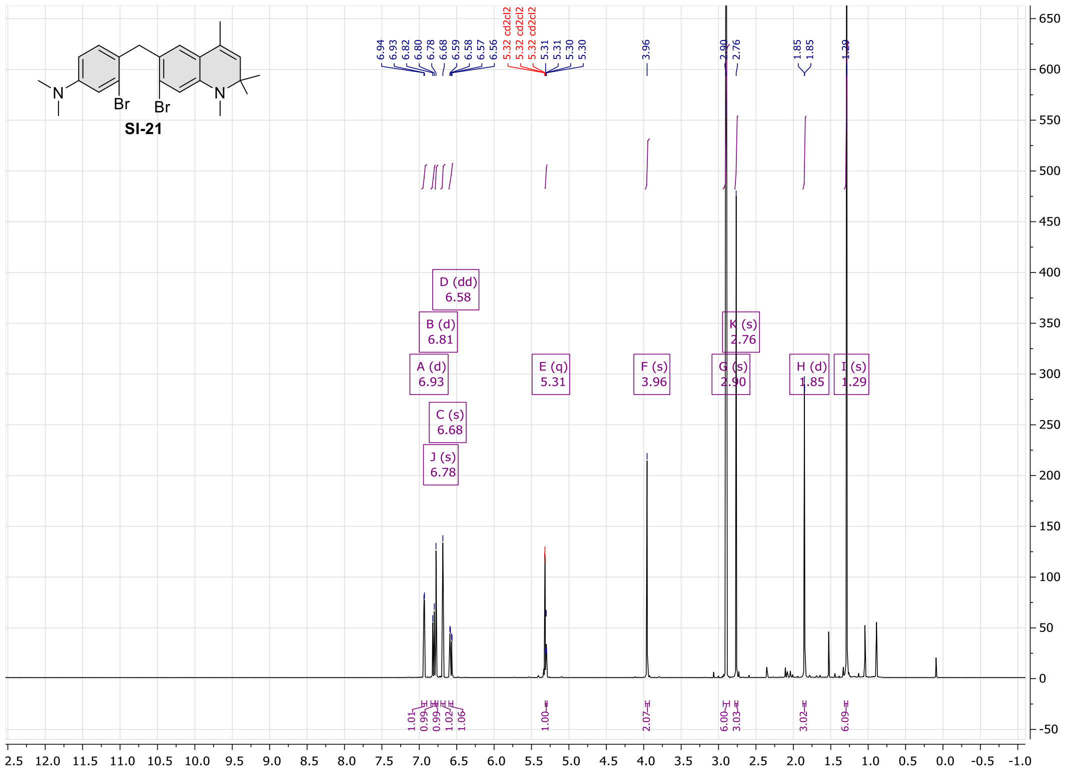

Supplementary Figure 147. ^{1}H NMR of compound **SI-21** (400 MHz, CD_2Cl_2).

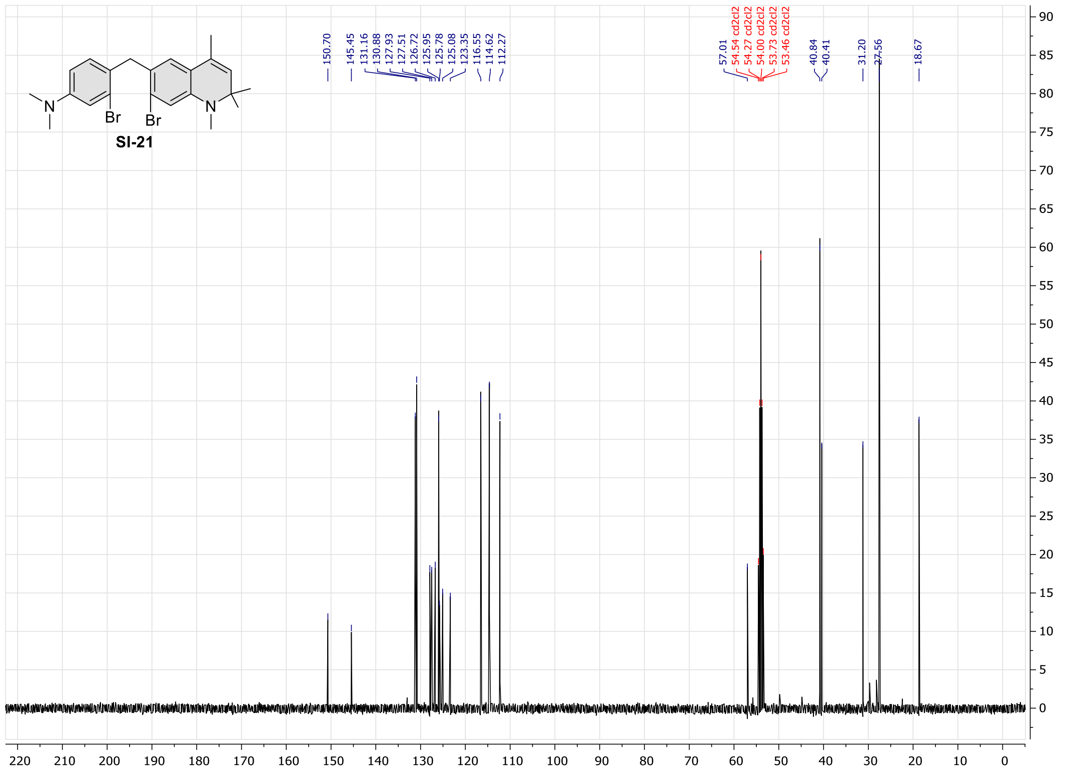

Supplementary Figure 148. ^{13}C NMR of compound **SI-21** (100 MHz, CD_2Cl_2).

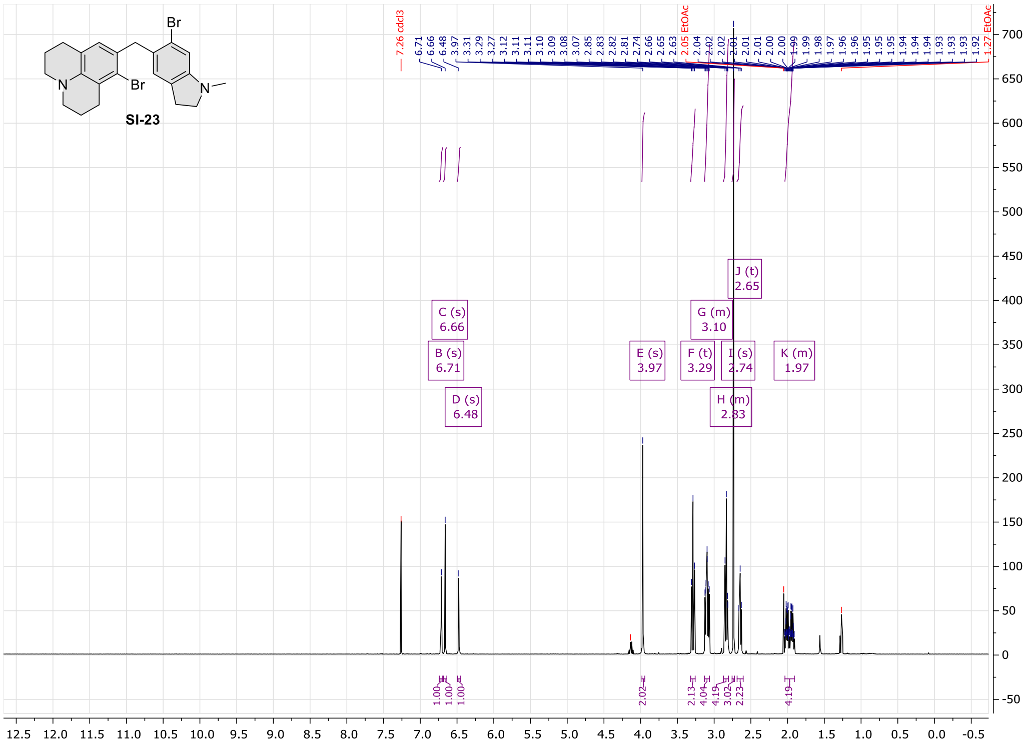

Supplementary Figure 149. ^{1}H NMR of compound **SI-23** (400 MHz, $CDCl_3$).

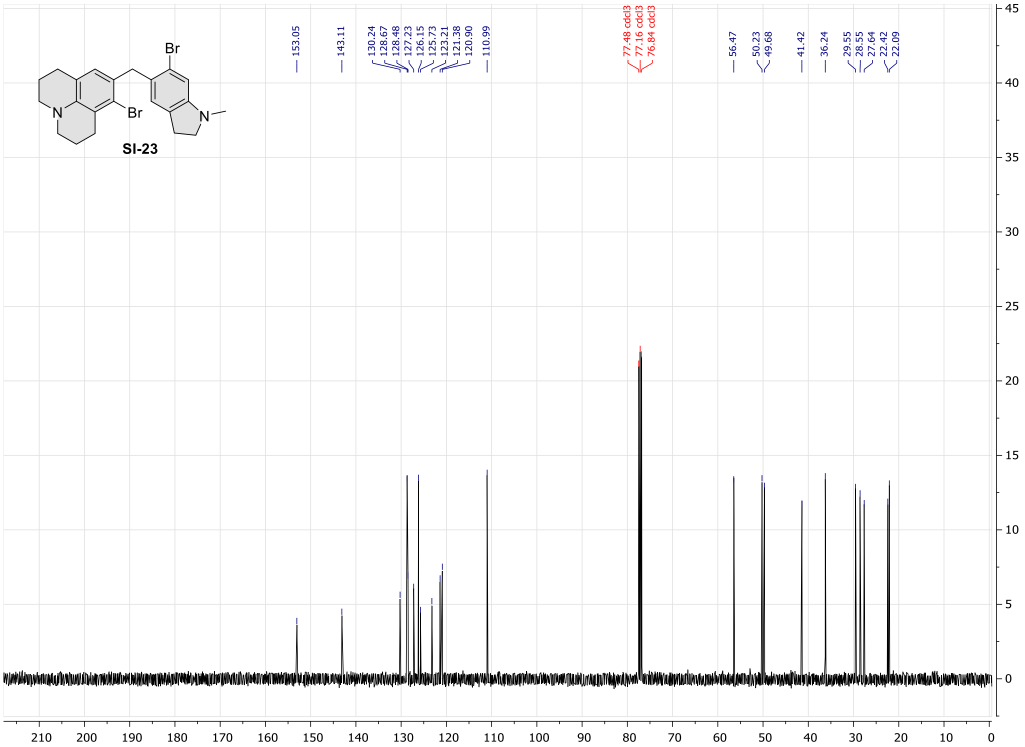

Supplementary Figure 150. ^{13}C NMR of compound **SI-23** (100 MHz, $CDCl_3$).

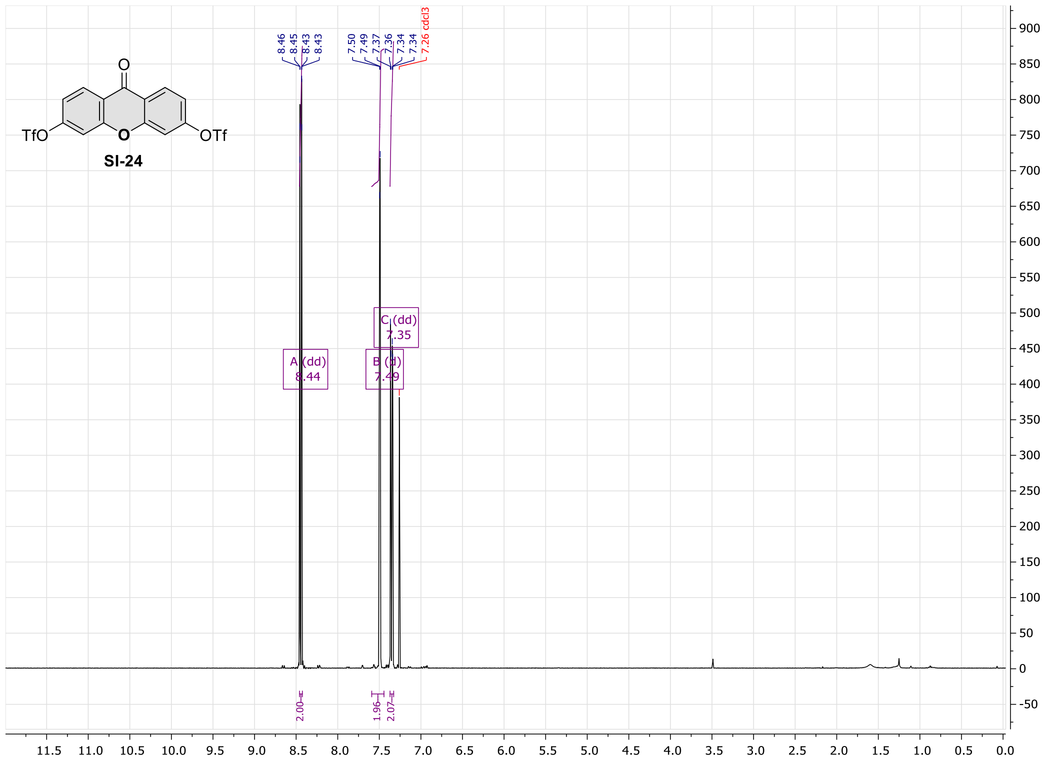

Supplementary Figure 151. ^{1}H NMR of compound **SI-24** (400 MHz, $CDCl_3$).

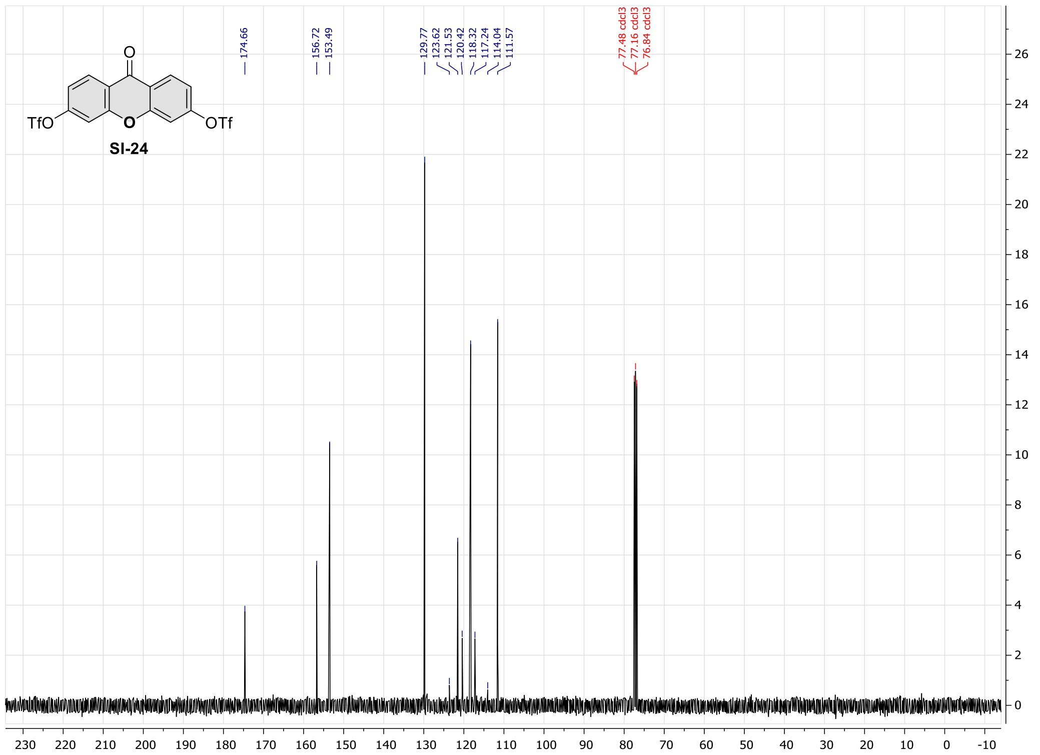

Supplementary Figure 152. ^{13}C NMR of compound **SI-24** (100 MHz, $CDCl_3$).

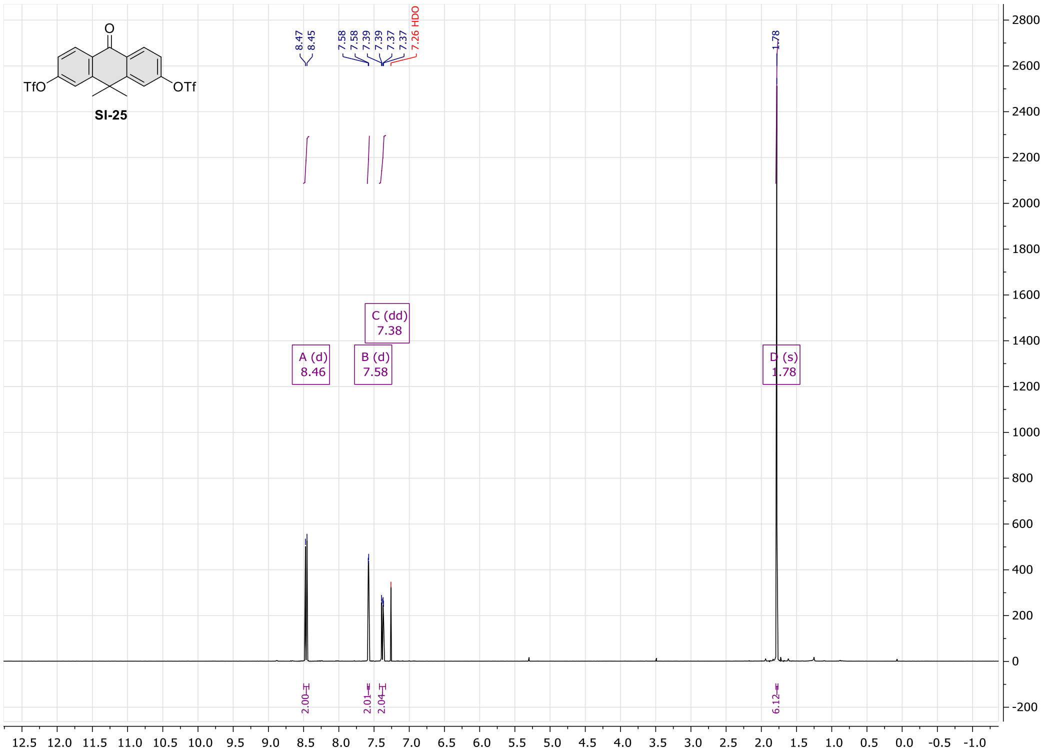

Supplementary Figure 153. ^{1}H NMR of compound **SI-25** (400 MHz, $CDCl_3$).

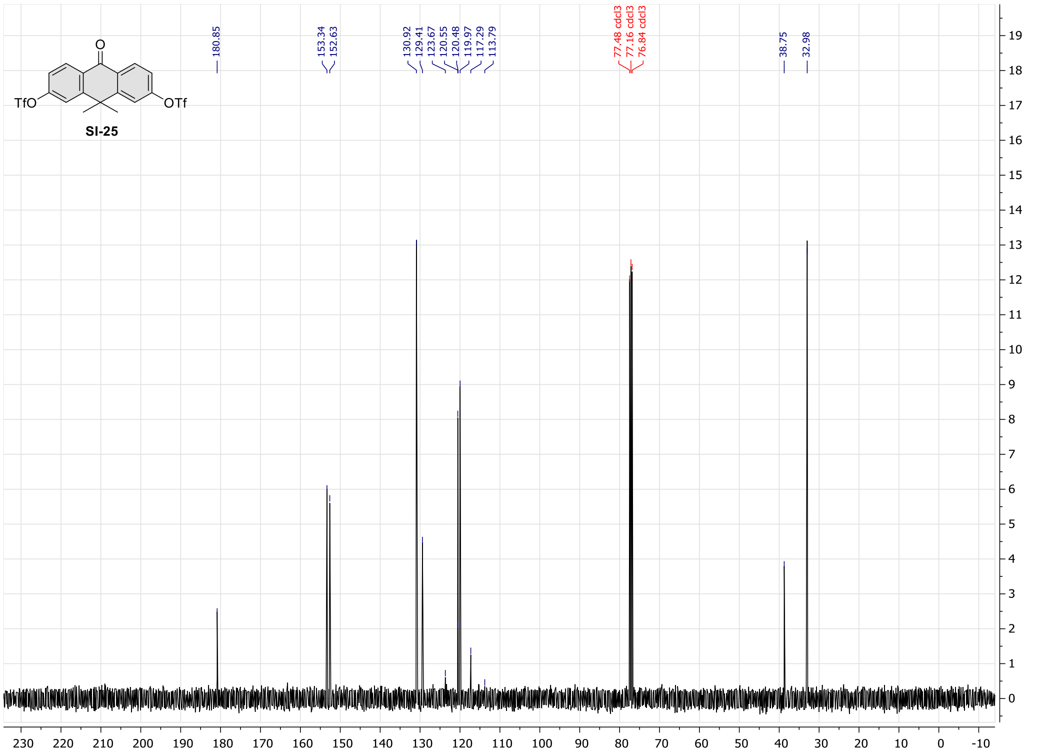

Supplementary Figure 154. ^{13}C NMR of compound **SI-25** (100 MHz, $CDCl_3$).

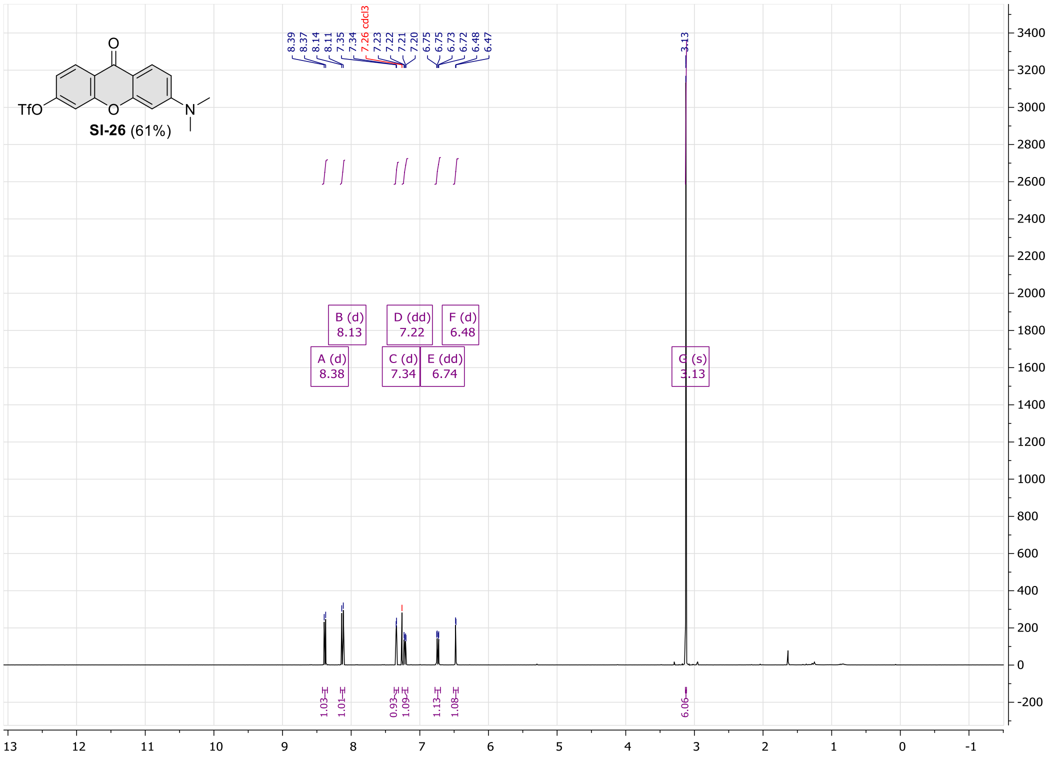

Supplementary Figure 155. ^{1}H NMR of compound **SI-26** (400 MHz, $CDCl_3$).

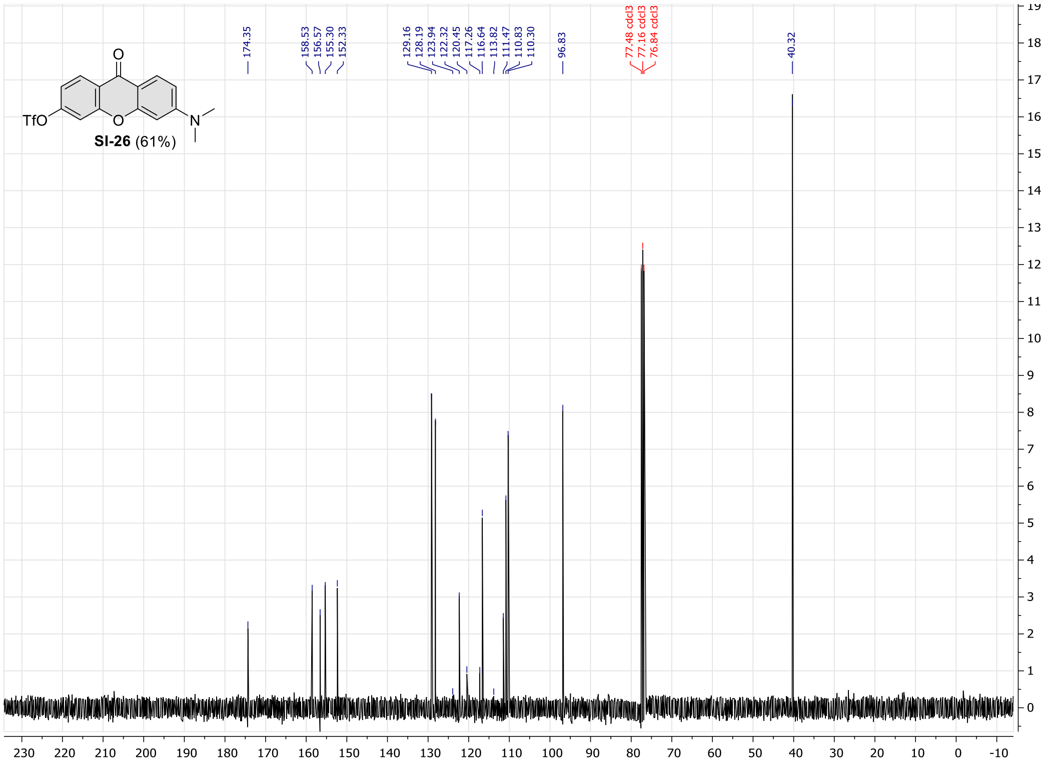

Supplementary Figure 156. ^{13}C NMR of compound **SI-26** (100 MHz, $CDCl_3$).

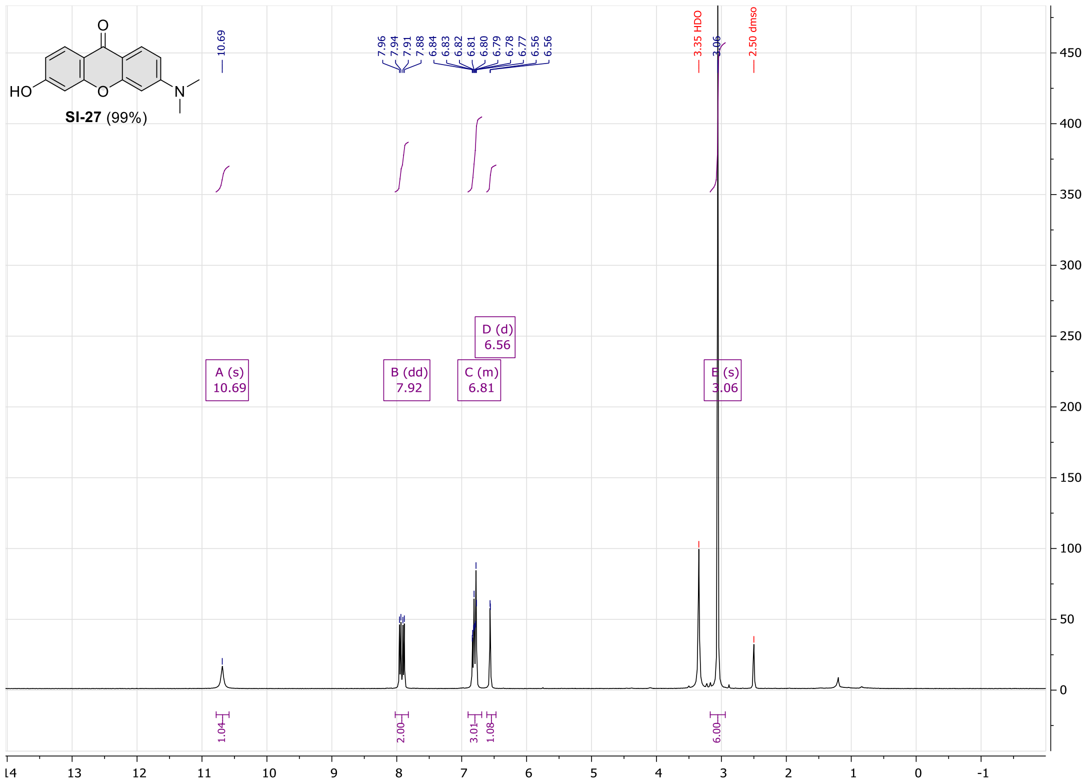

Supplementary Figure 157. ^{1}H NMR of compound **SI-27** (400 MHz, d_6-DMSO).

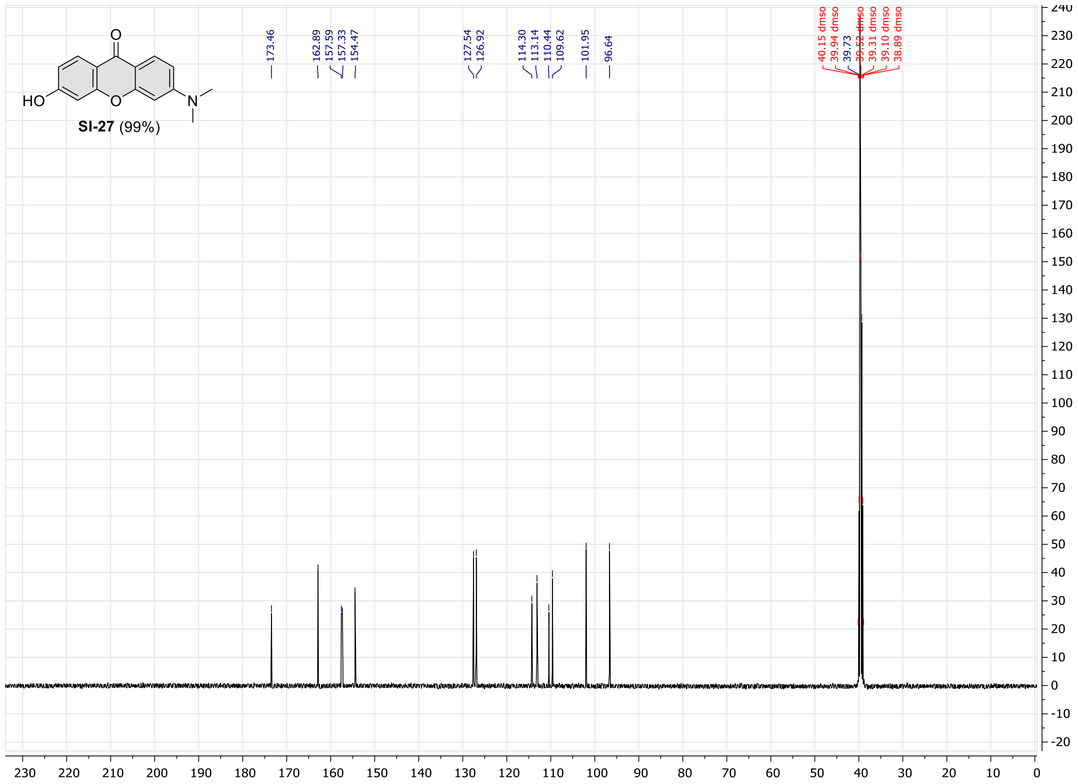

Supplementary Figure 158. ^{13}C NMR of compound **SI-27** (100 MHz, d_6-DMSO).

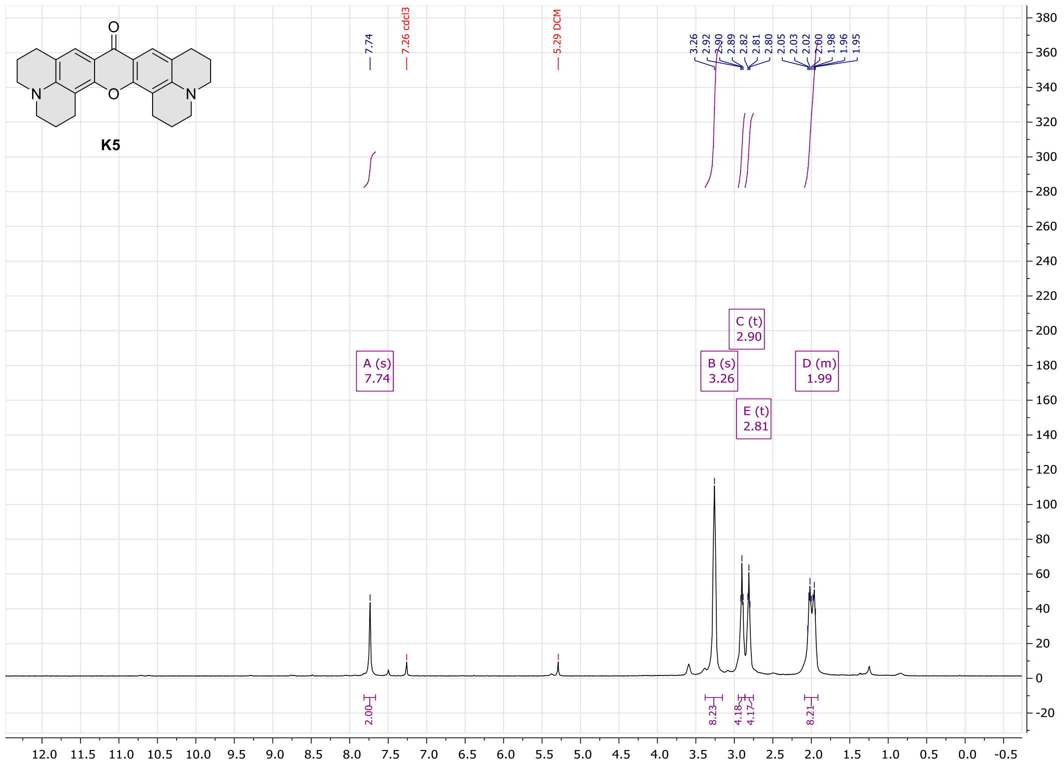

Supplementary Figure 159. ^{1}H NMR of compound **K5** (400 MHz, $CDCl_3$).

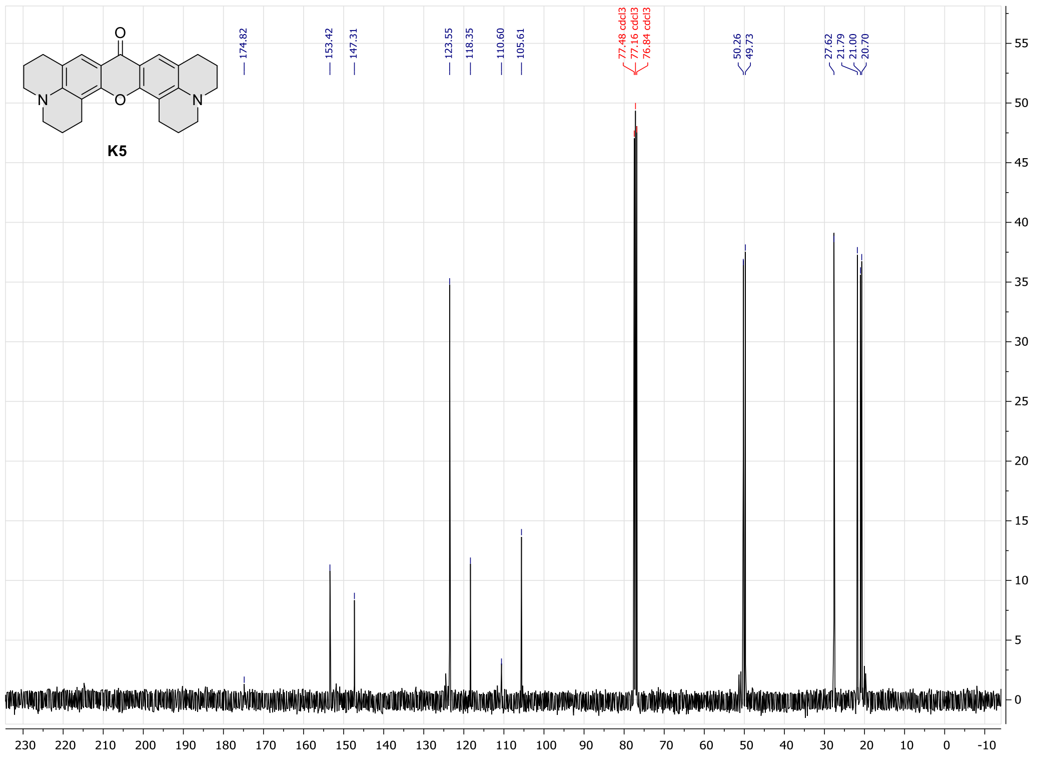

Supplementary Figure 160. ^{13}C NMR of compound **K5** (100 MHz, $CDCl_3$).

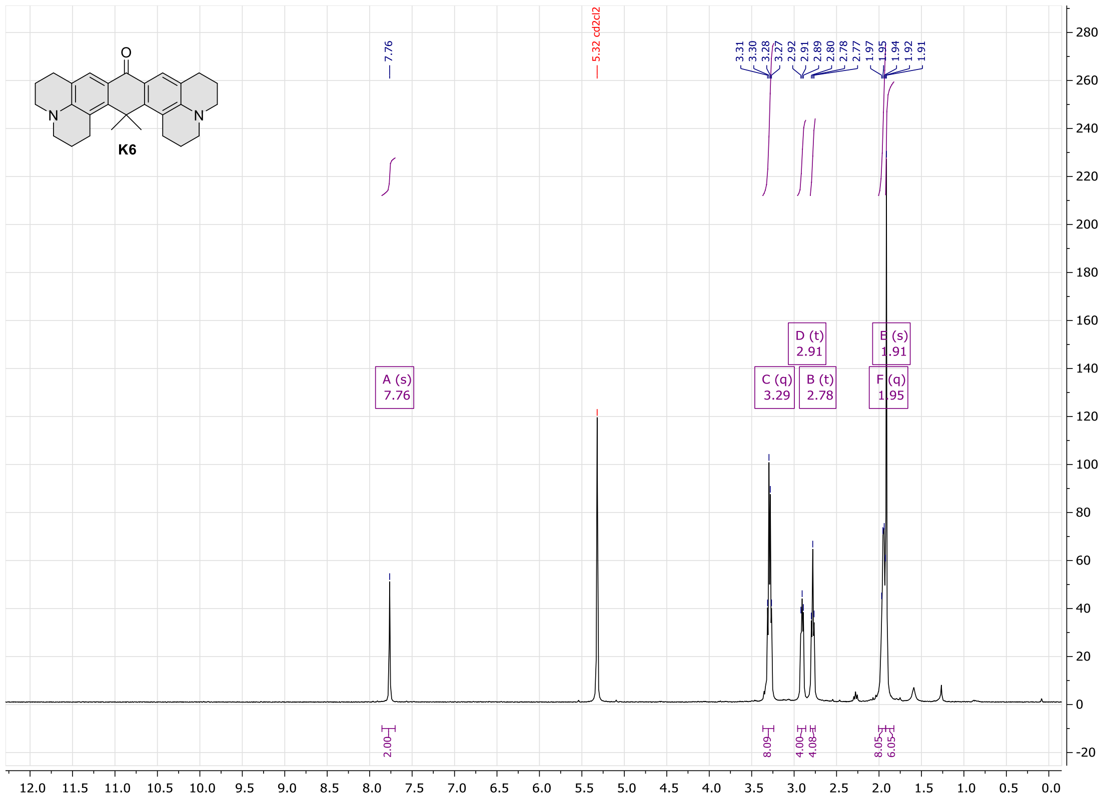

Supplementary Figure 161. ^{1}H NMR of compound **K6** (400 MHz, CD_2Cl_2).

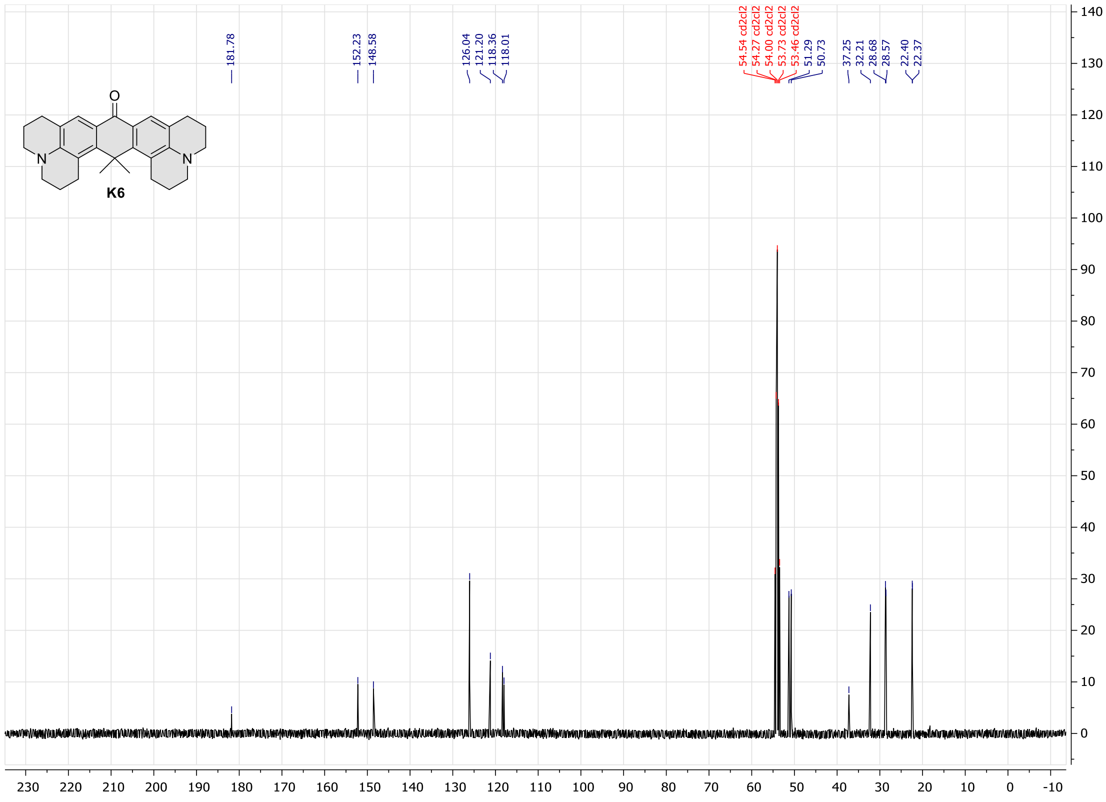

Supplementary Figure 162. ^{13}C NMR of compound **K6** (100 MHz, CD_2Cl_2).

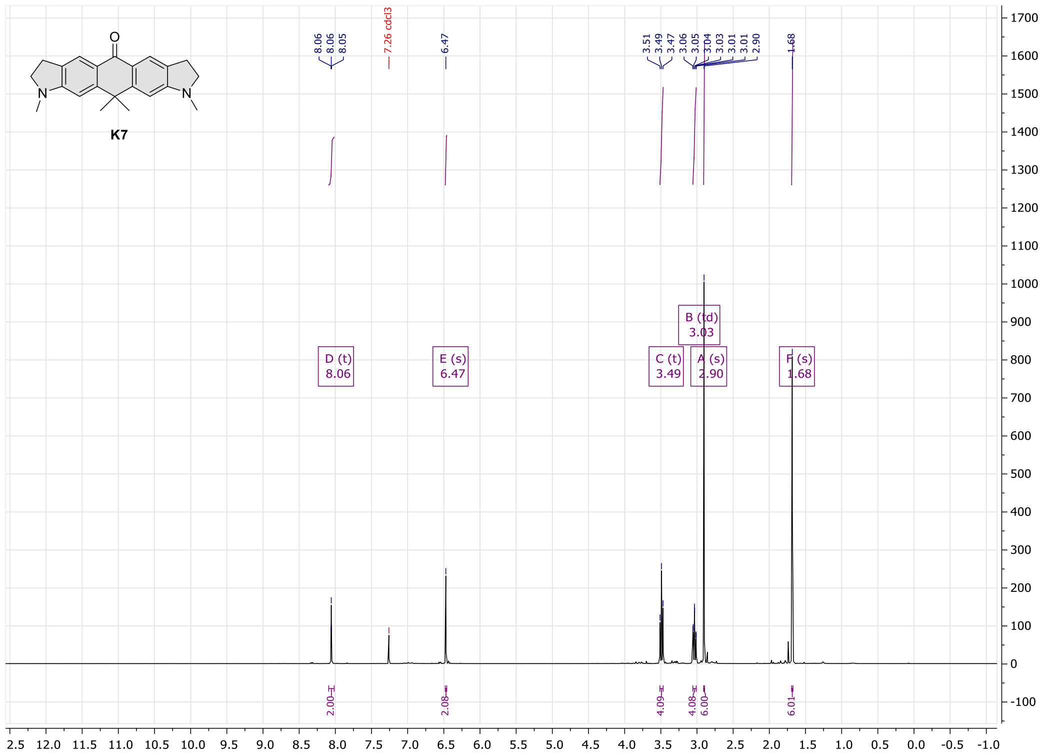

Supplementary Figure 163. ^{1}H NMR of compound **K7** (400 MHz, $CDCl_3$).

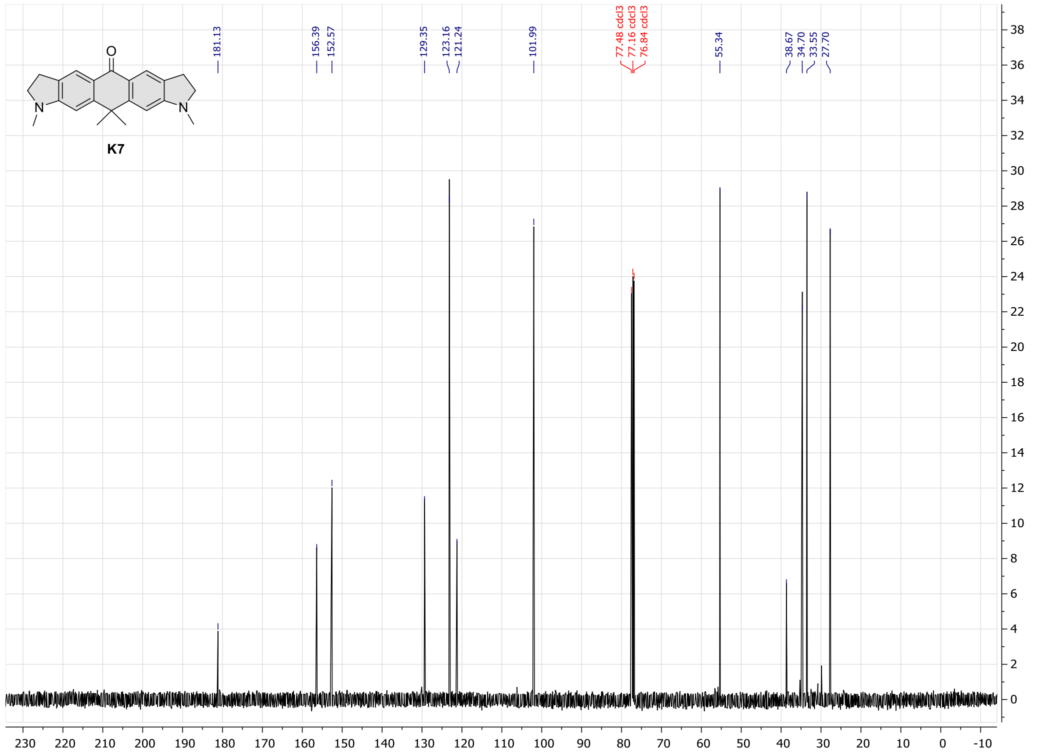

Supplementary Figure 164. ^{13}C NMR of compound **K7** (100 MHz, $CDCl_3$).

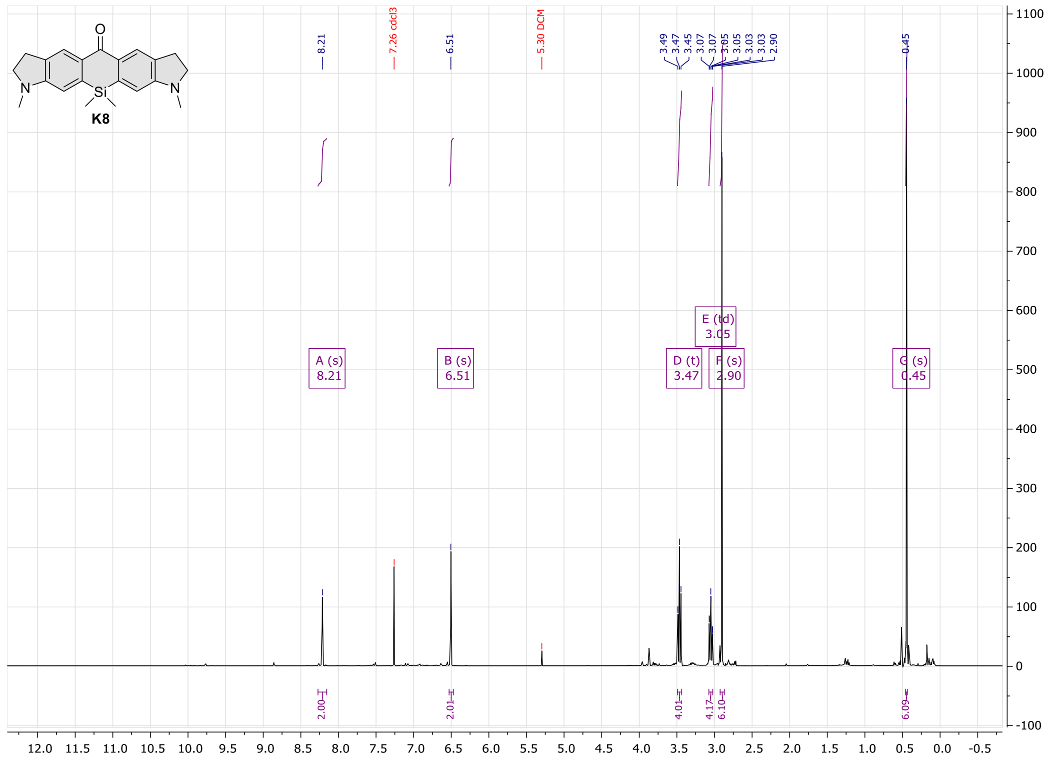

Supplementary Figure 165. ^{1}H NMR of compound **K8** (400 MHz, $CDCl_3$).

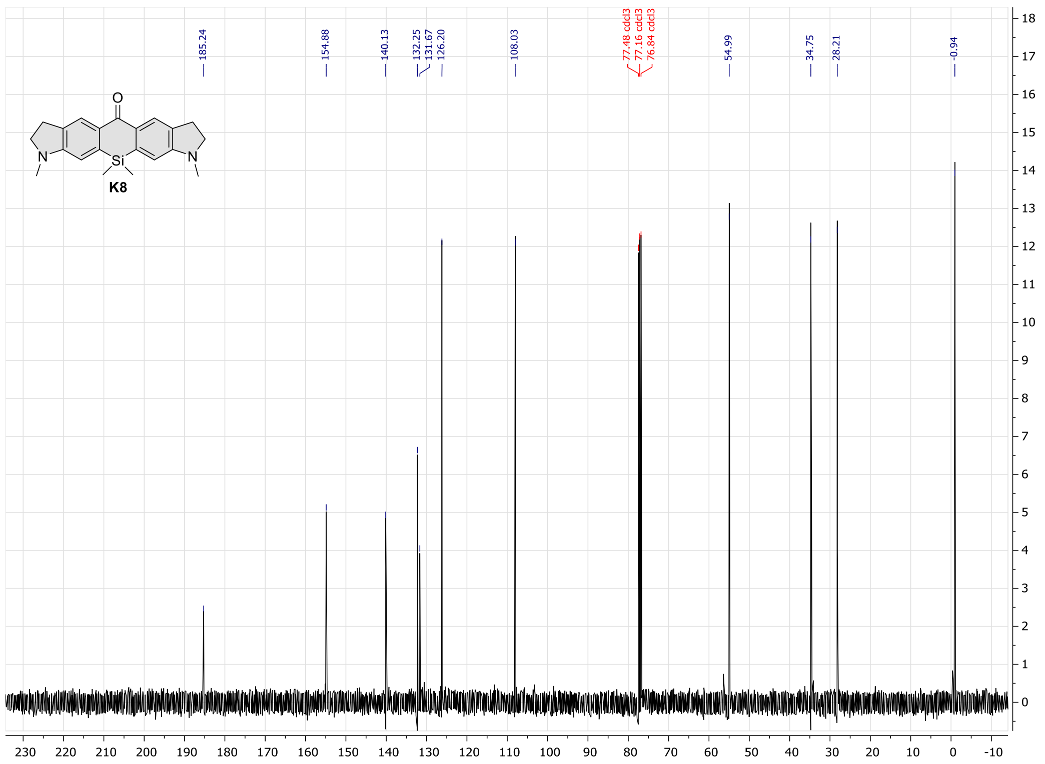

Supplementary Figure 166. ^{13}C NMR of compound **K8** (100 MHz, $CDCl_3$).

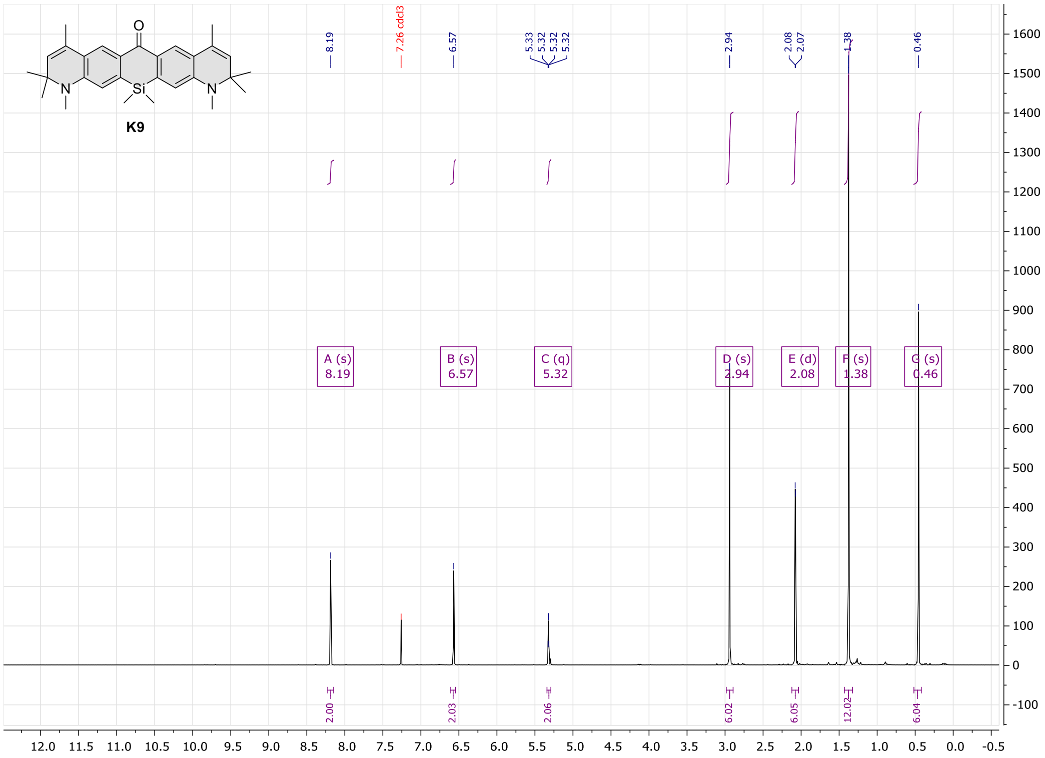

Supplementary Figure 167. ^{1}H NMR of compound **K9** (400 MHz, $CDCl_3$).

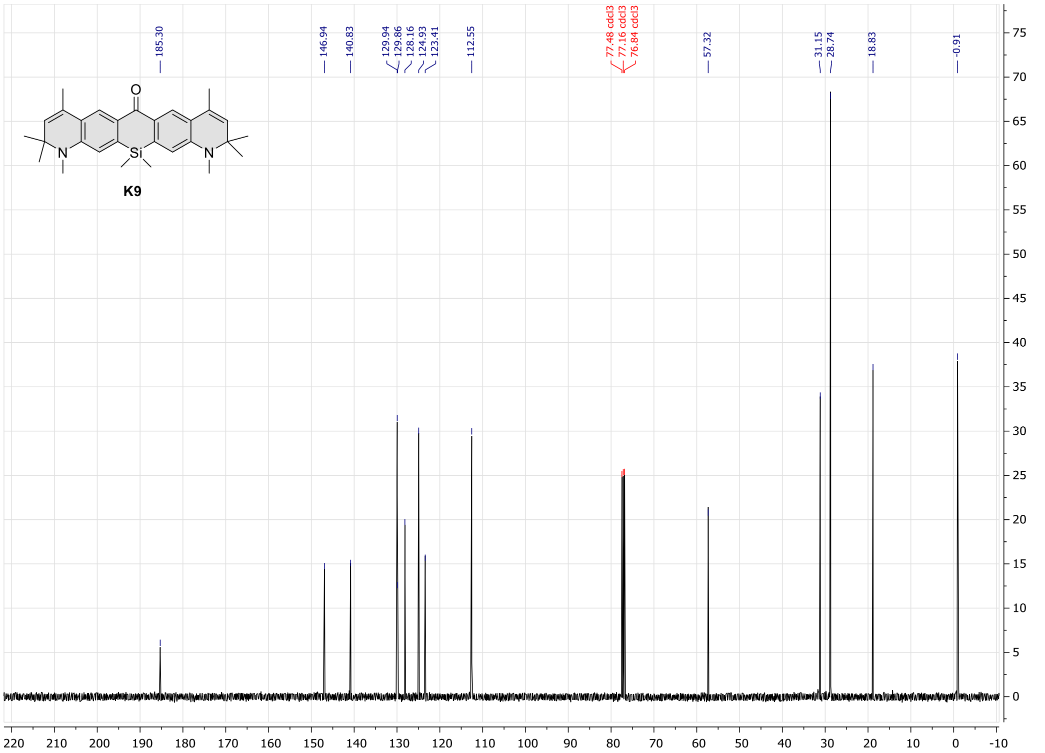

Supplementary Figure 168. ^{13}C NMR of compound **K9** (100 MHz, $CDCl_3$).

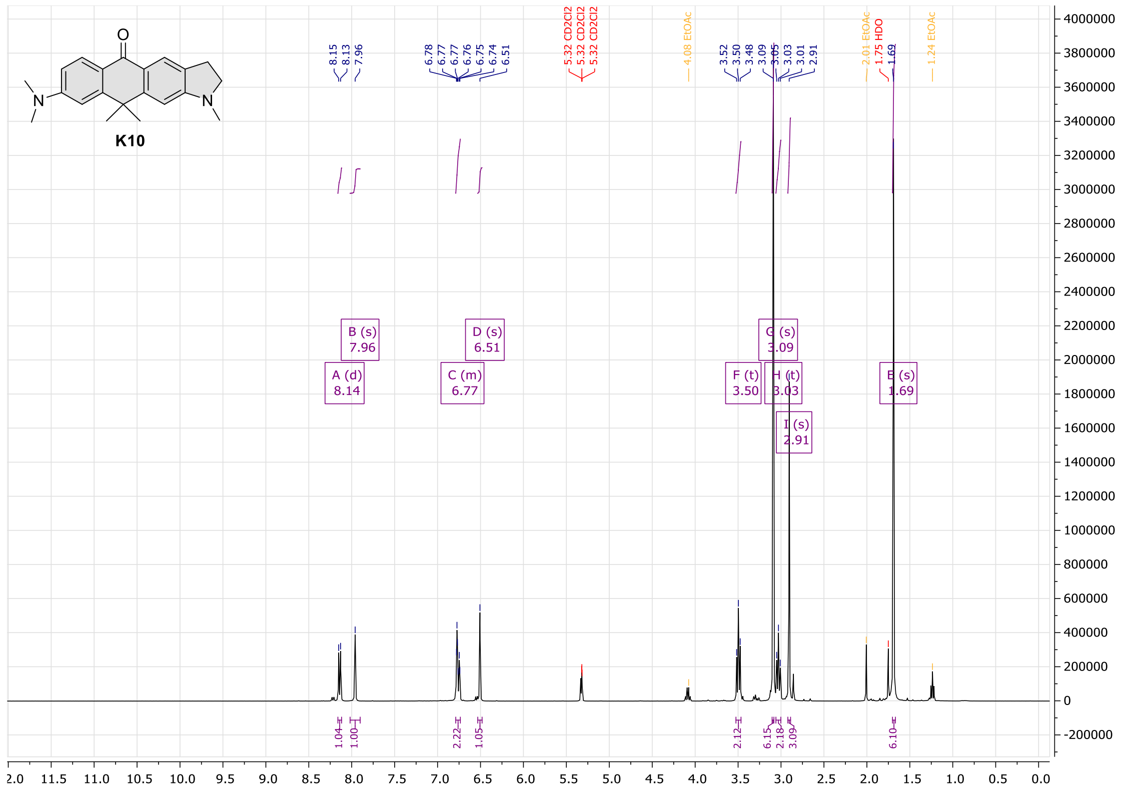

Supplementary Figure 169. ^{1}H NMR of compound **K10** (400 MHz, CD_2Cl_2).

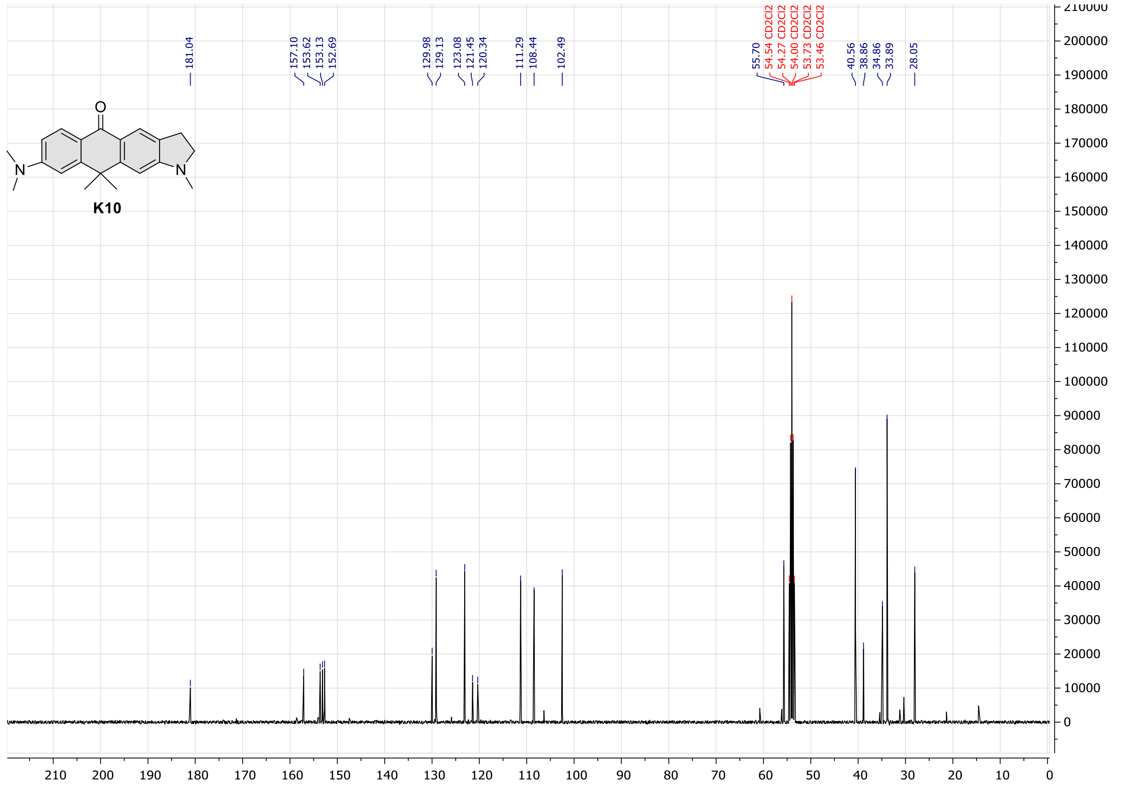

Supplementary Figure 170. ^{13}C NMR of compound **K10** (100 MHz, CD_2Cl_2).

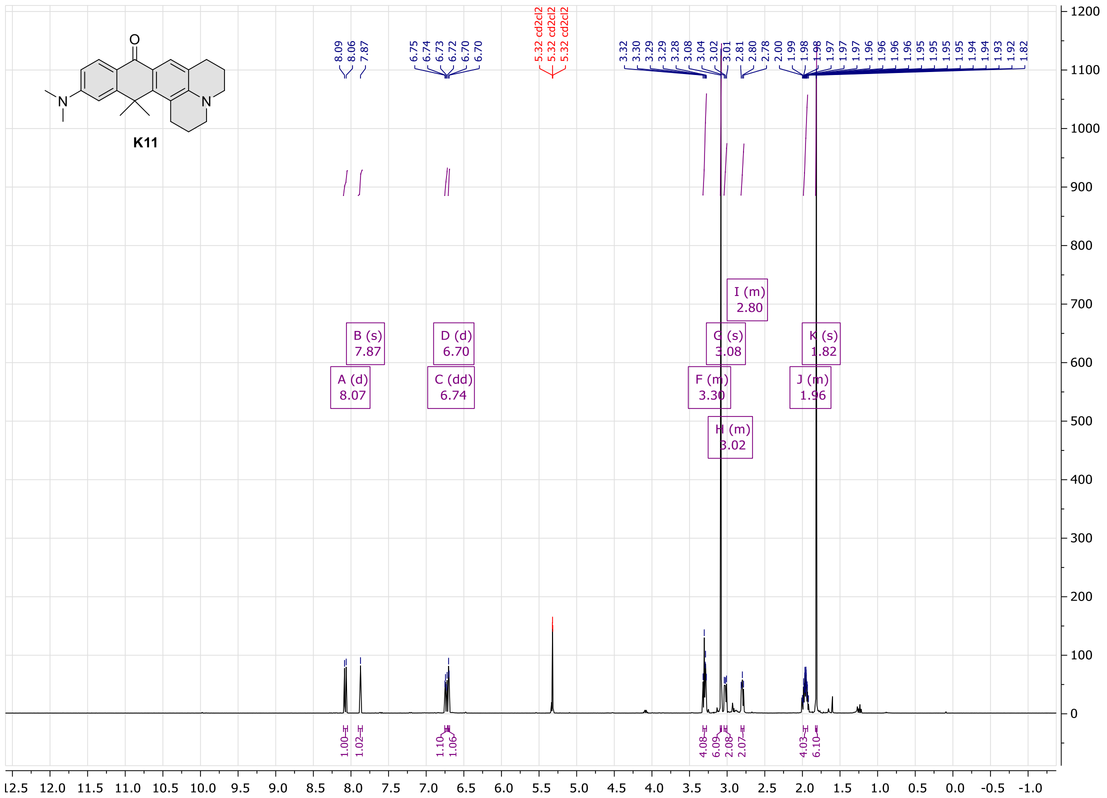

Supplementary Figure 171. ^{1}H NMR of compound **K11** (400 MHz, CD_2Cl_2).

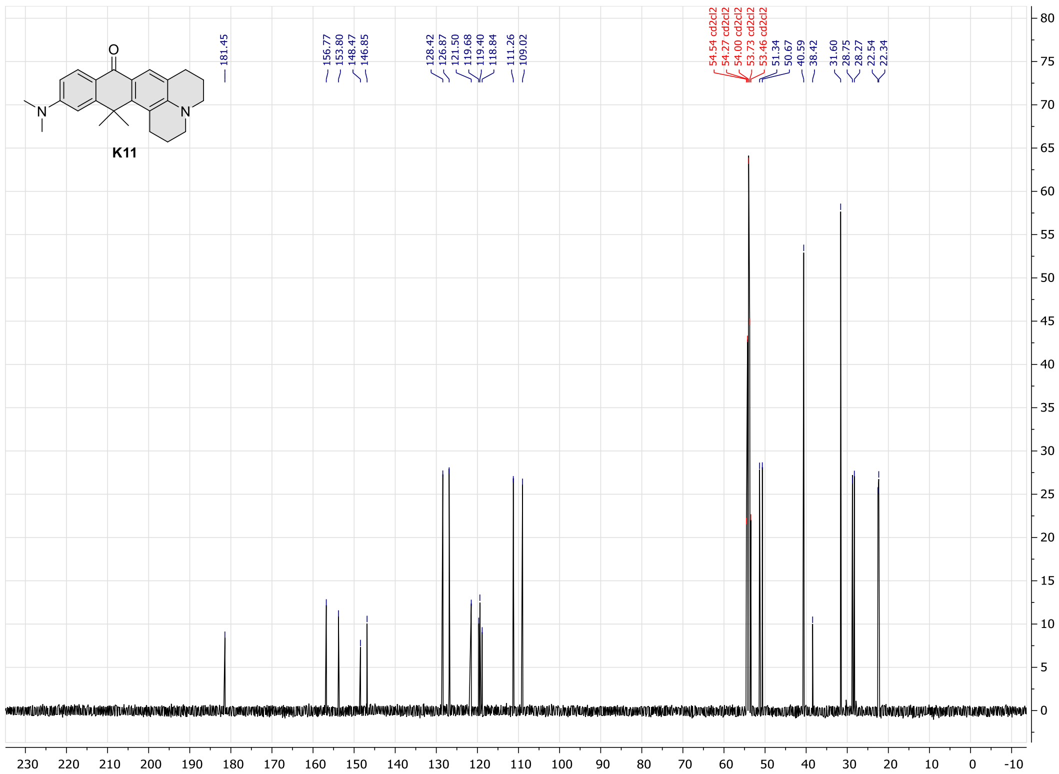

Supplementary Figure 172. ^{13}C NMR of compound **K11** (400 MHz, CD_2Cl_2).

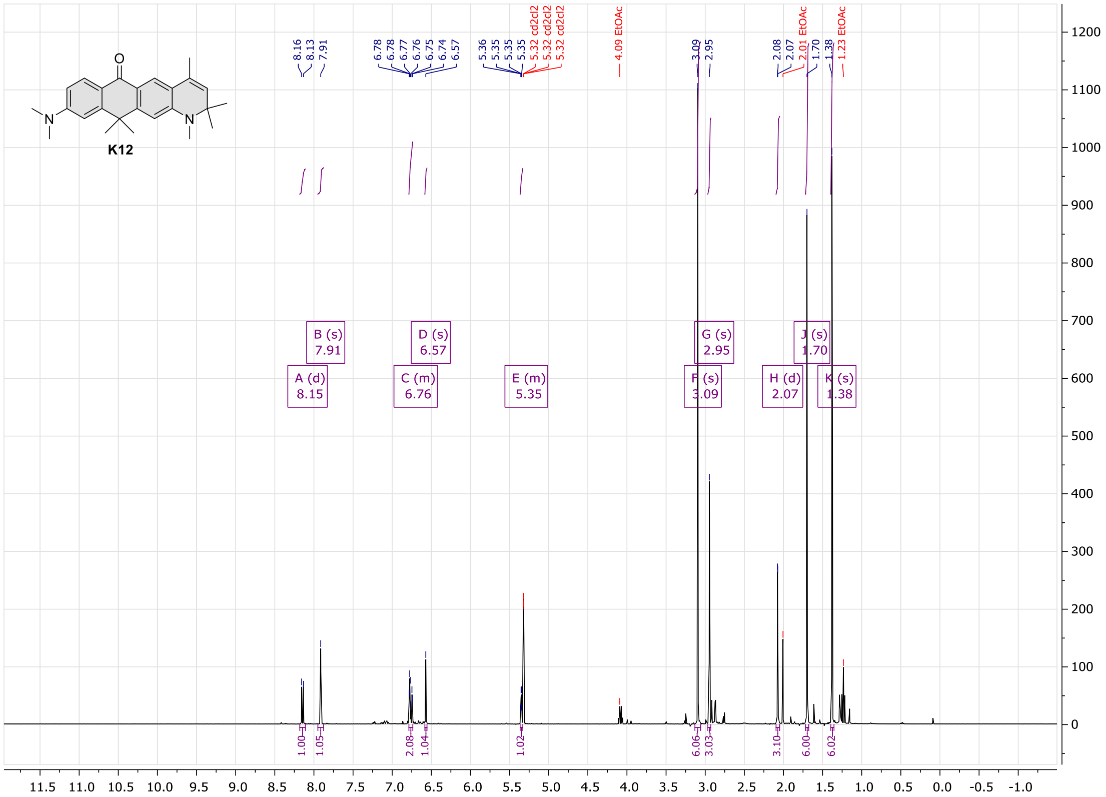

Supplementary Figure 173. ^{1}H NMR of compound **K12** (400 MHz, CD_2Cl_2).

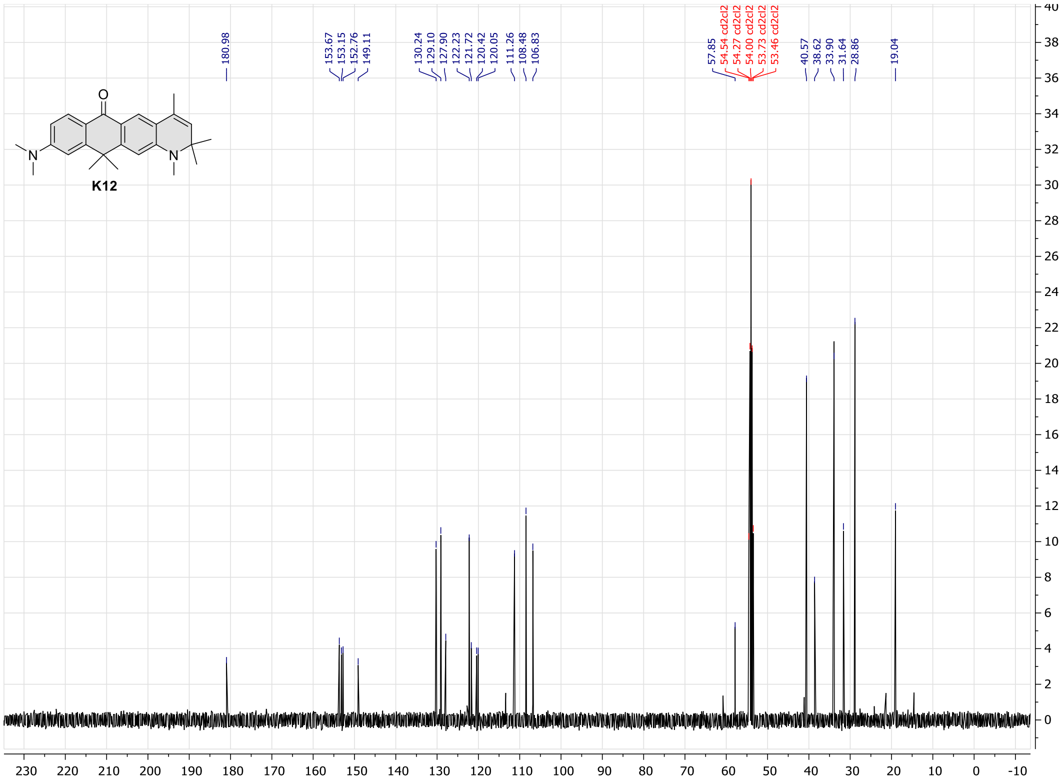

Supplementary Figure 174. ^{13}C NMR of compound **K12** (100 MHz, CD_2Cl_2).

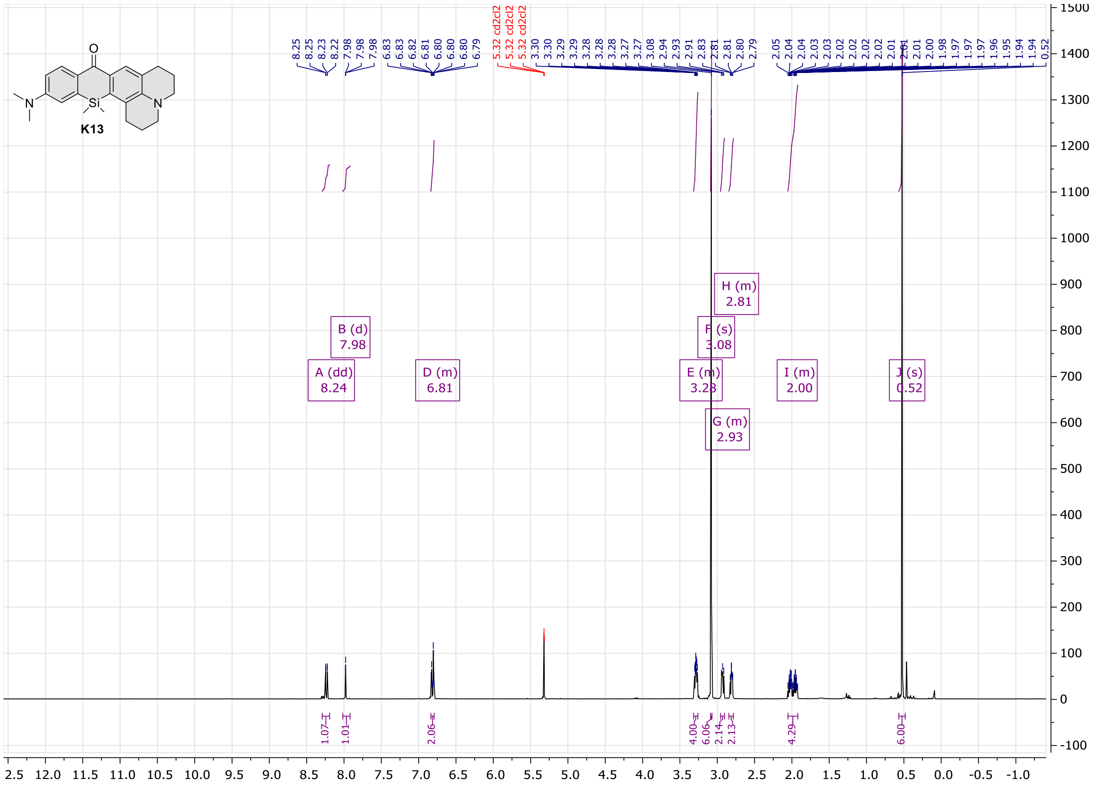

Supplementary Figure 175. ^1H NMR of compound **K13** (400 MHz, CD_2Cl_2)**.**

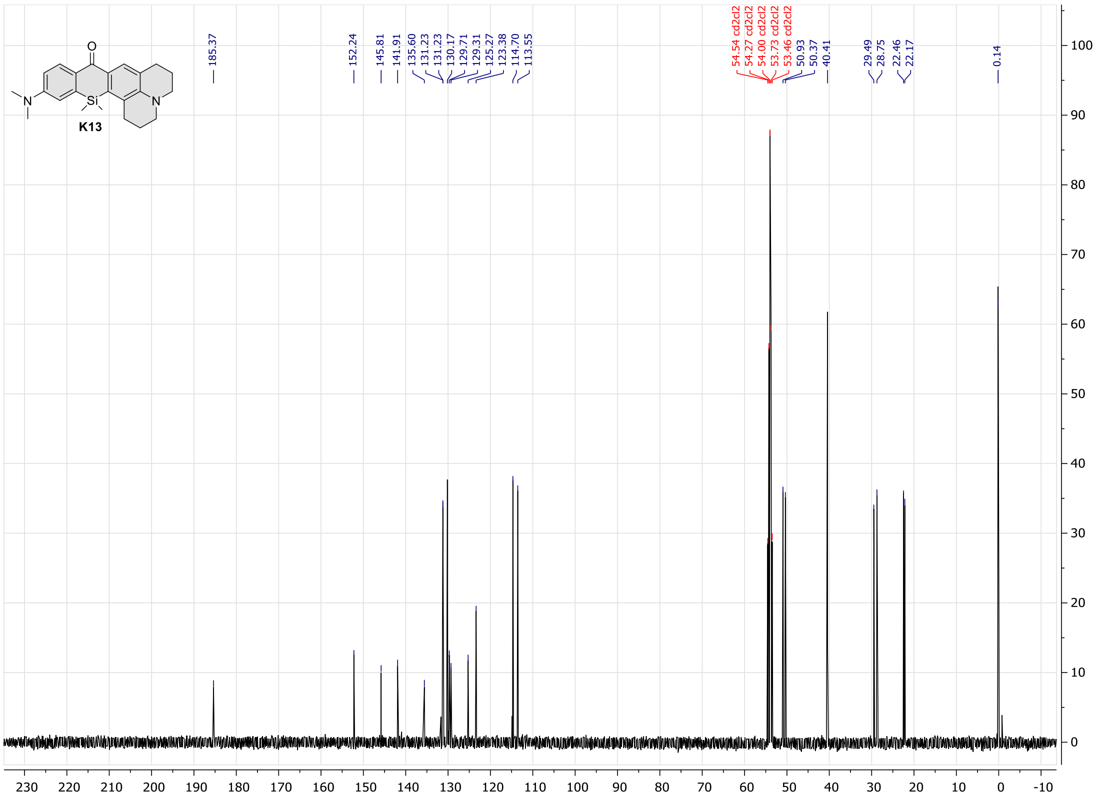

Supplementary Figure 176. ^{13}C NMR of compound **K13** (100 MHz, CD_2Cl_2).

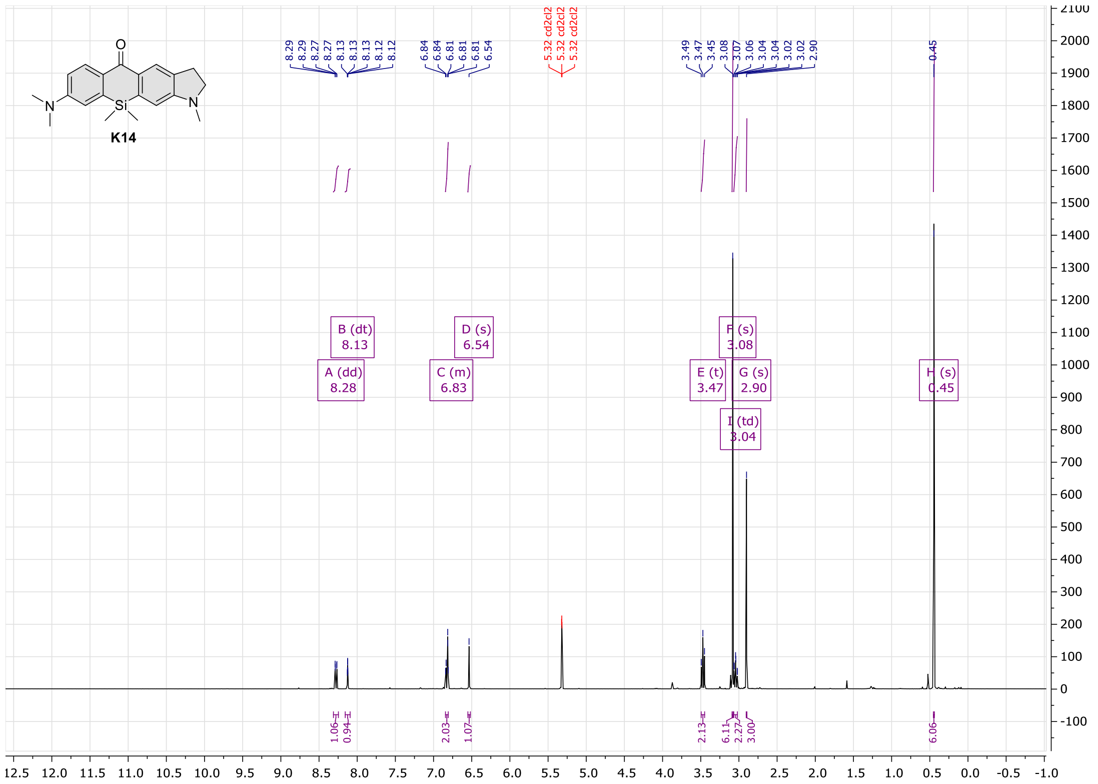

Supplementary Figure 177. ^{1}H NMR of compound **K14** (400 MHz, CD_2Cl_2).

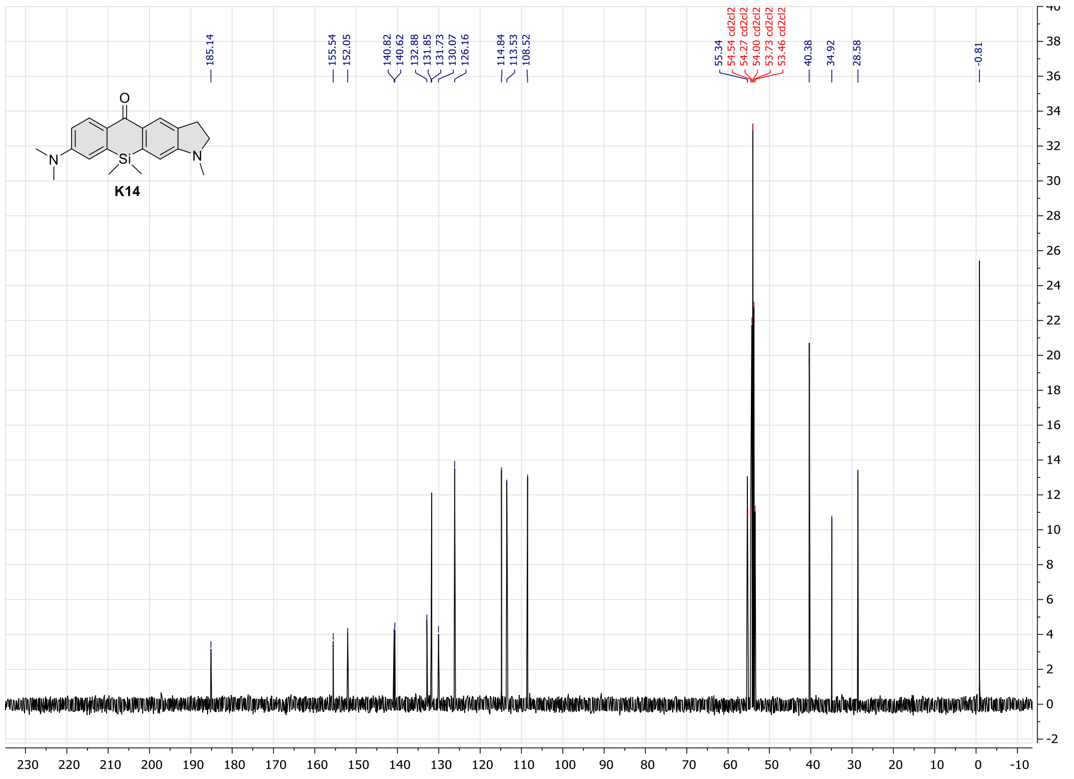

Supplementary Figure 178. ^{13}C NMR of compound **K14** (100 MHz, CD_2Cl_2).

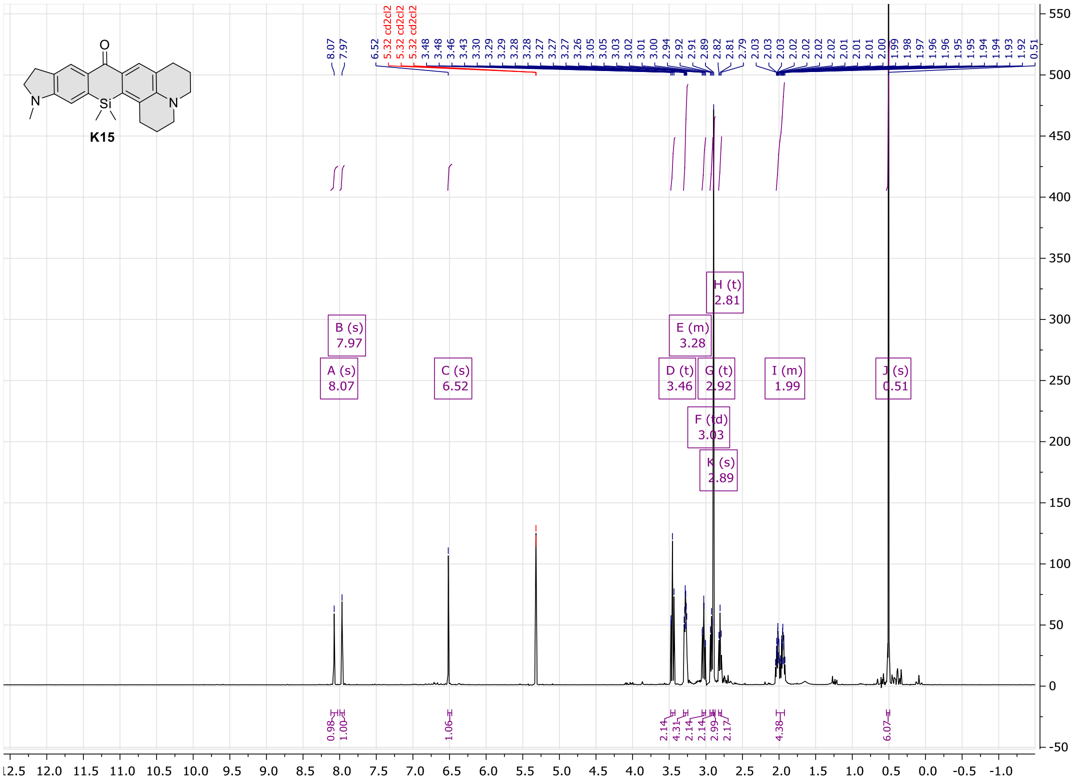

Supplementary Figure 179. ^{1}H NMR of compound **K15** (400 MHz, CD_2Cl_2).

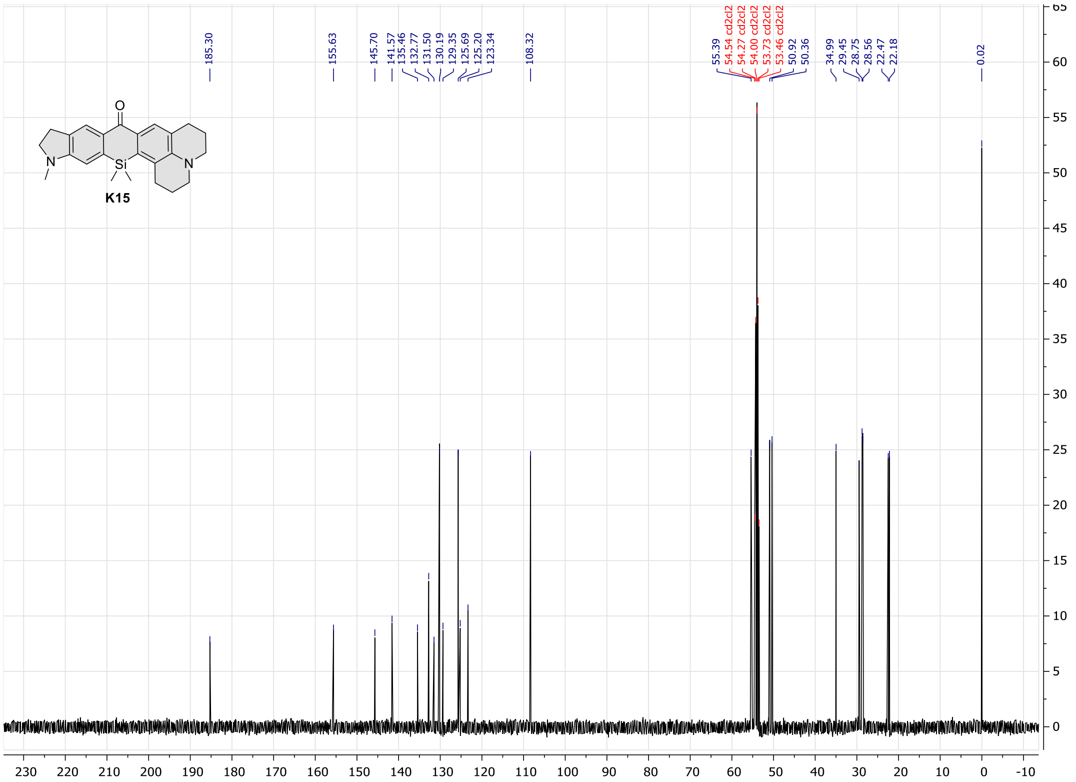

Supplementary Figure 180. ^{13}C NMR of compound **K15** (100 MHz, CD_2Cl_2).

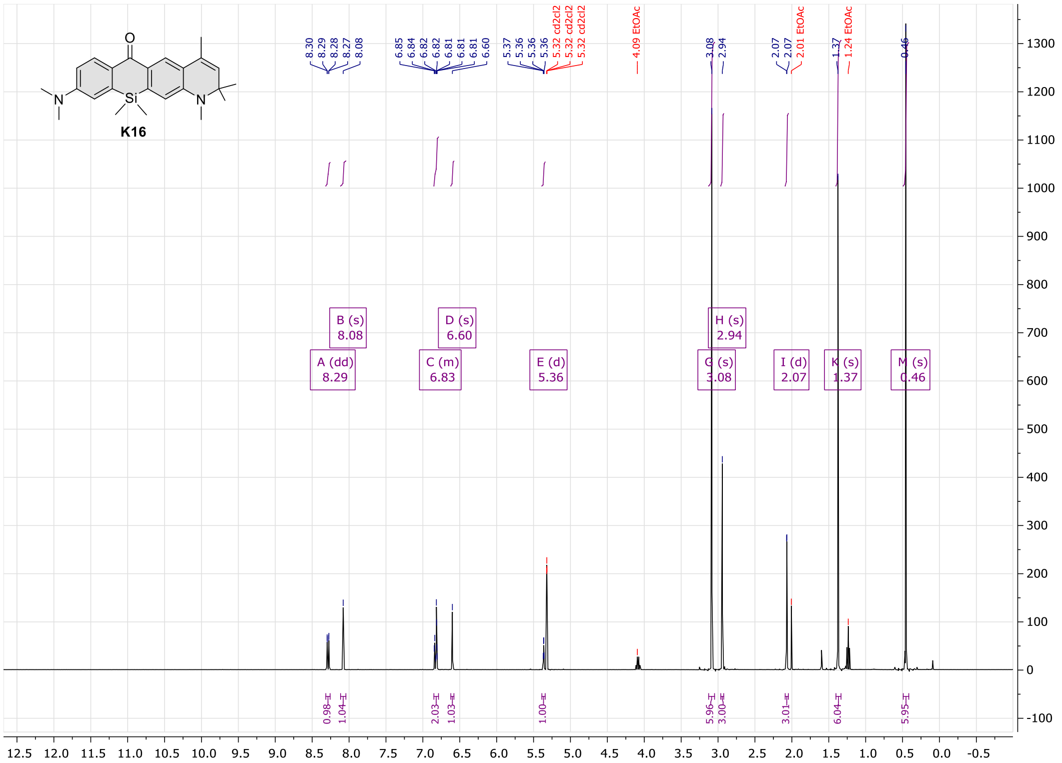

Supplementary Figure 181. ^{1}H NMR of compound **K16** (400 MHz, CD_2Cl_2).

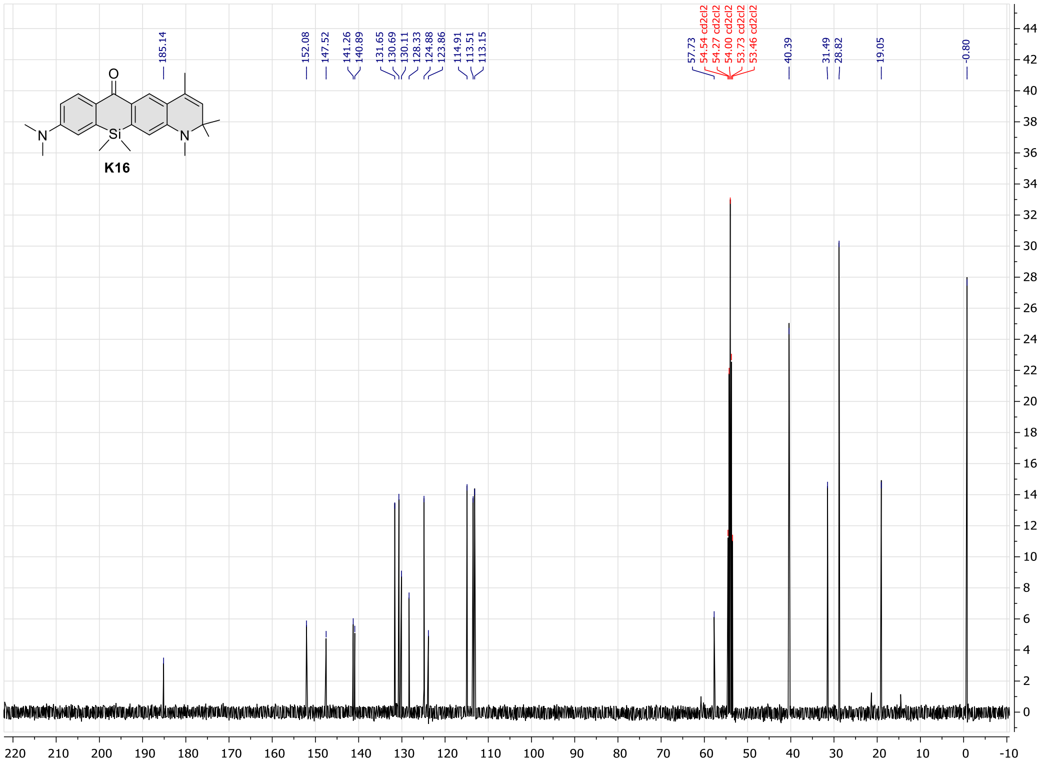

Supplementary Figure 182. ^{13}C NMR of compound **K16** (100 MHz, CD_2Cl_2).

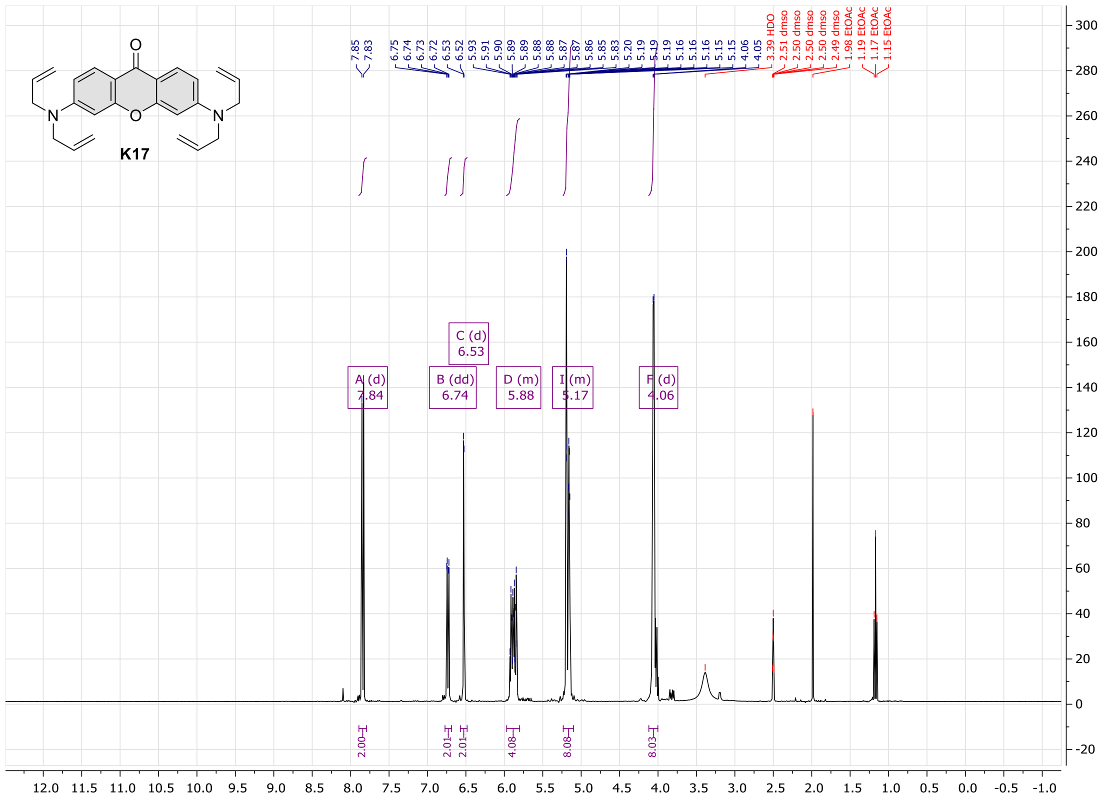

Supplementary Figure 183. ^{1}H NMR of compound **K17** (400 MHz, d_6-DMSO).

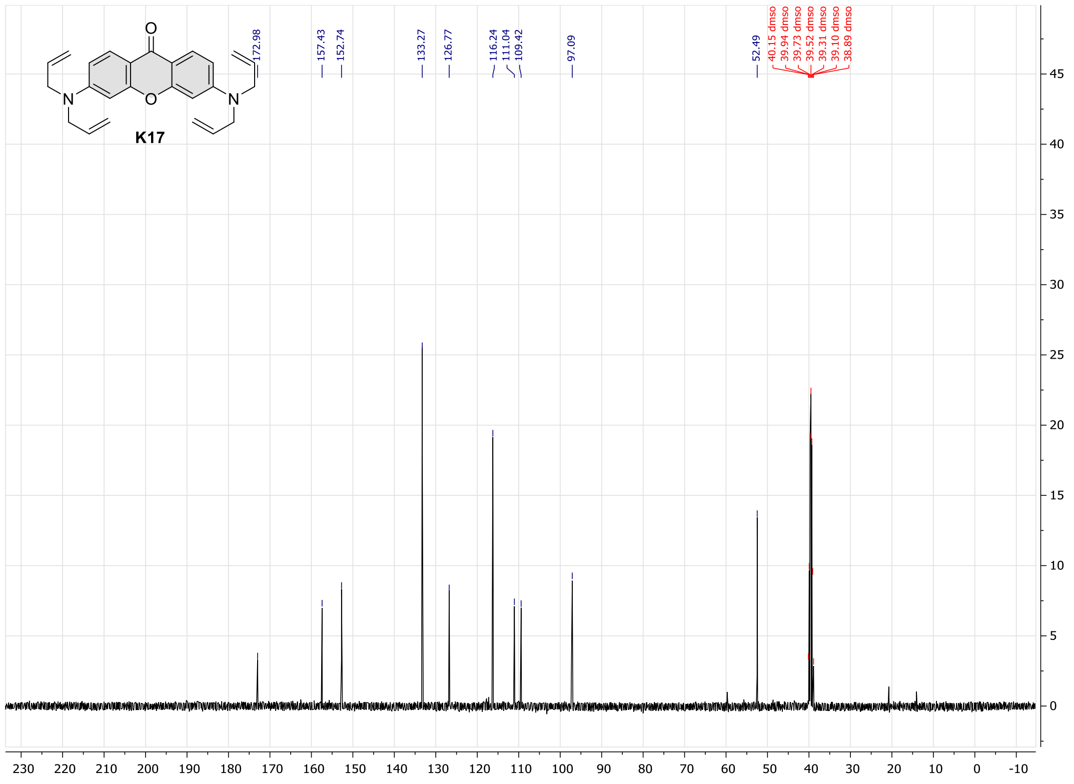

Supplementary Figure 184. ^{13}C NMR of compound **K17** (100 MHz, d_6-DMSO).

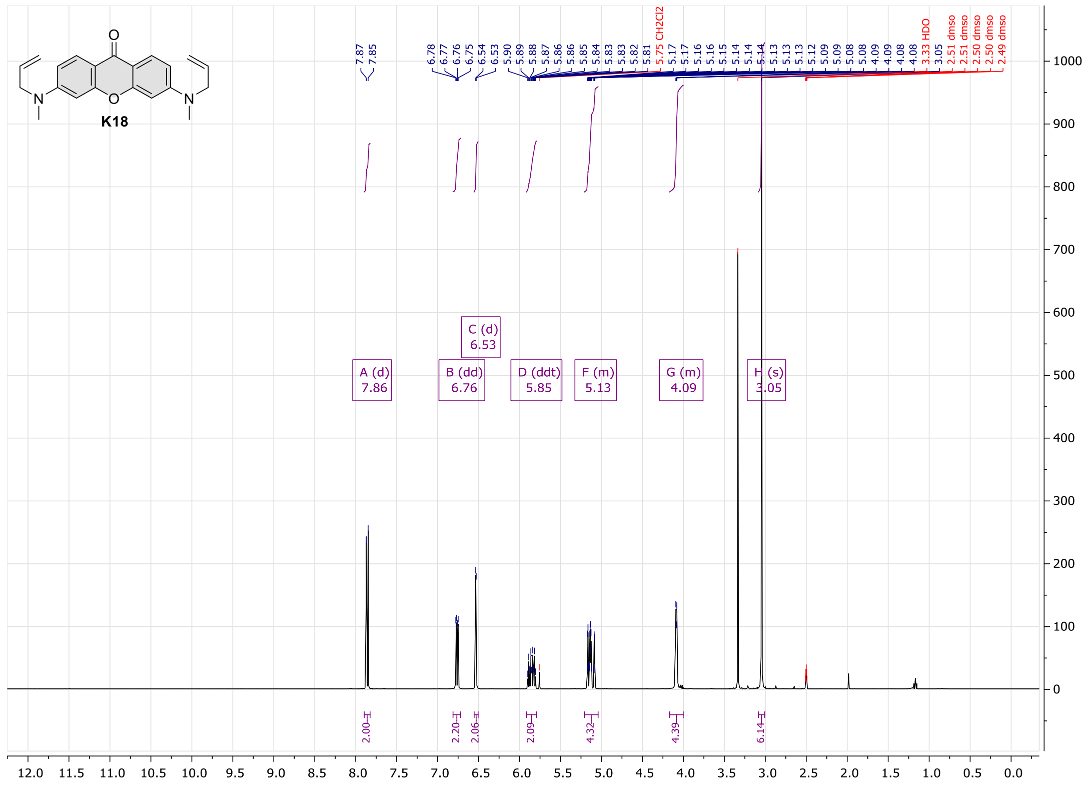

Supplementary Figure 185. ^{1}H NMR of compound **K18** (400 MHz, d_6-DMSO).

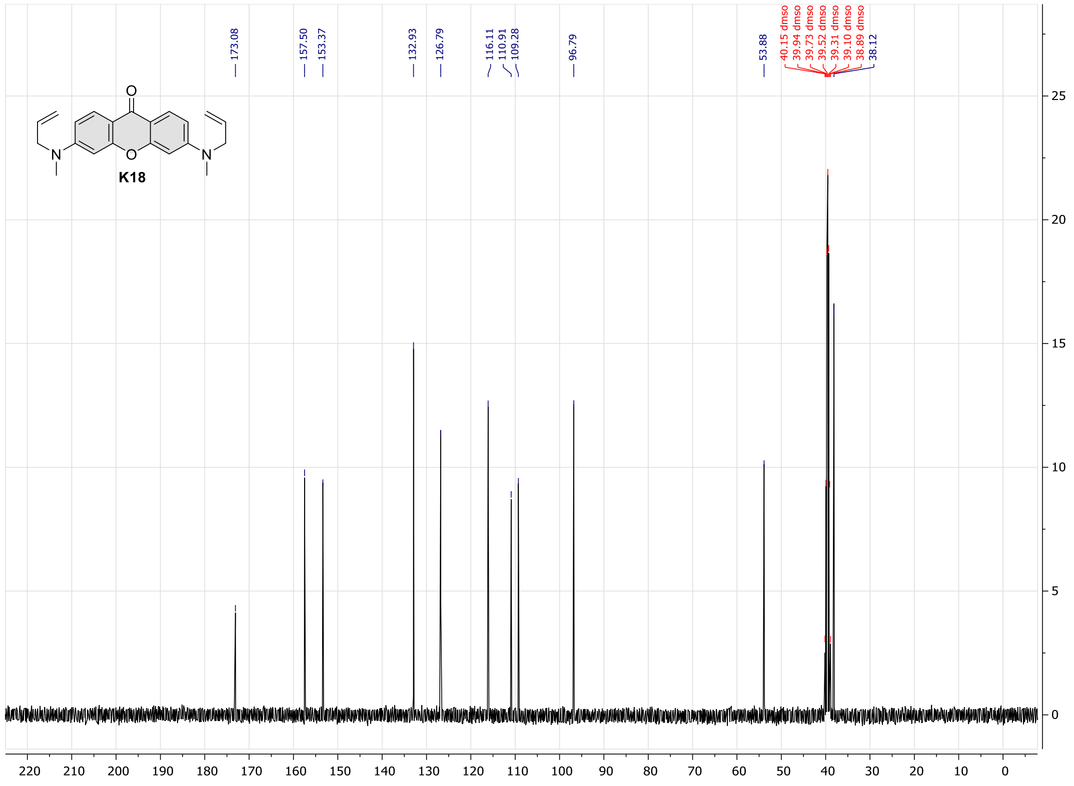

Supplementary Figure 186. ^{13}C NMR of compound **K18** (100 MHz, d_6-DMSO).

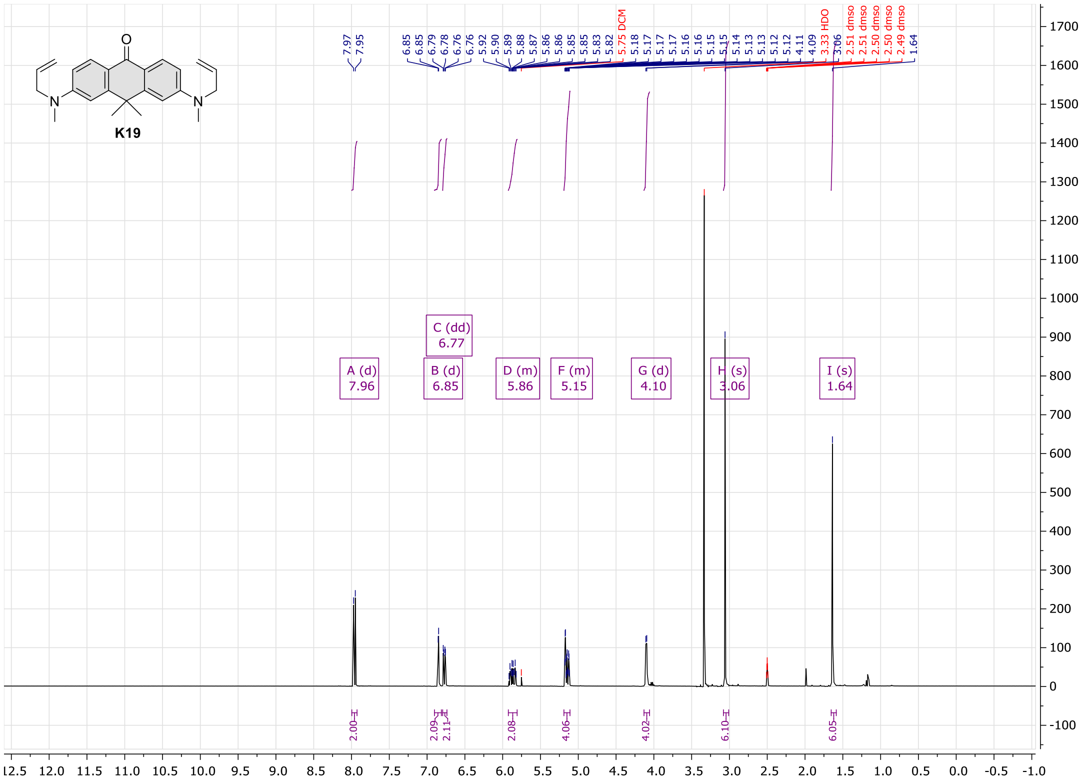

Supplementary Figure 187. ^{1}H NMR of compound **K19** (400 MHz, d_6-DMSO).

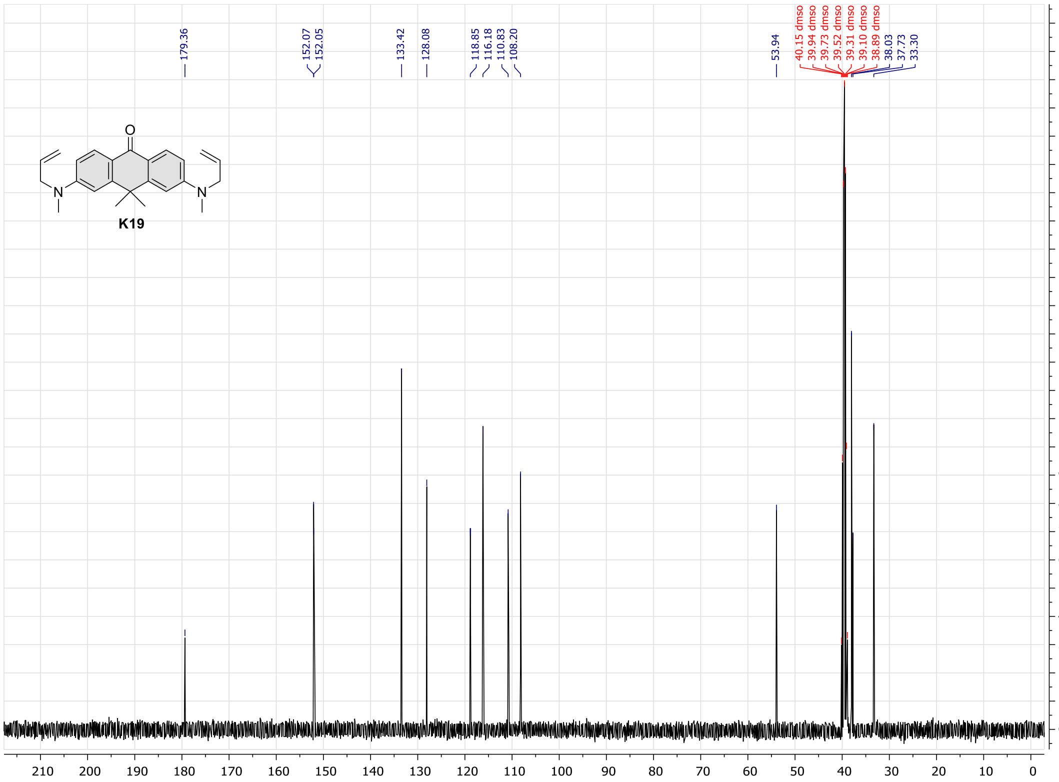

Supplementary Figure 188. ^{13}C NMR of compound **K19** (100 MHz, d_6-DMSO).

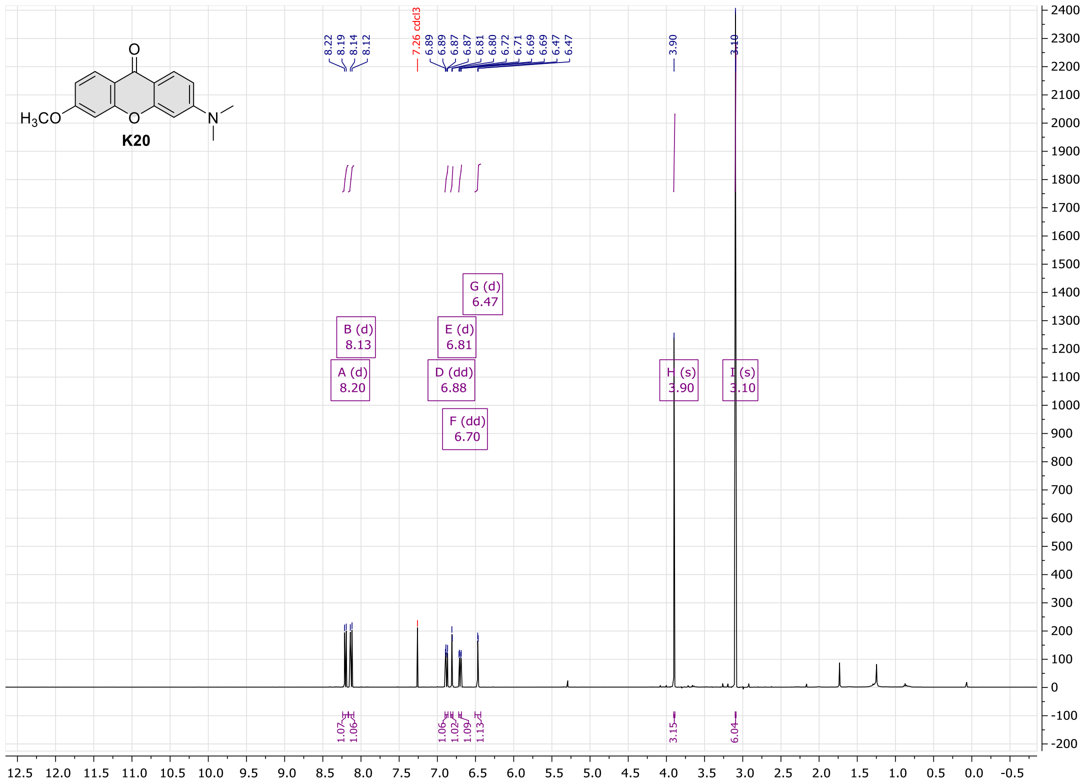

Supplementary Figure 189. ^{1}H NMR of compound **K20** (400 MHz, $CDCl_3$).

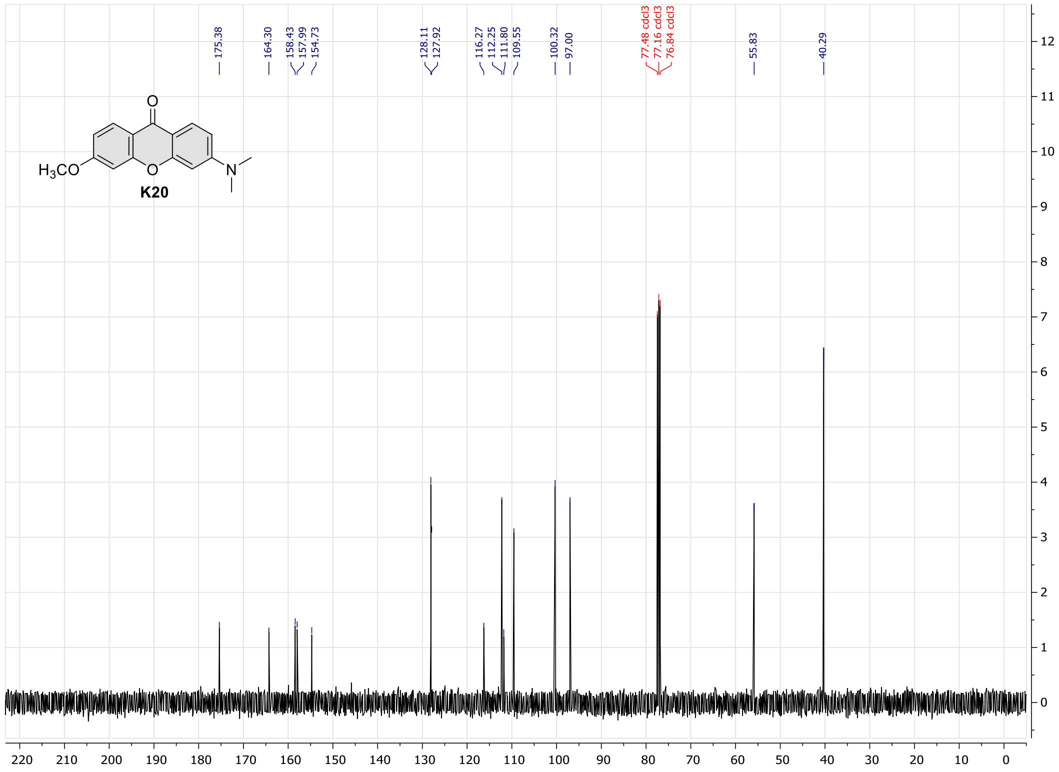

Supplementary Figure 190. ^{13}C NMR of compound **K20** (100 MHz, $CDCl_3$).

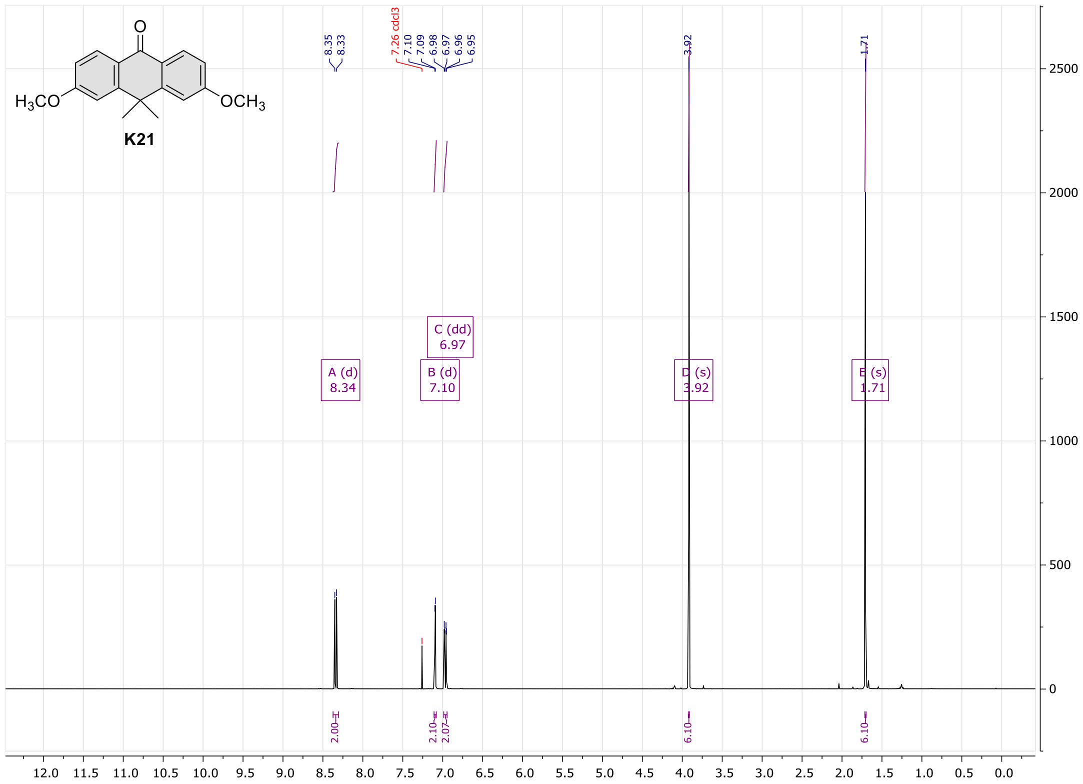

Supplementary Figure 191. ^{1}H NMR of compound **K21** (400 MHz, $CDCl_3$).

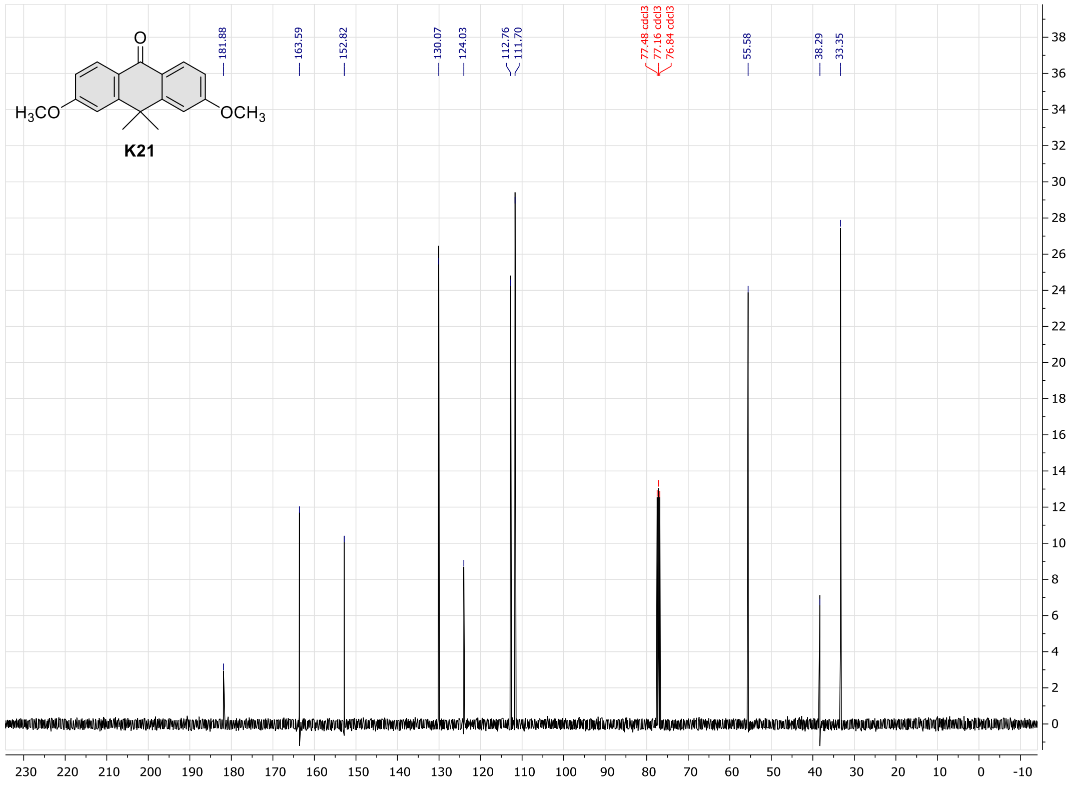

Supplementary Figure 192. ^{13}C NMR of compound **K21** (100 MHz, $CDCl_3$).

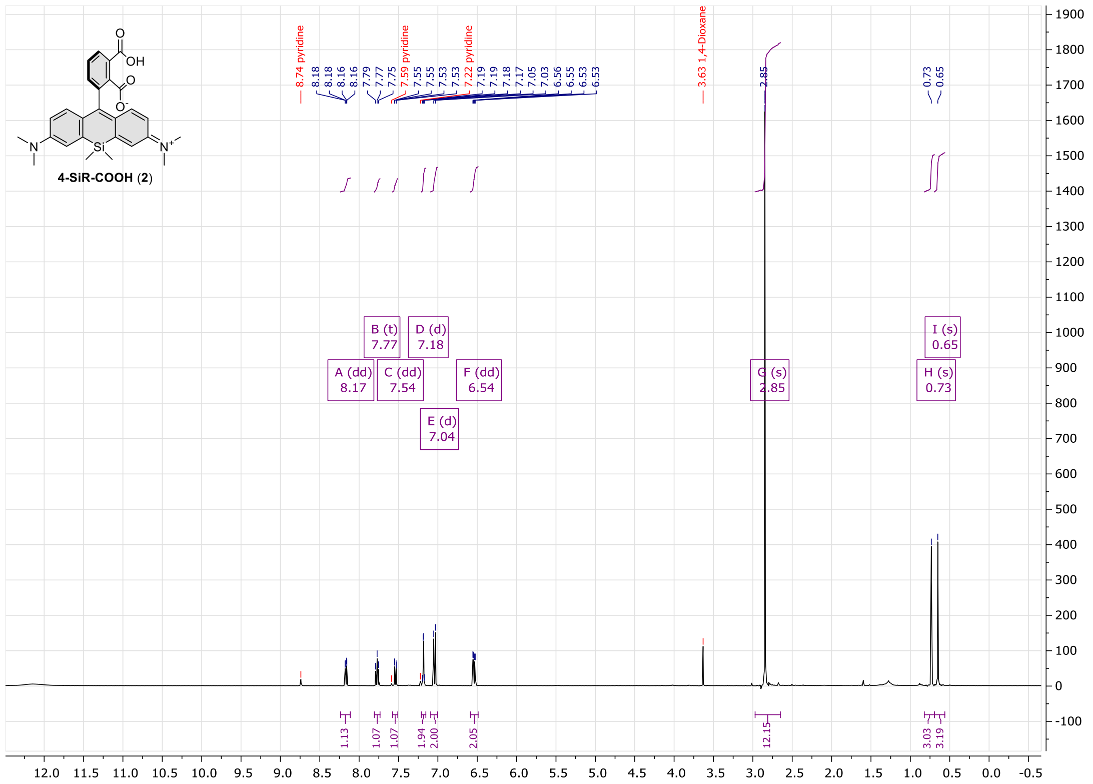

Supplementary Figure 193. ^{1}H NMR of compound **2** (400 MHz, d5-Pyridine).

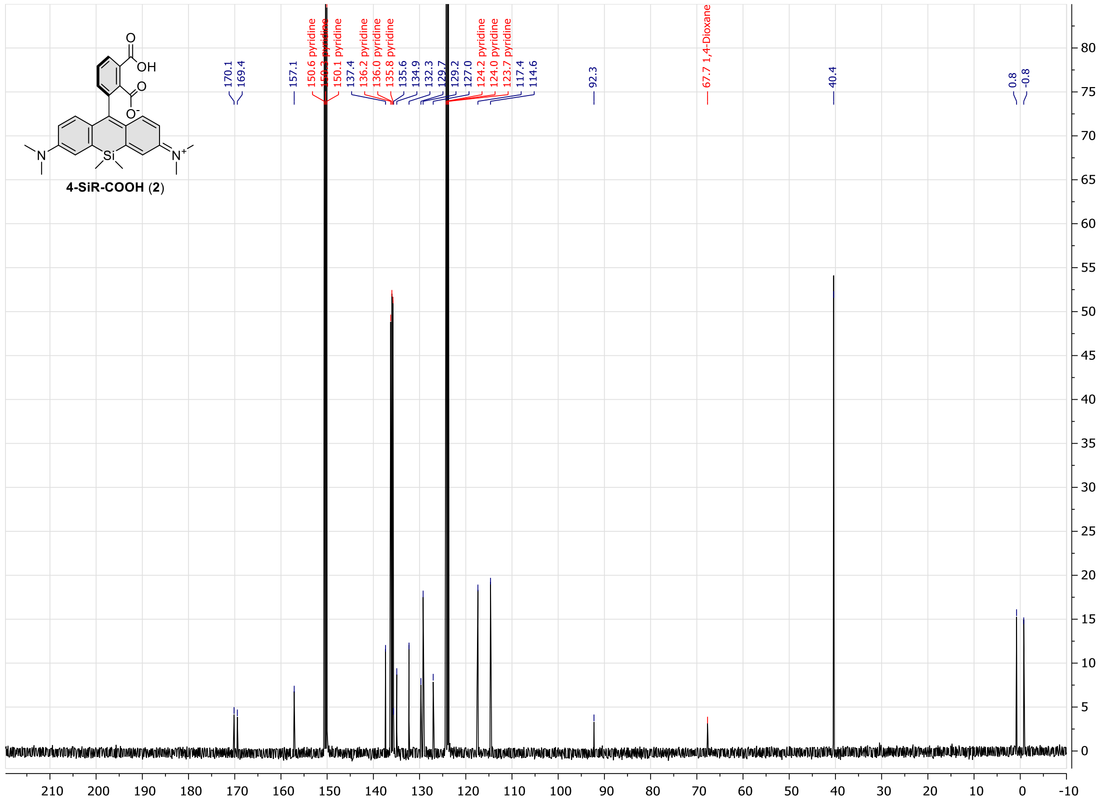

Supplementary Figure 194. ^{13}C NMR of compound **2** (100 MHz, d5-Pyridine).

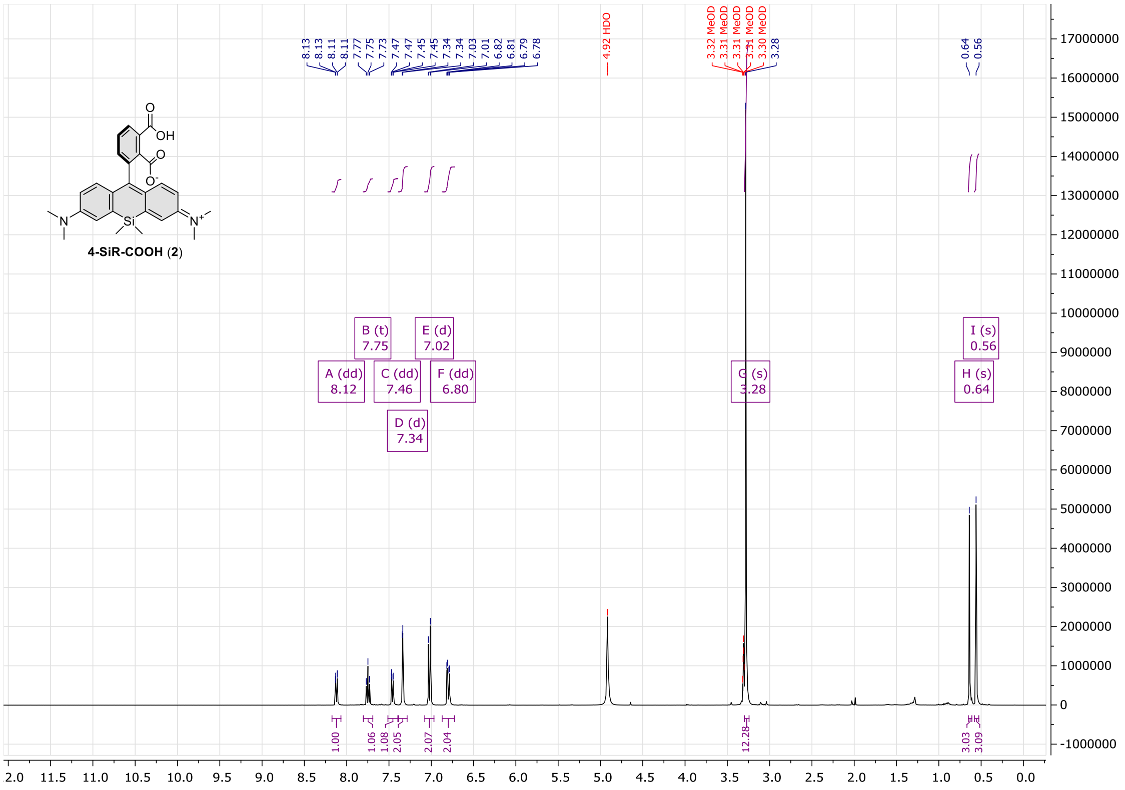

Supplementary Figure 195. ^{1}H NMR of compound **2** (400 MHz, CD_3OD+CF_3COOD).

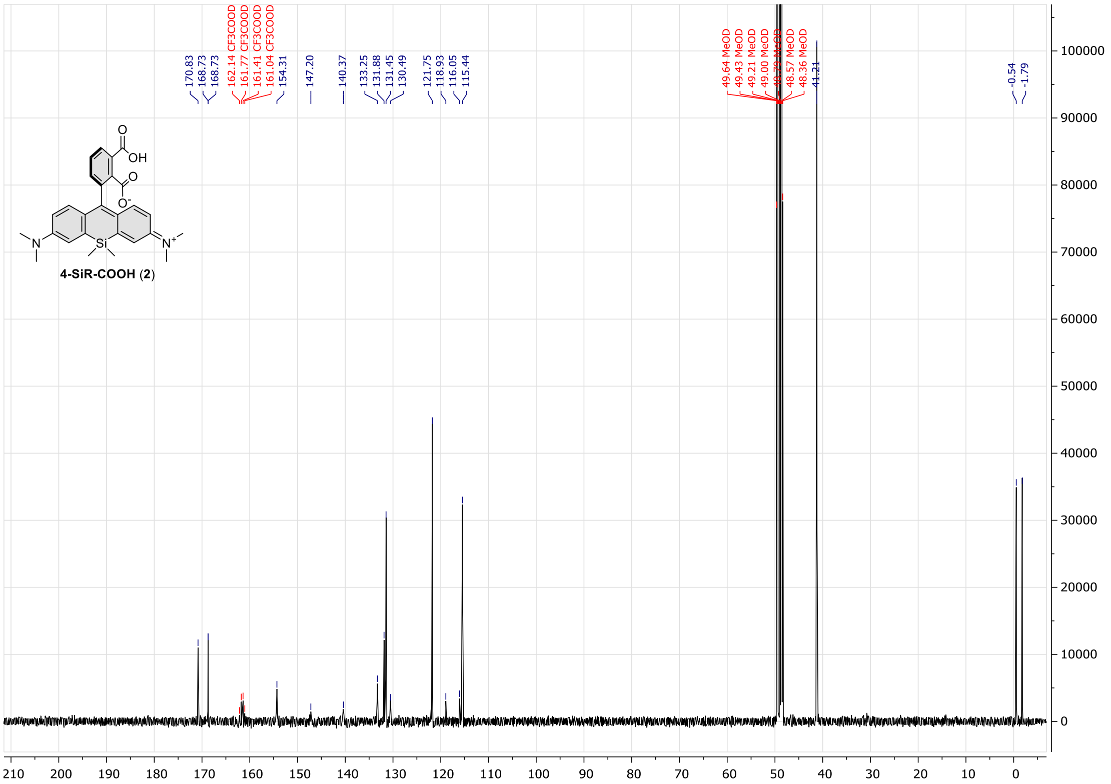

Supplementary Figure 196. ^{13}C NMR of compound **2** (100 MHz, CD_3OD+CF_3COOD).

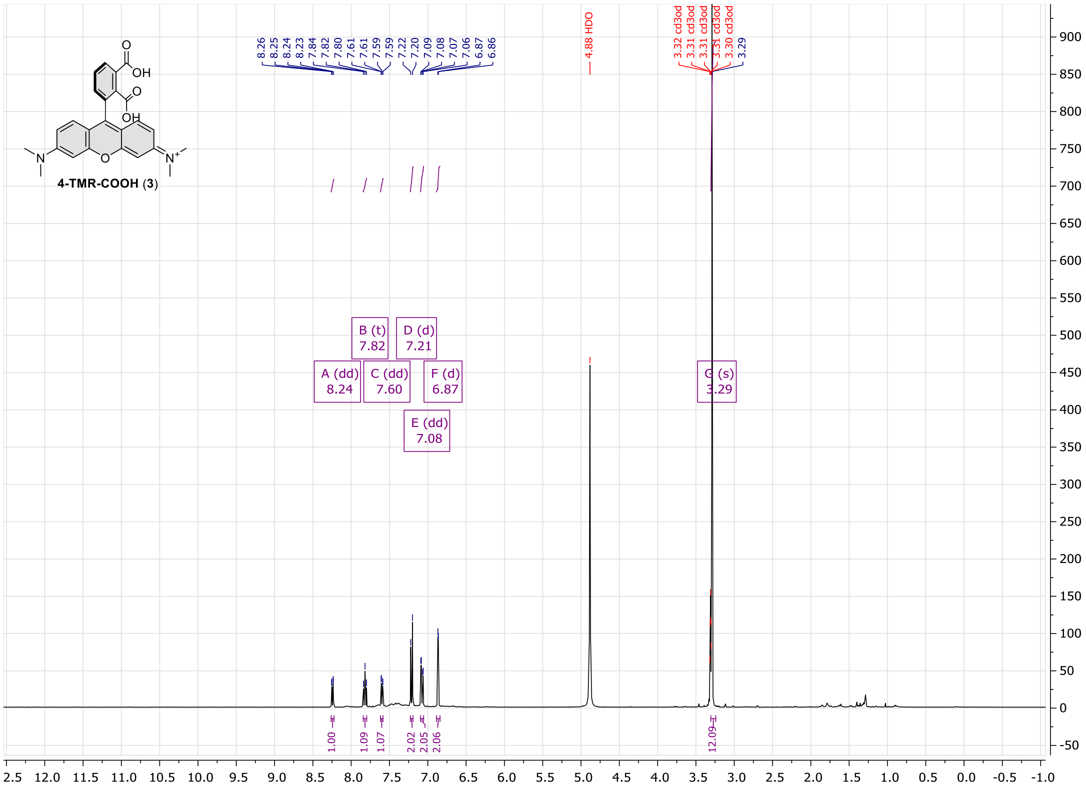

Supplementary Figure 197. ^{1}H NMR of compound **3** (400 MHz, CD_3OD).

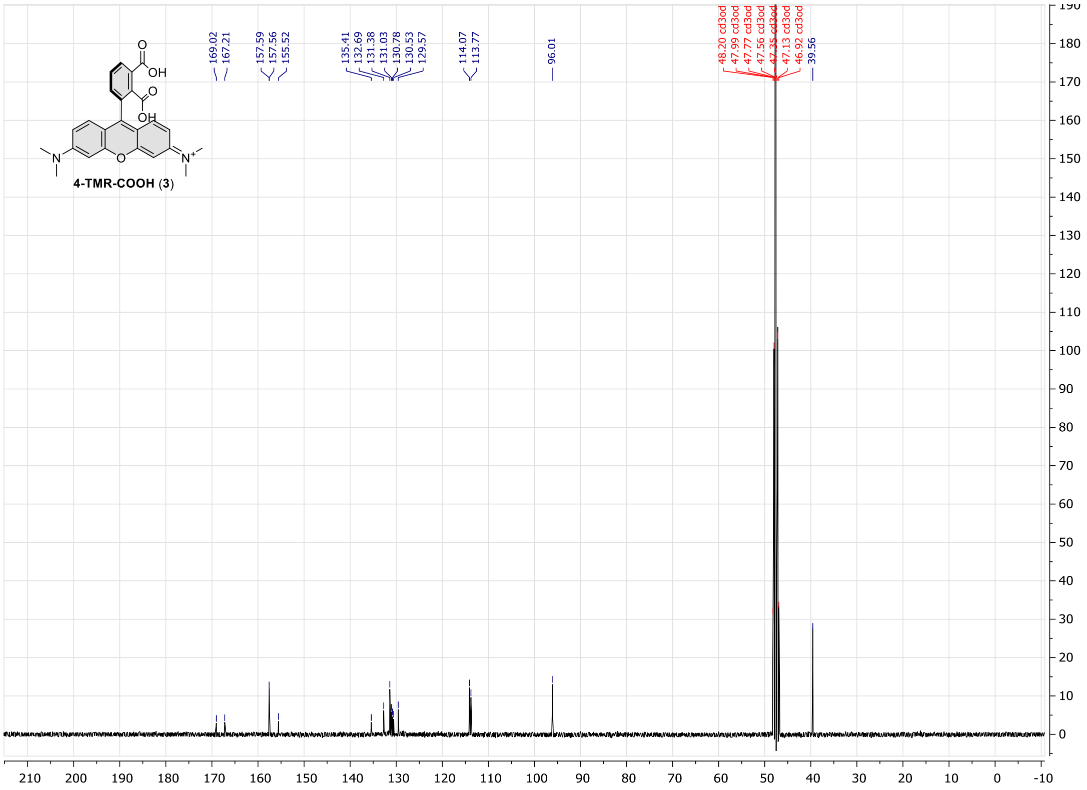

Supplementary Figure 198. ^{13}C NMR of compound **3** (100 MHz, CD_3OD).

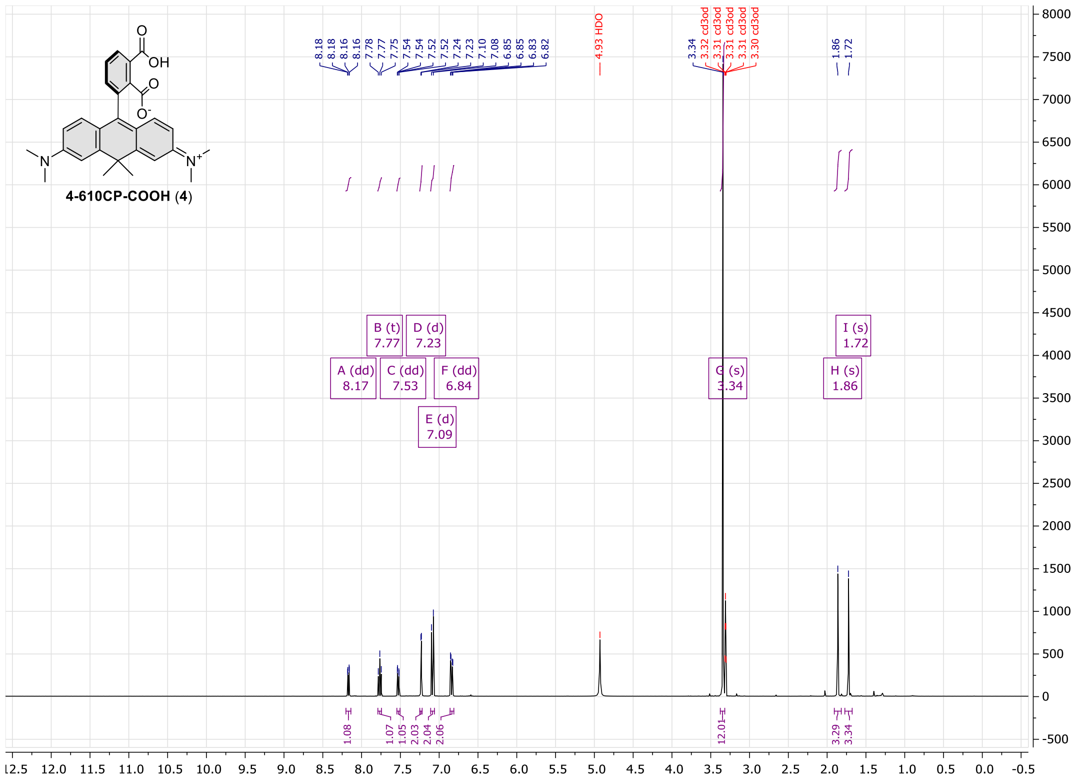

Supplementary Figure 199. ^{1}H NMR of compound **4** (400 MHz, CD_3OD).

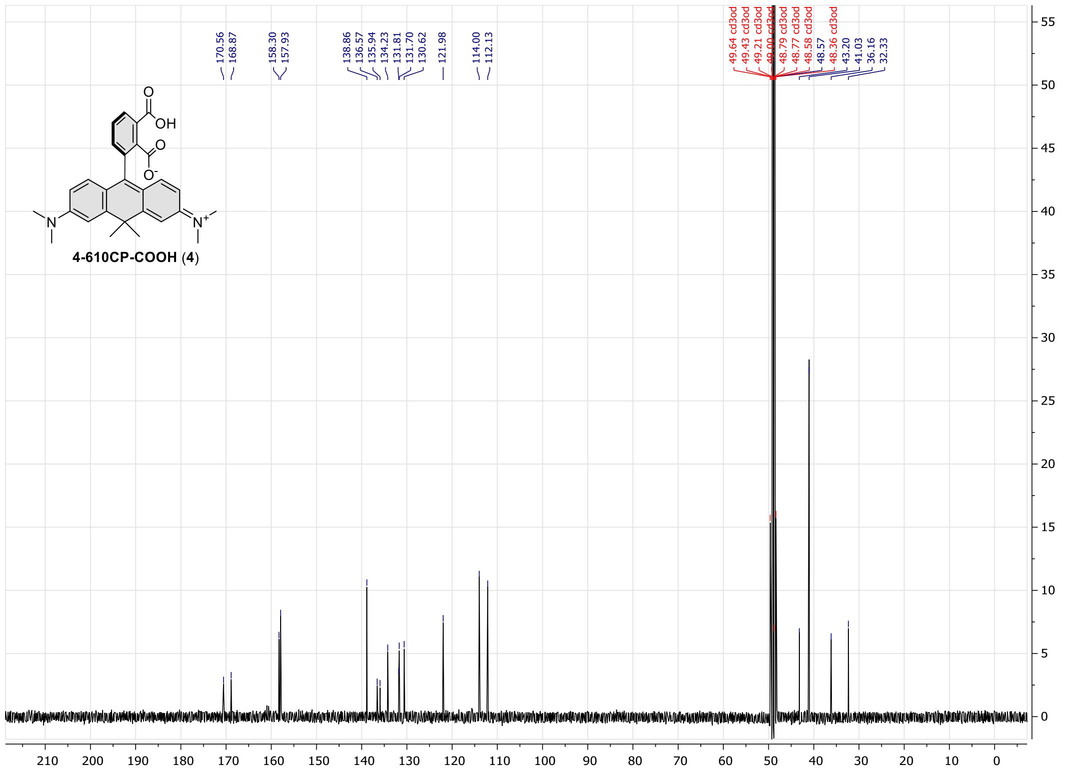

Supplementary Figure 200. ^{13}C NMR of compound **4** (100 MHz, CD_3OD).

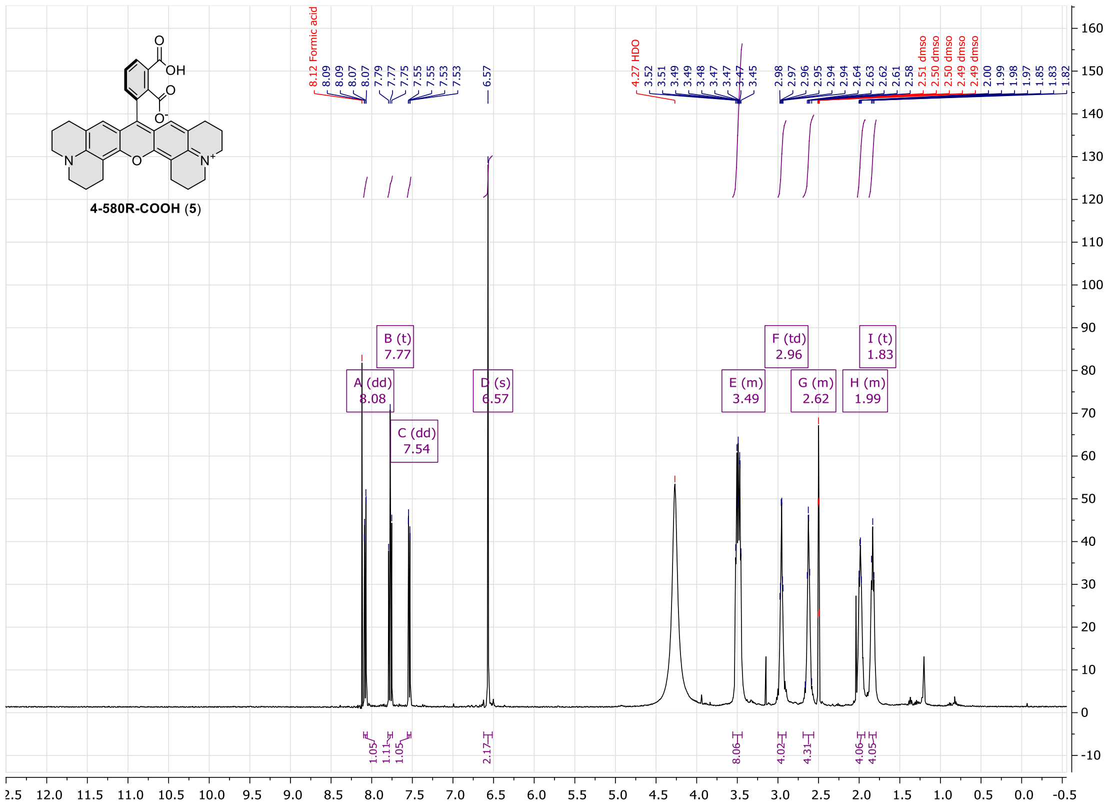

Supplementary Figure 201. ^{1}H NMR of compound **5** (400 MHz, d_6-DMSO+CF_3COOD).

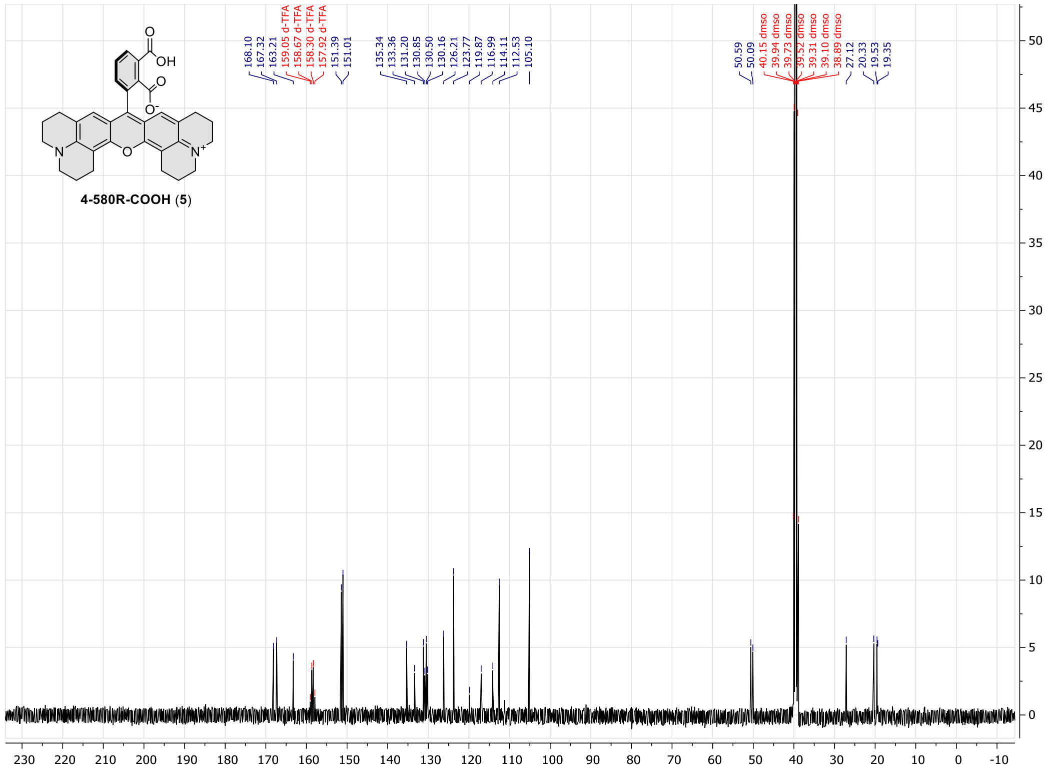

Supplementary Figure 202. ^{13}C NMR of compound **5** (100 MHz, d_6-DMSO+CF_3COOD).

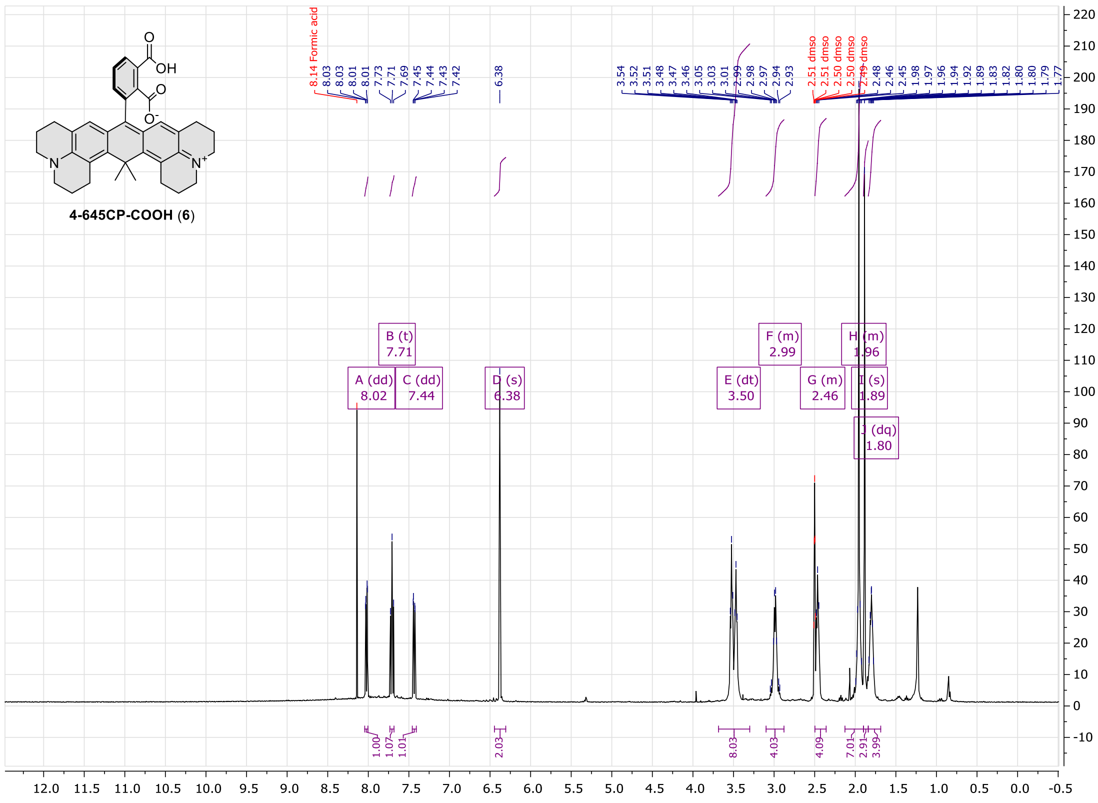

Supplementary Figure 203. ^{1}H NMR of compound **6** (400 MHz, d_6-DMSO+CF_3COOD).

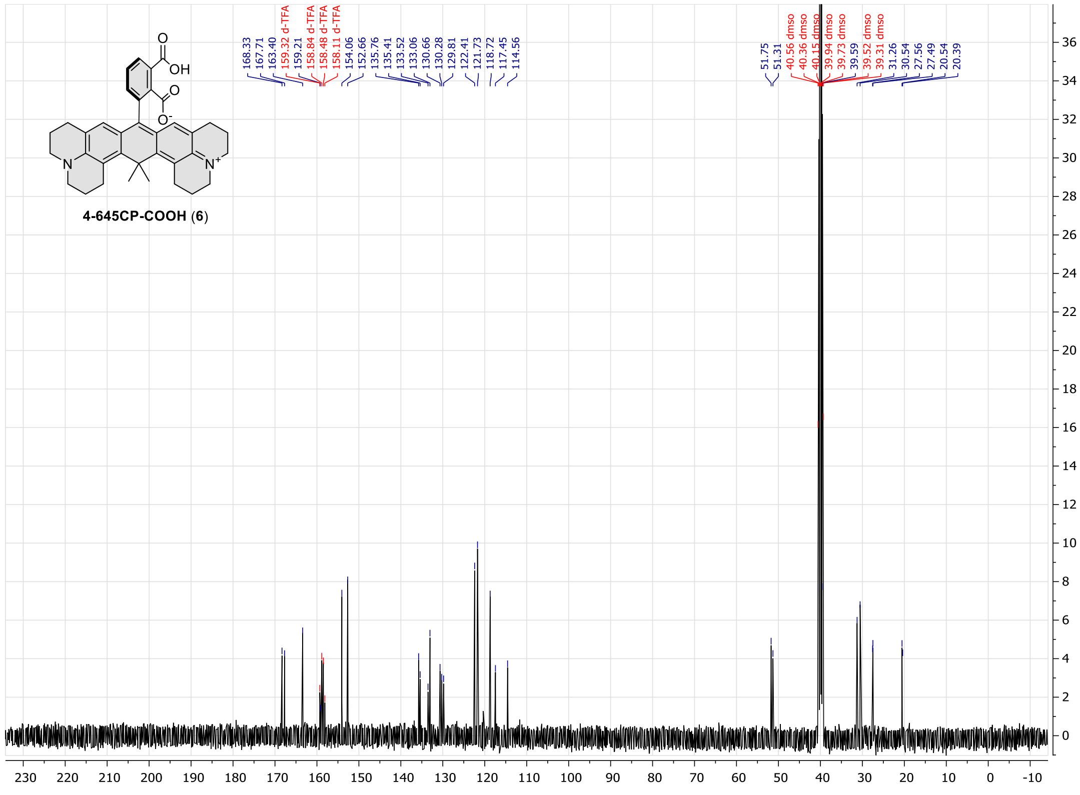

Supplementary Figure 204. ^{13}C NMR of compound **6** (100 MHz, d_6-DMSO+CF_3COOD).

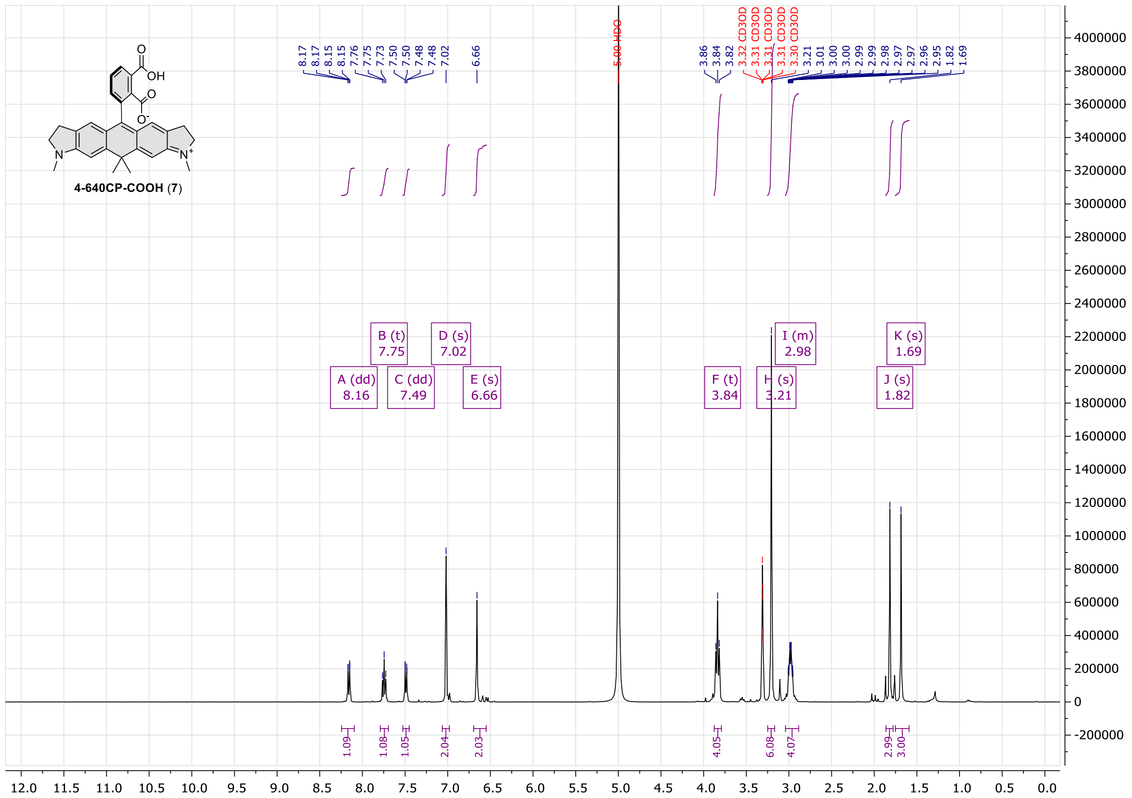

Supplementary Figure 205. ^{1}H NMR of compound **7** (400 MHz, CD_3OD+CF_3COOD).

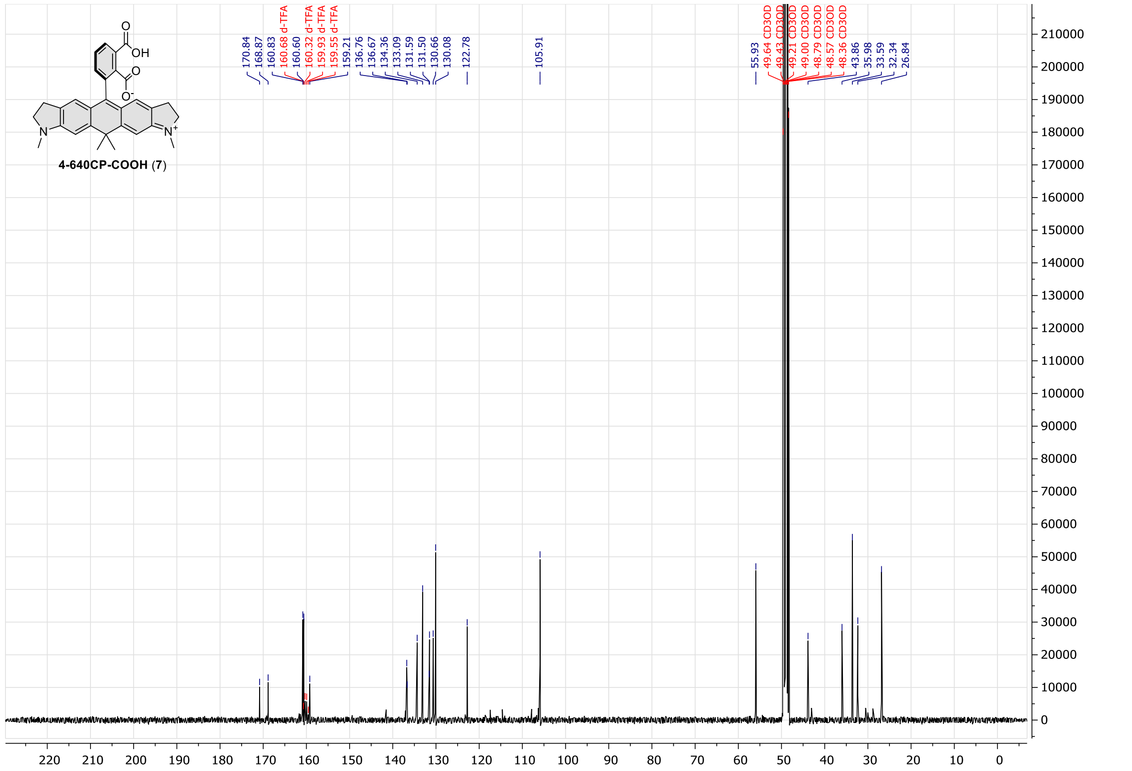

Supplementary Figure 206. ^{13}C NMR of compound **7** (100 MHz, CD_3OD+CF_3COOD).

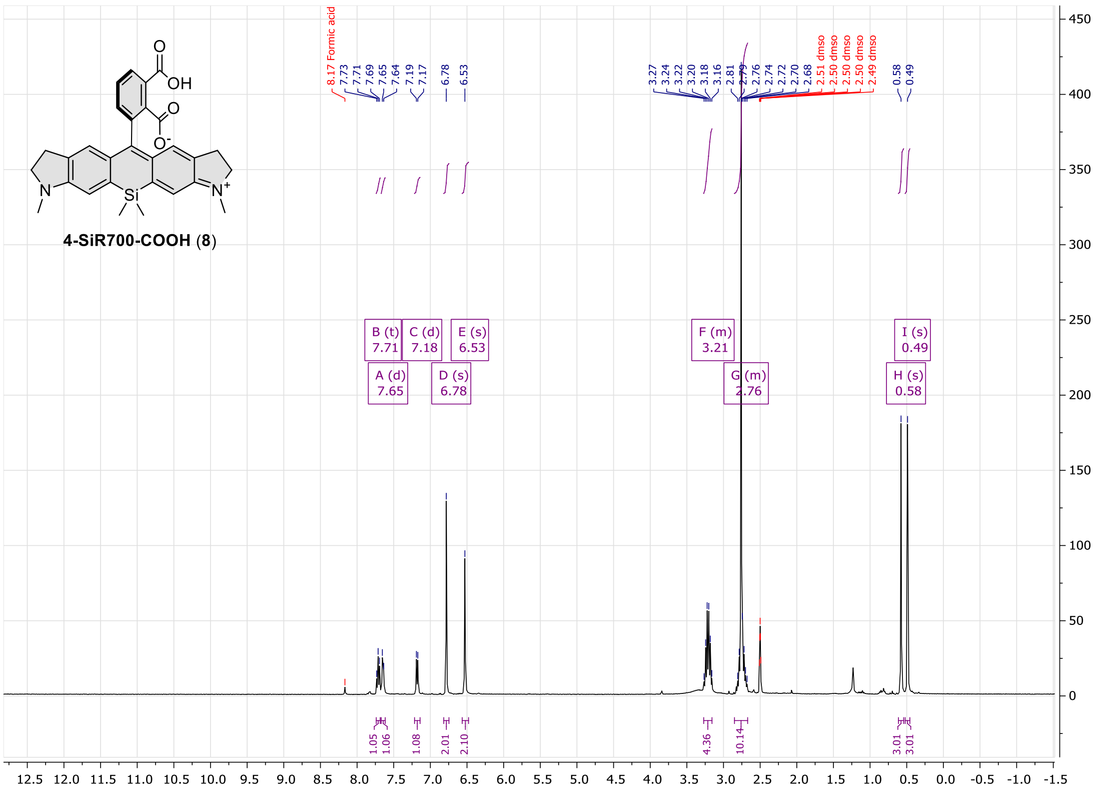

Supplementary Figure 207. ^{1}H NMR of compound **8** (400 MHz, d_6-DMSO).

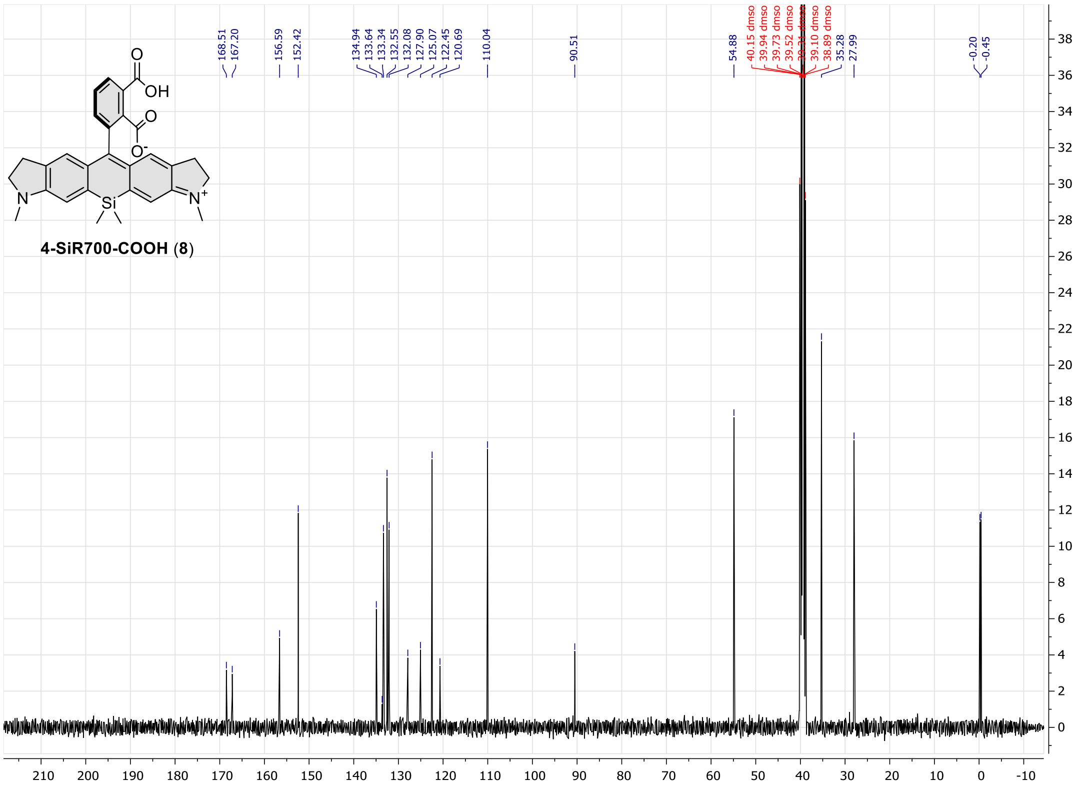

Supplementary Figure 208. ^{13}C NMR of compound **8** (100 MHz, d_6-DMSO).

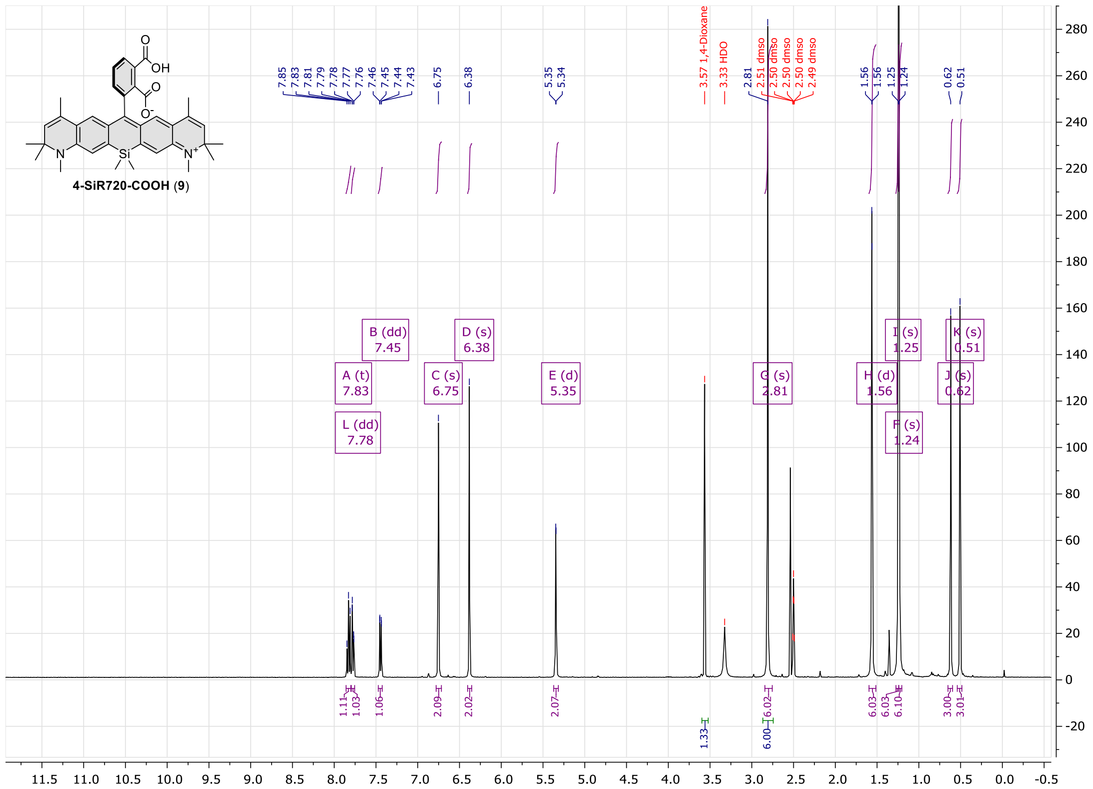

Supplementary Figure 209. ^{1}H NMR of compound **9** (400 MHz, d_6-DMSO).

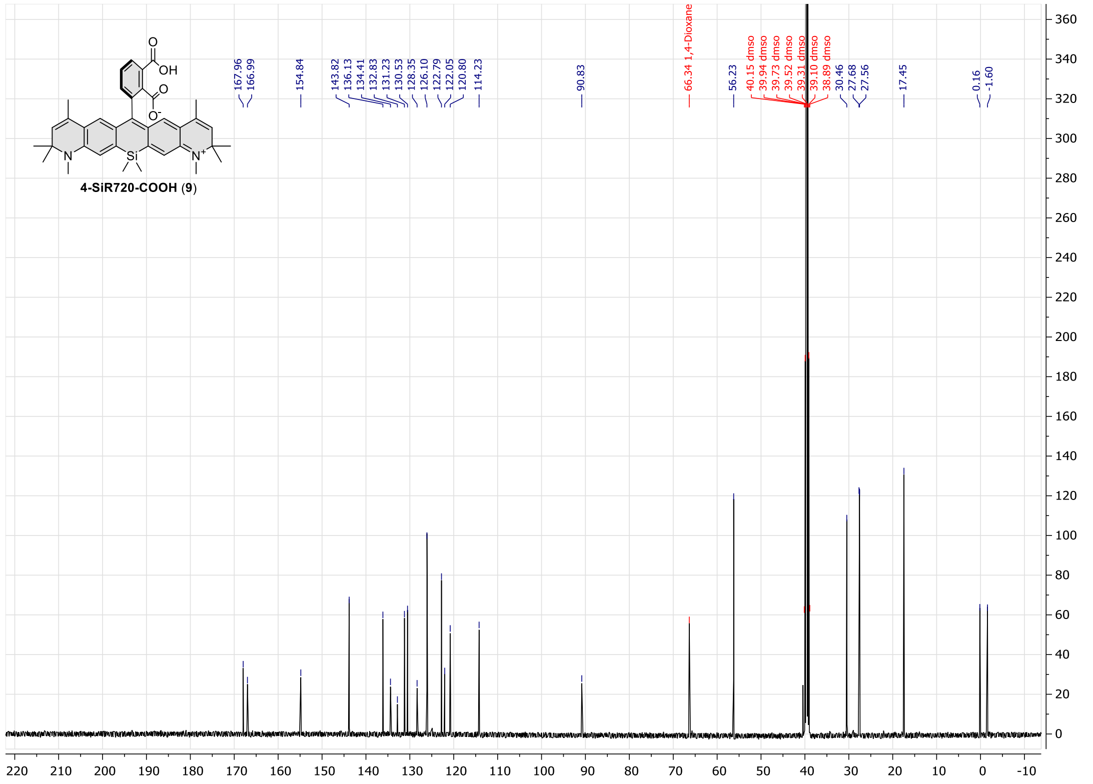

Supplementary Figure 210. ^{13}C NMR of compound **9** (100 MHz, d_6-DMSO).

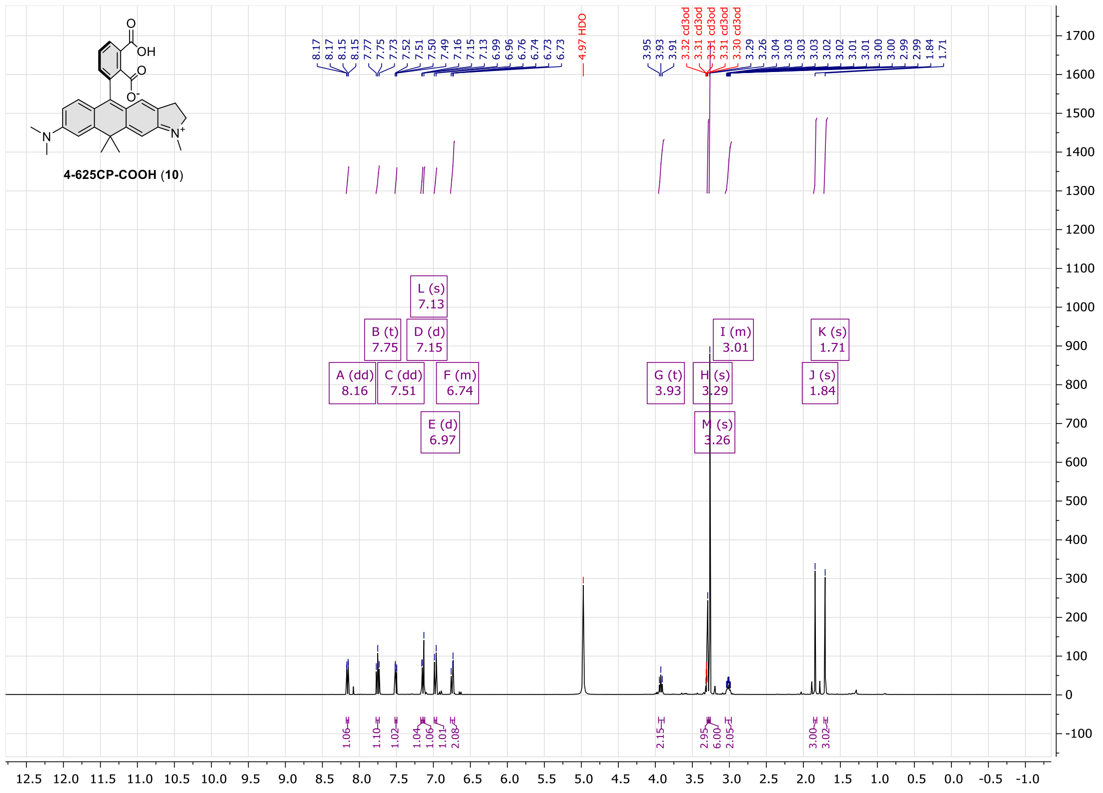

Supplementary Figure 211. ^{1}H NMR of compound **10** (100 MHz, CD_3OD).

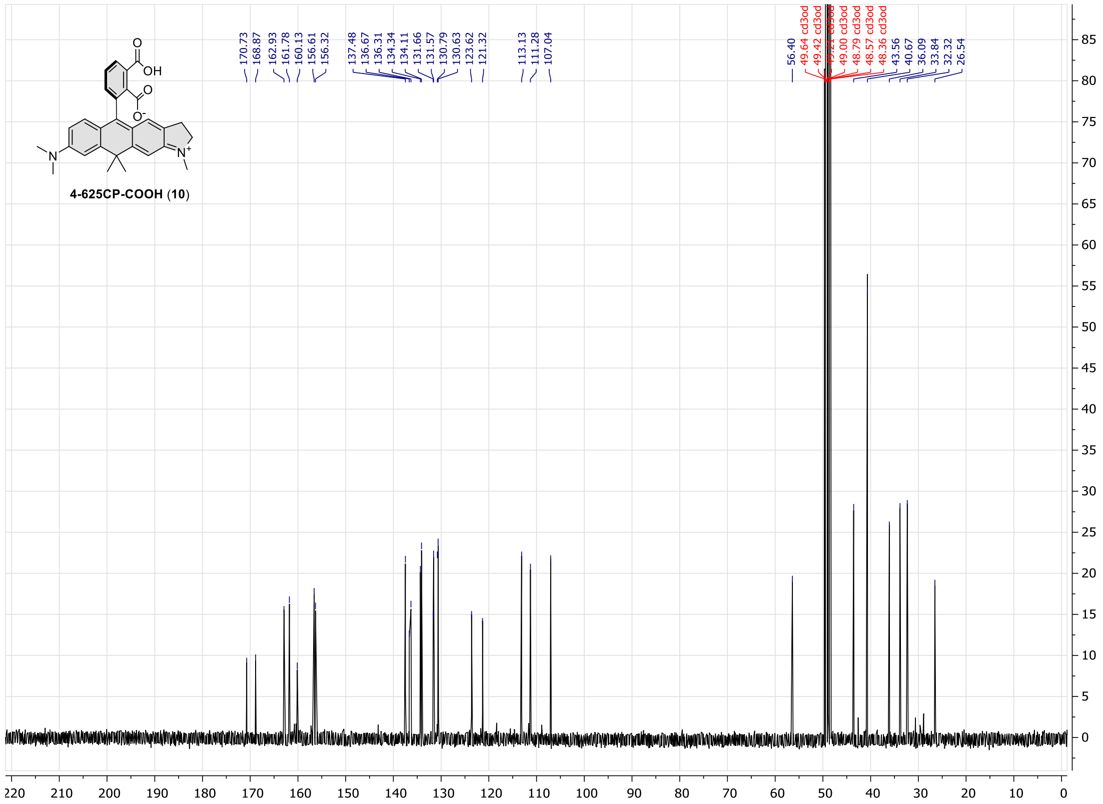

Supplementary Figure 212. ^{13}C NMR of compound **10** (100 MHz, CD_3OD).

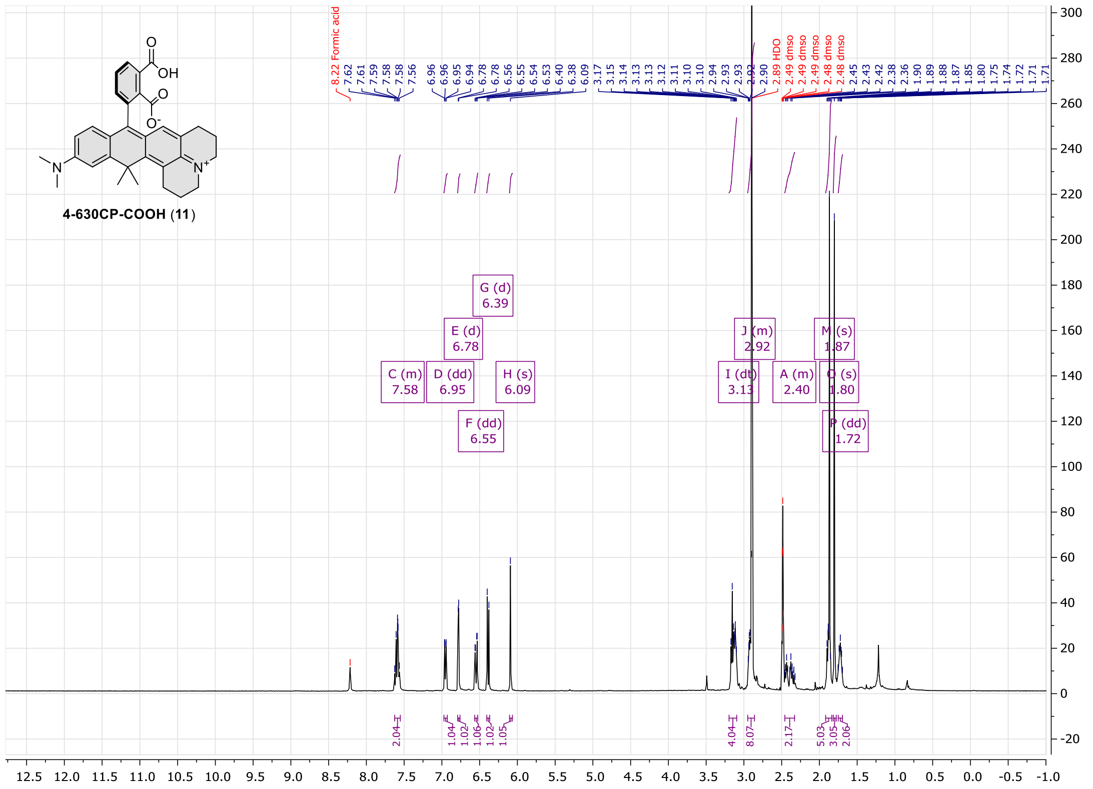

Supplementary Figure 213. ^{1}H NMR of compound **11** (400 MHz, d_6-DMSO).

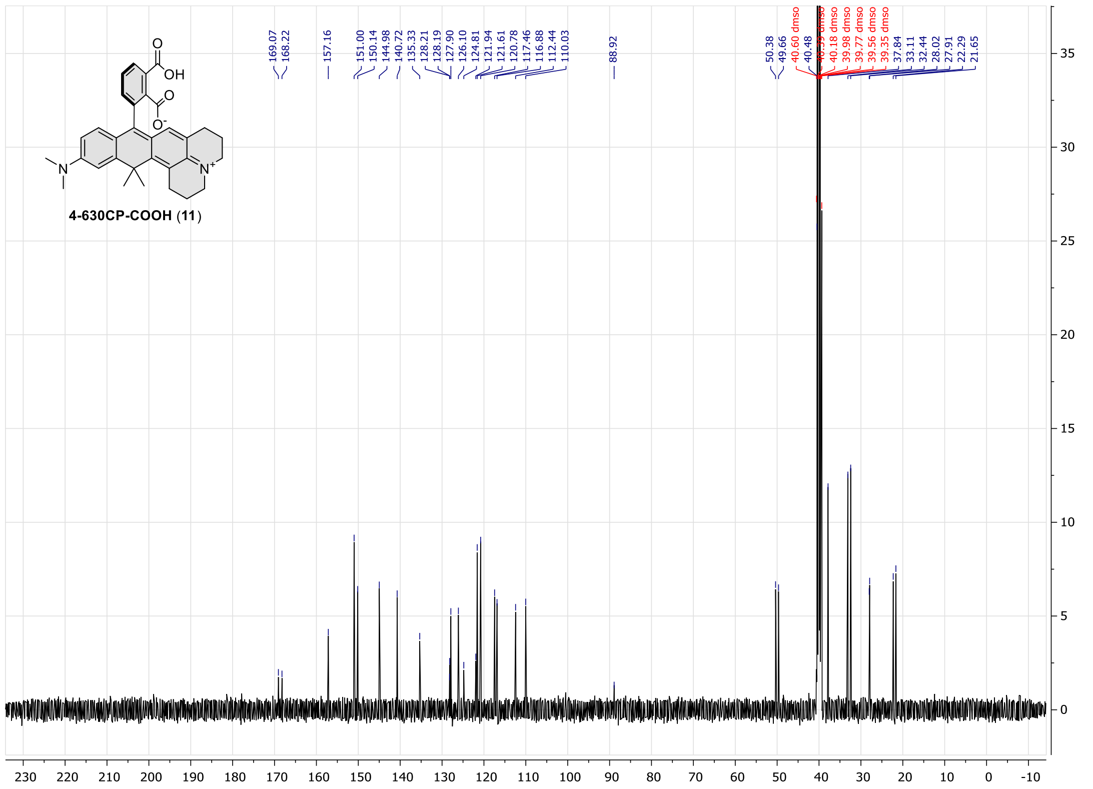

Supplementary Figure 214. ^{13}C NMR of compound **11** (100 MHz, d_6-DMSO).

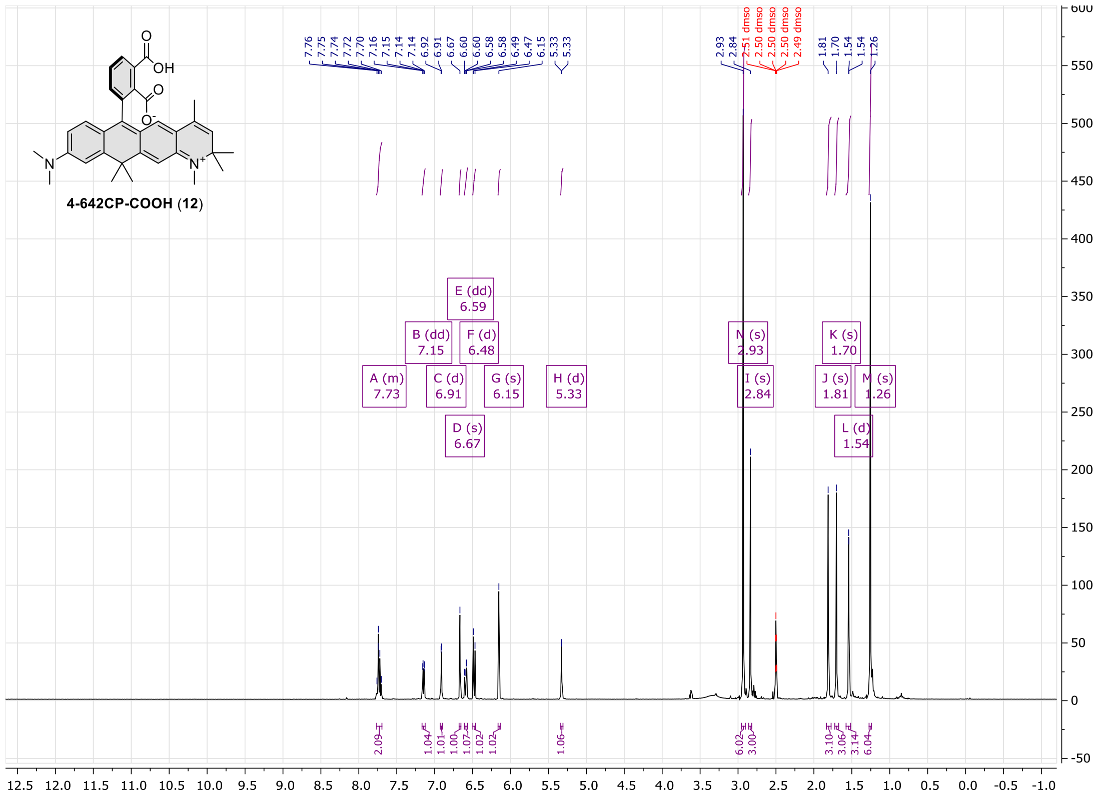

Supplementary Figure 215. ^{1}H NMR of compound **12** (400 MHz, d_6-DMSO).

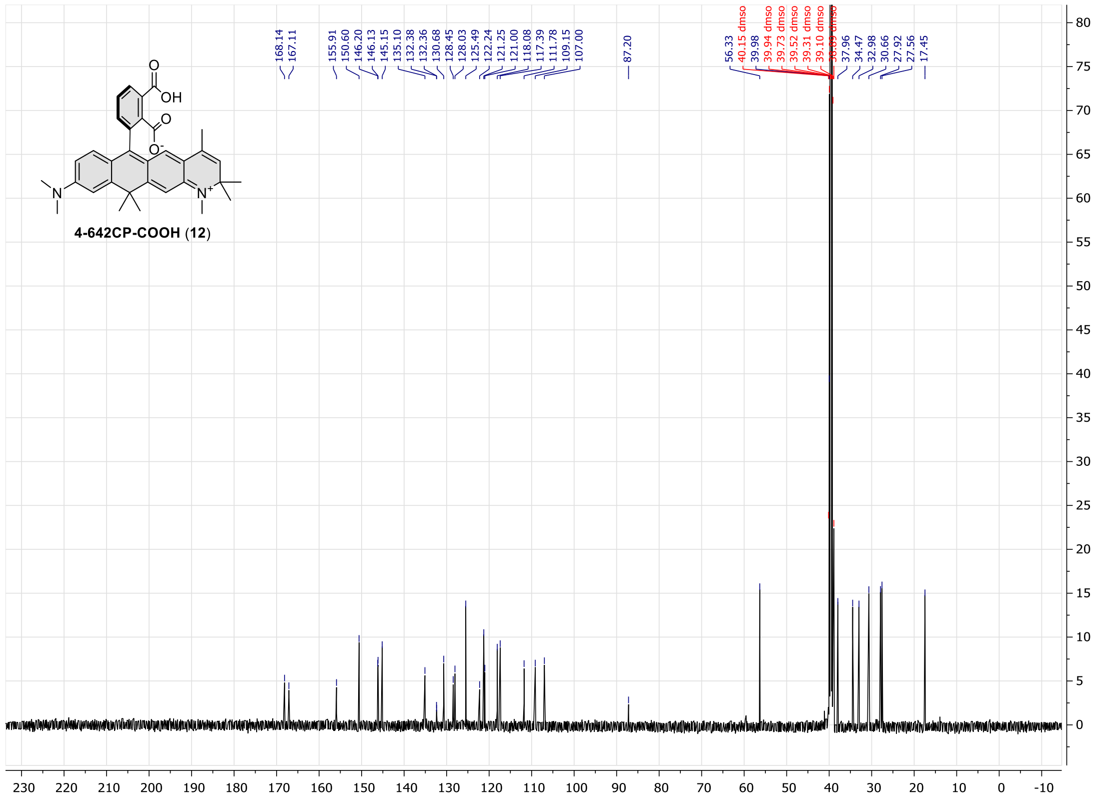

Supplementary Figure 216. ^{13}C NMR of compound **12** (100 MHz, d_6-DMSO).

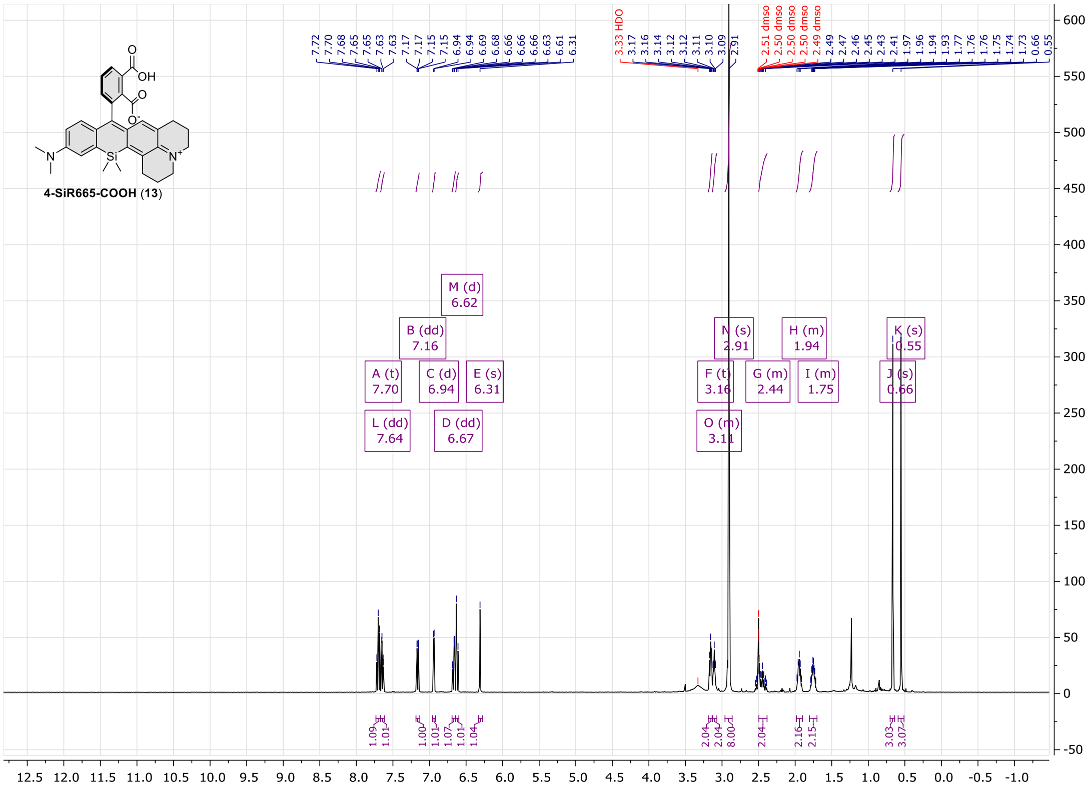

Supplementary Figure 217. ^{1}H NMR of compound **13** (400 MHz, d_6-DMSO).

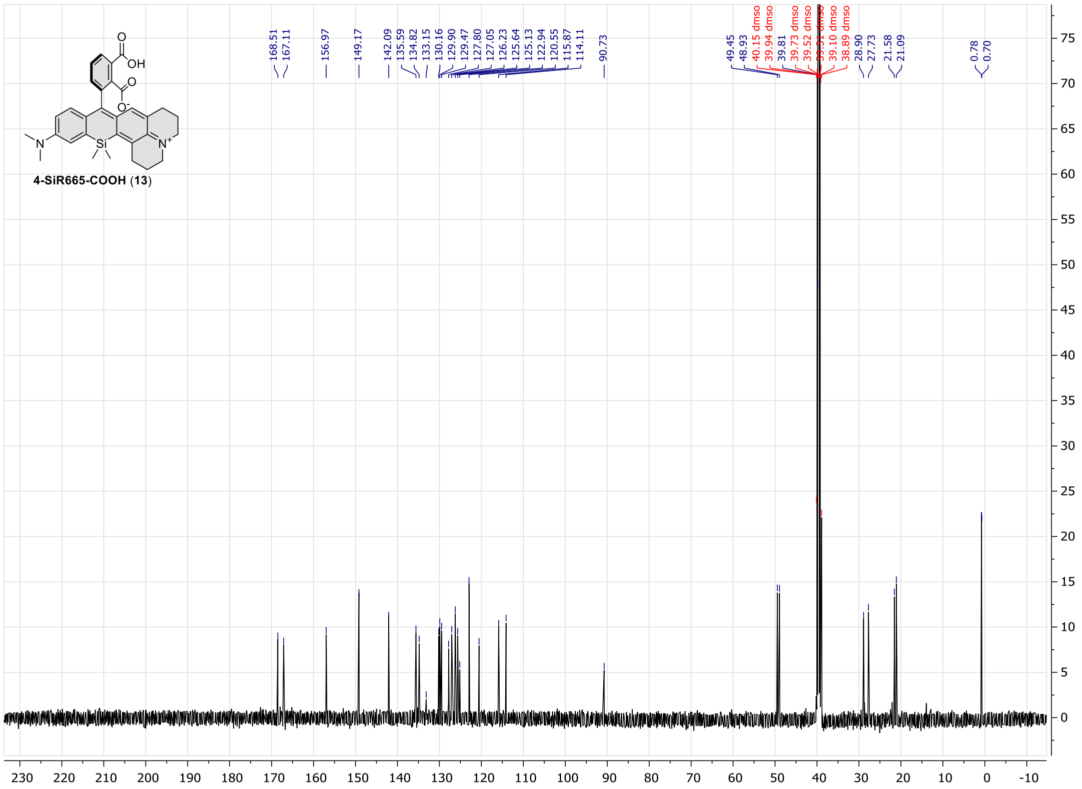

Supplementary Figure 218. ^{13}C NMR of compound **13** (100 MHz, d_6-DMSO).

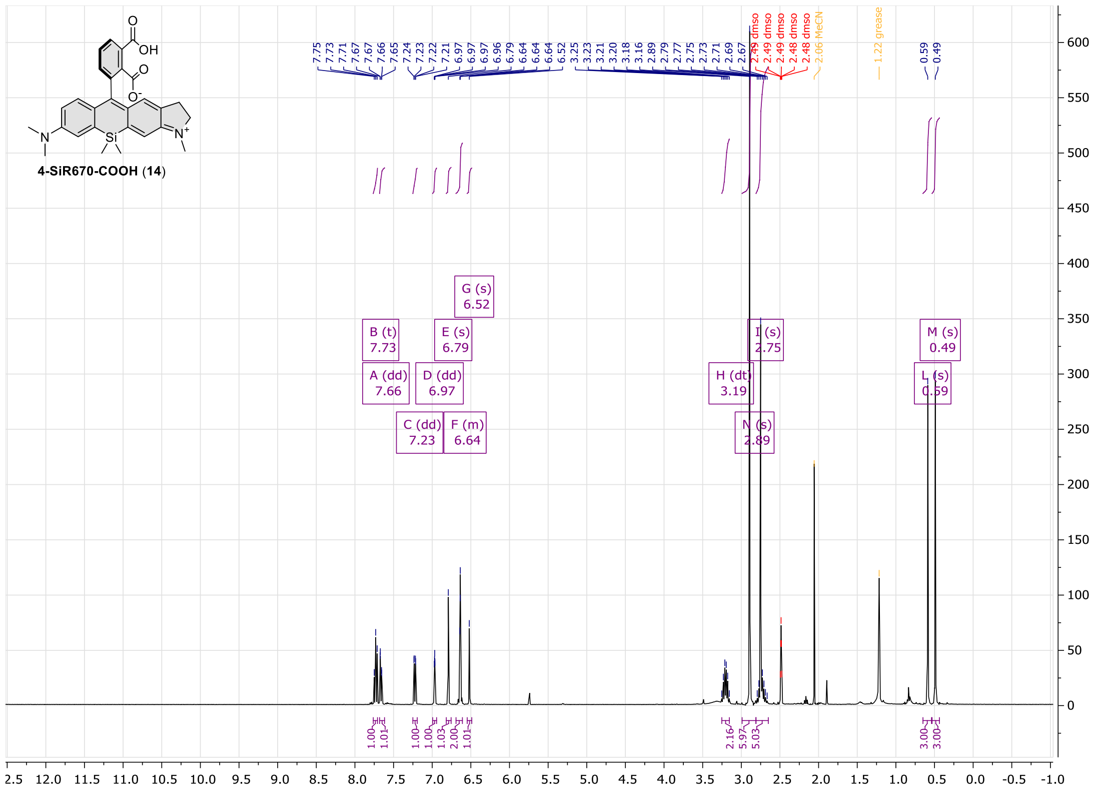

Supplementary Figure 219. ^{1}H NMR of compound **14** (400 MHz, d_6-DMSO).

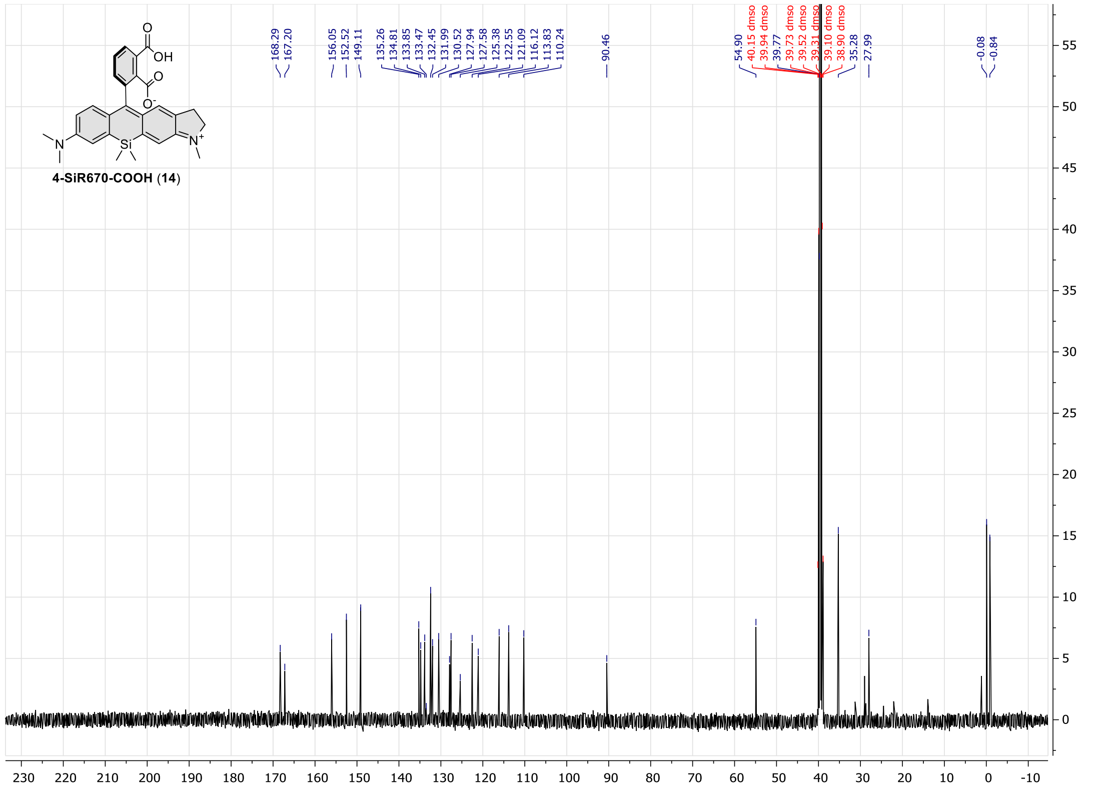

Supplementary Figure 220. ^{13}C NMR of compound **14** (100 MHz, d_6-DMSO).

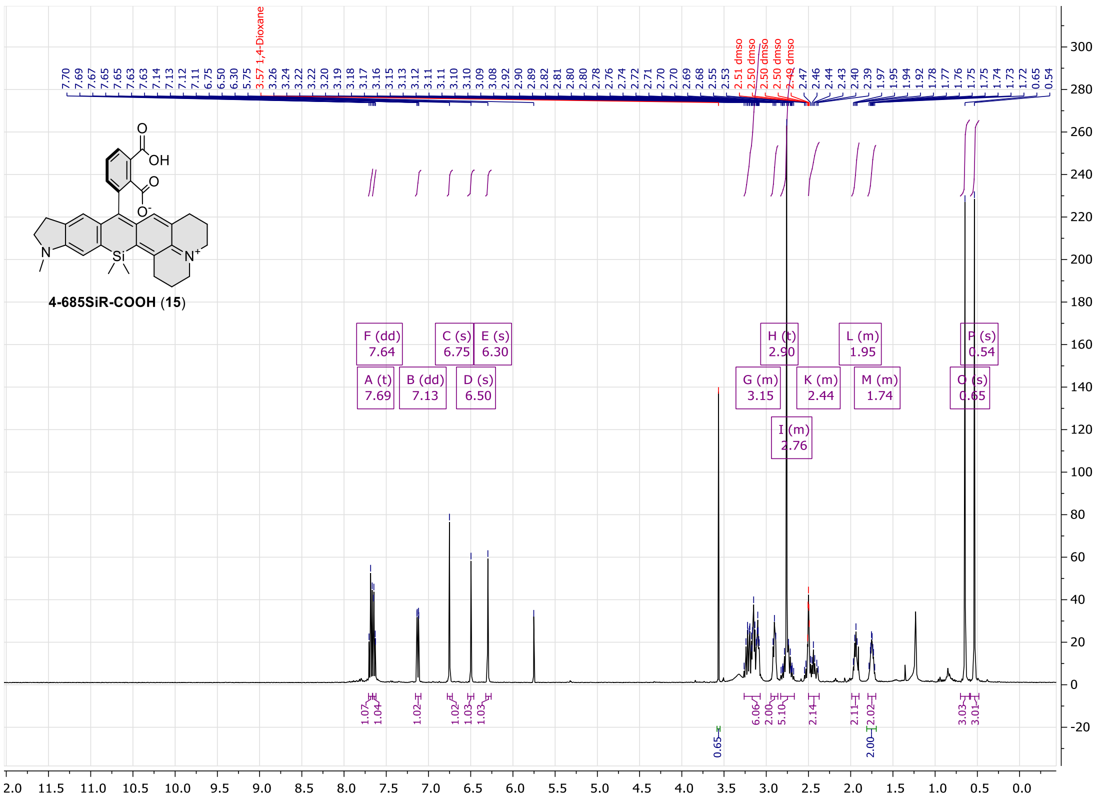

Supplementary Figure 221. ^{1}H NMR of compound **15** (400 MHz, d_6-DMSO).

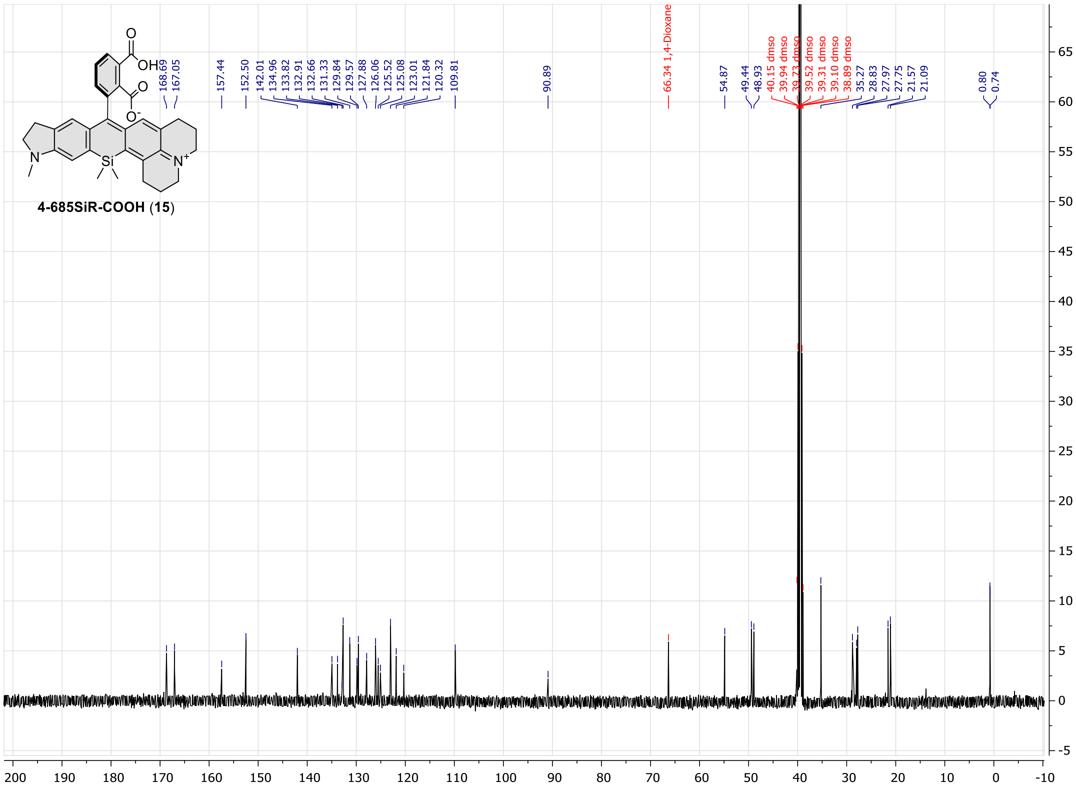

Supplementary Figure 222. ^{13}C NMR of compound **16** (100 MHz, d_6-DMSO).

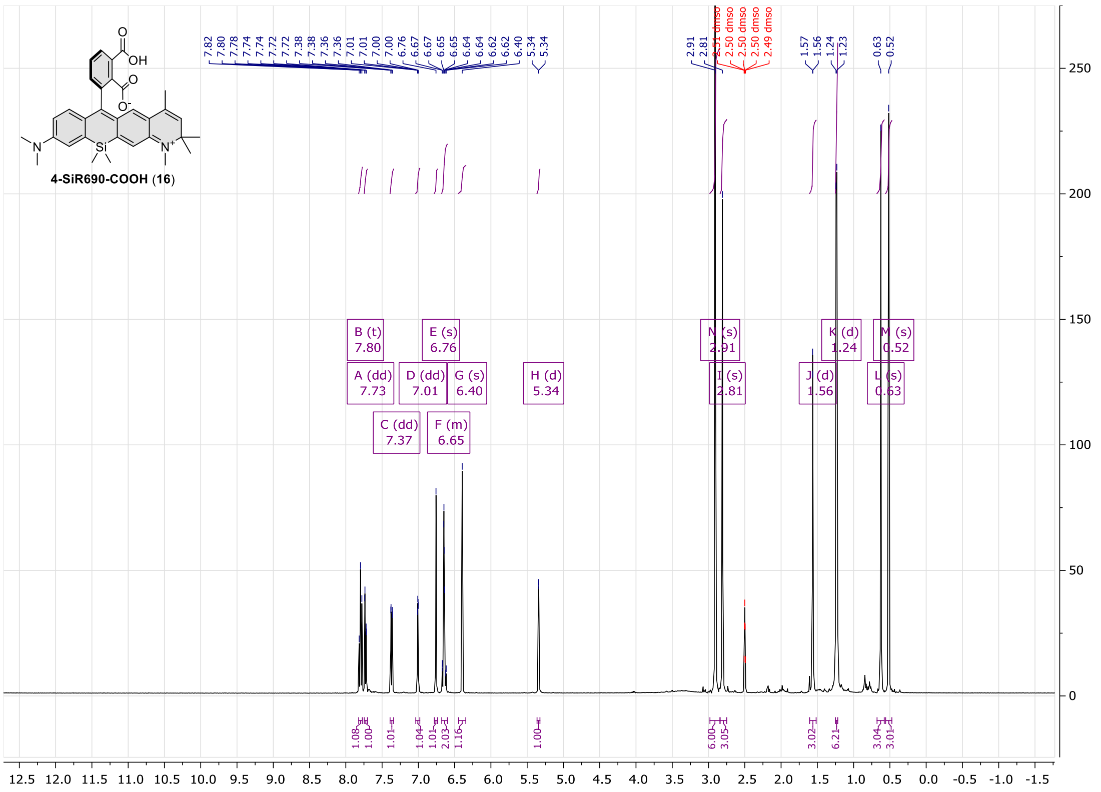

Supplementary Figure 223. ^{1}H NMR of compound **16** (400 MHz, d_6-DMSO).

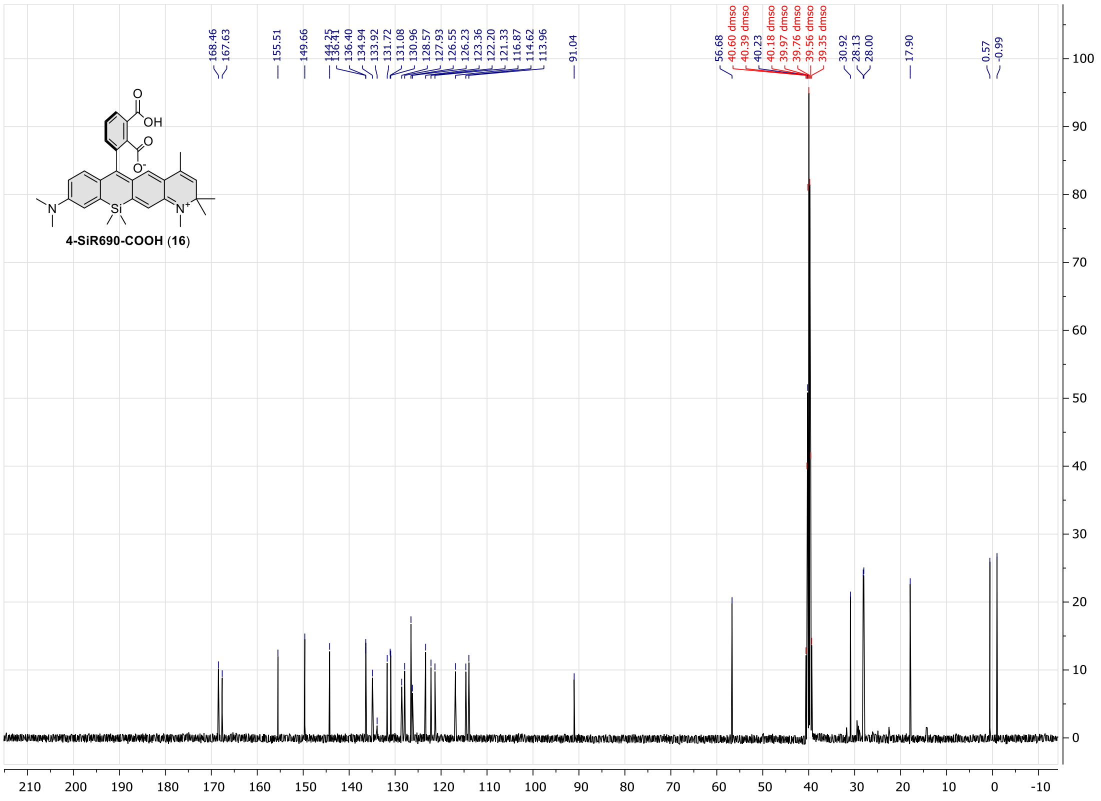

Supplementary Figure 224. ^{13}C NMR of compound **16** (100 MHz, d_6-DMSO).

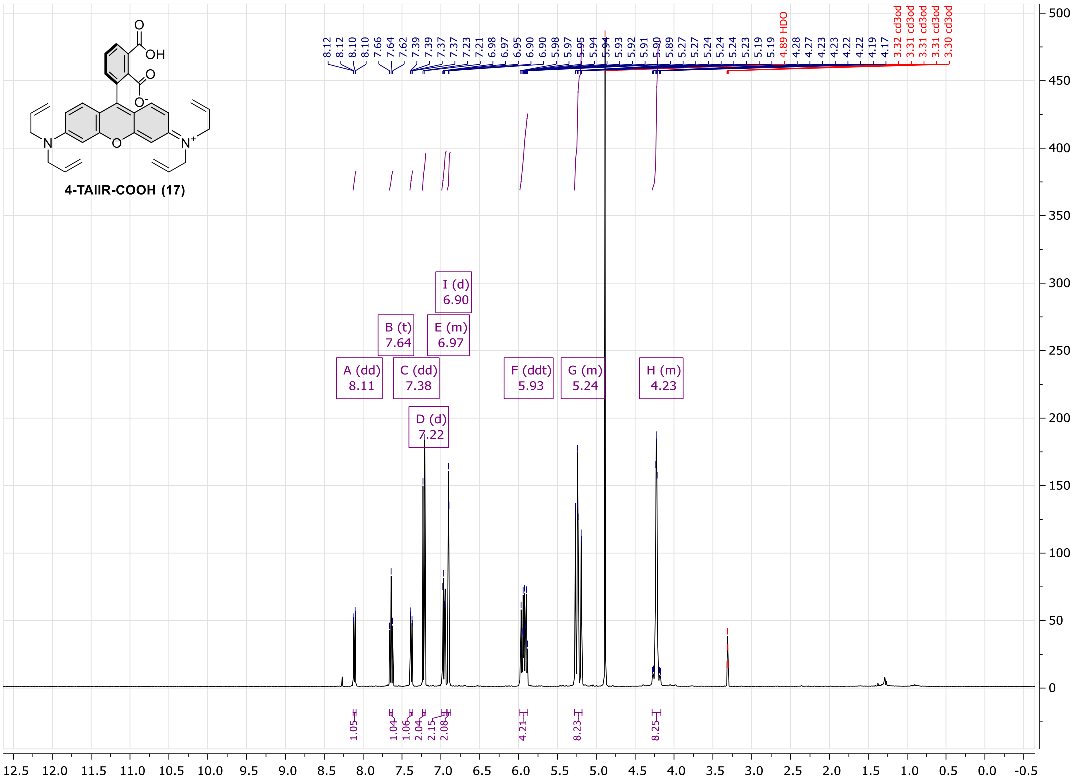

Supplementary Figure 225. ^{1}H NMR of compound **17** (400 MHz, CD_3OD).

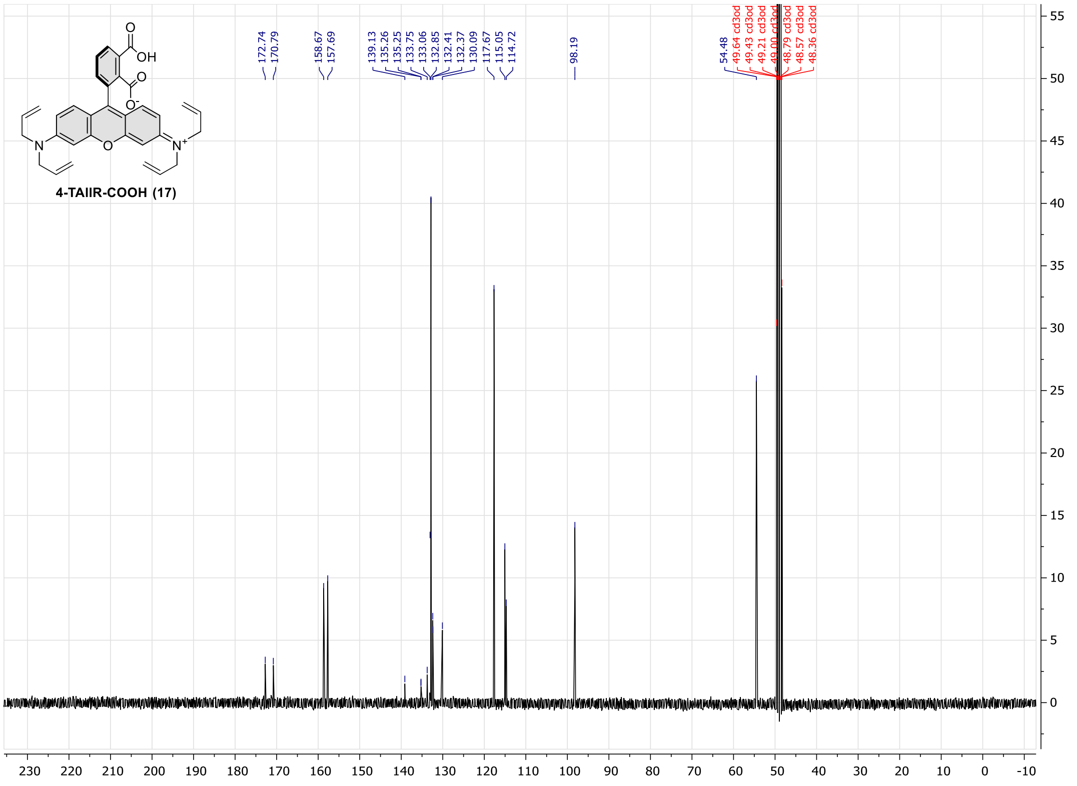

Supplementary Figure 226. ^{13}C NMR of compound **17** (100 MHz, CD_3OD).

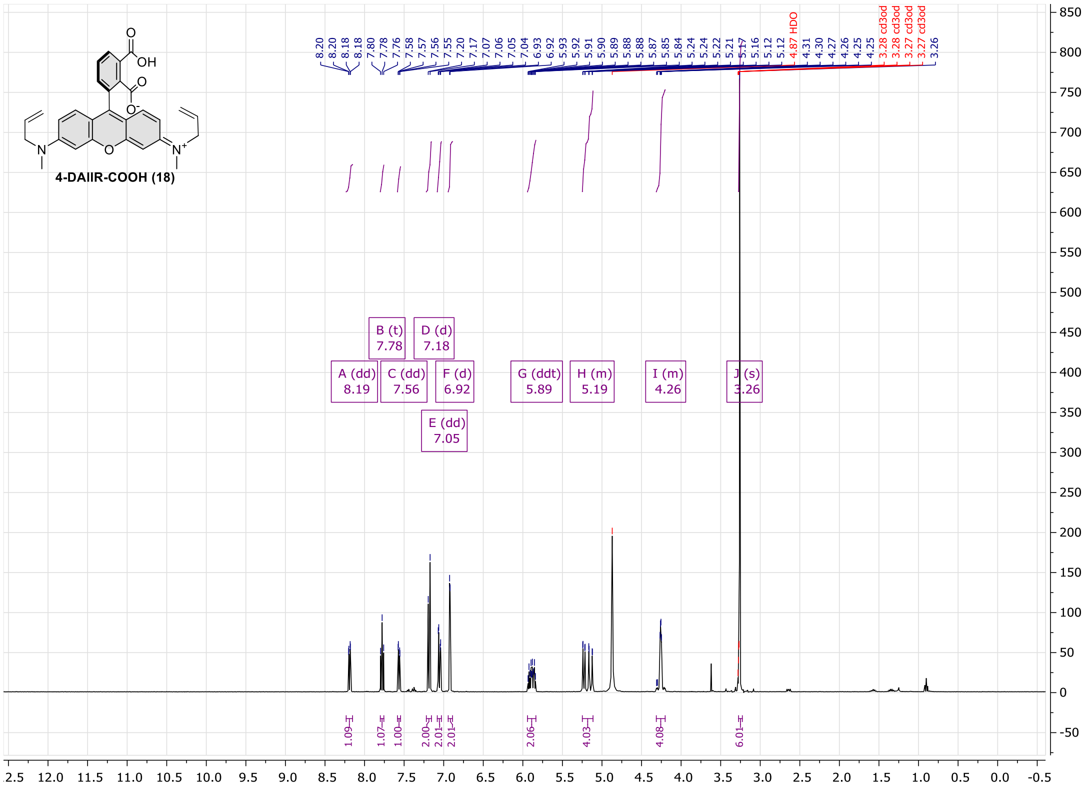

Supplementary Figure 227. ^{1}H NMR of compound **18** (400 MHz, CD_3OD).

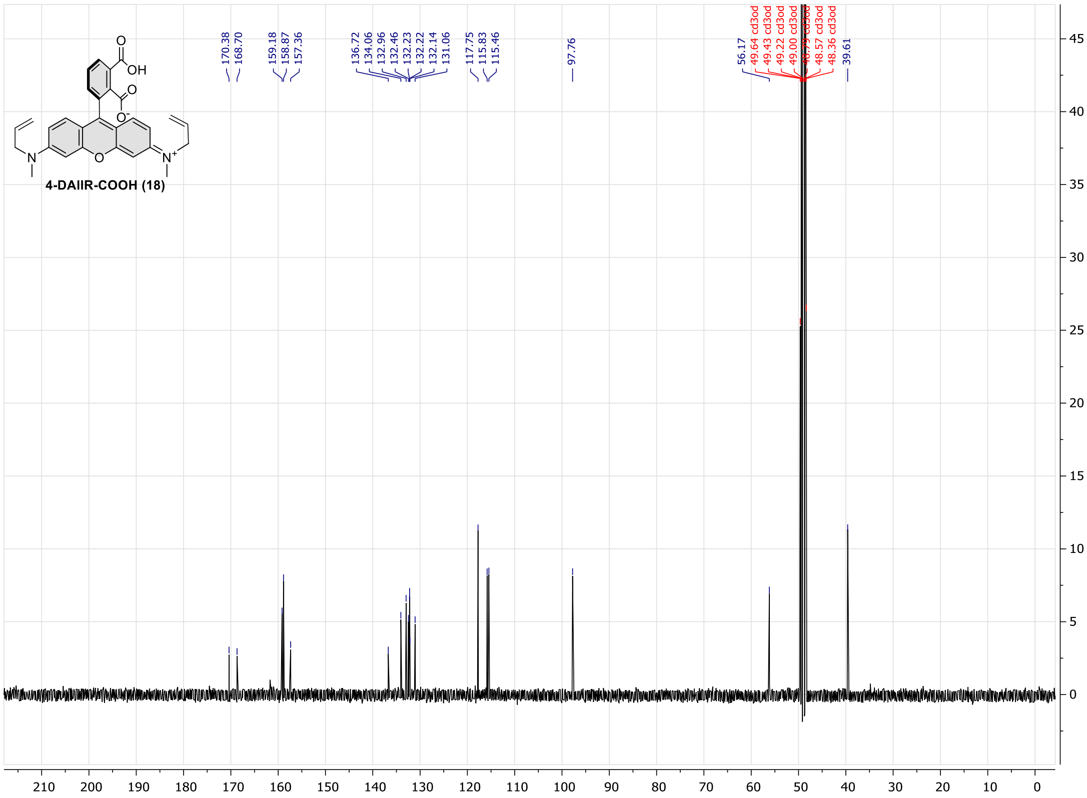

Supplementary Figure 228. ^{13}C NMR of compound **18** (100 MHz, CD_3OD).

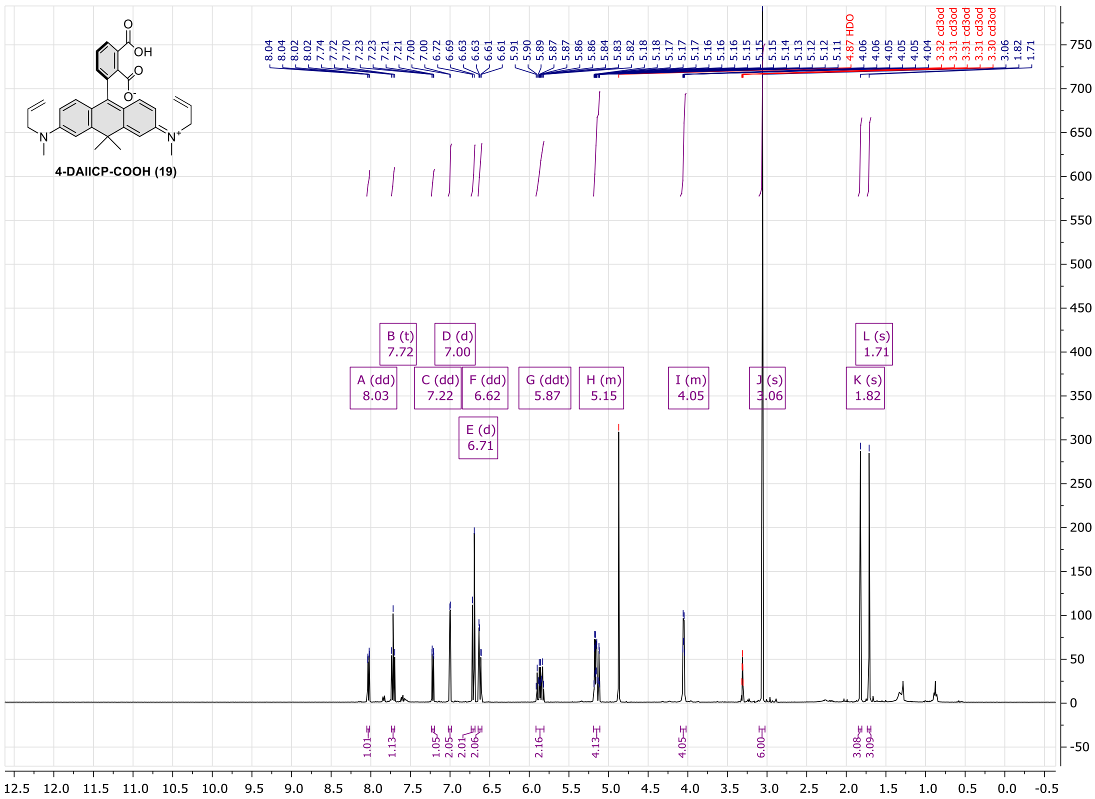

Supplementary Figure 229. ^{13}C NMR of compound **19** (400 MHz, CD_3OD).

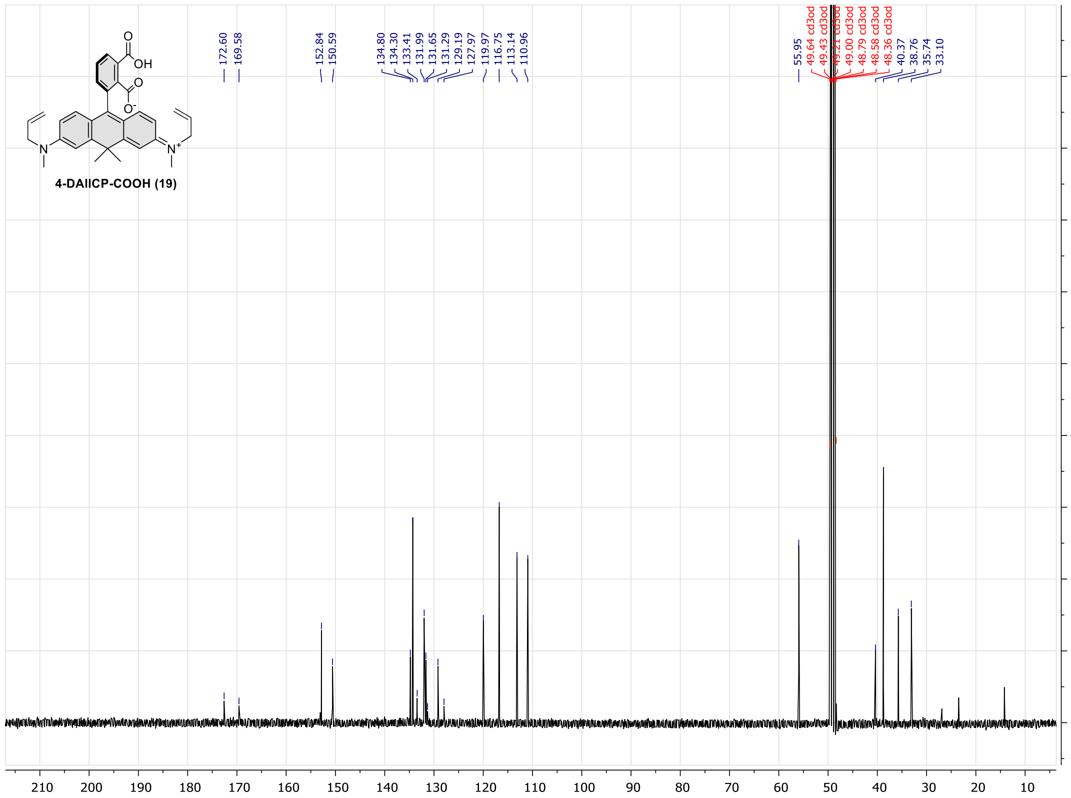

Supplementary Figure 230. ^{13}C NMR of compound **19** (100 MHz, CD_3OD).

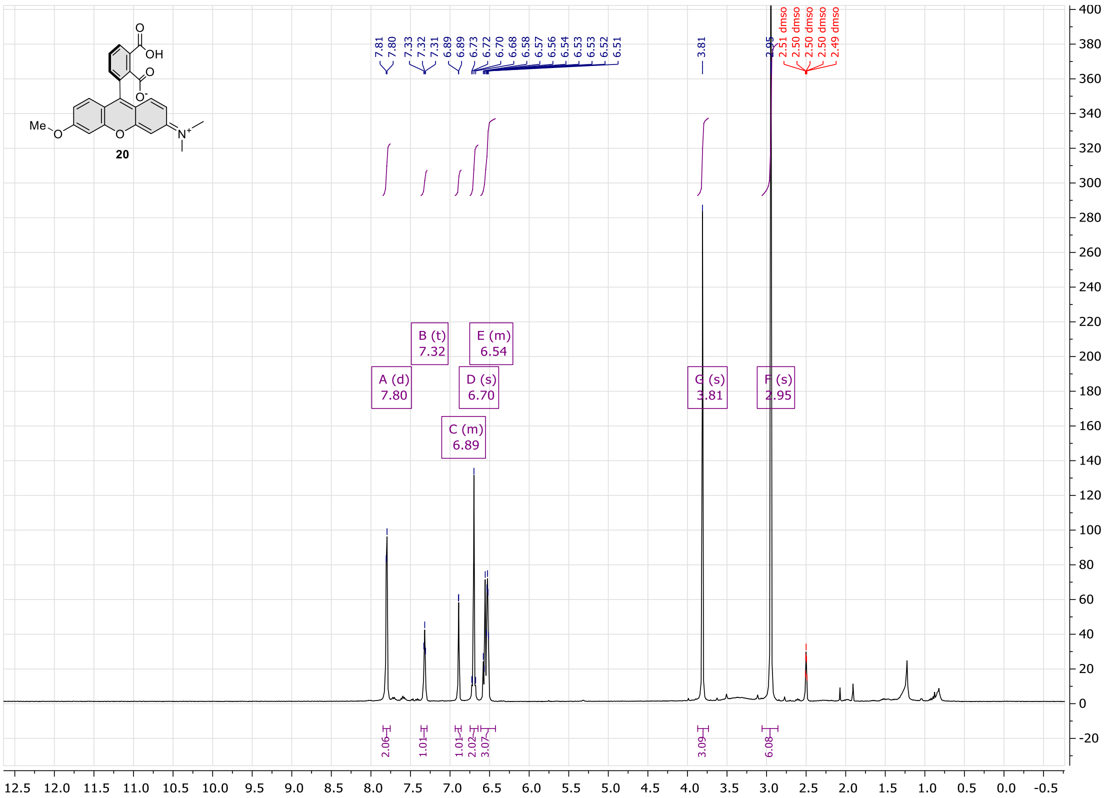

Supplementary Figure 231. ^{13}C NMR of compound **20** (100 MHz, d_6-DMSO).

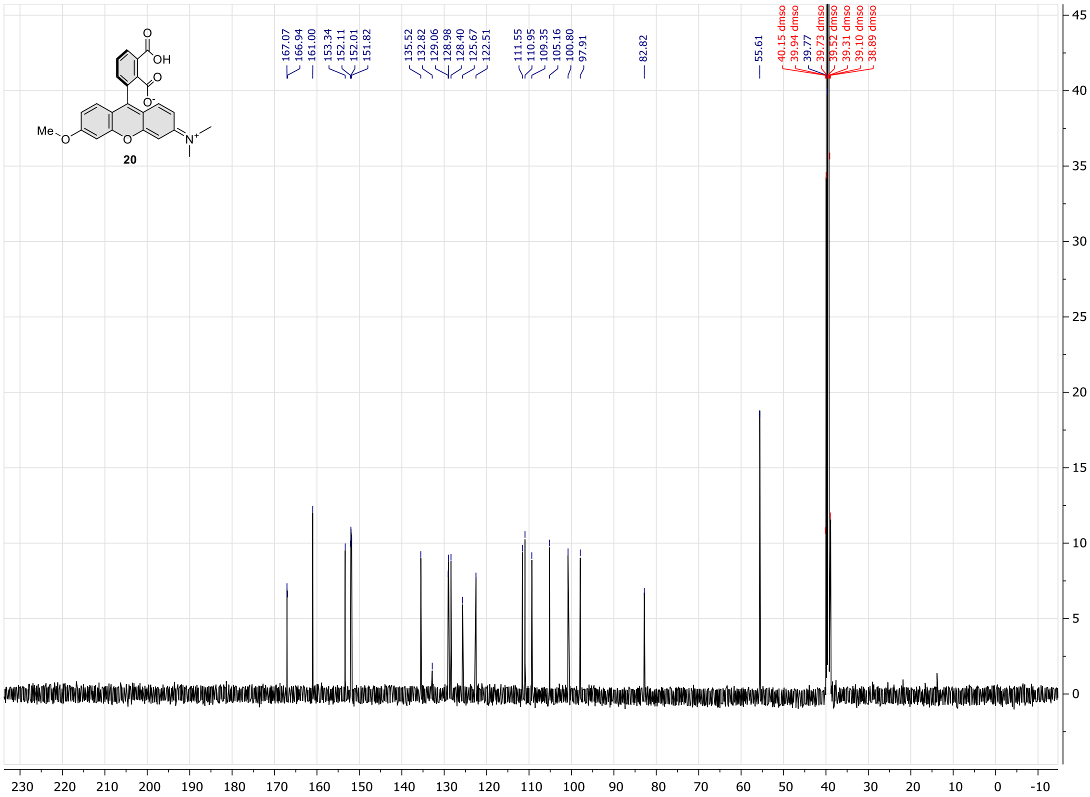

Supplementary Figure 232. ^{13}C NMR of compound **20** (100 MHz, d_6-DMSO).

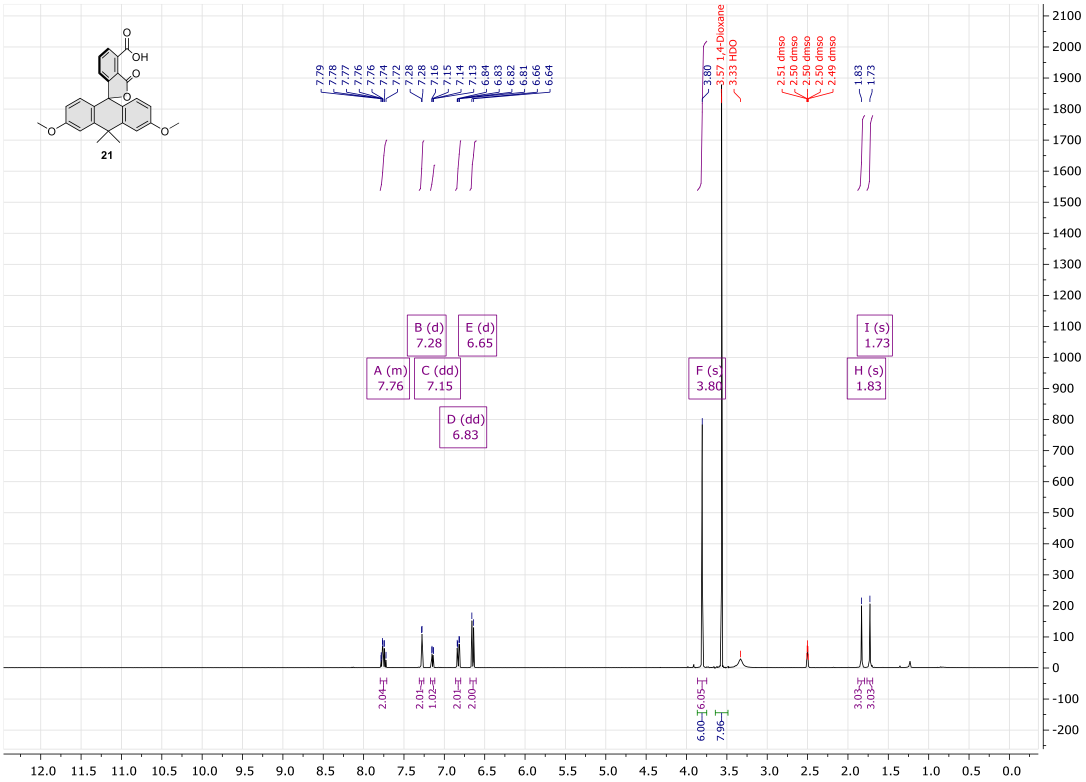

Supplementary Figure 233. ^{1}H NMR of compound **21** (400 MHz, d_6-DMSO).

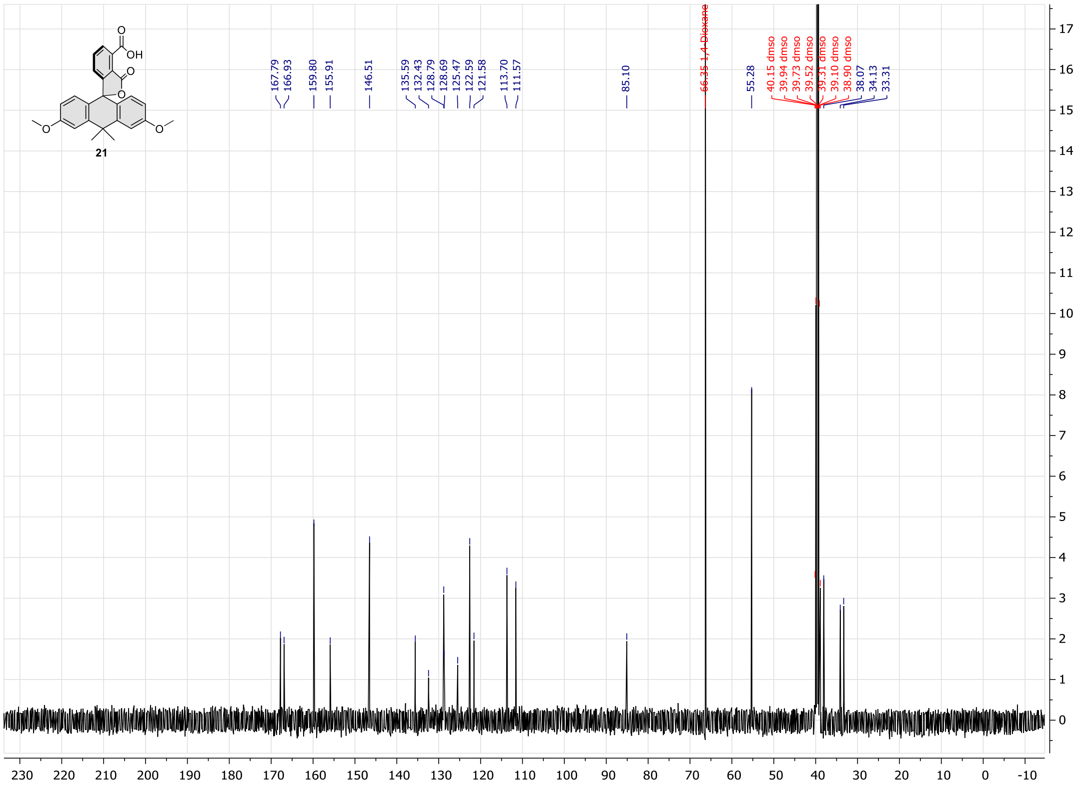

Supplementary Figure 234. ^{13}C NMR of compound **21** (100 MHz, d_6-DMSO).

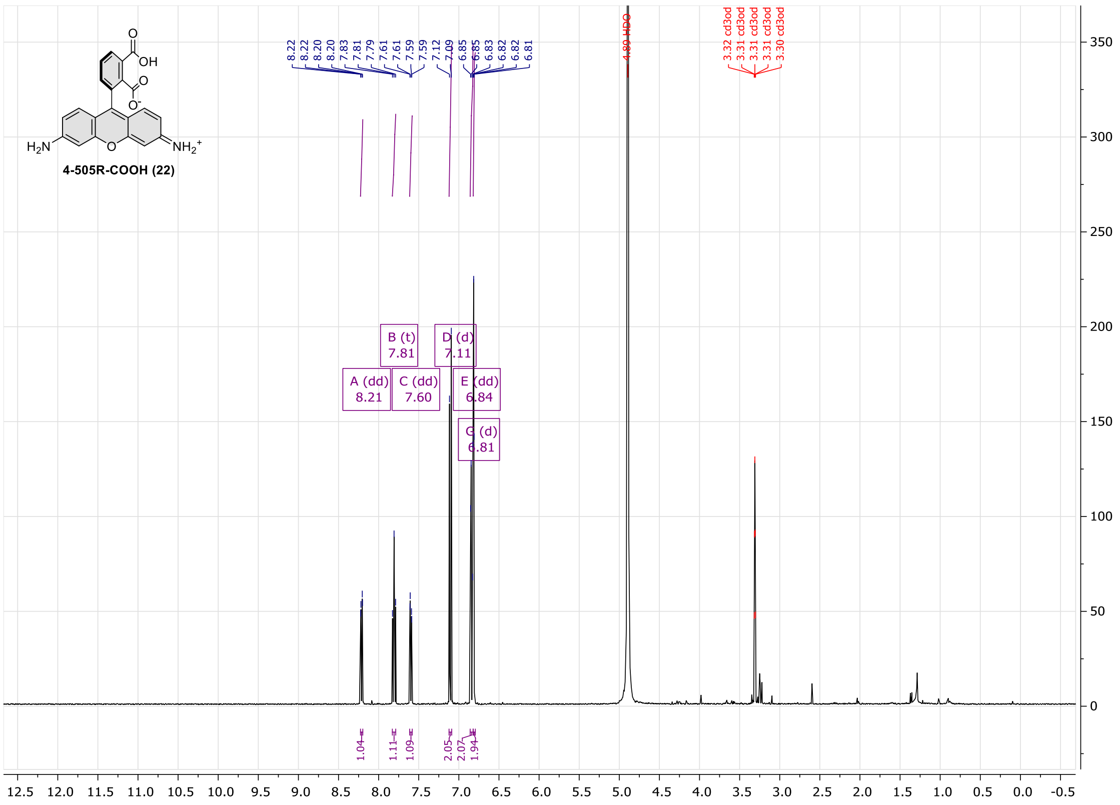

Supplementary Figure 235. ^{1}H NMR of compound **22** (400 MHz, CD_3OD).

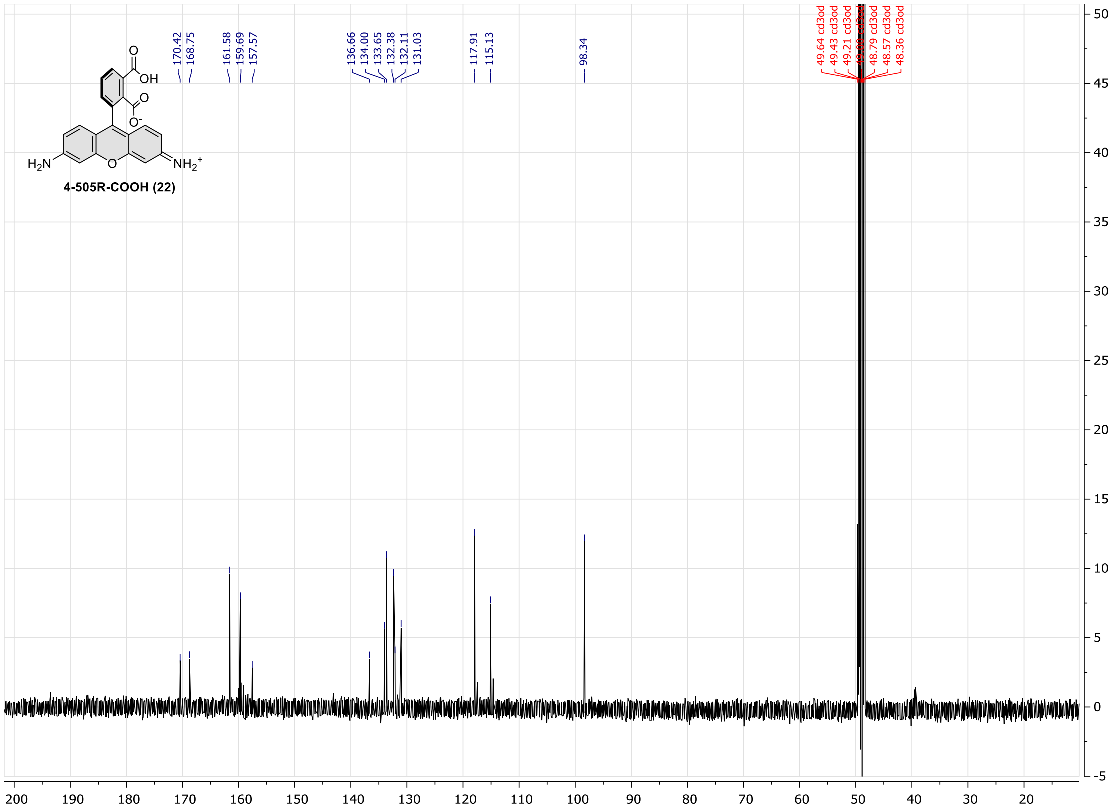

Supplementary Figure 236. ^{13}C NMR of compound **22** (100 MHz, CD_3OD).

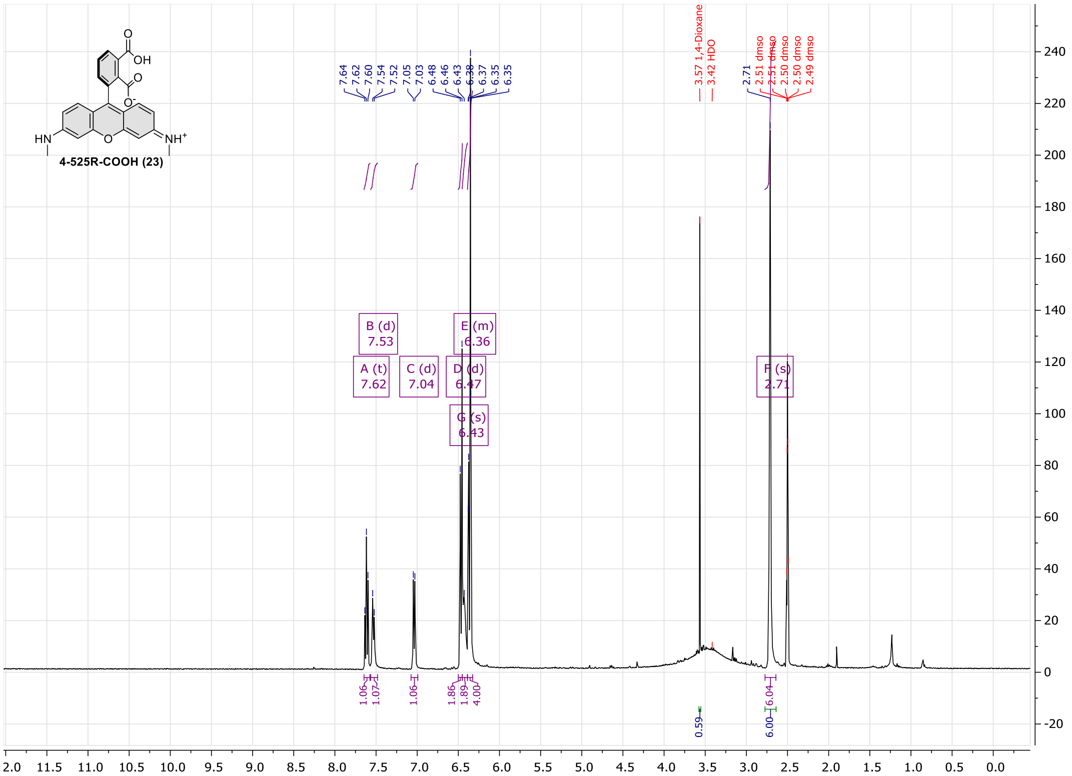

Supplementary Figure 237. ^{1}H NMR of compound **23** (400 MHz, d_6-DMSO).

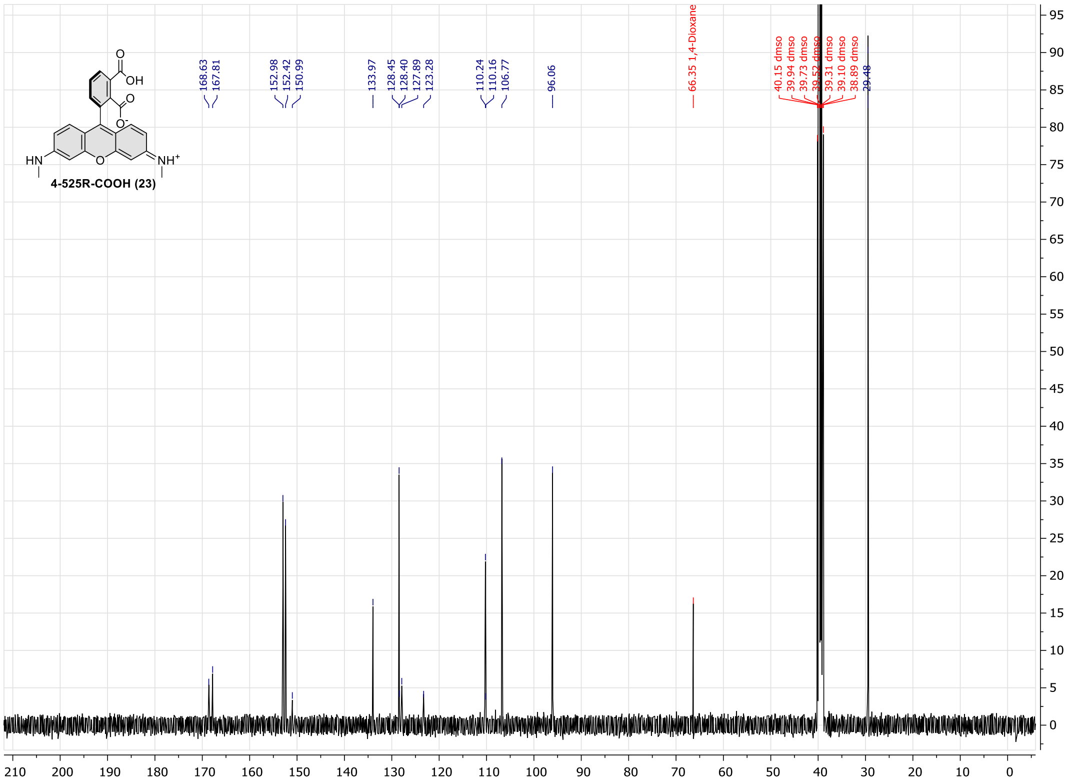

Supplementary Figure 238. ^{13}C NMR of compound **23** (100 MHz, d_6-DMSO).

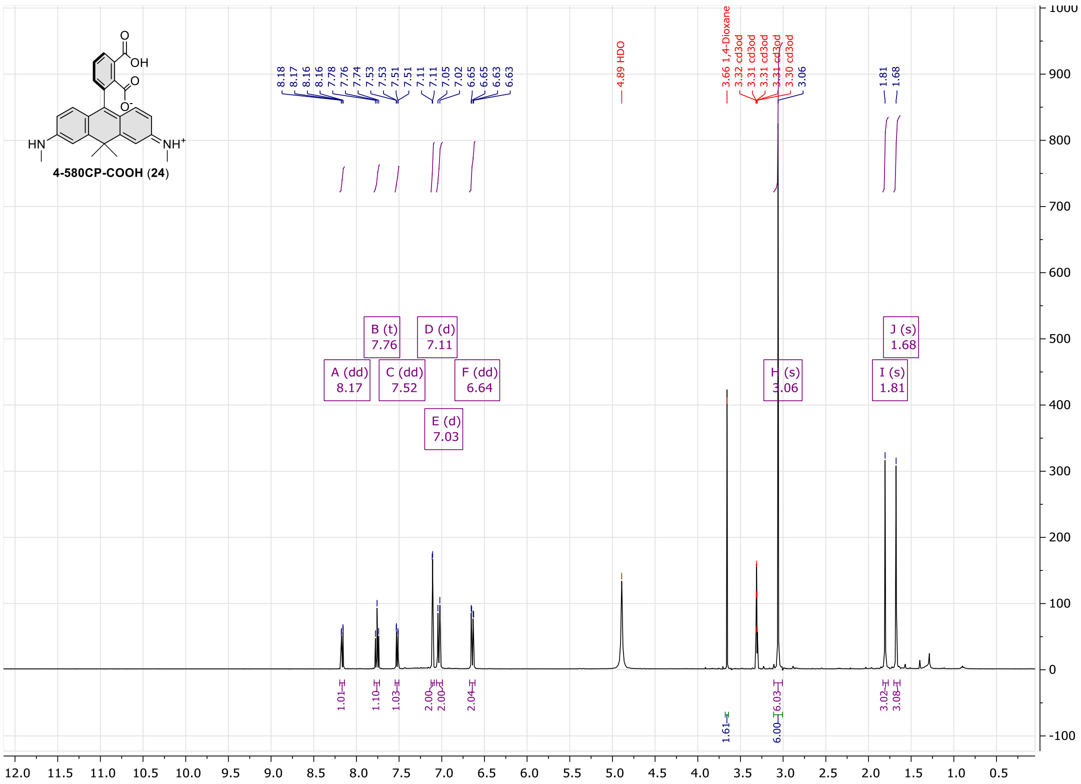

Supplementary Figure 239. ^{1}H NMR of compound **24** (400 MHz, CD_3OD).

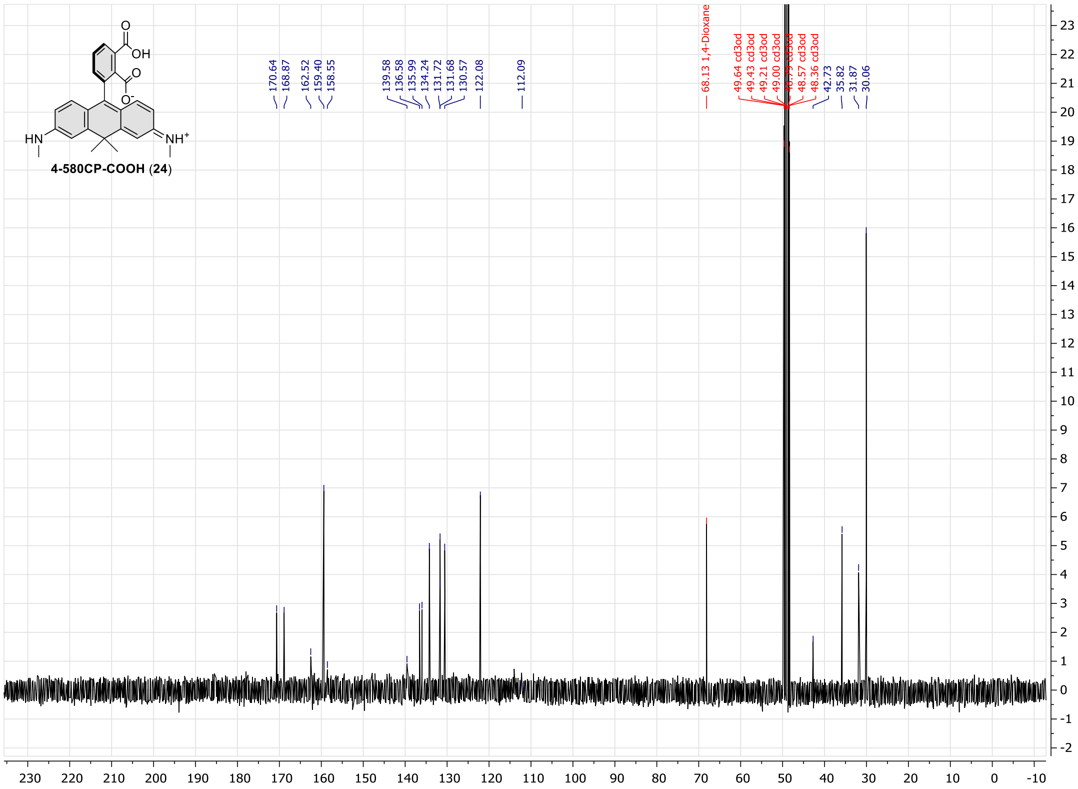

Supplementary Figure 240. ^{13}C NMR of compound **24** (100 MHz, CD_3OD).

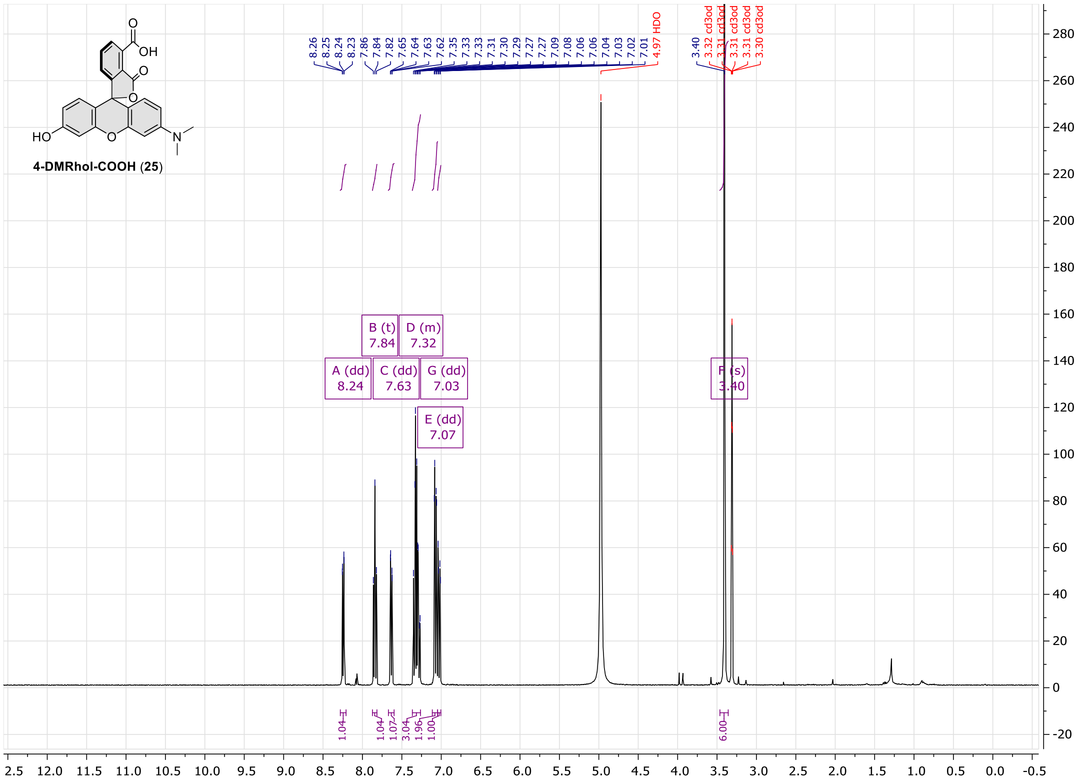

Supplementary Figure 241. ^{1}H NMR of compound **25** (400 MHz, CD_3OD).

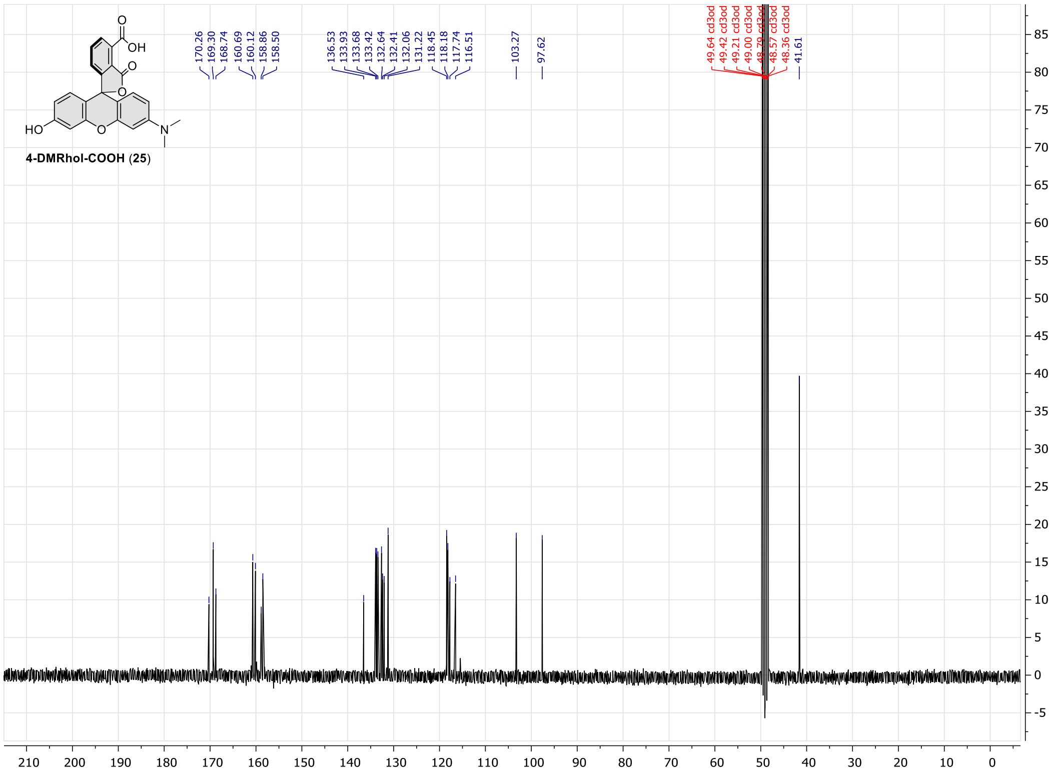

Supplementary Figure 242. ^{13}C NMR of compound **25** (100 MHz, CD_3OD).

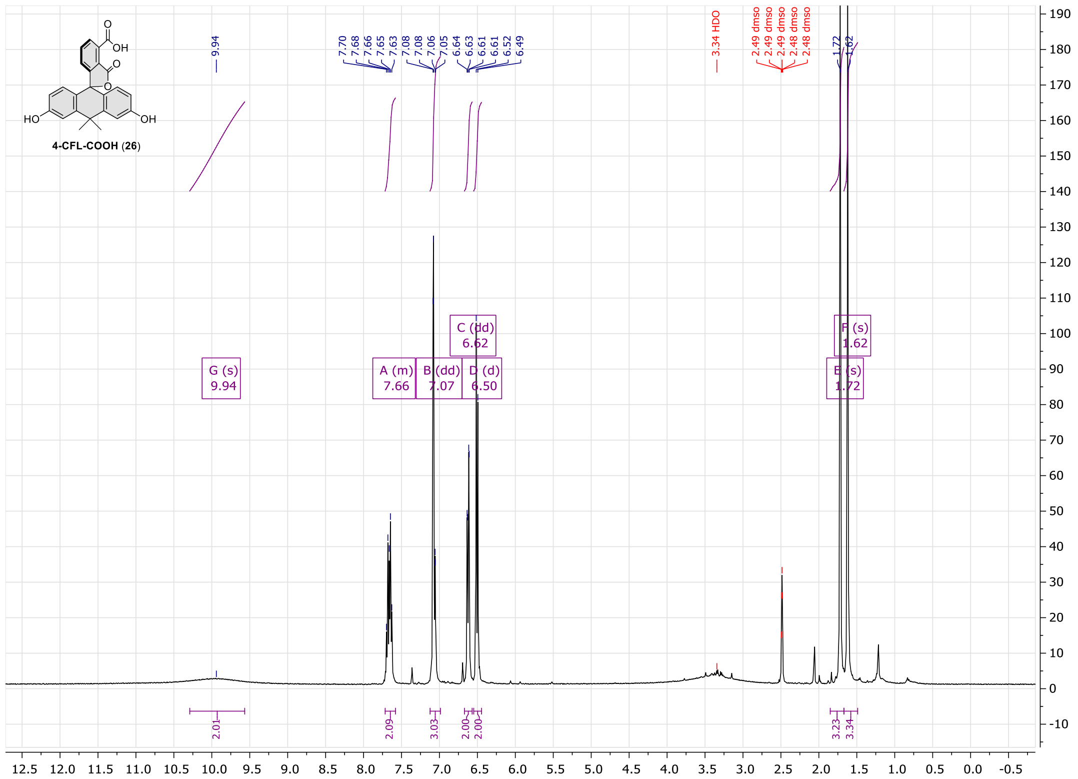

Supplementary Figure 243. ^{1}H NMR of compound **26** (400 MHz, d_6-DMSO).

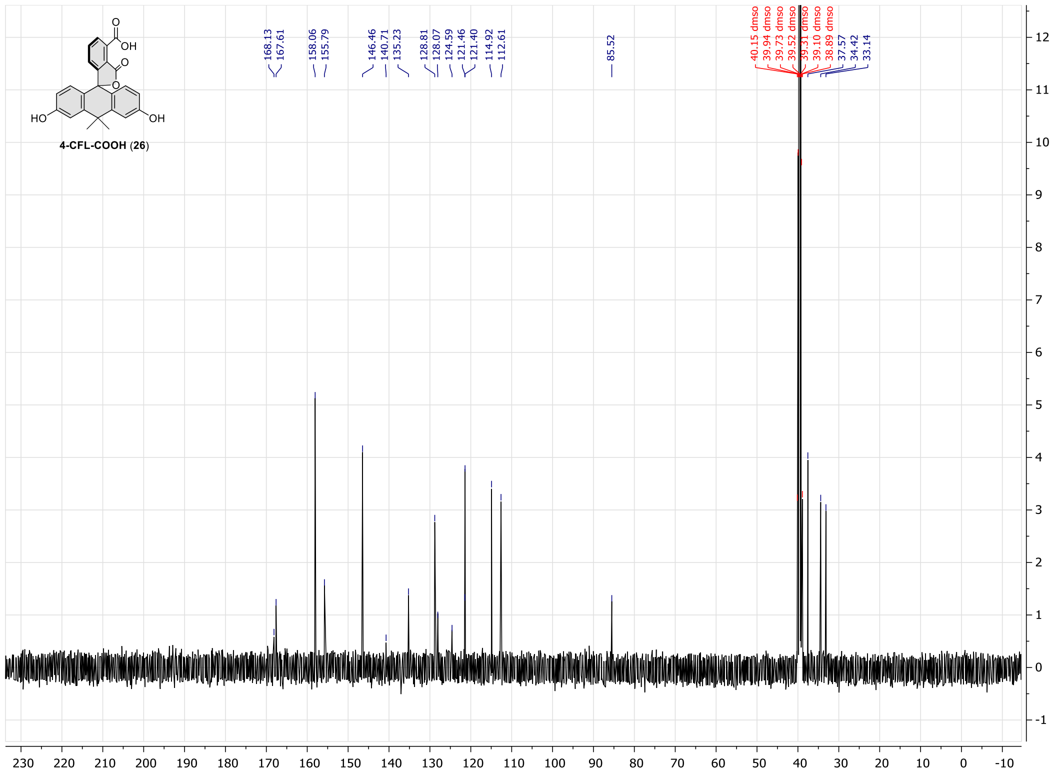

Supplementary Figure 245. ^{13}C NMR of compound **26** (100 MHz, d_6-DMSO).

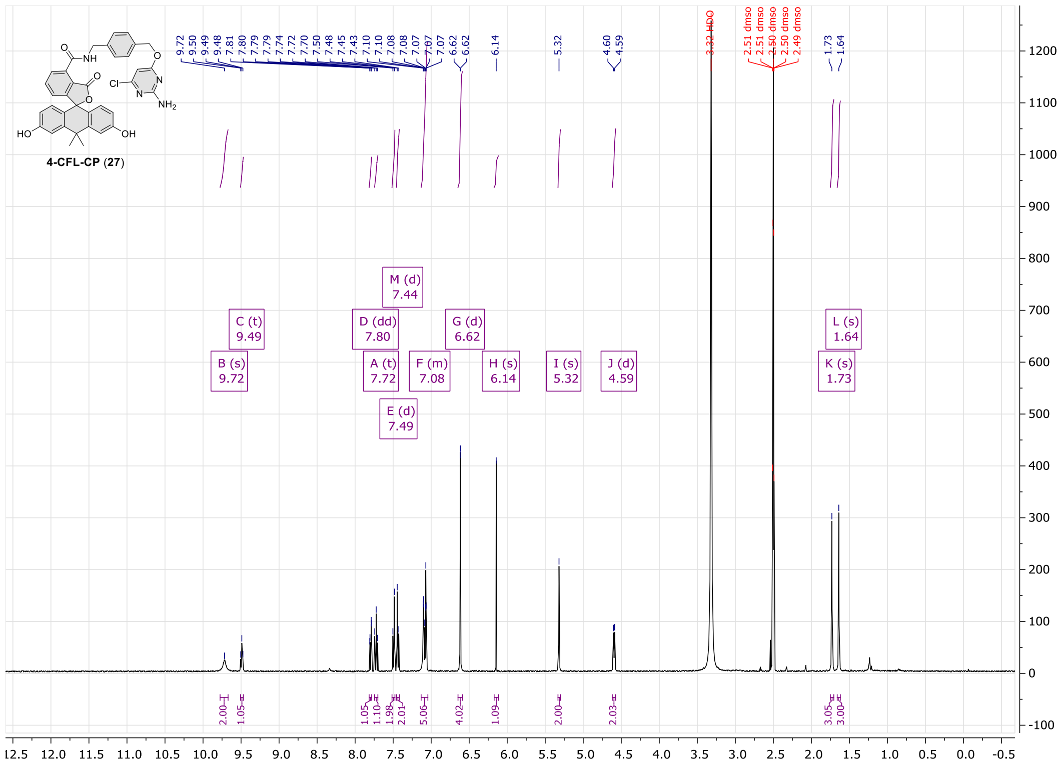

Supplementary Figure 246. ^{1}H NMR of compound **27** (400 MHz, d_6-DMSO).

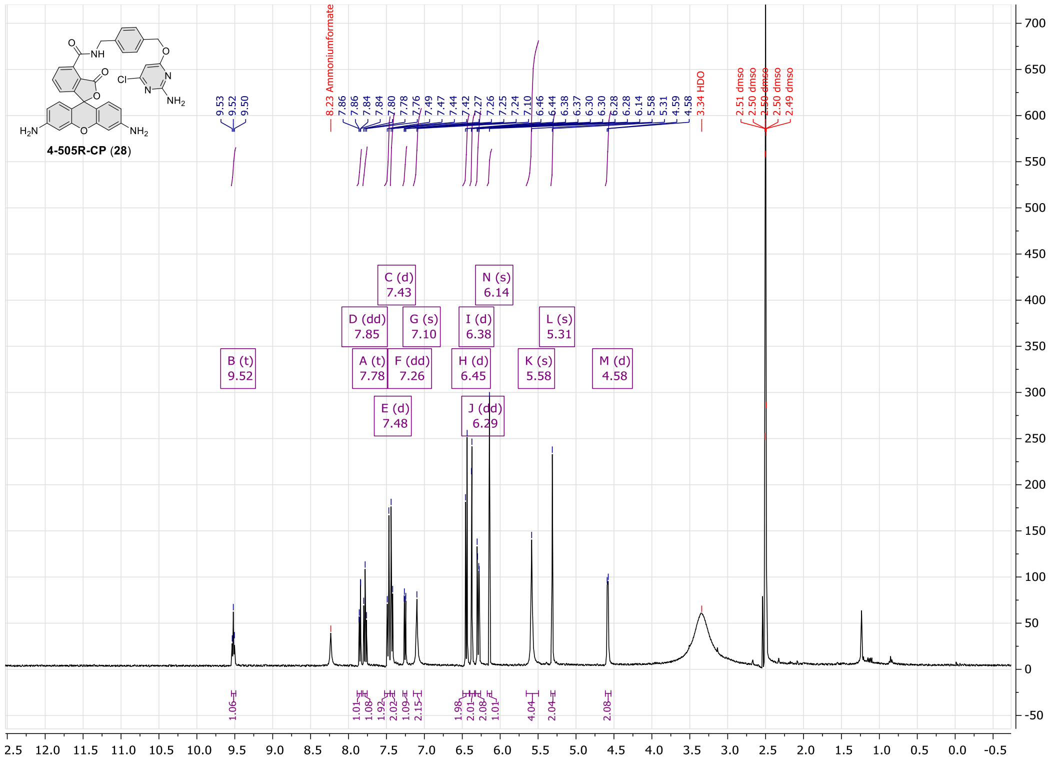

Supplementary Figure 247. ^{1}H NMR of compound **28** (400 MHz, d_6-DMSO).

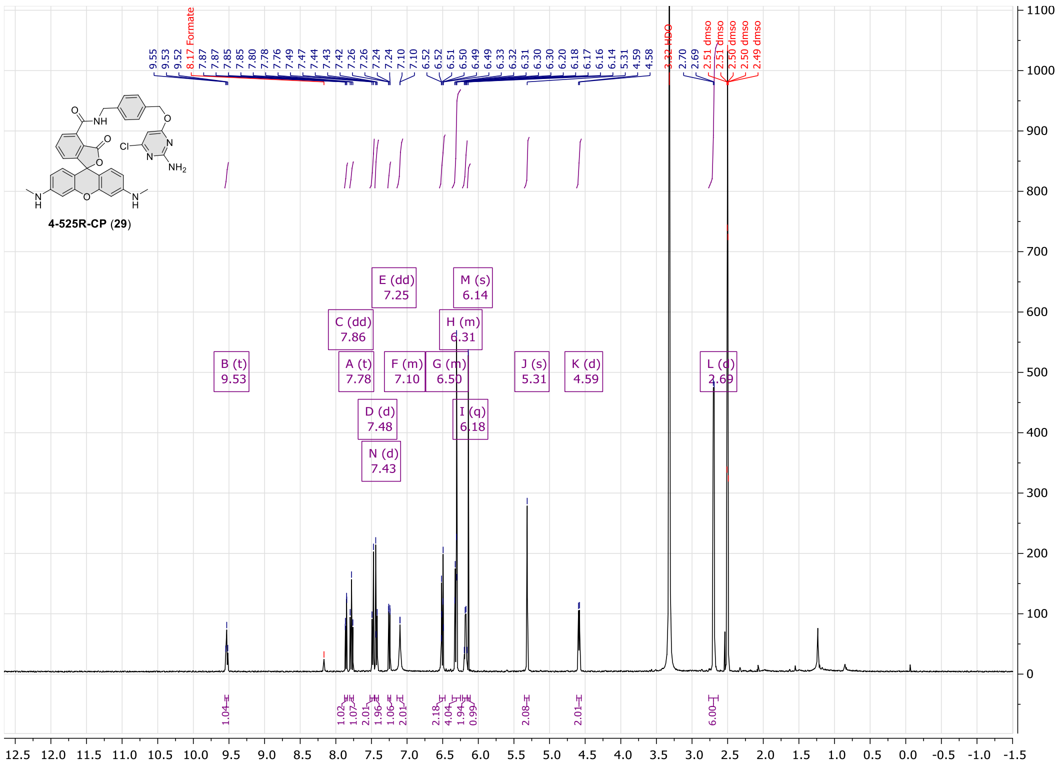

Supplementary Figure 248. ^{1}H NMR of compound **29** (400 MHz, d_6-DMSO).

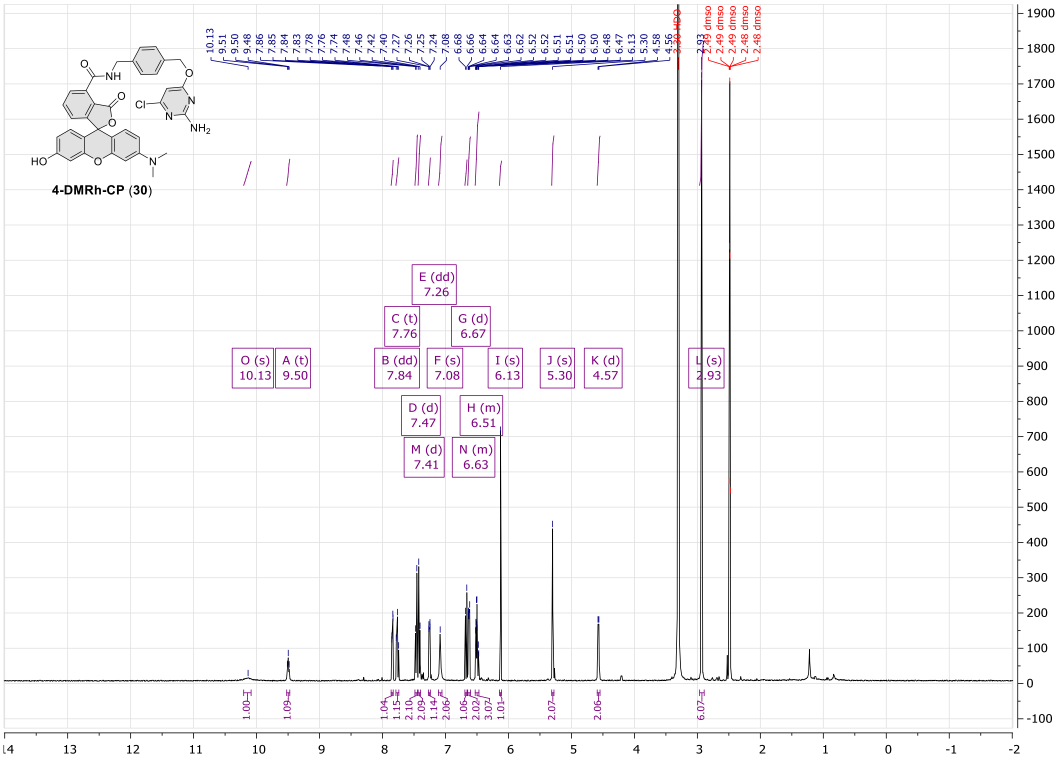

Supplementary Figure 249. ^{1}H NMR of compound **30** (400 MHz, d_6-DMSO).

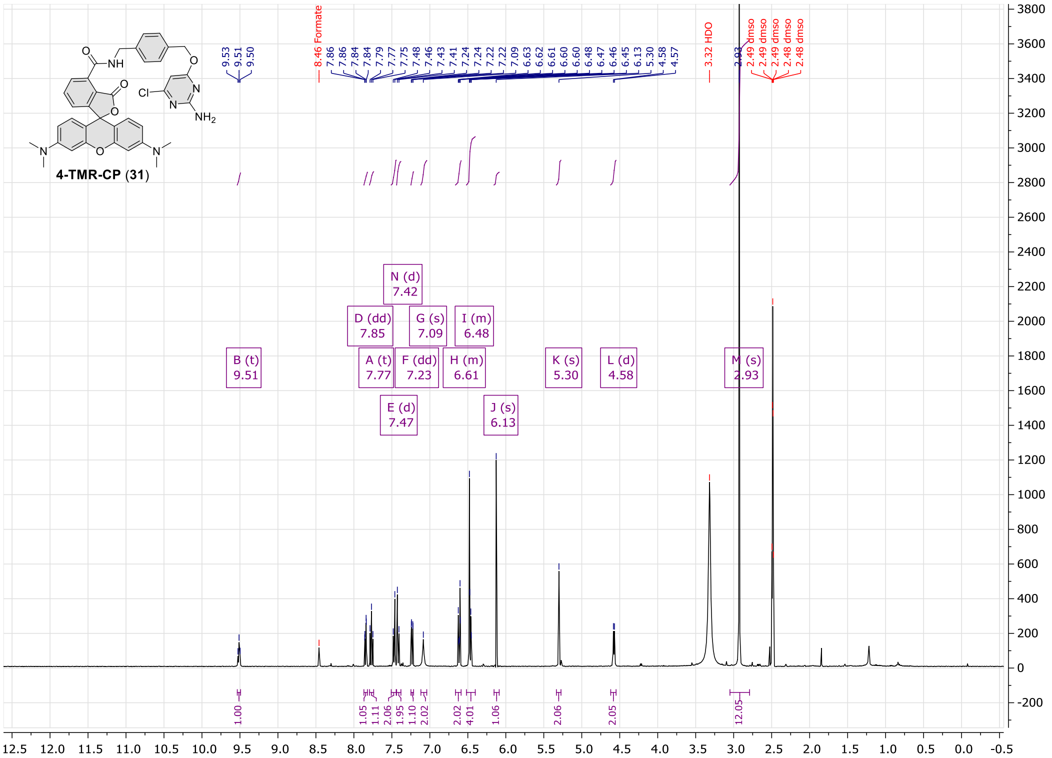

Supplementary Figure 250. ^{1}H NMR of compound **31** (400 MHz, d_6-DMSO).

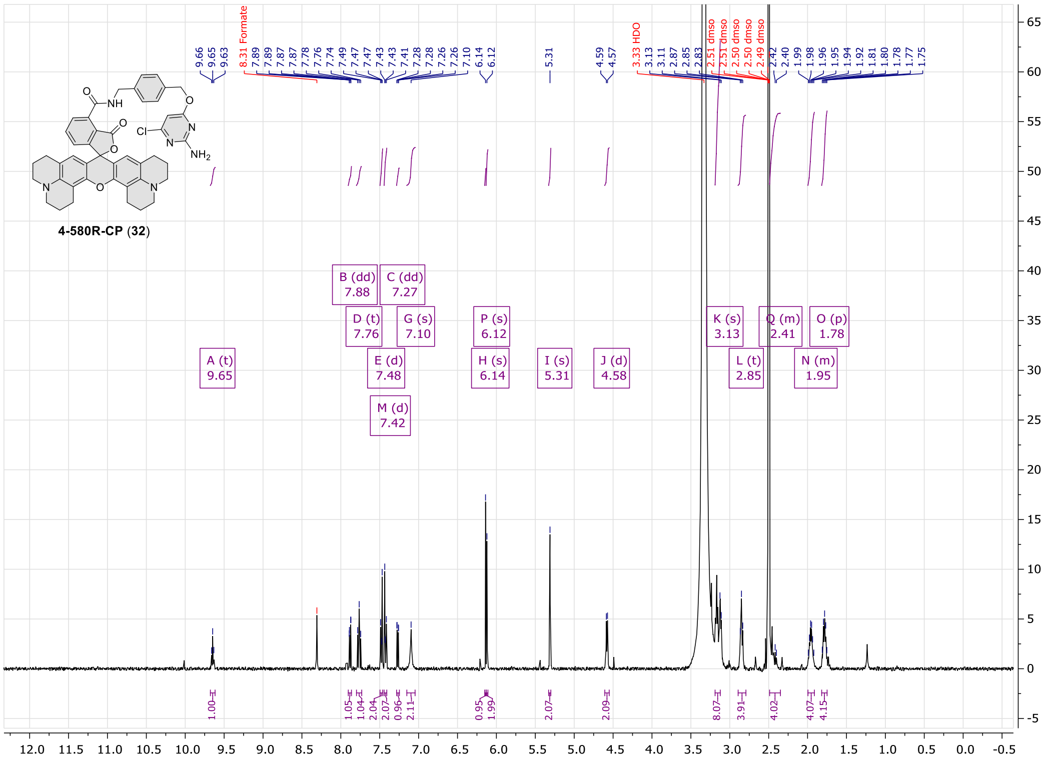

Supplementary Figure 251. ^{1}H NMR of compound **32** (400 MHz, d_6-DMSO).

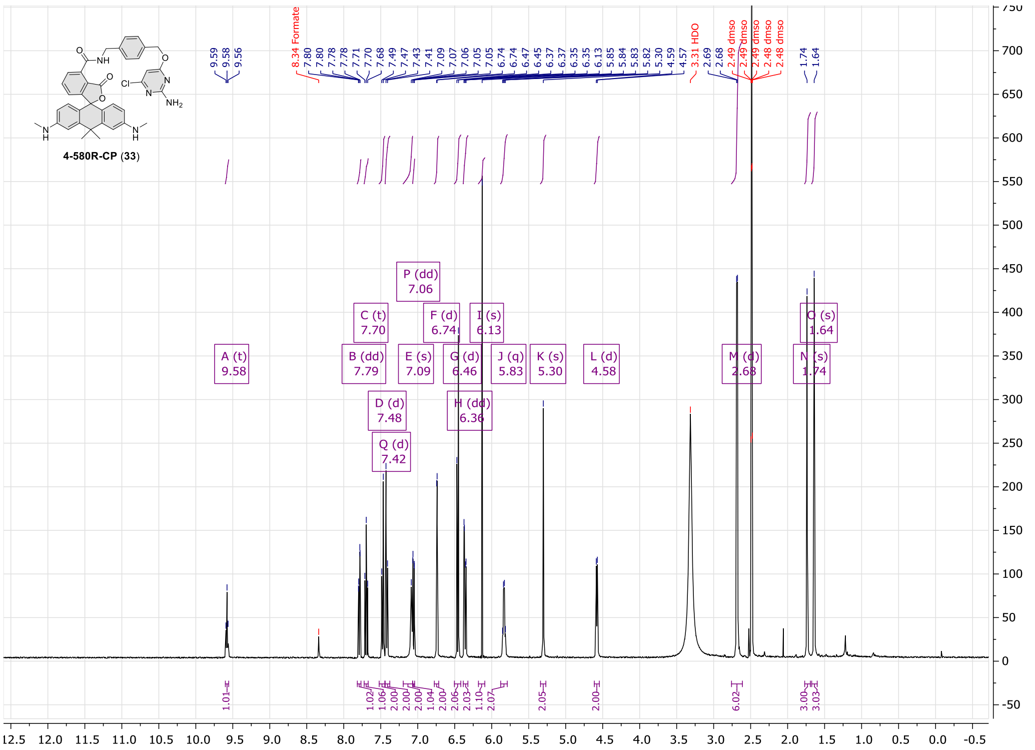

Supplementary Figure 252. ^{1}H NMR of compound **33** (400 MHz, d_6-DMSO).

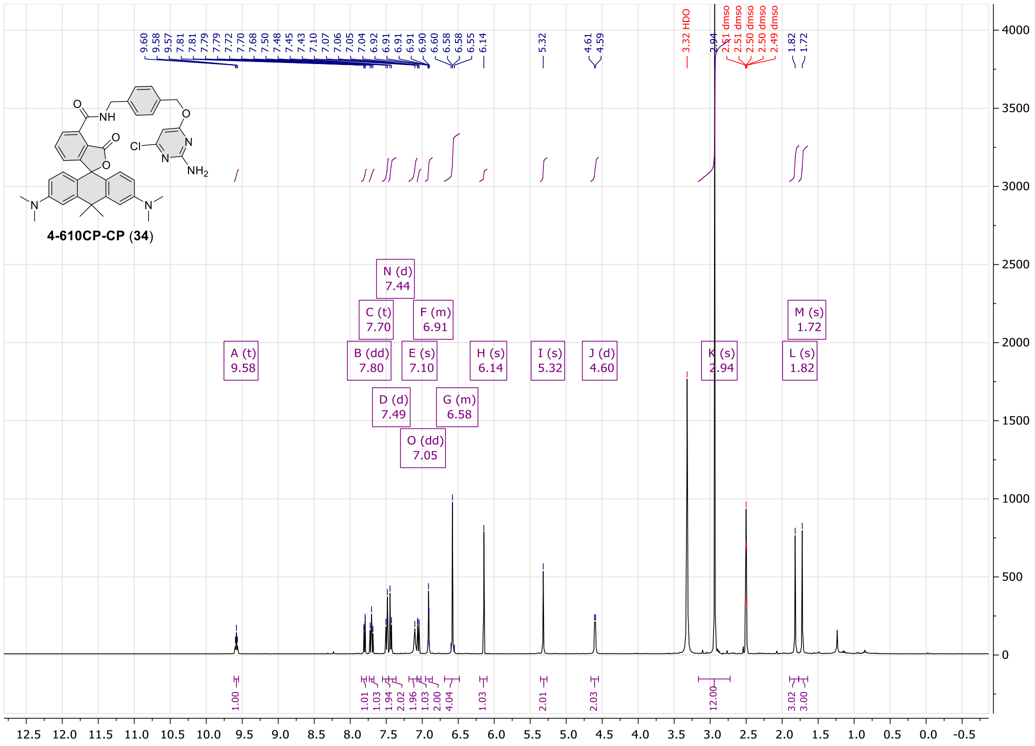

Supplementary Figure 253. ^{1}H NMR of compound **34** (400 MHz, d_6-DMSO).

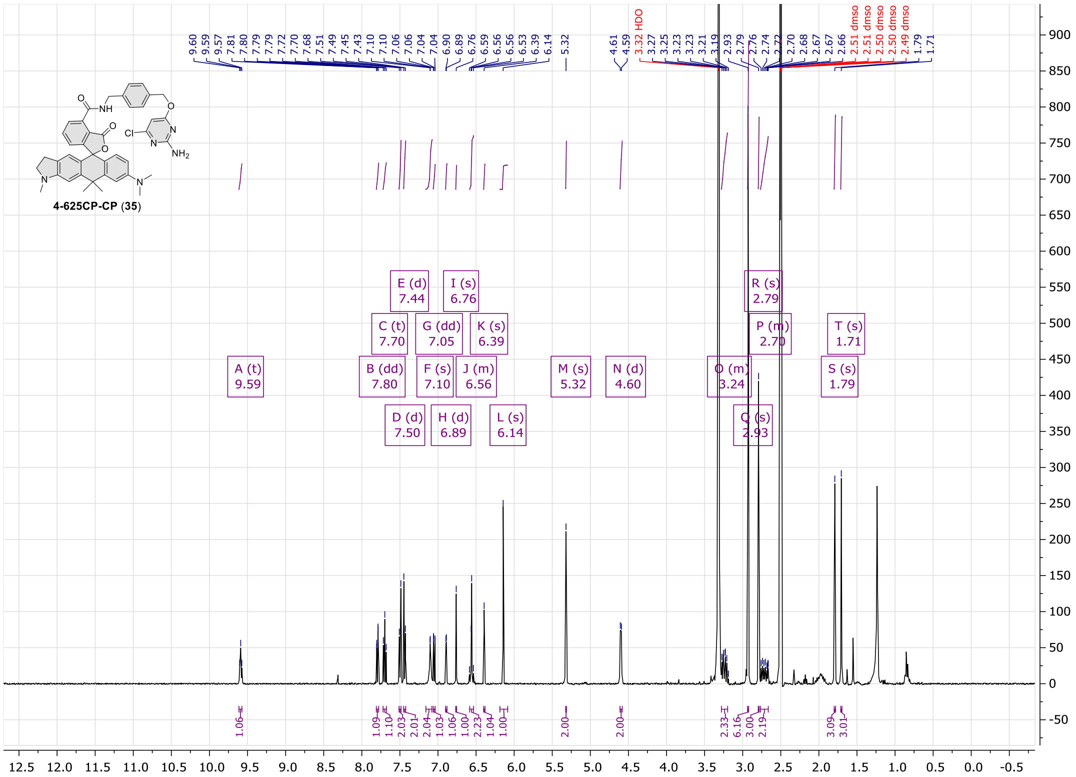

Supplementary Figure 254. ^{1}H NMR of compound **35** (400 MHz, d_6-DMSO).

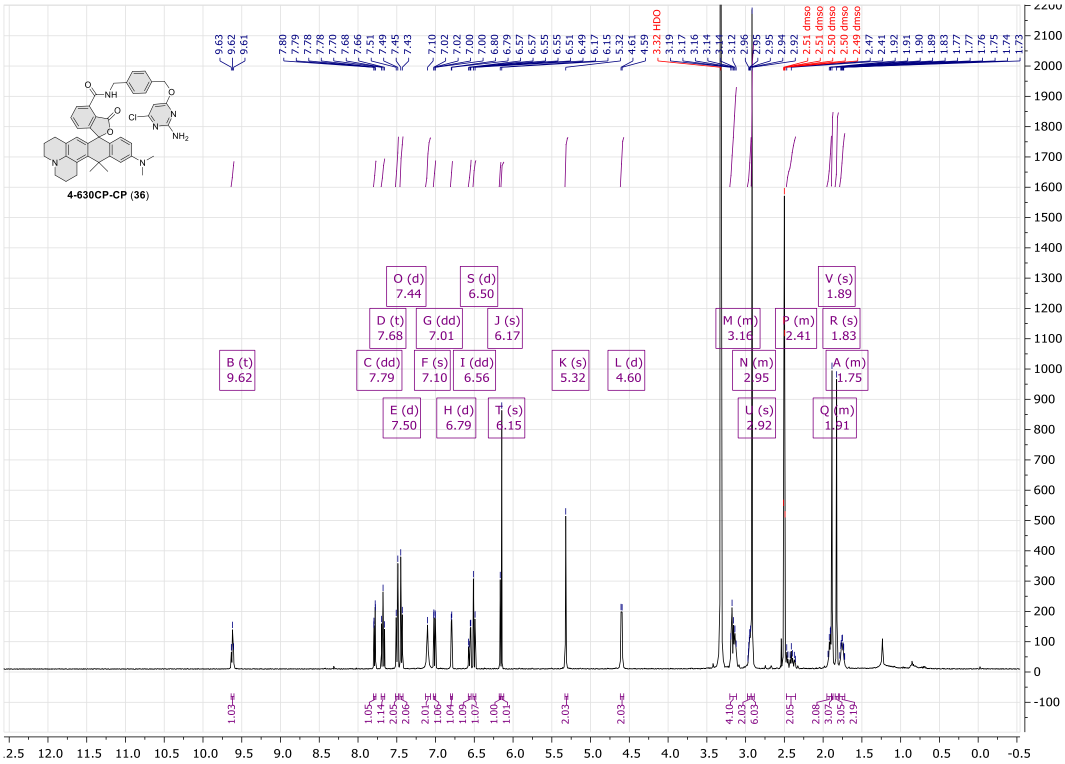

Supplementary Figure 255. ^{1}H NMR of compound **36** (400 MHz, d_6-DMSO).

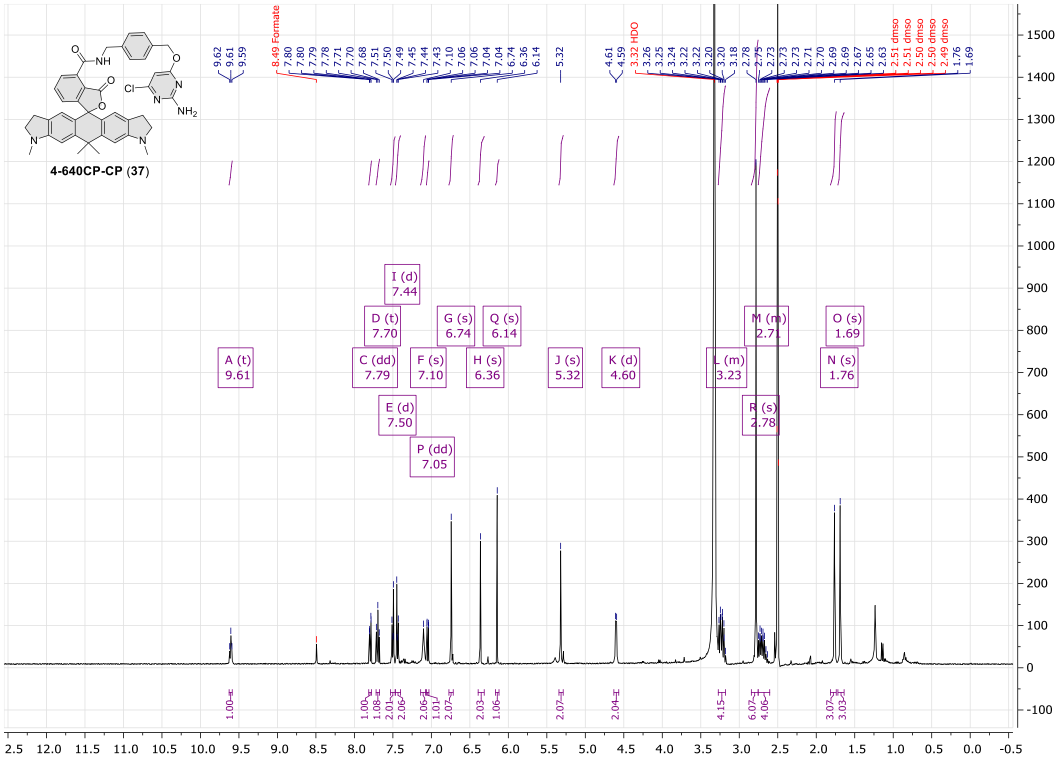

Supplementary Figure 256. ^{1}H NMR of compound **37** (400 MHz, d_6-DMSO).

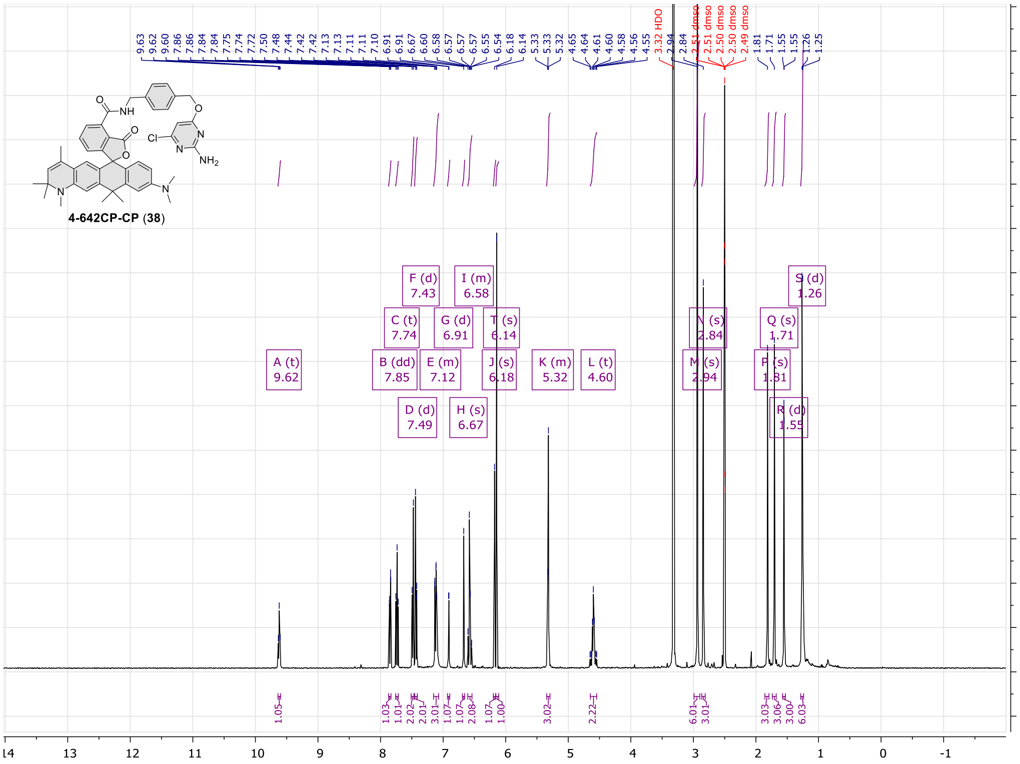

Supplementary Figure 257. ^{1}H NMR of compound **38** (400 MHz, d_6-DMSO).

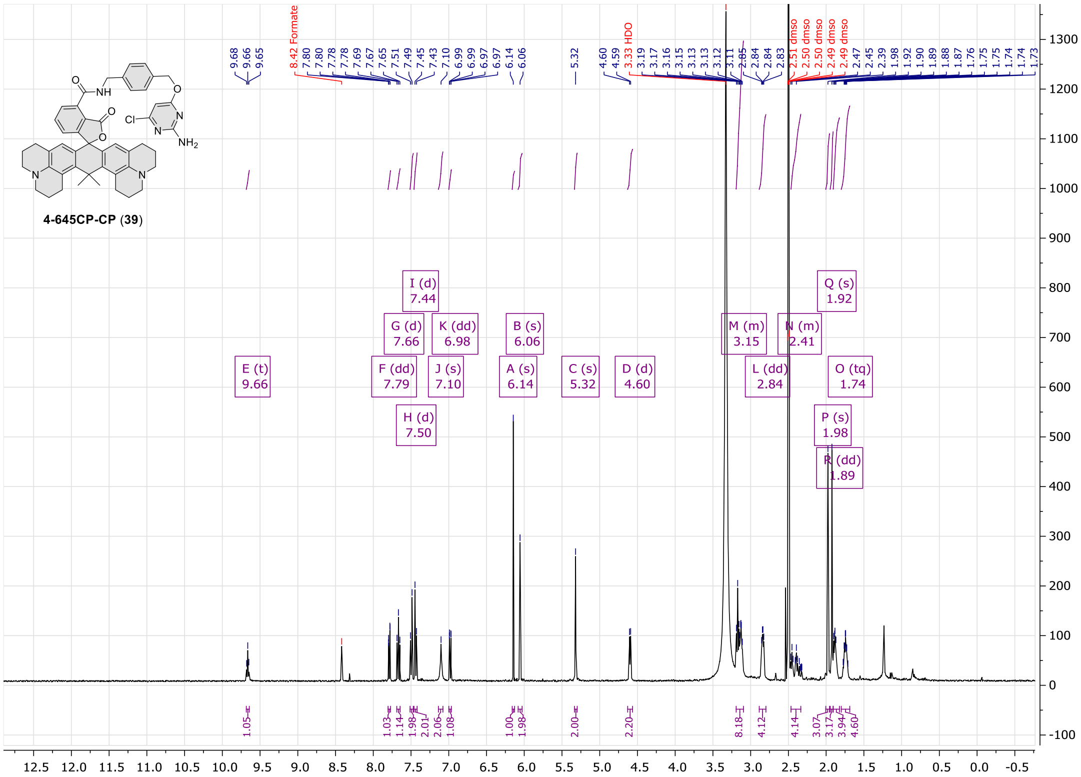

Supplementary Figure 258. ^{1}H NMR of compound **39** (400 MHz, d_6-DMSO).

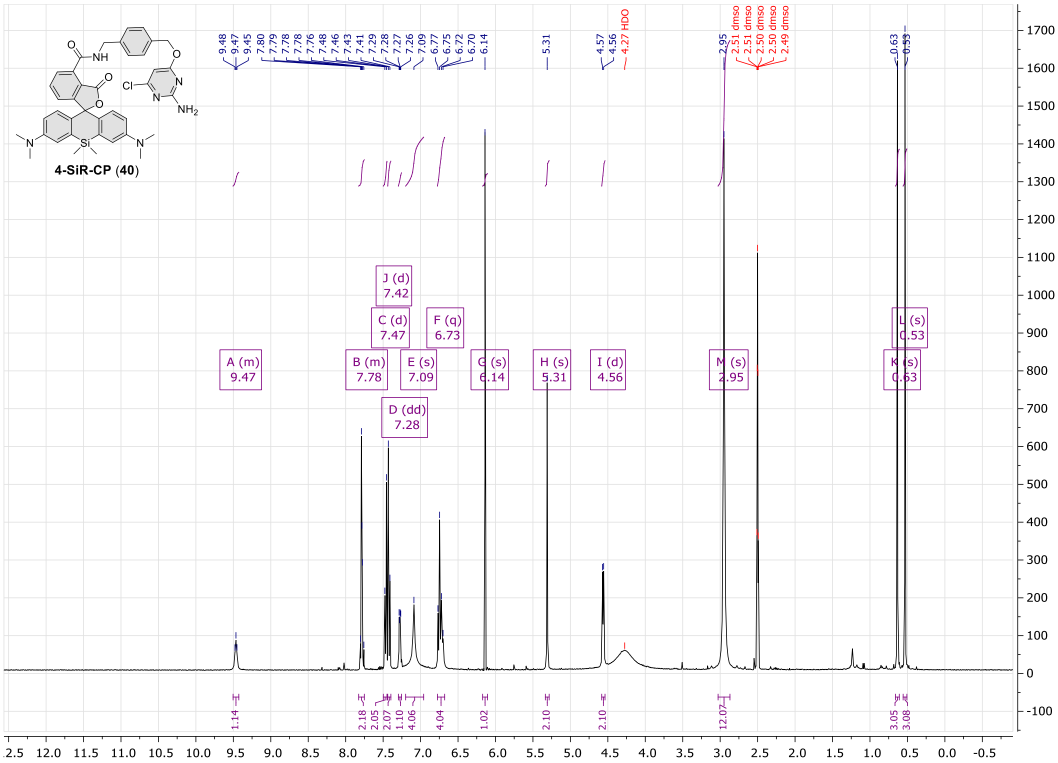

Supplementary Figure 259. ^{1}H NMR of compound **40** (400 MHz, d_6-DMSO).

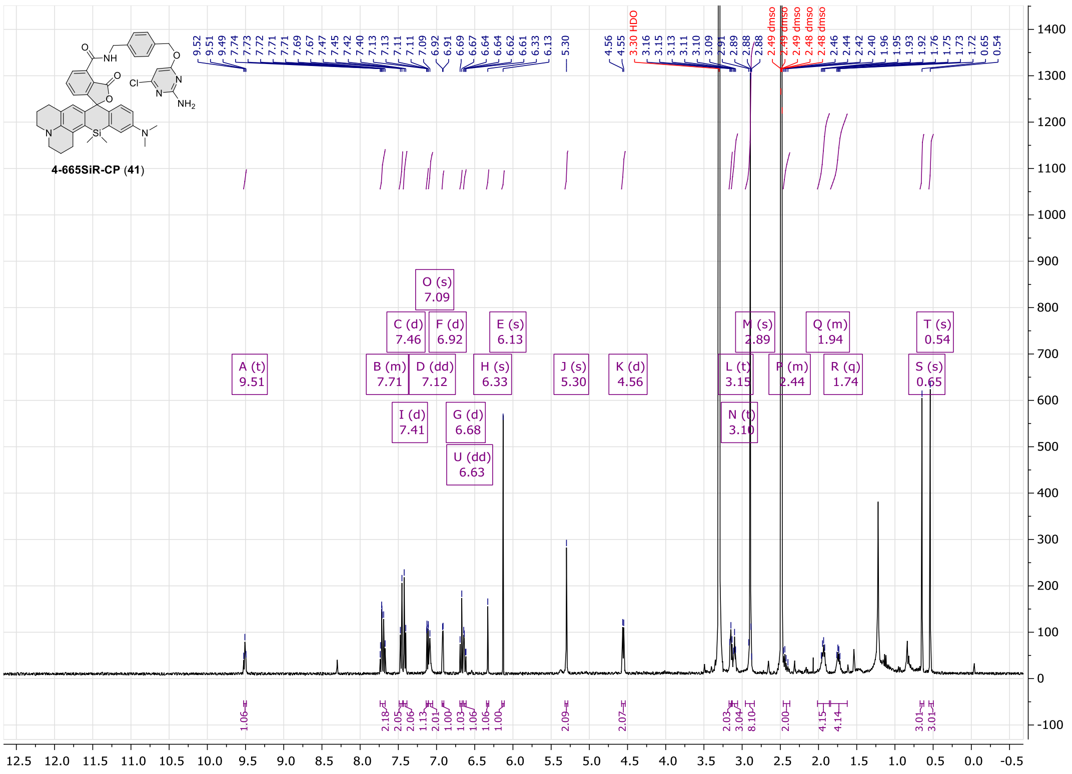

Supplementary Figure 260. ^{1}H NMR of compound **41** (400 MHz, d_6-DMSO).

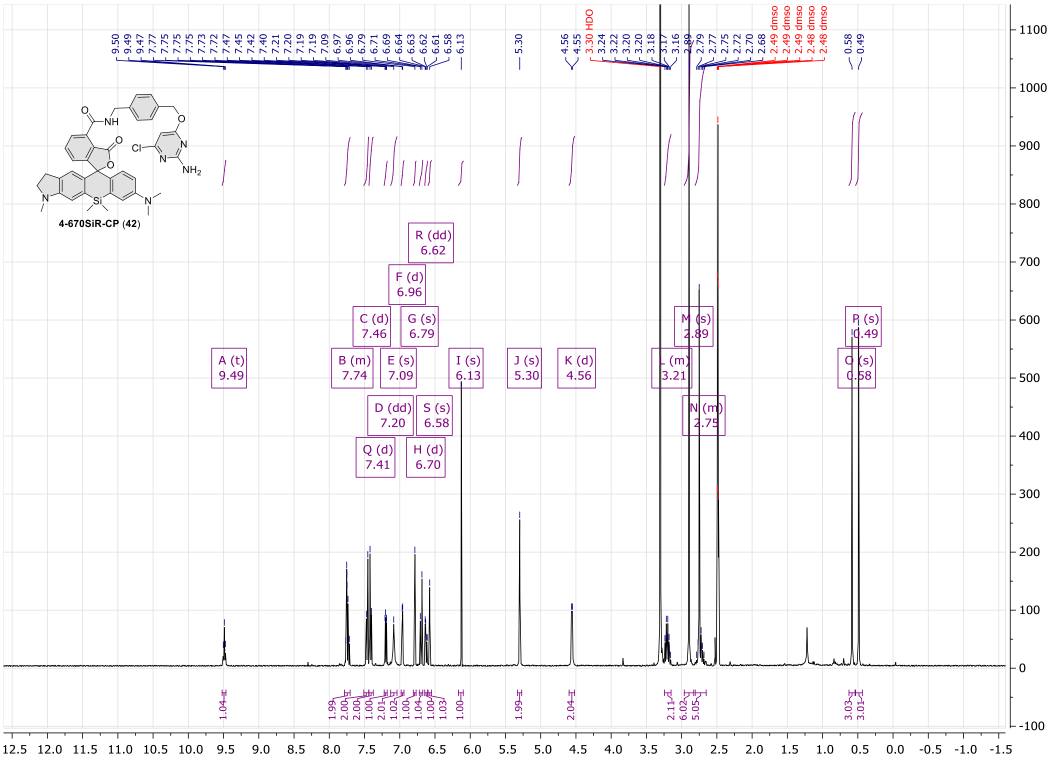

Supplementary Figure 261. ^{1}H NMR of compound **42** (400 MHz, d_6-DMSO).

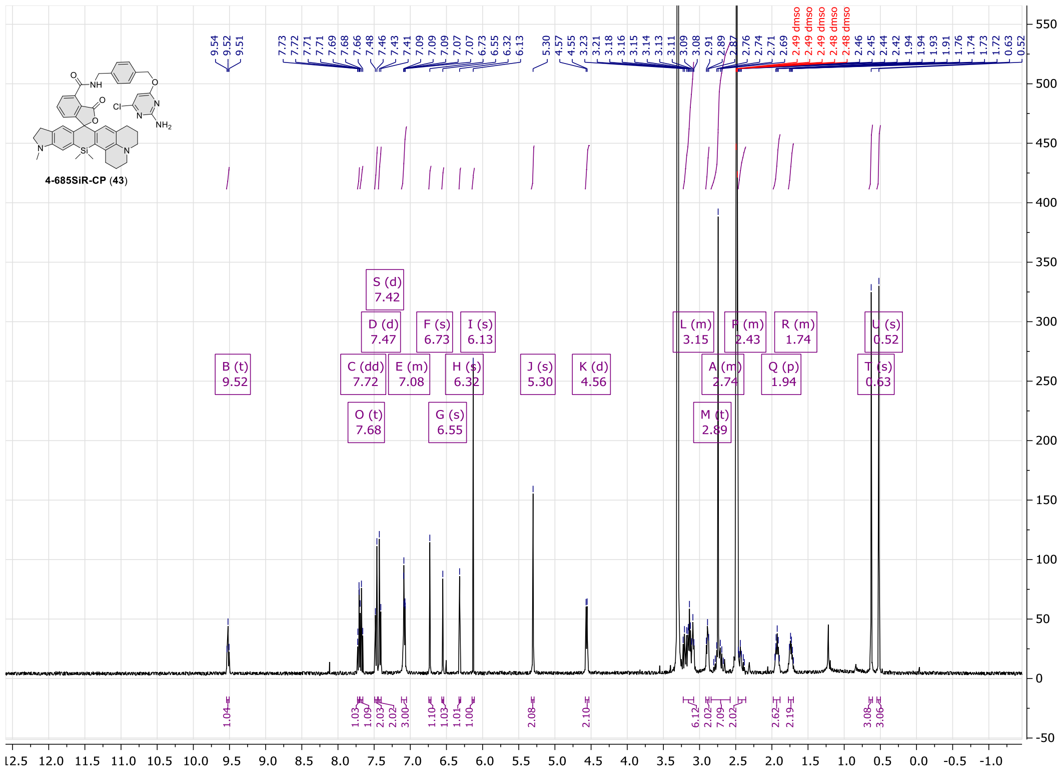

Supplementary Figure 262. ^1H NMR of compound **43** (400 MHz, d_6-DMSO).

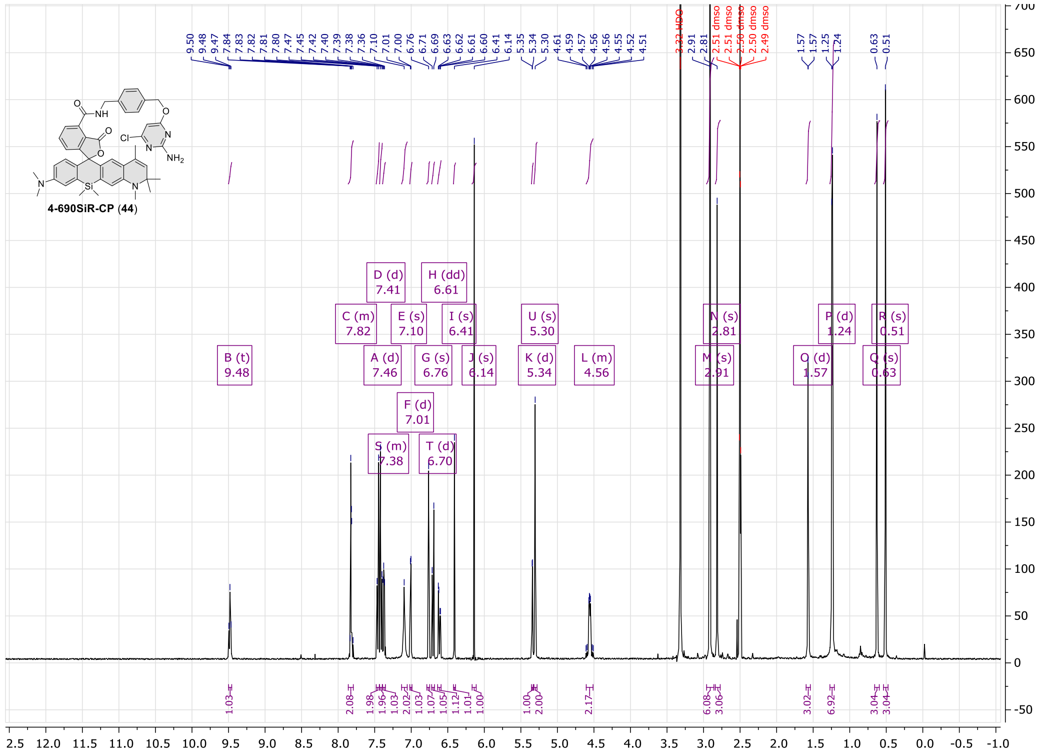

Supplementary Figure 263. ^1H NMR of compound **44** (400 MHz, d_6-DMSO).

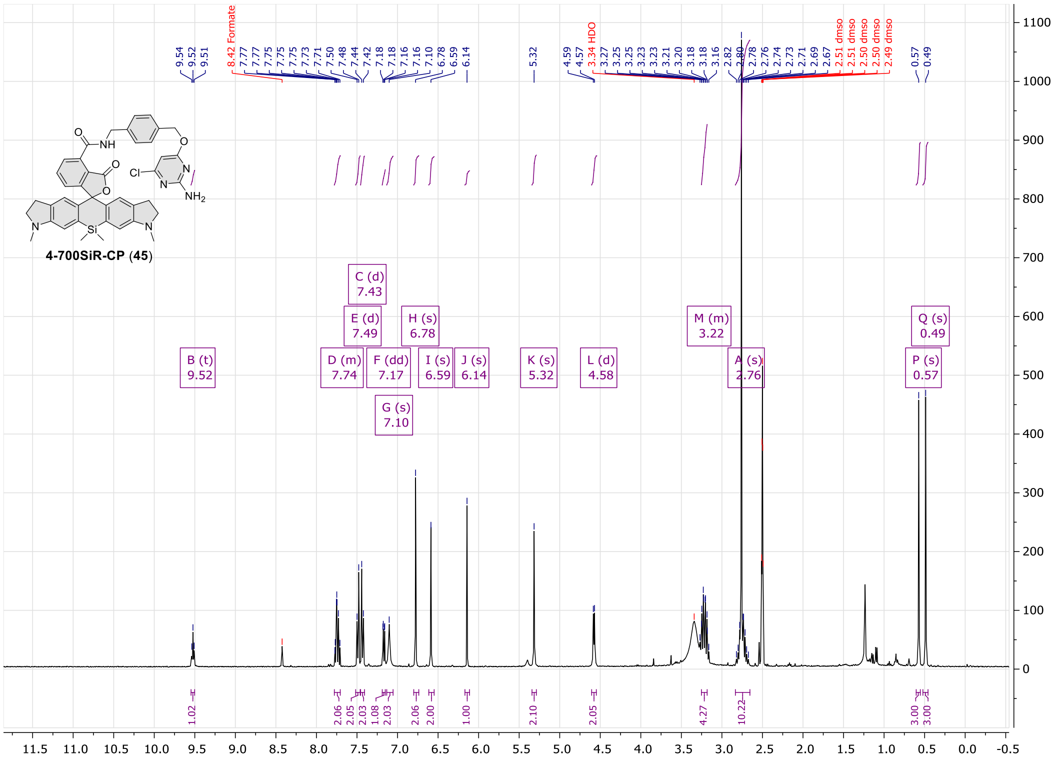

Supplementary Figure 264. ^{1}H NMR of compound **45** (400 MHz, d_6-DMSO).

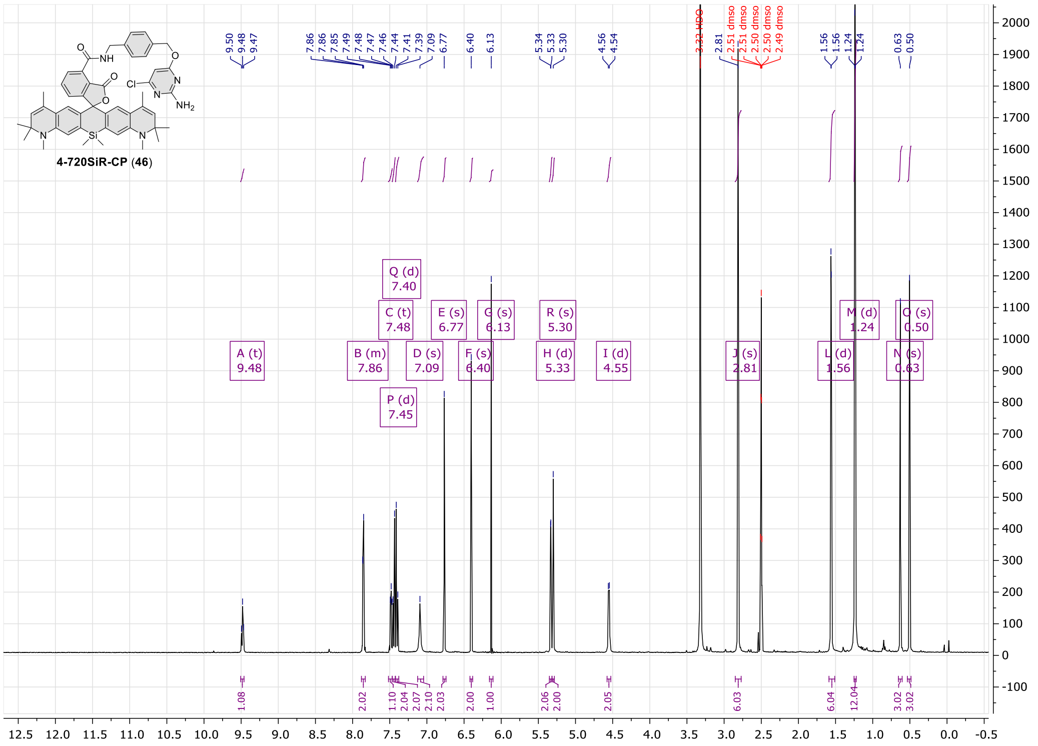

Supplementary Figure 265. ^{1}H NMR of compound **46** (400 MHz, d_6-DMSO).

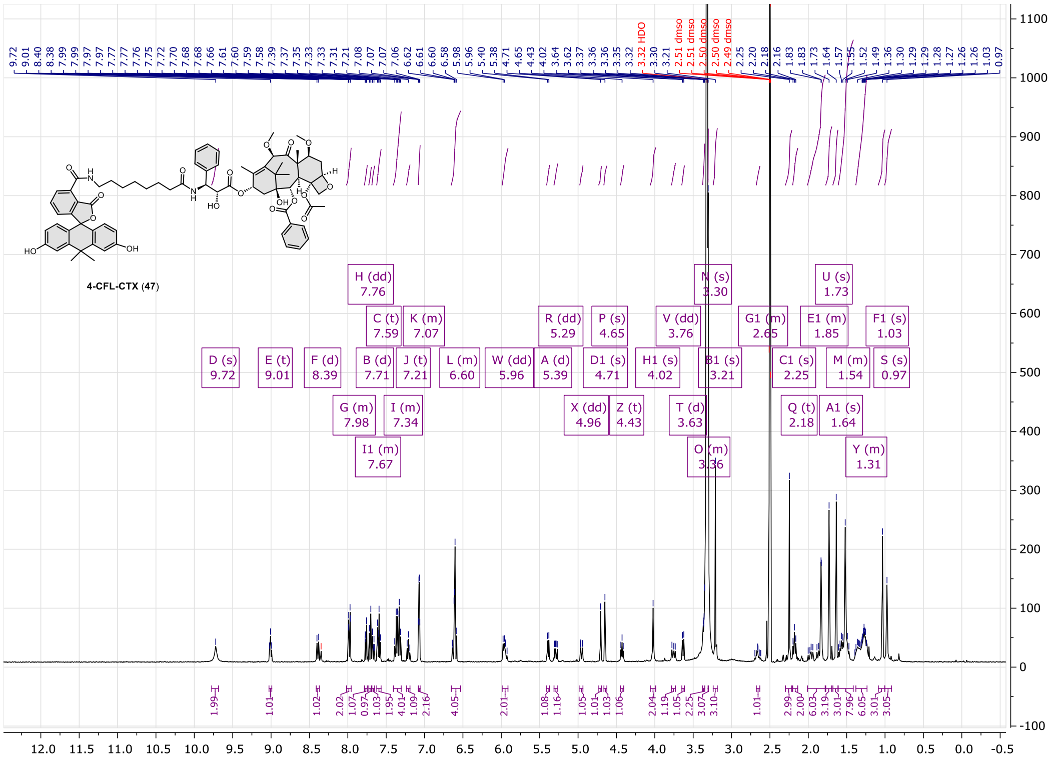

Supplementary Figure 266. ^{1}H NMR of compound **47** (400 MHz, d_6-DMSO).

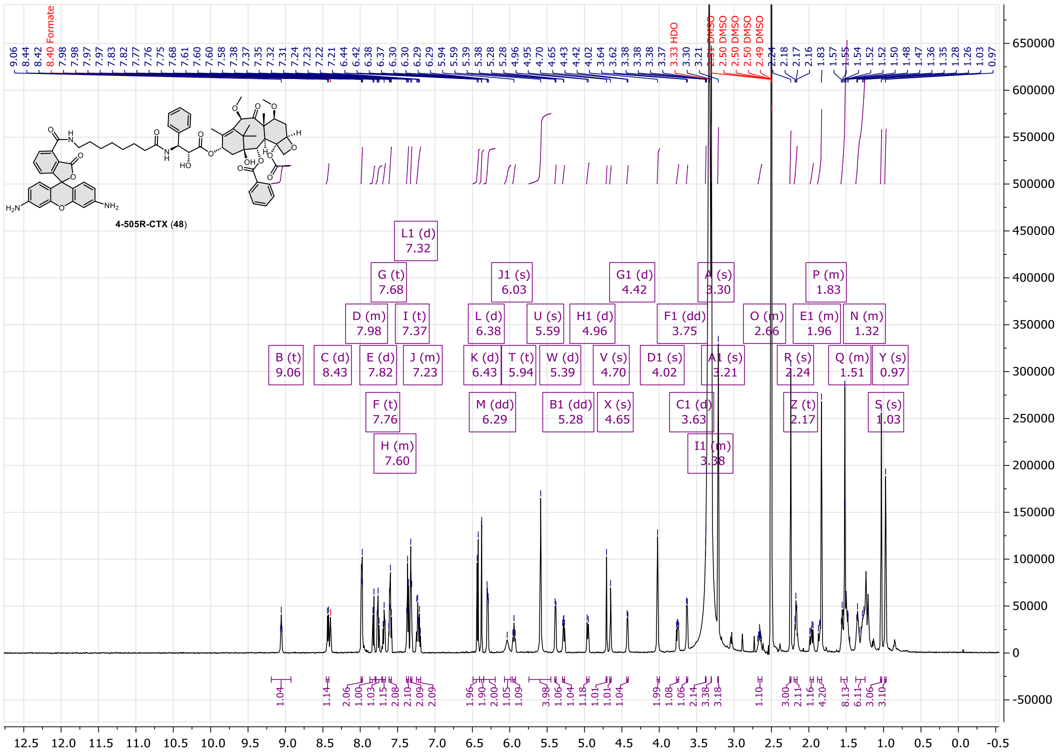

Supplementary Figure 267. ^{1}H NMR of compound **48** (400 MHz, d_6-DMSO).

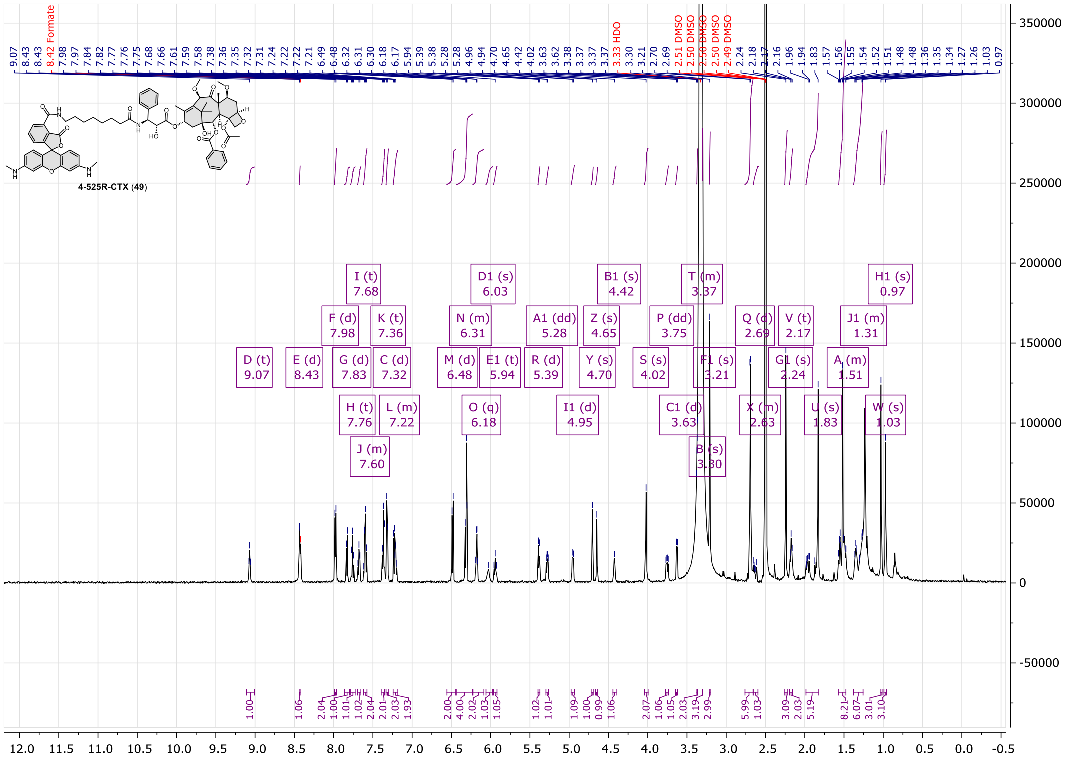

Supplementary Figure 268. ^{1}H NMR of compound **49** (400 MHz, d_6-DMSO).

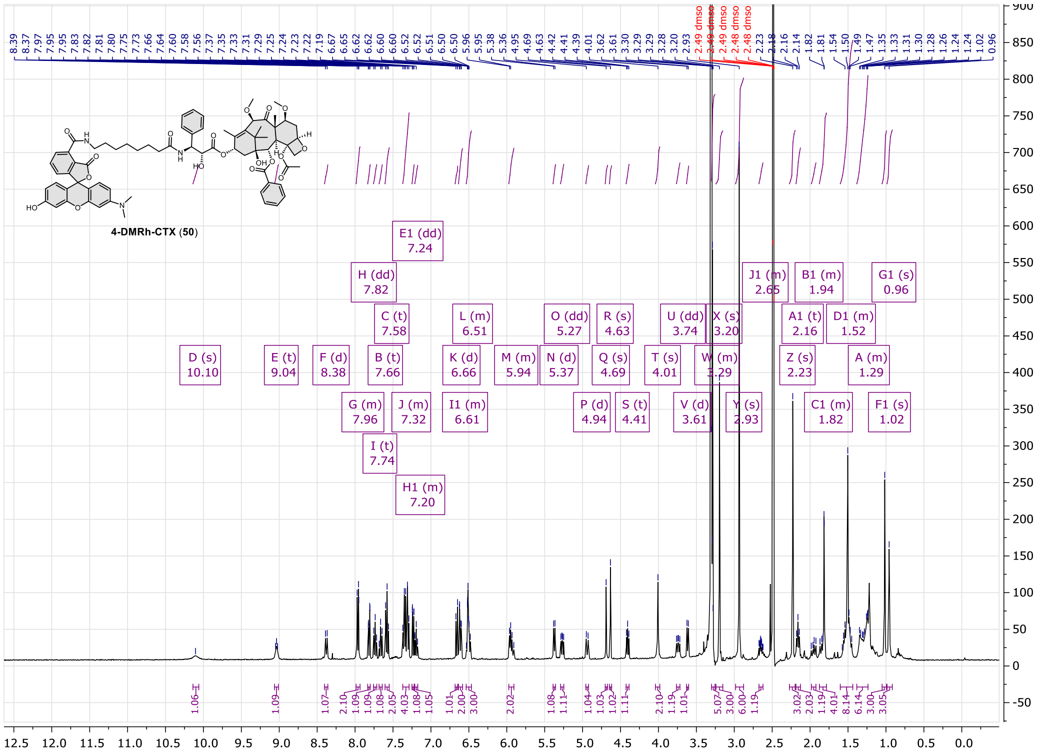

Supplementary Figure 269. ^{1}H NMR of compound **50** (400 MHz, d_6-DMSO).

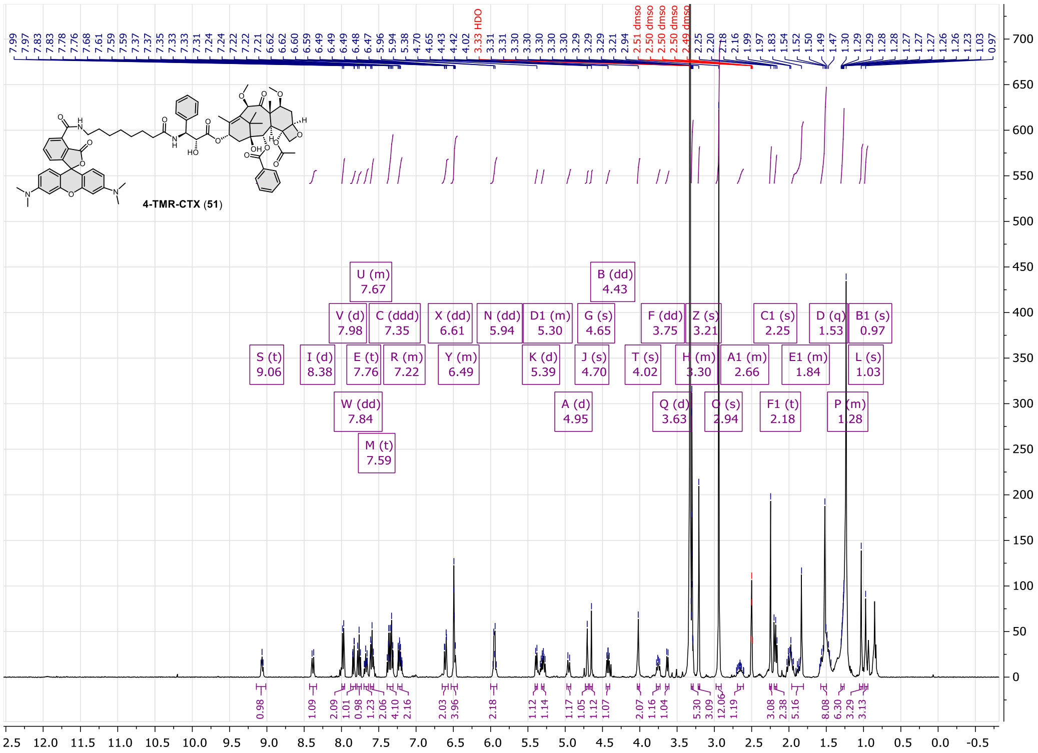

Supplementary Figure 270. ^{1}H NMR of compound **51** (400 MHz, d_6-DMSO).

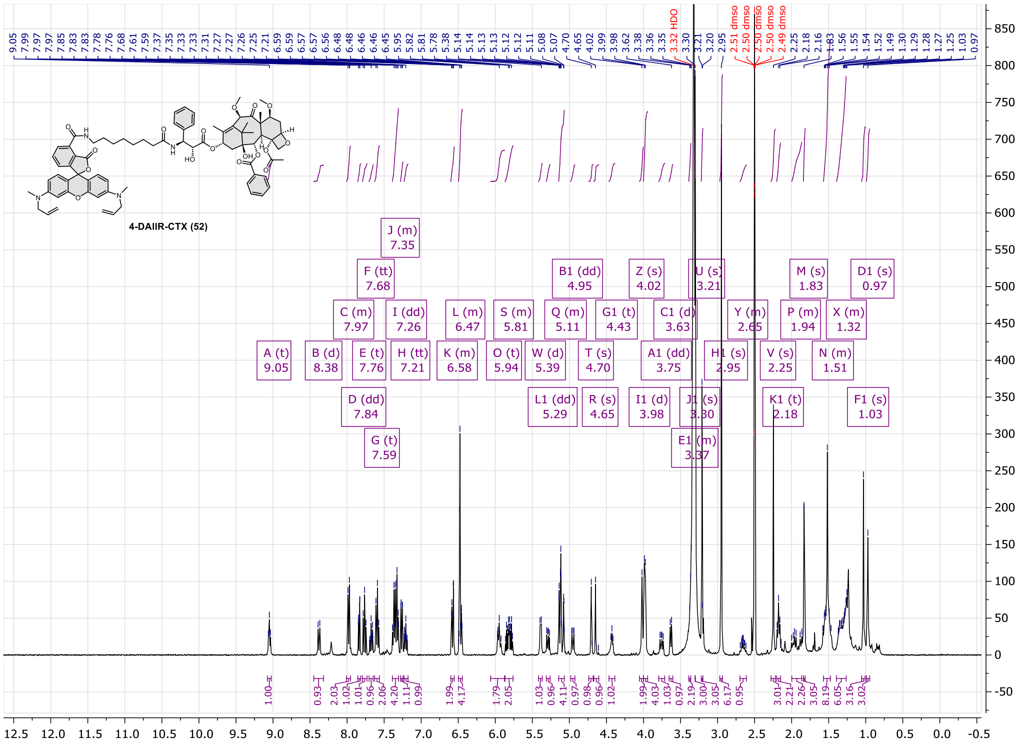

Supplementary Figure 271. ^{1}H NMR of compound **52** (400 MHz, d_6-DMSO).

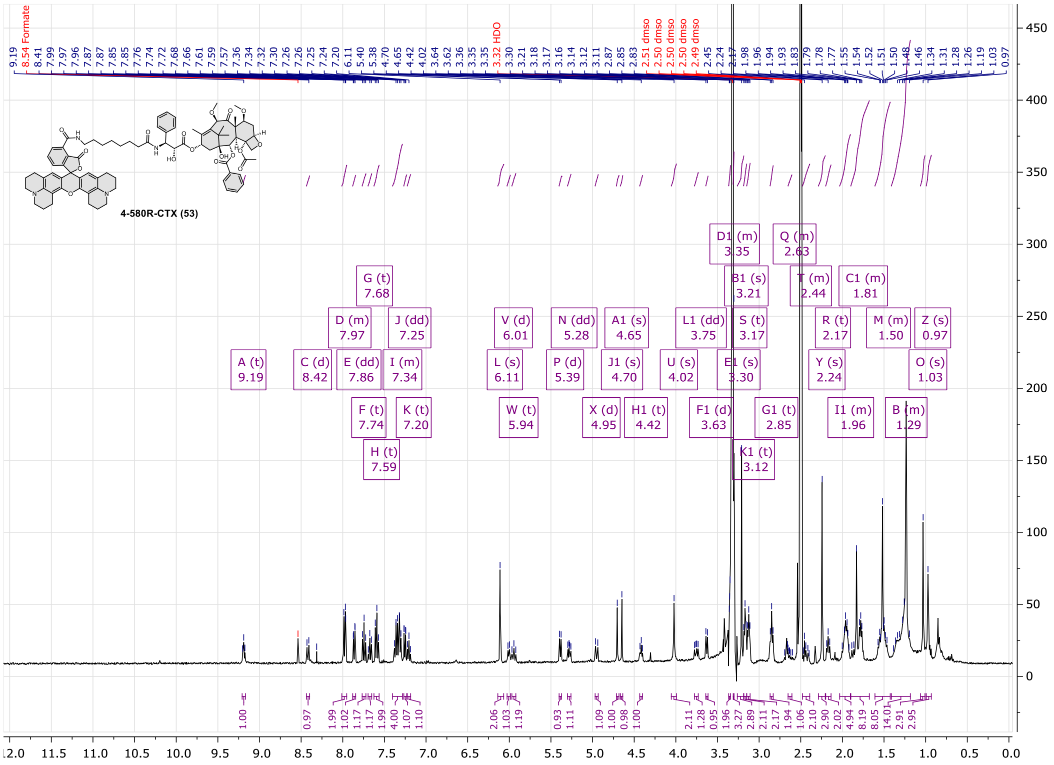

Supplementary Figure 272. ^{1}H NMR of compound **53** (400 MHz, d_6-DMSO).

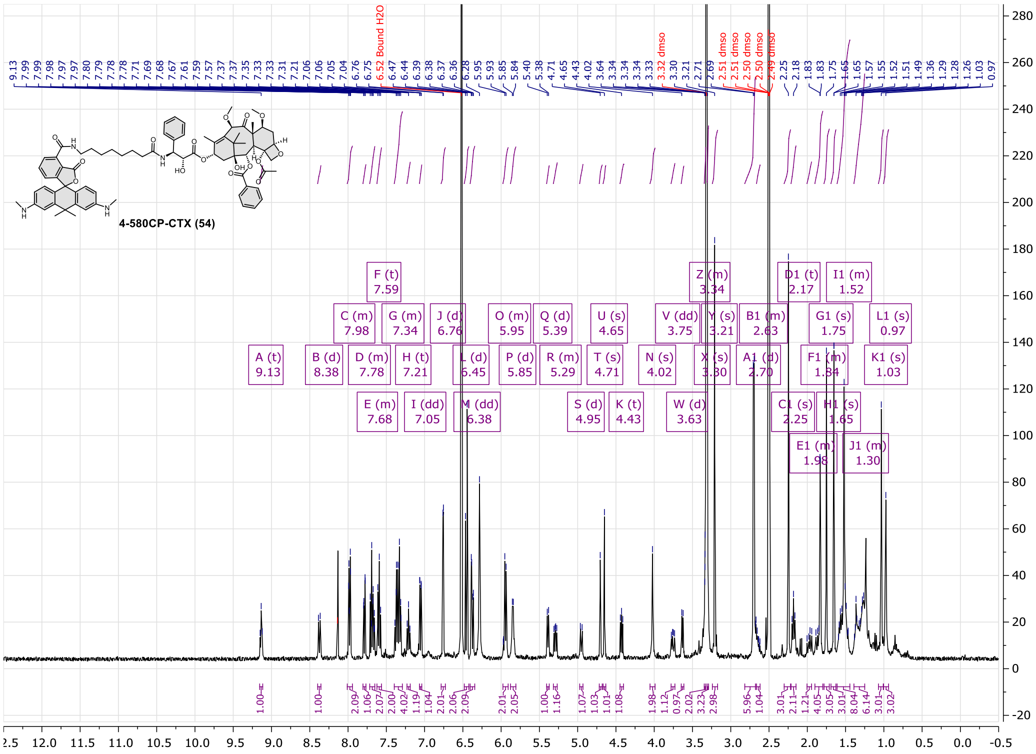

Supplementary Figure 273. ^{1}H NMR of compound **54** (400 MHz, d_6-DMSO).

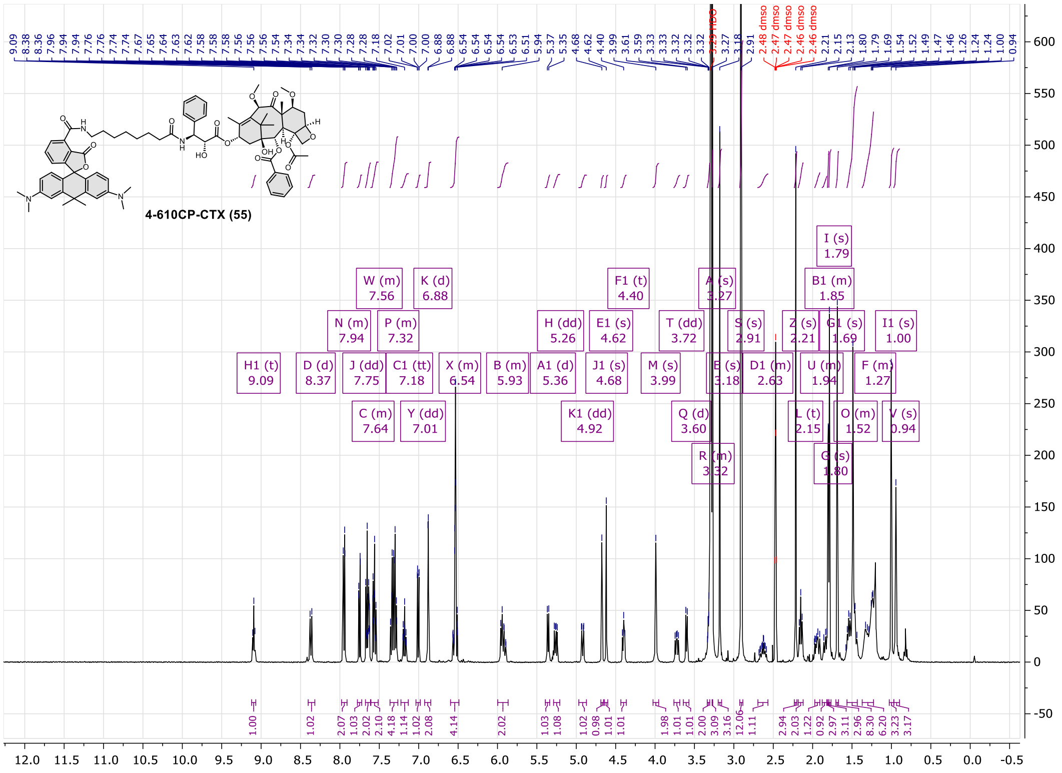

Supplementary Figure 274. ^{1}H NMR of compound **55** (400 MHz, d_6-DMSO).

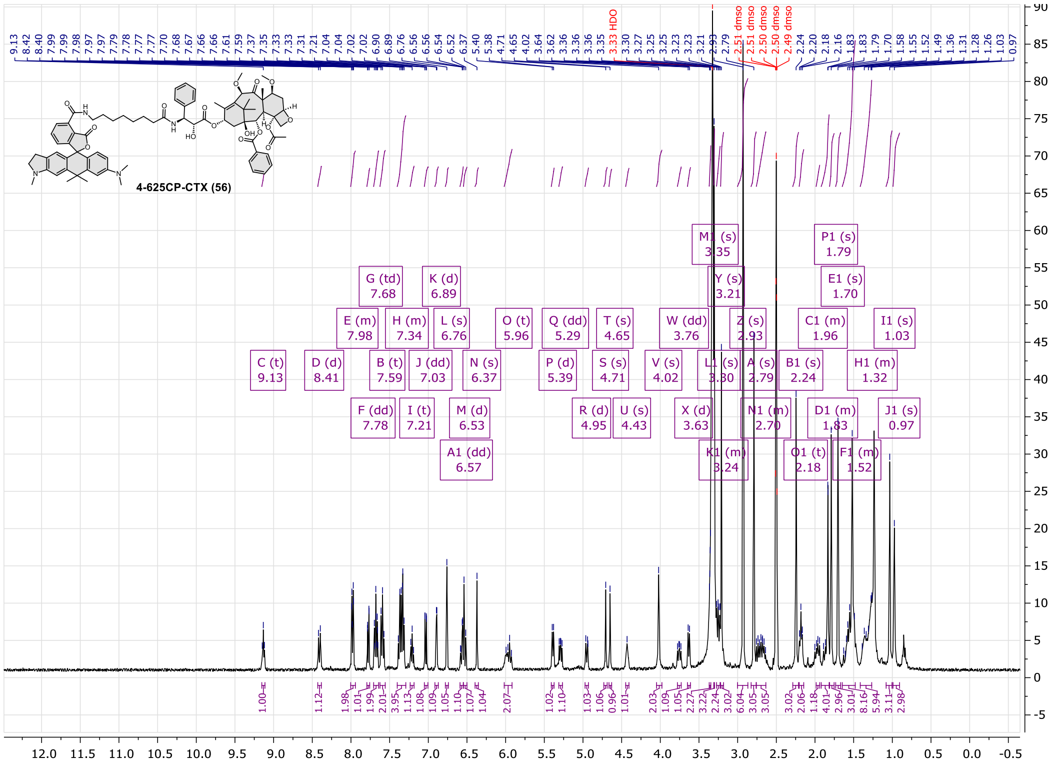

Supplementary Figure 275. ^{1}H NMR of compound **56** (400 MHz, d_6-DMSO).

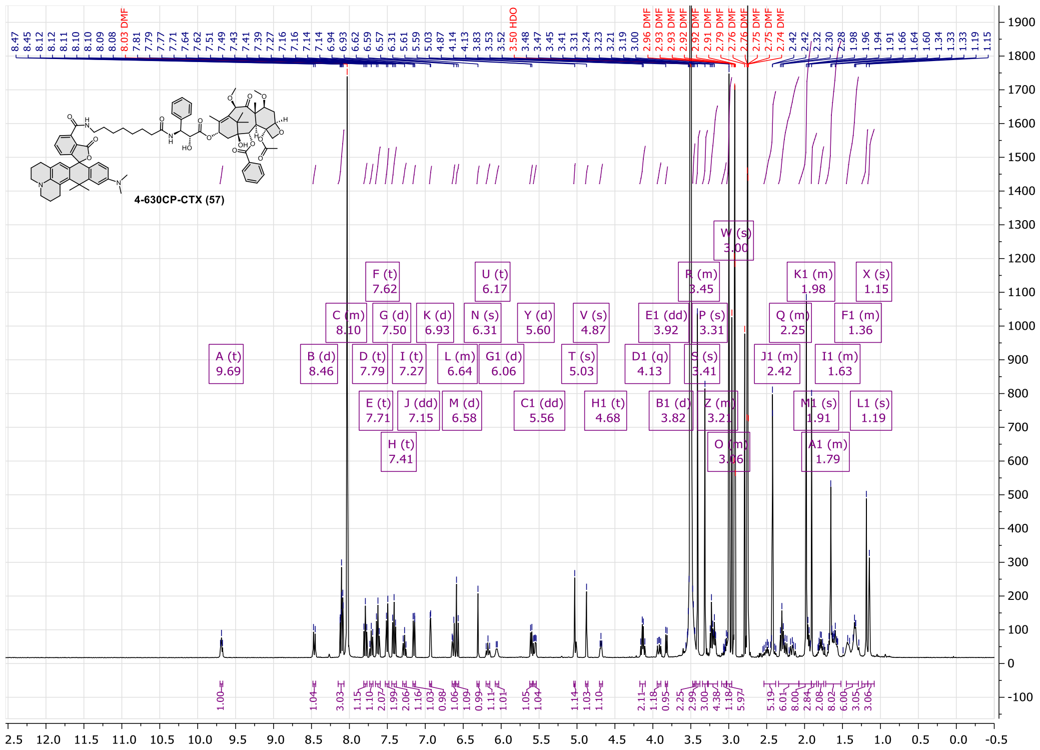

Supplementary Figure 276. ^{1}H NMR of compound **57** (400 MHz, d7-DMF).

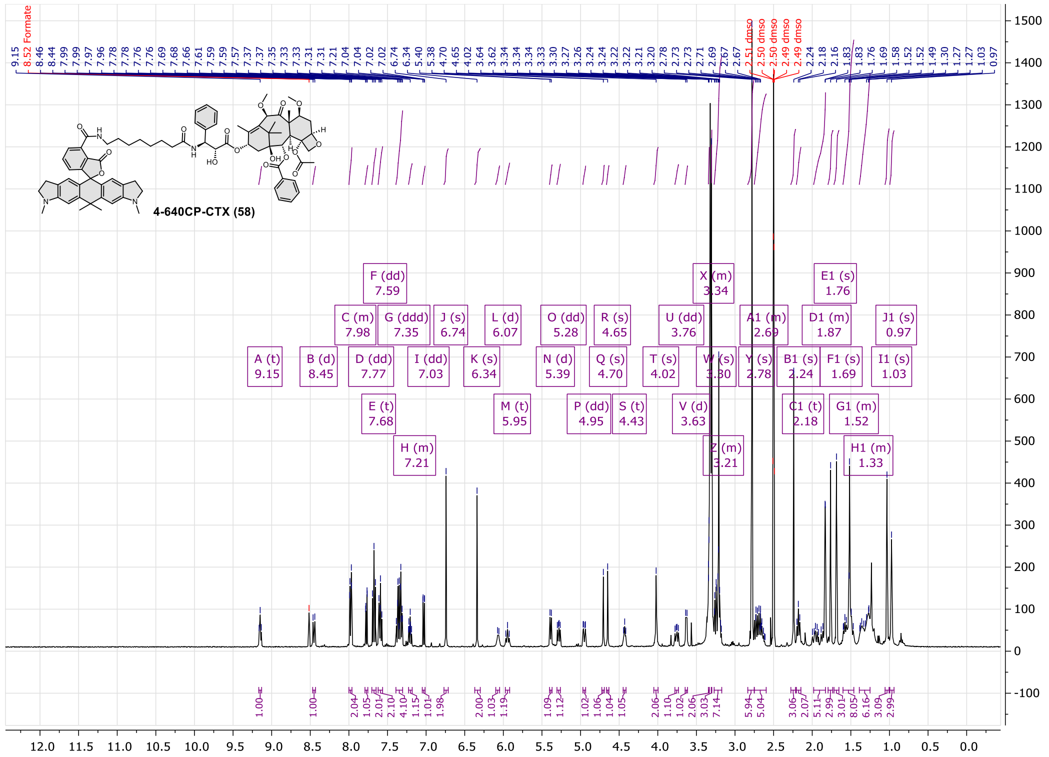

Supplementary Figure 277. ^{1}H NMR of compound **58** (400 MHz, d_6-DMSO).

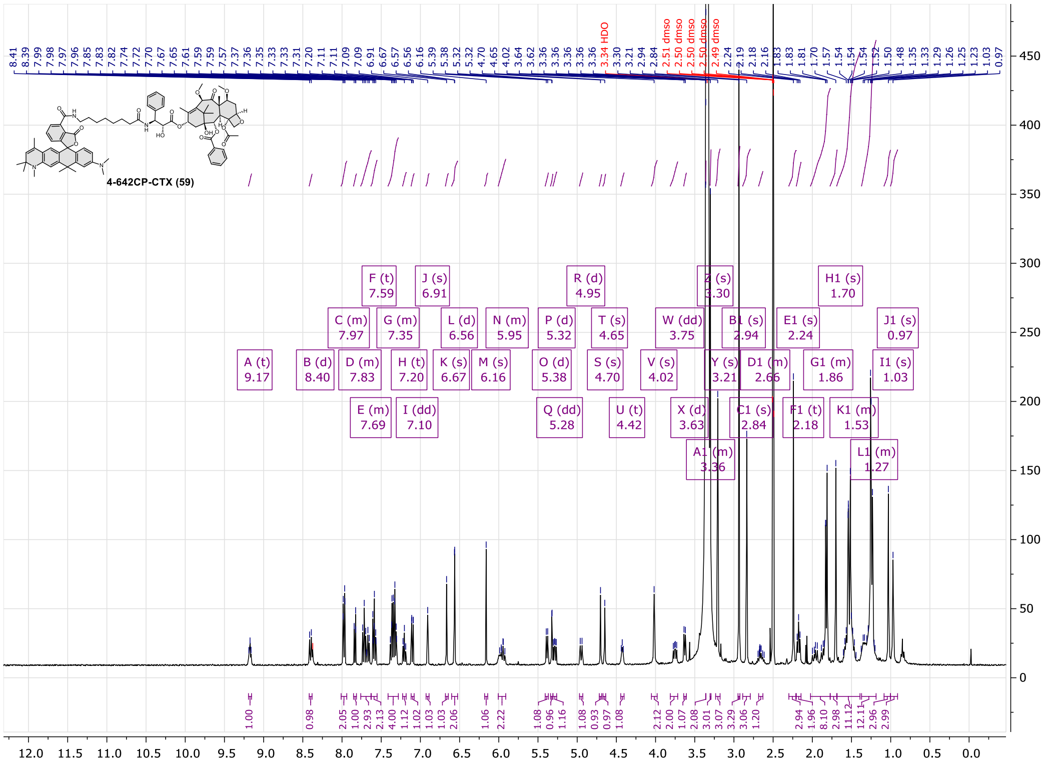

Supplementary Figure 278. ^1H NMR of compound **59** (400 MHz, d_6-DMSO).

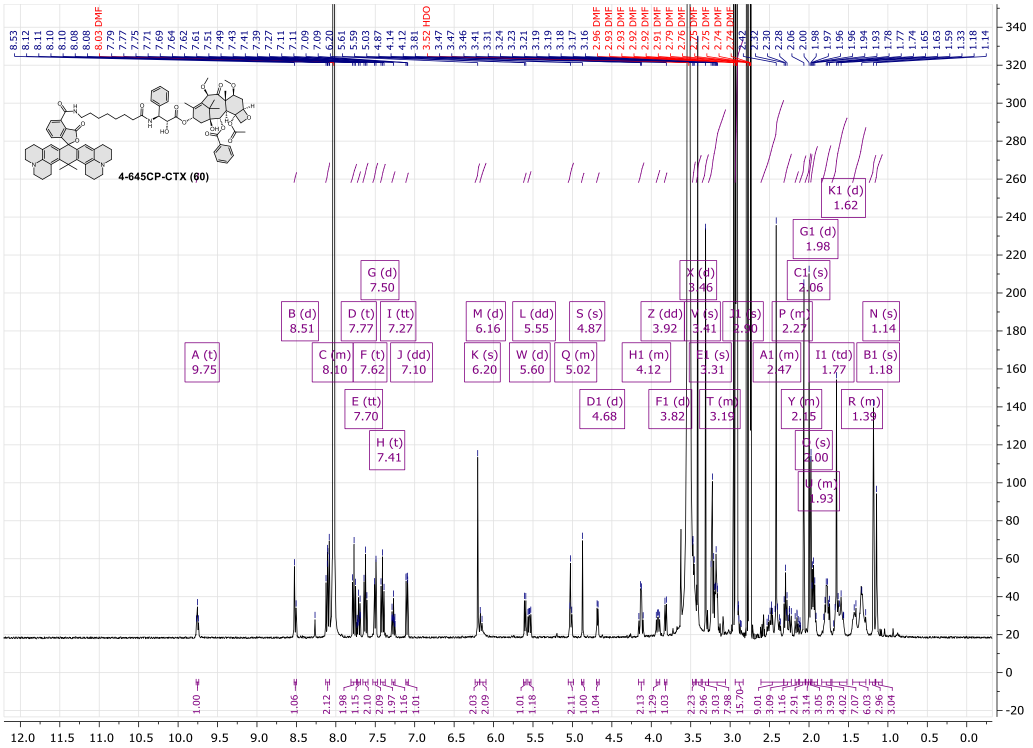

Supplementary Figure 279. ^1H NMR of compound **60** (400 MHz, d_7-DMF).

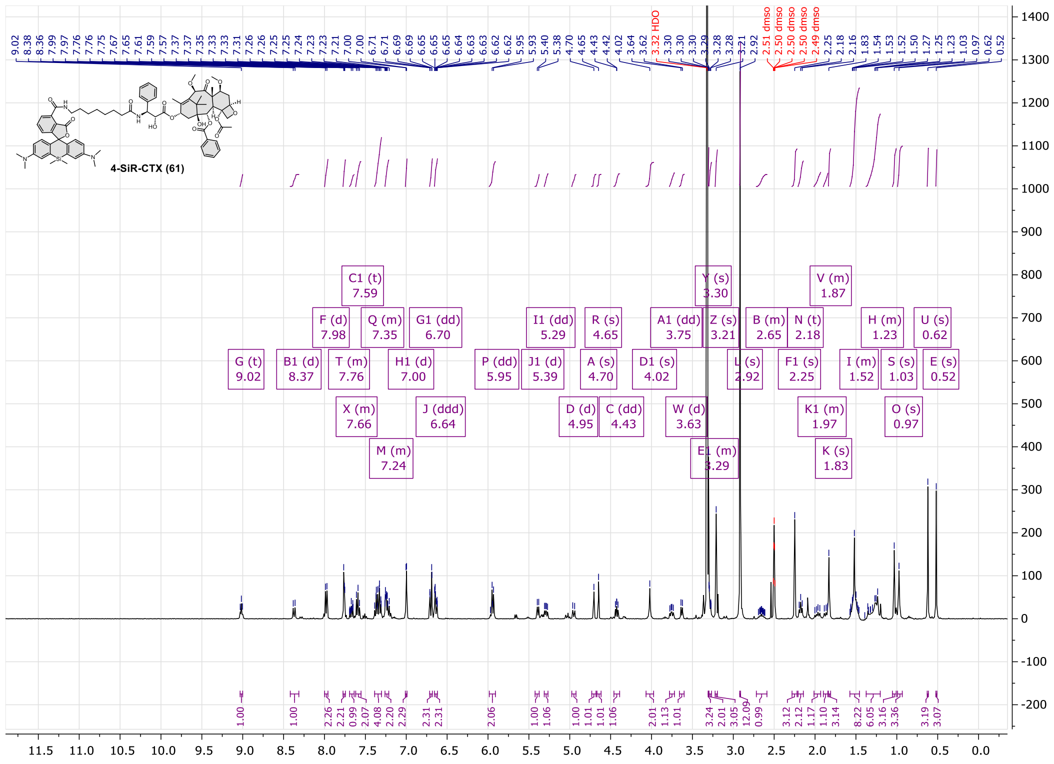

Supplementary Figure 280. ^{1}H NMR of compound **61** (400 MHz, d_6-DMSO).

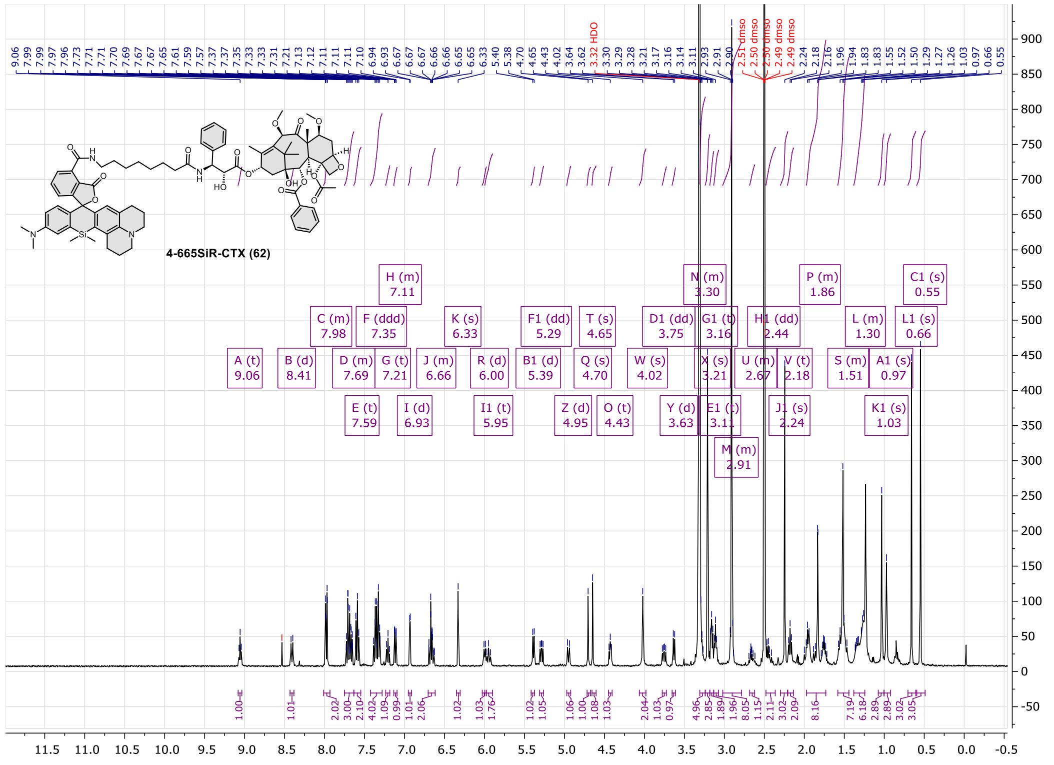

Supplementary Figure 281. ^{1}H NMR of compound **62** (400 MHz, d_6-DMSO).

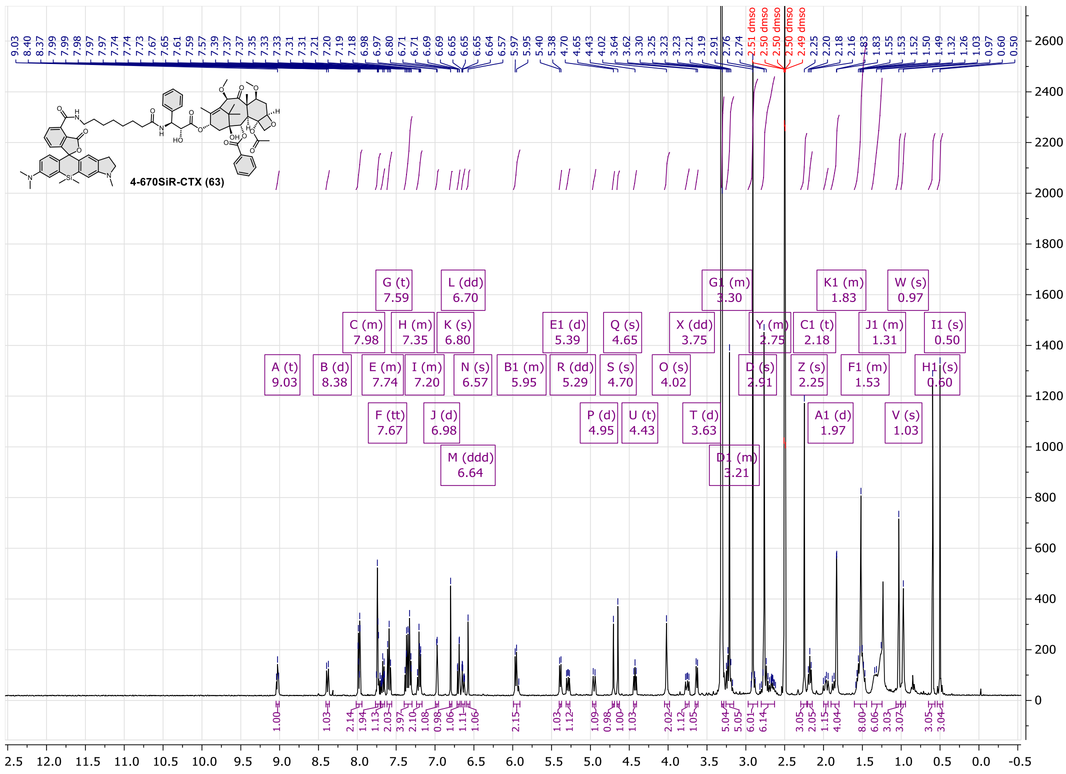

Supplementary Figure 282. ^{1}H NMR of compound **63** (400 MHz, d_6-DMSO).

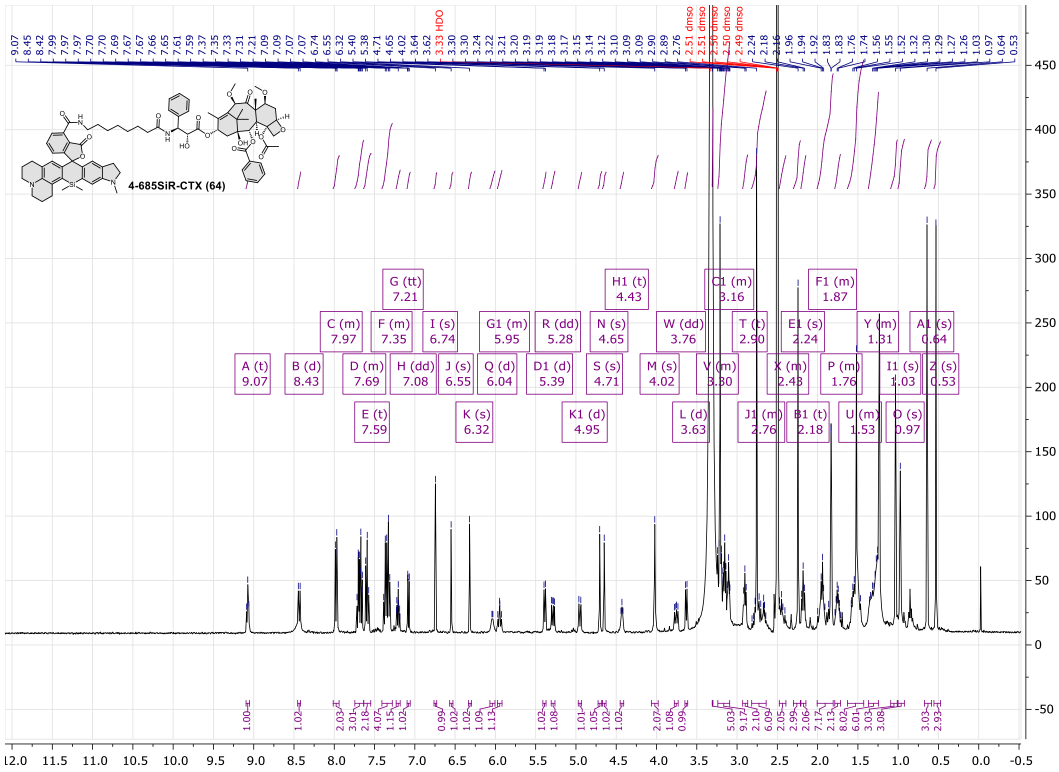

Supplementary Figure 283. ^{1}H NMR of compound **64** (400 MHz, d_6-DMSO).

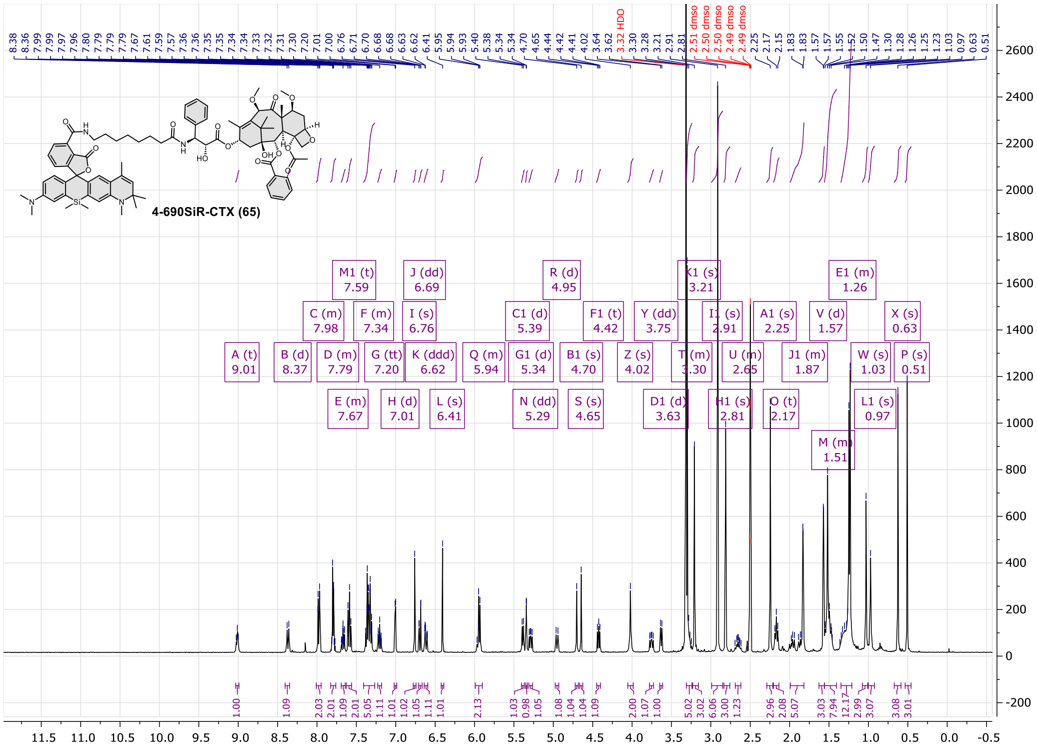

Supplementary Figure 284. ^{1}H NMR of compound **65** (400 MHz, d_6-DMSO).

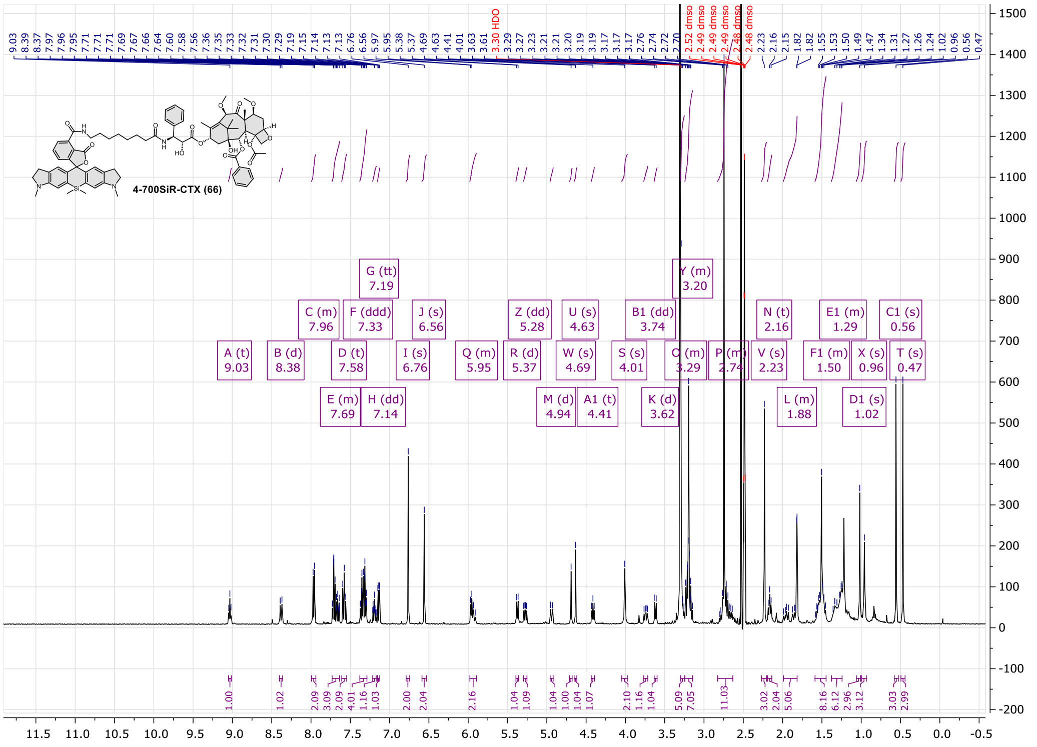

Supplementary Figure 285. ^{1}H NMR of compound **66** (400 MHz, d_6-DMSO).

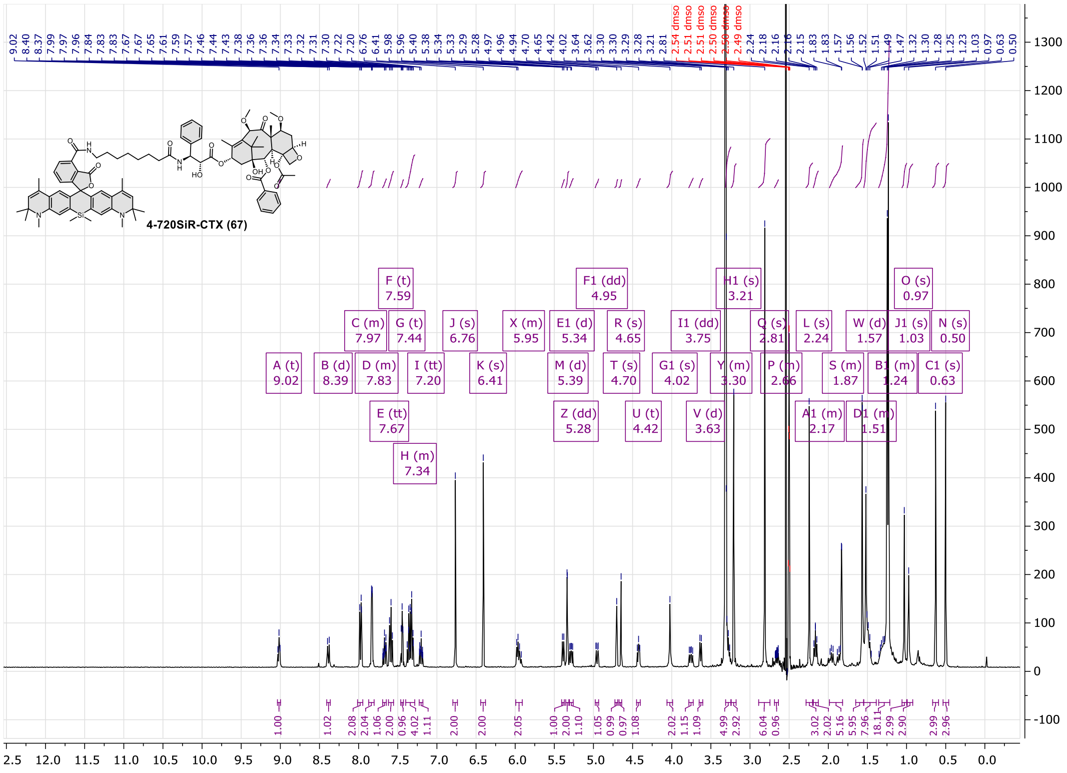

Supplementary Figure 286. ^{1}H NMR of compound **67** (400 MHz, d_6-DMSO).

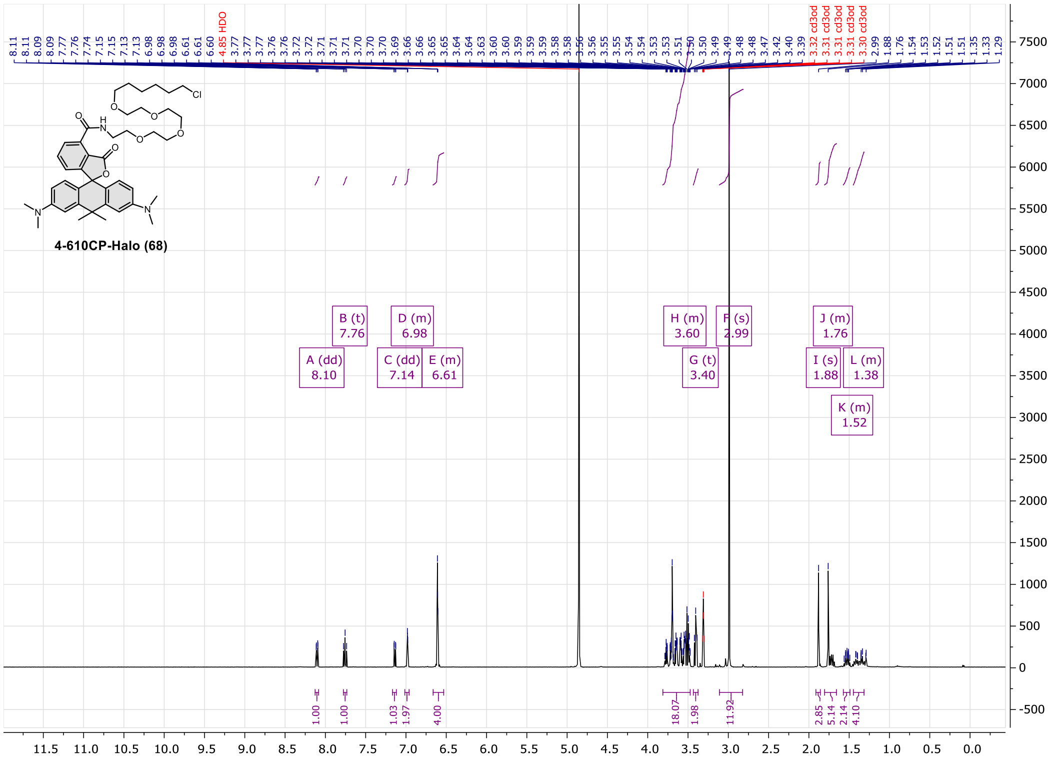

Supplementary Figure 287. ^{1}H NMR of compound **68** (400 MHz, CD_3OD).

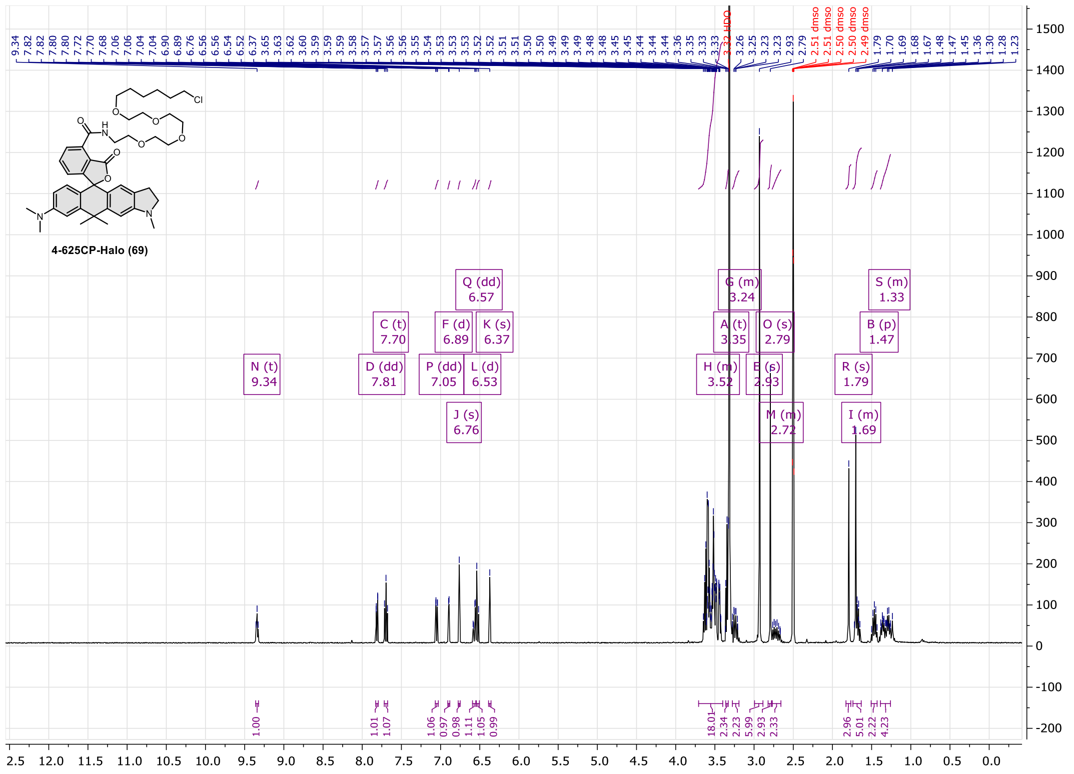

Supplementary Figure 288. ^{1}H NMR of compound **69** (400 MHz, d_6-DMSO).

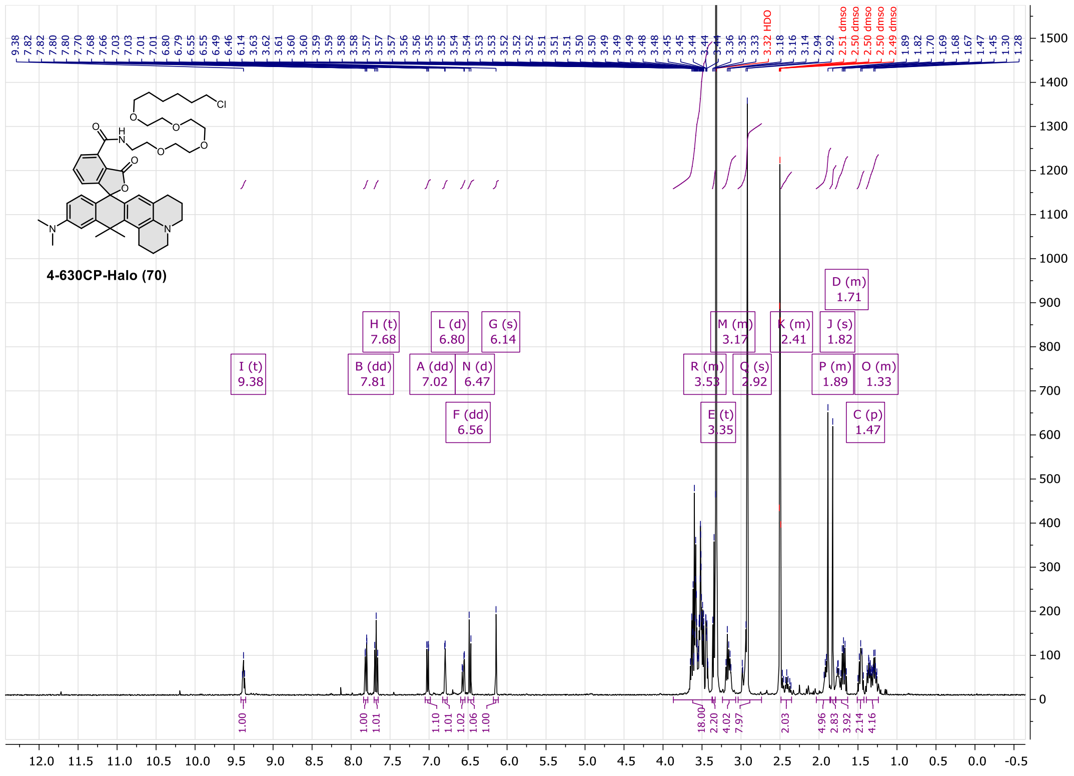

Supplementary Figure 289. ^{1}H NMR of compound **70** (400 MHz, d_6-DMSO).

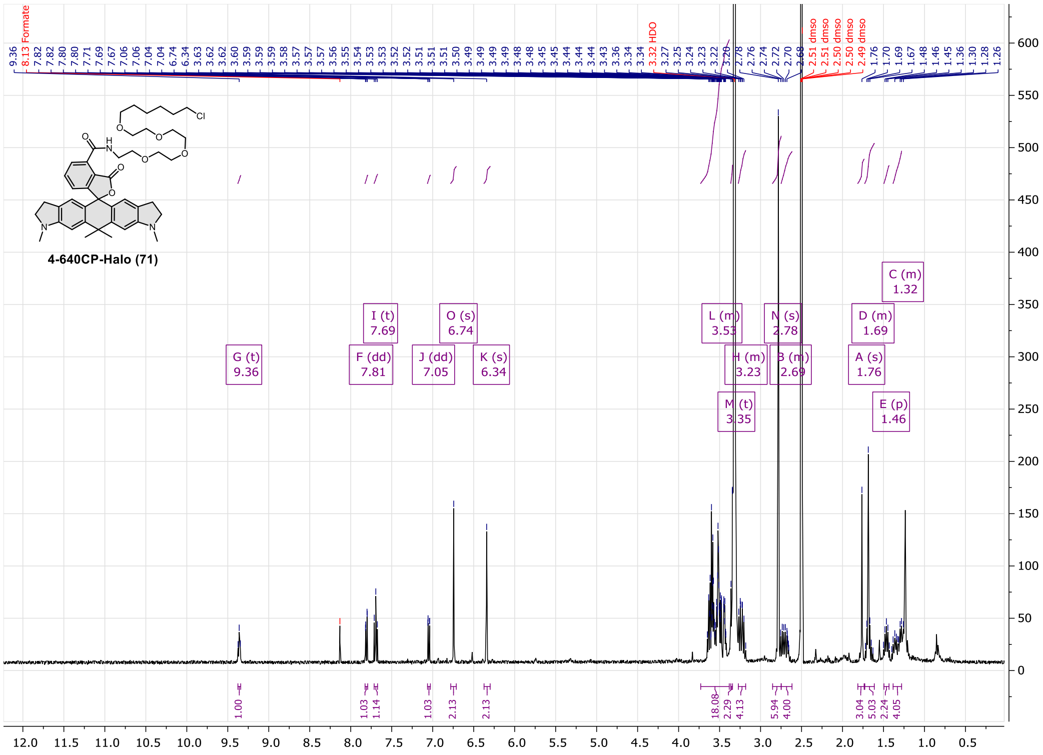

Supplementary Figure 290. ^{1}H NMR of compound **71** (400 MHz, d_6-DMSO).

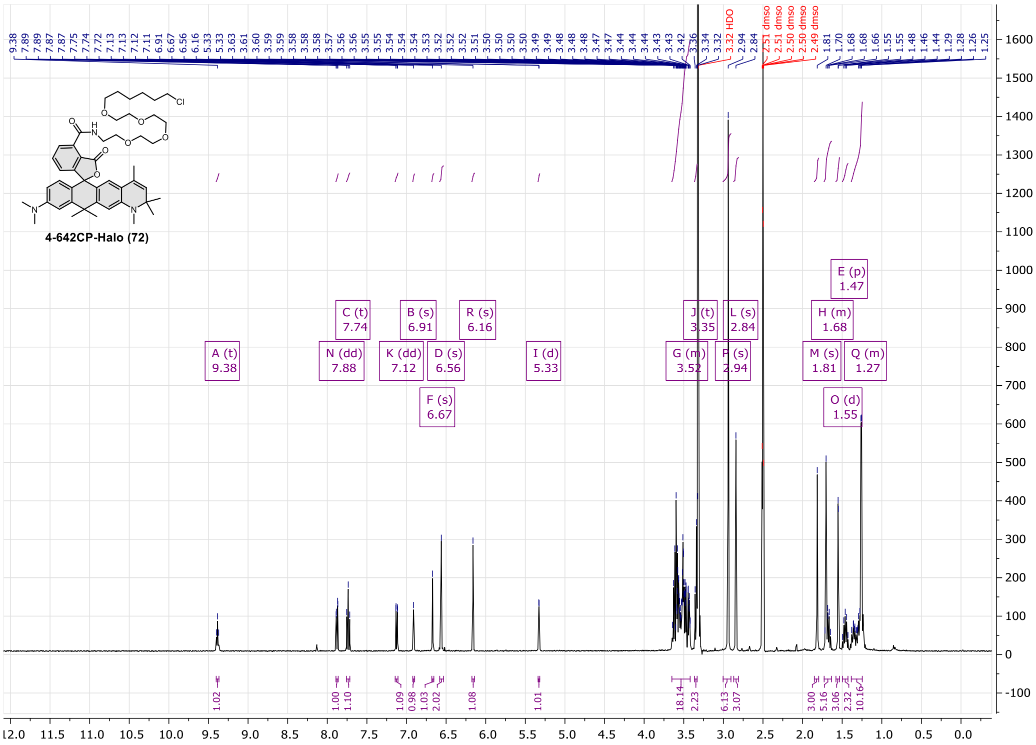

Supplementary Figure 291. ^{1}H NMR of compound **72** (400 MHz, d_6-DMSO).

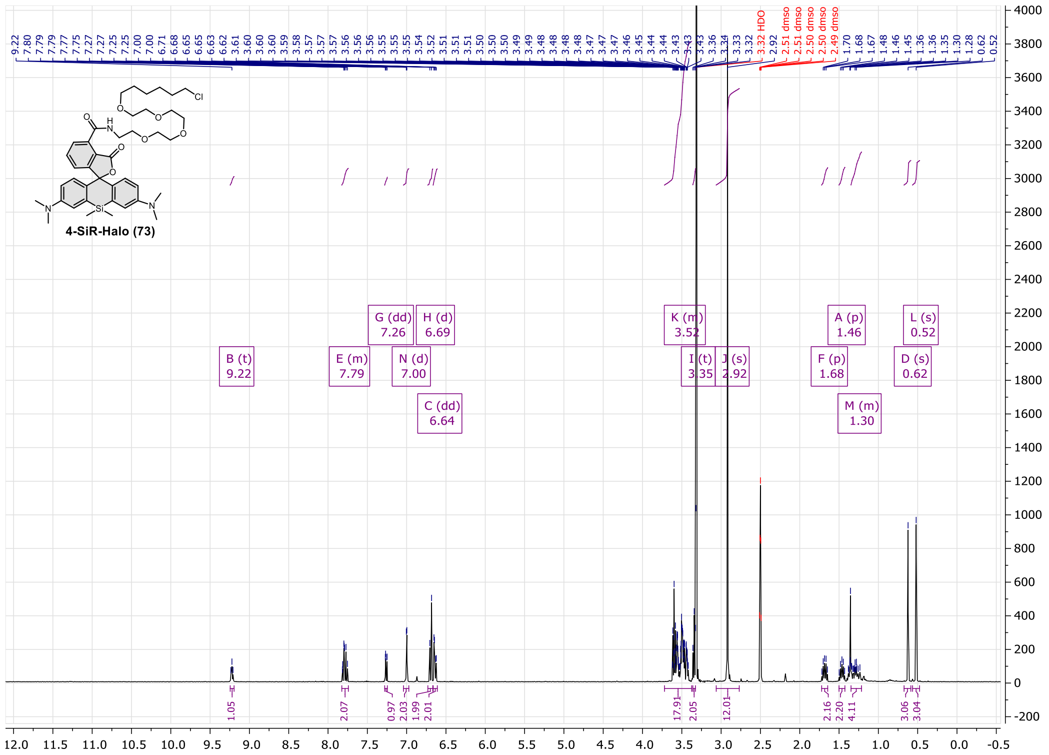

Supplementary Figure 292. ^{1}H NMR of compound **73** (400 MHz, d_6-DMSO).

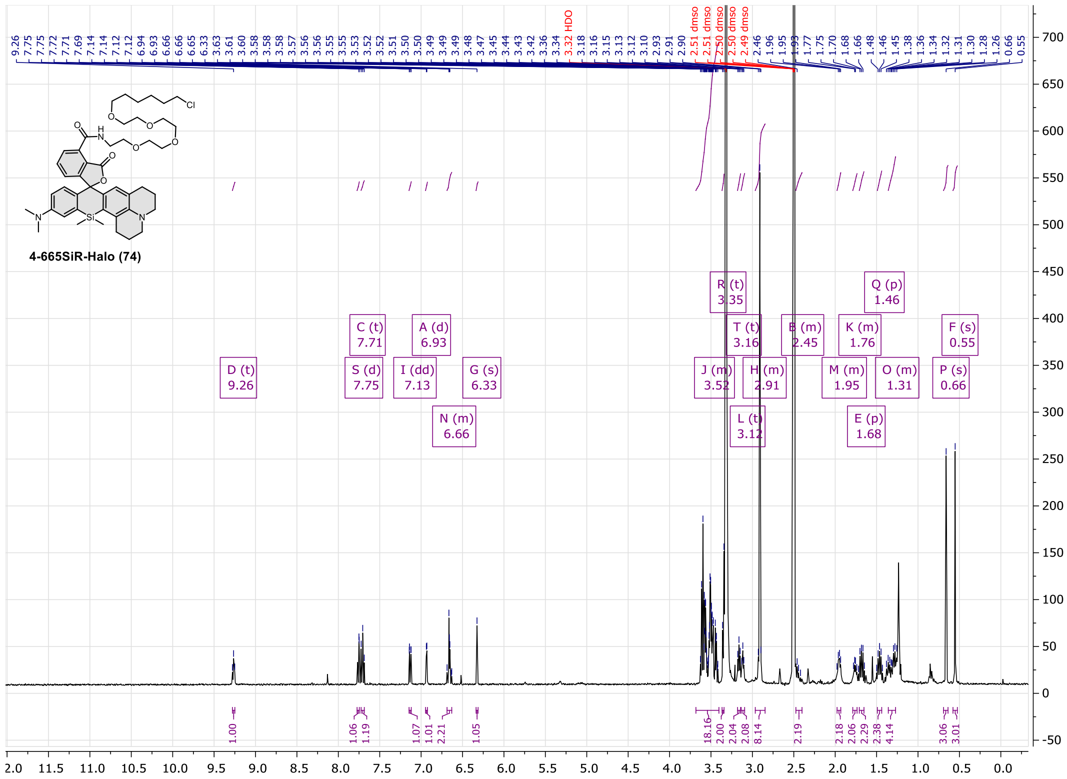

Supplementary Figure 293. ^{1}H NMR of compound **74** (400 MHz, d_6-DMSO).

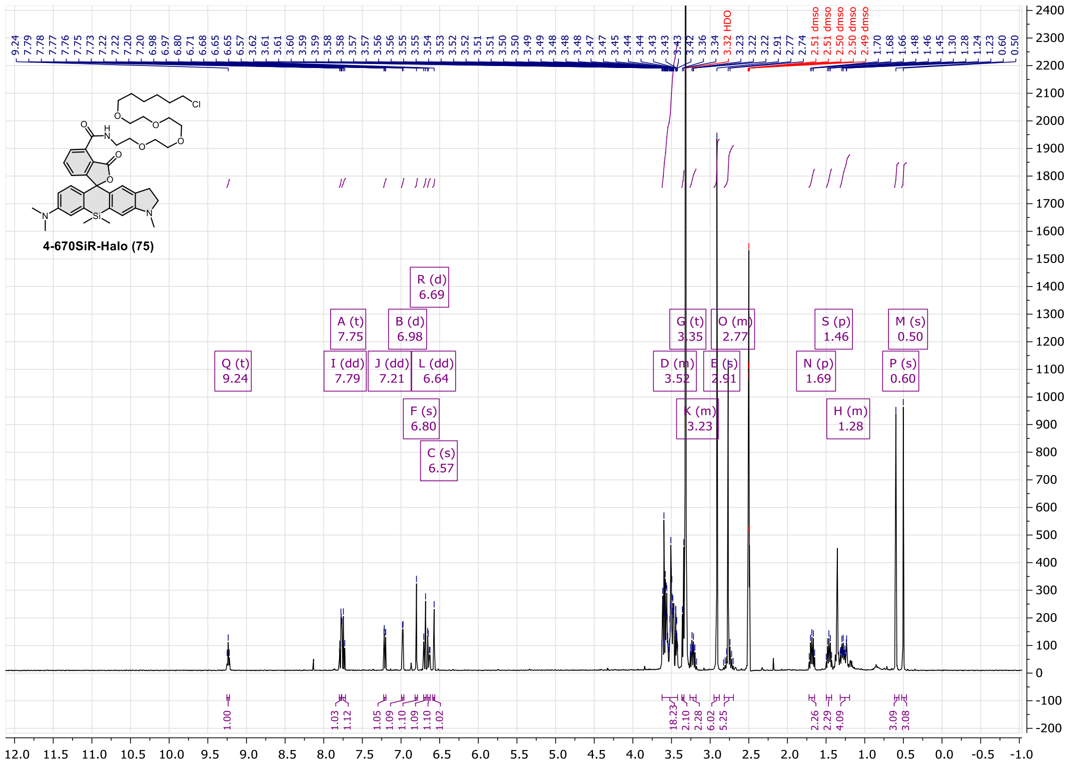

Supplementary Figure 294. ^{1}H NMR of compound **75** (400 MHz, d_6-DMSO).

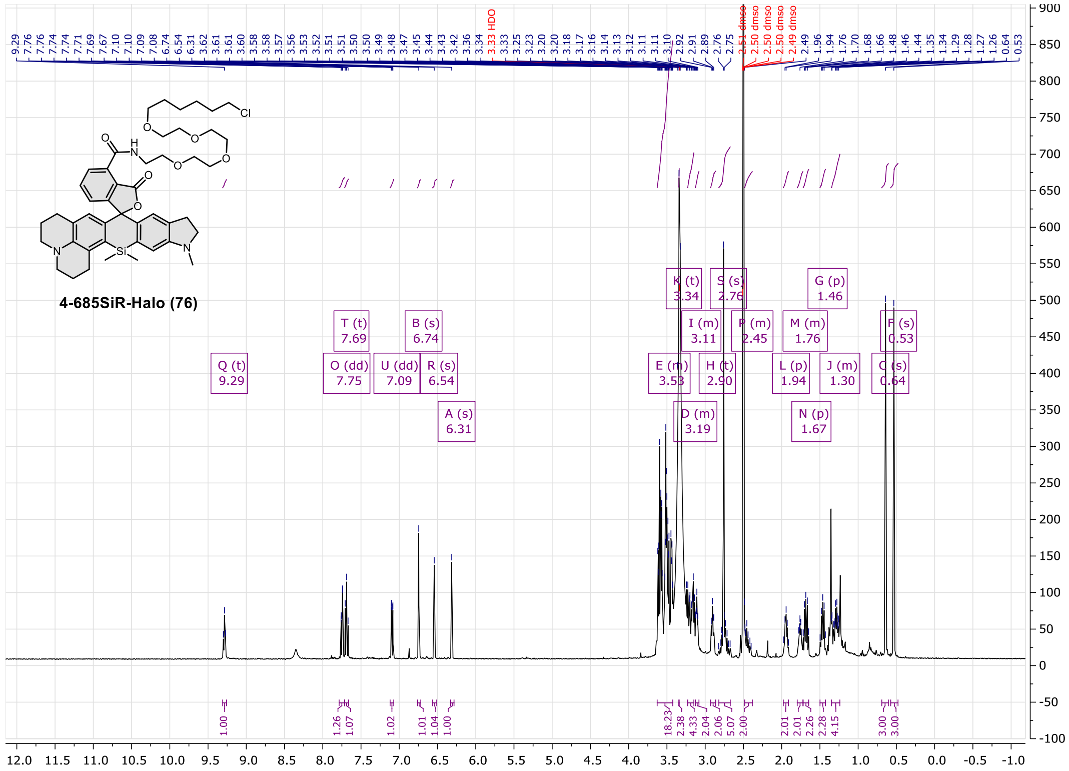

Supplementary Figure 295. ^{1}H NMR of compound **76** (400 MHz, d_6-DMSO).

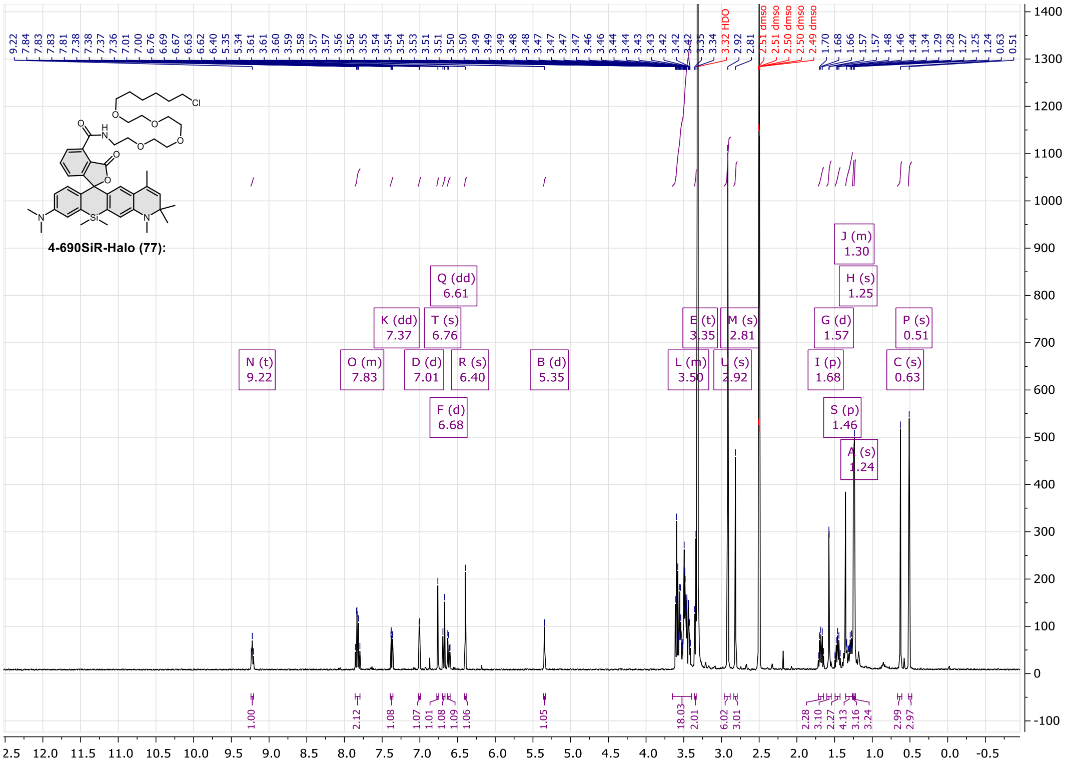

Supplementary Figure 296. ^{1}H NMR of compound **77** (400 MHz, d_6-DMSO).

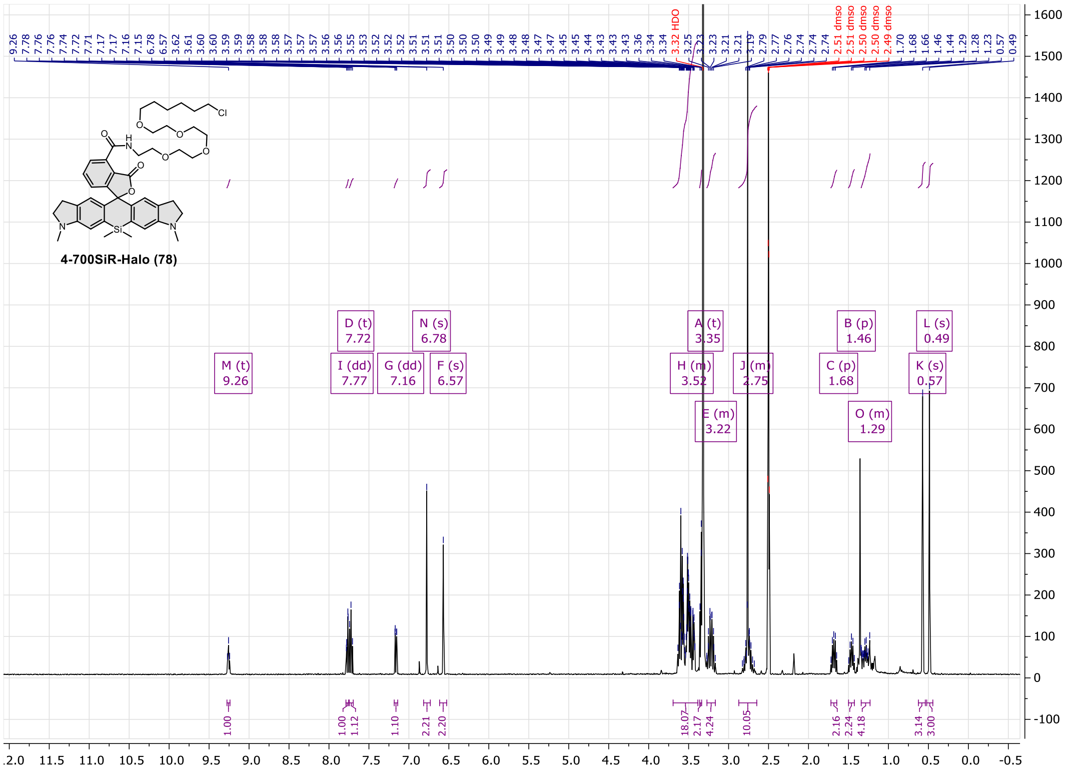

Supplementary Figure 297. ^{1}H NMR of compound **78** (400 MHz, d_6-DMSO).

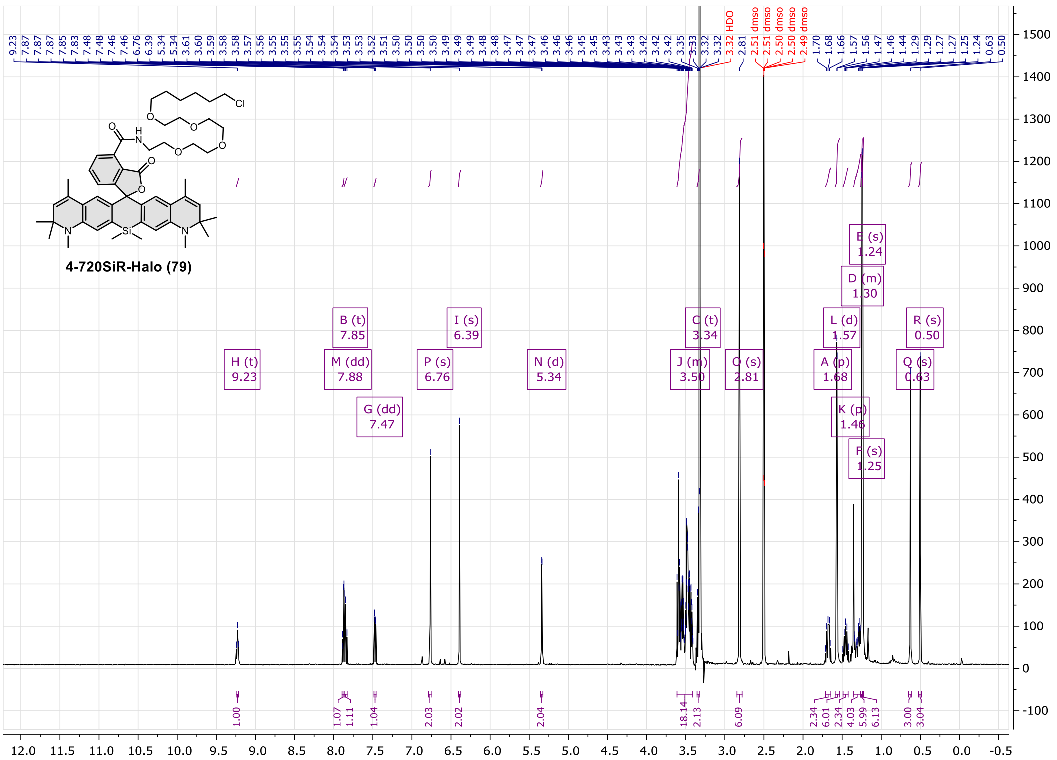

Supplementary Figure 298. ^{1}H NMR of compound **79** (400 MHz, d_6-DMSO).

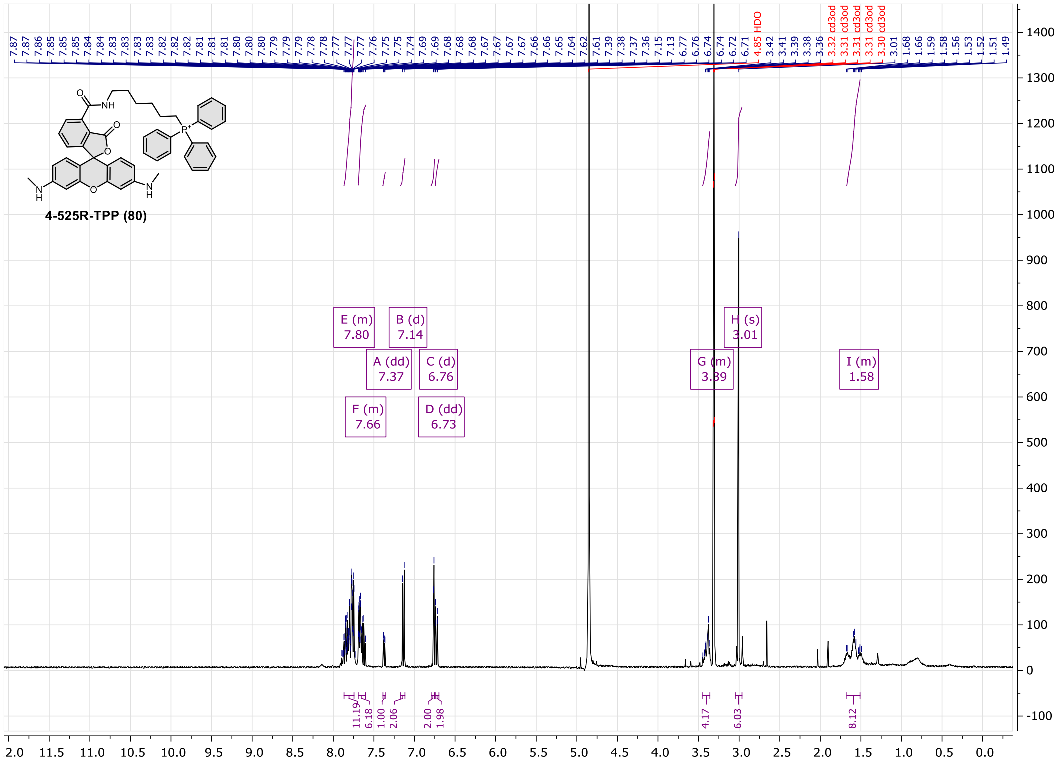

Supplementary Figure 299. ^{1}H NMR of compound **80** (400 MHz, CD_3OD).

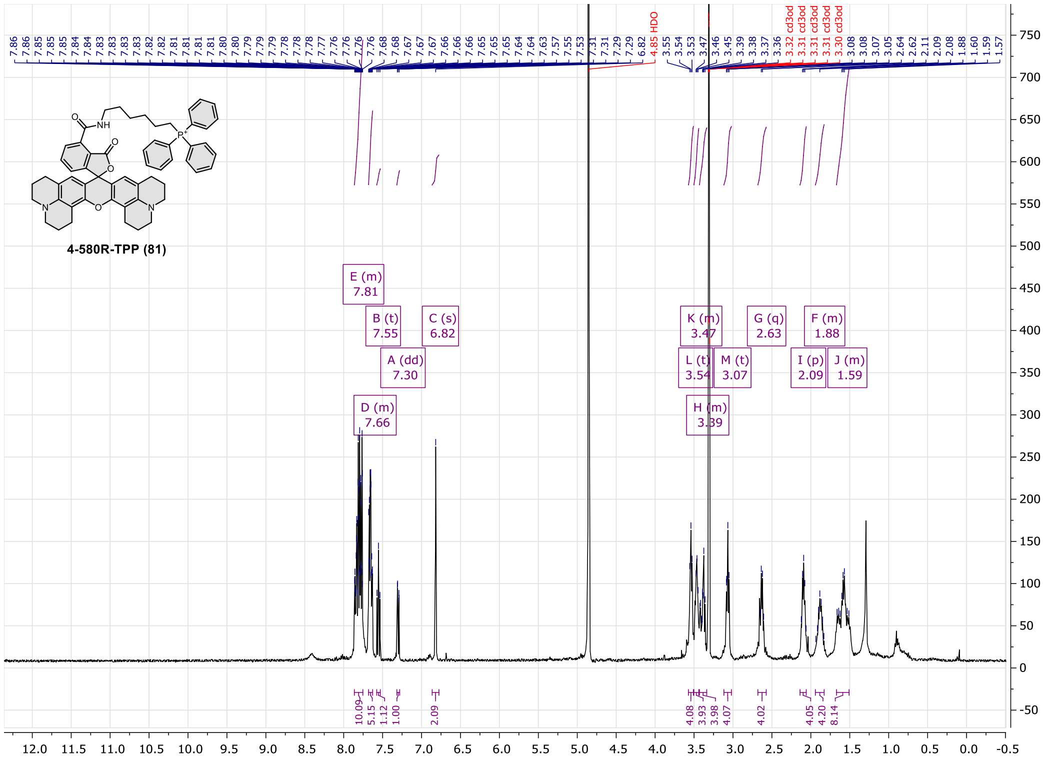

Supplementary Figure 300. ^{1}H NMR of compound **81** (400 MHz, CD_3OD).

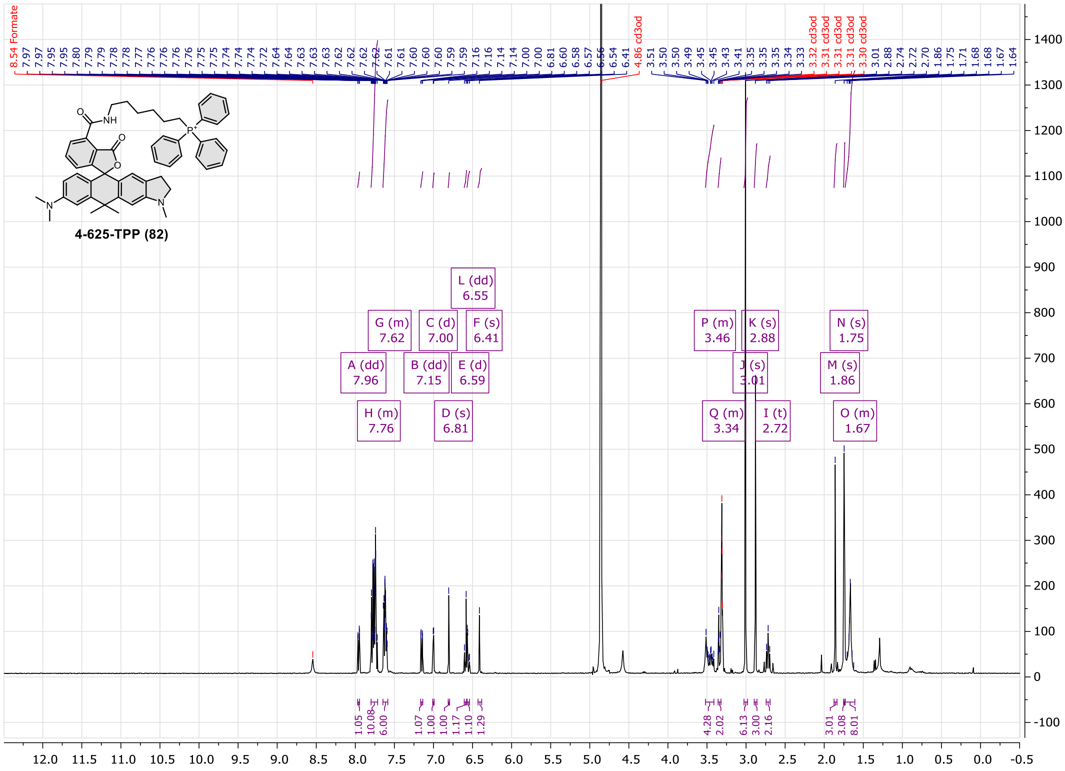

Supplementary Figure 301. ^{1}H NMR of compound **82** (400 MHz, CD_3OD).

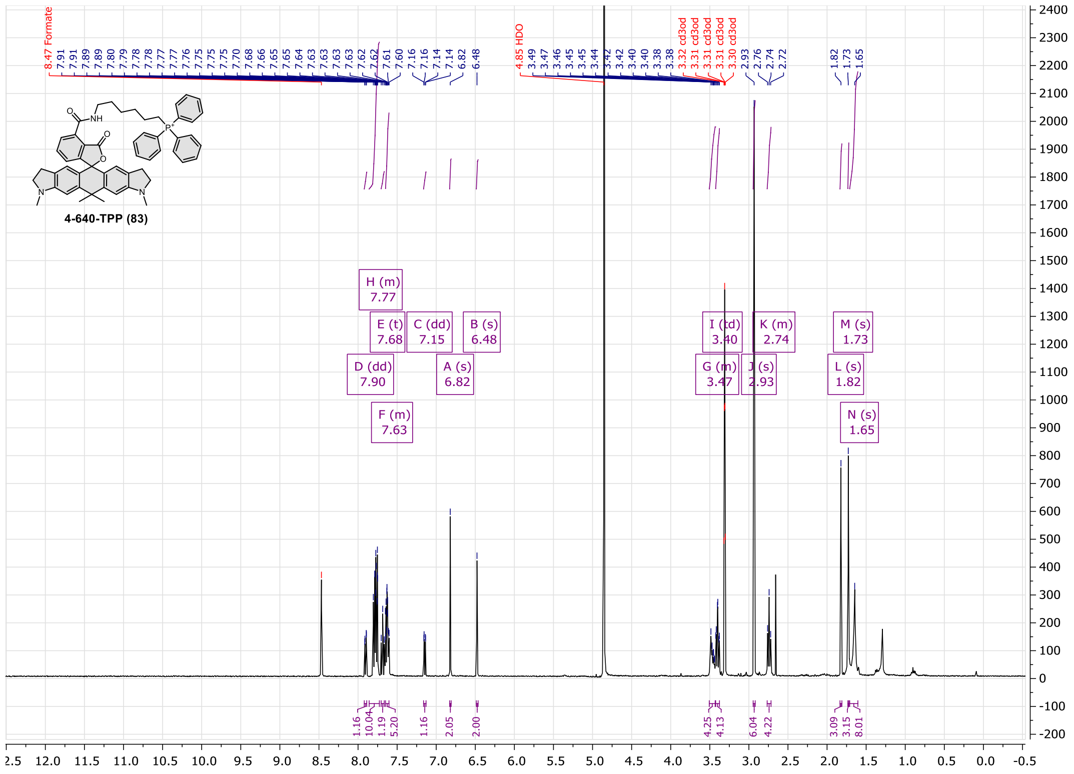

Supplementary Figure 302. ^{1}H NMR of compound **83** (400 MHz, CD_3OD).

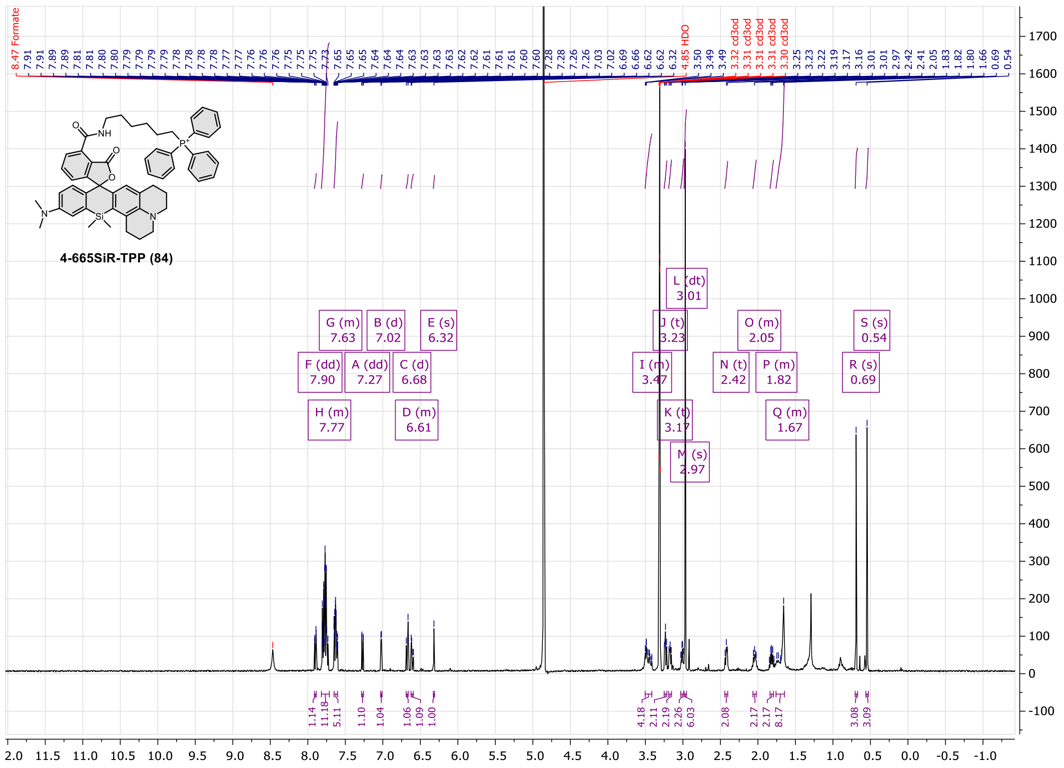

Supplementary Figure 303. ^{1}H NMR of compound **84** (400 MHz, CD_3OD).

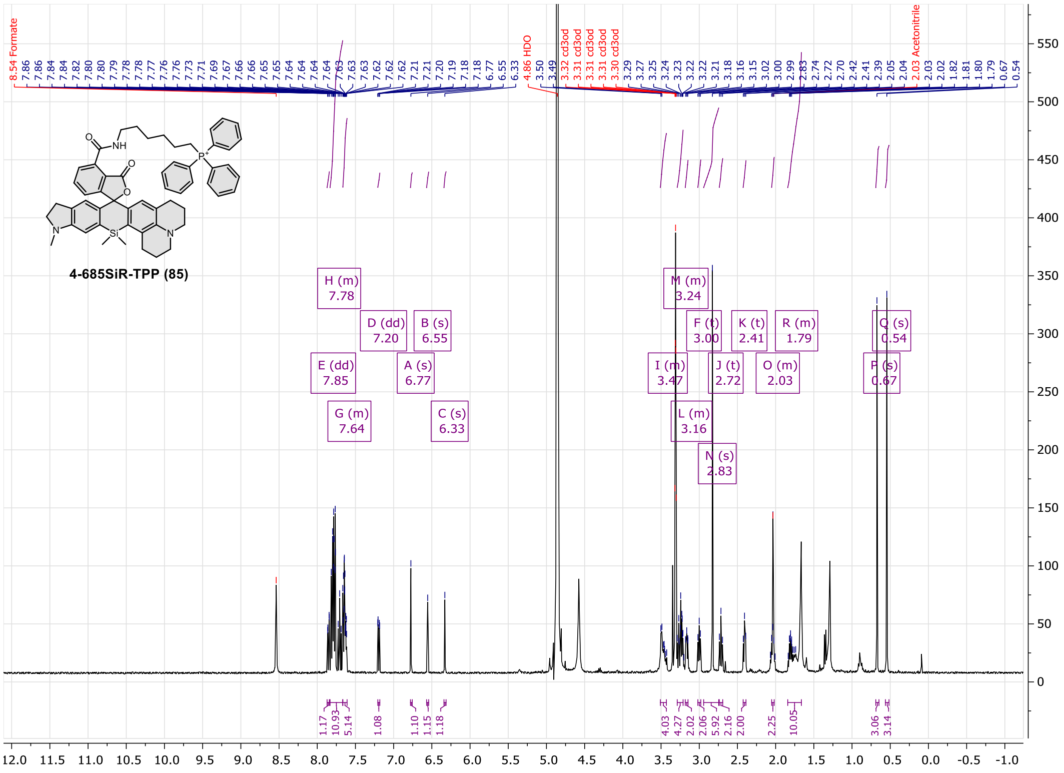

Supplementary Figure 304. ^{1}H NMR of compound **85** (400 MHz, CD_3OD).

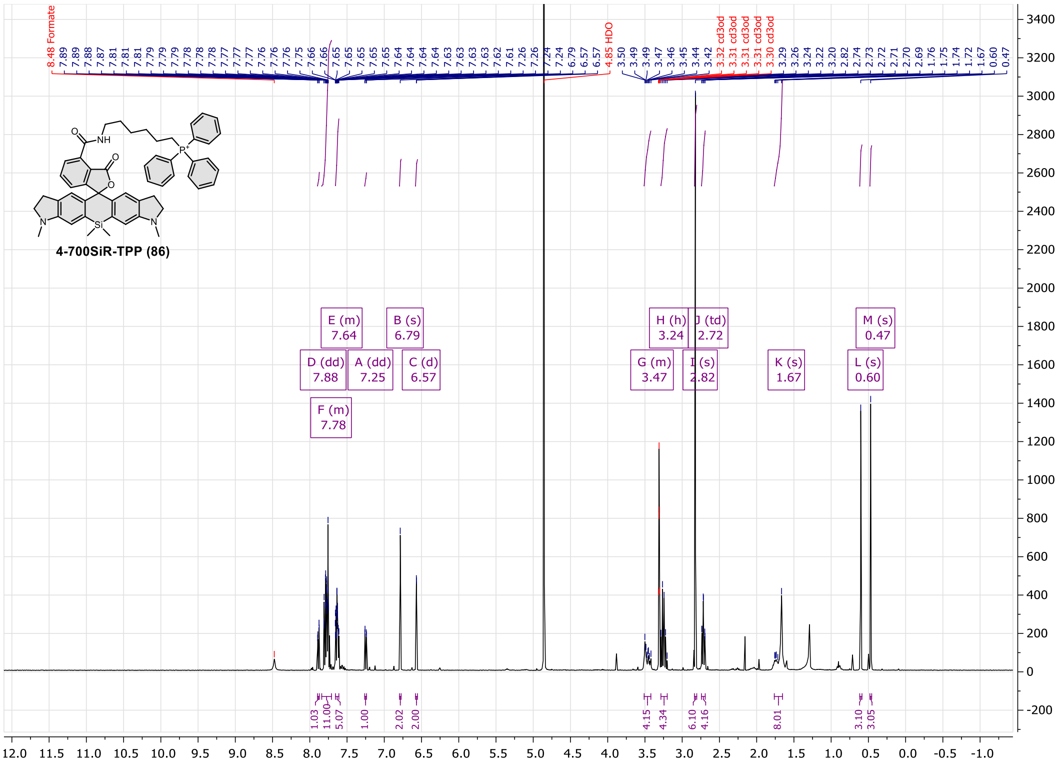

Supplementary Figure 305. ^{1}H NMR of compound **86** (400 MHz, CD_3OD).

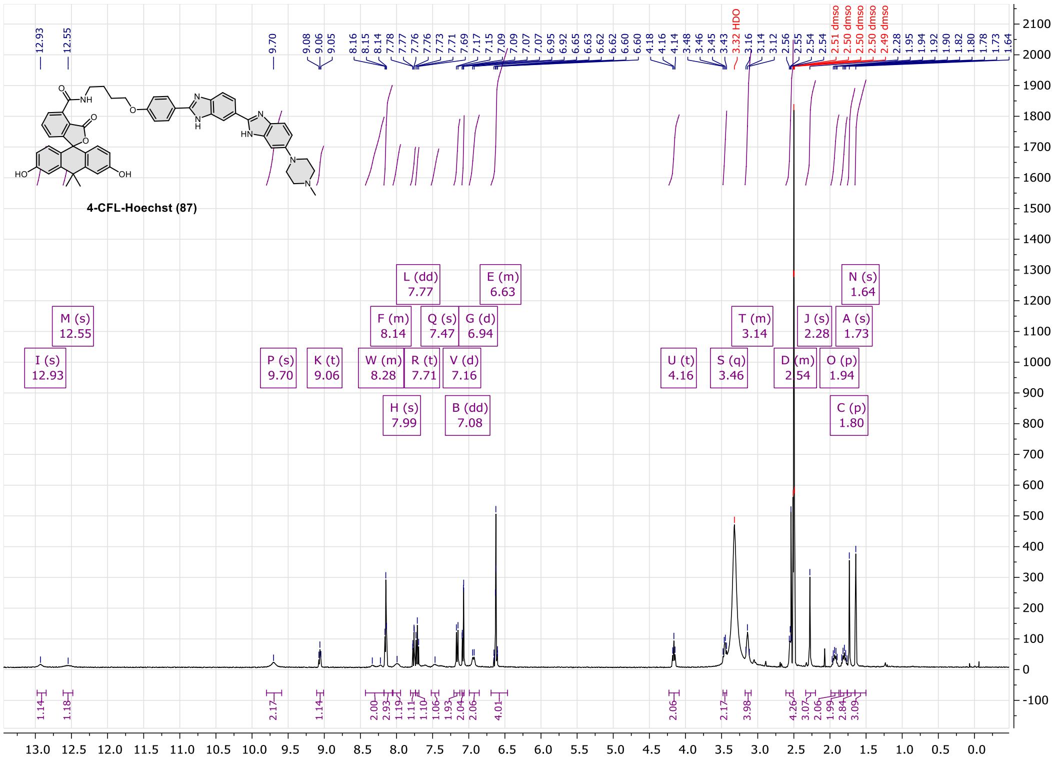

Supplementary Figure 306. ^{1}H NMR of compound **87** (400 MHz, d_6-DMSO).

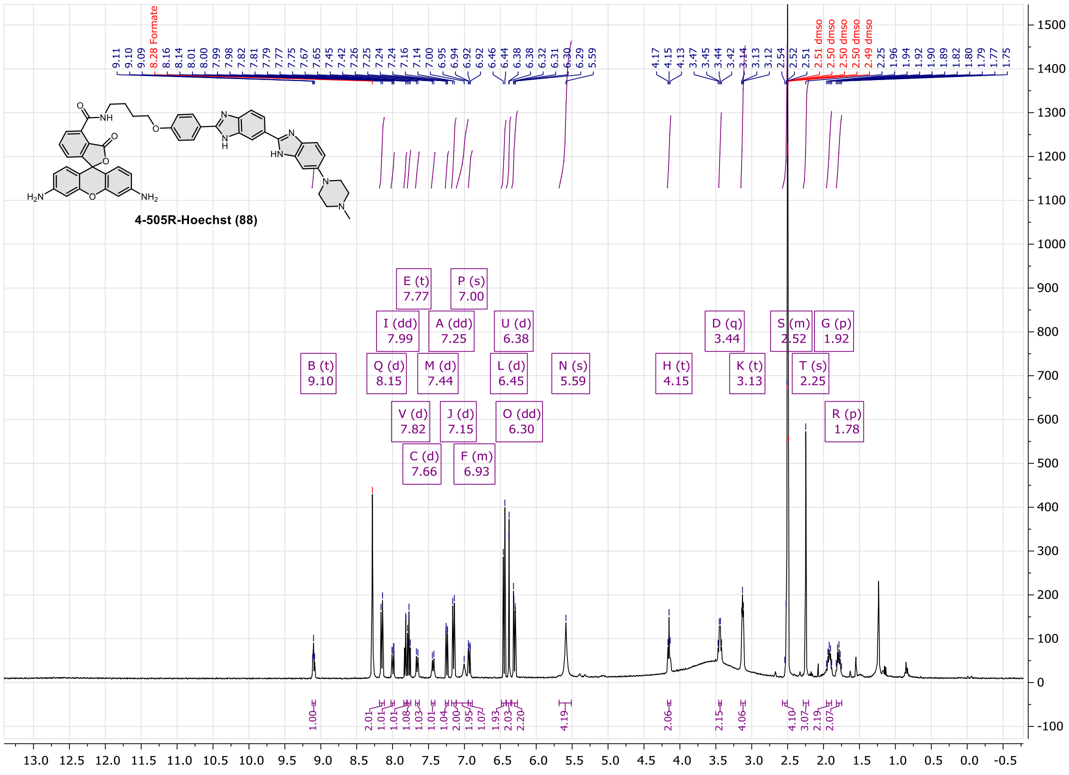

Supplementary Figure 307. ^{1}H NMR of compound **88** (400 MHz, d_6-DMSO).

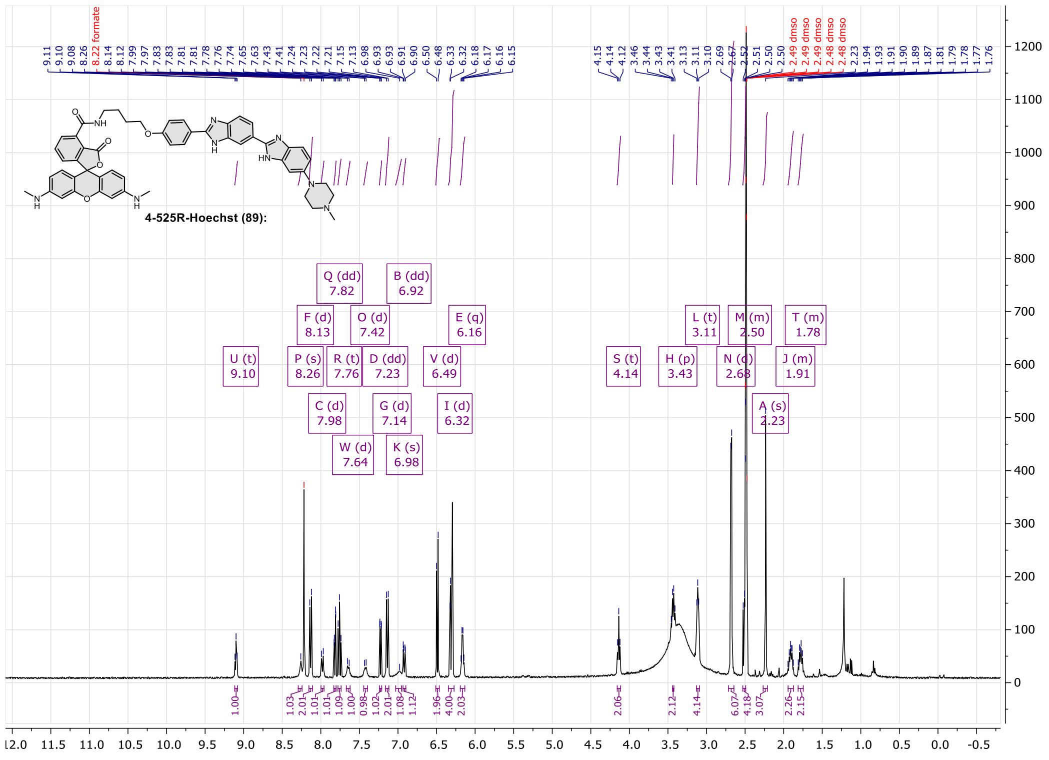

Supplementary Figure 308. ^{1}H NMR of compound **89** (400 MHz, d_6-DMSO).

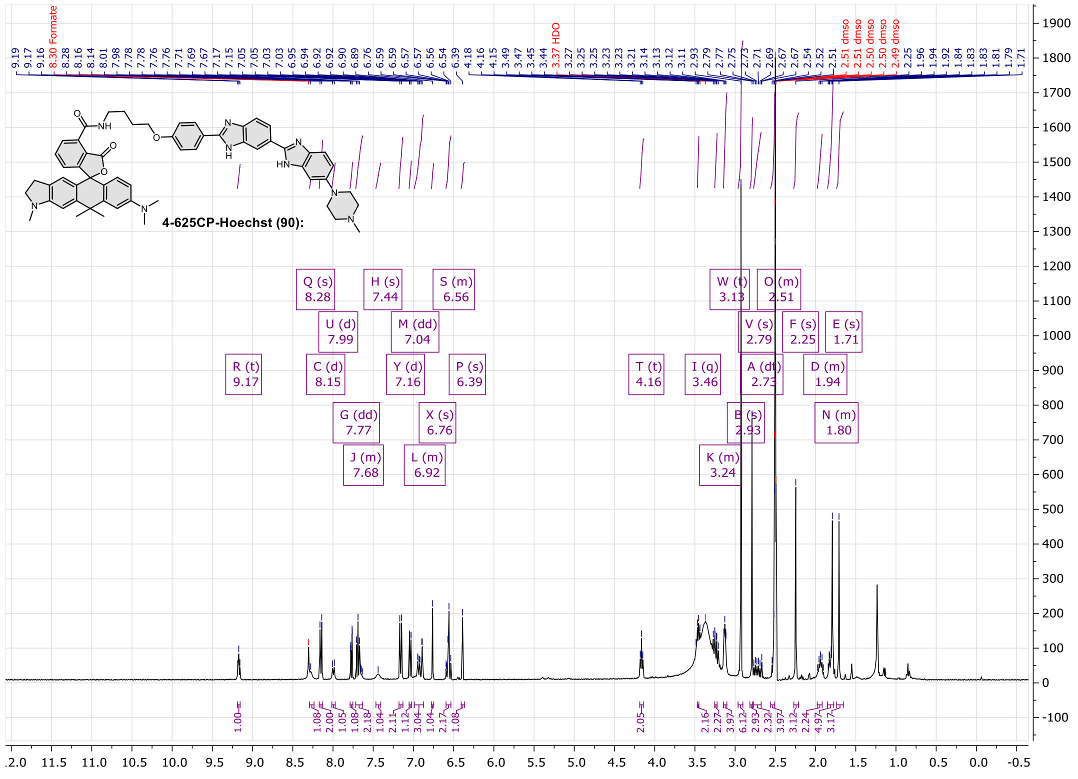

Supplementary Figure 309. ^{1}H NMR of compound **90** (400 MHz, d_6-DMSO).

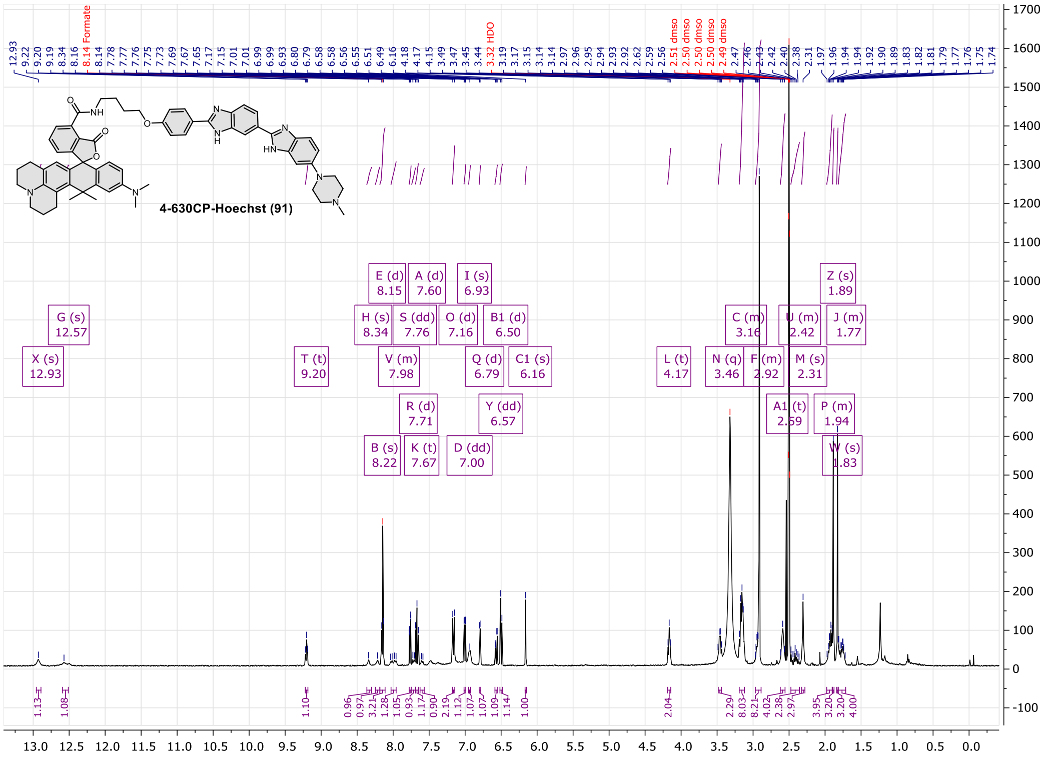

Supplementary Figure 310. ^{1}H NMR of compound **91** (400 MHz, d_6-DMSO).

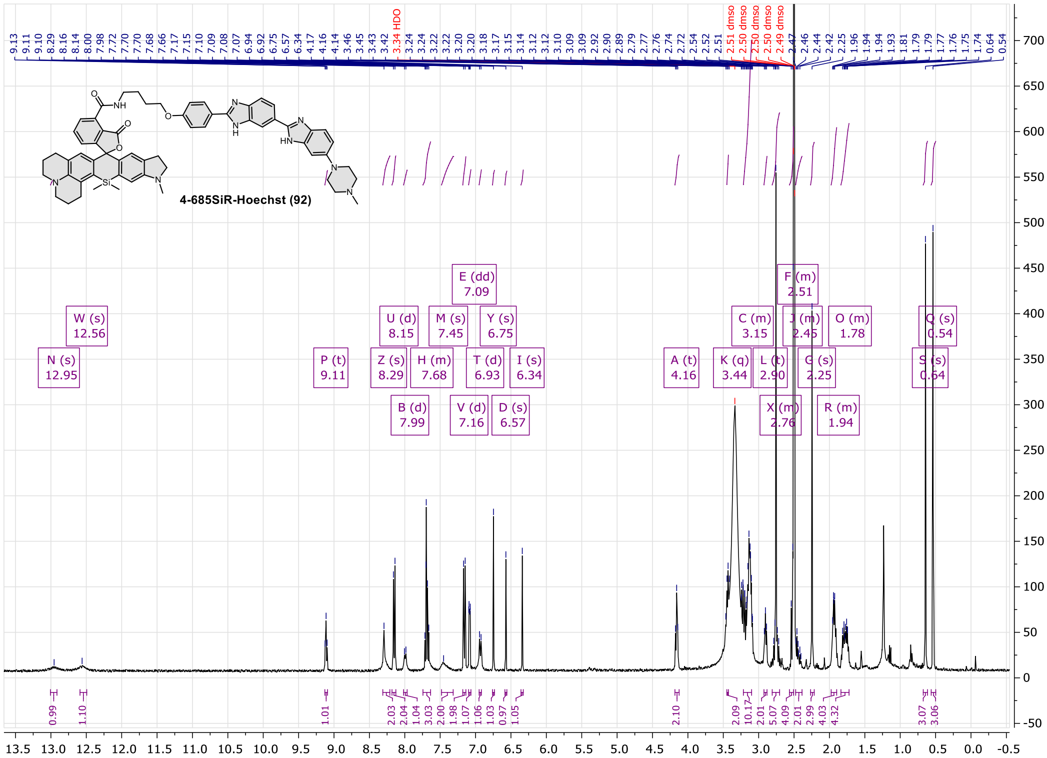

Supplementary Figure 311. ^{1}H NMR of compound **92** (400 MHz, d_6-DMSO).

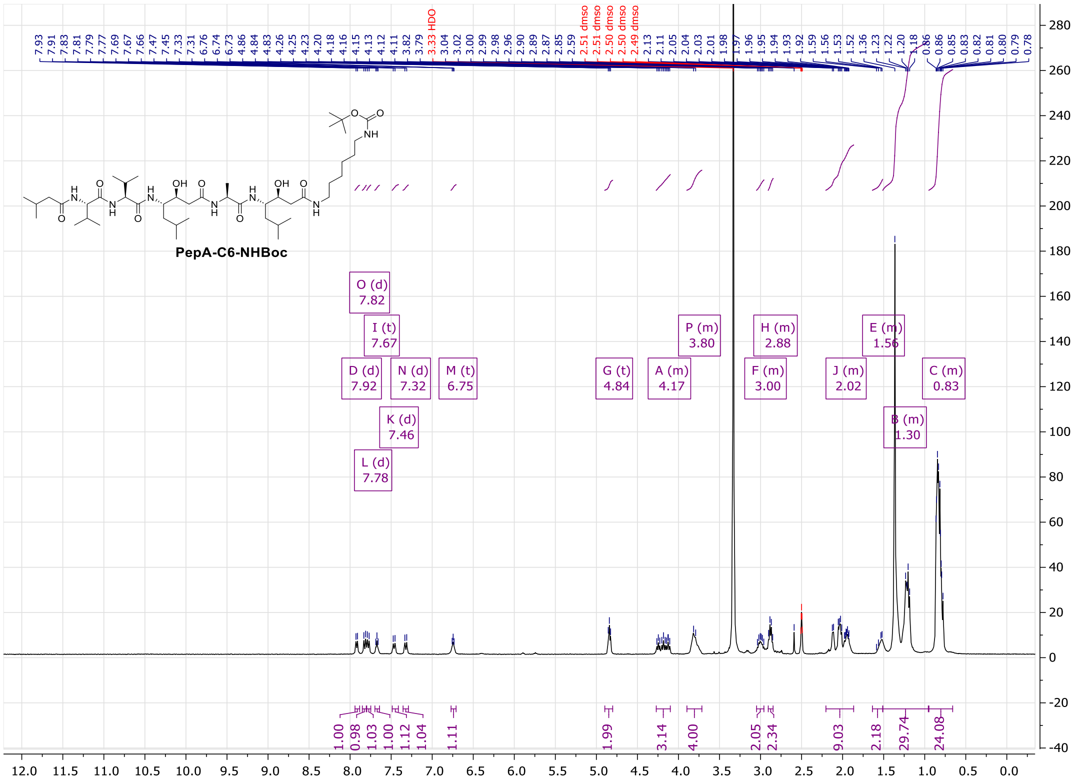

Supplementary Figure 312. ^{1}H NMR of compound **PepA-C6-NHBoc** (400 MHz, d_6-DMSO).

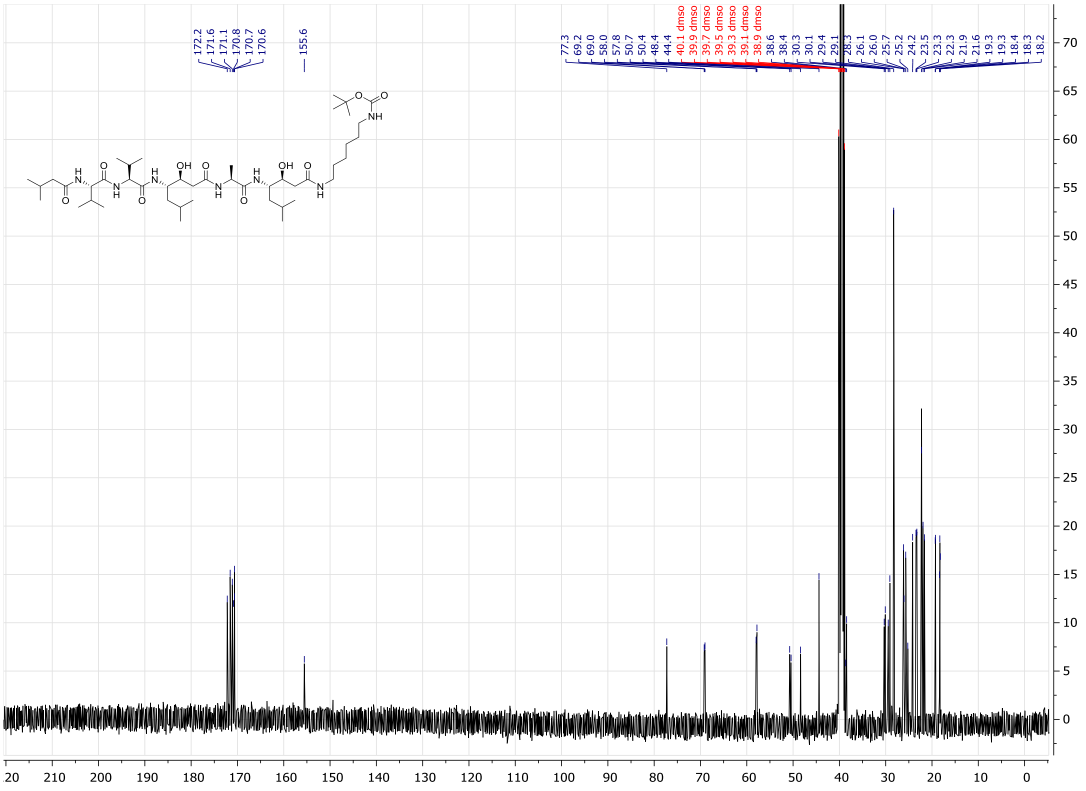

Supplementary Figure 313. ^{13}C NMR of compound **PepA-C6-NHBoc**. (100 MHz, d_6-DMSO)

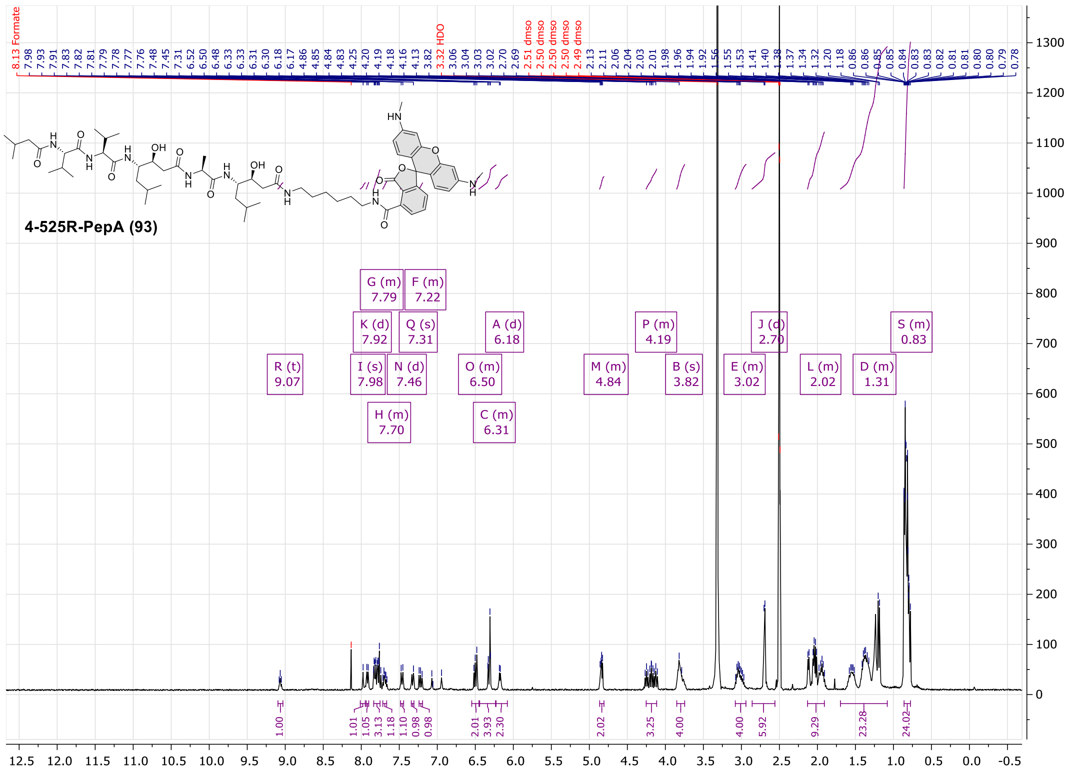

Supplementary Figure 314. ^{1}H NMR of compound **93** (400 MHz, d_6-DMSO).

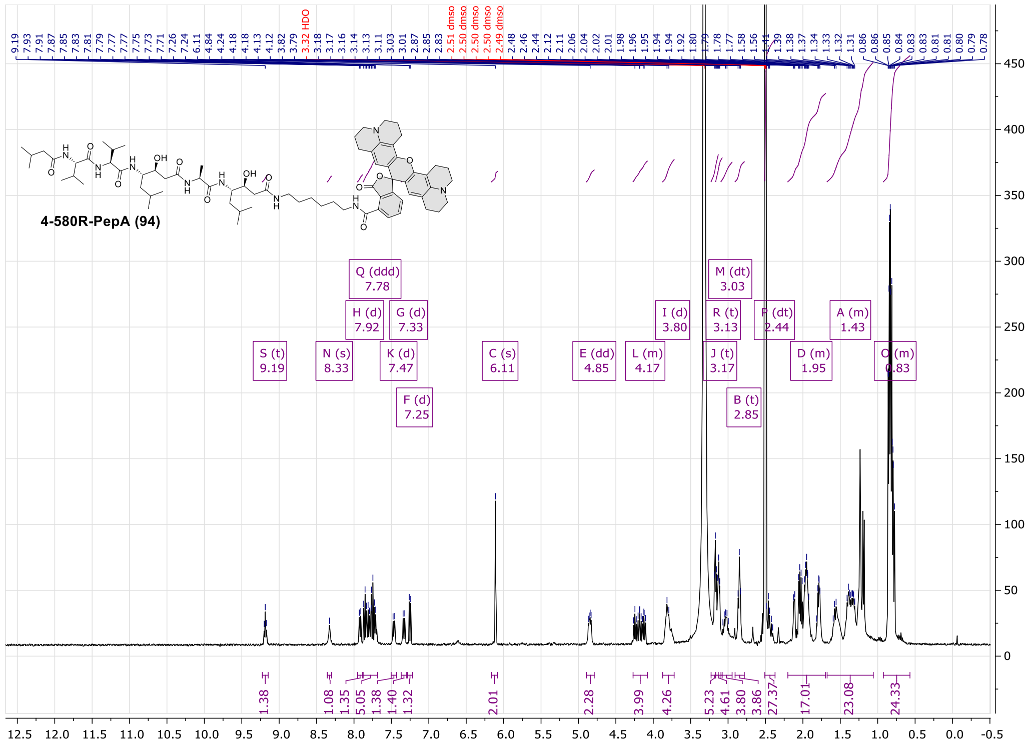

Supplementary Figure 315. ^{1}H NMR of compound **94** (400 MHz, d_6-DMSO).

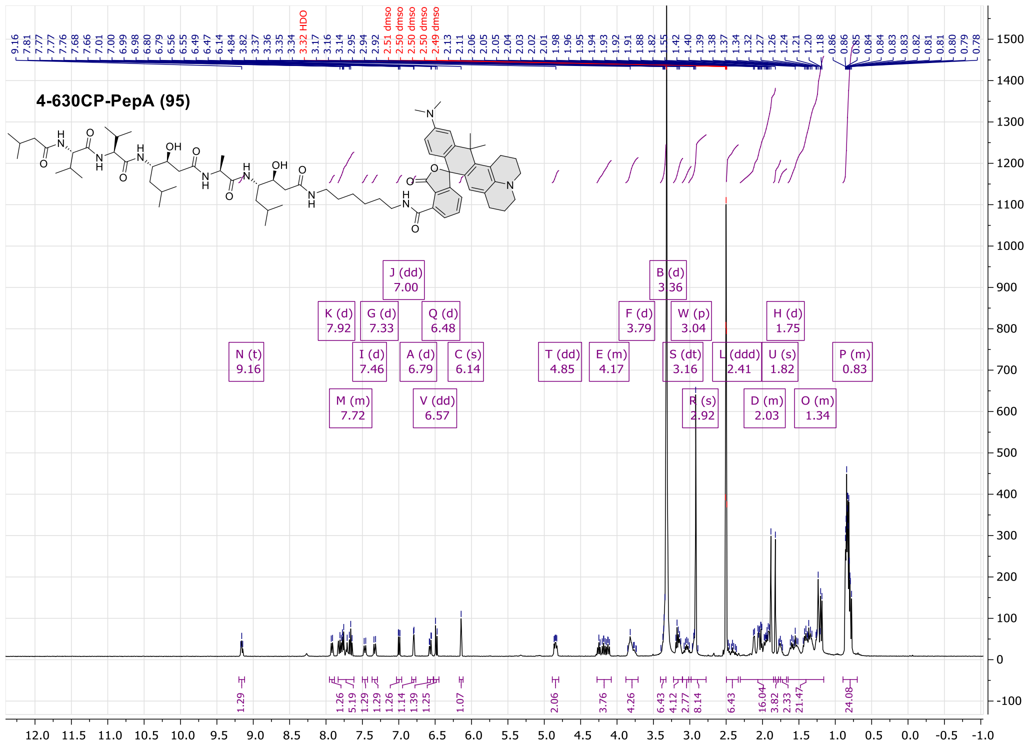

Supplementary Figure 316. ^{1}H NMR of compound **95** (400 MHz, d_6-DMSO).

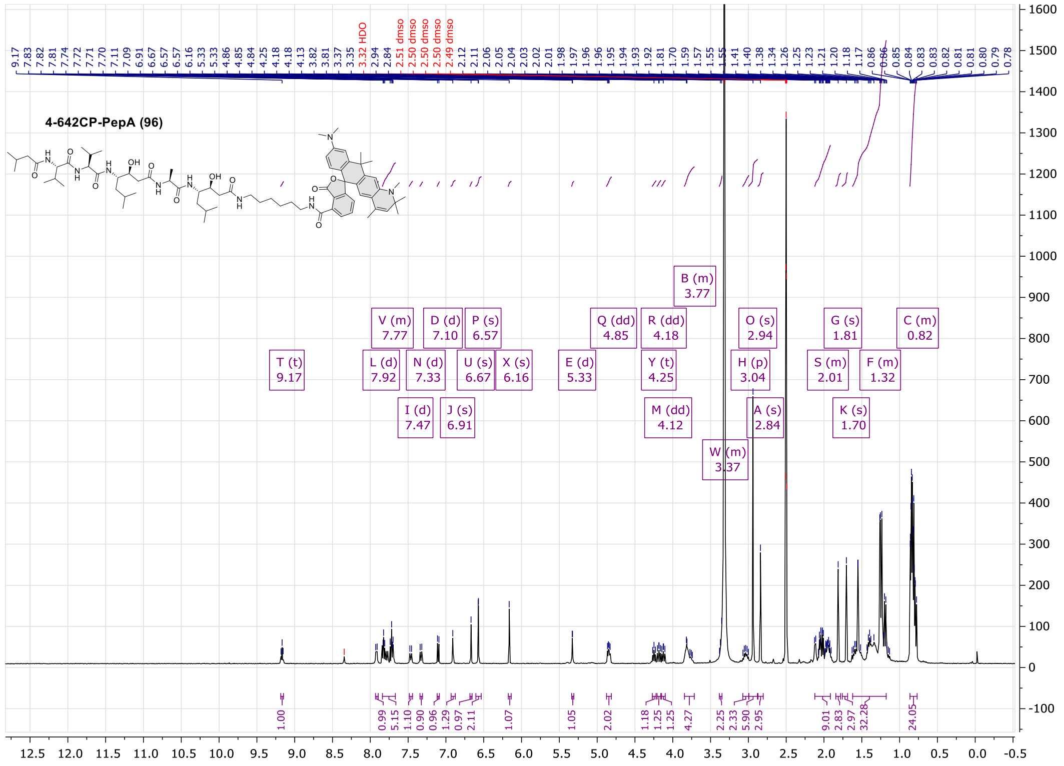

Supplementary Figure 317. ^{1}H NMR of compound **96** (400 MHz, d_6-DMSO).

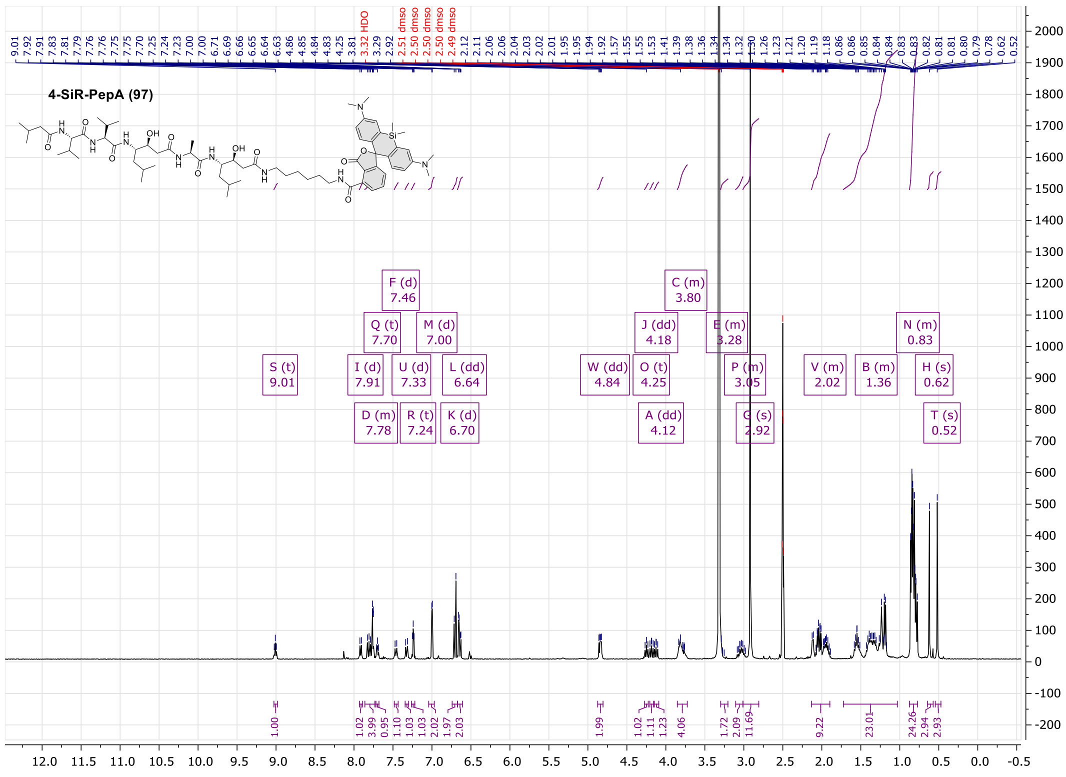

Supplementary Figure 318. ^{1}H NMR of compound **97** (400 MHz, d_6-DMSO).

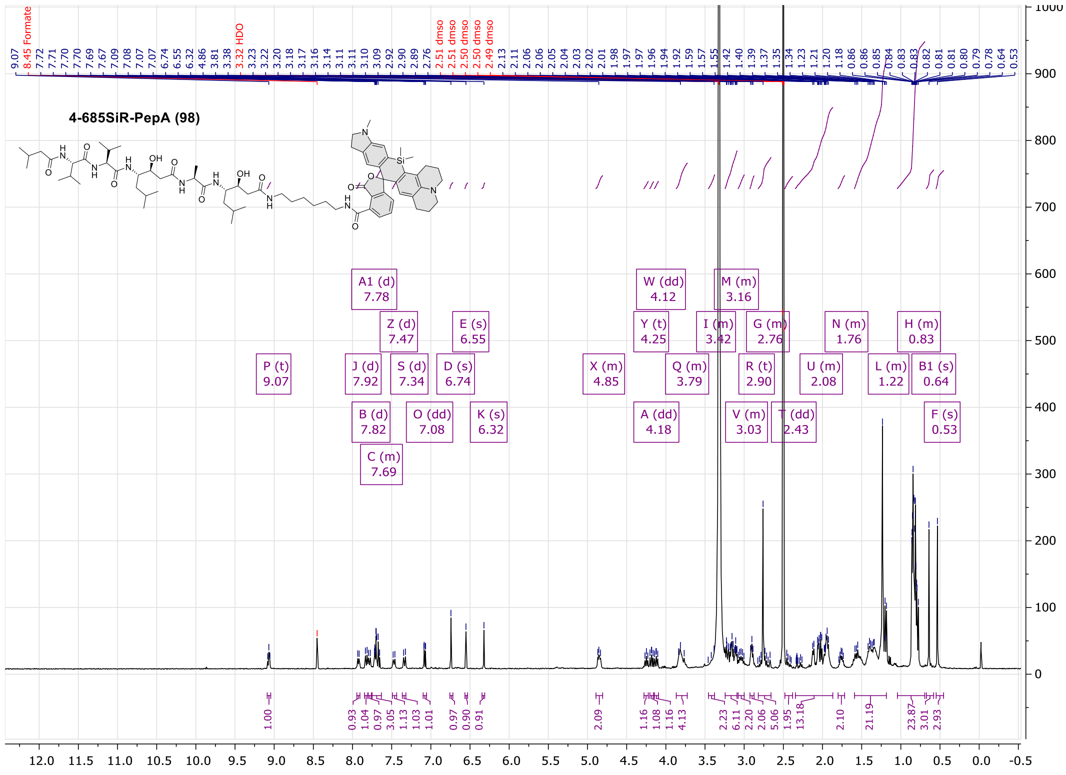

Supplementary Figure 319. ^{1}H NMR of compound **98** (400 MHz, d_6-DMSO).

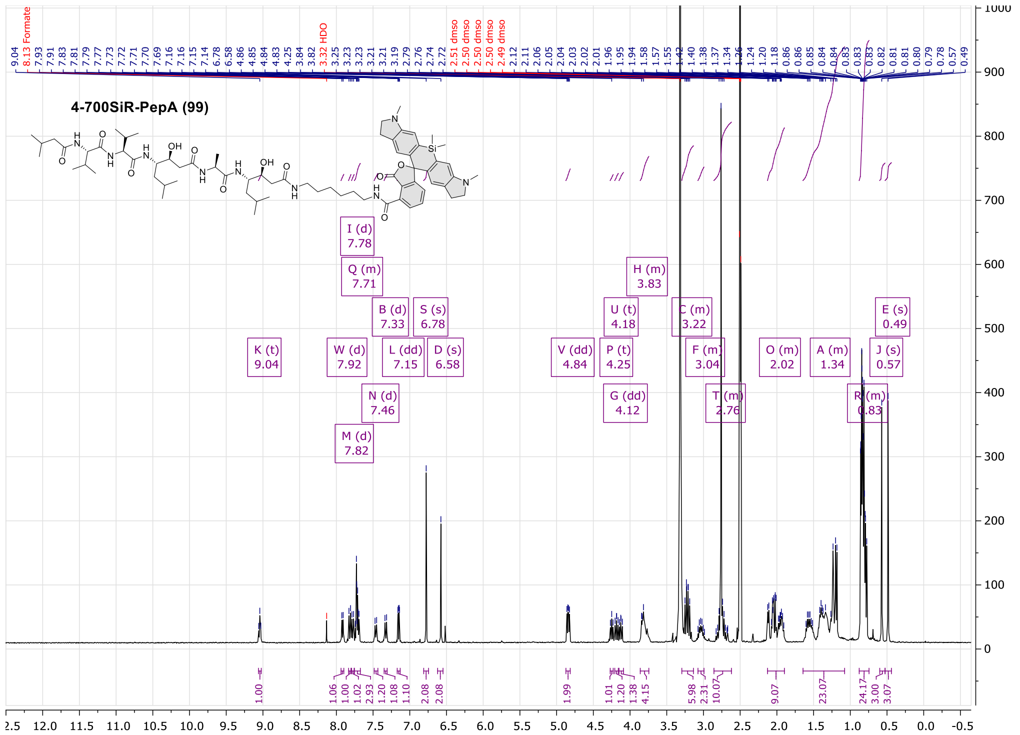

Supplementary Figure 320. ^{1}H NMR of compound **99** (400 MHz, d_6-DMSO).

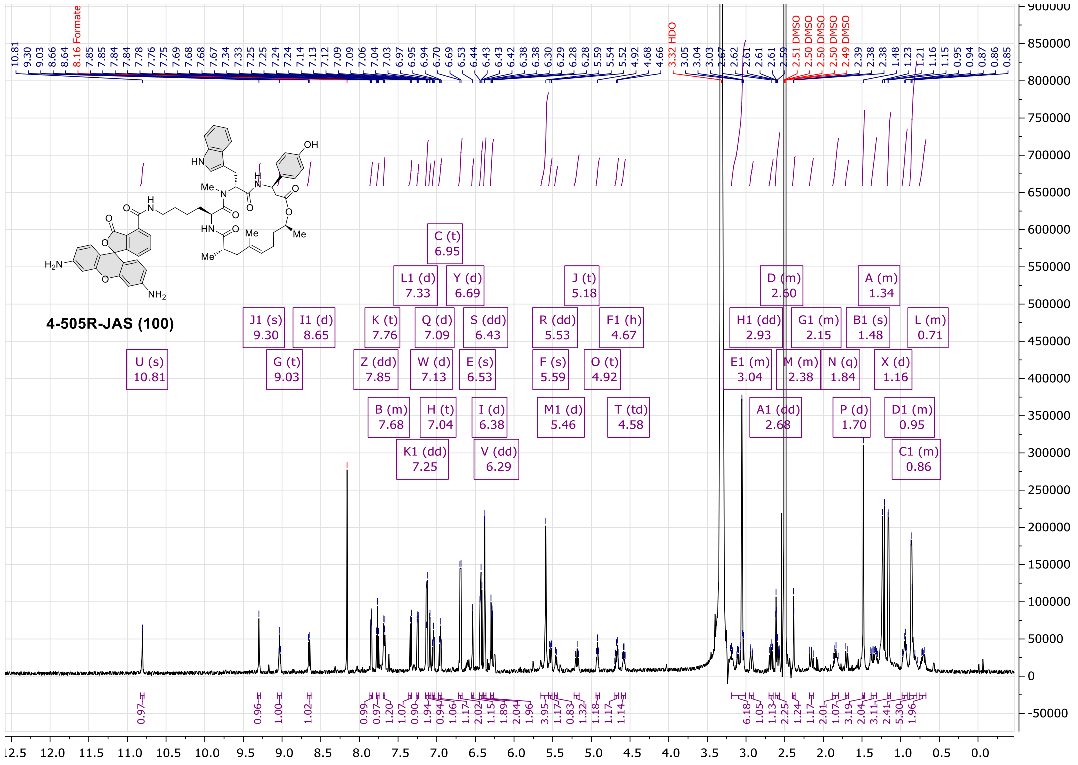

Supplementary Figure 321. ^{1}H NMR of compound **100** (400 MHz, d_6-DMSO).

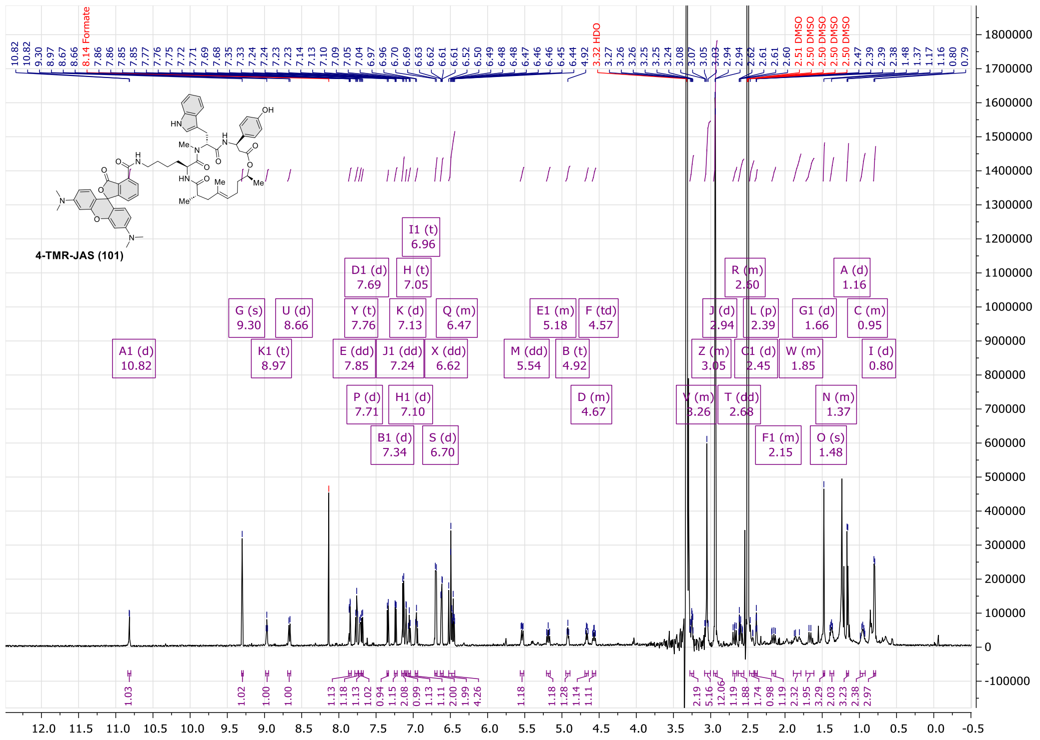

Supplementary Figure 322. ^{1}H NMR of compound **101** (400 MHz, d_6-DMSO).

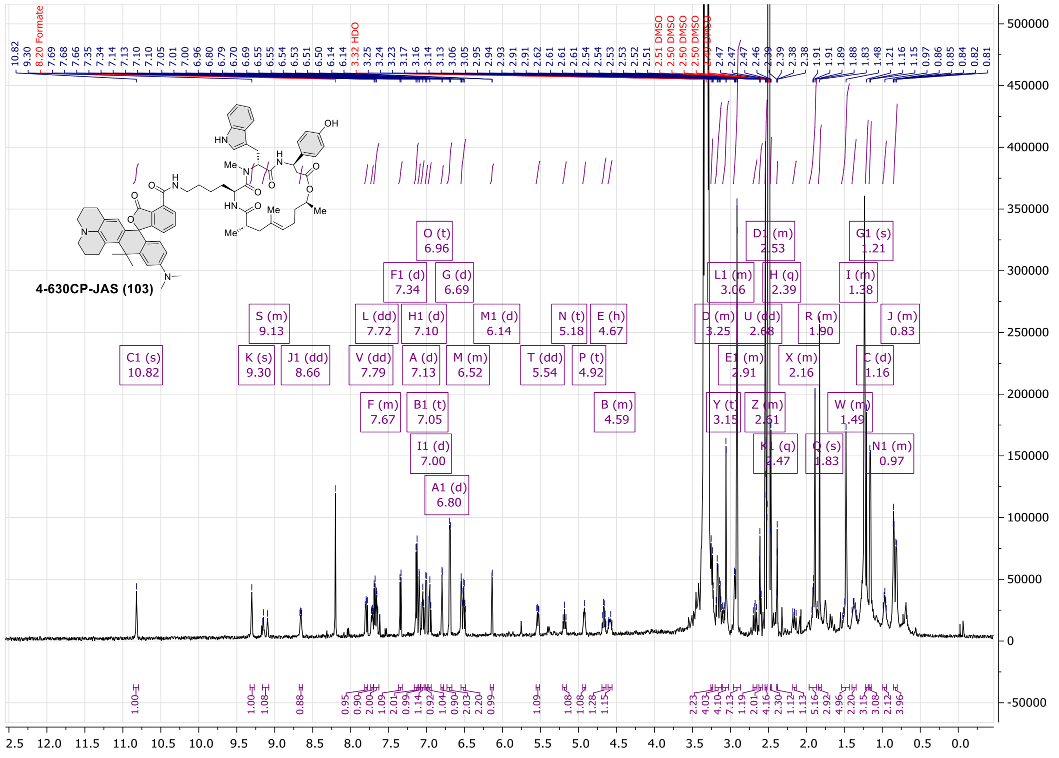

Supplementary Figure 323. ^{1}H NMR of compound **103** (400 MHz, d_6-DMSO).

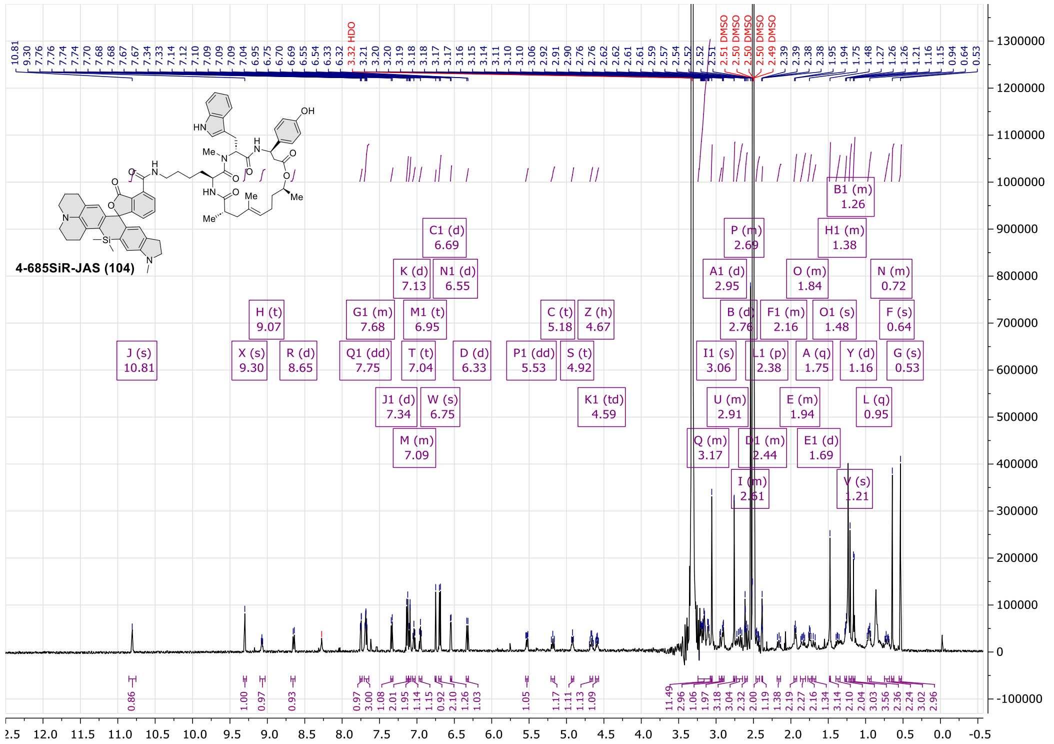

Supplementary Figure 324. ^{1}H NMR of compound **104** (400 MHz, d_6-DMSO).

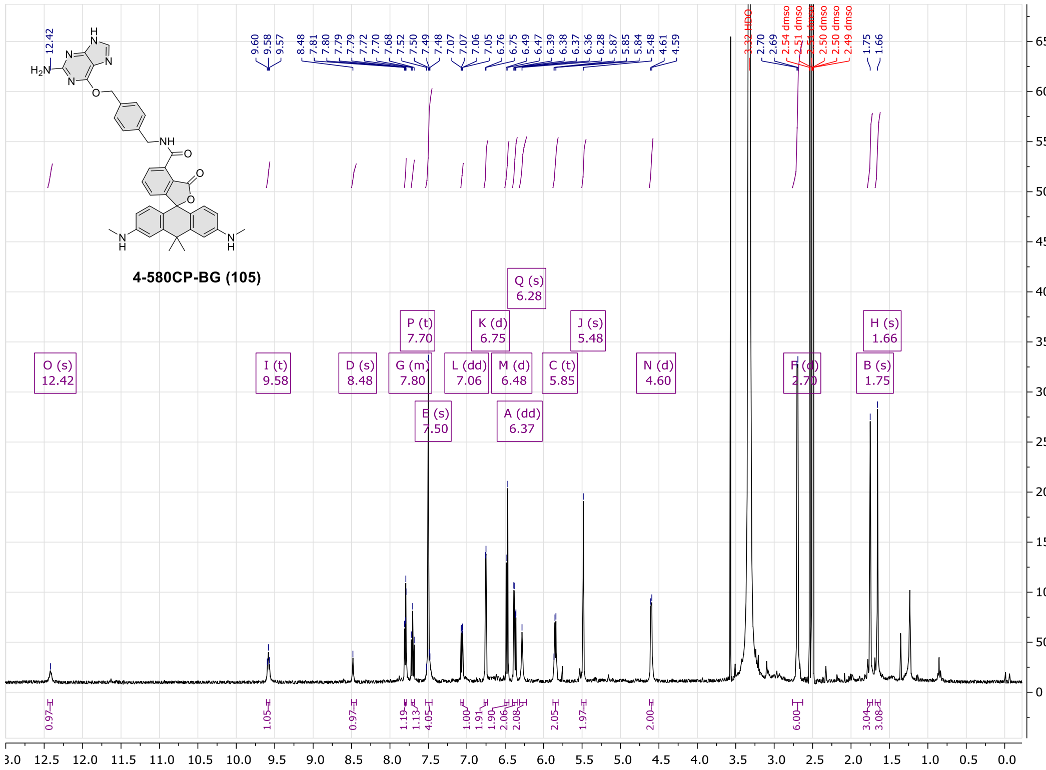

Supplementary Figure 325. ^{1}H NMR of compound **105** (400 MHz, d_6-DMSO).

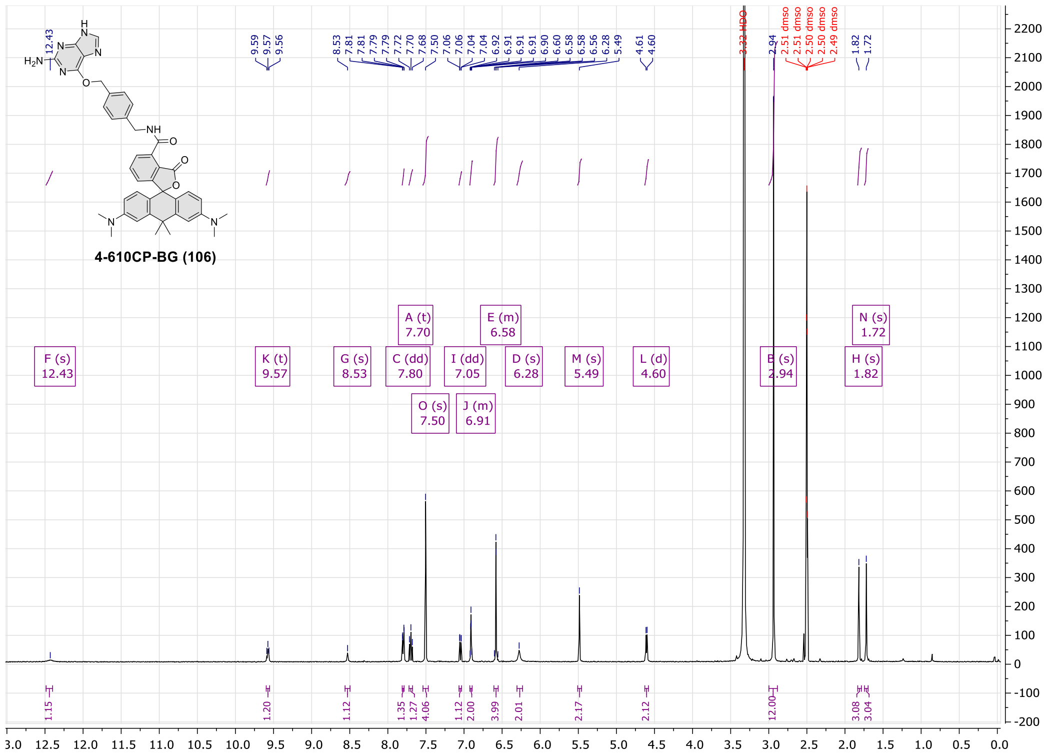

Supplementary Figure 326. ^{1}H NMR of compound **106** (400 MHz, d_6-DMSO).

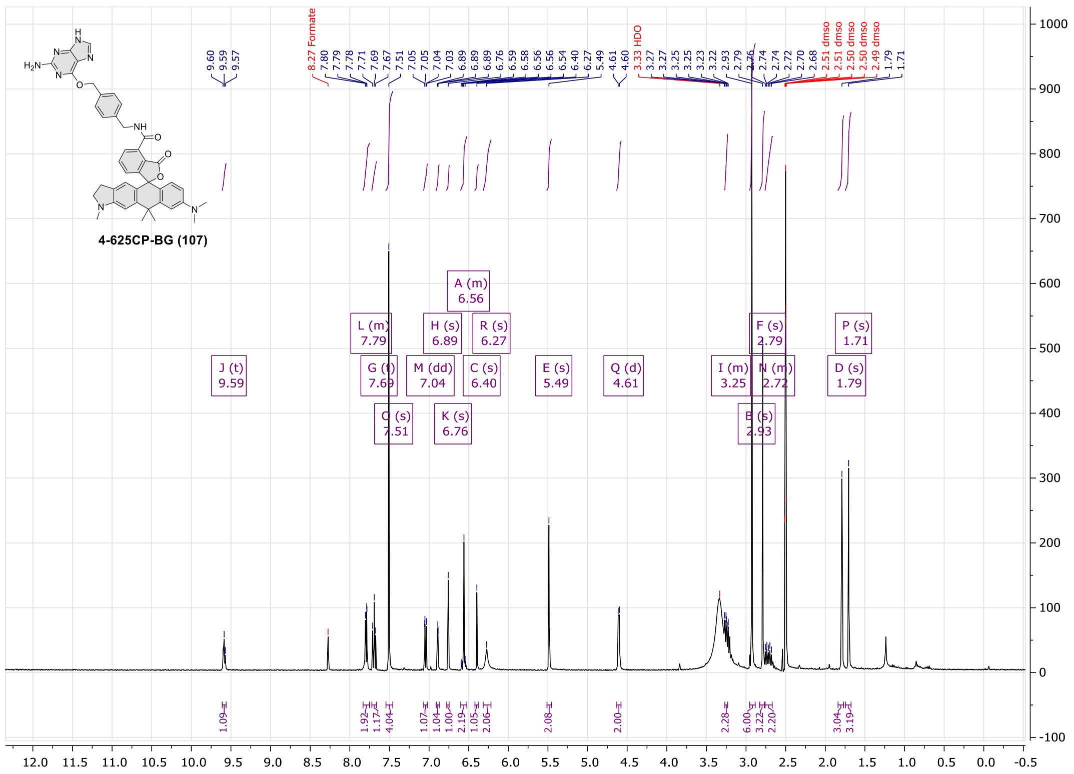

Supplementary Figure 327. ^{1}H NMR of compound **107** (400 MHz, d_6-DMSO).

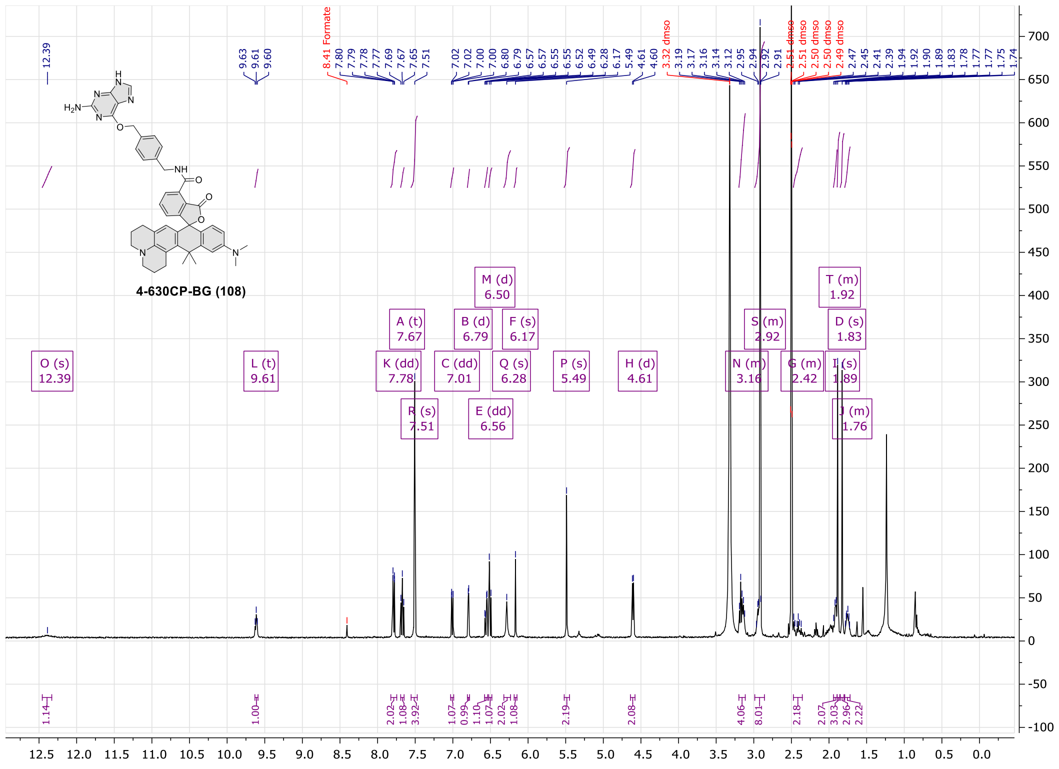

Supplementary Figure 328. ^{1}H NMR of compound **108** (400 MHz, d_6-DMSO).

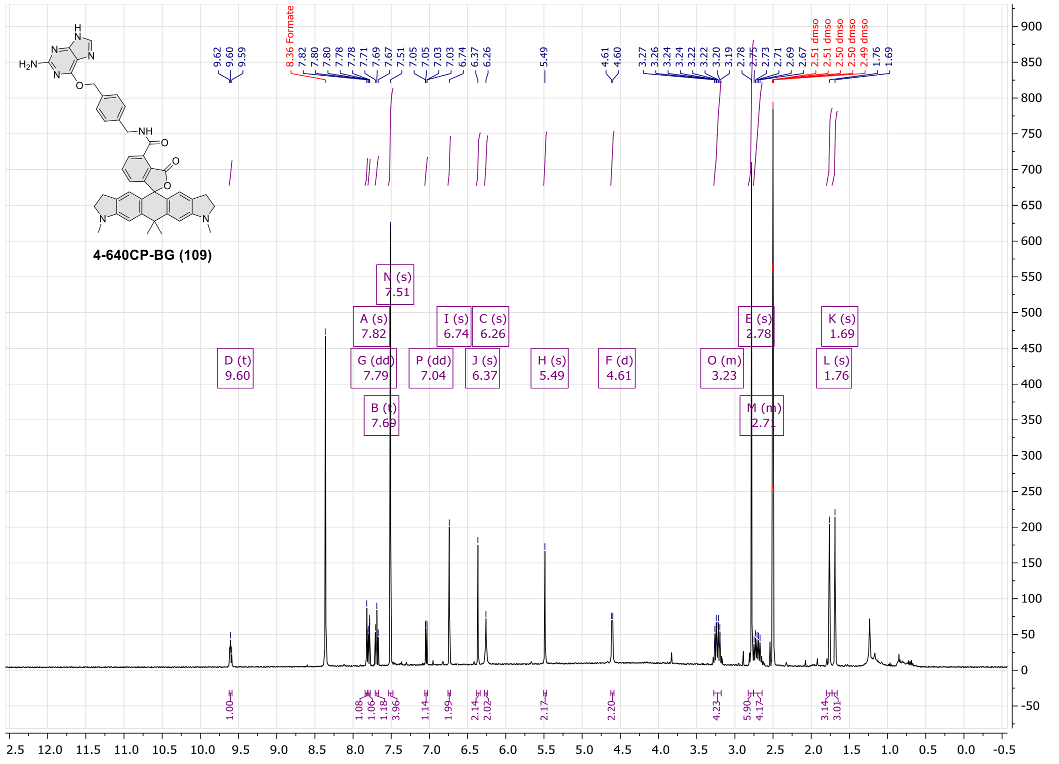

Supplementary Figure 329. ^{1}H NMR of compound **109** (400 MHz, d_6-DMSO).

Supplementary References

1 Lukinavicius, G. *et al.* Selective chemical crosslinking reveals a Cep57-Cep63-Cep152 centrosomal complex. *Curr Biol* **23**, 265-270, doi:10.1016/j.cub.2012.12.030 (2013).

2 Bach, M. *et al.* Fast set-up of doxycycline-inducible protein expression in human cell lines with a single plasmid based on Epstein-Barr virus replication and the simple tetracycline repressor. *FEBS J* **274**, 783-790, doi:10.1111/j.1742-4658.2006.05623.x (2007).

3 Ratz, M., Testa, I., Hell, S. W. & Jakobs, S. CRISPR/Cas9-mediated endogenous protein tagging for RESOLFT super-resolution microscopy of living human cells. *Sci Rep* **5**, 9592, doi:10.1038/srep09592 (2015).

4 Thevathasan, J. V. *et al.* Nuclear pores as versatile reference standards for quantitative superresolution microscopy. *Nat Methods* **16**, 1045-1053, doi:10.1038/s41592-019-0574-9 (2019).

5 D'Este, E., Kamin, D., Gottfert, F., El-Hady, A. & Hell, S. W. STED nanoscopy reveals the ubiquity of subcortical cytoskeleton periodicity in living neurons. *Cell Rep* **10**, 1246-1251, doi:10.1016/j.celrep.2015.02.007 (2015).

6 Molyneux, P. C., Pyle, L. A., Dillon, M. & Harrington, M. E. A Mouse Primary Hepatocyte Culture Model for Studies of Circadian Oscillation. *Curr Protoc Mouse Biol* **5**, 311-329, doi:10.1002/9780470942390.mo150101 (2015).

7 Schindelin, J. *et al.* Fiji: an open-source platform for biological-image analysis. *Nat Methods* **9**, 676-682, doi:10.1038/nmeth.2019 (2012).

8 Gottlieb, H. E., Kotlyar, V. & Nudelman, A. NMR Chemical Shifts of Common Laboratory Solvents as Trace Impurities. *J Org Chem* **62**, 7512-7515, doi:10.1021/jo971176v (1997).

9 Koide, Y. *et al.* Development of NIR fluorescent dyes based on Si-rhodamine for in vivo imaging. *J Am Chem Soc* **134**, 5029-5031, doi:10.1021/ja210375e (2012).

10 Gribble, G. W. & Nutaitis, C. F. Reactions of Sodium Borohydride in Acidic Media; XVI.1 N-Methylation of Amines with Paraformaldehyde/Trifluoroacetic Acid. *Synthesis* **1987**, 709-711 (1987).

11 Mayer, R. J., Hampel, N., Mayer, P., Ofial, A. R. & Mayr, H. Synthesis, Structure, and Properties of Amino-Substituted Benzhydrylium Ions – A Link between Ordinary Carbocations and Neutral Electrophiles. *European Journal of Organic Chemistry* **2019**, 412-421, doi:https://doi.org/10.1002/ejoc.201800835 (2019).

12 Li, Y. *et al.* Iron-Mediated Selective Sulfonylmethylation of Aniline Derivatives with p-Toluenesulfonylmethyl Isocyanide (TosMIC). *J Org Chem* **86**, 7179-7188, doi:10.1021/acs.joc.1c00500 (2021).

13 Pastierik, T., Sebej, P., Medalova, J., Stacko, P. & Klan, P. Near-infrared fluorescent 9-phenylethynylpyronin analogues for bioimaging. *J Org Chem* **79**, 3374-3382, doi:10.1021/jo500140y (2014).

14 Bai, L. *et al.* Novel aza-BODIPY based small molecular NIR-II fluorophores for in vivo imaging. *Chem Commun (Camb)* **55**, 10920-10923, doi:10.1039/c9cc03378e (2019).

15 Bachman, J. L., Pavlich, C. I., Boley, A. J., Marcotte, E. M. & Anslyn, E. V. Synthesis of Carboxy ATTO 647N Using Redox Cycling for Xanthone Access. *Org Lett* **22**, 381-385, doi:10.1021/acs.orglett.9b03981 (2020).

16 Lei, Z. *et al.* Synthesis of Sterically Protected Xanthene Dyes with Bulky Groups at C-3' and C-7'. *J Org Chem* **80**, 11538-11543, doi:10.1021/acs.joc.5b01746 (2015).

17 Hanaoka, K. *et al.* Synthesis of unsymmetrical Si-rhodamine fluorophores and application to a far-red to near-infrared fluorescence probe for hypoxia. *Chem Commun (Camb)* **54**, 6939-6942, doi:10.1039/c8cc02451k (2018).

18 Lukinavičius, G. *et al.* Fluorogenic Probes for Multicolor Imaging in Living Cells. *J Am Chem Soc* **138**, 9365-9368, doi:10.1021/jacs.6b04782 (2016).
19 Piao, W. *et al.* Development of azo-based fluorescent probes to detect different levels of hypoxia. *Angew Chem Int Ed Engl* **52**, 13028-13032, doi:10.1002/anie.201305784 (2013).
20 Grimm, J. B. *et al.* Carbofluoresceins and carborhodamines as scaffolds for high-contrast fluorogenic probes. *ACS Chem Biol* **8**, 1303-1310, doi:10.1021/cb4000822 (2013).
21 Jia, S., Ramos-Torres, K. M., Kolemen, S., Ackerman, C. M. & Chang, C. J. Tuning the Color Palette of Fluorescent Copper Sensors through Systematic Heteroatom Substitution at Rhodol Cores. *ACS Chem Biol* **13**, 1844-1852, doi:10.1021/acschembio.7b00748 (2018).
22 Bachman, J. L., Escamilla, P. R., Boley, A. J., Pavlich, C. I. & Anslyn, E. V. Improved Xanthone Synthesis, Stepwise Chemical Redox Cycling. *Org Lett* **21**, 206-209, doi:10.1021/acs.orglett.8b03661 (2019).
23 Wei, L. *et al.* Super-multiplex vibrational imaging. *Nature* **544**, 465-470, doi:10.1038/nature22051 (2017).
24 Sebej, P. *et al.* Fluorescein analogues as photoremovable protecting groups absorbing at approximately 520 nm. *J Org Chem* **78**, 1833-1843, doi:10.1021/jo301455n (2013).
25 Bucevičius, J., Kostiuk, G., Gerasimaitė, R., Gilat, T. & Lukinavičius, G. Enhancing the biocompatibility of rhodamine fluorescent probes by a neighbouring group effect. *Chem Sci* **11**, 7313-7323, doi:10.1039/d0sc02154g (2020).
26 Butkevich, A. N. Modular Synthetic Approach to Silicon-Rhodamine Homologues and Analogues via Bis-aryllanthanum Reagents. *Org Lett* **23**, 2604-2609, doi:10.1021/acs.orglett.1c00512 (2021).
27 Benink, H., McDougall, M., Klaubert, D. & Los, G. Direct pH measurements by using subcellular targeting of 5(and 6-) carboxyseminaphthorhodafluor in mammalian cells. *Biotechniques* **47**, 769-774, doi:10.2144/000113220 (2009).
28 Bucevičius, J., Keller-Findeisen, J., Gilat, T., Hell, S. W. & Lukinavičius, G. Rhodamine-Hoechst positional isomers for highly efficient staining of heterochromatin. *Chem Sci* **10**, 1962-1970, doi:10.1039/c8sc05082a (2019).
29 Belov, V. N. *et al.* Synthesis of Fluorescent Jasplakinolide Analogues for Live-Cell STED Microscopy of Actin. *J Org Chem* **85**, 7267-7275, doi:10.1021/acs.joc.0c00653 (2020).
