# Supplementary material for: A general highly efficient synthesis of biocompatible rhodamine dyes and probes for live-cell multicolor nanoscopy
Source: Nat Commun. 2023 Mar 9;14:1306. doi: 10.1038/s41467-023-36913-2 (PMC9998615; doi:10.1038/s41467-023-36913-2)
Supplement: Supplementary file 3 — Description of Additional Supplementary Files [file 41467_2023_36913_MOESM3_ESM.pdf]

**Description of Additional Supplementary Files**

**Supplementary Movie 1.** Human fibroblasts stained with 4-700SiR-TPP (mitochondria), 4-642CP-PepA (lysosomes), 4-DAlIR-CTX (microtubules) and 4-505R-Hoechst (nucleus).

**Supplementary Movie 2**. HUVEC cells stained with 4-700SiR-TPP (mitochondria), 4-642CP-PepA (lysosomes), 4-DAlIR-CTX (microtubules) and 4-505R-Hoechst (nucleus).

**Supplementary Movie 3**. Human fibroblasts stained with 4-700SiR-TPP (mitochondria), 4-642CP-PepA (lysosomes), 4-CFL-Hoechst (nucleus) and 4-505R-CTX (microtubules).

**Supplementary Movie 4**. U-2 OS vimentin-HaloTag cells stained with 4-700SiR-TPP (mitochondria), 4-642CP-Halo (vimentin), 4-DAlIR-CTX (microtubules) and 4-505R-Hoe (nucleus).
